# Telomeric DNA sequences in beetle taxa vary with species richness

**DOI:** 10.1038/s41598-021-92705-y

**Published:** 2021-06-25

**Authors:** Daniela Prušáková, Vratislav Peska, Stano Pekár, Michal Bubeník, Lukáš Čížek, Aleš Bezděk, Radmila Čapková Frydrychová

**Affiliations:** 1 Biology Centre of the Czech Academy of Sciences, Institute of Entomology, Branišovská 31, 370 05 České Budějovice, Czech Republic; 2 Faculty of Science, University of South Bohemia, České Budějovice, Czech Republic; 3 Department of Cell Biology and Radiobiology, Institute of Biophysics of the Czech Academy of Sciences, Brno, Czech Republic; 4 Department of Botany and Zoology, Faculty of Science, Masaryk University, Kotlářská 2, 611 37 Brno, Czech Republic

**Keywords:** Evolutionary biology, Entomology

## Abstract

Telomeres are protective structures at the ends of eukaryotic chromosomes, and disruption of their nucleoprotein composition usually results in genome instability and cell death. Telomeric DNA sequences have generally been found to be exceptionally conserved in evolution, and the most common pattern of telomeric sequences across eukaryotes is (T_x_A_y_G_z_)_n_ maintained by telomerase. However, telomerase-added DNA repeats in some insect taxa frequently vary, show unusual features, and can even be absent. It has been speculated about factors that might allow frequent changes in telomere composition in Insecta. Coleoptera (beetles) is the largest of all insect orders and based on previously available data, it seemed that the telomeric sequence of beetles varies to a great extent. We performed an extensive mapping of the (TTAGG)_n_ sequence, the ancestral telomeric sequence in Insects, across the main branches of Coleoptera. Our study indicates that the (TTAGG)_n_ sequence has been repeatedly or completely lost in more than half of the tested beetle superfamilies. Although the exact telomeric motif in most of the (TTAGG)_n_-negative beetles is unknown, we found that the (TTAGG)_n_ sequence has been replaced by two alternative telomeric motifs, the (TCAGG)_n_ and (TTAGGG)_n_, in at least three superfamilies of Coleoptera. The diversity of the telomeric motifs was positively related to the species richness of taxa, regardless of the age of the taxa. The presence/absence of the (TTAGG)_n_ sequence highly varied within the Curculionoidea, Chrysomeloidea, and Staphylinoidea, which are the three most diverse superfamilies within Metazoa. Our data supports the hypothesis that telomere dysfunctions can initiate rapid genomic changes that lead to reproductive isolation and speciation.

## Introduction

Telomeres are essential nucleoprotein structures located at the ends of linear eukaryote chromosomes. They play an indispensable role by compensating chromosome shortening, which results from the inability of conventional DNA polymerase to replicate the very end of a DNA molecule, and maintain genome stability by preventing end-to-end fusion or degradation of chromosomes^1^. Telomere length is most often maintained by a specialized RNA-dependent DNA polymerase called telomerase, which anneals to the 3′ end of chromosomes and repeatedly attaches short non-coding tandem sequences, usually 5–8-bp long, using its internal RNA subunit as a template. The activity of this enzyme is highly regulated and is generally associated with proliferating cells, such as embryonic or germ cells, while activity is low in quiescent, differentiated cells^2,3^. Telomeres distinguish natural chromosome termini from chromosomal breaks via a multiprotein structure called a telomere cap, and impairment in the telomere cap can lead to end-to-end fusions, genome instability, and cell death. Studies of ciliates, yeasts, vertebrates, and plants show that the formation of telomere caps depends on the interaction of numerous proteins, and some of the proteins bind to chromosome ends in a DNA sequence-dependent manner^4–6^. Therefore, telomeres of a satisfactory length containing a specific DNA sequence are essential to protect cell viability^7^.

Telomeric DNA sequences have generally been found to be exceptionally conserved in evolution, and the most common sequence pattern across eukaryotes is (T_x_A_y_G_z_)_n_. For example, the ancestral telomere motif of Metazoa is considered to be (TTAGGG)_n_, which is present in all basal metazoan groups, i.e., sponges, Cnidaria, Ctenophora, and Placozoa, the unicellular metazoan sister group Choanozoa, and all vertebrates^8^. In contrast, there are taxa in which telomerase-added DNA repeats frequently vary, show unusual features, or have been lost and replaced by a completely different mechanism of telomere loss compensation. For instance, telomeric repeats in closely related species of budding yeast display great diversity in length, sequence, and composition^9^. In addition, there are a number of plant families that have lost the plant-type telomeric sequence (TTTAGGG)_n_, which was replaced by a different one^10^. Regarding telomere sequence composition and elongation strategies, unusual features are seen in insects. Although the (TTAGG)_n_ sequence is considered the ancestral telomeric motif of arthropods, the sequence has been lost in numerous insect orders^11,12^. However, the uniqueness of Insecta in telomere biology is seen in dipteran species, which have no telomerase and instead employ a completely different telomere elongation system. Dipteran telomere systems involve either a co-option of non-long terminal repeat (non-LTR) retrotransposons, as observed in *Drosophila*^13,14^, or recombination of satellite sequences, as observed in chironomid midges^15^. The current review showed that the (TTAGG)_n_ telomeric sequence is either partially or completely lost in 12 of the 24 tested insect orders^16^, but the DNA sequences present at the telomeres of TTAGG-absent species are largely unknown. In fact, in addition to the dipteran retrotransposon and satellite telomeres, there is only one alternative sequence in Insecta that has been recorded: the (TCAGG)_n_ sequence in Tenebrionidae (darkling beetles)^17^.

Insects are considered to be the most evolutionary successful group of terrestrial organisms in life history, comprising more than one million species, which represents roughly 80% of the species of the world^18^. With 155,477 species^18^, Diptera is a highly successful order among insects, which shows that the loss of telomerase is not a limiting factor for insect evolution and leads us to a question how widespread non-telomerase systems and the insect telomeric motif are among insects^7^.

Coleoptera is the largest of all insect orders (386,500 species^18^), and based on available data, it seems that the telomeric sequence of beetles has evolved dynamically during their evolution. The insect telomeric motif has been lost five to six times during Coleoptera evolution^19,20^, and it has been replaced by an alternative sequence [(TCAGG)_n_] in Tenebrionoidea^17^. Furthermore, the occurrence of both sequences varies within families and even genera^17,20^. The high rate of change in beetle telomere sequences remains poorly understood. We can speculate about the factors that might allow frequent changes, such as high flexibility of the telomere capping complex, which might easily adopt new changes in the telomerase-added sequences, the sequence-independent binding of the telomere capping complex, or simply the evolution history in combination with Coleoptera diversity. However, given that beetles are the most diverse and species-rich group of insects, it should be pointed out that the sampling of representatives (14 families represented by one or two species) in previous studies appears to be inadequate and, therefore, the real variability in the presence of telomeric sequences might be highly underestimated.

The goal of our study was to understand the high rate of change in beetle telomere sequences via an extensive mapping of (TTAGG)_n_ and (TCAGG)_n_ telomeric sequences across the main branches of the order, a dramatic increase in species sampling compared to existing studies, identification of novel telomeric repeats, and interpretation of our data with the recently determined phylogenetic relationships, species richness and age of the tested clades.

## Results

### Distribution of telomeric (TTAGG) and (TCAGG) sequences

**Figure 1:**
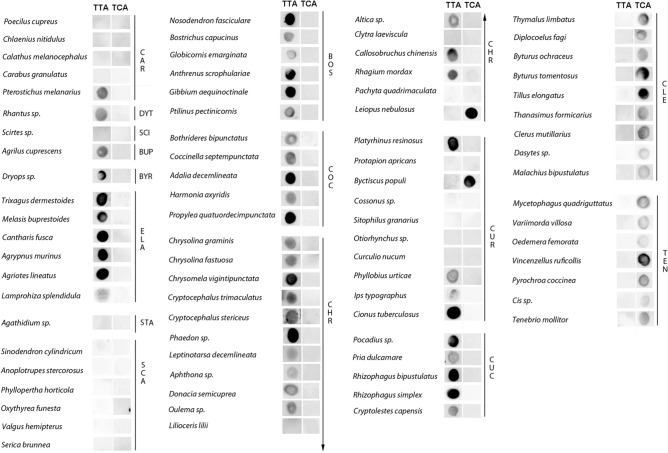
Dot-blot hybridizations. The presence of (TTAGG)_n_ and (TCAGG)_n_ sequences were examined using dot-blot hybridizations. *TTA* the (TTAGG)n sequence, *TCA* the (TCAGG)n sequence, *CAR* Caraboidea, *DYT* Dytiscoidea, *SCI* Scirtoidea, *BUP* Buprestoidea, *BYR* Byrrhoidea, *ELA* Elateroidea, *STA* Staphylinoidea, *SCA* Scarabaeoidea, *BOS* Bostrichoidea, *COC* Coccinelloidea, *CHR* Chrysomeloidea, *CUR* Curculionoidea, *CUC* Cucujoidea, *CLE* Cleroidea, *TEN* Tenebrionoidea.

**Figure 2:**
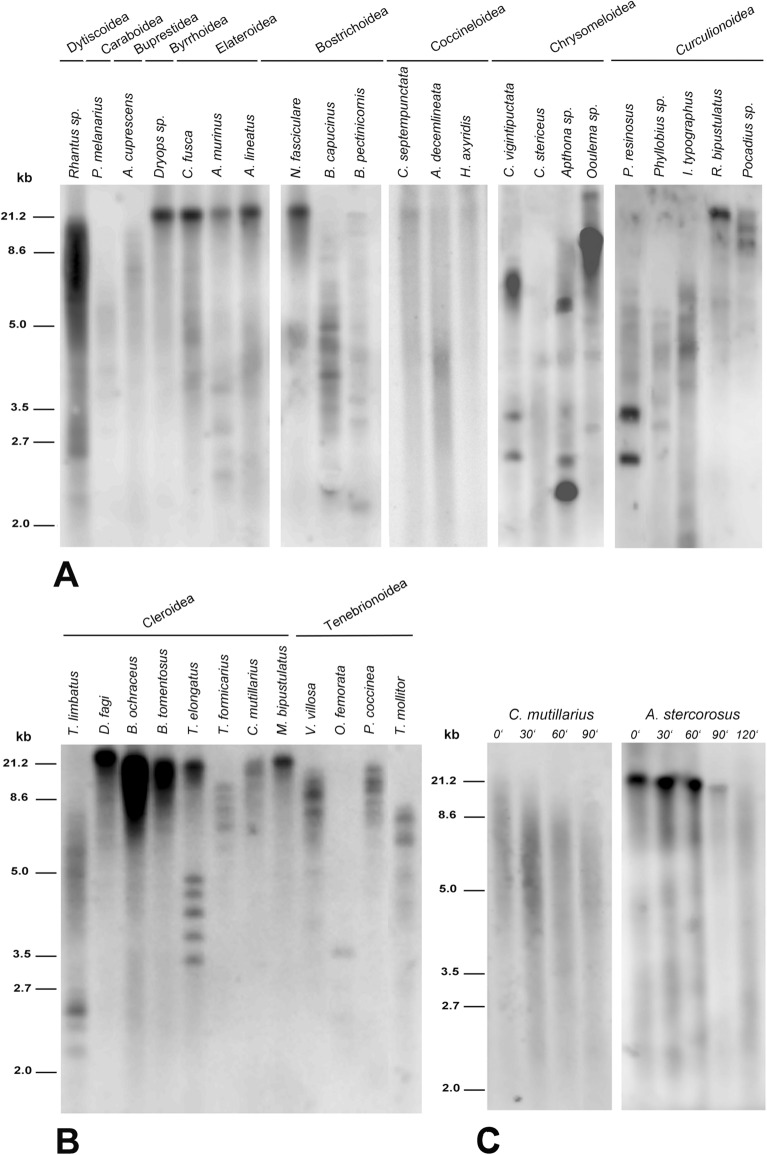
The character of hybridization signals revealed using Southern hybridization. The DIG-labeled probe specific to (TTAGG)_n_ (**a**) and (TCAGG)n (**b**) sequence was hybridized with RsaI/HinfI-digested genomic DNAs of selected coleopteran species. (**c**) Bal 31 exonuclease digestion of genomic DNA of *Anoplotrupes stercorosus* and *Clerus muttilarius.* Time-course digestion intervals are indicated above the lanes.

**Figure 3:**
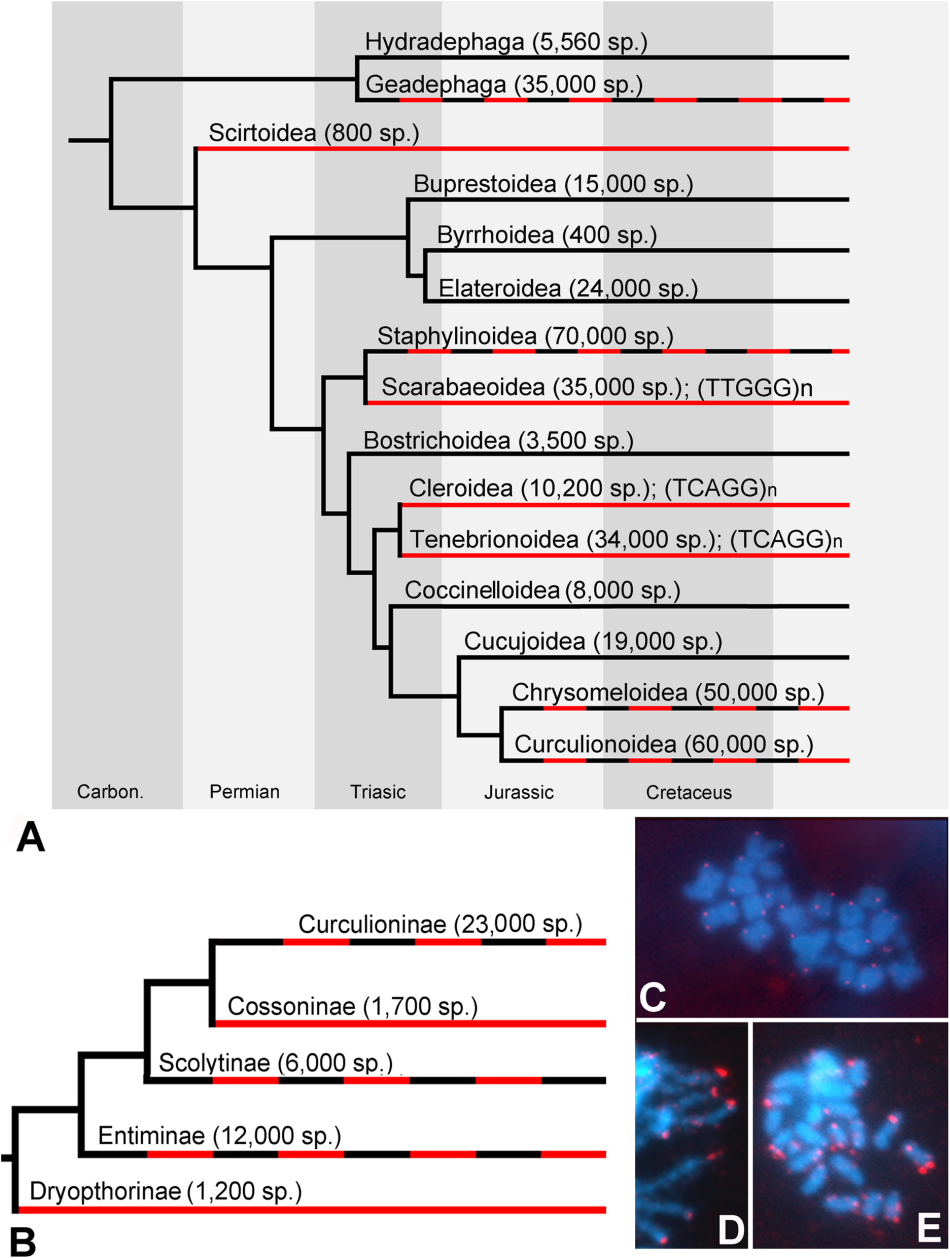
The presence of (TTAGG)_n_ telomeric sequence in Coleoptera. The (TTAGG)_n_ distribution in the whole order Coleoptera adopting the Coleoptera cladogram published by^21^ (**a**) and in the family Curculionidae based on a cladogram published by^22^ (**b**). Branch colors indicate the (TTAGG)n presence (in black), (TTAGG)n absence (in red), or variability in the (TTAGG)n presence/absence (in black and red). Approximate numbers of described extant species are indicated next to the family or subfamily level taxon names. (**c**) Fluorescence in situ hybridization with a probe against the (TCAGG)n in *Clerus mutillarius* (Cleroidea, Cleridae). (**d**, **e**) Fluorescence in situ hybridization with a probe against the (TTGGG)n in *Anoplotrupes stercorosus* (Scarabaeoidea, Geotrupidae).

**Figure 4:**
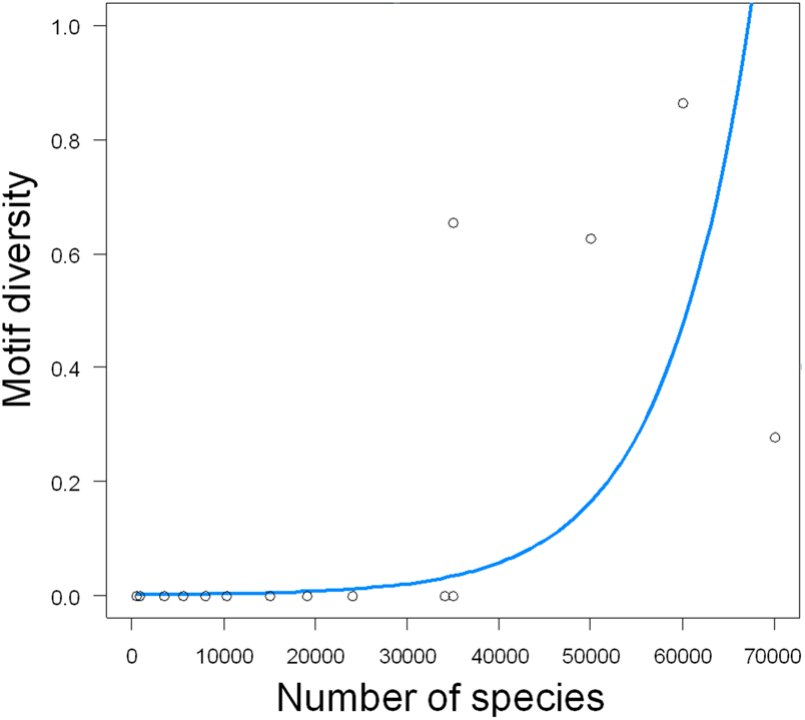
Relationship between motif diversity and species richness in taxa. The blue line represents the estimated model from GLS. The analysis was performed in the R environment (http://www.r-project.org/index.html) using R version 4.1.0^23^.

The first part of our study was an extensive survey of the distribution of (TTAGG)_n_ and (TCAGG)_n_ telomeric sequences across Coleoptera, for which we evaluated 44 families in 15 superfamilies (Table S1), covering the suborders Adephaga and Polyphaga. The mapping was performed using dot-blot hybridization (Fig. 1), Southern hybridization (Fig. 2), and by searching the National Center for Biotechnology Information (NCBI) databases for tandem repeats (Table S3). The obtained data (Table 1) were interpreted based on the recently determined phylogenetic relationships of Coleoptera (Fig. 3) and the species richness and age of taxa (Figs. 3, 4).

**Table 1:** A list of coleopteran species tested for the presence of (TTAGG)n and (TCAGG)n species.

Superfamily	Family/subfamily	Species	TTAGG	TCAGG	Source
**Caraboidea**	**Carabidae**				
	Harpalinae	*Selenophorus alternans*	(−)^a^	(−)^a^	
	Licinae	*Chlaenius nitidulus*	(−)	(−)	
	Cicindelinae	*Tetracha* sp*.*	(−)^a^	(−)^a^	
	Carabinae	*Carabus granulatus*	(−)	(−)	
	Platyninae	*Calathus melanocephalus*	(−)	(−)	
		*Miquihuana rhandiniformis*	(+)^a^	(−)^a^	
	Trechinae	*Bembidarenas* sp*.*	(+)^a^	(−)^a^	
		*Bembidion chilioperyphus*	(+)^a^	(−)^a^	
		*B. orion*	(+)^a^	(−)^a^	
		*B. castor*	(+)^a^	(−)^a^	
		*B. aeruginosum*	(−)^a^	(−)^a^	
		*B. musae*	(+)^a^	(−)^a^	
		*B. clemens*	(+)^a^	(−)^a^	
		*B. breve*	(−)^a^	(−)^a^	
		*B. laxatum*	(−)^a^	(−)^a^	
		*B. obscuripenne*	(−)^a^	(−)^a^	
		*B. lapponicum*	(−)^a^	(−)^a^	
		*B. flohri*	(+)^a^	(−)^a^	
		*B. scenicum*	(+)^a^	(−)^a^	
		*B. lachnophoroides*	(+)^a^	(−)^a^	
		*B. ulkei*	(+)^a^	(−)^a^	
		*B. subfusum*	(+)^a^	(−)^a^	
		*B. aeruginosum*	(+)^a^	(−)^a^	
		*B. eupetedromus*	(+)^a^	(−)^a^	
		*Lionepha casta*	(+)^a^	(−)^a^	
		*L. chintimini*	(+)^a^	(−)^a^	
		*L. erasa*	(−)^a^	(−)^a^	
		*L. lindrothellus*	(+)^a^	(−)^a^	
		*Pogonus chalceus*	(+)^a^	(−)^a^	
	Pterostichinae	*Pterostichus melanarius*	(+)	(−)	
		*P. oblongopunctatus*	(−)	Untested	^b^
		*Pseudamara arenaria*	(+)^a^	(−)^a^	
		*Poecilus cupreus*	(−)	(−)	
**Dytiscoidea**	**Dytiscidae**				
	Colymbetinae	*Rhantus* sp*.*	(+)	(−)	
	Hydroporinae	*Stictotarsus aequinoctialis*	(+)^a^	(−)^a^	
		*Graphoderus cinereus*	(+)	Untested	^b^
	Gyrinidae:	*Orectochilus villosus*	(−)	Untested	^b^
**Scirtoidea**	**Scirtidae**: Scirtinae	*Scirtes* sp*.*	(−)	(−)	
**Buprestoidea**	**Buprestidae**				
	Agrilinae	*Agrilus cuprescens*	(+)	(−)	
		*Agrilus planipennis*	(+)^a^	(−)^a^	
**Byrrhoidea**	**Dryopidae**	*Dryops* sp*.*	(+)	(−)	
**Elateroidea**	**Throscidae:** Throscinae	*Trixagus dermestoides*	(+)	(−)	
	**Eucnemidae**: Melasinae	*Melasis buprestoides*	(+)	(−)	
	**Cantharidae:** Cantharinae	*Cantharis fusca*	(+)	(−)	
	**Elateridae**				
	Elaterinae	*Agriotes lineatus*	(+)	(−)	
		*Ampedus sanguineus*	(+)	Untested	^b^
	Dentrometrinae	*Diacanthous undosus*	(+)	Untested	^c^
	Agrypninae	*Agrypnus murinus*	(+)	(−)	
	Melanotinae	*Melanotus legatus*	(+)	Untested	^c^
	**Lampyridae**				
	Lampyrinae	*Lamprohiza splendidula*	(+)	(−)	
		*Ellychnia corrusca*	(+)^a^	(−)^a^	
		*Lucidota atra*	(+)^a^	(−)^a^	
		*L. punctata*	(+)^a^	(−)^a^	
		*Phausis reticulata*	(+)^a^	(−)^a^	
		*Photinus australis*	(+)^a^	(−)^a^	
		*P. brimleyi*	(+)^a^	(−)^a^	
		*P. carolinus*	(+)^a^	(−)^a^	
		*P. cooki*	(+)^a^	(−)^a^	
		*P. indictus*	(+)^a^	(−)^a^	
		*P. macdermotti*	(+)^a^	(−)^a^	
		*P. obscurellus*	(+)^a^	(−)^a^	
		*P. scintillans*	(+)^a^	(−)^a^	
		*P. pyralis*	(+)^a^	(−)^a^	
		*Pyractomena borealis*	(+)^a^	(−)^a^	
		*Pyractomena angulata*	(+)^a^	(−)^a^	
		*Pyractomena. marginalis*	(+)^a^	(−)^a^	
		*Pyropyga decipiens*	(+)^a^	(−)^a^	
	Luciolinae	*Aquatica lateralis*	(+)^a^	(−)^a^	
	Photurinae	*Photuris frontalis*	(+)^a^	(−)^a^	
**Staphylinoidea**	**Leiodidae**				
	Leiodinae	*Agathidium* sp*.*	(−)	(−)	
	**Silphidae**	*Silpha obscura*	(+)	Untested	^b^
		*Nicrophorus orbicollis*	(+)^a^	(−)^a^	
	**Staphylinidae**				
	Pselaphinae	*Adranes taylori*	(+)^a^	(−)^a^	
	Oxytelinae	*Carpelimus* sp*.*	(−)^a^	(−)^a^	
	Aleocharinae	*Aenictocupidus jacobsonorum*	(+)^a^	(−)^a^	
		*Mimaenictus wilsoni*	(+)^a^	(−)^a^	
		*Weissflogia rhopalogaster*	(+)^a^	(−)^a^	
		*Dalotia coriaria*	(+)^a^	(−)^a^	
		*Earota dentata*	(+)^a^	(−)^a^	
		*Diploeciton nevermanni*	(+)^a^	(−)^a^	
		*Deinopsis erosa*	(+)^a^	(−)^a^	
		*Dorylogaster longipes*	(+)^a^	(−)^a^	
		*Ecitomorpha arachnoides*	(+)^a^	(−)^a^	
		*Ecitophya simulans*	(+)^a^	(−)^a^	
		*Drusilla canaliculata*	(+)^a^	(−)^a^	
		*Ecitoglossa quadriceps*	(+)^a^	(−)^a^	
		*Platyusa sonomae*	(+)^a^	(−)^a^	
		*Tetradonia laticeps*	(+)^a^	(−)^a^	
		*Labidoglobus nevermanni*	(+)^a^	(−)^a^	
		*L. appendiculatus*	(+)^a^	(−)^a^	
		*Pseudomimeciton antennatum*	(+)^a^	(−)^a^	
		*Oxypoda opaca*	(+)^a^	(−)^a^	
		*Sceptobius lativentris*	(+)^a^	(−)^a^	
	Tachyporinae	*Coproporus ventriculus*	(−)^a^	(−)^a^	
**Scarabaeoidea**	**Geotrupidae**	*Anoplotrupes stercorosus*	(−)	(−)	
		*Geotrupes stercorarius*	(−)	Untested	^b^
	**Scarabaeidae**				
	Rutelinae	*Phyllopertha horticola*	(−)	(−)	
		*Chrysina resplendens*	(−)^a^	(−)^a^	
		*Popillia japonica*	(−)^a^	(−)^a^	
	Valginae	*Valgus hemipterus*	(−)	(−)	
	Dynastinae	*Oryctes borbonicus*	(−)^a^	(−)^a^	
	Scarabaeinae	*Canthidium* sp*.*	(−)^a^	(−)^a^	
		*Onthophagus taurus*	(−)^a^	(−)^a^	
		*Pachysoma endroedyi*	(−)^a^	(−)^a^	
		*P. striatus*	(−)^a^	(−)^a^	
	Sericinae	*Serica brunnea*	(−)	(−)	
	Cetoniinae	*Oxythyrea funesta*	(−)	(−)	
	**Lucanidae**				
	Syndesinae	*Sinodendron cylindricum*	(−)	(−)	
**Bostrichoidea**	**Bostrichidae:** Bostrichinae	*Bostrichus capucinus*	(+)	(−)	
	**Anobiidae:** Anobiinae	*Stegobium paniceum*	(+)	Untested	^b^
	**Dermestidae**				
	Megatominae	*Globicornis emarginata*	(+)	(−)	
		*Anthrenus scrophulariae*	(+)	(−)	
	**Ptinidae**				
	Gibiinae	*Gibbium aequinoctinale*	(+)	(−)	
	Ptilininae	*Ptilinus pectinicornis*	(+)	(−)	
	**Nosodendridae:**	*Nosodendron fasciculare*	(+)	(−)	
**Tenebrionoidea**	**Mordellidae:** Mordellinae	*Variimorda villosa*	(−)	(+)	
	**Oedemeridae:** Oedemerinae	*Oedemera femorata*	(−)	(+)	
	**Salpingidae:** Salpinginae	*Vincenzellus ruficollis*	(−)	(+)	
	**Pyrochroidae:** Pyrochroinae	*Pyrochroa coccinea*	(−)	(+)	
	**Mycetophagidae**				
	Mycetophaginae	*Mycetophagus sp.*	(−)	(+)	
		*Typhaea stercorea*	(−)	(+)	^d^
	**Ciidae:** Ciinae	*Cis* sp*.*	(−)	(+)	
	**Tenebrionidae**				
	Tenebrioninae	*Tenebrio mollitor*	(−)	(+)	
		*Phylacinus fisheri*	(−)^a^	(+)^a^	
		*Tribolium castaneum*	(−)^a^	(+)^a^	^d^
		*T. freemani*	(−)	(+)	^d^
		*T. confosum*	(−)	(+)	^d^
		*T. audax*	(−)	(+)	^d^
		*T. brevicornis*	(−)	(+)	^d^
		*T. anaphe*	(−)	(+)	^d^
		*T. destructor*	(−)	(+)	^d^
	Pimeliinae	*Pimelia elevata*	(−)	(+)	^d^
		*P. cribra*	(−)	(+)	^d^
	**Meloidae**				
	Meloinae	*Hycleus cichorii*	(−)^a^	(+)^a^	
		*H. phaleratus*	(−)^a^	(+)^a^	^d^
	**Anthicidae**	Anthicidae sp*.*	(−)^a^	(+)^a^	
**Cleroidea**	**Melyridae**				
	Malachiinae	*Malachius bipustulatus*	(−)	(+)	
	Dasytinae	*Dasytes* sp*.*	(−)	(+)	
	**Cleridae**				
	Clerinae	*Clerus mutillarius*	(−)	(+)	
		*Thanasimus formicarius*	(−)	(+)	
	Tillinae	*Tillus elongatus*	(−)	(+)	
	**Trogossitidae:** Peltinae	*Thymalus limbatus*	(−)	(+)	
	**Biphyllidae**	*Diplocoelus fagi*	(−)	(+)	
	**Byturidae**				
	Byturinae	*Byturus ochraceus*	(−)	(+)	
		*B. tomentosus*	(−)	(+)	
**Coccinelloidea**	**Bothrideridae:** Bothriderinae	*Bothrideres bipunctatus*	(+)	(−)	
	**Coccinellidae**				
	Coccinellinae	*Coccinella septempunctata*	(+)	(−)	
		*Adalia decemlineata*	(+)	(−)	
		*Harmonia axyridis*	(+)^e^	(−)^e^	
		*Propylea quatuordecimpunctata*	(+)	(−)	
		*Eriopis connexa*	(+)^a^	(−)^a^	
	Scymninae	*Nephaspis* sp*.*	(+)^a^	(−)^a^	
**Cucujoidea**	**Monotomidae**				
	Rhizophaginae	*Rhizophagus bipustulatus*	(+)	(−)	
		*R. simplex*	(+)	(−)	
	**Nitidulidae**				
	Meligethinae	*Pria dulcamare*	(+)	(−)	
	Nitidulinae	*Pocadius* sp*.*	(+)	(−)	
	**Silvanidae:** Silvaninae	*Oryzaephilus surinamensis*	(+)	(−)	^d^
	**Cucujidae:** Laemophloinae	*Cryptolestes capensis*	(+)	(−)	
**Chrysomeloidea**	**Chrysomelidae**				
	Chrysomelinae	*Chrysolina graminis*	(+)	(−)	
		*C. fastuosa*	(+)	(−)	
		*C. polita*	(+)	Untested	^d^
		*C. americana*	(+)	Untested	^d^
		*C. herbacea*	(+)	Untested	^d^
		*Chrysomela vigintipunctata*	(+)	(−)	
		*Plagiodera versicola*	(+)^a^	(−)^a^	
		*Phaedon* sp*.*	(+)	(−)	
		*Gonioctena quinquepunctata*	(+)^a^	(−)^a^	
		*Leptinotarsa decemlineata*	(+)^e^	(−)^e^	
	Bruchinae	*Callosobruchus chinensis*	(+)^e^	(−)^e^	
		*Acanthoscelides obtectus*	(+)	Untested	^d^
	Cryptocephalinae	*Cryptocephalus trimaculatus*	(+)	(−)	
		*C. stericeus*	(+)	(−)	
	Eumolpinae	*Colaspis* sp*.*	(+)^a^	(−)^a^	
	Galerucinae	*Diabrotica barberi*	(−)^a^	(−)^a^	
		*D. virgifera*	(−)^a^	(−)^a^	
		*Aphthona* sp*.*	(+)	(−)	
		*Altica* sp*.*	(+)	(−)	
	Donaciinae	*Donacia semicuprea*	(+)	(−)	
	Criocerinae	*Lilioceris lilii*	(−)	(−)	
		*Oulema* sp*.*	(+)	(−)	
	Clytrinae	*Clytra laeviscula*	(−)	(−)	
	**Cerambycidae**				
	Lepturinae	*Pachyta quadrimaculata*	(−)	(−)	
		*Rhagium mordax*	(+)	(−)	
		*Rhagium inquisitor*	(+)	Untested	^d^
	Lamiinae	*Leiopus nebulosus*	(−)	(+)	
		*Anoplophora glabripennis*	(−)^a^	(−)^a^	
	Cerambycinae	*Phymatodes lengi*	(+)^a^	(−)^a^	
	Aseminae	*Arhopalus coreanus*	(+)	Untested	^c^
	Spondylinae	*Spondylis buprestoides*	(+)	Untested	^c^
**Curculionoidea**	**Anthribidae**	*Platyrhinus resinosus*	(+)	(−)	
	**Attelabidae:** Rhynchitinae	*Byctiscus populi*	(−)	(+)	
	**Brentidae**	*Protapion apricans*	(−)	(−)	
	**Curculionidae**				
	Entiminae	*Otiorhynchus* sp*.*	(−)	(−)	
		*Phyllobius* sp*.*	(+)	(−)	^d^
		*Phyllobius urticae*	(+)	(−)	
	Dryophthorinae	*Sitophilus granarius*	(−)	(−)	
	Cossoninae	*Cossonus* sp*.*	(−)	(−)	
	Curculioninae	*Curculio nucum*	(−)	(−)	
		*Cionus tuberculosus*	(+)	(−)	
		*C. nigritarsis*	(−)	(−)	^d^
		*Anthonomus grandis*	(−)^a^	(−)^a^	
	Scolytinae	*Dendroctonus jeffreyi*	(−)^a^	(−)^a^	
		*D. ponderosae*	(−)^a^	(−)^a^	
		*Hypothenemus hampei*	(+)^a^	(−)^a^	
		*Ips typographus*	(+)	(−)	

(+) signal present, (−) signal absent.

^a^Data extracted from Database.

^b^Data from Frydrychova et Marec^20^.

^c^Data from Okazaki et al.^19^.

^d^Data from Mravinac et al.^17^.

^e^Data based on the NCBI and dot-blot hybridization.

In Adephaga, we targeted representatives of Carabidae (Geadephaga), which is the family covering the majority of adephagan species^21,24–26^. Using dot-blot hybridization, we tested representatives of four subfamilies (Carabinae, Pterostichinae, Licininae, and Platyninae), and the presence of (TTAGG)_n_ was detected only in one representative of two tested species of Pterostichinae. A further search of Carabidae was conducted using the NCBI database. In Trechinae, the data showed (TTAGG)_n_ in 17 species and the absence of this sequence in six species, and (TTAGG)_n_ was absent in Cicindelinae and Harpalinae species. The (TCAGG)_n_ sequence was not found in any of the Carabidae species examined. Next, we detected (TTAGG)_n_ in *Rhantus* sp. (Colymbetinae, Dytiscidae) using dot blot hybridization and in Hydroporinae (Dytiscidae, Hydradephaga) in the NCBI database.

We tested one species of Scirtoidea (*Scirtes* sp., Scirtidae), which is a small superfamily of beetles with a basal position within Polyphaga, sharing archaic morphological features with Archostemata and Adephaga^21,27,28^. Scirtoidea species displayed no hybridization signal for either of the tested sequences.

All tested Elateriformia species representing Throscidae, Eucnemidae, Cantharidae, Elateridae, and Lampyridae showed the presence of (TTAGG)_n_ and absence of (TCAGG)_n_ motif by dot-blot hybridization or the NCBI search.

Next, we investigated Staphylinoidea, which includes the majority of the Staphylinoformia species, and we tested subfamilies of Staphylinidae and Leiodidae, which are the two largest Staphylinoidea families^29^. DNA hybridization experiments revealed the absence of the (TTAGG)_n_ motif in Leiodinae (Leiodidae), and the NCBI search showed its absence in Oxytelinae and Tachyporinae (both Staphylinidae). In contrast, based on the NCBI search, 19 species of Aleocharinae were found to be (TTAGG)-positive. The (TCAGG)_n_ sequence was not found in any of the examined species. Together with the published data^20^, we can conclude that Staphylinoidea varies in the presence/absence of the (TTAGG)_n_ motif.

Scarabaeoidea is the only superfamily of Scarabaeiformia, herein represented by Geotrupidae, Lucanidae, and Scarabaeidae. None of the tested representatives displayed the presence of either (TTAGG)_n_ or (TCAGG)_n_.

Cucujiformia is an infraorder represented by seven superfamilies (Coccinelloidea, Cucujoidea, Chrysomeloidea, Curculionoidea, Cleroidea, Lymexyloidea, and Tenebrionoidea)^30^. We detected the (TCAGG)_n_ as the alternative telomeric motif in Tenebrionoidea families: Pyrochroidae, Meloidae, Salpingidae, Oedemeridae, Mordellidae, Ciidae, Mycetophagidae, Tenebrionidae, and Anthicidae. Moreover, our data showed that the (TTAGG)_n_ sequence had been replaced with the (TCAGG)_n_ motif in all representatives of the Cleroidea superfamily, which has been recently reported as a sister group of Tenebrionoidea^21^. The tested species of Cleoridea represented Melyridae, Cleridae, and Trogossitidae as well as Biphyllidae and Byturidae, two additional families recently transferred into Cleroidea^28,31^. The presence of the (TCAGG)_n_ motif at the chromosome termini of Cleroidea was confirmed using Bal31 digestion and fluorescence in situ hybridization (FISH) (Figs. 2c, 3c).

Next, we tested Cucujoidea, which is an extremely diverse and taxonomically difficult superfamily^32^. Despite this diversity, all tested families of Cucujoidea (Cucujidae, Monotomidae, Silvanidae, and Nitidulidae) had the (TTAGG)_n_ sequence. The presence of (TTAGG)_n_ motif was also detected in all tested representatives of the Coccinelloidea (Bothrideridae and Coccinellidae). None of the tested representatives displayed the presence of (TCAGG)_n_.

In both Curculionoidea and Chrysomeloidea, which are considered sister groups^32^, the occurrence of the (TTAGG)_n_ sequence highly varied. In Curculionoidea, while the sequence was detected in Anthribidae, no signal was observed in Brentidae and Attelabidae, and variability in sequence occurrence was observed at family and subfamily levels within Curculionidae. Consistent with previous findings, Curculionidae displayed the presence of the (TTAGG)_n_ sequence in *Ips typograpus* (Scolytinae; consistent with^33^) and *Phylobius urticae* (Entiminae, Phylobiini; consistent with^17^). However, based on hybridization data and the NCBI database Scolytinae, Entiminae and Curculioninae showed variability in the (TTAGG)_n_ occurrence, and the sequence was not detected in Dryophthorinae and Cossoninae. In Chrysomeloidea, instability in the (TTAGG)_n_ presence was observed at family and subfamily levels within Chrysomelidae and Cerambycidae. Based on our data, the sequence was absent in four of the 22 tested species of Chrysomelidae within Criocerinae (in one of two tested species), Galerucinae (in two of four tested species), in one tested species of Clytrinae, and three of six Cerambycidae representatives within Lepturinae (in one of three tested species) and two tested species of Lamiinae. Interestingly, the (TCAGG)_n_ motif was detected in two representatives of Phytophaga: *Byctiscus populi* (Curculionoidea, Attelabidae) and *Leiopus nebulosus* (Chrysomeloidea, Cerambycidae).

In the superfamily Bostrichoidea, which is a sister group of Cucujiformia^31^, all tested families (Bostrichidae, Anobiidae, Dermestidae, Nosodendridae, and Ptinidae) showed the presence of the (TTAGG)_n_ sequence and the absence of the (TCAGG)_n_ sequence.

A more detailed characterization of the hybridization signals was conducted using Southern hybridization in selected representatives of the tested taxa. The signals were formed by long smears, mostly ranging from 2 kb to more than 21 kb, and mostly revealed numbers of hybridization bands of different molecular weights (Fig. 2a,b).

Collectively, our study indicates that the (TTAGG)_n_ sequence has been repeatedly or completely lost in Geadephaga and more than half of the tested polyphagan superfamilies (Fig. 3a,b), and the sequence was replaced with (TCAGG)_n_ motif in sister groups Tenebrionoidea and Cleroidea.

### Search for telomeric sequence variants: the (TTGGG)_n_ sequence as a novel telomeric sequence in beetles

A search of the NCBI databases for tandem repeats revealed that the (TTGGG)_n_ and (TGAGG)_n_ sequences are candidates for novel telomeric sequences in Coleoptera (Table S3). Using dot blot hybridization, we examined the TTAGG- and TCAGG-negative species for the presence of these sequences. Besides, we examined these species for the presence of several other sequence variants that have been reported as telomeric motifs in different organisms, including (TTTGGG)_n_^34^, (TTGGGG)_n_^35^ (from ciliate protozoans), (TTAGGG)_n_ (from vertebrates)^36^, and TTTAGGG (from plants)^37^. Except for (TTGGG)_n_, no positive signals were detected for any of the other tested sequences. Consistent with the NCBI database showing the presence of the (TTGGG)_n_ in three scarabaeoid species (*Onthophagus taurus*, *Pachysoma striatus*, and *Canthidium* sp.), the sequence was detected in *Anoplotrupes stercorosus*, and the presence of the sequence on chromosome termini was confirmed using Bal31 digestion and FISH (Figs. 2c, 3d,e). Surprisingly, (TTGGG)_n_ has not been confirmed in other tested scarabaeoid species (not shown).

### Repeated losses of telomeric motifs in certain taxa reflect the species richness but not the age of the taxa

The presence/absence of the (TTAGG)_n_ sequence varied within the Curculionoidea, Chrysomeloidea, and Staphylinoidea, which are the three most diverse metazoan superfamilies. In these taxa, variance in the presence of (TTAGG)_n_ was observed even at the family or subfamily level, that is, within Staphylinidae (> 63,000 species^38^), Chrysomelidae (> 32,000 species^21^), Cerambycidae (> 32,000 species), and Curculionidae (> 51,000 species^21^) (Fig. 3). Similarly, the occurrence of (TTAGG)_n_ varied in the highly diverse Carabidae (> 34,000 species^21^). In contrast, no variance was observed in, for instance, the less diverse, but highly sampled, Tenebrionoidea, Cleroidea, or Elateroidea.

To test the hypothesis that more speciose taxa have greater telomere diversity, we used the GLS method. We found that there was a significant effect of motif diversity on the species richness per taxon (GLS, F_1,12_ = 11.2, P = 0.006, Fig. 1): the diversity increased exponentially with richness (Fig. 4). We also tested the effect of age of the taxon; the effect of age was not significant (GLS, F_1,11_ = 1.3, P = 0.28).

## Discussion

There are two main hypotheses to explain the number of species in a clade^39–42^. The clade-age hypothesis proposes that species richness increases with the age of the clade. According to the diversification-rate hypothesis, the number of species depends on net diversification, which reflects speciation minus extinction over time; thus, old clades with low richness have low net diversification rates, while young clades with a high number of species have high rates of diversification. In our study, we observed a positive relationship between species number and diversity in the telomere sequence in clades, regardless of the age of the clades. Therefore based on our data, we hypothesize that the key reason for the frequently observed telomeric losses in certain coleopteran clades is the high species diversity within the clades; further, it should be considered that the frequency of sequence loss might be in some cases accentuated by insufficiently clear phylogenetic relationships in clades, such as those within Chrysomeloidea^32^, Adephaga^43,44^ or between Scarabaeiformia and Staphyliniformia^26,45–50^.

The positive correlation between species richness and diversity in telomeric sequences seems to be consistent with the highly frequent (TTAGG)_n_ losses within the Insecta, with 1,020,007 species, representing about 66% of all animals, and the variance in telomeric sequences is observed in one of the most diverse insect orders, which are, along with Coleoptera, Hemiptera (104,000 species), Hymenoptera (117,000 species), and Diptera (156,000 species)^16,51^. In contrast, 57 tested representatives of Orthoptera (27,000 species^39^) had the (TTAGG)_n_ sequence^16^. Certain similarities can be observed in other highly diverse classes of organisms, such as Magnoliophyta (flowering plants, 300,000 species^52^). Loss of the plant ancestral telomere DNA sequence, (TTTAGGG)_n_, has been reported in numerous flowering plants, in which the sequences were replaced by alternative motifs^10,53–57^, and high diversity in telomere repeats was observed in two species-rich orders, Asparagales, the largest order within the monocotyledons, consisting of around 30,000 species^58–61^, and Lamiales, consisting of 23,000 species^55^. Besides, numerous plant orders possess unknown telomere sequences^10^. It is also debatable whether the positive correlation between species richness and telomere sequence diversity is due to the direct involvement of telomere biology in the process of speciation, or if it is simply because the diversity in species-rich clades can be detected more easily.

Telomeric DNA sequences interfere in the formation of a telomere capping structure^62^, and therefore, we can assume that a small deviation in the telomere sequence can affect whole-genome stability and have an enormous evolutionary impact. It is well known that telomere dysfunction results in chromosomal fusions, large-scale genomic rearrangements, and instability across the genome, which are all hallmarks of speciation. Chromosomal rearrangements are associated with high mortality and, due to meiotic anomalies, reduced fertility, which collectively results in a generation of isolated groups within the population; each of the groups could develop into a new species^63^. Therefore, we can suppose that telomeres might provide a powerful mechanism for rapid genomic changes that can lead to reproductive isolation and speciation, and our data supports this premise.

We propose that chromosomal rearrangements triggered by telomere dysfunction might result in species extinction or a formation of new species, which includes either stabilization of the existing telomeric system or development of a new one. Our study showed that numerous Coleoptera species lack the ancestral insect telomere sequence, but the exact telomeric motif in most of these species remains unknown. We can only speculate whether the sequence was replaced by another short telomeric sequence or a completely different system independent of telomerase. To adopt new features, telomerase system can be very plastic as documented by the remarkable divergence in telomere sequences in budding yeast, which shows extraordinary lengths, occasional degeneration, and a frequent absence of G/C-richness^64^, or it can be documented by numerous species with backup pathways for telomere lengthening when telomerase activity is compromised^65–68^.

Various studies identified transposable elements as modifiers of adaptive response upon exposure to a stressful environment. It has been shown that transposable elements can be activated by diverse stressors such as DNA damaging agents, thermal stress, and also telomere dysfunction^69–72^. Transposable elements are known to induce genomic rearrangements^73^, and we assume that the activation of transposable elements by telomere dysfunction not only contributes to speciation, but at the same time, it allows the development of the retroelement-based telomeric system during the speciation process. Although telomeric retroelements are a hallmark of *Drosophila* telomeres, they are found also in distantly related species incorporated in the telomerase-added sequences at their chromosome termini. It needs to be as well pointed out that telomeric retroelements are not universal systems of telomere elongation in the genus *Drosophila* and not at all in Diptera, as telomere maintenance in some species uses telomere-telomere recombination^74–79^. Nevertheless, we can suppose that the retroelement and recombination systems work as the backup pathways for telomere lengthening when telomerase activity is compromised^65–68^.

Together, these findings reveal the plasticity of chromosome ends for incorporating new features to maintain telomere integrity and functionality, perhaps pointing to the mechanisms by which telomeres contribute to the speciation and adaptation process. We believe that telomere diversity in insects provides the right opportunity to research such underexplored aspects of telomere biology.

## Material and methods

### Taxon sampling

In this study, we sampled 175 coleopteran species representing 14 superfamilies, 46 families, and 84 subfamilies (Table S1).

### Hybridization probes

Hybridization probes were prepared using non-template PCR. The list of primers is provided in Table S2. The non-template PCR reaction contained 10 μM forward primer, 10 μM reverse primer, 10 mM dNTP mix, and Taq polymerase (5 U/μl). The PCR products were labeled by random primed labeling with digoxigenin-11-dUTP using a DIG DNA Labeling Kit (Roche diagnostics) and biotin-14-dATP using a Biotin-Nick Translation Mix (Roche diagnostics).

### Southern hybridization

Genomic DNAs were isolated by a standard procedure of phenol–chloroform-isoamyl alcohol extraction. From each species, about 1 μg DNA was digested with a mixture of RsaI/HinfI restriction endonucleases (New England BioLabs) (1:1), separated on 1% agarose gel, and blotted onto a Hybond-N + nylon membrane (Amersham Biosciences) by capillary transfer in 20× SSC overnight, followed by Southern or dot-blot hybridization with chemiluminescence detection as described previously^80^. Briefly, the hybridization was performed for 3 h (at 50 °C) in 5 ml hybridization solution (1 M NaCl, 10 mM Tris–HCl pH 7.6, 1 mM EDTA, 40% formamide, 0.5% SDS, 5× Denhardt’s solution) containing DIG-labeled probe (10 ng/ml), followed by washing in 2× SSC, 0.1% SDS (for 10 min, at room temperature) and 0.2× SSC, 0.1% SDS (for 20 min, at 68 °C). The chemiluminescence detection was performed using the alkaline phosphatase-CDP-Star system (Sigma-Aldrich), and the signals were detected using the CCD camera LAS-3000.

### Bal 31 nuclease assay

To confirm the terminal positions of tested sequences on chromosome ends, the genomic DNA was subjected to BAL 31 exonuclease. Bal 31 nuclease degrades 3′ and 5′ termini of duplex DNA without generating internal scissions in the intact double helix. The assay was performed as described previously^81^. Briefly, the genomic DNA (15 μg) was incubated with 0.03U BAL 31 nuclease (New England BioLabs) in a total volume of 180 μl at 30 °C, and 60-μl aliquots were taken before the BAL31 addition and after 30, 60, 90, and 120 min of the treatment. The reaction was immediately stopped by incubation at 65 °C for 10 min in the presence of 20 mM EGTA. Samples were purified using phenol–chloroform-isoamyl alcohol extraction, and DNA was extracted using the standard ethanol precipitation by adding 1/10th volume of sodium acetate and 2 volumes of ethanol. Then, the DNA samples (1 µg) were digested with the restriction enzymes RsaI and Hinf I (New Englands Biolabs) and subjected to Southern hybridization.

### Chromosomes preparations and FISH

Chromosome preparations were prepared from gonads of tested adults. The gonads were dissected in the Ringer’s solution, incubated in a hypotonic solution (0.075 M KCl) for 10–20 min, and then fixed in freshly prepared Carnoy solution (ethanol:chloroform:acetic acid, 6:3:1). Using tungsten needles, tissue was ripped up in a drop of 60% acetic acid and spread on the microscope slide placed on a heating plate (45 °C). Preparations were dehydrated in an ethanol series (70%, 80%, 90%, 30 s each) and stored at − 20  °C before their use.

Fluorescence in situ hybridization (FISH) with biotinylated probes was performed overnight at 37 °C in a solution of 60% formamide, 2× SSC, 10% dextran sulfate, 20 mM sodium phosphate, and 10 ng/μl of the DNA probe. Post-hybridization washes were done in 50% formamide/2× SSC at 37 °C. Biotin-labeled probes were detected with streptavidin-Cy3 (Jackson ImmunoResearch Laboratories) and biotinylated anti-streptavidin (Jackson ImmunoResearch Laboratories). The chromosomes were counterstained with DAPI (0.1 μg/μl) mounted in 20 μl of an anti-fade medium (25 mg/ml of 1,4-Diazobicyclo-(2,2,2) octane in 90% glycerol/10% 1xPBS) and analyzed with a Zeiss Axioplan 2 microscope. Images were taken separately for each fluorescent dye with a CCD camera (F-view, Soft Imaging System GmbH) in black and white resolution and subsequently stained and merged in Adobe Photoshop CS4.

### Search NCBI databases

Short read archive at National Center for Biotechnology Information (SRA, NCBI) was searched for datasets from Coleoptera with restrictions to the WGS strategy, genomic source, and random selection. If available, only the first ten thousand spots were downloaded at maximum and analyzed using Tandem Repeats Finder with the options set as described previously for Bal31-NGS^82^. The presence/absence of telomeric motifs was checked manually in the Tandem Repeats Finder output.

### Statistical analysis

The phylogenetic generalized least squares (GLS) from the ape package^83^ was used to test the hypothesis that more speciose taxa have greater telomere diversity. GLS was used because the measurements were not independent (as they may have a common evolutionary history)^84^ and the relationship was not linear (to be tested by correlation). We constructed a truncated phylogenetic tree using the most recent phylogenetic hypothesis of Coleoptera. As branch distances were not reported, these were computed using Grafen’s method^85^. Shannon (entropy) index was used to estimate the motif diversity and this response variable was then logarithmically transformed to fit an exponential relationship. Species richness was estimated at the superfamily level. The linear predictor included the number of investigated species (per taxon) to correct for different intensities of measurements and the age of the taxa. Brownian motion model of trait evolution was used in the analysis. The analysis was performed in the R environment (http://www.r-project.org/index.html) using R version 4.1.0^23^.

### Image processing

All images were processed with Adobe Photoshop CS6 (Adobe Systems; V.6.0.1), using proportionate adjustments of brightness or contrast.

## Supplementary Information

Supplementary Table S1.

Supplementary Table S2.

Supplementary Table S3.
