# Supplementary material for: Telomeric DNA sequences in beetle taxa vary with species richness
Source: Sci Rep. 2021 Jun 25;11:13319. doi: 10.1038/s41598-021-92705-y (PMC8233369; doi:10.1038/s41598-021-92705-y)
Supplement: Supplementary file 1 — Supplementary Table S1. [file 41598_2021_92705_MOESM1_ESM.docx]

| **Supplementary Table 1. Taxonomy of tested species and source of the samples.** | | | |  |  |  |  |  |
| --- | --- | --- | --- | --- | --- | --- | --- | --- |
| **Superfamily** | **Family / subfamily** | **Species** | **Sample source** | **Sample collected / identified by:** |  |  |  |  |
| Caraboidea |  |  |  |  |  |  |  |  |
|  | **Carabidae:** |  |  |  |  |  |  |  |
|  | Harpalinae | *Selenophorus alternans* | NCBI database, SRR5651490 | n/a |  |  |  |  |
|  | Licinae | *Chlaenius nitidulus* | Czechia | Prušáková, Frydrychová / Bezděk |  |  |  |  |
|  | Cicindelinae | *Tetracha sp.* | NCBI database, SRR5651489 | n/a |  |  |  |  |
|  | Carabinae | *Carabus granulatus* | Czechia | Prušáková, Frydrychová / Bezděk |  |  |  |  |
|  | Platyninae | *Calathus melanocephalus* | Czechia | Prušáková, Frydrychová / Bezděk |  |  |  |  |
|  |  | *Miquihuana rhandiniformis* | NCBI database, SRR3421278 | n/a |  |  |  |  |
|  | Trechinae | *Bembidarenas sp.* | NCBI database, SRR2939023 | n/a |  |  |  |  |
|  |  | *Bembidion chilioperyphus* | NCBI database, SRR2939027 | n/a |  |  |  |  |
|  |  | *B. orion* | NCBI database, SRR2939024 | n/a |  |  |  |  |
|  |  | *B. castor* | NCBI database, SRR5230398 | n/a |  |  |  |  |
|  |  | *B. aeruginosum* | NCBI database, SRR5230397 | n/a |  |  |  |  |
|  |  | *B. musae* | NCBI database, SRR2939016 | n/a |  |  |  |  |
|  |  | *B. clemens* | NCBI database, SRR5230406 | n/a |  |  |  |  |
|  |  | *B. breve* | NCBI database, SRR5514454 | n/a |  |  |  |  |
|  |  | *B. laxatum* | NCBI database, SRR5514452 | n/a |  |  |  |  |
|  |  | *B. obscuripenne* | NCBI database, SRR5230404 | n/a |  |  |  |  |
|  |  | *B. lapponicum* | NCBI database, SRR2939026 | n/a |  |  |  |  |
|  |  | *B. flohri* | NCBI database, SRR5230420 | n/a |  |  |  |  |
|  |  | *B. scenicum* | NCBI database, SRR5230409 | n/a |  |  |  |  |
|  |  | *B. lachnophoroides* | NCBI database, SRR2939022 | n/a |  |  |  |  |
|  |  | *B. ulkei* | NCBI database, SRR5230416 | n/a |  |  |  |  |
|  |  | *B. subfusum* | NCBI database, SRR2939014 | n/a |  |  |  |  |
|  |  | *Lionepha casta* | NCBI database, SRR5230408 | n/a |  |  |  |  |
|  |  | *L. chintimini* | NCBI database, SRR2939021 | n/a |  |  |  |  |
|  |  | *L. erasa* | NCBI database, SRR5230423 | n/a |  |  |  |  |
|  |  | *L. lindrothellus* | NCBI database, SRR5230400 | n/a |  |  |  |  |
|  |  | *Pogonus chalceus* | NCBI database, SRR5427959 | n/a |  |  |  |  |
|  | Pterostichinae | *Pterostichus melanarius* | Czechia | Čížek |  |  |  |  |
|  |  | *Poecilus cupreus* | Czechia | Prušáková, Frydrychová / Bezděk |  |  |  |  |
|  |  | *Pseudamara arenaria* | NCBI database, SRR3421213 | n/a |  |  |  |  |
| Dytiscoidea | **Dytiscidae:** |  |  |  |  |  |  |  |
|  | Colymbetinae | *Rhantus sp.* | NCBI database, SRR5437743 | n/a |  |  |  |  |
|  | Hydroporinae | *Stictotarsus aequinoctialis* | NCBI database, SRR1145745 | n/a |  |  |  |  |
| Scirtoidea | **Scirtidae:** Scirtinae | *Scirtes sp.* | Czechia | Čížek |  |  |  |  |
| Buprestoidea |  |  |  |  |  |  |  |  |
|  | **Buprestidae:** Agrilinae | *Agrilus cuprescens* | Czechia | Prušáková, Frydrychová / Bezděk |  |  |  |  |
|  |  | *Agrilus planipennis* | NCBI database, SRR1174015 | n/a |  |  |  |  |
| Byrrhoidea | **Dryopidae** | *Dryops sp.* | Czechia | Čížek |  |  |  |  |
| Elateroidea |  |  |  |  |  |  |  |  |
|  | **Throscidae:** Throscinae | *Trixagus dermestoides* | Czechia | Čížek |  |  |  |  |
|  | **Eucnemidae**: Melasinae | *Melasis buprestoides* | Czechia | Čížek |  |  |  |  |
|  | **Cantharidae:** Cantharinae | *Cantharis fusca* | Czechia | Prušáková, Frydrychová / Bezděk |  |  |  |  |
|  | **Elateridae:** |  |  |  |  |  |  |  |
|  | Elaterinae | *Agriotes lineatus* | Czechia | Bezděk |  |  |  |  |
|  | Agrypninae | *Agrypnus murinus* | Czechia | Prušáková, Frydrychová / Bezděk |  |  |  |  |
|  | **Lampyridae:** |  |  |  |  |  |  |  |
|  | Lampyrinae | *Lamprohiza splendidula* | Czechia | Čížek |  |  |  |  |
|  |  | *Ellychnia corrusca* | NCBI database, SRR5434917 | n/a |  |  |  |  |
|  |  | *Lucidota atra* | NCBI database, SRR5434916 | n/a |  |  |  |  |
|  |  | *L. punctata* | NCBI database, SRR5434915 | n/a |  |  |  |  |
|  |  | *Phausis reticulata* | NCBI database, SRR5434914 | n/a |  |  |  |  |
|  |  | *Photinus australis* | NCBI database, SRR5434910 | n/a |  |  |  |  |
|  |  | *P. brimleyi* | NCBI database, SRR5434909 | n/a |  |  |  |  |
|  |  | *P. carolinus* | NCBI database, SRR5434908 | n/a |  |  |  |  |
|  |  | *P. cooki* | NCBI database, SRR5434907 | n/a |  |  |  |  |
|  |  | *P. indictus* | NCBI database, SRR5434906 | n/a |  |  |  |  |
|  |  | *P. macdermotti* | NCBI database, SRR5434905 | n/a |  |  |  |  |
|  |  | *P. obscurellus* | NCBI database, SRR5434904 | n/a |  |  |  |  |
|  |  | *Pyractomena angulata* | NCBI database, SRR5434898 | n/a |  |  |  |  |
|  |  | *P. borealis* | NCBI database, SRR2127933 | n/a |  |  |  |  |
|  |  | *P. marginalis* | NCBI database, SRR5434897 | n/a |  |  |  |  |
|  |  | *Pyropyga decipiens* | NCBI database, SRR5434911 | n/a |  |  |  |  |
|  | Luciolinae | *Aquatica lateralis* | NCBI database, DRR119297 | n/a |  |  |  |  |
|  | Photurinae | *Photuris frontalis* | Czechia | Prušáková, Frydrychová / Bezděk |  |  |  |  |
| Staphylinoidea |  |  |  |  |  |  |  |  |
|  | **Silphidae** | *Nicrophorus orbicollis* | NCBI database, SRR5308050 | n/a |  |  |  |  |
|  | **Leiodidae:** Leiodinae | *Agathidium sp.* | Czechia | Čížek |  |  |  |  |
|  | **Staphylinidae:** |  |  |  |  |  |  |  |
|  | Pselaphinae | *Adranes taylori* | NCBI database, SRR5906382 | n/a |  |  |  |  |
|  | Oxytelinae | *Carpelimus sp.* | NCBI database, ERR2799448 | n/a |  |  |  |  |
|  | Aleocharinae | *Aenictocupidus jacobsonorum* | NCBI database, SRR7814865 | n/a |  |  |  |  |
|  |  | *Mimaenictus wilsoni* | NCBI database, SRR7779820 | n/a |  |  |  |  |
|  |  | *Weissflogia rhopalogaster* | NCBI database, SRR7779821 | n/a |  |  |  |  |
|  |  | *Dalotia coriaria* | NCBI database, SRR4301137 | n/a |  |  |  |  |
|  |  | *Earota dentata* | NCBI database, SRR7826983 | n/a |  |  |  |  |
|  |  | *Diploeciton nevermanni* | NCBI database, SRR4342174 | n/a |  |  |  |  |
|  |  | *Deinopsis erosa* | NCBI database, SRR5176562 | n/a |  |  |  |  |
|  |  | *Dorylogaster longipes* | NCBI database, SRR7806411 | n/a |  |  |  |  |
|  |  | *Ecitomorpha arachnoides* | NCBI database, SRR7778898 | n/a |  |  |  |  |
|  |  | *Ecitophya simulans* | NCBI database, SRR4301374 | n/a |  |  |  |  |
|  |  | *Drusilla canaliculata* | NCBI database, SRR5906249 | n/a |  |  |  |  |
|  |  | *Ecitoglossa quadriceps* | NCBI database, SRR5259914 | n/a |  |  |  |  |
|  |  | *Platyusa sonomae* | NCBI database, SRR5909600 | n/a |  |  |  |  |
|  |  | *Tetradonia laticeps* | NCBI database, SRR4342885 | n/a |  |  |  |  |
|  |  | *Labidoglobus nevermanni* | NCBI database, SRR6000456 | n/a |  |  |  |  |
|  |  | *L. appendiculatus* | NCBI database, SRR7826023 | n/a |  |  |  |  |
|  |  | *Pseudomimeciton antennatum* | NCBI database, SRR5170144 | n/a |  |  |  |  |
|  |  | *Oxypoda opaca* | NCBI database, SRR5176940 | n/a |  |  |  |  |
|  |  | *Sceptobius lativentris* | NCBI database, SRR5909496 | n/a |  |  |  |  |
|  | Tachyporinae | *Coproporus ventriculus* | NCBI database, SRR4301367 | n/a |  |  |  |  |
| Scarabaeoidea |  |  |  |  |  |  |  |  |
|  | **Geotrupidae** | Anoplotrupes stercorosus | Czechia | Prušáková, Frydrychová / Bezděk |  |  |  |  |
|  | **Scarabaeidae:** |  |  |  |  |  |  |  |
|  | Rutelinae | *Phyllopertha horticola* | Czechia | Prušáková, Frydrychová / Bezděk |  |  |  |  |
|  |  | *Chrysina resplendens* | NCBI database, SRR7262138 | n/a |  |  |  |  |
|  |  | *Popillia japonica* | NCBI database, SRR7541039 | n/a |  |  |  |  |
|  | Valginae | *Valgus hemipterus* |  | Čížek |  |  |  |  |
|  | Dynastinae | *Oryctes borbonicus* | NCBI database, SRR2968118 | n/a |  |  |  |  |
|  | Scarabaeinae | *Canthidium sp.* | NCBI database, SRR5651516 | n/a |  |  |  |  |
|  |  | *Onthophagus taurus* | NCBI database, SRR1201274 | n/a |  |  |  |  |
|  |  | *Pachysoma endroeydi* | NCBI database, SRR5359850 | n/a |  |  |  |  |
|  |  | *P. striatus* | NCBI database,SRR5360616 | n/a |  |  |  |  |
|  | Sericinae | *Serica brunnea* | Czechia | Čížek |  |  |  |  |
|  | Cetoniinae | *Oxythyrea funesta* | Czechia | Čížek |  |  |  |  |
|  | **Lucanidae:** Syndesinae | *Sinodendron cylindricum* | Czechia | Čížek |  |  |  |  |
| Bostrichoidea |  |  |  |  |  |  |  |  |
|  | **Bostrichidae:** Bostrichinae | *Bostrichus capucinus* | Czechia | Čížek |  |  |  |  |
|  | **Dermestidae:** Megatominae | *Globicornis emarginata* | Czechia | Čížek |  |  |  |  |
|  |  | *Anthrenus scrophulariae* | Czechia | Čížek |  |  |  |  |
|  | **Ptinidae:** |  |  |  |  |  |  |  |
|  | Gibiinae | *Gibbium aequinoctinale* | Laboratory culture* | n/a |  |  |  |  |
|  | Ptilininae | *Ptilinus pectinicornis* | Czechia | Čížek |  |  |  |  |
|  | **Nosodendridae** | *Nosodendron fasciculare* | Czechia | Čížek |  |  |  |  |
| Tenebrionoidea |  |  |  |  |  |  |  |  |
|  | **Mordellidae:** Mordellinae | *Varimorda villosa* | Czechia | Bezděk |  |  |  |  |
|  | **Oedemeridae:** Oedemerinae | *Oedemera femorata* | Czechia | Bezděk |  |  |  |  |
|  | **Salpingidae:** Salpinginae | *Vincenzellus ruficollis* | Czechia | Čížek |  |  |  |  |
|  | **Pyrochroidae:** Pyrochroinae | *Pyrochroa coccinea* | Czechia | Čížek |  |  |  |  |
|  | **Mycetophagidae:** Mycetophaginae | *Mycetophagus quadriguttatus* | Czechia | Bezděk |  |  |  |  |
|  | **Ciidae:** Ciinae | *Cis sp.* | Czechia | Čížek |  |  |  |  |
|  | **Tenebrionidae:** |  |  |  |  |  |  |  |
|  | Tenebrioninae | *Tenebrio mollitor* | Laboratory culture* | n/a |  |  |  |  |
|  |  | *Phylacinus fisheri* | NCBI database, SRR6898367 | n/a |  |  |  |  |
|  | **Meloidae:** Meloinae | *Hycleus cichorii* | NCBI database, SRR5710158 | n/a |  |  |  |  |
|  | **Anthicidae** | *Anthicidae sp.* | NCBI database,SRR5651487 | n/a |  |  |  |  |
| Cleroidea |  |  |  |  |  |  |  |  |
|  | **Melyridae:** |  |  |  |  |  |  |  |
|  | Malachiinae | *Malachius bipustulatus* | Czechia | Prušáková, Frydrychová / Bezděk |  |  |  |  |
|  | Dasytinae | *Dasytes sp.* | Czechia | Prušáková, Frydrychová / Bezděk |  |  |  |  |
|  | **Cleridae:** |  |  |  |  |  |  |  |
|  | Clerinae | *Clerus mutillarius* | Czechia | Čížek |  |  |  |  |
|  |  | *Thanasimus formicarius* | Czechia | Prušáková, Frydrychová / Bezděk |  |  |  |  |
|  | Tillinae | *Tillus elongatus* | Czechia | Čížek |  |  |  |  |
|  | **Trogossitidae:** Peltinae | *Thymalus limbatus* | Czechia | Čížek |  |  |  |  |
|  | **Biphyllidae** | *Diplocoelus fagi* | Czechia | Čížek |  |  |  |  |
|  | **Byturidae:** Byturinae | *Byturus ochraceus* | Czechia | Čížek |  |  |  |  |
|  |  | *B. tomentosus* | Czechia | Čížek |  |  |  |  |
| Coccinellidea |  |  |  |  |  |  |  |  |
|  | **Bothrideridae:** Bothriderinae | *Bothrideres bipunctatus* | Czechia | Čížek |  |  |  |  |
|  | **Coccinellidae:** Coccinelinae | *Coccinella septempunctata* | Czechia | Prušáková, Frydrychová / Bezděk |  |  |  |  |
|  |  | *Adalia decemlineata* | Czechia | Prušáková, Frydrychová / Bezděk |  |  |  |  |
|  |  | *Harmonia axyridis* | Czechia | Prušáková, Frydrychová / Bezděk |  |  |  |  |
|  |  | *Propylea quatuordecimpunctata* | Czechia | Prušáková, Frydrychová / Bezděk |  |  |  |  |
|  |  | *Eriopis connexa* | NCBI database, SRR5651504 | n/a |  |  |  |  |
|  | Scymninae | *Nephaspis sp.* | NCBI database, SRR5651493 | n/a |  |  |  |  |
| Cucujoidea |  |  |  |  |  |  |  |  |
|  | **Monotomidae:** |  |  |  |  |  |  |  |
|  | Rhizophaginae | *Rhizophagus bipustulatus* | Czechia | Čížek |  |  |  |  |
|  |  | *R. simplex* | Czechia | Čížek |  |  |  |  |
|  | **Nitidulidae:** |  |  |  |  |  |  |  |
|  | Meligethinae | *Pria dulcamare* | Czechia | Čížek |  |  |  |  |
|  | Nitidulinae | *Pocadius sp.* | Czechia | Prušáková, Frydrychová / Bezděk |  |  |  |  |
|  | **Cucujidae:** Laemophloinae | *Cryptolestes capensis* | Laboratory culture* | n/a |  |  |  |  |
| Chrysomeloidea |  |  |  |  |  |  |  |  |
|  | **Chrysomelidae:** |  |  |  |  |  |  |  |
|  | Chrysomelinae | *Chrysolina graminis* | Czechia | Prušáková, Frydrychová / Bezděk |  |  |  |  |
|  |  | *Chrysomela vigintipunctata* | Czechia | Prušáková, Frydrychová / Bezděk |  |  |  |  |
|  |  | *Phaedon sp.* | Czechia | Prušáková, Frydrychová / Bezděk |  |  |  |  |
|  |  | *Leptinotarsa decemlineata* | Czechia | Prušáková, Frydrychová / Bezděk |  |  |  |  |
|  | Bruchinae | *Callosobruchus chinensis* | Laboratory culture* | n/a |  |  |  |  |
|  | Cryptocephalinae | *Cryptocephalus trimaculatus* | Czechia | Prušáková, Frydrychová / Bezděk |  |  |  |  |
|  |  | *C. stericeus* | Czechia | Prušáková, Frydrychová / Bezděk |  |  |  |  |
|  | Eumolpinae | *Colaspis sp.* | NCBI database, SRR5651512 | n/a |  |  |  |  |
|  | Galerucinae | *Diabrotica barberi* | NCBI database, SRR987700 | n/a |  |  |  |  |
|  |  | *D. virgifera* | NCBI database, SRR1106904 | n/a |  |  |  |  |
|  |  | *Aphthona sp.* | Czechia | Prušáková, Frydrychová / Bezděk |  |  |  |  |
|  |  | *Altica sp.* | Czechia | Prušáková, Frydrychová / Bezděk |  |  |  |  |
|  | Donaciinae | *Donacia semicuprea* | Czechia |  |  |  |  |  |
|  | Criocerinae | *Lilioceris lilii* | Czechia | Prušáková, Frydrychová / Bezděk |  |  |  |  |
|  |  | *Oulema sp.* | Czechia | Prušáková, Frydrychová / Bezděk |  |  |  |  |
|  | Clytrinae | *Clytra laeviscula* | Czechia | Prušáková, Frydrychová / Bezděk |  |  |  |  |
|  | **Cerambycidae:** |  |  |  |  |  |  |  |
|  | Lepturinae | *Pachyta quadrimaculata* | Czechia | Prušáková, Frydrychová / Bezděk |  |  |  |  |
|  |  | *Rhagium mordax* | Czechia | Čížek |  |  |  |  |
|  | Lamiinae | *Leiopus nebulosus* | Czechia | Čížek |  |  |  |  |
|  |  | *Anoplophora glabripennis* | NCBI database, SRR941723 | n/a |  |  |  |  |
|  | Cerambycinae | *Phymatodes lengi* | NCBI database, SRR6984058 | n/a |  |  |  |  |
| Curculionoidea |  |  |  |  |  |  |  |  |
|  | **Anthribidae:** Anthribinae | *Platyrhinus resinosus* | Czechia | Čížek |  |  |  |  |
|  | **Attelabidae:** Rhynchitinae | *Byctiscus populi* | Czechia | Čížek |  |  |  |  |
|  | **Brentidae:** Apioninae | *Protapion apricans* | Czechia | Bezděk |  |  |  |  |
|  | **Curculionidae:** |  |  |  |  |  |  |  |
|  | Entiminae | *Otiorhynchus sp.* | Czechia | Čížek |  |  |  |  |
|  |  | *Phyllobius urticae* | Czechia | Prušáková, Frydrychová / Bezděk |  |  |  |  |
|  | Dryophthorinae | *Sitophilus granarius* | Laboratory culture* |  |  |  |  |  |
|  |  | *S. oryzae* | Laboratory culture* |  |  |  |  |  |
|  | Cossoninae | *Cossonus sp.* | Czechia | Čížek |  |  |  |  |
|  | Curculioninae | *Curculio nucum* | Czechia | Prušáková, Frydrychová / Bezděk |  |  |  |  |
|  |  | *Anthonomus grandis* | NCBI database, SRR5651518 | n/a |  |  |  |  |
|  | Scolytinae | *Dendroctonus jeffreyi* | NCBI database, SRR6279177 | n/a |  |  |  |  |
|  |  | *D. ponderosae* | NCBI database, SRR6279176 | n/a |  |  |  |  |
|  |  | *Hypothenemus hampei* | NCBI database, SRR1986395 | n/a |  |  |  |  |
|  |  | *Ips typographus* | Czechia | Prušáková, Frydrychová / Bezděk |  |  |  |  |
| * Crop Research Institute, Prague | | | | |  |  |  |  |
|  | | | | |  |  |  |  |
|  |  |  |  |  |  |  |  |  |
|  |  |  |  |  |  |  |  |  |
|  |  |  |  |  |  |  |  |  |
|  |  |  |  |  |  |  |  |  |
