# Supplementary material for: Telomeric DNA sequences in beetle taxa vary with species richness
Source: Sci Rep. 2021 Jun 25;11:13319. doi: 10.1038/s41598-021-92705-y (PMC8233369; doi:10.1038/s41598-021-92705-y)
Supplement: Supplementary file 2 — Supplementary Table S2. [file 41598_2021_92705_MOESM2_ESM.docx]

**Table S2. The list of primers**

| Telomere sequence | Forward primer | Reverse primer |
| --- | --- | --- |
| TTAGG | TAGGTTAGGTTAGGTTAGGT | CTAACCTAACCTAACCTAAC |
| TCAGG | CAGGTCAGGTCAGGTCAGGTCAGGT | CTGACCTGACCTGACCTGACCTGAC |
| TTGGG | GTTGGGTTGGGTTGGGTTGGGTTGG | ACCCAACCCAACCCAACCCAACCCA |
| TGAGG | GTGAGGTGAGGTGAGGTGAGGTGAG | ACCTCACCTCACCTCACCTCACCTC |
| TTACC | CTTACCTTACCTTACCTTACCTTAC | AGGTAAGGTAAGGTAAGGTAAGGTA |
| TTTGGG | TGGGGTTTTGGGGTTTTGGGGTTT | CCAAAACCCCAAAACCCCAAAACC |
| TTGGGG | GGTTGGGGTTGGGGTTGGGGTTG | CCCCAACCCCAACCCCAACCCCAA |
| TTTAGGG | GGGTTTAGGGTTTAGGGTTTA | CCCTAAACCCTAAACCCTAAA |
| TTAGGG | GGGTTAGGGTTAGGGTTAGGGTTA | CCCTAACCCTAACCCTAACC |
