# Supplementary material for: Telomeric DNA sequences in beetle taxa vary with species richness
Source: Sci Rep. 2021 Jun 25;11:13319. doi: 10.1038/s41598-021-92705-y (PMC8233369; doi:10.1038/s41598-021-92705-y)
Supplement: Supplementary file 3 — Supplementary Table S3. [file 41598_2021_92705_MOESM3_ESM.docx]

# Table S3. The NCBI data. The numbers indicate numbers of repeats. The predicted telomeric sequence is highlighted in yellow.

# Caraboidea, Carabidae

| **Tetracha (#SRR5651489)** | | | |
| --- | --- | --- | --- |
| 15392 | CGCT | 97 | GGCCGATCAATAAGTATCCGTTTTATCACAG |
| 4451 | CTAAAATTGTGAGA | 96 | TATATAAA |
| 3272 | CGCA | 91 | CATATATA |
| 2694 | GTGAG | 91 | CACGCACA |
| 1638 | ATA | 88 | AACTGGTCTGAACAGGTTATC |
| 1178 | CGG | 87 | TTATA |
| 1042 | TAGTGTAGTA | 86 | ACCTTTG |
| 564 | GGCCGATCAATAAGTACCCGTTTTATCACAG | 86 | CGTACGCGTTATATTACGTAACGCGTA |
| 533 | GTGGT | 86 | GGTGCC |
| 498 | CTTATTGATCGGCCCTGTTATAAAACGGGTA | 86 | AGTGAAATGGAGCGCTAGTT |
| 479 | AATA | 85 | CCGTTTTATAACAGGGCCGATCAATAAGTAT |
| 468 | TAAAATTTTGAGAC | 85 | GGCGGT |
| 445 | GTTTGAACAG | 85 | CAATTTAAGTCTCA |
| 439 | AAACA | 84 | CGGTTCGTTATTTTAC |
| 431 | GAATTTAGCCTCA | 79 | TTTTAGTCCCACAA |
| 422 | CTTCGG | 77 | GCGTGTGC |
| 401 | ACT | 76 | CGATCAATAAGTACCCGTTTTGTCACAGGGT |
| 394 | AAAACGGGTACTTATTGATCGGCCCTGTGAC | 75 | AATTTTAGGCCCAC |
| 336 | CTGGTTGGGACATGAGGTC | 75 | CACA |
| 332 | GGCGCCACGCGCCACCA | 75 | CGGCGA |
| 308 | CAAA | 74 | GCGGCA |
| 298 | ACGAACTGGTCTGAACAGGTT | 72 | TAATATTAATAATTAA |
| 292 | CGAGTGAG | 71 | GATAAAACGGGTACTTATTGATCGGCCCTGC |
| 275 | AAG | 70 | TCCAT |
| 260 | ATAT | 70 | CAGTTCAAACCTGTTCAGAC |
| 259 | TCACCACACGAAACCTCACC | 69 | ACACAC |
| 247 | GCGGC | 69 | GTACCCGTTTTATCACAGAGCCGATCAATAA |
| 236 | TAAAATTGTGAGGC | 68 | GACCTTGACCTTT |
| 224 | TGAAACTAAAATTG | 68 | AAAACGGATACTTATTGATCGGCCCTGTGAC |
| 224 | TAAG | 67 | AATT |
| 224 | AGC | 67 | ATTTTATTT |
| 204 | CCGACG | 63 | GGTCTGAACA |
| 181 | TGTGAGACTAAAT | 62 | GAGCAAC |
| 176 | TACA | 62 | ATATAAT |
| 175 | TATGTA | 62 | GTGTCCTACACGTGGCACTGGGGT |
| 171 | CAGA | 61 | ATTTAGCCCCAGA |
| 167 | ACAATTTTAGTCTT | 60 | AGGGCCGATCAAAAAGTACCCGTTTTATCAC |
| 164 | GCTAATTCTGAG | 59 | CCCCGCCCACTAATTAAA |
| 162 | CACACG | 59 | TAACC |
| 152 | CTACA | 59 | AAAATTGTGAGACA |
| 150 | ATATAT | 58 | GGCCGATCAATAAGTACCCGTTTTATTACAG |
| 138 | CTAAAATTGTGGGG | 58 | ACGCGTACGTACGCGTTATATTATGTA |
| 131 | CGCTCGCTTTCT | 57 | ACTATGACTCTCTTTTCGGGGGAGTA |
| 124 | AAACGGGTACTTATTGATCGGCCTTGTGATA | 56 | ATGAGACTAAAATT |
| 122 | CTAAATTTGTGAGA | 56 | AGATGCTGCTCTCGCTGGTTGG |
| 121 | TACTA | 56 | CTCTATCTCTATCCCTATCT |
| 120 | TCA | 56 | CTATCTCTCTATATCTATCC |
| 120 | GCCGAATTCGCGAATTCGG | 55 | TCG |
| 118 | GTTCATAACCTGTTCAGACCA | 54 | AATAAAATTTTTAAAAA |
| 118 | AACTGGTTTG | 53 | GAATTCGCGAATTCGGCC |
| 114 | AACACA | 52 | TTAGTCACACAATT |
| 113 | AGGTGTGGTG | 52 | CTGCGG |
| 107 | TATTGATCGGCCCTGTGATAAACGGGTACT | 50 | GGCCCC |
| 107 | TTG | 50 | CACT |
| 102 | GTG | 49 | TGTATATATA |
| 102 | AGTGTAGTTTCTTCG | 49 | CAAAACGGATACTTATTGATCGGCCCTGTGT |
| 99 | TTTAT | 49 | CCGATCAATAAGTACCCGTTTATAACAGGG |
| 97 | GGCGGCG | 48 | TATTATTAATTATTA |
| 47 | ATAATATAT | 28 | CGTCGGCGT |
| 46 | TTTTGAGACTAAA | 28 | TGTTTT |
| 45 | TTGTTTTT | 27 | CAACGGCGACCACAG |
| 44 | CCAGTTGATAACCAGTTAAAACCTGTTCAGA | 27 | GCCGAATTCGACGAATTCGG |
| 43 | TTAGTCTCACATTT | 27 | AATTT |
| 43 | CGATCAATAAGTACCCGTTTTATCACAAAGC | 27 | ACATTTTAGTCTC |
| 43 | GGTTAGCAACTGGTCTGAACA | 27 | AGGCTAAATTCTA |
| 42 | AGTACTG | 27 | ACACACACGC |
| 42 | TATATAG | 27 | GGCGTCGTCGGCGTC |
| 41 | CTGGTTGAGGACATGAGGTC | 27 | GTTGATAACCTGTTCAAACCTGTTCAAACCA |
| 41 | GCGCCC | 26 | AGTAATATG |
| 41 | CTGTTCAGACCAGTTCTAAC | 26 | TCTATC |
| 41 | CTTTTCGCGGGAGTAACTATGACTCT | 26 | GAACTGGTCT |
| 40 | CGGTG | 26 | TTGACTGTAATTTTAACAGTC |
| 40 | AGGTGTGGTGAGGTG | 26 | CAACTGTATATCAACTGAA |
| 39 | TGGA | 26 | GTCGGCG |
| 39 | GCGGACG | 26 | AAAATA |
| 39 | ATATATAT | 25 | TATTTTT |
| 38 | GGGGATTAATTAGTGGGC | 25 | CAGAGTTA |
| 38 | CCGATCAATAAGTACCCGTGTTATCACAGGG | 25 | AATTTTGGTACCAA |
| 38 | GTACTTATTGATCGGCCTTGTGACAAAACGG | 25 | AAAACGGGTACTTATTGATCGGTTCTGTTAT |
| 38 | ATATTT | 25 | CACGC |
| 38 | AACCGCCC | 25 | ATATATCTAT |
| 38 | AAAATTAATGAAC | 25 | CTAAGAA |
| 37 | ATATAAATATAAT | 24 | GTAACGCGTACGTACGCGTTATAATAT |
| 36 | TACACA | 24 | AGCC |
| 36 | AGAACT | 24 | ACTGCTATTATTCA |
| 35 | ACTATAATTGTGAG | 24 | ATATATATATGT |
| 35 | AATAATA | 24 | TGAGAATAAAATTG |
| 35 | TATATTTATA | 24 | AATC |
| 34 | ACTAGCGCTTCATTTCACTA | 24 | TGAACAGGTTATCAACTGGTCTGAACAGGTT |
| 34 | CAGAGCCGATCAATAAGTACCCGTTTTGTCA | 24 | ACCT |
| 34 | TATATATATA | 24 | AGTAC |
| 34 | AGCACC | 24 | GACGAGC |
| 34 | GCGGGG | 23 | ACACTACACTATACT |
| 33 | TGAGGCTAAATTA | 23 | AAGTACCCGTTTTATAACAGAGCCGATCAAT |
| 33 | CACCC | 22 | CACGCGCACACATGCACT |
| 33 | TGGTCGTAGAGAGCGGTGTGC | 22 | AATTCTGAGGCTT |
| 33 | CCTTGA | 22 | TATATATATAA |
| 33 | TTGTGAGACTCAAA | 22 | AGTTCGTAACCTGTTCAAACC |
| 33 | CACACATA | 22 | GATTTTTTGCTAACGAATGGCACCA |
| 32 | TAACCAGTTCAGACCAGTTCA | 21 | ACAATTTTAGACTC |
| 32 | TCTCACCATTTTAG | 21 | ACAGGGCCGATCAATAAGTACCCGTTTTTTA |
| 32 | TGTGTGTGTA | 20 | TTTGTTTTTTTT |
| 31 | CGGGTACTTATTGATCGGCCTGTGATAAAA | 20 | GAACAGGTCTGAACAGGTTAC |
| 31 | CCACTAGTTTTTTAGTTCGACGACAGATGGCG | 20 | AGTAGTAGTAGTAT |
| 31 | AACCTGTTCAAACCAGTTGAT | 20 | GCTAAAATTGTAGG |
| 31 | AACCTGTTCAAACCAGTTCA | 20 | CGAACCGGTAAAATA |
| 31 | TGAGACTAATATTG | 20 | GCCGATCAATAAGTACCCGTTTTATAACAAA |
| 30 | TGAGGTAAGGTTTCGTGTGG | 20 | TTTTATAACAGGGCCGATCAATAAGTACCG |
| 30 | AATTTTAGTCTCAG | 20 | CTAAAATTGTCAGA |
| 30 | TCACAATTTTAGCT | 19 | TGATCGGCCCTGCGACAAAACGGATACTTAT |
| 29 | ACTTTAGTTCTTAGT | 19 | ACAATTTTAGTCTA |
| 29 | GTACCCGTTTTATAACAAGGCCGATCAATAA | 19 | CAATTTTAGGCTCA |
| 29 | TTTTTGT | 19 | ACAATTTTAATCTC |
| 29 | GTTCATAACCTGTTCAAACCA | 19 | AATTTTAGTCTCA |
| 28 | ACTA | 19 | TATATTAT |
| 28 | AACC | 19 | GCGCCACCTAGTAGCCATTTTCAAAAACTCTCCCA |
| 28 | GTCGGT | 18 | CTCAAAATTTTAGC |
| 47 | ATAATATAT | 28 | CGTCGGCGT |
| 18 | GTGGTCTTGACGGTG | 11 | TCGGTGCCGGTG |
| 18 | GTTTTTTTTT | 11 | TTTATTTTTTT |
| 18 | CGCGTGAGTGCATGTG | 11 | CAATTTTAGCTCTCA |
| 18 | CAAAACGGATACTTATTGATCGGCCCTGTTA | 11 | GTGCGTGTGC |
| 18 | AGAAGAAAA | 11 | AAGA |
| 18 | CGATCAATAAGTACCCGTTTTATCACAGAAC | 11 | TTATGAACTGG |
| 18 | CGCGAC | 11 | CCCCCCGCC |
| 17 | ATTTTTTTTT | 11 | TGTGAGACTAAAAC |
| 17 | AATTTTAA | 11 | CCACCCCC |
| 17 | GGCCGATCAATAAGTACCCGTTTTATAACA | 11 | CAAAACGGGTACTTATTGATCGGCCCTGTAA |
| 17 | ACCTTGACCTTG | 11 | CCCCCCCG |
| 17 | TATC | 11 | GCGGCTG |
| 17 | ATATATCT | 11 | AACAGGTCTGAACAGGTTATG |
| 17 | CCCACCCCC | 11 | TAAACTTGTAATTTTGTATAAAAATAA |
| 17 | AAAACGGGTACTTATTGATCGGCCCTGTGTC | 11 | TTATTTATTA |
| 16 | TCTCACAATTTTCG | 11 | TTGCAGCCGTTGCAGCCACAGTCGGTG |
| 16 | AGGGCCGATCAATAAGTACCCGTTTGGTCAC | 11 | GCGAGCGAGTGA |
| 16 | TTTTATTTT | 11 | GTGAAGTTTCGTGTGGTGAG |
| 16 | ACAATTTTAGCCTT | 11 | ACCAGATTTTTTGCAGACGAATGGC |
| 16 | TTTTATTT | 11 | TTAACTATAAATAAATAATTA |
| 16 | TGGGCGGGGGATTAATTAG | 11 | AAAATTTAGCCTC |
| 16 | TGCTGGTGGAGGCGCTGCCCTCGC | 11 | GTGTGG |
| 16 | CCCCCCACCC | 10 | CTAAAATTGTGAAGA |
| 15 | TCC | 10 | AGAGACAACAACATT |
| 15 | AATAAATAATA | 10 | AATTTTAATCTCAA |
| 15 | GCCGATCAATAAGTACCCGTTTTAAAACAGG | 10 | ACGGGTACTTATTGATCGACCCTGTGATAAA |
| 15 | GCCCTGTGACAAAACGGGTACTTTTTGATCG | 10 | CACACACA |
| 15 | TGAGGTGACG | 10 | GCCGATAAATAAGTACCCGTTTTATCACAGG |
| 15 | CACCTCACCC | 10 | CCACAGCAGCCGACGCCCGTCATCGCGGCA |
| 14 | CGGCGGTGG | 10 | CCGATCAATAAGTACCCGTTTTAACACAGGG |
| 14 | TTTTTTTTTGT | 10 | AAACGAGTACTTATTGATCGGCCCTGTGACA |
| 14 | CACAGCAACAACGGCGAC | 10 | CAAAACGGGTACTTATTGATCAACCCTGTGA |
| 14 | TTCAAACCAGTTCATAACCAG | 10 | CGCCCCCCCCCC |
| 14 | CCCCCCCCCG | 10 | CAACTGAATATCAACTGAA |
| 14 | TTTTAGCTCACAA | 10 | GAACGAGCGAGC |
| 13 | AGGTGGCCTACAAAAA | 10 | CAATTTTAGCATCA |
| 13 | TATAATGTAG | 10 | AATTCTTTATTAAAGACACAC |
| 13 | TGTTGC | 10 | CGCCCCCCCCC |
| 13 | TTGTCC | 10 | TGCGTGCGTGCT |
| 13 | CGACGAGAC | 10 | GGCGG |
| 13 | GTTTCGCGGTCGGCG | 10 | GAGTA |
| 13 | TCGCAC | 10 | TATTGATCGGCCCTGTTACAAAACGGGTACT |
| 13 | TTGAG | 10 | TCGCTCGT |
| 13 | CGGCGACGGCGG | 10 | GCCGATCAATAAGTACCCGTTTTGTCACAAA |
| 12 | TGCCGT | 10 | TGTTTTCAAAATTCTGTTTTATTACCT |
| 12 | GCTGGA | 10 | ATCAATAAATACCCGTTTTGTCACAGGGCCG |
| 12 | ATCAATAAGTACCCGTTTTATCACACGGCCG | 10 | GGGTACTTATTGATCGGCCCTGCAATAAAAC |
| 12 | CTCCGG | 10 | TAATTTTAACAGTCTTTACTG |
| 12 | CTTATTGATCGGCCCTGTGATAAAACGGTA | 10 | TCGTTATTTTAACGGC |
| 12 | ATAACGCGTACGTACGCGTTACGTAATA | 10 | CCGATCAATAAGTACCCGTTTTATAACAGGA |
| 12 | ATATATATCTAT | 10 | CTAAAATTTTGAAA |
| 12 | GCCGATCAATAAGTACCCGTTTATCACAGA | 10 | CAGCA |
| 12 | AATATAAAAT | 10 | CCATTTTCAACTTGGTGTC |
| 12 | ACTACATTAC | 10 | AAAACGGGTACTTATTGATCGTTCTGTTAT |
| 12 | CCCCGCC | 10 | TAAGTACCCGTTTTATCACAGGACCGATCAA |
| 12 | AAACGAGTACTTATTGATCGGCCCTGTGATA | 10 | GATCGGCCCTGTGACAAAACGGTACTTATT |
| 11 | AACTGGTCTGAACAGGTGTTTGAACTGGTTACC | 10 | CACACACACGCA |
| 11 | TACACG | 10 | GTGTGTGTGTAT |
| **Selenophorus alternans #SRR5651490)** | | | |
| 22035 | CGCT | 127 | CACAGGGTCGATCAATAAGTACCCGTTTTGT |
| 6405 | TGAGACTAAAATTG | 124 | AACAGGTCTG |
| 4206 | GGTGA | 123 | ACGCGTACGTACGCGTTATATTACGTA |
| 2480 | GCAC | 121 | TATATTA |
| 2096 | AAT | 120 | TTGACCT |
| 1932 | TACACTATAC | 118 | ACGGATACTTATTGATCGGCCCTGTGACAAA |
| 973 | GCG | 115 | AACTAGCGCTCCATTTCACT |
| 841 | AATAAGTACCCGTTTTATCACAGGGCCGATC | 115 | TTG |
| 771 | AACCTGTTCA | 114 | GCGTGTGT |
| 745 | CACCA | 114 | GCGTGTGC |
| 683 | CCGATCAATAAGTACCCGTTTTATAACAGGG | 112 | GGT |
| 647 | AATTTTAGTCTCAA | 112 | GTCGCC |
| 633 | AATA | 109 | GGCGGT |
| 612 | AGAATTTAGCCTC | 109 | GTTCGTTATTTTACCG |
| 569 | ATAAGTACCCGTTTTGTCACAGGGCCGATCA | 109 | CTTATTGATCGGCTCTGTGATAAAACGGGTA |
| 569 | TAC | 108 | GAGACTAAAATTAT |
| 526 | TGTTT | 102 | CCGTTTTATAACAGGGCCGATCAATAAGTAT |
| 516 | AAG | 100 | GTGTGT |
| 510 | GAACTGGTCTGAACAGGTTAC | 99 | CTGTTCAGACCAGTTCAAAC |
| 448 | TCGTGTGGTGAGGTGAGGTT | 98 | AAAACGGGTACTTTTTGATCGGCCCTGTGAT |
| 417 | ACCAGGACCTCATGTCCCA | 97 | TGTG |
| 409 | AAAC | 97 | ACTC |
| 390 | CCTGGTGGCGCGTGGCG | 97 | TTTATATA |
| 384 | AGTGAGCG | 97 | CCGGTG |
| 355 | CTTCGG | 96 | AGCAGCATCTCCAACCAGCGAG |
| 300 | GTCTCACAATTTA | 95 | GGGCGGC |
| 291 | ATAT | 95 | GCCGATCAATAAGTACCCGTTTTATCGCAGG |
| 290 | CACAATTTTAGTTT | 89 | ACAGGGCCGATCAATAAGTACCCGTTTATA |
| 279 | GCGCC | 89 | TAATA |
| 273 | TAAG | 88 | CACTAATTAATCCCCGCC |
| 272 | ACAATTTTAGCCTC | 87 | ACCTTGACCTTTG |
| 266 | CACTA | 86 | TATTT |
| 264 | GCT | 86 | AATAAAATA |
| 253 | TACA | 86 | GGTTAGCAACTGGTCTGAACA |
| 237 | CGACGC | 85 | AAATAAT |
| 226 | CATATA | 81 | TCTCACAATTTTTG |
| 222 | TACTA | 80 | TAATATTAATAATTAA |
| 220 | GTCT | 78 | CTATCTCTATCCCTATCTCT |
| 214 | TGGTTTGAAC | 77 | GCCGAATTCGCGAATTCGG |
| 204 | TGTGAGACTAAATT | 77 | GCCGCT |
| 201 | GGCTAATTCTGA | 76 | TTAATTAGTGGGCGGGGT |
| 195 | GTTTCTTCGAGTGTA | 76 | TATATATATT |
| 192 | CGAGAAAGCGAG | 74 | TACGTACGCGTTATATTATGTAACGCG |
| 192 | AATTTTAGTCTTAC | 73 | CGA |
| 192 | CACGCA | 72 | GCTAAATTCTGGG |
| 189 | TAACCTGTTCAGACCAGTTCA | 70 | CTAAAATTGTGGGC |
| 188 | ATATAT | 70 | TTGACC |
| 184 | CAT | 68 | TGCGGC |
| 177 | GATCAATAAGTACCCGTTTTATCACAAGGCC | 68 | TTGACTGTAATTTTAACAGTC |
| 175 | TGTATATA | 67 | AATT |
| 172 | GCTAAAATTGTGGG | 66 | TCGGTCGC |
| 161 | AACCTGTTCAGACCAGTTGAT | 65 | GGCCGATCAATAAGTACCCGTTTTATTACAG |
| 148 | AACACA | 64 | TATTATTAATTATTA |
| 147 | GGTACTTATTGATCGGCCCTGTGATAAACG | 64 | ACCTGTTCAGACCAGTTGATAACCAGTTAAA |
| 140 | GGTGTGGTGA | 63 | AGAGACAACAACATT |
| 134 | TAGTCCCACAATTT | 63 | CGTGTGTGTG |
| 133 | CAGGGCCGATCAATAAGTATCCGTTTTATCA | 62 | AATTTTAGTCTCAG |
| 130 | TATATCTATCCCTATCTCTC | 61 | GGCACTGGGGTGTGTCCTACACGT |
| 129 | AAATTGTGAGACTT | 61 | ACCAGGACCTCATGTCCTCA |
| 60 | TATATATATG | 64 | ACCTGTTCAGACCAGTTGATAACCAGTTAAA |
| 59 | AATAAAATTTTTAAAAA | 63 | AGAGACAACAACATT |
| 58 | TAGTCTCACCATTT | 63 | CGTGTGTGTG |
| 58 | CGAAAAGAGAGTCATAGTTACTCCCG | 62 | AATTTTAGTCTCAG |
| 57 | TAGTATA | 61 | GGCACTGGGGTGTGTCCTACACGT |
| 57 | GCCGATCAATAAGTATCCGTTTTGACACAGG | 61 | ACCAGGACCTCATGTCCTCA |
| 57 | TTCGGGGGAGTAACTATGACTCTCTT | 41 | AAACAA |
| 56 | TAACCTGTTCAAACCTGTTCAGACCAGTTGA | 41 | GCGCCACCTAGTAGCCATTTTCAAAAACTCTCCCA |
| 56 | GGCCGATCAATAAGTACCCGTTTTATAACAA | 40 | TGTGTGTGTGCG |
| 56 | AAAAAAAC | 40 | GAACAGGTCTGAACAGGTTAC |
| 55 | TCTCTA | 40 | CTTATTGATCGGCCCTGTGATAAAACGGTA |
| 55 | CGATCAATAAGTACCCGTTTTATCACAGGC | 40 | ACCAGTTCAAACCTGTTCAA |
| 55 | GTGAGACTAAAAAT | 40 | CGAGCGAT |
| 53 | CAGAGCCGATCAATAAGTACCCGTTTTATAA | 40 | GTCGAACTAAAAAACTAGTGGCGCCATCTGTC |
| 53 | CAACGGCGACCACAG | 40 | TATATATGTATA |
| 53 | AGTAATATG | 39 | GATGGCGCCACTAGTTTTTAGTTCGACGACA |
| 53 | ACTACATTAT | 39 | CTCACGTCAC |
| 53 | TATTATATA | 39 | AATTTTAGCCTTAC |
| 53 | CAGGGCCGATCAATAAGTATCCGTTTTGTCG | 39 | CCAGTTCAGA |
| 53 | ATATAA | 39 | ATATATAT |
| 52 | CTTATTGATCGGCTTTGTGATAAAACGGGTA | 39 | AATTATTTATATATAGTTAAT |
| 51 | TAAC | 39 | AAAACGGGTACTTATTGATCAGCCCTGTGAT |
| 51 | ATAAGTACCCGTTTTGTCACAGAGCCGATCA | 38 | ATAATAAATA |
| 50 | AACTGGTTATGAACTGGTCTG | 38 | CGAAAAGAGAGTCATAGTTACTCCC |
| 50 | TTGCTCG | 38 | AACCTGTTCAAACCAGTTGAT |
| 50 | AAATTTAGTCTCA | 38 | TGAGACTAATATTG |
| 49 | GGTGAGGTGAGGTGT | 38 | GGCTTCCGCTTCCGCTTC |
| 48 | TGAGGTAAGGTTTCGTGTGG | 37 | AGACCAGTTCTAACCTGTTC |
| 48 | TAATTTTGTTCAT | 37 | ATTTTAATCTCACA |
| 48 | ACTGCTATTATTCA | 36 | GAATTCGCGAATTCGGCC |
| 48 | AGAACT | 36 | GTGTATGTGT |
| 48 | CACAGGGCCGATCAATAAGTACCCGTGTTAT | 36 | CTAAAATGTGAGA |
| 47 | CACAATTTTAGTCA | 36 | AATTCTTTATTAAAGACACAC |
| 47 | AATAATAT | 36 | GTTCAAACCAGTTCATAACCT |
| 47 | TAGTTAGTGAAATGAAGCGC | 36 | TTTAGGCTCACAAT |
| 47 | GAGA | 36 | AGGCTAAATTCTA |
| 47 | CACAATTATAGTCT | 35 | TTTCGTGTGGTGAGGTGAAG |
| 46 | CGCCCG | 35 | GTGGTCTTGACGGTG |
| 46 | GGCCGG | 35 | CACGCGCACACATGCACT |
| 46 | TGGGCGGGGGATTAATTAG | 35 | TAAACTTGTAATTTTGTATAAAAATAA |
| 46 | ATATATATAT | 34 | ACAATTTTAGTCTA |
| 45 | GGCCGATCAATAAGTACCCGTTTTGTCACAA | 34 | AAAAAACAAA |
| 45 | GGTAAAATACGAACC | 33 | ATTAA |
| 44 | TATGTG | 33 | ACCCGTTTTATCACAGGGCCGATCAATAAAT |
| 44 | CACGCGCACATGCACT | 33 | GCTAAAATTGTAGG |
| 44 | TTTAGCCTCATAA | 33 | TTTTTA |
| 43 | CGTCGCT | 33 | GTTTTTT |
| 43 | CTGTAACT | 33 | TATATCTATA |
| 42 | ACTCAGT | 33 | AAATTGTGAAGCTA |
| 42 | ACTACACTATACTAC | 33 | GGTGTG |
| 42 | TGGTCGTAGAGAGCGGTGTGC | 32 | GTACTTATTGATAGGCCCTGTGACAAAACGG |
| 42 | CTAAGAA | 32 | CGTTTTGTAACAGGGCCGATCAATAAGTATC |
| 42 | CACCAG | 32 | TATTGATCGACCCTGTGATAAAACGGGTACT |
| 42 | GTGCG | 32 | CCGGCT |
| 42 | GACTAAAATTTGA | 32 | TTACGAACTGGTTTGAACAGG |
| 41 | ATGTGTGT | 30 | CACAGGGCCGATCAAAAGTACCCGTTTTAT |
| 41 | AGTAGTAGTAGTAT | 30 | TTATATATC |
| 41 | GATCGGCCCTGTGTCAAAACGGGTACTTATT | 30 | AACC |
| 41 | CGCTCGTT | 30 | TTGATCGGCCTGTGACAAAACGGGTACTTA |
| 41 | ATATAAATATAAT | 30 | CATATTTA |
| 28 | GAGTA | 30 | TACACTATACTATAC |
| 28 | TAGA | 30 | GGTACTTATTGATCGGCCCTGTTATAAAAC |
| 28 | GCCGTCGCC | 29 | ACTATCTC |
| 28 | GGCTAAAATTTTGA | 29 | CCGATCAATAAGTACCCGTTTGGTCACAGGG |
| 28 | TCACCTTACCTCACC | 29 | CGTTTTGTCACAGGGCCGATCAAAAAGTACC |
| 28 | CCCTCGCTGCTGGTGGAGGCGCTG | 28 | AAACGGGTACTTATTGATCGGCCCTGTGAC |
| 28 | TCGACGTGA | 19 | TTTTTTTTTA |
| 28 | AAGTACCCGTTTTAAAACAGGGCCGATCAAT | 19 | TATAGTGTAA |
| 27 | ACAAGG | 19 | TGAACAGGTTATCAACTGGTA |
| 27 | TAGG | 19 | GTGAGACTAAAACT |
| 27 | TTTGTTTTTTTT | 18 | CGTTTTATAACAGGACCGATCAATAAGTACC |
| 27 | CGCTCGATCGCT | 18 | CTCGTTCGCTCG |
| 27 | CGCCACCGC | 18 | ACCCGTTTTATAACAGAACCGATCAATAAGT |
| 27 | GGTTATGAACT | 18 | CGACGAGAC |
| 27 | ATCCCTATCTCTCTATTTCT | 18 | GCCGAATTCGACGAATTCGG |
| 27 | AAACGGGTACTTATTGATCGGCTCTGTGAT | 18 | CCCCCCCG |
| 27 | TAAAATTGTGAGAA | 18 | CAGGGCCGATCAATAAGTACCCGATTTATAA |
| 27 | AATATATATAT | 18 | TATTTTATAT |
| 27 | GTACA | 17 | TATATATATATC |
| 27 | AAAACGGGTACTTATTGATCGGCCCTGTGTT | 17 | TAATAAATAAA |
| 26 | GATCGGCCTTGTGATAAAACGGATACTTATT | 17 | CCCCCGCCC |
| 26 | AAAACGGGTACTTATTGATCGGCCCTGTCAT | 17 | ACTACATTAC |
| 26 | AAACGGGTACTTATTGATCGGCCTGTTATA | 17 | ATTTTTTTTTT |
| 26 | ATAATAATAT | 17 | GTACTTATTGATCGGCCCTGTGACAAAACGA |
| 26 | ATAATATTA | 17 | ATACAAAT |
| 26 | TGAACAGGTTATGAACAGGTT | 17 | GAATTAAGCCTCA |
| 25 | CAGC | 16 | ATTTTTT |
| 25 | TGTACG | 16 | CTCACAATTTTAGA |
| 25 | CGTCGGCGT | 16 | CCCCCCCCA |
| 25 | CAAAAAAAA | 16 | GTACTTATTGATCAGCCCTGTTATAAAACGG |
| 25 | TTTTTTTTTTG | 16 | TGGCG |
| 25 | CACCACACCACACCT | 16 | AAACGGATACTTATTGATCGGCCCTGCGATA |
| 24 | TATAACGCGTACGTACGCGTTACATAT | 16 | ACAGC |
| 24 | AATTATATTTCAATACAA | 16 | GGCCGATCAATAAGTACCCGTTTTTAACAG |
| 24 | GCGGTGGCGGCG | 16 | CGCGGGCTGCTGCGC |
| 24 | TTATATATATTTGA | 15 | AATTAAATT |
| 24 | AAATTTTGGTACCA | 15 | CCTCACCTCA |
| 24 | CCCCCCACCC | 15 | CCCCCCCCCGC |
| 23 | TTAAGTATTTCCACC | 15 | GCGTGTGCGTGC |
| 23 | CCGATCAATAAGTACCCGTTTTATAACAAAG | 15 | ATATATCT |
| 23 | CAAACCTGTTCAAACCAGTTGATAACCTGTT | 15 | CTAGCGCTTCACTTCACTAA |
| 22 | TGCGGCG | 15 | ATTTTAGCCCACA |
| 22 | CACAGCAACAACGGCGAC | 15 | CGCGGTCGGCGGTTT |
| 22 | AGGTCAAAGGTCAATGACCT | 15 | AGAGAGAAAG |
| 22 | CAACTGTATATCAACTGAA | 15 | GACGAATGGCACCAGATTTTTTGCA |
| 22 | TCAATAAGTACCCGTTTTATCACAGGACCGA | 15 | GGTTATGAACAGGTCTGAACA |
| 22 | CGGGGCG | 15 | GACCGC |
| 21 | TGCCCG | 15 | CCGCCCCCCC |
| 21 | GTACTTATTGATCGGCCCTGTAATAAACGG | 15 | TTGTAAAACTAAAA |
| 21 | TATTTTTT | 15 | GGGGTTCAGGCCATTGGCG |
| 21 | GTACTTATTGATCGGCCCTGTGATAAAACGA | 15 | TCGTTATTTTAACGGC |
| 21 | TTAACTATAAATAAATAATTA | 15 | AGGTCATTGACCTAGGTCAAGGTC |
| 20 | AACTGGTTACGAACTGGTCTG | 14 | AACTGGTCTGAACAGGTGTTTG |
| 20 | CACCC | 14 | TCGGTGCCGGTG |
| 20 | GCCGATCAATAAGTACCCGTTTTGTCACAAA | 14 | CAGAAATTAGCCT |
| 20 | AAAAAATCTGGTGCCATTCGTCAGC | 14 | TGTCGTG |
| 19 | GATAAAACGGGTACTTATTGATCGGCCCTAT | 14 | AGGTGAGGTTTCGTGTGGTA |
| 19 | CCACAGCAGCCGACGCCCGTCATCGCGGCA | 14 | GTAACGCGTACGTACGCGTTATAATAC |
| 19 | CGTCGGTCA | 14 | TCTCTCTCTCTT |
| 19 | TTAAAATT | 14 | ACGGGTACTTATTGATCGGCCCTGTATAAA |
| 13 | CGATCAATAAGTACCCGTTTTATCACAGGGA | 14 | CACGCGCACACGCACT |
| 13 | ATTGATCGGCCCTGTGACAAAACAGGTACTT | 14 | GTAAGCAGTTGATAACCTGTTCAAACCTGTTC |
| 13 | AGTACTTTAGTTCTT | 13 | ACACTACACTACACTATACT |
| 13 | AATAAAATTTATAAAAA | 13 | CATCGC |
| 13 | AATTTCAGTCTCAC | 13 | TGAACAGGTTTGAACAGGTC |
| 13 | ACCTTGACCTTG | 13 | TTTTAAATTTAA |
| 13 | TCACCCCACC | 11 | ACACAAACACAC |
| 13 | CAATA | 11 | AGACGGCTAGTGAATGAGT |
| 13 | GGCCGATCAATAAGTACCCGTTTATCACAA | 11 | AAGTACCCGTTTTATCACAGGGCCGATAAAT |
| 13 | CTATCCCTATCGCTCTATAT | 11 | GAGCGG |
| 13 | CTCACCACACAAAACCTCAC | 11 | AGTAGTAGTAGTGAT |
| 13 | TTTTTTTTTC | 11 | GTGCGTGTGC |
| 13 | ACAGGGCCAATCAATAAGTACCCGTTTTATA | 11 | CCGACGCCGACGACG |
| 13 | ACACACACACAT | 11 | CACCTCACCTCACCC |
| 13 | TTCATTTTCTTGTTC | 11 | CAAAAATCTGGCGCCATTCGTCAG |
| 13 | CGCCGCCGCCGT | 11 | GTTCATAATGAGACACCAA |
| 13 | CAGCTC | 11 | CCCCCCG |
| 13 | ATTAAT | 11 | TTGCAGCCGTTGCAGCCACAGTCGGTG |
| 12 | ACCCGC | 11 | ACGCGTTCGTACGCGTTATATTACGTA |
| 12 | GCCGTCC | 11 | TTAATTAGTGGGCGGGGTT |
| 12 | TCAATAAGTACCCGTTTTATTACAGGGCTGA | 11 | AATTTTAGTCTTCAC |
| 12 | TGCAAAAAATCTGGCGCCATTCGTC | 11 | AAGTTGAAAATGGGACACC |
| 12 | GTTTTATCATAAGGCCGATCAATAAGTACCC | 11 | AAACGGGTACTTATTGATCGGCCCTGTGAA |
| 12 | GGCGGTTG | 11 | CTAAAAGTGTGAGA |
| 12 | CACTGTACTAC | 11 | CACAGGGCCGATAAATAAGTACCCGTTTTGT |
| 12 | GGTGAGGTAA | 11 | GTCC |
| 12 | GGTTTCAACTGGTCTGAACA | 11 | TATATATATAAA |
| 12 | ATCC | 11 | AAAATTGTCAGACT |
| 12 | ATCAAAAAGTACCCGTTTTATAACAGGGCCG | 11 | ACTCA |
| 12 | AAAATTGTGAGACG | 11 | AATTAAACCCCCCCCCCC |
| 12 | TGTTGTAAATCACTAGATTAAGAAAACATTG | 11 | CACAAATA |
| 12 | GTTTTATCACAAGGCCGATCAAAAAGTACCC | 11 | CGCACCGCGC |
| 12 | TAACCAGTTCAAACCAGTTCA | 11 | AAAACGGGTACTTATTGATCGGCCCTGTGAG |
| 12 | CTCCGGG | 11 | AAAACGGGTACTTATTGATCGGCCCTGTTAC |
| 12 | TGACCTTTGACCTAGGTCAA | 11 | CCCGTTTTATCACAAGGCTGATCAATAAGTA |
| 12 | TACTTATTGATCGGCCCTGTTAAAAAACGGG | 10 | GGTGCGC |
| 12 | TTTTTTTAT | 10 | GGGGGC |
| 12 | AAAACGGGTACTTATTGATCGACCCTGCGAC | 10 | TAAAACGGGTACTTATTGATCGGCTCTGTGG |
| 12 | CCAAAATTTTAGCC | 10 | CTTATTGATCGGCCCTGTTATAAAACGGGAA |
| 12 | ATTATAAT | 10 | GTACTTATTGATCGGCCCTGCAATAAAACGG |
| 12 | AACCCGCCGCGA | 10 | ATTGATCGGCCCTGTGAAAAAACGGGTACTT |
| 12 | TTTTTTTTTTAT | 10 | AGTGAGCGAGCG |
| 12 | CCTTATCAGTTGTGACGTCA | 10 | GGGCG |
| 12 | ATAACGCGTACGTACGCGTTACGTAATA | 10 | CAAAACGGATACTTATTGATCGGCCCTGTAA |
| 12 | CCCGTTTTGTCGCAGGGCCGAACAATAAGTA | 10 | ATAATTA |
| 12 | GCCGCCACT | 10 | TACTTATTAATCGGCCCTGTGATAAAACGGG |
| 11 | CTCTCTTT | 10 | GGTAA |
| 11 | AAATTTTAGTCTCAC | 10 | TATACAATA |
| 11 | CGTGAGTGCTTGCGTA | 10 | TAAGTAC |
| 11 | GTGAGACTAAAATA | 10 | TGCTGT |
| 11 | TATAATATTTA | 10 | GCAG |
| 11 | GGCCGATCAATAAGTACCGTTTTATCACAA | 10 | CGCCCCCCCCCC |
| 11 | AGATTTTTGCAGACGAATGGCGCC | 10 | CATTACATA |
| 11 | AAATATAAGTGTTATTAT | 10 | AACCTGTTCAAACCAGTTCATAACCAGTTCA |
| 11 | ATATATATATATGTAT | 10 | CCCGTTTTATAACAGGGCCGATCAATAAATA |
| 11 | TGGAGATGGTCTCGCTGCACG | 10 | CCTGGTTGTGGACATGAGGT |
| 11 | AGTGCTTGCGTG | 10 | GCCGTTGTTGTGT |
| 11 | ATAACTTAT | 10 | CCTAA |
| 11 | GGGGATTAATTAGTGGGTG | 10 | CAGGGCCGATCAATAAGTACCCGCTTTATAA |
| 11 | GGTACTTATTGATCGGCCCTGTGACAAAAC | 10 | CTTATTGATCGGCCCTATGACAAAACGGGTA |
| 10 | CGTTTTATCGCAGGCCGATCAATAAGTACC |
| 10 | CGCCCGA |
| 10 | GGCACT |
| 10 | TGAGGTAAAGTTTCGTGTGG |
| 10 | TTGGTAACCAGTTCAAACACCTGTTCAGACCAG |
| 10 | AAACCTGTTCAGACCAGTTCG |
| 10 | TACTATTATAAT |
| 10 | GAAACGAGTGAGTCGATGT |
| 10 | CAAACACA |
| 10 | AGTTCTCT |
| 10 | TTTGAACAGGTTACGAACTGGTCTGAACAGG |
| 10 | TATATATATATATA |
| 10 | CAAAAAATCTGGTGCCATTCGTTAG |
| 10 | TTTAGTCTCAAAAA |
| 10 | TGAC |
| 10 | ACAATTTTAGCTC |
| 10 | ACAGGTTTGAACTGATCTGA |
| 10 | GTGTGTGT |
| 10 | ACCACGACG |
| 10 | AAAAACGCGAACCGGT |

| **Miquihuana rhadiniformis (#SRR3421278)** | | | |
| --- | --- | --- | --- |
| 3256 | TGTA | 36 | ACATA |
| 2106 | GTGTCATGAATTAAATATTTTATA | 35 | CATACA |
| 1989 | AAATAAAATATTTTATAGTGTCATG | 34 | CTATTTTAAATTTC |
| 1323 | TAAGTAAAATAAAAG | 34 | ATAAAATATTTTATTTCATGACACA |
| 1088 | GATG | 33 | AATTATGGACAG |
| 1043 | GTCT | 33 | AGCAGTCCGAGCCA |
| 836 | **GTTAG** | 33 | CGAAAAAGGTTAGCGGA |
| 829 | TAAG | 30 | AGCAGACCGAGCCA |
| 803 | TAAATAGA | 29 | CTTTG |
| 576 | AAAT | 29 | ATTTTTG |
| 566 | CTATAAAATATTTTAATTCATGACA | 29 | AATTAAATATTTCATAGTGTCATG |
| 530 | CTATAAAATATTTATTTCATGACA | 29 | TATTACCCG |
| 436 | AAAC | 28 | AATTGAA |
| 328 | TTC | 28 | ATC |
| 285 | GTCGGTCT | 28 | ATGGATAACGCTATATAAA |
| 275 | CTAT | 28 | GAAATACAAAC |
| 235 | TAAAATAAAAG | 28 | ATAAATAAATAC |
| 234 | AATATTTTATAGTGCCATGAATTA | 27 | TTAA |
| 219 | CATACACA | 27 | TCTGGACAGAAT |
| 185 | CTCA | 26 | ATGTGTGTGTGT |
| 181 | TCATGAATAAAATATTTTATAGTG | 25 | TTTTTTACTATT |
| 170 | AAAACAGAC | 25 | TTTTGCTTTTACTATTAAAGCCTCTTG |
| 154 | AGATACAT | 24 | ATATGATTATG |
| 143 | CTAA | 24 | ATTCATGGCACTATAAAATATTTTA |
| 126 | CTTT | 24 | CATGAATTAAATATTTTATAGTGA |
| 98 | TATCC | 24 | ATCAAATA |
| 97 | CACA | 24 | AATAAAAACTAATTTTTATTTATATA |
| 89 | TTTATCCATTCTATATAGCG | 24 | CTGTCTTAAAATCTAGT |
| 87 | CCTG | 24 | TATAATTTTTTAAAAAT |
| 84 | AATAAAAACTAATTTTTATTTAAATA | 23 | AATATTTCATAGTGTCATGAAATAA |
| 73 | TTG | 23 | AAGCAAAC |
| 70 | ATAGTGTCATGAAATTAAATATTTT | 23 | TTTTTATTTTACTTA |
| 68 | GTAG | 22 | ATTTTTTTT |
| 64 | GCATAACTCAA | 20 | ATTTTTTTTTT |
| 64 | TAAAATATTTTATTTCATGGCACTA | 20 | CTTAAAACCTAAATACATTTG |
| 62 | ATCCACAC | 19 | AATTAAAAATTTAAAGTTTGAA |
| 60 | CTTG | 19 | ATTTTAA |
| 57 | TACATAAA | 19 | TTTTATTTTACTTAA |
| 57 | ACGAAAAAGGTTTGCGG | 18 | TATAATTTTTAAAAAT |
| 54 | ACTATCGACTTCG | 17 | TTTTG |
| 53 | ATTTCCTATTTAA | 17 | GTGGT |
| 53 | TGAA | 17 | ACTTACTTTATTTT |
| 47 | CGAC | 17 | AAATAAAAGTAAGT |
| 46 | GATAAAACA | 16 | TGCCATGAATAAAATATTTTATAG |
| 45 | ATCCACACATCC | 16 | AAAATTTT |
| 44 | ATATTATCG | 15 | AGTCGA |
| 44 | TTTTA | 15 | TATTATCAATTTCC |
| 42 | AGAGATAG | 14 | AAGAGCGTCGTGTAGGGA |
| 42 | GTGTATGTGT | 14 | TTTTTTTA |
| 42 | AGTC | 14 | ATTATTTGATTAATCG |
| 42 | AATTAAGGCTGCTTTC | 13 | AATAAAAACTAATTTTTATTTATTTA |
| 40 | TAT | 13 | GGGGAGGGG |
| 40 | TGTGTGTT | 13 | ATTCATCC |
| 39 | TTTTTTATTT | 13 | AAATTAAATATCTTGGTATAATCATAGGTAATTG |
| 39 | AATAG | 13 | AAATAAAAGTAAATA |
| 38 | GTGTGT | 12 | AGCGTTTATCCATTCTAAAT |
| 37 | GTCTTTAAGGACCG | 12 | CATGAAATAAAATATTTCATAGTGC |
| 37 | ACAGCA | 12 | TTGTTTTGT |
| 36 | TAAATAAAAATTAATTTTTATTTATT | 11 | TGTCTGTTTGATT |
| 11 | AAATTTAGAA |
| 11 | TCGATAAATA |
| 11 | ATAAAATTTTTAATTCATGACACT |
| 11 | TCGAATAT |
| 11 | TTTTTTATTCTTT |
| 11 | ATTTTTAAAA |
| 11 | CATCAACTATTACATTGTTGT |
| 11 | ATATCGAT |
| 10 | TTAAATTAGGAAAT |
| 10 | GCGTTATTTAACGCAGAGTTTCTTG |
| 10 | GTCTGCTTGGCTCGG |
| 10 | TCGTTTGTGCT |
| 10 | AAGTAAGTAAAAGAA |
| 10 | CAGTATA |
| 10 | ATCTTTATAAAACAGAATTACTGCAGAGGAACTC |

| **Pseudamara arenaria (#SRR3421213)** | | | |
| --- | --- | --- | --- |
| 1539 | GTAAAGTGA | 10 | TAATTAGC |
| 1481 | AGTTAGCT | 10 | AACTAACA |
| 1172 | **TTAGG** | 10 | GAACCGAACCGTTTAATA |
| 841 | TGTC | 10 | TTATTTATTTTTA |
| 517 | ATT | 10 | AAAAC |
| 502 | CGAACCGGTTTATTGAAC | 10 | CTTGCTG |
| 395 | TAAC |
| 334 | GTAACTTGTAACT |
| 329 | CACACAGACAGA |
| 322 | TTAATCGTAATT |
| 260 | TGAC |
| 241 | GTAAAAGTG |
| 225 | TATT |
| 206 | AGTTACA |
| 198 | TAAACGGTTCGGTTCAA |
| 179 | TGTCTGTG |
| 168 | TTCT |
| 138 | AACCGGTTCGTTCAATA |
| 134 | TTTTTAGCAAC |
| 130 | CCAACTAA |
| 98 | TTTACTTAC |
| 95 | TGTA |
| 93 | CCAATTTCA |
| 92 | GTTAACTA |
| 90 | GAACCGAACCGTTTACATAAACCGGTTTATT |
| 89 | CACA |
| 86 | CAACTTCCGGTACC |
| 77 | ACTCC |
| 76 | TCGCGTTAAGGAATTCGAGCATA |
| 66 | TTTG |
| 64 | CGGTTCAATTCAATAAAC |
| 52 | TCTTCTAAATTTCTAGAATTTACTTCCAATTTC |
| 52 | GTTGAA |
| 51 | CACTTTGAC |
| 51 | CGGTTCGATTCAATAAAC |
| 50 | GCA |
| 48 | CCGGTTTATTGAACTGAA |
| 48 | TCTTG |
| 46 | GTTTTTAGCAA |
| 44 | TTTTTA |
| 42 | AATGG |
| 42 | TGTGTG |
| 42 | ATCT |
| 40 | TTC |
| 40 | ACTCAACTCGACTA |
| 39 | AAAAAACGAACTAA |
| 38 | GTGTGC |
| 38 | AATTTT |
| 37 | ACCACCCAAAAATCACTCTTTACCGTCC |
| 37 | CAACTTCCGGTTT |
| 37 | AGTGTCCGTTATAAGAG |
| 37 | AAAAAAACGAACT |
| 36 | TTG |
| 36 | GTTCGGTTCAATAAACCGGTTCTATATAAACG |
| 35 | TTTAAATAGTTATA |
| 35 | TAACGCGATATGCTCGAATTCCG |
| 33 | TGTCTGTGTGTG |
| 32 | TGTCTGGCTGTG |
| 32 | CTATTTAAATCCATAAATTGAAATATAA |
| **Bembidarenas (#SRR2939023)** | | | |
| 759 | TTTCAAAGTTTGGGATCT | 30 | ATTTTTT |
| 370 | **GGTTA** | 30 | ATCTAAGAACCTGAAATAAAAAACTAT |
| 355 | ATA | 30 | TCTCAAT |
| 250 | TATG | 29 | CAGACAGACCTGTTTCTCACCCAACAGCTAAA |
| 209 | CCAA | 28 | TTTCTTTTTTT |
| 191 | ATAA | 28 | GATCTTTTCAAAGTTTGC |
| 190 | TATCGAAGG | 26 | TGGAA |
| 180 | TAAAGAAA | 25 | AGGGGGGGGGGG |
| 152 | TTCTCT | 24 | TTTTTTATTTCAGGTCTTAGATATAG |
| 122 | GTGAGGGTAG | 24 | TTTTTA |
| 118 | TCGATACTT | 24 | TAAATGAA |
| 96 | ACCTGTACAGAT | 23 | CTCAGTAGTTGTTGTAGC |
| 95 | CTCTT | 23 | TAAAATCAACCCC |
| 90 | TTCTTAGATTAAGTTTTTTTATTTCAGG | 23 | GTACCCGGTTCCAG |
| 84 | AAC | 23 | TTTGGGTAATGTTGGTAATG |
| 77 | GGGGGGGAG | 22 | AAACTTCCAAACTACCCATT |
| 73 | CATTCC | 22 | CGGGGGGGGGG |
| 68 | TTTTTTATTTCAGGTTCTTGGATTAAGT | 21 | AAAAAAAAATAA |
| 66 | GATAGGAAAAG | 20 | ATAACCTTCATTTCAC |
| 65 | TTCCAAGTACCCGG | 19 | GATAGCGAAAAG |
| 65 | GGGGGGGGAG | 18 | ATTTTTATTATGC |
| 60 | AAAATGGATAGG | 18 | TTTTCCTATCCTTTTCGCTATCCA |
| 59 | AATTATGAAT | 18 | TATGTATGTA |
| 58 | GAACGGTGGGGT | 16 | TATCCTTTTTCC |
| 57 | AAAAAAAAAAATTTT | 16 | GGGGCGGGGGGG |
| 56 | CAGC | 16 | GGGAGGG |
| 56 | TTTTCAAAGTTTACGATG | 16 | GGGGGGAG |
| 55 | CTT | 15 | AAAAAAAAAAC |
| 54 | GGGGGGAGGGG | 15 | TTTGTATTTTAAT |
| 48 | CTTA | 15 | ACTGATAAATCGTAGAACAAACAGT |
| 47 | AAAGAGAGTTGT | 15 | GGGGGGGAGGGGG |
| 47 | ATAAAAAAAA | 14 | AAACAAAAA |
| 47 | TTTTTATT | 14 | GGGGGGAAGG |
| 46 | CTTTGAAAAGATCCCAAG | 14 | AAAAAACAAA |
| 45 | TAAAA | 14 | TTAGGCACTAA |
| 44 | TTGGTACCCGGTTC | 14 | CGGATAGTGGATT |
| 43 | TATTTTTTT | 14 | ACCGGGTACTAAGA |
| 42 | CCCGGTTCCGGGTA | 13 | GAT |
| 42 | CGGTTCCTAGGACC | 13 | CCCCTAAAACCAA |
| 41 | AAAAAAAAAG | 13 | AGTTTTTTATTTCAGGTTCTTGGATTA |
| 40 | ACGTTGGGATCTTTTCAA | 13 | GTTTTAAAAC |
| 39 | AGTTTTTTATTTCAGGTTCTTAGATTA | 13 | AATTT |
| 39 | CCTGAAATAAAAAACTATATCTAAGAC | 13 | AAAAAAGGA |
| 38 | TGTTTTTCACCCAACAGCTAAACAGACAGACC | 12 | TTTTCAAAGTTTACGATC |
| 37 | CGATGTTTTCAAAGTTTT | 12 | GAAAATGGATAGC |
| 37 | TAAATCGTAGAACGAAGAGTAGATACTGA | 12 | CTTTTTTTTTTC |
| 37 | CATACATACA | 12 | TTTTTCTTTTCCTTTCTC |
| 37 | TTTTCAAAGTTTGAGATC | 12 | AAACCCCA |
| 37 | TTTCTTCA | 11 | CGGGGGGGGG |
| 36 | TCCTTCT | 11 | TTGGTAATGT |
| 36 | AGAGAAAGAG | 11 | AGAAAGTTTGAAAGAAAATTCT |
| 35 | TAAAAAAAAAA | 11 | CAGCTCATTCTGAAGAAA |
| 35 | TCTT | 11 | TTCCCTCTTTTTCCC |
| 34 | AAAAAGAAA | 11 | ATCTTTTCAAAGTTTAGG |
| 34 | ATAG | 11 | AGTAA |
| 33 | AACATTACC | 11 | AAATAAAAACTTAATCCAAGAACCTG |
| 32 | GGGTTTTAGGGGT | 11 | AATATTTGTCGACAAATATTA |
| 31 | GTG | 11 | ATTTTTATAATAT |
| 31 | CCCTAAAAACCAC | 10 | TGTTTCTTCAGAATAAAC |
| 10 | TCCCTTTTCCTA |
| 10 | CTC |
| 10 | TTAATAATCGA |
| 10 | TTTAGGGGTAGGTT |
| 10 | TTATTCTAAAGAAACAGT |
| 10 | AAAAGAAGGAAGAGAA |
| 10 | AAGTACCCGGTTTC |
| 10 | TGATTGA |

| **Bembidion chilioperyphus (#SRR2939027)** | | | |
| --- | --- | --- | --- |
| 846 | ATATTGAATACATGGGCACATAGCGT | 5 | TACTTG |
| 330 | **ACCTA** | 5 | TACAAA |
| 179 | TGT | 5 | CGTTT |
| 65 | ACGAT | 5 | AGTTGC |
| 52 | ATCCAGC | 5 | AGCATTC |
| 36 | GTA | 5 | GTATTC |
| 30 | TCTAATGTGCTCCAGTACAT | 5 | TGCTCGT |
| 30 | GTTG | 5 | TATAGC |
| 30 | AATGG | 5 | **AACCC** |
| 30 | CAGCAA | 5 | ACCAA |
| 29 | GAT | 5 | AGTATTTC |
| 28 | TTA | 5 | CTAAATCTTCGTTAATTCGTCG |
| 23 | TTGTGC |
| 22 | GTAT |
| 20 | GCAAAA |
| 19 | CAAA |
| 19 | CAACGA |
| 18 | GTTGACCTGAACCGAAAGGG |
| 18 | AATTTTTTGCAAAA |
| 17 | CGA |
| 17 | TGC |
| 16 | AAGTAG |
| 16 | AGCATCCC |
| 14 | CAACAC |
| 14 | TTTTGAGAGA |
| 13 | GTTGAT |
| 12 | TTTGCG |
| 12 | AGCCGCGCTAGTTTTA |
| 11 | ATTTTTCTCAAATCAATGTT |
| 11 | ATTGAGC |
| 11 | CAACAGCAA |
| 11 | CACGTT |
| 10 | TCAACCCCTTTCGGT |
| 10 | ATTCAAC |
| 9 | ACGAGA |
| 9 | CTGTAA |
| 8 | ACATTC |
| 8 | AATTTTTCTGCGAAAAA |
| 8 | CAAG |
| 7 | GGATGTT |
| 7 | CGTTTT |
| 7 | TTTTTCGAAAAAAA |
| 7 | ACAAC |
| 7 | AATTTTTCGCGAAA |
| 6 | CGATGA |
| 6 | AAAACAAAA |
| 6 | CGAG |
| 6 | AAATTGCAACGC |
| 6 | CAACTT |
| 6 | GGTAACG |
| 6 | TAAA |
| 6 | AAAATCGTCGT |
| 6 | CGCGGCTTAAAACTAA |
| 6 | CCATCA |
| 6 | TTTACG |
| 6 | GCATCTA |
| 6 | CTACTG |
| 5 | AACACTTGAAACGGATAAAT |
| 5 | CAAAA |
| **Bembidion eupetedromus (#SRR2939025)** | | | |
| 282 | AGATGCCACGCTTTGTTCTAAGCCATAGGCTG | 8 | AGCGGTTTCTG |
| 171 | CCCCAAAA | 8 | GGGGAGGGGGGGG |
| 145 | **CCTAA** | 8 | AACCAGGTCAATCTCCAG |
| 137 | TCCAT | 8 | TAAATTAAATTAATAT |
| 118 | GGTTAGGTTAG | 8 | ATG |
| 97 | AAC | 7 | TTAACATGCGACACT |
| 75 | TAT | 7 | TTTTTATT |
| 75 | TCTC | 7 | ATAGCACTGCACTG |
| 63 | ACCACCTCAATCGCGAGA | 7 | CCCTAACCTAAT |
| 54 | AAACATGTCAA |
| 48 | TGGAATGGATGGA |
| 48 | AGAGAA |
| 47 | CTCTCT |
| 47 | GCA |
| 42 | TTTA |
| 36 | GGGGGGGGGA |
| 35 | TGGA |
| 35 | TATTCGTATAAAACGCCGTTT |
| 34 | GGGGGGGAGGG |
| 33 | TACA |
| 33 | AGA |
| 32 | GGGGGGGAG |
| 31 | CCAAACC |
| 28 | AAAATGTCTTT |
| 27 | TATTTTTTT |
| 26 | TTGGCCATA |
| 25 | AAAAAATAAA |
| 25 | CTTTGTTCTAAGCCATAGGCTAAGATGCCACG |
| 25 | CTAAAAACCGCTCAGAAACCG |
| 25 | GTTGTTG |
| 22 | ATTTTTT |
| 22 | TTCT |
| 21 | CCCCAATCGAGCGCACCA |
| 21 | GTCGCGTTAGTTTTAA |
| 21 | TTATAAAATATT |
| 20 | GCGA |
| 18 | GGGTTTA |
| 17 | GTCTGTGT |
| 17 | TCTCTTTC |
| 14 | GGGGGCGGGGG |
| 14 | TCCATTCCA |
| 12 | AGCGCACCACCTCAATCG |
| 11 | TAG |
| 11 | CTAACGCGACCTAAAG |
| 10 | GTTTTTTTTT |
| 10 | ATTTGGCATTTGCGCTTATGATG |
| 10 | TCTCTTTCTC |
| 10 | ACTCCTAACCTA |
| 10 | GGGGAGGGGGGG |
| 10 | AGGTGAATCTCAAGAACC |
| 10 | GCGCAAATGCCAAACCATCATAA |
| 9 | TAAAAAAAAAA |
| 9 | AAACCGCTCAA |
| 9 | TGTGTC |
| 9 | TAAAAAAAAAAA |
| 8 | AGTGCCCAGC |
| 8 | CGTTTTATACGAATAAAACGC |
| 8 | GAACAAAGCGTAGCATCTCAGCCTATGGCTTA |
| 8 | CCACAGAC |
| **Bembidion orion (#SRR2939024)** | | | |
| 1022 | AAT | 27 | TAG |
| 583 | AATCGCGCTCGCCATCCACTGTACGCAAAGCAGA | 26 | CGCGATTTCTGCCTTTCGTACAGTGGATGGCGAG |
| 472 | AACCT | 26 | GAGCGCGATTTCTGCCTTGCATACAGTGGATGGC |
| 391 | CAA | 25 | AAAAAAAATA |
| 367 | CGCAAGGCAGAAATCGCGCTCGCCATCCACTGTA | 24 | TATTACTGTATGAGCTGTA |
| 340 | GTATC | 24 | CAGTGGATGGCGAACGCGATTTCTGCCTTGTGTA |
| 277 | AAAT | 24 | TTGTA |
| 227 | TGAAACAGTAATACACAGA | 23 | AAAAAAGAAAA |
| 195 | TACAACG | 23 | GAACAGCTATACCTGTTCGAGA |
| 183 | TATG | 23 | CAGCGCAGAAAGCGTGTTTTA |
| 147 | AATCGCGCTCGCCATCCACTGTACGCAACGCAGA | 23 | CAACAGCAA |
| 119 | TTTTTTCTTT | 21 | TAAAGTA |
| 118 | TATTTTCA | 21 | GTAAC |
| 117 | AGC | 21 | AAATCTTGTGATATCAATAGTT |
| 116 | GAGC | 20 | AAGCAGAAATCGCGCTCGCCATCCACGGTACGCA |
| 109 | GCATACT | 20 | CGCTCACCATCCACTGTACGCAAAGCAGAAATCG |
| 108 | CAAAGCAGAAATCGCGGTCGCCATCCACTGTACG | 19 | TCGTAT |
| 107 | AAAC | 17 | CCAA |
| 107 | TTTTTAGCTG | 17 | AACATC |
| 106 | GGCAGAAATCGCGCTCGCCATCCACTGTACACAA | 16 | TGTC |
| 99 | TTTCTGCTTTGTGTACAGTGGATGGCGAGCGCGA | 16 | GTGGATGGTGAACGCGATTTCTGCCTTGTGTACA |
| 93 | TCT | 16 | GCAGAAATCGCGCTCGCCGTCCACTGTACGCAAA |
| 91 | TTTCTTAGCTGTTTTTAGCTG | 15 | AAAAAACGCTTTCTGCGCTGT |
| 88 | CGTT | 14 | AAAGTACGACTA |
| 86 | GCTGTTTCTTA | 14 | CGCAAAGCAGAAATCGCGCTCACCGTCCACTGTA |
| 81 | GGCTATTC | 13 | CAGTGGATGGCGACCGCGATTTTTGCCTTGCGTA |
| 72 | GCTAAGGAAGGAGT | 13 | ATCGCGCTCGCCATCCACTGTAAACAAGGCAGAA |
| 65 | ATTTTTA | 13 | AAAAATA |
| 63 | ATGGCGACCGCGATTTCTGCCTTGCGTACAGTGG | 13 | TTAAATAAATTATA |
| 58 | ATTGTT | 13 | ATCAGC |
| 56 | TCAA | 13 | GTTGTTG |
| 56 | AACTGCTTACTG | 13 | GCTTTT |
| 54 | CCTTTAA | 13 | GTGGATGGCGACCGCGATTTCAGCTTTGCGTACA |
| 52 | GTACAGTGGATGGCGAACGCGATTTCTGCTTTGC | 12 | ATCGCGCTCGCCATCCACTGTACGAAAAGCAGAA |
| 48 | CCTAAC | 12 | ACACCA |
| 48 | CAGCTCAGAAACAGCTAAAAA | 12 | ATATT |
| 48 | CAT | 12 | AGCGTGTTTACAGCGCAAGAA |
| 47 | TTTCTGCCTTGTGTACAGTGGATGGTGAGCGCGA | 12 | AAGAAACAC |
| 46 | TTTTTA | 12 | AAAAATTTTTTTAAA |
| 41 | AAACTA | 12 | ACTTCT |
| 40 | AACAGC | 12 | CAAAGCAGAAATCGCGCTCGCCATCCACTGTACC |
| 39 | TATTT | 12 | TTTGT |
| 39 | TCG | 11 | AATATATATAT |
| 39 | CAGTGGACGGCGAACGCGATTTCTGCCTTGCGTA | 11 | GGCAGAAATCGCGCTCGCCGTCCACGATACGCAA |
| 38 | TGGCGACCGCGATTTTTGCCTTGCGTACCGTGGA | 11 | GCAGAAAGCGTTTTTTCAAA |
| 37 | TTTCTGCCTTGCGTACAGTGGATGGCGAACGCGA | 11 | TCTCGT |
| 37 | TCCACTGTACACAAAGCTGAAATCGCGGTCGCCA | 11 | CTGTACGCAAGGCAGAAATCGCGCTCACCATCCA |
| 36 | ACTGCTTACTAA | 11 | ACTAAAACTAAA |
| 36 | GTGGACGGCGAGCGCGATTTCTGCCTTGCGTACA | 11 | CGATG |
| 35 | ACGAA | 11 | TCTCTATCTCTGTC |
| 35 | GACCAAG | 11 | GCTATT |
| 33 | GGATATTT | 11 | GTTCT |
| 32 | TAGACCTGA | 11 | TTGTAT |
| 31 | CGATATCTGCCTTGCGTACAGTGGATGGCGAGCG | 11 | ATTTTTGCCTTGCGTACCGTGGATGGCGAACGCG |
| 30 | TCCACTGTACGCAAAGCAGAAATCGCGTTCGCCG | 10 | TGGATG |
| 30 | TTTTTTTA | 10 | ATAAAAAAA |
| 30 | ATTTTTTTTTT | 10 | AATTGTCC |
| 28 | CTTTCTGCTCTGAAAAAACG | 10 | TTAATA |
| 27 | TTAAA | 10 | GTTTTAATTTTA |
| 10 | CAAGGCAGAAATCGCGCTCACCATCCACGGTACA |
| 10 | GTGTT |
| 10 | TTTATCGACGT |
| 10 | ACAGAAT |
| 10 | TGGATGGCGACCGCGATTTCTGCCTTGTGTACAG |
| 10 | AACTAC |

| **Bembidion castor (#SRR5230398)** | | | |
| --- | --- | --- | --- |
| 1886 | **AACCT** | 123 | AGGGGGGG |
| 1157 | TTG | 121 | TTTTTTTTTC |
| 1123 | GGAAGATAAA | 120 | GAAGATGAA |
| 813 | TTA | 120 | TTAGGTTTGGGT |
| 542 | GGACGTTTCATCCGC | 116 | AATTGGGATGAAATTTTGGAAA |
| 517 | CCTTCATCTT | 115 | AAAAACAAAAA |
| 511 | GAAGATAAA | 115 | ATTAGT |
| 493 | TAAA | 107 | GTTGAAT |
| 453 | GTC | 107 | ATGCTGTG |
| 398 | CGGTACTTTTCGCGCCCGCTTC | 105 | ATAATGT |
| 395 | ACT | 105 | TTTTTA |
| 342 | AAAAAAAATA | 104 | GGGGAGGGGGGG |
| 323 | ATATAAA | 103 | GTTTT |
| 309 | ATACTTT | 102 | CCAACAT |
| 288 | TTTTATTTT | 102 | TTTTGC |
| 269 | AATAAATAATACAAT | 101 | AAATA |
| 258 | CCAGCAT | 101 | GGGGCGGGGGG |
| 258 | AATACTGATACTCAGTCTTGGCCC | 101 | TTCAGTAT |
| 258 | ATGT | 99 | CCAAGGCTGAGTATCAGTATTGCG |
| 246 | AAAATAC | 98 | TTTTTTTGT |
| 238 | ATTTAAGATATCTTAAATTGA | 96 | AAATACG |
| 238 | GAT | 96 | ATTGTAT |
| 232 | ACCAAAACCAA | 95 | TCATTAATTAA |
| 231 | GAAGATAAG | 94 | AACCTAACGTGAGAT |
| 230 | GAGGGGGGGG | 93 | GTAATT |
| 222 | AAAATGT | 92 | TGC |
| 222 | AAAATAAA | 85 | GGATGAAACGTCCGCT |
| 221 | TTTTTATTTTT | 84 | GGACGTTTCATCCC |
| 205 | AAGTAACGT | 84 | ATATTCT |
| 205 | GGAGGGGGG | 84 | TTTTTTTG |
| 202 | TTACAAT | 83 | AAAAATAAAAAAA |
| 192 | TATTCTG | 83 | AAATACT |
| 191 | AGGGGGGGGGG | 83 | AGAAGT |
| 191 | GGATGAATCGTCCGC | 82 | CTCTCTTT |
| 190 | ATTTTTT | 82 | ACGTTC |
| 178 | AAGCGGGCCGAAAAGTACCGG | 79 | TATGTTG |
| 176 | GGCACAATACTGATACTCAGTCTT | 79 | GCGA |
| 175 | CTACATT | 78 | CTAAACTTAG |
| 168 | TATTG | 78 | CATAT |
| 164 | CTAACTATT | 77 | ATTCTG |
| 163 | CATTAA | 76 | AGCATCCAC |
| 162 | AAACAGAACATGACA | 75 | ACACTACTACTACACT |
| 162 | TTATAGT | 75 | GCAACA |
| 154 | TTTTTTACCTGCTTTTGCGCA | 74 | AATACG |
| 150 | TTTTGTTTTT | 74 | TTTTTTCTTTT |
| 150 | TTCTAGA | 73 | CAAAACCTTGTCAAATTCTCG |
| 147 | AACCGAAACCA | 73 | CAATCCA |
| 142 | AAAACGT | 72 | AATTTTAC |
| 142 | TATAGAT | 72 | TTCAGTA |
| 140 | TAAATTA | 71 | ACGTGGTTCGTCAATGTTCCGGAAAC |
| 139 | TTGTAT | 71 | AAG |
| 138 | AGAACAGTGACAAAAC | 71 | TTGTAGA |
| 138 | TTTGG | 71 | TGTTTT |
| 138 | AAAAAAAATAAA | 70 | TATGTG |
| 137 | CTATTA | 70 | CTCCTTTTGCGCATTTTTTAC |
| 135 | CAAGGCTGAGTATCAGTATTGTGC | 68 | TGTATAC |
| 129 | TGGTTT | 68 | TTAC |
| 129 | AACATC | 67 | TGTTAC |
| 129 | ACGTA | 66 | GTTGTTG |
| 64 | CTTGAAT | 46 | GGGGGA |
| 64 | GATGTTCA | 46 | AATTTCAGATAACCTAACTT |
| 64 | ATCTTCCTC | 46 | AAAAAAAAATT |
| 64 | ACGAA | 46 | AAAAAGAA |
| 64 | ACGTAA | 46 | TTTATGT |
| 63 | ATTTTTAT | 45 | AGCGCAGCGAATCGAGTCGAAACGA |
| 63 | GAAAACA | 45 | AATGTTTC |
| 63 | AACG | 45 | TAATAT |
| 62 | ATGTGA | 44 | AGTTTATT |
| 62 | AGCAAT | 44 | AACATTTT |
| 61 | GGATGAATCGTCCGCT | 43 | TTCATA |
| 61 | GGTAACG | 43 | AATTGTAG |
| 60 | TTTTTTTTTTTG | 42 | ACTACTTTCATGCACTACTACTACACTAA |
| 60 | GGGAGGG | 42 | GAAGATAAAA |
| 60 | CATCTTA | 42 | ATGACAAGGTTTTAGAACAT |
| 59 | AAAAGAAAA | 42 | CTGCATCATCACCGTCG |
| 59 | AATACTAACTT | 41 | CTTCAA |
| 58 | CCTTA | 41 | AAAACCTAACT |
| 58 | CAGTTTT | 41 | AAAAAAAAAAAATAA |
| 58 | TTTTTATTTTTTTT | 41 | TAGGCGT |
| 58 | ACGACA | 41 | CTCACAAATACATCTCGTGAGGTCTG |
| 57 | GAAGCACAAAC | 39 | AACTGTA |
| 56 | AAATGCG | 39 | TTTCATG |
| 56 | ACGTTAT | 39 | GGGGGAGGGGGGGG |
| 56 | AATCCTG | 38 | TTCGTT |
| 56 | TAGTATA | 38 | CGTTAA |
| 56 | AACGAGA | 38 | ATTTTTGCT |
| 56 | TATTACAA | 38 | TACTTGGA |
| 55 | TATCGAAAG | 38 | CGACTAC |
| 55 | ATCGTGA | 38 | TGTAGGA |
| 54 | TGTGGA | 38 | CGAAT |
| 54 | GTCGCTGCACCGAAAC | 37 | ACATCCCA |
| 54 | TTTTTTG | 37 | TTAAAAAAAAAA |
| 54 | GAACACT | 37 | ATGTTCAA |
| 53 | TTTGTACTGTA | 37 | TTAAAAAAAAAAA |
| 53 | ATTCTTG | 37 | AATTTTTTTT |
| 53 | GTGAGGGCAATCCAGTGAGTCGGATT | 37 | TTCTA |
| 53 | TACAAG | 36 | TTTG |
| 52 | GGTTTTGGTTA | 36 | AAATAAT |
| 52 | ACATATA | 36 | CGAATAA |
| 52 | CATGTT | 36 | AGGGGGGGGA |
| 51 | CTTCTACTTTAT | 36 | CAGTATC |
| 51 | ATGTAA | 35 | AGAACAGTGACTAAAC |
| 50 | AGCAACAAC | 35 | TTTGCAG |
| 50 | TTTCTCTCTC | 35 | AAGA |
| 50 | ACCA | 35 | CTGAGAA |
| 50 | GGAGGGGGGGGGG | 35 | TACTAAAA |
| 49 | TATTTAC | 35 | GGATGAACCGTCCGC |
| 49 | CACAAG | 35 | GTGGTGATTCGACGGC |
| 49 | CGTTGC | 35 | TTTCTC |
| 49 | GTTATT | 35 | TTTTTACG |
| 49 | GATGCTGG | 34 | TTACTTACT |
| 48 | TACCACGCCA | 34 | TAATTTG |
| 48 | GAATACG | 34 | ATCGTA |
| 48 | TTCACAA | 34 | CAGAATC |
| 47 | AAGC | 33 | ATATTCTCTTT |
| 47 | TTGATA | 33 | AACAC |
| 47 | GTCCCTTATATCGAG | 33 | GTG |
| 46 | CAAACTAAAAC | 33 | AAAATTTCATCCTAATTTTTCC |
| 46 | AAAGT | 33 | TGCTTC |
| 32 | ATACGAAT | 26 | TACAAACG |
| 32 | AATACTGATACTCAGGCTTGGCAC | 26 | AACATCTC |
| 32 | TGAAAACA | 26 | ATGACT |
| 32 | TGAACGT | 26 | ATAAAT |
| 32 | GCAATTC | 26 | ACATATCC |
| 32 | CCCAAA | 26 | GAACGTA |
| 31 | TTTTC | 26 | TTTAATG |
| 31 | TGAGTTTTTTTT | 26 | AATTTAAGATATCTGAAATTC |
| 31 | TTTTGCA | 26 | AGTTTC |
| 31 | TTTCAGTG | 26 | CATGGAA |
| 31 | ATTTTTAC | 26 | GTAAATG |
| 31 | AAATTGAATTTAAGATATCTC | 26 | AAGAAGATAAAGTAGAAGATG |
| 31 | ACAAGAT | 26 | ACTTAAA |
| 31 | TCTC | 26 | GTTTTGT |
| 31 | AACAT | 26 | AAAACGA |
| 31 | ATACAT | 26 | CTTGAATA |
| 31 | AGGTTT | 25 | TATTTCTCGCAG |
| 31 | TTCTTGG | 25 | CTTTATCTTCCTCATCTTCTA |
| 31 | TAGTGT | 25 | AACCAAGAG |
| 31 | GCTTT | 25 | ATCAGTATTGGGCCAAGGCTGAGT |
| 31 | TGGCGCAATACTGTAACTCAGCCT | 25 | TTCATTCG |
| 30 | GGGGGCGGGG | 25 | AAATATAAAAA |
| 30 | CATACAG | 25 | AAGCTTG |
| 30 | CTTTATCTTCTTTATCTTCTA | 25 | TTTTCA |
| 30 | GTGAAA | 25 | TTCTG |
| 30 | AAACAGAACATGATA | 24 | GTTTGGTTTCA |
| 29 | AGTGAAAAAC | 24 | ATAAATAAAAA |
| 29 | AACTATTCTA | 24 | CTTCTTCAATTTCAA |
| 29 | TTGTTC | 24 | TATAG |
| 29 | TGTCTTCCCTTGAATGTTC | 24 | CAGTATTTT |
| 29 | CTTTTTTTTTTT | 24 | AGCATTC |
| 29 | ATTACAT | 24 | AGATATCTTAAATTGAATTCG |
| 29 | CTTCCTTTATCTTCCTTCAT | 24 | GATATTCA |
| 29 | TGTACAA | 24 | AATTTAAGATATCTCAAATTC |
| 29 | AAATAACG | 24 | GGAGAGGGGG |
| 29 | ATATTCAC | 24 | TATTACGA |
| 29 | TTGGCACAATACTGTAACTCAGCC | 24 | TTCATAAA |
| 28 | TATATTTTATTCTT | 24 | AACTATAGTTT |
| 28 | ATTCTGA | 23 | GAAACGC |
| 28 | ACTTTTAT | 23 | GGGGGGGGGCGG |
| 28 | ATTTTCGT | 23 | ATAG |
| 28 | ACTGTA | 23 | AAATTGAATTTAAGATATCTG |
| 28 | TATGTT | 23 | AGATGATAACGAATT |
| 28 | GATTCTAATTA | 23 | CTGTGA |
| 28 | TTCGACTCGATTCGCTGCGCTTCGA | 23 | AGCATCA |
| 28 | TTTATTC | 23 | TTAAATA |
| 28 | GAAAAAA | 23 | AAAAAAAAAACAA |
| 28 | AAATAAATA | 23 | ATTCGC |
| 28 | GTGATG | 23 | ACGTCTTTATTTAAATAT |
| 28 | CGTTGATGATTTTTGCAAAT | 23 | TATAATA |
| 28 | AATTAGAATCC | 23 | AAATTTTCCAAAATTTCATCCC |
| 28 | TGAATAT | 23 | AAAAATTGGTCAAGTTAAATTTTG |
| 27 | GCGGATTGC | 23 | TAGATTAGAA |
| 27 | GTCGTA | 23 | TTTTTTTTTATA |
| 27 | TACATTTA | 22 | AGCACAAAATT |
| 26 | TGTGTGTATG | 22 | TTTCTAG |
| 26 | AACAAACC | 22 | AAAAAATTAAAAAA |
| 26 | CGTCGATTATCCGAA | 22 | AATATAGAAT |
| 26 | ATTTTTGACC | 22 | AACGTAC |
| 26 | TGAAATCC | 22 | GTTGTA |
| 21 | TACTTAA | 14 | TCTCAAT |
| 21 | CAAAACAGAACAATGA | 14 | GTTAAAC |
| 21 | CAAAGATT | 14 | TCTCGCA |
| 21 | ACAATCCGC | 14 | ATTTCAC |
| 21 | GTTACACA | 14 | AACGAAAG |
| 21 | TTTTTTTTTTTTTTTA | 14 | AAGCGGGCGCGAAAACTACCGG |
| 21 | ATACATCAT | 14 | TAACAAA |
| 21 | CCTCGATATAACGGA | 14 | TTTCTT |
| 21 | TTATCT | 14 | TCGTTTCA |
| 21 | CAAAATAA | 14 | TCTTCAATTTCAACC |
| 21 | TTTTTTTTTGG | 14 | GGGGGGGGGAAGGG |
| 20 | GAAATAA | 14 | TGAAATA |
| 20 | TACGATGATGTT | 14 | AAATACAA |
| 20 | GTCCGCTGGATGAAACGTCCGCGGATGAAAC | 14 | AGTTC |
| 20 | TCTG | 14 | GGGGGGGAGAG |
| 20 | CGGAAGCGGGCGAAAAAGTAC | 14 | TGGATGCT |
| 20 | GTTTCT | 13 | ATTTTTTTTTTTTTTTT |
| 19 | AAGATGAAGAAGATAAAGG | 13 | CGTATTA |
| 19 | GAATTAAATGATACATTGTTA | 13 | CTTAG |
| 19 | AATAGAA | 13 | TTCGTTC |
| 19 | AAAACTA | 13 | AAAAGAGT |
| 18 | AAGAAT | 13 | CGATCTTA |
| 18 | TGTG | 13 | ATACTTTA |
| 18 | CCACGCCGTCGAATCC | 13 | TAGTGTAGTAGTAGTT |
| 18 | TTGAAATAACGTT | 13 | TGTACAGAGCAGAATGTGCTGTG |
| 18 | TTCATCCGCGGACTA | 13 | AAACGTTG |
| 18 | TATAATATACAAATA | 13 | TTCAATAT |
| 18 | AATTTTTTT | 13 | CCAAAACATTAAAAGAAGAGCTTCTAGAAAGTT |
| 18 | GCAATGCAAGAC | 13 | AGCAAACA |
| 18 | GGGGGGGGGAA | 13 | AAATTCT |
| 17 | TGGGATA | 13 | ACAAACT |
| 17 | TTCTAACAATGCATCATTTAA | 13 | ATTGAGG |
| 17 | TTTATTTATA | 13 | ATAGAAGT |
| 16 | GATGCAA | 12 | TATTTTATG |
| 16 | GTATAA | 12 | ATCACG |
| 16 | AATTAAATGATACAATGTCAG | 12 | CGGGGGGGG |
| 16 | TTCAGTAA | 12 | ACTGGAAA |
| 16 | GTTGTTC | 12 | AAAATC |
| 16 | TACATGTACA | 12 | TTTATTCG |
| 16 | GCTTCA | 12 | GTGGGATTCGACGGC |
| 16 | GTCGTTGCACCGAAAC | 12 | TTTACCGT |
| 16 | AACACAC | 12 | GGGGCGGG |
| 15 | AGTAACC | 12 | ATTATTTT |
| 15 | ATTGGGCCAAGACTGAGTATCAGC | 12 | AACTCGC |
| 15 | GGGGGGGGGT | 12 | ATTAATAA |
| 15 | CGGGGGGGGGGA | 12 | CATTCTATATT |
| 15 | CTTGCATTGCT | 12 | TGCGAGGC |
| 15 | GAGAGGCATAAAACC | 12 | AAATCGTG |
| 15 | GGGGGGGGGGGGGGA | 12 | AACGAAAC |
| 15 | AGTTGTA | 12 | AAGCGGGCGCGAAAAGTACAGG |
| 15 | TAAAAATGCGCAAAAGCAGG | 12 | AATTG |
| 15 | AGGAAGATAAAGAAGATAAAGTAGAAGATG | 12 | GGGGGGAAGGGG |
| 14 | TTGAG | 12 | ACGAGCA |
| 14 | ATTCAAAT | 12 | TATATATT |
| 14 | CGTTTTCA | 12 | AATCAAC |
| 14 | AAGAGCAGGTAAAAAATGCGC | 12 | AACGTGT |
| 14 | TGTTTGCTGGGTTGCT | 12 | GAAATGA |
| 14 | AAAAAAAAAAAAG | 12 | GACTGAGTATCAGTATTGCGCCAA |
| 14 | CACGATC | 11 | CATTTTCA |
| 14 | ATTGTATTT | 11 | CATCCAAA |
| 11 | GAAAAAGAA | 10 | AAATTTC |
| 11 | ATCAACG | 10 | GTACATC |
| 11 | GAATATACC | 10 | AACTGAGA |
| 11 | TGTGTAA | 10 | ATTGGGCCAAGACTGAATATCAGT |
| 11 | CAACAGCAG | 10 | CAAACATT |
| 11 | TTTTTATATT | 10 | CGCGAATAATCGAATAAC |
| 11 | GAAAGAATGGAAATGGGTGAA | 10 | TGTATTTC |
| 11 | AAAAAAAAAACAAA | 10 | AACTCGA |
| 11 | ACTGTGCCAAGACTGAGTATCAGT | 10 | GATGC |
| 11 | TCTGATAT | 10 | CATCTTCCTTTATCTTCCT |
| 11 | AAATTCTG | 10 | TTGTGA |
| 11 | GGAAACT | 10 | GCAAAC |
| 11 | ACCAGTT | 10 | AATTTTAT |
| 11 | GTGTTCCA | 10 | TAAACGG |
| 11 | CATTAT | 10 | TGTTCAA |
| 11 | ATCACGTCGGAT | 10 | GGGGGAAGG |
| 11 | AATCGAC | 10 | GGGGGGGGGTG |
| 11 | TGAGTTTTTTTTT | 10 | ATGA |
| 11 | TTCGTC | 10 | ATTACC |
| 11 | TCCTACTACTCC | 10 | ACGTATTT |
| 11 | AAAAATGGGATAGGGG | 10 | ACGTTTCC |
| 11 | TTTTACTG | 10 | CCAACATAT |
| 11 | CCAAAGAA | 10 | TATTATG |
| 11 | AGCATCATACCCGATCCAGGCGT | 10 | AAAATATT |
| 11 | GTAAAAA | 10 | AACCTAACCCT |
| 11 | ATATGTAT | 10 | GGATGAAATTTTGGAAAAATA |
| 11 | TGTTTGC | 10 | GGAGAGGGGGGGG |
| 11 | ACATTAAA | 10 | ACATATATACACAT |
| 11 | CTGTTCAAAAATACTAACTCT | 10 | TGCTAC |
| 11 | GTAACGCT | 10 | TACTTTTCGCCCGCTTCCGG |
| 11 | ATCAACC | 10 | GAGGAGGGGGGGGG |
| 11 | CTAACAG | 10 | GTATGTA |
| 11 | ACAATTCA | 10 | TTGTTGC |
| 11 | GTATTAAGTCA |
| 10 | GAATACTG |
| 10 | TCTCGAT |
| 10 | TTCTTGGA |
| 10 | TCCAGTTC |
| 10 | TTCGAGA |
| 10 | CAATTG |
| 10 | AACAGCTAAAGACAGCTCCGA |
| 10 | ATCA |
| 10 | AAATGCT |
| 10 | AAAATTAC |
| 10 | AGCATCTC |
| 10 | ACTGGTAA |
| 10 | TTTACCAT |
| 10 | TTTTCAGG |
| 10 | TAGATTT |
| 10 | GTGTGTA |
| 10 | TTAAATAT |
| 10 | GGGGGGGGGGGGGAGG |
| 10 | GGGGGTGGG |
| 10 | AAATTTAC |
| 10 | CTCTGCTTGTACCTCTGCTGT |
| 10 | TACAACGT |
| 10 | AAAATAAAAAAAAAAAAA |
| 10 | CAATACTGATACCCAGTCTTGGCC |
| 10 | CGGACGATTCATCC |
| 10 | GGGATGAAATTTTGAAAAAATT |
| **Bembidion flohri (#SRR5230420)** | | | |
| 7107 | TTTA | 112 | AACCCCAA |
| 4639 | AAG | 111 | TTGTCATGTCCGAGTTT |
| 4596 | TAA | 109 | ACAGAAT |
| 3276 | TCAAATAAA | 108 | GTTTTT |
| 2700 | AAAAAACC | 106 | CTATAAT |
| 2435 | AAATAAT | 104 | TTTTC |
| 2404 | ATGGA | 102 | AGATCGGAAGAGCACACGTCTGAACTCCAGTC |
| 2387 | **GGTTA** | 100 | TTTATGT |
| 1846 | CAGATCGGAAGAGCACACGTCTGAACTC | 99 | AATACATA |
| 1288 | TTATTTTAT | 96 | CACT |
| 1117 | TGT | 96 | AGATCGGAAGAGCACACGTCTGAACTCCT |
| 929 | TTATTT | 95 | GATTTATTC |
| 806 | ATATTTT | 93 | CACTC |
| 792 | ATATGTAC | 91 | ACGTAAA |
| 705 | TTCATTTTA | 91 | TGC |
| 644 | AGATCGGAAGAGCACACGTCTGAACTCCC | 90 | TACATA |
| 611 | GATGGCATCATATAGTGCGACTATGTTTT | 88 | GATT |
| 566 | AGTT | 88 | TATTG |
| 519 | TAC | 85 | GAACAACAAGAT |
| 503 | ATGT | 84 | TTCCATTCGA |
| 383 | TTCT | 84 | TTATAGT |
| 336 | ATAAATT | 83 | TTTTTTTA |
| 330 | GAAAAA | 83 | AGATCGGAAGAGCACACGTCTGAACTC |
| 295 | TATAATA | 83 | TAATAG |
| 282 | ATAAAT | 81 | AAGTG |
| 272 | ATAAA | 79 | TGATTCCATTCGATTCCATTCGA |
| 263 | TATATATT | 76 | AGATCGGAAGAGCACACGTCTGAACTCCAC |
| 261 | TATC | 75 | CTATATT |
| 259 | TTTGTAT | 75 | TTACAATA |
| 252 | ATTAAT | 75 | ATTTTTTTTTT |
| 229 | TTTTTTTTGTA | 74 | AGAGGA |
| 217 | GGTTTGGTTTT | 74 | AAAACTGA |
| 216 | ACATTTT | 74 | TTTTAA |
| 214 | TGA | 73 | AAAAACA |
| 199 | AATAAAA | 72 | GGT |
| 197 | TAAATTAAATATAT | 72 | ATAATTA |
| 189 | ATATA | 72 | AAAAAAAAT |
| 183 | TCC | 72 | TTATTTTATTAT |
| 183 | AACA | 71 | AGATCGGAAGAGCACACGTCTGAACTCCTC |
| 182 | ATTGTAA | 71 | CTCT |
| 178 | TTTGT | 70 | TTGTAA |
| 173 | TTCTTC | 69 | GGGTTAGGTT |
| 170 | ATTAATTTATAAAT | 69 | AACATT |
| 165 | GGAA | 67 | TTTACAAT |
| 148 | TGTC | 67 | GTATATT |
| 148 | TACAAAAAAAAA | 67 | TTGAATG |
| 141 | AATACAATAATAAAT | 67 | AACAAAAATAAAAATTTA |
| 138 | CTCCTT | 66 | TTTGG |
| 137 | ATTTACTTG | 66 | CATTATA |
| 130 | AGTGACAAAACAGAAC | 65 | AAACATG |
| 130 | TTTATAC | 65 | AAATAC |
| 129 | TTTATTCA | 64 | TCATCATCAAATGGAATCGAATGGAA |
| 128 | AGATCGGAAGAGCACACGTCTGAACTCCAGTCC | 64 | TCTTCTTA |
| 128 | AAAGAAGACGAAAACTAAGAAG | 64 | TTCTTCTTAACTGGC |
| 117 | TTTTTTTATT | 62 | AGATCGGAAGAGCACACGTCTGAACTCCCC |
| 117 | TAAAAATTTTTAATTATTTTTA | 59 | TATTTATTTGTTGA |
| 117 | TTAATAT | 59 | TTTTTAG |
| 116 | GGAT | 59 | TGTTCTGTTTTGTCA |
| 115 | CCTAAC | 58 | TATTTAC |
| 57 | AAGTAAA | 36 | CAGATCGGAAGAGCACACGTCTC |
| 56 | AGAGCACACGTCTGACTCCAGATCGGA | 36 | TTCGTAA |
| 56 | GTATTA | 35 | AACAGC |
| 54 | TTTTTCT | 35 | AGATCGGAAGAGCACACGTCTGAACTCCTCC |
| 54 | TATATAG | 34 | TTGCTC |
| 53 | TCCATTCCATTCGAT | 34 | AAAAGAAAAA |
| 53 | CAATCCC | 34 | AAAGAT |
| 53 | TTTTTTTC | 34 | GAAAC |
| 52 | TACAAC | 34 | ATACC |
| 51 | TTATTATT | 34 | TTTGTATTTATATC |
| 49 | AACCAA | 33 | GAAAAAAAA |
| 49 | TTAAAAT | 33 | TCTTCTTCTCCT |
| 47 | ATCATCGAATGGAATCGAATGGAATC | 33 | TAAAG |
| 47 | AATTA | 33 | ATTTTTGCT |
| 47 | GCATACGTCA | 32 | TTGTTATT |
| 47 | TTAAAATG | 32 | TGTTCGTTATCCGATC |
| 47 | TCAGTTT | 32 | AGATCGGAAGAGCACACGTCTGAACTCCATCCC |
| 47 | TCTACAT | 31 | TATATATAA |
| 46 | ACATTT | 31 | TTTCAATA |
| 46 | AATCCTG | 31 | CATCA |
| 45 | CTAAATCAAC | 31 | AACC |
| 45 | AAACTA | 31 | TTTTATTAAGTTCTTCACGTATTT |
| 45 | ACG | 31 | TTTTTTTTTTTA |
| 45 | AAAGAAAAT | 31 | AGATCGGAAGAGCACACGTCTGAACTCCAGTCCC |
| 45 | AATAAG | 31 | AGATCGGAAGAGCACACGTCTGAACTCCATCC |
| 45 | TTCTGTTTAGTCACTG | 31 | TTTTGTG |
| 44 | TCTAGAT | 30 | CATTTTG |
| 44 | AATG | 30 | TTTCTTTCTCTT |
| 44 | AAATACG | 30 | TAGGGTT |
| 44 | TTGTATG | 30 | TCTCTTTCG |
| 44 | TACACA | 30 | AGATCGGAAGAGCACACGTCTGAACCC |
| 43 | TTTGTATATTATATC | 30 | CGTTTA |
| 43 | TTGCTT | 29 | AATTCAACCTTAAACTACCCT |
| 42 | ATTCCATTCGATG | 29 | ATCGGAAGAGCACACGTCTGAACTCCACCAG |
| 42 | TATTTCT | 29 | TTCTATCTTC |
| 42 | GATGTT | 29 | TATATCTTC |
| 42 | AGATCGGAAGAGCACACGTCTGATCTCC | 29 | AGAGAGAA |
| 41 | TATTTAG | 29 | TGTGAA |
| 41 | TTTTAC | 29 | TATTCA |
| 41 | AAACAGA | 28 | TGAATATT |
| 40 | ATTTGTA | 28 | CCTTA |
| 40 | GAAAAT | 28 | AATATTTT |
| 39 | AGATCGGAAGAGCACACGTCTGAACCCC | 28 | CCCAGATCGGAAGAGCACACGTCTGAT |
| 39 | TTTTCTA | 28 | TCATTCA |
| 39 | AAAATTAC | 28 | ACTGTT |
| 39 | TTAC | 28 | GAAATAT |
| 39 | TCTCCTTCT | 28 | TCTTCTTCC |
| 39 | TTTTAGTGAAGTG | 28 | TTAGTTTTAAGTCCGCG |
| 39 | ACAATTC | 27 | CTTCCTTCTTTGCTTTTTCTTCAG |
| 38 | ATAACA | 27 | ATACTT |
| 38 | TTTTAGTAG | 27 | TCTTCA |
| 37 | TTTTTTGT | 27 | TAACACCC |
| 37 | ACCCTAACCTA | 27 | CATTTTTA |
| 37 | AAAAAGGA | 27 | AAATCACAAATCGC |
| 36 | CAACTT | 27 | ATACCGGT |
| 36 | CAAAACCT | 26 | CACCAT |
| 36 | ATATAAAAT | 26 | TAAACA |
| 36 | GTATTTCA | 26 | TATTTATTTATTTGTTGCA |
| 36 | TAATGCGATAATTAA | 26 | AACAGA |
| 36 | CAGATCGGAAGAGCACACGTCTGACTCC | 25 | ATATCA |
| 25 | AATCTAC | 17 | TATATATATATTATAAATATT |
| 25 | AGATCGGAAGAGCACACGTCTGATCTCCC | 17 | CCCAGATCGGAAGAGCACACGTCTGA |
| 25 | AATTTAG | 17 | AACATAT |
| 25 | CTTGATT | 17 | AAAAAAAAAAAC |
| 25 | TTCAA | 17 | ACACA |
| 25 | ATAATGA | 17 | TTTCTTG |
| 25 | TGAATTTA | 17 | TCAGTATTT |
| 25 | AAAAAACAAA | 16 | CCTCA |
| 25 | TTATAAGCCGCGTTAG | 16 | AGATCGGAAGAGCACACGTCTGAACTCT |
| 24 | AATGA | 16 | TTAAATAT |
| 24 | AATTCTAC | 16 | TTTCTTTTCTTTTCTTCCTC |
| 24 | TTTCTCG | 16 | TTTTCCTCT |
| 24 | CCTAATTCAA | 16 | AAAAAATAATAAAAAAGAA |
| 24 | GTATTC | 16 | AGATCGGAAGAGCACACGTCTC |
| 24 | CCCTAATAA | 16 | CACA |
| 24 | ACTTTTA | 16 | CACAC |
| 24 | TGATTCCATTCGAGTCCATTCGA | 16 | GCATTTC |
| 24 | TCAAAAAATG | 16 | TCCACTCCAT |
| 23 | TTAAAAA | 16 | TGCTAT |
| 23 | TTCTCGCAGTAT | 16 | AGATCGGAAGAGCACACGTCTGAACTCCATC |
| 23 | ATCCAGC | 16 | GTTGGG |
| 22 | AAGAAAAAAT | 15 | GATTT |
| 22 | ATATTTTG | 15 | TTCG |
| 22 | AGATCGGAAGAGCACACGTCTGAACTCTCC | 15 | CTTCTTCTT |
| 22 | AAATACTA | 15 | GCGACTTATAACTAAC |
| 22 | TCTTACA | 15 | TCCTCCTTC |
| 22 | GATTTTCA | 15 | TTATATAATTA |
| 22 | GCAAAC | 15 | TAATACA |
| 22 | AAAGGAAGAAAAAATGGAAG | 15 | CAGATCGGAAGAGCACACGTCTGAC |
| 22 | CTTTC | 15 | ATATAATATAAAAG |
| 22 | TTTTACTTTTAT | 15 | TTCTTCTTCCTC |
| 22 | TTTTAATTTCA | 15 | TTTATTTTTATTT |
| 21 | ATTA | 15 | TTTTCG |
| 21 | TCATGTTT | 15 | CTGA |
| 21 | AAAACTAAAAAGAAAGAAGACG | 14 | ATATATAC |
| 21 | TTAGTTATAAGTTTCA | 14 | TACTTC |
| 21 | TTTCTTC | 14 | GATTTCT |
| 21 | TCTTGT | 14 | ACACT |
| 20 | TATTATCACATTCTA | 14 | TTTCCTTGA |
| 20 | CTTG | 14 | TAAAAATTTATTATATATTA |
| 20 | AAAAAAAAAGA | 14 | ATAAATTAATTTTAATTAAT |
| 20 | TGTTTTCTA | 14 | TATATATATG |
| 19 | CTAC | 14 | CAATTTT |
| 19 | TAATTTATTTTATG | 14 | ATAATT |
| 19 | TCTTC | 14 | GGAG |
| 19 | AGTTTTA | 14 | TTTTTTTTTCA |
| 19 | AAAAAAAAC | 14 | TTATTTATATAAAAAATTTAT |
| 19 | ATTTTTC | 14 | AATCATCAAATGGAATCGAATGG |
| 18 | AAATATTTT | 14 | TTCCTG |
| 18 | ACTATGTGATGCCATCAAAACATAGTCGC | 14 | AGATCGGAAGAGCACACGTCTGAACTCCAGTCAC |
| 18 | ATTCAAT | 14 | CGCCTC |
| 18 | AGATCGGAAGAGCACACGTCTGAACTCCAGTCT | 14 | TCTCTG |
| 18 | TTATGCATTATTAA | 14 | GCTACG |
| 18 | CCAGGAC | 14 | TACAAAAGAAAA |
| 18 | TCCCAGATCGGAAGAGCACACGTC | 14 | TTAAGTTCTTCACGTAATCCTTTA |
| 18 | GATTCCATTCGATGAT | 13 | AGATCGGAAGAGCACACGTCTGAACTCCTCCC |
| 17 | CATTCT | 13 | CAACAA |
| 17 | TTTTTTAAT | 13 | TGTTCTGTTTTATCA |
| 17 | AGATCGGAAGAGCACACGTCTGAACCTCC | 13 | TTTTTATTTTC |
| 17 | TTTTTGTAT | 13 | TATACAG |
| 13 | AAATAAAATACACTAATAAAAA | 11 | CTCCCT |
| 13 | GCCAGATCGGAAGAGCACACGTCT | 11 | GTTCTGTTTCGTCACT |
| 13 | TTTGATTTC | 11 | TTATTAG |
| 13 | ATATCTAA | 11 | TACTG |
| 13 | ATCCT | 11 | TCCAGATCGGAAGAGCACACGTCTGACC |
| 13 | AATAAT | 11 | AATTCAT |
| 13 | CAATGTTT | 11 | AATCCTA |
| 13 | ATTTACAA | 11 | AACAACC |
| 13 | CTCAT | 11 | AGATCGGAAGAGCACACGTCTGAACTCCACTCC |
| 13 | GGAATCGAATGGAATCATTGAAT | 11 | TAAAATAAATAAA |
| 13 | GTAACTT | 11 | TCTCTC |
| 13 | AAACAAAAAAA | 11 | AAAGTT |
| 13 | TTATAAG | 11 | TATTTTATTTT |
| 13 | GAAAATAA | 11 | CATGCAACTATTAATCTAT |
| 13 | CGCACA | 11 | TTTTTACG |
| 13 | CTGTT | 11 | GTGC |
| 13 | GATGCG | 11 | AGATCGGAAGAGCACACGTCTGAACTCCATCTCC |
| 13 | TTCGTAAA | 11 | TCTTGAA |
| 13 | ACATTAA | 11 | ATTCCATTCCACTCC |
| 13 | CCCAGATCGGAAGAGCACACGTCTGAACTCCA | 11 | TGTTTCGT |
| 13 | CTAACTATT | 11 | TTTATTTCTTTC |
| 12 | TTTTTCATTCTTTTATATTCTTTTTTT | 11 | GATATCTGAAATTCAATTTAA |
| 12 | AAATAAATATGCAATAAAT | 11 | CATCTTCTTC |
| 12 | ATAT | 11 | TCGTTTT |
| 12 | TTCACTTCCCAAGACGGTA | 11 | CTCCAGATCGGAAGAGCACACGTCTG |
| 12 | ACACATAC | 11 | GTGTTTCA |
| 12 | AACTGTA | 11 | TTCCATTCGAGTCCA |
| 12 | TTTATTTCA | 11 | ACGTTA |
| 12 | TTTTTTTTTGTAAT | 11 | CCAACAAA |
| 12 | GTACATAAAATATGAAA | 11 | AGTCCATTCCATTCC |
| 12 | TTCGTCTTCTTTCTTCTTAGAT | 11 | AGATCGGAAGAGCACACGTCTGAACTCTC |
| 12 | CTAATATT | 11 | AGATCGGAAGAGCACACGTCTGATCCTCC |
| 12 | GATCGGAAGAGCACACGTCTGATCCCCA | 11 | TCTTTTC |
| 12 | AGATCGGAAGAGCACACGTCTGTCCC | 10 | TAAATTAAAA |
| 12 | GTACAAAT | 10 | TTTTACCA |
| 12 | TTCATTTTTGTTTAGAATTATATGTTTTG | 10 | TTTTTTTAATTATTT |
| 12 | TGATGATTCCATTCGAGTCCATT | 10 | CAACTTA |
| 12 | TATACTTT | 10 | ACGTACAA |
| 12 | AAAATAGAGAAGAGAGAAG | 10 | TTATG |
| 12 | TATTTTCG | 10 | ACCCTAATTCAACCTAAATCA |
| 12 | CTTCTG | 10 | ATTCCATTAGATGATGATTCCATTCG |
| 12 | AATTTCAC | 10 | TTTATCACTGTTCTGT |
| 12 | TAAATACG | 10 | CTCTTA |
| 12 | ACTGAAT | 10 | TTTTGTTTTTG |
| 12 | TTTATTTCAT | 10 | TTTTATATTA |
| 12 | ATATTTGATGTACTTTC | 10 | ACATTCAC |
| 12 | TGTGTG | 10 | TTTTTTAATATA |
| 12 | GATGATTCCATTCGATTCCATTCAT | 10 | TTATCA |
| 12 | ATTATTTTA | 10 | ATTCCATTTGATGATGATTACATTCG |
| 12 | GATCGGAAGAGCACACGTCTGAACTCCAA | 10 | AGAGTAA |
| 12 | CTTTG | 10 | GAAAAACA |
| 12 | CAAACAA | 10 | AAATTATA |
| 12 | ATCTATTTAATTTTTTT | 10 | AACATCC |
| 11 | CGTTATA | 10 | AGATCGGAAGAGCACACGTCTGAACTCCAT |
| 11 | TGTGTCTG | 10 | CATTCGATAATTCCATTCGATTG |
| 11 | GTACGTT | 10 | CTATT |
| 11 | AATTTA | 10 | GATTCTATTCCATTA |
| 11 | GATCGGAAGAGCACACGTCTGAATCTCCA | 10 | TTTCGTTA |
| 11 | ATTTTCTAC | 10 | CTTCTGT |
| 13 | AAATAAAATACACTAATAAAAA | 10 | ATGATT |
| 10 | TTATGTATTCTGTATTTTGTA |
| 10 | AAGACAAAGAGAGA |
| 10 | AATACGCAACAAATAAATA |
| 10 | TTTCTC |

| **Bembidion lapponicum (#SRR2939026)** | | | |
| --- | --- | --- | --- |
| 5505 | GATTACGTAACTT | 70 | GATTGACAACGTTTTACGCT |
| 4384 | GATTACGTAAGC | 69 | AAAACAGCTCA |
| 2391 | TTACGTAATCGA | 67 | TTTTCAATGGTGACGCAATTGACGATG |
| 1966 | GTCACCATTGAAAACATCGTCAATTGCG | 66 | GGCCCGGCTT |
| 1755 | AAAACATCGTCAATTGCGATTG | 66 | GGTCACATTGAAAACATCGTCATTTGC |
| 1135 | ACTTGATTACGTAAGCGATTACGTA | 64 | AATTGACGATGTTTTCAATGGTGACCTT |
| 888 | AAACATCGTCAATTGCAGTCACCATTGA | 61 | AATTCGCAATTGACGATGTTTTC |
| 769 | TTACGTAATCAAA | 60 | GTTTTATTTAAGATTGACAAC |
| 622 | GTAC | 58 | GACGATGTTTTCAATGATGACCGCAATT |
| 618 | TGCAATTGACGATGTTTTCAAT | 58 | CGCAATTGACGATGTTTTCAATGGTAAC |
| 614 | AAACATCGTCAATTGCGGTCAAATTGA | 56 | ACATCGTCATTTGCGTCAAATTGAAA |
| 536 | TAAAACGTTGTCAATCTTTAA | 55 | ACATCGTCAATTGCGATCAAATTGAAA |
| 474 | CGGTCAAATTGAAAACATCGTCATTTG | 54 | GGTCAAATTGAAAACATCGTCAATTGT |
| 418 | GCAATTGACGATGTTTTCAATA | 53 | TTGAAAACATCGTCAATTGCGGTAACA |
| 403 | GTAA | 52 | CAATTGACGATGTTTTCAATGCTGACCA |
| 371 | GTCAATCTTTAATAAAACCTC | 52 | CTGGATTACGTAAGCGATTACGTAA |
| 350 | CCACAATTGACGATGTTTTCAATGGTGA | 52 | GTTTTATAAAGATTGACAAC |
| 306 | CGTAATCCAGTTA | 50 | TTTTCAATGTTGACCACAATTGACGATG |
| 267 | TTGCAGTCAAATTGAAAACATCGTCAT | 50 | GTTTTACGCTGATTGACGAG |
| 256 | CATCGTCAATTGCGGTCACATTGAAAA | 49 | ATTGACGATGTTTTCAATAACA |
| 242 | GCAATTGACGATGTTTTCAATTTGACT | 49 | ATTACGTAAGTG |
| 227 | AATGG | 49 | CGCT |
| 207 | TTTATAAAACGTTGTCAATCT | 43 | CGCAATTGACGATGTTTTCAATGGTGAT |
| 207 | CGCAATTGACGATGTTTTCAATGTTGAC | 43 | GCATGCCTTCGTTTCTACCAATCT |
| 202 | GTTTTCAATGCTGACCGCAATTGACGAT | 43 | ACGTAATTGATT |
| 196 | ACGATGTTTTCAATTGTGACCGCAATTG | 43 | CGTACGTCCGTC |
| 190 | ATTGACGATGTTTTCAATGGTTACCGCA | 42 | GTATGCACGTAC |
| 185 | GCAATTGACGATGTTTTCAATGTTGACT | 41 | CATTGAAAACATCGTCAATTG |
| 177 | TTACGTAATCAG | 40 | AATTGCAGTCACAATTGAAAACATCGTC |
| 174 | GATTACGTAACGT | 40 | TCAATTGCGGCCAAATTGAAAACATCG |
| 163 | AAGCC | 39 | CAGCAA |
| 163 | GACG | 39 | CGCAAATGACGATGTTTTCAAT |
| 152 | CAATTGACGATGTTTTCAATTGTGACCA | 39 | ATTGAAAACATCGTCATTGCGGTCAA |
| 149 | TTGCGTCAAATTGAAAACATCGTCAA | 39 | TGACGATGTTTTCAATGCTGACCGCAAA |
| 148 | GCGATTACGTAATTTGATTACGTAA | 39 | CGATGTTTTCAATAGTGACTGCAATTGA |
| 146 | CCTAA | 38 | TGTTTTCAATACGCAATTGACGA |
| 127 | ACCATTGAAAACATCGTCATTTGCGGTC | 38 | CGGTCACCATTGAAACATCGTCAATTG |
| 123 | GTTTTATTAAACATTGACAAC | 37 | TGATGCTTTCCGCTGCGTCAA |
| 119 | ACGTAATCAAGCTT | 37 | ATTGAAAACATCGTTAATTGCG |
| 118 | TGACCGCAATTGACGATGTTTTTAATGG | 37 | ATTGACAACGTTTTACTCTG |
| 112 | GTTTTACGCCAATTGACGAG | 37 | AAAATGCATGTTTTTGCTC |
| 111 | GATTACGTAAC | 36 | TGAAACGATGTTTTATAACTTCGA |
| 104 | GTTTTTAACGAAAAATGCAT | 36 | TATCTCTCTCTC |
| 102 | ATGTTTTCAATGGTGACCTCAATTGACG | 36 | CAATTGACAATGTTTTCAATGGTGACCG |
| 98 | TGT | 35 | GTTTTATTCAAGATTGACAAC |
| 97 | TTTATAAAACCTCGTCAATCT | 34 | TCAATCTTAAATAAAACCTCG |
| 89 | TAAAACGTCGTCAATCTTTAA | 34 | GAATGGAATGT |
| 89 | GTTTTATTAAATATTGACAAC | 33 | CAATTGACGATGTTTTCAATGG |
| 87 | ACTGCAATTGACGATGTTTTCAATGGTT | 33 | AACATCGTCAATTGCGGTCAATTGAA |
| 86 | AGC | 33 | ATTGACAACGTTTTATTAAAA |
| 81 | GCAATTGACGATGTTTTCAATGTGACT | 33 | ATTGCAGTCAGCATTGAAAACATCGTCA |
| 80 | ACGATGTTTTCAATGTGACCACAATTG | 33 | TTACGTAATCGCTTACGTAATCAG |
| 78 | TTGTGGTCAAATTGAAAACATCGTCAT | 32 | TAATCAAATTATG |
| 78 | CGCAATTGACGATGTTTTTAAT | 32 | CTGTTTTGAG |
| 78 | GTCAATCAGTGTAAAACGTT | 31 | TAAAACGTTGTCAATTTTAA |
| 77 | TGTTTTCAATAGTGACCGCAATTGACGA | 31 | AAACGATTTAATTCACTTTG |
| 71 | ATTGACAACGTTTTACGCCA | 31 | GTTTTACACTGATTGACGAG |
| 70 | TGTACGTA | 31 | TAAAACCTCGTCAATTGGAA |
| 70 | GAATTTTATT | 30 | AAAACATCGTCAATTGCGGTACCATTG |
| 29 | AAAACATCGTCAATTGCGACTG | 15 | AACATCGTCAATTGCGGCAACATTGAA |
| 29 | GTAAGCGATTACGTAACGTGATTAC | 14 | AAATTGAAAACATCGTCAATTGCGGC |
| 28 | TGTTTTCAATACTGCAATTGACGA | 14 | GATTACGTAACTGATTACGTAACTT |
| 28 | CGATGTTTTCAATTTGACCGCAAATAA | 14 | ATTGACGAGGTTTTATTAAA |
| 28 | GATTGACGACGTTTTATAAAA | 14 | CGCAAATGACGATATTTTCAATTTGAC |
| 26 | ATCGTCAATTGCGCAATTGAAAAC | 14 | AAAACATCGTCAATTGCGGCCACCATTG |
| 26 | CAGCAGCAACAG | 14 | CATTGAAAACATCGTCAATTGAAGTCAC |
| 26 | GGTTTTATTAAAGATTGACAA | 14 | ATCGTCAATTGAGATCACCATTGAAAAC |
| 25 | TGAAAACATCGTCAATTGCGAA | 14 | AAACCTCGTCAATCACTATA |
| 25 | CGATGTTTTCAATGCTGGCCGCAATTGA | 14 | CAGGCGAGCACGGGGAAGGGTAGGA |
| 24 | AAACATCGTCAATCACTATA | 14 | CGTCAATTGCCGTCAAATTGAAAACAT |
| 24 | CCTTACAGAGCCATTGGTGGG | 14 | AACATCGTCAATTGCGGTCACCTTTGAA |
| 24 | TGTTTTCAATGGTGACCCAATTGACGA | 13 | GTTTTAGTAAAGATTGACAAC |
| 24 | TTTACGTAAAAACAGTCGTT | 13 | ACGTTTTACGCCAATTGACG |
| 23 | ATTGACGAGGTTTTACTAAAG | 13 | TGTTTTCAATTACGCAATTGACGA |
| 23 | ATGTTTTCAATGGTGACTACAATTGACG | 13 | GTTTTTAGCAAAAAATGCAT |
| 23 | GTCAATCTTTAATAAAACATC | 13 | TAAAACATCGTCAATATTTAA |
| 23 | CGTAACTTGATAA | 13 | AAAAACAGTCGTTTTTAACG |
| 22 | CATCGTCAATTGCGGTCACCATTAAAA | 13 | ATCAAATTACGTAAGCGATTACGTA |
| 22 | GTCATTTGCAGTCACCATTGAAAACATC | 13 | ACCGCAATTGACGACGTTTTCAATGGTG |
| 22 | TTGCAGTCAACATTAAAAACATCGTCAA | 13 | GTC |
| 22 | AAACATCGTCAATTGCAGTCACCATTAA | 12 | TTACGTAATCAGC |
| 21 | ACATACATACGT | 12 | CATTGAAAACATCGTCAATTGCG |
| 21 | TAATCAACTTACG | 12 | GTTTTACTAAAGATTGACAAC |
| 21 | ACGATGTTTTCAATTTGACCGCAATA | 12 | TGTA |
| 21 | GCAATTGACGATGTTTTCAATGGTAACT | 12 | GTCAATTAGCGTAAAACGTT |
| 21 | CAATTGACGATGTTTTCAATGATGACTG | 12 | ATTGAAAACATCGTCAATTACA |
| 20 | TCAATTGCGGCCACATTGAAAACATCG | 12 | CATTGAAAACATTGTCAATTGCAGTCAA |
| 20 | TTTTGAGCAAAAAATGCATGT | 12 | AAACATCGTCAATTGCAGTCAATTGA |
| 19 | ATTGACAACGTTTTACGCAG | 12 | TCAATTGCGGTCAACATTAAAAACATCG |
| 19 | TTGACGACGTTTTACACTGA | 12 | TTGAAAACATCGTCAATTGCGTCACA |
| 19 | ATTGAAAACATCGTCAATTGCAGTAAC | 12 | TAAAACCTCGTCAATCTTGAA |
| 19 | TCGCAATTGACGATATTTTCAA | 12 | GCAATTGACGATGTTTTCAATTTACC |
| 18 | AACGTCGTCAATATTTAATAA | 12 | GTTTTCAATTTGACAGCAAATGACGAT |
| 18 | TGTTTTCAATGGTGACAGCAATTGACGA | 12 | AAACATCGTCATTGCGGTCACCATTGA |
| 18 | GACCGCAATTAACGATGTTTTCAATGGT | 11 | AAACATCATCAATTGCGGTCACCATTGA |
| 18 | AAAAACAGCT | 11 | ATTGAAAACATCGTCAATAGCG |
| 18 | TTACGTAAGCGATTACGTAATCAAG | 11 | ATTGACGAAGTTTTATTAAAG |
| 18 | AAACATCGTCAATTGCGTCAATTGA | 11 | TCAATCTTAATAAAACCTCG |
| 17 | TCGTCAATTACGGTCACCATTGAAAACA | 11 | GTCAATTGCAGTAACCATTAAAAACATC |
| 17 | ATTGAAAACATCGTCAATTGCGATCACA | 11 | CATTGAAAACATCGTCAATTGCGTAC |
| 17 | TGACGATGTTTTCAATGGTGACCACAAA | 11 | ATTGAAAACATCGTCA |
| 16 | GACCGCAATTGACGATGTTTTCAATCT | 11 | ATAAAACATCGTCATTATATTT |
| 16 | ACATACGTACGT | 11 | CGATTACGTAATT |
| 16 | AAAACATGCATTTTTCGTT | 11 | ATGTTTTCAATGGTGACTGCAATTGACA |
| 16 | CATTAAAAACATCGTCAATTGTGGTCAC | 11 | CGATGTTTTCAATTTGACTGCAATGA |
| 16 | GGATACTTTGA | 11 | GACCGCAAATGACGATGTTTTCAATT |
| 16 | GACGATATTTTCAATGGTGACCGCAATT | 11 | ATTACGTAATCGATTACGTAATCAA |
| 16 | GTCACAATTGAAAACATCGTCATTTGCG | 11 | GTATCCGCAAA |
| 15 | CCGCAATTGACGATGTTTTAAATGGTGA | 11 | CATCGTCAATTGCGGAATTGAAAA |
| 15 | TGCGGTCAAATTAAAAACATCGTCAAT | 11 | AAAACATAGTCAATTGCGTCAAATTG |
| 15 | GCAATTGAAAACATCGTCAATA | 11 | CATTGAAAACATCGTCAATGCGGTCAC |
| 15 | ATTGACGATGTTTTCAATAGTGACCACA | 11 | TGACGATGTTTTCAATGTTGACCGCAAA |
| 15 | ATCAAATTATGTAATCGATTACGTA | 11 | GTTTTATTGAAGATTGACAAC |
| 15 | TGAAAACATCGTCAATTGCGTAAAT | 10 | ATCGTCAATAGCGGTCACCATTGAAAAC |
| 15 | AAACGTCGTCAATCTTTATA | 10 | TTTACGTAAAAACAGTCGGTT |
| 15 | TGCCGCAATTGACGATGTTTTCAATG | 10 | GCGATTACGTAAC |
| 15 | AAAACAGCTCAAAAACAGCT | 10 | GTTTTACACTAATTGACGAG |
| 15 | TGACGATGTTTTCAATGATGACCACAAT | 10 | AAACGATTTAATTCACTTTTG |
| 15 | GTTAGG | 10 | ATTGAAAACATAGTCAATTGCG |
| 10 | TTGACGACGTTTTACGCTGA |
| 10 | ATTTTATGA |
| 10 | GCAATTGACGATGTTTTCAATGGTGATT |
| 10 | GTTGACCGCAATTGACGATGTTTTCAATG |
| 10 | CGCAATTGACGAAGTTTTCAAT |
| 10 | TTTCAAAAGCAATTGACGATGT |
| 10 | TTACGTAATCGCTTACGTAATCGC |
| 10 | TTTTCAATCGCAATTGACAATG |
| 10 | GAAAACATCGTCAATTGCAGTAAAATT |
| 10 | CAATTGACGATGTTTTCAATGGTGACT |
| 10 | GACGATGTTTTTAATTGTGACCACAATT |
| 10 | ATTGAAAACATCGTCATTTGCGATCAA |
| 10 | TGACAACGTTTTATTAATGAT |
| 10 | TTTTCAATCGCAATAGACGATG |
| 10 | CGATGTTTTCAATCGCATTGA |
| 10 | GATTACGTTACTT |
| 10 | CCTGATTCAAAACCCACTTTA |
| 10 | CGTAAATTGATTA |

| **Bembidion subfusum (#SRR2939014)** | | | |
| --- | --- | --- | --- |
| 228 | GGAAT | 8 | AAAAAAAGAA |
| 197 | AAC | 8 | TAG |
| 181 | GGGGGGGGAG | 8 | AAAAAAAAG |
| 175 | AGGC | 8 | TTAAAAAAAAAA |
| 172 | GGGGAGGGGGG | 8 | GGGAGAGGGGGG |
| 168 | GAGGGGGGG | 8 | GGGGGCGG |
| 165 | AAG | 8 | TTTTTTAGCTG |
| 141 | CAGC | 8 | TGTTGT |
| 125 | TCA | 7 | AAAAAAAAACAA |
| 109 | CTCAAAAACAG | 7 | GGGGGGGGGGGAGA |
| 105 | AGCGGC | 7 | AAAAACAGT |
| 97 | **GGTTA** | 7 | TTGTTT |
| 90 | GTGGGTTTTGAATCAGGTAAA | 7 | AAAAAAAT |
| 90 | TAGCTGTTTT | 7 | AGGGGGGGGGGGA |
| 87 | GGAGGGGGGGGG |
| 87 | CGGGGGGGGGG |
| 86 | TGCTGT |
| 56 | GGGGAGGG |
| 48 | GGAGGGGGGGGGG |
| 47 | ACGTGTTTTT |
| 46 | GTTTTTTTTT |
| 46 | AAGTGTTTTT |
| 43 | TAATCGATTACG |
| 39 | GGGGGGGGGGCG |
| 38 | GGGAAGGGGG |
| 32 | AAAAAAAAAAC |
| 31 | GTTTTTAGTGAAAAAACGACT |
| 27 | GGGGGGGGGC |
| 27 | GATGAA |
| 27 | AGGGGGGGGGGGGG |
| 25 | CTGATTCAAAACCCACTTTAT |
| 24 | ACAAAAAAA |
| 24 | GGGGGGA |
| 23 | GTTTTTAAGC |
| 23 | TGCGCCTTCA |
| 23 | TAT |
| 21 | TTTACCTGATTCCAAACCCAC |
| 19 | TTTC |
| 17 | TTCTTCTTT |
| 17 | CCCCAAAA |
| 16 | TTTTCC |
| 15 | GGGGGGGGC |
| 15 | ACAAAAAA |
| 15 | GGGGGAAGGGG |
| 14 | TGTTTTGAGC |
| 14 | GAGGGGGGGGA |
| 13 | CCAAA |
| 13 | AAAAAATAAA |
| 11 | AAAGAAAGAAGCAGAAGAAAAAGC |
| 11 | AAAACAGCTTA |
| 11 | AAACACGTGTTT |
| 10 | GGGGGGCGGGGGG |
| 10 | GGGGGGACGGGG |
| 10 | AAGAGG |
| 9 | TGAAGATGA |
| 9 | GGGGAAGGGGGG |
| 8 | GGACGGGGGGG |
| 8 | GGAGATCACTG |
| 8 | AAAGAAAA |
| **Bembidion aeruginosum (#SRR5230397)** | | | |
| 4734 | AAT | 146 | TTTAGTT |
| 1650 | TTAT | 144 | ACAAAA |
| 1285 | AACCT | 144 | TTAATTTA |
| 1224 | ACAT | 143 | ATTTAGGAATTCGAC |
| 982 | AGTTAATTAAAATATAA | 142 | TAGCACAAACC |
| 887 | AAC | 137 | TAAT |
| 878 | CCATATTTCCCAATGG | 137 | AAAATA |
| 877 | ACAACTTTCAAAGACGTTCACCGCTGTGCTCAC | 135 | TCTATACTATCCGGACGCGAAGCG |
| 829 | ACTTTTACGCCCGCATCGTT | 131 | TTTTTCTATAC |
| 780 | GTCGAAAATACCACGGCACGTAGAC | 131 | TTTTCGGTGAATTG |
| 663 | ATATAAA | 129 | TGCGTGCGGTAAAATAAAT |
| 650 | CACACAATTTTCAAAGACGTTCACCGCTGTGCT | 126 | GTAAAAATGTATGGTG |
| 600 | CATACGTA | 125 | CATAG |
| 531 | ACACACAT | 122 | AAATACTTCCAATCGC |
| 508 | ACTTTTACGCCCGCTGCGTT | 118 | ATTTTTACCACCAGAC |
| 453 | AAATA | 115 | ATTTATAC |
| 452 | ATTTTTCATCTTCCTATTTTTTC | 114 | ATTTTTACCACCATAT |
| 447 | TCCCAAATGTCGAATAG | 113 | TATATTA |
| 433 | ATGTTTTC | 109 | ATATAA |
| 424 | TTTTTCATCTTCCTATTTT | 109 | TAATTTTGTCTCAACA |
| 403 | CTGTGTGCGCTTTTTT | 108 | ATCT |
| 334 | GAAGGTGAAAAAAGAAAAATAAAATAGTAG | 105 | GACGCTTCGCGTCTGGATAGTAAA |
| 329 | ACACAAAAATGTCCAAAAACTC | 104 | AACTATCGTTA |
| 326 | TTTGTGT | 102 | TACATTT |
| 319 | TATATTTTAATTAAC | 102 | TAAATTG |
| 300 | AGTATAA | 102 | TGTGTGTATGTG |
| 286 | TCGAATGGTCCCAAATG | 100 | AATATT |
| 283 | TAAATTA | 98 | ATTTTGT |
| 280 | TTC | 97 | GTAGGAAGGTGAAAAAAGGAAAATAAAATA |
| 272 | TATTA | 94 | GTCTTTGAAAAATTGTGTGAGCACAGCGGTGAAC |
| 258 | GTCTATACTATCAGGACGCGAAGC | 94 | ATTTTCTGGTCTCAG |
| 238 | ATGTATGTACGT | 94 | TCGACATTTGGGAA |
| 237 | CAT | 92 | CTAAACGCAATTTTTC |
| 233 | TTTATTTTACCGCACGCAT | 92 | CAAAA |
| 232 | GCGAATGGACAGATGA | 92 | GTC |
| 224 | ACTTTTTTTC | 92 | TAATTTTTAATAATTATA |
| 218 | AATAAATACA | 89 | GAATA |
| 216 | TACG | 89 | AAGTGATTCTTTTAGTCCCTAGGGAGTAA |
| 200 | GCTAAGTTTA | 87 | TTTTTTA |
| 190 | ACT | 86 | AGCACTAACGA |
| 189 | AAATGTCGAATTCCC | 86 | AGCACAGCGGTGAACGTCTTGAAAATTATTTT |
| 188 | TTCATTTTTCATCTTCCTTTTTT | 86 | ATAATT |
| 180 | TATCAGGACGCGAAGCGTCGAAAATAC | 85 | GATAAGAGAAAT |
| 179 | GTATAGACGTCTACGTGCCGTG | 84 | ATTTTTACCACTCC |
| 172 | TTTTTCA | 82 | ATACTATCGGACGCGAAGCGTCGAAA |
| 167 | TAATTTAAAAT | 81 | GCCGAGTTGATCCGC |
| 164 | CGTTAAAAACATGAA | 80 | CATCAG |
| 163 | GGGAAATATGTCCATT | 80 | TTTTTACCACTATATT |
| 163 | TGC | 79 | CAAA |
| 162 | CGTACGTATGTA | 77 | ATTTTTAAGGACAAATTATATGAA |
| 161 | CGTTAGTTTTAAGTCG | 76 | GATCAACTCGGCGCA |
| 159 | TTTTTGCAATTTTCTTCCTATTTTT | 76 | TATTG |
| 159 | TTTATTA | 76 | GTCTTTGAAAAATTTGTAGCACAGCGGTGAAC |
| 159 | GAAGCACAAAC | 75 | AATTTT |
| 156 | ATTAA | 74 | CTCACACAACTGTAAGACGTTCACCGCTGTG |
| 155 | ATCCAGC | 73 | GTTGCT |
| 151 | TTTTTTATTTTTCTATCGAGCACT | 73 | ATTATTA |
| 150 | AAAAATAATAAA | 73 | ATACTATCCGGACGCGAAGCGTCGAAA |
| 148 | AAAAAATAGGAAGAG | 72 | GAAATACGCTGGCCCGTTAAAAGTAAA |
| 72 | ATATTTA | 51 | TTTTTTTTTAT |
| 72 | TGTATGTGTG | 51 | AGACGTTCACCACTGTGCTCACACAACTTTCAA |
| 72 | AAATAAAA | 51 | AAAATATA |
| 71 | GTGTAT | 51 | CATTTTTCATCTTCCTTTTTTT |
| 71 | CGTTTCGCCGCGGC | 50 | GCGCCGAGTTGATCG |
| 70 | TTCTTCCATCCTTCTGGAAGTTTCTTTGGCCTC | 50 | GCCCACCG |
| 70 | TAAATAAAA | 50 | GAGGAAAAATGAAAAATACATTATTAGGAA |
| 70 | TGAAAGTTGTGTGAGCACAGCGGTGAACGTTTC | 50 | ATCCAC |
| 70 | CCAATCGCAAAATCAC | 50 | TAAAATATTTTA |
| 69 | ATTCGACATTTGGG | 49 | AAATG |
| 68 | TATTTTATTGAATACATTT | 49 | TTCA |
| 67 | TTTCTGTG | 48 | ACGTCTTAAAAAATTGTGTGAGCACAGCGGTGA |
| 67 | ACCA | 48 | AAAAAAAACA |
| 66 | AAAAACAGCTC | 47 | CTCACACAACTTTAAAGACGTTCACCGCTGTG |
| 65 | AAAAAAAAT | 47 | TTGAATTCAACAATTGTTCAACAATTGATCAT |
| 65 | ATACTACGGCACGTAGACGTCT | 47 | GATTTTACGATTAGGT |
| 65 | CTTGCGTTACTT | 47 | TATATTAT |
| 65 | CGCGCCGACTTGATC | 47 | AAAAGCGCACATACC |
| 64 | TAACA | 47 | TTTTTATTTTTCTATCGAGCACAT |
| 64 | ATTTTAA | 46 | AGCACAAACG |
| 63 | CTTTTG | 46 | GTTTTTCTGGACG |
| 63 | AACTGC | 45 | CAAATAAAATA |
| 61 | TGTTTTTTT | 45 | TATATTTTTTACCACCA |
| 61 | GACA | 45 | ATATTAATA |
| 61 | AGGAAAATTCTTCGAA | 44 | TATAATTATAA |
| 61 | ACTTTATATTACT | 44 | TGTGAGTTTTTGGACATTTT |
| 61 | ACGAAT | 44 | CGTCAAAAATACCACGGCACGTAGA |
| 61 | AAAAAAGCGCACATAG | 44 | CCGCGACTTAAAACTAA |
| 60 | CTGTCGACCTTTTTCGGC | 44 | GATCAAGTCGGCGCGA |
| 59 | ATATGTATTA | 44 | CCGAGCA |
| 59 | GACGTCTACGTCCGTGGTATTTTC | 44 | GTATTT |
| 59 | CGAATTGTGTAGGAGA | 43 | TGTG |
| 59 | TTATATGTGTATTTTTAAGGACAAA | 43 | TTACAA |
| 58 | AAAAAAAATA | 43 | GTCGACAGGCAGAAAAAG |
| 58 | GATCAACTCGGCGCGA | 43 | AAATGAAAAAATAGGAAGAGGAA |
| 58 | CCAACA | 43 | TCAA |
| 57 | TATGTCAATT | 43 | GGATAAGCTCTTAGGACAAAT |
| 57 | TAAAG | 43 | AGGCACTAATT |
| 57 | AATTC | 43 | AAAAGGTATAG |
| 57 | TTCATTTCATC | 43 | AGTTAATTAAAATATA |
| 57 | TTGTTCAAGATA | 43 | AATTCCCAATCCAT |
| 56 | AGCACAGCGGTGAACGTCTCTGAAAGTTGTGTG | 42 | TTGAGCGGTTT |
| 56 | GATTTTGCGATTAGGT | 42 | CGCGCCGAGTTGACCT |
| 56 | TTTAAA | 42 | TTATATAATTATATATTT |
| 56 | AAAAACA | 42 | ATATTTTGTACGAAAATACCGCAA |
| 56 | AAAATC | 42 | GCTTCA |
| 56 | TTTCCGTGTGCGCTTT | 41 | TTGAAAA |
| 55 | TTACACATAT | 41 | TGTGAGCACAGTGGTGAACGTCTTAAAAAATTG |
| 55 | TAGACGTCTACGTGCCGTGGTATTT | 41 | TCGACATTTGGGACAT |
| 54 | CACCAT | 41 | GACATTTGGAATTC |
| 54 | ATTTATAT | 41 | TGTTGGA |
| 54 | AAAAATGAAATAGACATTAATAGGAAGAGG | 40 | AAAAAATATAGTGGT |
| 54 | CTTAAAACTACCGCGA | 40 | TGCCGAATTGTCGTTG |
| 54 | CAAACT | 40 | CATATATA |
| 54 | TATGGCCATTGGGAAT | 40 | ACTGAAA |
| 53 | TTTATTTTTA | 40 | TTTTATTA |
| 53 | GACGTCTACGTGCCGTGATATTTTC | 40 | TTTTCCTATAATTGTTTTGCATTTTTCC |
| 53 | AGAAGC | 40 | TGTGAA |
| 52 | TACAAAAACCGAAAA | 40 | ATAGTA |

| 39 | TTTCGGTTTTTGTAC | 30 | ATGGTGGTAAAAATGTC |
| --- | --- | --- | --- |
| 39 | TAAAATATA | 30 | AAACGA |
| 39 | CATAGCACAG | 30 | AACACTG |
| 39 | AGTTTA | 30 | GCAATTT |
| 39 | ACAAACGCAACAAAATTAACA | 30 | TATCAGGACGCGAAGCGTCGAAAA |
| 39 | ATTTCAA | 30 | ACATACAGACAT |
| 39 | TTCTTTTTTT | 30 | GCAGTCTTAGCA |
| 38 | ATTTTACA | 30 | TCGTATT |
| 38 | GTCGAATTCCCAAAC | 30 | TTATAAAT |
| 38 | GTTGAT | 29 | CTATGTTATG |
| 38 | TAATTTT | 29 | CGAACTAAGTCCGGACCCAAAAAATC |
| 37 | TAGTATTTCGACGCTTCGCGTCCTGA | 29 | AACTGTA |
| 37 | TTCGGTATTTTTAAAGCTT | 29 | ATTTTACT |
| 37 | ATACATAC | 29 | TACGGC |
| 37 | CGCGATTAATCGAATAAC | 29 | TCTTTTTTT |
| 37 | AAAATAAAATGTATTTAAT | 29 | AAAGAA |
| 37 | AAATGA | 29 | TACGT |
| 36 | TTAGT | 29 | ATGTCTGT |
| 36 | TATAATTGTAA | 29 | TTTTCTTTTTT |
| 36 | TTGTAAT | 29 | CACTGT |
| 36 | CAGAACGAAAAAGACTTTTACGTC | 29 | ATGCAAAAATATTATTAGGTACAGGAAAA |
| 36 | TCCATAATTCCCAG | 29 | CTTTATTATTTTAATTTATAGTTTTT |
| 36 | TGTAAACT | 28 | ATTAC |
| 36 | AAAATATAATAGGTACAGGAAAAATGCAA | 28 | AGTTTTGCTTATTTAGTTCAGTAAAGTTAA |
| 36 | GAG | 28 | TTTTTTTATTTT |
| 35 | TTTTC | 28 | TGAGCGAATCCAGT |
| 35 | ATTATTAATT | 28 | CTCACACTCAT |
| 35 | GACGTCAACGTGCCGTGGTATTTTC | 28 | TCTCGT |
| 35 | CGTCCAGAAAAA | 28 | GTGAACGTCTCTTAAAGTTGTGTGAGCACAGCG |
| 35 | CGATTGGGTGATTTTA | 28 | TCAACTG |
| 35 | CGAATTGTGGGAGA | 28 | TTCTAGA |
| 35 | CGAAGCGTCGAAAATACCACGGCA | 28 | TCTATTT |
| 35 | AAATATC | 28 | TATACTA |
| 35 | CTCAGA | 27 | ACAGTTGAATAACATCAACT |
| 34 | GTCGAAGAAACTTCCAGAAGGATGGAAGAAGAG | 27 | TTAAATGCAAAAGACTCTAAAG |
| 34 | TTCGCAT | 27 | TTATTTATTA |
| 34 | CACAGCGGTGAACGTCTTGAAAATTGTGTGAG | 27 | TTTTGCGGTTAACTTTAGAGTC |
| 34 | AATTGTGTGAGCACAGCGGTGAACGTCTCTAAA | 27 | TGTATGTGTGTA |
| 34 | AATGTTTTTAA | 27 | CAGAATC |
| 34 | TTTGTTAGTGC | 27 | CGTCTTGAAAGTTGTGTGAGCACAGCGGTGAA |
| 34 | GGTTAATCGATTAATCGC | 27 | GTG |
| 33 | CTATTG | 26 | ATTAAATA |
| 33 | AACTT | 26 | TATATTTTAAATAACTT |
| 33 | TTTTTAAAGACAAATTATATGAAA | 26 | ATTCAGT |
| 33 | AGTGTGACGTCAGTGTGTG | 26 | CAAAATATCACGAATATATATTTG |
| 32 | TCTTCA | 26 | ATTTCCCAATGCCAT |
| 32 | CGGTGAACGTCGTTGAAAGTTGTGTGAGCACAG | 26 | TAAATAAATA |
| 32 | CTGAATT | 26 | TCAATATATCGACTTTATTC |
| 32 | TTTTACT | 26 | ATATTTGCAAATATATATTCGTG |
| 32 | CGTCGAAAATACCACAGCACGTAGA | 26 | TCATATT |
| 32 | GCATTTT | 26 | CAAATATTTGCGGTATTTTCGTA |
| 32 | ATAGACCTG | 25 | CATCTA |
| 31 | CATCCCAG | 25 | GGAAAAATGCAAAAAAATTATAGGAAAA |
| 31 | AGTAGC | 25 | CGTTGAGCCGAATTGT |
| 31 | TTGTGGTTCTTGTATAGTCGTCTCCAC | 25 | TTTTACTGCCGGCAACAAAAC |
| 31 | GACGTTCACCGCTGTGCTCACACAACTTAAAA | 25 | CGCAACAAAAATAACAACAAA |
| 31 | TGGTA | 25 | TGGTT |
| 31 | CTTT | 25 | AAACGTA |
| 31 | TTTATAG | 25 | ACAATTCGTCTTCC |

| 25 | TACAAC | 20 | GAAAATTCTTCGAAA |
| --- | --- | --- | --- |
| 25 | ATTTTTCATCTTCCT | 20 | TTCACCGCTGTGCTCACAAAACTTTCAAGACG |
| 25 | ACTGCATACTGCTGGAA | 20 | TTCTGTG |
| 25 | CTTACAAGTTGTGTGAGCACAGCGGTGAACGT | 19 | CAGCAACAA |
| 25 | ATAAAACGG | 19 | AAAAAAAC |
| 25 | AAAATACAAAAAA | 18 | GGACAAATGAGCGAAT |
| 25 | TTTTTGCAATTTTCTTCCTATTTT | 18 | CTCACACAACTTTCAAAACGTTCACCACTGTG |
| 25 | TTGTGTGAGCACAGCGGTGAACGTCTTTAAAAAAA | 18 | TGAGTGTGAGT |
| 25 | CTCTTCCTATTAATGTCTTTTTCATTTTTC | 18 | ATGTTTCC |
| 25 | AAACTACCGCAACTTA | 18 | GAAATACT |
| 24 | AAAATATATTATTATATA | 18 | TAATAATATTTAA |
| 24 | AATTTC | 18 | ACTTTTACGCCCGCATAATT |
| 24 | TACAGGAA | 18 | GGAATCGACATTTA |
| 24 | CTGATT | 18 | CGTCTAAACTATCCAGACGCGAAG |
| 24 | TTTTTTTGTATT | 18 | TTATATTTATAA |
| 24 | CTAATGTAT | 17 | TATTCTG |
| 24 | AAATTCACC | 17 | GACGCTTCGCGTCTGGATAGTATA |
| 24 | GAAAAATAAATA | 17 | CGTGTCCGGATTGTATAGACGTCTA |
| 24 | GTAA | 17 | TTTCGAAGAATTGC |
| 24 | GTGAAC | 17 | AACTATAGTTA |
| 24 | TCAATAT | 17 | TATTAAAT |
| 24 | **CGTGGT** | 17 | CGGACAAAACCGCGAA |
| 24 | AGCGTCGAAAATACCACGGCACGT | 17 | AAAAAAAAAACA |
| 24 | ATTTAC | 17 | ATTGCGA |
| 23 | CCAACAA | 16 | ACTGCAT |
| 23 | ACAAATAAATT | 16 | AAAAGGATACAAACGAAAAATTA |
| 23 | GCTATGCAGT | 16 | CCAGAAAAACCAT |
| 23 | CTTTTGACGGCGTGGA | 16 | CTACATT |
| 23 | TTCAC | 16 | AGAAAGGTCCAACGGCGGAAGATCCCTGA |
| 23 | AATTAAATTTAGGTTTTTCGCTAAAATTA | 16 | CTGTGCTCACACAATATTTTAAAGACGTTCACCG |
| 23 | GAAATTATACTTA | 16 | ATTTTATAAA |
| 23 | AGAAAAA | 16 | GATTTTCTGGTTTCA |
| 22 | AAAATTAC | 16 | TATCACGGCACGTAGACGTCTATAC |
| 22 | TCACCGCTGTGCTCACACAACTTTCAAAACGT | 16 | TGAAATG |
| 22 | GACGCTTAGCGTCCTGATAGTATA | 16 | TTCCTAAACGTAATTT |
| 22 | CGTCTAAACTATCAGGACGCGAAG | 16 | TAAAGTTAACTTTAATT |
| 22 | ACTTTTACGCCCGCTCGTT | 16 | AATTCAC |
| 22 | AATTCCCAATGCCAT | 16 | AACTTAACTTT |
| 22 | CTTCATCTT | 16 | TTTACTTTTAACGGACCAGCGTATTTC |
| 22 | AGGAAAATTCTTCGA | 15 | AAAAAAAAACAAA |
| 22 | GTTA | 15 | AAAAGCGCACATAC |
| 22 | CATTCGCTCATCTGG | 15 | ACTTATAATAATAT |
| 21 | TCGT | 15 | TGCTGTA |
| 21 | GAAAAAAGCGCACACC | 15 | GAAGTA |
| 21 | GACGTCTGCGTGCCGTGGTATTTTC | 15 | AGAAATGTCCAACGGCGGAAGATCCCTGA |
| 21 | GGACAAAACCGCGAC | 15 | ACTTGACTGAACTAAAATAGCAAAACTTTA |
| 21 | AAATTTATA | 15 | ACGCAA |
| 21 | ATGTAT | 15 | TCGCGCCGACTTGA |
| 21 | AATTAG | 15 | TCTCTAAAAATTGTGTGAGCACAGTGGTGAACG |
| 21 | AATAAAATT | 15 | AAGTCAATATATTGAGAATA |
| 21 | ATGAGCGAAAGCCAG | 15 | TTACGATTAGGCTATT |
| 21 | CGTCGAAAATACTACGGCACGTAGA | 15 | AAAACTATAAATTAAAAAAATAAACTAAAGA |
| 21 | CTGCCGA | 15 | ATTCTTCGAAAGAA |
| 21 | ATATATTAT | 15 | AGACGTTCACCGCTGTGCTCACACAATTTTAA |
| 20 | ATTTATATAT | 15 | CTTGCATTACTT |
| 20 | TTTTAATAAAGTTATCTAG | 15 | TTAATATA |
| 20 | TTTAAATA | 15 | TTCTATTTGATTTTCAGAAAT |
| 20 | CCTTCCTACTATTTTATTTTTATTTTTTCA | 15 | AGGAAAAATGCAAAAAATATTAATAAGAAT |
| 20 | CTGCGCCGAGTTGAC | 15 | TGTTC |

| 14 | GTCCTTAGTAAAATATGAT | 12 | ATAGCC |
| --- | --- | --- | --- |
| 14 | CAGCATCCA | 12 | AAAAAATTA |
| 14 | TAGCTGTTTT | 12 | TGTGTG |
| 14 | AAAAACG | 12 | AAGATC |
| 14 | TATAAAAATA | 12 | AAAATAGAGTGGTA |
| 14 | AATGTTC | 12 | CCCCACCCC |
| 14 | ACGTGCCGTGGTATTTTCGACGCTTA | 12 | AGTATA |
| 14 | TTTTCAAGACTTTCACCGCTGTGCTCACACAA | 12 | TTAACATT |
| 14 | GTAGTCG | 12 | GAATAA |
| 14 | AACTGTAA | 12 | AATAACTAAACTAA |
| 14 | TTATGTGAAACAG | 12 | TTATTTTTTCTACTAT |
| 14 | AAATGAATTTCAGATATCAA | 12 | TTATTG |
| 14 | TTTTAAAA | 12 | TGCAAAAA |
| 14 | ATGTTGTCATGTTTTC | 12 | TGAAATTTCAAGAAAAAAGG |
| 13 | GTAAAC | 12 | CATTTATCCTTCTATG |
| 13 | TTAAAAAT | 12 | TACCCGT |
| 13 | ATTATAAATCTAA | 12 | TAAATAC |
| 13 | ACTTTTACGCCCGCATAGTT | 12 | AAATACAATGATCGCGAGAGA |
| 13 | TCACCACTGTGCTCACACAACTTTCAGAAACGT | 12 | TTAAATATAAAGGAAGATTC |
| 13 | GTCTATACTATCAGGACGCGAAAC | 12 | TTTTAAATA |
| 13 | CCACAAT | 12 | CTTTG |
| 13 | TATTTTAATAAAA | 12 | AAGAATC |
| 13 | GGTTGT | 12 | CTACTAAGCTCACATACCTAAAAGGCATGTGA |
| 13 | ACTTTTACGCCCGCATCATT | 11 | AATAAAATAAT |
| 13 | GGTATATGGGACAACCTTA | 11 | TGCAATG |
| 13 | TTTAGTAAATATTACG | 11 | TCGTTCG |
| 13 | AAAACAATTCACCGAA | 11 | GAGAATT |
| 13 | GTCTGTGT | 11 | CTTTTGCTTCTG |
| 13 | TTCGTAA | 11 | GAAAGATCCCTGAA |
| 13 | TTTGGAAATTCGACATTCGGGAATTCGACA | 11 | TTTTTTTATTTTTCATCTTCCTA |
| 13 | GGTGGTAAAAAAATAT | 11 | AATTAACACTA |
| 13 | TAGTTTTAAGTCGCGA | 11 | AGTGCACATAATTTGTCCTTAAAA |
| 13 | CGTCTTTAATATCCAGACGCGAAG | 11 | AAGAAAATTCCAGAAGGATGGAAGAAGAGGCCA |
| 13 | GTTCGCCGCCAAGCCA | 11 | AAATATTAA |
| 13 | TTGATA | 11 | ATGATGTTG |
| 13 | TATTTATG | 11 | TTTAATG |
| 13 | TAAATATCTGAATATAACTAAG | 11 | TTGTTG |
| 13 | TGCGGGCATAAAAGTAACGA | 11 | CTATCCACAGAATCG |
| 13 | TTATTAAAAAA | 11 | AAAGAAATTTCG |
| 13 | AACTGCAG | 11 | TAAAACA |
| 12 | TGGATAC | 11 | TACTTTTTT |
| 12 | AACAGCTCTTAATACAGGGTAGA | 11 | CGTCTTAACTATCCAGACGCGAAG |
| 12 | GCAGTTCAGCA | 11 | ATTTTCAG |
| 12 | CATAGAACATTGAA | 11 | AGTTATTTAAAATAT |
| 12 | TAGTTTTAAGTCGCGGG | 11 | CAGAAC |
| 12 | ACAACG | 11 | GACGTCTACGTGCCGTAGTATTTC |
| 12 | AATTTAAGCAAGACTTAA | 11 | GTGTGAGCACAGCGGTGAACGTCTCTAAAAGTT |
| 12 | TAGATTG | 11 | GCTTCGGTGAATCTGGC |
| 12 | TATTATTTCATTTTA | 11 | GACGTCAACGTGTCGTGGTATTTTC |
| 12 | AAAAATATAGTGGT | 11 | ATTTATTTATT |
| 12 | CTATTTA | 11 | TAACAT |
| 12 | ACAAAGCCGCCAACTCGGCGCA | 11 | AACTAA |
| 12 | TAATATGT | 11 | TTTATTTCACATGTTCTTATT |
| 12 | CCATATTTCCCAGT | 11 | GTGTTTTCTATATGATTTGTATACGTATTTTTTAA |
| 12 | ACCGCAACTTAAAACTACCGCGACTTAAAACTA | 11 | TATAG |
| 12 | AATGTTG | 11 | ATGGTAG |
| 12 | CCTTTTTAATATTTCGTTTGTAT | 11 | CTGTTTTGAG |
| 12 | GGTGGA | 11 | ATTTG |
| 12 | CGAACAA | 11 | AAATGTAA |

| 11 | TACAAACATTGGC | 10 | ATCAT |
| --- | --- | --- | --- |
| 11 | CATTTATG | 10 | ACTTTTTC |
| 11 | AGACGTCGATAATACCACGGCACGT | 10 | GATGTAG |
| 11 | AATGTAGA | 10 | ACATACATACGG |
| 11 | TATTATAA | 10 | CGTCGAAAATACCACGGCATGTAGA |
| 11 | ACTGCTGCTAA | 10 | ATTTTCCTAAGAGCTTATCC |
| 11 | TTTAATTCTTAA | 10 | TCCTCAA |
| 10 | AGTTGACTCGCGCCG | 10 | CCAAAT |
| 10 | ATTCTTACATTTCTTGCTTGTTTGAA | 10 | ATGAACAAATGAGTTCAATGTTTGCAAATAT |
| 10 | AACAGCTCTTAAACAGGGTAGA | 10 | TGGCGACGACC |
| 10 | CGACAGAAACAT | 10 | CATTGTTAACTTTAATCA |
| 10 | CTAAACGCAATATTTA | 10 | TACGTATA |
| 10 | AAATGTGCCTAATAGTA | 10 | TTGGGAAATATAGCCA |
| 10 | AACGACGTAAAAAGACGATAGAATTAA | 10 | TCAACAAACAACTTCAAATTCACG |
| 10 | AAAAGTAACGCATGCGGGCGT | 10 | GGCGGCGAACTGGCC |
| 10 | TTTTCGAAGAATTGC | 10 | TAATATATAA |
| 10 | AAGAAAAACTTCTAGAACAATCCAAAATATTGG | 10 | AAAATACGTATACAAATCATATAGAAAACATTAAA |
| 10 | CATCTCG | 10 | ATCAACA |
| 10 | GTACTCCTGCAGCACTATCAATTCAAAAAA | 10 | AGGAAGATGAAAAATAAAAAAA |
| 10 | TGTTTAG | 10 | TTAATTAAAACTAGATAAC |
| 10 | ATTTAAAC | 10 | TAAGCTCTTAGGACAGATGGA |
| 10 | TCTGGCTT | 10 | TTTCATAT |
| 10 | TCTTCG | 10 | TAAACTGCTGG |
| 10 | GAAGATAGA | 10 | CTAAAG |
| 10 | TTTGATATTTTGATATT | 10 | AGTTAAAGTTTTGCTATTTTAGTTCAGTAA |
| 10 | TCATAT | 10 | ATATAATT |
| 10 | ATATATTTCAGACATAATATACAAATTT | 10 | ATTTTTTTTA |
| 10 | ATGTTGTATGTTTTC |
| 10 | ATTCACCGAATAACA |
| 10 | CGGAAAAT |
| 10 | CGAAGACGTCGAAAATACCACGGCA |
| 10 | CTGGAAAA |
| 10 | TGTGAAAACGTTTTCGCACGCT |
| 10 | AAAAAAAATACAAA |
| 10 | CCTGTC |
| 10 | ATTTTCAA |
| 10 | AGATTC |
| 10 | CTTATCCATTTTTCCTAAGAG |
| 10 | AGAAGGC |
| 10 | AAATTTATT |
| 10 | GACGTCTACGCGCCGTGGTATTTTC |
| 10 | CATTGTTAACATTAATCA |
| 10 | GACGCTTCGCGCCTGGATAGTAAA |
| 10 | TTTTTCATCTTCCTT |
| 10 | ACGTTCACCGCTGTGCTCACACAACTTTAAAAG |
| 10 | ACGATTA |
| 10 | AAAAGAGGAAGATGAAAAA |
| 10 | AATTTCAA |
| 10 | GCTGTA |
| 10 | AAAATAAC |
| 10 | GAAGGTGAAAAAAAAAAATAAAATAGTAG |
| 10 | CGAAGCGTCGAAAATACCACGACA |
| 10 | TTTTTTCACCTTCCTACTATTTTATTTTC |
| 10 | CGTCTACACTATCCAGACGCGAAG |
| 10 | GTTTTCTATATGATTTGTATACGTTTTTTTAAAT |
| 10 | TAATTTAAAT |
| 10 | AAAAATGGATTCTATAAAATCCACTTTTGC |
| 10 | TAGAAAAAAGAATTAA |
| 10 | TAAGTTAGGT |

| **Bembidion breve (#SRR5514454)** | | | |
| --- | --- | --- | --- |
| 46258 | TTCATATATTTGCAAACATTGAACTCATTTG | 1586 | AGGTT |
| 24922 | TTTGCAAAAATATGAACAAATGAGTTCAATG | 1583 | CATATTTCATATTTTTACAAATACTAAACTC |
| 18783 | ATATGAACAAATGAGTTCAATGTTTGTAAAT | 1572 | CAA |
| 16981 | ATTTGCAAACATTGAACTCCATATTTCATAT | 1551 | TTACATTCACCATCAATTTCCC |
| 12267 | TTCATATTTTTACAAATACGAAACTCATAT | 1544 | ATATGAACAAATAAGTTTAATGTTTGCAAAT |
| 11432 | AACTCATTTGTTCATATTTTTACAAATACGA | 1501 | TTTGCAAAAATATGAAATATGGAGTTTTGTA |
| 11284 | GAATGAAATTGATGGAGAATGT | 1498 | TTCATATATTTACAAACACTGAACTCATTTG |
| 11275 | CAAGGAT | 1479 | AACTCCACATTTCATATATTTGCAAACATTG |
| 9896 | TTTCATATATTTGCAAACATTGAACTCATT | 1467 | ATATGAAATATGAAGTTTCGTATTTGTAAAA |
| 8229 | TTTGCAAAAATATGAAATATGAGTTTCGTA | 1378 | CAAATGAGTTCAATGTTTGCAAATATATGA |
| 8155 | TTCATATATTTGCAAACATTAAACTCATTTG | 1376 | GTAAAGAAATTGTATGCTGGCCCGTTAAAA |
| 7505 | AACTCATATTTCATATATTTGCAAACATTG | 1374 | TTTGCAAAAATATGAAATATGAGTTTAGTA |
| 7370 | TGGAGTTTCGTATTTGTAAAAATATGAAATA | 1347 | TTTGCAAAAATATGAACAAATGAGTTCAGTG |
| 6940 | TTTGCAAATATATGAACAATTGAGTTCAATG | 1344 | TATTTCCCTTACATTCACCATC |
| 6471 | AAACTCCATATTTCATATATTTGCAAACATT | 1340 | AACTCATTTTTCATATATTTACAAACATTG |
| 6379 | TTTACAAATACGAAACTCATATTTCATATA | 1312 | AACTCCATATTTTATATATTTGCAAACATTG |
| 6356 | ATT | 1308 | ATATTTGCAAACATTGAACTCATTTGTTC |
| 5023 | TATATTTGCAAACATTGAACTCATTTGTT | 1298 | TTCATATTTTTACAAATACGAAACTGCATAT |
| 4985 | TTTGCAAAAATATGAAATATGGAGTTTCGTA | 1294 | TGTAATGGGAAATGATGGAGAA |
| 4327 | TTCATATTTTTACAAACATTGAACTCATTTG | 1259 | AAACTCATTGTTCATATTTTTACAAATACG |
| 4114 | TATAA | 1244 | AACTCATTTGTTCATATTTTTACAAATATGA |
| 4012 | TAAACTCATTTGTTCATATTTTTACAAATAC | 1236 | ATATGAAAAAATGAGTTCAATGTTTGCAAAT |
| 3732 | TTCATATTTTTGCAAACATTAAACTCATTTG | 1185 | TTCATATTTTACAAATACGAAACTCATAT |
| 3578 | TTTGCAAAAATATGAACAATTGAGTTCAATG | 1183 | ATATGAACAAATGAGTTCAATGTTTTCAAAT |
| 3539 | ATTTTTACAAATACAAAACTCATTTGTTCAT | 1174 | AACTCATATTTCATATATTTACAAATACAA |
| 3489 | TTTGTAAATATATGAAATATGGAGTTTCGTA | 1172 | TTCATATATTTACAAAAACAAAACTCCATAT |
| 3272 | TCGATCC | 1171 | TTTGCAAATATATGAACAATTGAGTTTAATG |
| 3257 | AACTCAATTGTTCATATTTTTACAAATACGA | 1166 | ATGT |
| 3228 | ATGAGTTTAATGTTTGCAAATATATGAAAT | 1161 | CCCTACTTCACCTAATTTAG |
| 3204 | CAAACATTGAACTCAATTGTTCATATATTTA | 1157 | GAATAGAAAAAATAGAAAATGTGCTCGGTTG |
| 3114 | ACACAACGTTAATTTACTTTTTTTTCCATC | 1153 | TTTCATATTTTTACAAATACTAAACTCATA |
| 3052 | TAAACA | 1109 | TTCATAAATTTGCAAACATTGAACTCATTTG |
| 2960 | TTCATATATTTGCAAACATTGAACTTATTTG | 1083 | TTTGCAAAAATATGAAATATGCAGTTTCGTA |
| 2869 | AAACATTAAACTCATTTGTTCATATATTTAC | 1041 | GTTGTCCTCT |
| 2706 | TAATGGAAATAGATGCCGAATT | 1034 | AAACATTAAACTCATATGTTCATATATTTGC |
| 2667 | AACTCATATGTTCATATTTTTACAAATACGA | 1030 | TAAAAATATATGAAATATGGAGTTTCGTATTTG |
| 2571 | AATGTTTGCAAATATATGAAAAATGGAGTTC | 1028 | ATTATTTTCTAACCGAGCACTT |
| 2529 | ATATGAACATATGAGTTCAATGTTTGCAAAT | 1021 | CAAAAGTGTAAATTATATTCCACACTTTTG |
| 2411 | TTTATATATTTGCAAACATTGAACTCATTTG | 992 | AACTCTATATTTCATATATTTGCAAACATTG |
| 2405 | TATTTGCAAATATATGAACAAATGAGTTCAA | 965 | TTTGCAAATATTGAACTCCATATTTCATATA |
| 2247 | ATGTTTACGTTT | 943 | TTCATATTTTGCAAACATTGAACTCATTTG |
| 2219 | ATATTTTTGCAAATATTGAACTCATTTGTTC | 943 | AGGATTG |
| 2207 | AACTCATTTTTCATATTTTTACAAATACGA | 930 | TTCATATATTTACAAACATGAACTCATTTG |
| 2196 | TTCATATTTTTGCAAACATTGAACTCATTT | 927 | GAGTTTCGTATTTGTAAAATATGAACAAAT |
| 2146 | ATATGAAATATGAGTTTTGTATTTGTAAAA | 926 | ATATTTTTGCAAACATTGAACTCATTTGTTT |
| 2136 | ATCAC | 921 | TTCATATATTTACAAACATTGAACTTATTTG |
| 2120 | TTTGCAAACATTGAACTCCATATGTTCATATA | 914 | TTTGCAAAAATATGAAATATGAAGTTTCGTA |
| 2103 | TTAT | 912 | TTCATATATTTGCAAATACGAAACTCCATAT |
| 2035 | TTTGCAAAAATATGAACAAATAAGTTCAATG | 908 | TTTGCAAAAATATGAACAATGAGTTCAATG |
| 2003 | AACTCATTTGTTCATATTTTTACAAAAACAA | 898 | TTCATATATTTGCAAACATGAACTCATTTG |
| 1913 | TACACACA | 888 | TTCATATTTTTACAAAAACAAAACTCCATAT |
| 1843 | ATTGAACTCATTTGTTCATATTTTTACAAAT | 885 | GAGTTTTGTATTTGTAAAAATATGAACAATT |
| 1777 | ATATGAAATATGGAGTTTTGTATTTGTAAAA | 860 | AAACTCCATATTTCATATATTTGCAAACACG |
| 1740 | TTTGCAAACACTGAACTCATTTGTTCATATA | 855 | ATATGAACAAATGAGTTCAATGTTTGCTAAT |
| 1737 | ATATGAACAATGAGTTCAATGTTTGCAAAT | 851 | TGCAAAAGTGTGGATTATACTTTACACTTT |
| 1726 | AATGTTTGCAAATATATGAAAAATGAGTTT | 850 | GTCT |
| 1682 | ATATGAACAAATGAGTTCAATGTTGCAAAT | 826 | TTATATTTTTACAAATACGAAACTCATTTGT |
| 1680 | TTTGCAAAAATATGAAATATGGAGTTTAGTA | 819 | TTTACAAATACGAAACTGCATATTTCATATA |

| 802 | AGAGTAAATTAACGTTGTGTGATGGAAAAAA | 538 | TTTTACGCCCGCATTTTTAC |
| --- | --- | --- | --- |
| 801 | TTTGTAAAAATATGAACAAATGAGTTTAATA | 538 | TTTCGTATTTGTAAAAATATGACAAATGAG |
| 795 | TTCATATATTTGCAAACATTGAATTCATTTG | 536 | GAGCTCTAAAT |
| 780 | TGATCCTTGATCAT | 535 | TTCATATTTTTACAAATATGAAACTCATAT |
| 776 | ATTGAACTCATTTGTTCATATATTTACAAAT | 526 | TTCAATGTTTGCAAATATATGAAATATGGAGT |
| 770 | TTGTTCATATTTTTACAAACATTAAACTCAT | 525 | GAAGAATTGCTTTTC |
| 761 | TGAGGAGTTCAACTCCTTTTT | 525 | TTCATATATTTACAAACAATGAACTCATTTG |
| 755 | ATCACACAACCTAAATTTACTTTTTTTTCC | 524 | TTTTCGGTGAATTG |
| 738 | ATATTTTTACAAATACGAAACTCATATTTCAT | 524 | TGTAAAAATATGAACAAATGAGTTTCAATATT |
| 733 | TGAGTTCAATGTTTGCAAATATATGAAAAT | 523 | TTTGCAAAAATATGAAAAAATGAGTTCAATG |
| 733 | AACTCCAATTTCATATATTTGCAAACATTG | 518 | AACTCCATATTTCATATATTTGCAAACATG |
| 730 | TTTGCAAATATATGAAATATGGAGTTTAATA | 516 | AAATTAACGTTGTGTGATGGGT |
| 723 | TAAATATATGAAATATGGAGTTTTGTATTTG | 516 | TATTTGCAAACATTAAACTCCATATGTTCATA |
| 722 | TGAGTTCAATGTTTGCAAATATATGAAATAT | 513 | AACTCAATTGTTCATATTTTTACAAAAACAA |
| 717 | TTCATATTTTTGCAAACATTGAACTCATATG | 508 | TTCCTAGCCTCG |
| 713 | AACTCCATATTTCATTTATTTGCAAACATTG | 506 | ATATATGAAATATGGAGTTCAATGTTTGTAA |
| 711 | AACTCATATGTTCATATATTTACAAACATTG | 503 | GAGTTTAGTGTTTGCAAATATATGAACAAAT |
| 709 | TCCTGTTG | 503 | TTCATATATTTGCAAACATTGAACACATTTG |
| 708 | CATATTTCATATATTTGCAAACACGAAACT | 497 | CAAACATTGAACTCATTTGTTTATATATTTA |
| 697 | TTTCATATTTTACAAATACGAAACTCCATA | 494 | CATATTTTTACAAATACGAAACTCCATTTTT |
| 697 | GTTCATATTTTTACAAATACGAAACTCCATAT | 492 | CAAACATTGAACTCACTTGTTCATATATTTG |
| 696 | GGGGAAAAAAGTGCC | 489 | TTTGTAAAAATATGAAATATGAGTTTCGTG |
| 694 | TTTCATATATTTGCAAACATTAAACTCCATT | 489 | TATGAAATATGGAGTTTCATATTTGTAAAAA |
| 694 | TTCATATATTTGCAAACATTTAACTCATTTG | 487 | GGAATATAATTTACACTTTTGCAAAAATGT |
| 688 | TTCATATATTTACAAACATTGAACTCATTG | 483 | AACTCACTTGTTCATATTTTTACAAATACGA |
| 679 | AATGTTTGCAAATATATAAAATATGGAGTTT | 478 | TTCATATATTTACAAACATTGAATTCATTTG |
| 675 | TTTGCAAACAATGAACTCATTTGTTCATATA | 476 | GTATTTGTAAAAATATGAACAAATGAGTTCA |
| 667 | TCATATATTTACAAACATTGAACTCATTTG | 472 | ATATGAAATATGAGTTTCATATTTGCAAAA |
| 664 | AACTCATATTTTATATATTTGCAAACATTG | 471 | AAACACA |
| 661 | ATATGAAATATGAGTTCGTATTTGTAAAA | 469 | AAATATGACAAATGAGTTCAATGTTTGCAA |
| 656 | TTTGCAAAAATATGAAATATAGAGTTTCGTA | 468 | TTTGTATAATCGA |
| 651 | TATAAAG | 464 | TTCATATATTTACAAACATTAAACTCAATTG |
| 639 | TAGGGCTAAATTAAGTGAAG | 457 | TTGTTCATATTTTTACAAATACTAAACTCAA |
| 629 | GAGTTTAGTATTTGTAAAAATATGAAAAAT | 455 | AACTCCATATTTCATATATTTGCAAACACTA |
| 627 | AATTTTTTTTCCTGA | 448 | ATTCTTCGAAAGCA |
| 626 | GTATTTGTAAAAATATGAACAAATGAGTTC | 446 | TTAAAAGTGAGAGAAAAAAGTAGGCTAGTGGTCCG |
| 624 | TATTCCAACCGAGCACATTTTCTATATTTTC | 445 | ATTGCAAATATATGAACAAATGAGTTCAATG |
| 623 | TTTGTTCATATTTTTACAAATACGAAACTCCA | 444 | AGTTTAATGTTTGCAAATATATGAAATATGA |
| 615 | GAAATTGATGGGGAATGTGAAT | 442 | TTTGCAAAAATATGAAATATGAGTTCGTA |
| 614 | ATAAAAT | 441 | GAGTTTCGTATTTGTAAAAATATGAAATATT |
| 614 | ATGAACAAATGAGTTCAATGTTTGCAAATAA | 439 | TTTCATATATTTACAAATACGAAACTTCATA |
| 603 | TTCGTGTTTGTAAAAATATGAACAAATGAGT | 436 | TTTGCAAAAATATGAAAAATGAGTTTAATG |
| 601 | TTTGCAAAAATATGAACAATTGAGTTTAATG | 434 | ATATGAAAAATGAGTTCAATATTTGCAAAT |
| 600 | TTCAATGTTTGCAAATATATGAAAAATGAGT | 434 | TACAAACACGAAACTCATATTTCATATATT |
| 599 | TAAACG | 431 | AAAATATGAACAAATGAGTTTAATATTTGCA |
| 590 | TAATTTACACTTTTGCAAAAGTGTGGATTA | 430 | TTTGCAAAAATATGAAATATGAGTTTTGTA |
| 588 | AATATGAACAATTGAGTTCAATGTTTGTAAA | 428 | CGAGCACCTTTTTTATTTTACTAT |
| 587 | TGTATGTGTGTG | 427 | TAGCTGATCTTGCGA |
| 585 | TGTTCATATTTTTACAAATACGAACTCATA | 426 | ATTCGCTCATCTGTCC |
| 580 | AAATGTGGATTATAATTTACACTTTTGCAA | 424 | ATATGAACAAATGAGTTCAATGTTGGCAAAT |
| 577 | GAGTTCAATGTTTGCAAATATATAAAATGTG | 422 | TTC |
| 567 | ATTTTTACAAATACGAAACTCCACATTTCAT | 420 | AAACTCCACATTTCATATATTTGCAAACATT |
| 567 | ATATTTCATATATTTGCAAATATTGAACTC | 419 | AACTCATATTTCATATATTTACAAACATTG |
| 567 | TTTGCAAAAATATGAAATATGAGTTCAATG | 413 | AAATATAAACAAATGAGTTTAATGTTTGC |
| 566 | TTTGCAAAAATATGAACAAATGAGTTAAATG | 413 | ATATGAAAAATGAGTTCAATGTTGCAAAT |
| 560 | AAACTCTATATTTCATATATTTGCAAACATT | 413 | TTTGCAAAAATATGAACAAATGAATTCAATG |
| 558 | AACTCCATATTTCATATTTTTGCAAACATTG | 412 | ACATTTCATATATTTGCAAACATTGAACTC |
| 557 | TTTGCAAAAATATGAAAAATGAGTTTCGTA | 408 | AGTTCAATGTTTGCAAATATATGAAATATGC |
| 546 | TACGTACA | 405 | ATATGAACAAATGAGTTAATGTTTGCAAAT |

| 397 | TTCATATTTTTACAAATACAAAACTCCACAT | 299 | TATTTTTACAAATACAAAACTCATATGTTCA |
| --- | --- | --- | --- |
| 394 | GTATTTGTAAAAATATGAAATGTGAGTTTC | 298 | ATGAACAAATGAGTTCAATTTTTGCAAATAT |
| 392 | AACTCATTTGTTCATATTTTTACAAAAACGA | 297 | TTTGCAAATATATGAAATATGAGTTTCATA |
| 391 | ATTTGCAAAAATATGAAATATAGAGTTTAGT | 297 | GAGTTCAGTGTTTGCAAATATATGAAATATG |
| 389 | AGCCTACTTTCTTCTCTCACTTTTAACGGACCACT | 296 | TTCATAAATTTGCAAACATTGAACTCAATTG |
| 388 | TTCATATATTTACAAATACGAAACTCTATAT | 296 | AAACTCATATTTCATATATTTACAAAAACG |
| 385 | CGATCCA | 296 | TTTCATATATTTGCAAACATTGAACTTATT |
| 381 | AACTTCATATTTCATATATTTGCAAACATTG | 296 | AATGAGTTTAGTATTTGCAAAAATATGAAA |
| 378 | CAAAAATATGAAATATGGAGTTTCATATTTG | 294 | TTTACAAATACGAAACTCATTTTTCATATA |
| 371 | TTCATATATTTGCAAACATTAAACACATTTG | 294 | TATTCCAACCGAGCACATTTTCTATATTTTA |
| 370 | TTTGCAAAAATATGAACAAATGAGTTAATG | 293 | AAACTCAATTTTCATATTTTTACAAATACG |
| 365 | AACTCATTTGTTATATTTTTACAAATACGA | 293 | ATGAGTTTAATGTTTGCAAATATATAAAAT |
| 363 | CAT | 293 | AACTCATATTTCATATTTTTACAAAAACGA |
| 363 | AAAAT | 293 | AAATATAAACAATTGAGTTCAATGTTTGC |
| 363 | TTAGTTTTAAGTCGCG | 292 | TGGCCCGTTAAAAGTAAAGAAATTGTATAC |
| 363 | TTGCAAATATATGAACAAATGAGTTTAATAT | 291 | TTCATATATTTGCAAACATTGAACTCCATT |
| 362 | ATATTTCATATATTTGCAAACATTGAAACTC | 291 | TATTTGTAAAAATATGAAATATGGAGTTCG |
| 361 | TCTAACCGAGCACTAATTATTT | 291 | TTTCATATATTTACAAATATGAAACTCCATA |
| 360 | TTGATCA | 291 | AACTCATTTGTTCATATATTTACAAATACGA |
| 360 | TTACAAACACGAAACTCCATATTTCATATAT | 289 | TTTGCAAATATATGAAATATGGAGTTTCATA |
| 359 | ACTAAACTCATTTGTTCATATATTTACAAAC | 288 | TTCATATATTTGCAAACATTGAACTTAATTG |
| 359 | TTCATATATTTGCAAAATTGAACTCATTTG | 285 | AAACTCATTTTTCATATATTTACAAACATT |
| 356 | ATGAAAAATGAGTTTTGTATTTGTAAAAAT | 284 | TTTGCAAAAATATGAAATATAAGTTTCGTA |
| 353 | CATTGAACTCATTTTTTCATATATTTACAAA | 283 | ATATTTTTACAAATACGAAACTCATATTTT |
| 353 | TGGAGTTTAGTATTTGTAAAAATATGAACAAA | 280 | AATGTTTGCAAATATATGAAAAATGGAGTTTC |
| 352 | GAGTTAAATGTTTGCAAATATATGAAATATG | 277 | CCGAGCACTTTTTATTTTCTAA |
| 351 | TGC | 276 | GAGTTCAATGTTTGCAAATTTATGAAATATG |
| 351 | TTCATATATTTGCAAACATAGAACTCATTTG | 273 | TTTGCAAAAATATGAACAATTAAGTTCAATG |
| 343 | TTAG | 273 | AGTTTAGTATTTGTAAAAATATAAACAAATG |
| 341 | AGTTCAATGTTTGCAAATATATAAAAAATG | 272 | ATGAACAAATAAGTTCAATATTTGCAAATAT |
| 340 | AAATATGAACAATTGAGTTCAATGTTTGC | 270 | GTGT |
| 339 | TTTCATATATTTACAAATATGAAACTCATA | 270 | TTCATATTTTTACAAAAACAAAACTCATAT |
| 339 | TGAGTTCAATGTTTGCAAATATATGAAAT | 269 | TAG |
| 338 | GGATCGAGGATTGAGGATCGT | 268 | TAAATTACGTAGTAATGCA |
| 338 | GAACTATAGTT | 267 | AATATATGAAATATGAGTTTAGTGTTTGCA |
| 337 | TTTCATATATTTGCAAACATTAAACTCATTT | 266 | TATTTGCAAAAATATGAACAAATGAGTTCAG |
| 337 | GATTATATTTCACACTTTTGCAAAAGTGTG | 266 | AGTTTCGTATTTGTAAAAATATGAACAAATA |
| 334 | TAAATTA | 264 | TTTCCCCAGGCCCTTT |
| 333 | AACTCATATGTTCATATATTTACAAATACGA | 264 | AACTCATTTGTACATATTTTTACAAATACGA |
| 331 | TATGAAATATGAAGTTTTGTATTTGTAAAAA | 262 | ATATGAAATTGGAGTTTCGTATTTGTAAAA |
| 330 | TTCATATTTTTACAAACACTAAACTCATTTG | 261 | TTACAAATACGAAACTCCACATTTCATATAT |
| 326 | ATATTTGCAAACATTAAACTCATTTGTTC | 260 | AACTCATATTTCATATATTTACAAATACG |
| 324 | ACTTTTACGCCCGCTCCTGTT | 259 | TGTTTGCAAATATATGAAATATGGAGTTT |
| 319 | ATATGAAAATGAGTTCAATGTTTGCAAAT | 258 | CATATTTTTGCAAACATTGAACTCCATATGTT |
| 318 | TTCATATATTTACAAATACGAAACTCAATAT | 257 | TTTGCAAAAATATGAACAAATGAGTTCATG |
| 316 | AAACTCAATATTTCATATATTTGCAAACATT | 256 | TATTTGTAAAAATATGAACATATGAGTTTCA |
| 316 | AGTTCAATGTTTGCAAATATATGAAAAATAG | 256 | ATTTATTA |
| 315 | AAGTTCAATGTTTGCAAATATATGAAAAATG | 256 | TTTGCAAAAATATGAAATATGGAGTTCGTA |
| 314 | AAATATAAACAAATGAGTTCATTGTTTGC | 256 | GTAAATAAGAACTCC |
| 312 | TCGTATTTGCAAAAATATGAACAAATGAGTT | 252 | ATCACACAACCTAAATTTACTTTTTTTTTCC |
| 312 | TTTTGTGTTTGTAAAAATATGAACAAATGAG | 251 | GATCCTCAATCCTCGATTTAC |
| 312 | AAAAAAAGAAATTAAAGTTGTGTGATCAA | 251 | TACG |
| 309 | ATTTCATATATTTGCAAATATTAAACTCAT | 251 | ACAGGTCTA |
| 308 | TTCATATATTTGCAAACATTAAACTCATTG | 250 | AAACTCATTTGTTCATATATTTACAAACACG |
| 307 | CTTAAAGACAG | 248 | AATATGAACAAATGAGTTCAATGTTTGCAAAT |
| 304 | CTCATATGTTCATATATTTACAAACATTAAA | 247 | CCGAGCACATTTTCTATTCAAA |
| 303 | CAAAAATATGAACAAATGAGTTTAGTGTTTG | 247 | TTTTTC |
| 303 | AAACATTGAACTCATTTTTCATATTTTTAC | 246 | AAACTGCATATTTCATATATTTGCAAACATT |
| 302 | GAGTTTTGTATTTGTAAAAATATGACAAAT | 246 | TTCATATATTTGCAAACATTGAAATCATTTG |

| 245 | ATATGACAAATGAGTTTAGTATTTGTAAAA | 199 | TTTGCAAAAATATGAACAAATGAGTTCATTG |
| --- | --- | --- | --- |
| 245 | TTTGCAAAAATATGAAATGTGGAGTTTCGTA | 198 | TTCATATTTTTACAAAAACGAAACTCCATAT |
| 244 | TTCATATATTTACAAAAATTGAACTCATTTG | 197 | ATTGT |
| 243 | GAGTTTAGTATTTGCAAAAATATGAACAAAT | 197 | TTCATATATTTGCAAACATCGAACTCATTTG |
| 242 | GAGTTCAATGTTGCAAATATATGAAATATG | 195 | GAACTCATTTGTTTATATTTTTACAAACATT |
| 242 | TTCATATATTTGCAAATACGAAACTTCATAT | 195 | TTCATATTTTTACAAACATCGAACTCATATG |
| 242 | GAGTTTCGTATTTGTAAAATTATGAACAAAT | 195 | TTTGCAAAAATATAAAATATGAGTTTCGTA |
| 241 | AAATATATGAAATATGAGTTTCGTATTTGTA | 194 | TTGCAAATATATGAAATATGGAGTTTCATGT |
| 240 | AACTCAATTGTTCATATTTTACAAATACGA | 194 | ATTTAACTATCGAGCACCTTTTTT |
| 238 | CACACACATA | 194 | ACATTTTTACCACCAT |
| 237 | ATATGAAAAATGGAGTTTTGTATTTGTAAAA | 194 | ATATGAAATGTGGAGTTTAGTATTTGCAAAA |
| 237 | ATATGAACAAATGAGTTCAATGTTTACAAAT | 193 | TTCATATTTTCACAAATACGAAACTCATAT |
| 237 | GAGTTCAATGTTTGCAAATATATAAAATTG | 193 | GAGTTTTGTATTGTAAAAATATGAACAAAT |
| 236 | TTCATATATTTACAAATACGAAACTCCAAT | 193 | AACTCATTTGTTTTAATTTTTACAAATACGA |
| 233 | TTCATATATTTGCAAATATTGAACTCAATTG | 193 | ATATGAACAAATTAGTTCAATGTTTGCAAAT |
| 232 | AAACTCCATATTTCATATATTTGCAAATACT | 193 | CTCATTTGTTCATATTTTTACAAATATCGAA |
| 230 | ATGAGTTTCATGTTTGCAAATATATGAAAT | 192 | AAACTCACATTTCATATATTTGCAAACATT |
| 230 | ACAAAA | 191 | AATTTCATAAATCTTACAGCAATAGTT |
| 230 | TGCTGGA | 191 | GTTGTGTGATCAAAAAAAAGTAGGACTTAAT |
| 229 | TATTTGTAAAAATATGAACAAATGAGTTCA | 191 | TTCATATATTTGCAAACATTGAACTCATTAG |
| 228 | TAATTTAAAAT | 191 | TTTCATATATTTGCAAACATTAAACTTATT |
| 225 | TTTGCAAAAATATGAAATATAGAGTTTTGTA | 190 | TTCATATTTTACAAATACGAAACTGCATAT |
| 224 | AAAAAAAATTTCAGGA | 190 | ATATTTTTACAAACATCGAACTCATTTGTTC |
| 223 | TGTTTGCAAAAATATGAAATATGAGTTTCG | 190 | TCCCAAATGTCGAATGG |
| 223 | TTGCAAATATATGAAATATGGAGTTCATAT | 190 | TATGAAATATGAAGTTTAGTATTTGTAAAAA |
| 222 | AAAGTAACAGCAGCGGGCGTA | 189 | TTGTTCATATTTTTACAAATATTGAACTTAT |
| 222 | TTCATATTTTGCAAACATTGAACTTATTTG | 189 | AAACTCCATATTTCATATATTTGCAAACACA |
| 220 | TTTGCAAAAATATGAAATATGAGTTTAATA | 189 | ATGAAAAAGGAGTTCAATGTTTGCAAATAT |
| 220 | AAATATGAACAAATAAGTTTAATGTTTGCAA | 189 | AAACATTGAACTCACTTGTTCATATATTTAC |
| 219 | TTTCATATATTTGCAAACACTGAACTCATT | 189 | CACATTTTCTATTCCAACCGAG |
| 219 | TTCATATATTTACAAATACAAAACTGCATAT | 189 | GTGTGT |
| 218 | TTTACAAATACGAAACTCATTTTCATATT | 188 | ATAG |
| 218 | TTCATATATTTACAAATACAAAACTCCACAT | 188 | TGTTTGTAAAAATATGAAATATGGAGTTTCG |
| 217 | TTCATATTTTTACAAATACGAAACTCAAT | 187 | CTCATTTGTTCATATATTTACAAACAATTAA |
| 216 | TCATTTTTCATATAATTGCAAACATTGAAC | 187 | ATATGAAATTGGAGTTTAGTATTTGTAAAA |
| 216 | TGAGTTCAATGTTTGCAAATATATGAAAATA | 186 | TTCATATATTTGCAACATTGAACTCAATTG |
| 215 | TTTATT | 186 | ATATGAACAAATGAGTTCAATGTTTGAAAAT |
| 214 | GTTCATATTTTTACAAACACGAAACTCATAT | 186 | TTTGTAAAAATATGAACAAATAAGTTCAATG |
| 213 | GCTTTT | 186 | TATTTGCAAAAATATGAAAAATGAGTTCAA |
| 213 | TTCATATTTTTACAAATACGAAACTTATAT | 186 | AAACTCATTTGTTCATATTTTACAAATATG |
| 213 | TTTGCAAAAATATGAACAAAGGAGTTCAATG | 185 | AAATGAGTTTTGTATTTGTAAAAATATAAAC |
| 213 | TTCATATATTTGCAAATACGAAACTGCATAT | 185 | AGTTTTGTATTTGTAAAAATATGAACAAATA |
| 212 | TAAAAGTAACGATGCGGGCG | 185 | TTTGCAAAAATATGAAAAATGGAGTTTCGTA |
| 211 | ATATGAACAAATGAGTTCAAAGTTTGCAAAT | 184 | AACAAATGAGTTTCATATTTGCAAAAATATG |
| 211 | AAACTCCATATTTCATATTTTTGCAAACATT | 184 | TTCATATATTTACAAACATAGAACTCATTTG |
| 210 | CTTACATTCGCCATCTATTTCC | 183 | TGAAATATGGAGTTTCGTATTTGTAAAATATA |
| 210 | ATATGACAAATGAGTTTAATGTTTGCAAAT | 183 | TTTCGAAGAATTTTCC |
| 209 | GTTCATATTTTTACAAATACAAAACTCCATTT | 183 | TTCATATATTGCAAACATTGAACTCATTTG |
| 207 | TTTGCAAATATATAAACAAATGAGTTTAATG | 182 | ATCAGC |
| 206 | ACAAATGAGTTCAATGTTTGTAAAAATATG | 182 | TTTGCAAATATATGAAATATGGAGTTTTGTA |
| 205 | TTAATTTCTTTTTTTTTTGATCACACAACT | 182 | TCCACATTTTTGCAAAAGTGTAAATTAAAA |
| 204 | CGGATCTCTCTGAACAA | 182 | AACTCATTTGTTCATATTTTTACAAATTCGA |
| 203 | TGGAGTTTAGTATTTGTAAAAATATGAAAAA | 182 | TTCATATTTGTAAAAATATGAAAAATGAGT |
| 203 | TTTCATATATTTGCAAATACGAAACTCATT | 182 | AGTGTGATGTGATAAC |
| 203 | GAACAAATGAAGTTCAATGTTTGCAAATATAT | 180 | TTTACAAACACAAAACTCCATATTTCATATA |
| 202 | ATATGAAATATGGAGTTTTGTTTTGTAAAA | 180 | GATTAAG |
| 201 | AAGCACTAACG | 180 | GTTTGCAAATATATGAAAAATGAGTTCAT |
| 201 | AACTCAATTGTTCATATTTTTACAAATACG | 178 | TTTTTCAATA |
| 201 | ATATGAAATGTGGAGTTTAGTATTTGTAAAA | 178 | ATATGAACAAATGAGTTCAATGTTTGCAATT |

| 176 | AGGAGTTCAACTCCTTATTTG | 152 | TTGCAAATATATGAAATATGAGTTCAGTGT |
| --- | --- | --- | --- |
| 175 | CTTCATATATTTGCAAACATTGAACTCATTT | 152 | TTTCATATATTTGCAAACATGAAACTCATT |
| 175 | TTCATTCACACTCTCCATCAAT | 151 | TTTGCAAATATATGAAATATGGAGTTTCAATA |
| 174 | TTTCATATTTTTGCAAACATTGAACTCCATT | 151 | TAGCACAAACC |
| 174 | AACAAATGGGTTCAATGTTTGCAAATATATG | 151 | CCCGTTAAAAGCAAAGAAATTGTATGCTGG |
| 174 | TTCATATATTTACAAACATTGAACACATTTG | 150 | ATTTTTACAAACACAAAACTCATATTTCAT |
| 174 | TATTTGTTCATATTTTTACAAATACGAAACTC | 150 | TTTGCAAAAATATGAAATATGGAGTTTTGTT |
| 173 | TTCATATATTTACAAATACGAAACTACATAT | 150 | CAAC |
| 173 | TTTCATATATTTGCAAACATTGAACTGCATT | 149 | GCGTTTTTC |
| 172 | AACTCATTTGTTCATATTTTACAAATACAA | 149 | TTCATATATTTGCAAACATTGAAACTCATTTG |
| 172 | GAGTTTCGTATTTGTAAAATATAAACAAAT | 149 | TTCATATTTTTACAAATACAAAACTGCATAT |
| 170 | TATGAACAAGTGAGTTTAATGTTTGCAAATA | 149 | TCATTCACATCCTCCATCAATT |
| 170 | TTTGCAAAAATATGAACAAGTGAGTTCAATG | 148 | AACTCCTTATTTCATATATTTGCAAACATTG |
| 169 | ACTTAAAACTATCGCG | 148 | GTGTGATGAAAAAAAAGGAAATTAACGTT |
| 169 | ATAAACGAAAACAAC | 148 | ATATATTTACAAACATCGAACTCATTTGTTC |
| 169 | GAGTTCAATGTTTGCAAATATATGAACAATG | 148 | TCATATATTTACAAACATTAAACTCATTTG |
| 168 | ATATGAACAATGAGTTCAAAGTTTGCAAAT | 147 | ATTAAA |
| 168 | ATATAAAATATGGAGTTTCGTATTTGTAAAA | 147 | GCAAAAATATGAACAAATGAGTTTCAATATTT |
| 168 | TTCATATTTTTACAAATACGAAACTCCAAAT | 147 | CAAACATTGAACTCCATATGTTCATATATTTA |
| 168 | TTCATATATTTGCAAACAATAAACTCATTTG | 146 | TTTATTTTACCGCACGCAT |
| 167 | TTTTCATATTTGCAAACATTGAACTCAT | 144 | GCAAAAATATGAAATATGCAGTTTAGTATTT |
| 167 | CTCATTTGTTATATATTTGCAAACATTGAA | 143 | GGTGTT |
| 167 | TTTGCAAAAATATGAACAAATCAGTTCAATG | 143 | ATTGAGTTTAATGTTTGCAAATATATGAAA |
| 166 | AACTCCATAATTCATATATTTGCAAACATTG | 143 | TACTTTTAACGGGCCAGCGTATTTCTT |
| 165 | AAAAATATGAACAAATGAGTTCATATTTGC | 142 | TTTGCAAAAATATGAACAATTGAGTTCAATA |
| 164 | AATGAGTTCAATGTTTGCAAATATATGAAAT | 142 | TATGAAATATAGAGTTTTGTATTTGTAAAAA |
| 164 | TCATATATTTGCAAACATTGAACTCCACATGT | 142 | TTCATATTATTGCAAACATTGAACTCATTTG |
| 163 | CACGCA | 142 | AAACTCCAATTTCATATATTTGCAAACATT |
| 162 | ACGGGT | 142 | TACAAATACGAAACTCCATTTTTCATATATT |
| 162 | ATATATTTACAAAAACGAAACTCCATATTTC | 141 | TTTGTAAAAATATGAACAAATGAGTTCAGTG |
| 162 | ATGAAAAAATGAGTTTAATGTTTGCAATTAT | 141 | TTTGTAAAAATATGAACAAATGAGTTCATG |
| 162 | TTCATATATTTACAAACATTAAACTTATTTG | 141 | AAATATTAACAAATGAGTTCATTGTTTGC |
| 161 | TTTGCAAAAATATAAAATATGGAGTTTCGTA | 140 | GCGCCGAGTTGATCC |
| 160 | TTCATATATTTACAAATACTAAACTCATAT | 139 | CTTACATTCACCATCAATTTCT |
| 160 | ATATGAAATATAGAGTTTAGTATTTGTAAAA | 139 | TTTCTTTACTTTTAACGGGCCAGAATACAA |
| 159 | GGTCCGATCGCAGAT | 139 | CAACAG |
| 159 | AACTGGGAACACACACAGCTCAATATAACTCCATG | 138 | TTCAACTCCTTTTTAGAGGAG |
| 159 | AAATATATGAAATATGCAGTTTCGTATTTGTAA | 138 | AACTCCATTTTTCATATATTTACAAATACAA |
| 159 | CATTCGGCATCTATTTCCATTA | 138 | AACTCACTTGTTCATATTTTTACAAATACAA |
| 159 | AACTACATATTTCATATATTTGCAAACATTG | 138 | TATATTTTTACAAATACGAAACTCACATTTCA |
| 158 | TTTGCAAAAATATGAACAAATGATTTCAATG | 137 | TTTTAGT |
| 158 | TTTGTAAAATATGAACAAATGAGTTCAATG | 136 | TTGCAAAAATATGAAATATGGAGTTTCAATAT |
| 158 | TCTTAAA | 136 | ATGTTTTTGTAAAAATATGAACAAATGAGTTCA |
| 157 | TCATTTTTCATATATTTGCAAACAATGAAC | 136 | TTCATATATTTACAAACATGAACTCAATTG |
| 156 | TTTATCC | 136 | ATATATTTACAAACATTGAACTCATTTATTC |
| 156 | GAACTCATATGTTCATATTTTTACAAATATC | 136 | TATTTGTAAAAATATGAACATATGAGTTCAA |
| 156 | AACTGC | 135 | GAGTTTCATATTTGTAAAAATATAACAAAT |
| 156 | ATATGAACAAATGAAATCAATGTTTGCAAAT | 135 | CAAAGGAGTTCAATGTTTGCAAATATATGAA |
| 156 | ACGTCAACTTTTAACGTGAAAATG | 135 | TTTGCAAAAATATGAAATATGAAGTTTAGTA |
| 155 | AACTCCACAATTTATATATTTGCAAACATTG | 134 | ATTTACA |
| 155 | AACTCCATATTTCATATATTTGCAAACATT | 134 | ATATGAAATATGAGTTCAATGTTTGCAATT |
| 155 | TATTTGCAAACATTGAACTCATTTGTTAA | 133 | AACTCATATGTTCATATTTTTACAAACATTG |
| 155 | CTCCATATTTCATATATTTGCAAACATAAAA | 133 | CAATGTTTGCAAATATATGAAAAATAGAGTTT |
| 154 | ATATGAAATATGAAGTTTCGTATTTGAAAAA | 133 | TTCATATTTTTACAAATACGAAACTCCATT |
| 154 | TTCATATATTTACAAACATTAACTCATTTG | 132 | TATATTTTTACAAATACGAAACTCCAATTTCA |
| 153 | GATGGCGAATGTAAGGGAAATT | 132 | TAGCTGTTTCTGACCTGTTTT |
| 153 | AAACTCATTTTTCATATATTTGCAAACACG | 132 | AACTCATATTTCATATATTTGCAAACATG |
| 153 | TCG | 132 | ATGGAGATGTGATGTGAATGAAATTG |
| 153 | GTCGCGGTAGTTTTAA | 132 | TTCATATTTTTACAAATACGAAACTACATAT |

| 131 | TTTGCAAAAATATGAAATATGAGTTTCGT | 114 | ACCTTTTTTAATTTTACTATCGAGC |
| --- | --- | --- | --- |
| 130 | GTTAATTTACTTTTTTTCCATCACACAAC | 114 | CATATATTTGCAAACATTGAACTCATTTGTA |
| 130 | ATATATTTACAAATACGAAACTTTATATTTT | 114 | TTTGCAAAAATATGAACAAATGAGTTTCAATG |
| 130 | ATATGAAATATGAGTTTCGTATTTGAAAAA | 114 | CTGAGCACATTTTCTATTCCAA |
| 129 | AACTCATATTTTATATATTTACAAATACGA | 114 | TTTGCAAAAATATGAACAAATGAGTTCAAAG |
| 129 | TTTGTAAAATATGAACAAATGAGTTTAGTA | 113 | TGTTTGCAAAAATATGAAATATGGAGTTTCG |
| 129 | TTTTTTTCCTGAAA | 113 | TTTGCAAAAATATAAAAAATGAGTTCAATG |
| 128 | AGTTTCGTATTTGTAAAAATATATAAAATATG | 113 | AACTCCATATTTCATATATTTGCAAACTTTG |
| 128 | ACTATTTCTTTACTTTTAACGGGCCAGCGT | 113 | TATTTGTAAAAATATGAAAAATGAGTTCAA |
| 128 | TTTGCAAAAATATGAACAATTGAGTTAAATG | 113 | AACTCACATGTTCATATTTTTACAAATACGA |
| 128 | TTGTTCATATTTTTACAAATACAAAACTCA | 112 | TCATTTGTTCATATTTTTACAAACATGAAAC |
| 127 | TGTTGA | 112 | TTCATATATTTGCAAATACAAAACTCATAT |
| 127 | TTCGATGCAACGCCAA | 112 | TACAAATATAAAACTCCATATTTCATATATT |
| 126 | TTCATATATTTACAAACATTGAACTAATTTG | 112 | TTTGCAAAAATATGAACATATGAGTTTAATG |
| 125 | ATTTGCAAACATTGAACTCATTTATTCATAT | 111 | TTACGTTGTGTGATAGAAAAAAGAGTAAAT |
| 125 | TTTGCAAACATTGAACTCACATGTTCATATA | 111 | ATAATTTTTTT |
| 125 | ATATGAAATATGAAGTTTCGTATTTGTAAA | 111 | AACTCCAAATTTCATATATTTGCAAACATTG |
| 124 | TAAAAGTAAAGAAATAGTAGGCTGGCCCGT | 111 | AAATATATGAAAAATTGAGTTCAATGTTTGC |
| 124 | TTCATATTTTTACAAACATTAAACTCAATTG | 111 | ATATTTTTACAAATACAAAACTCCACATTTTAT |
| 124 | GTATTTGTAAAAATATGAACATATGAGTTCA | 111 | AACTCATTTGTTCATATTTTTACAAACATCGA |
| 123 | CTCCATATTTCATATATTTACAAACATTAAA | 111 | TTCATATATTTACAAACATTGAACTTAATTG |
| 123 | AACTCCATATTTCATATATTTGCAAACAATG | 110 | ATTTACACTTTTGCAAAAATGTAGATTATA |
| 123 | TTCATATATTTGCAAACAATTAACTCATTTG | 110 | TTTGCAAAAATATGAACAAATGAAATCAATG |
| 122 | TTCATATATTTACAAACATTGAAATCATTTG | 110 | TTTGCAAAAATATGAAAAATAAGTTCAATG |
| 122 | AAACTCATATTTCATATATTTACAAATACC | 110 | CTCAAGGGATAGAAGTATTTCTAG |
| 122 | TCAAGTCGGCGCGAGA | 110 | TAGAA |
| 122 | ATTAAAGTTGTGTGATCAAAAAAAAGAA | 109 | CGTCGC |
| 122 | TTTGTAAAAATATGAAATATCAGTTTCGTA | 109 | TTTTCCCCGGCTGT |
| 122 | AAAACTCATTTGTTTATATTTTTACAAAAAC | 109 | TTTGCAAAAATATGAAAATTGAGTTCAATG |
| 122 | TTCATATATTTACAAACAATAAACTCATTTG | 109 | AAAAAAT |
| 121 | GTACAATTTCTTTACTTTTAACGGGCCAGC | 108 | AGAAAATTCCAGAATTCTTAAAGAAGAATTGCT |
| 121 | AACTCCATTGTTCATATTTTTACAAATACGA | 108 | AAAATATATGAAATATGGAGTTCAATGTTTG |
| 120 | AAAAAAC | 108 | TTTTTTCTGAAATTT |
| 120 | ACTCCACATTTTATATATTTGCAAACATTAA | 108 | TTTGCAAAAATATAAACAAATGAGTTTAATG |
| 120 | TTCAATGTTTGCAAATATATGAAAAATGCAGT | 108 | AACTCATTTTTCATATTTTACAAATACGA |
| 120 | ATATGAACAAATAAGTTCAATGTTGCAAAT | 108 | ATATATTTACAAAAATTGAACTCATTTGTTT |
| 119 | TATTTGCAAAAATATGAACATATGAGTTCAA | 108 | AAACTCATTTGTTCATATTTTTACAAATAAG |
| 119 | AAGTGTGAATTATAATCCACACTTTTGCAA | 107 | TCGTATTTGTAAAAATATGAACATATGGAGT |
| 119 | ATATATTTGCAAACATTAAACTCATTTTC | 107 | TCGTTTGTGCT |
| 119 | AACTCCATATTTCATATATTTGAAAACATTG | 107 | AACTCCATTTTTCATATATTTGCAAATATTG |
| 119 | TTTCATATTTTTGCAAACATTAAACTCATA | 107 | AACTCATTTTTTCATATTTTTACAAATACGA |
| 119 | ATGAAATGTGAAGTTTCGTATTTGTAAAAAT | 107 | CAAAAATTGAACTCATTTGTTCATATTTTTA |
| 118 | TACTATTTTTTCACTTTTTACGGGCCAGCC | 107 | TGTTTGCAAATATATAAAATATGGAGTTT |
| 118 | TTCATATTTTTACAAATACAAAACTCACAT | 106 | TTCATATTTTTACAAATACGAAACTTCATATG |
| 118 | TTTGCAAAAATATGAACAAATGGGTTCAATG | 106 | AACTCATTTGTTCATATATTTACAAACATGA |
| 118 | TTCATATATTTGCAAATACGAACTCATAT | 106 | AAAGTGTGGAATATAATTCACACTTTTGCA |
| 118 | TATATTTGCAAACACTGAACTCATTTGTT | 106 | GAATAGAAAAAATAGAAAAAGTGCTCGGTTG |
| 118 | TATATTTGCAAACATTGAACTTATTTGTT | 106 | ATGAGTTCAATGTTTGCAAATATATGAACAAT |
| 117 | TTCATATTTTTACAAATACAAAACTCAAT | 106 | AAACTTTATATTTCATATATTTGCAAACATT |
| 117 | ATTTA | 106 | CAAACTTTGAACTCATTTGTTCATATATTTA |
| 116 | AACTCATATGTTCATATTTTTACAAATACTA | 105 | TTCATATATTTACAAATACGAAACTCAAT |
| 116 | ATATATTTACAAATATTAAACTCATTTGTTC | 105 | TTCATATATTTGCAAACATGAAACTCATTTG |
| 116 | TTTGCAAAAATATGAAATATGGAGTTTGTA | 105 | AAAAATATGAACAAATAAGTTCAGTGTTTGC |
| 115 | CGAAGTAATATCTA | 105 | TTCATATATTTACAAATATTGAACTCATATG |
| 115 | GTATTTGTAAAAATATGAACAAATGAGTTTCA | 104 | TATATTTTTACAAATACAAAACTCATATTTCA |
| 115 | CGTATT | 104 | TATTTCATATTTTTCAAATACGAAACTCA |
| 115 | AACTCATTTTTCATATTTTTACAAAAACAA | 104 | TTTGCAAAAATATGAACAAATGAAGTTCAATG |
| 115 | CTGGGGAAAAAAGGC | 104 | ATAGAAAATGTGCTCGGTTCGA |
| 115 | AACTCAATTGTTCATATTTTTACAAAAACGA | 103 | TATGAACAAATCAGTTCAATGTTTGCAAATA |

| 103 | ACTCACATTTCATATATTTACAAATACGAA | 93 | CACTTTTGCAAAAATGTGGATTATACTTTA |
| --- | --- | --- | --- |
| 103 | AAACTCATATTTCATATATTTACAAACATT | 93 | TTTGCAAAAATATGAAATATGAGTTTCGAA |
| 103 | AACTCCATTTTTCATATATTTACAAACATTG | 93 | ATGAGTTTCAATATTTGCAAATATATGAAAT |
| 103 | GAAACTCCATTTTTCATATATTTGCAAACAC | 93 | TTCATATATTTACAAAAACAAAACTTCATAT |
| 103 | AGTTTCGTATTTGTAAAAATATGAACATATGC | 93 | CATATTTTTACAAACACGAAACTCAATTGTT |
| 102 | TTGTGATAGCTGATC | 92 | ACTATCGAGCACCATTTTTATTTA |
| 102 | TCATATTTTTACAAATTCGAAACTCATATT | 92 | TTTGTAT |
| 102 | TATATTTTTACAAATACGAAACTCCACATTTCA | 92 | TTTGCAAATATATGAACAATTGAGTTAAATG |
| 102 | TTGTAAAAATATGAACAAATGAGTTTCAATGT | 92 | TGGAGTTTCGTATTTGTAAATATATGAACATA |
| 102 | TTTGTTCATATATTTGCAAACATTAAACTCCA | 92 | GCCTCA |
| 101 | GTAAAAATATGAACAATTGAGTTTCAATATTT | 91 | AACTCCATATTTCATTTATTTGCAAACATG |
| 101 | GATGGGGAATGTGTATGAAATT | 91 | AACTCATTTGTTCATATTTTTACAAAAACTA |
| 101 | AAATTATGAACAATGCAGTTTAGTATTTGTA | 91 | AAAAAAAATTTCAG |
| 101 | TTTGCAAAAATATGAAAAAATGAGTTTAATG | 91 | GAACTCAATTGTTCATATTTTTACAAACATC |
| 101 | TTGTCTG | 91 | AAAAATATGAACAAATGAGTTCAATGTTTG |
| 101 | TTCATATATTTACAAATACAAAACTCTATAT | 91 | AGTTTCGTATTTGAAAAAATATGAACAAATG |
| 100 | TTTGCAAAAATATGAAATATGCAGTTCGTA | 90 | ATATGAACAAATGAGTTCAACGTTTGCAAAT |
| 99 | AGTTCAATGTTTGCAAATATATGAACAAATAG | 90 | TTATATA |
| 99 | ACAAAGGACTCTAAGTTAAATCC | 90 | TCAAT |
| 99 | TGGATG | 90 | ATAAGTTCAATATTTGCAAATATATAAAAT |
| 99 | GTATTTGTAAAAATATGAAATATGAGTTCA | 90 | ATTTGTAAAAATATGAAAAAGGAGTTTCGT |
| 99 | TATTTGCAAATATATAAACAAATGAGTTCAA | 90 | AAACTCATATTTCATATATTTACAAACACA |
| 99 | TTTGCAAAAATATGAACAATGAGTTTAATG | 89 | TTTATATATTTGCAAACATGAACTCATTTG |
| 99 | TTTGCAAAAATATGAAATATTGAGTTTCGTA | 89 | TTTGCAAATATATAAACAAATGAGTTCAGTG |
| 99 | CATTCGCTCATCTGG | 89 | AAAACT |
| 99 | TTCATATATTTACAAACATTAAACTCATTG | 89 | TTTGCAAAAATATGAAATATCAGTTTCGTA |
| 99 | AAATATC | 89 | ATATGAACAAAAGAGTTCAATGTTTGCAAAT |
| 98 | CAAAAATTGCTTATCAGCACCTGCAGGAAAAGA | 89 | TTCATATATTTACAAATACGAAACTTATAT |
| 98 | TCAATGTTTGCAAATATATGAAAAATGAAGTT | 89 | AAAGAAATAGTAGGCTGGCCCGTGAAAAGT |
| 98 | GAGTTTTATATTTGTAAAAATATGAACAAAT | 89 | GAGTTCGTATTTGTAAAAATATGAAAAAT |
| 98 | ATATGAACAAATGAGTTTTGTTTTGTAAAA | 89 | AGTTTCGTATTTGTAAAAATAAAACAAATG |
| 98 | TATTTGCAAAAATATGAACAAATAAGTTCAA | 89 | TTCATATATTTGCAAAAACAAAACTCCATAT |
| 97 | AAAATCTAAACTCG | 89 | TGAACAAATAAGTTCAGTGTTTGCAAATATA |
| 97 | CACACACG | 89 | AACTCATTTGTTCATATATTTACAAACACAA |
| 97 | TTTCTAGGTGCAGGTATTGGTACATTG | 88 | AACTCCATATTTCATATATTTGCAAACATCG |
| 97 | TTCATATATTTGCAAACATTGATCTCATTTG | 88 | AACTCATTTGTTAATATTTTTACAAATACGA |
| 96 | TTCATAAATTTGCAAACATTAAACTCATTTG | 88 | TTTGTAAAAATATGAAAAATGGAGTTTCATA |
| 96 | TGTAAAA | 88 | CTTAATGTTGTGTGATCAAAAAAAAGAA |
| 96 | TTGCAAATATATGAAATATGAGTTTAGTAT | 88 | AGTTTAATATTTGTAAAAATATGAAATATGG |
| 96 | TTTGCAAAAATATGAAATATGGAGTTTTGT | 88 | TTTGCAAAAATATGAACAAATAAGCTCAATG |
| 96 | ATTTGCAAAAATATAAACAAATGAGTTCAAT | 87 | AACTCACATGTTCATATTTTTACAAATACAA |
| 95 | TTTGCAAATATATGAACAAATGAGTTTTATG | 87 | TGAACAAATGAGTTCAATGTTTGCTAAAAATA |
| 95 | GAGTTCAATGATTGCAAATATATGAAATATG | 87 | TGTGAGCACAGCGGTGAACGTCCCTTAAAATTG |
| 95 | ATATGAACAATTGAGTTCAGTGTTTGCAAAT | 86 | GCGCCGAGTTGATCG |
| 95 | TTTCCCCGGCCCTTT | 86 | TCATATATTTGCACACATTGAACTCATTTGT |
| 95 | CAAAAATATGAAATATGGAGTTCAATATTTG | 86 | ACTCCATATTTCATATATTTGCAAATACGA |
| 95 | ATGTTTGCAAATATATGAAAAGTGAGTTCA | 86 | AGTTTTGCTTATTTAGTTCAGTAAAGTTAA |
| 94 | AAATAAAA | 86 | AACTCTATATTTCATATATTTGCAAACAATG |
| 94 | TGTGTGAGCACAGCGGTGAACGTCTTTTAAAGT | 86 | AAATATT |
| 94 | TTTG | 85 | TATGAACAAATGAGTTCATATTTGCAAATA |
| 94 | GTACGTATGTAC | 85 | TTTGCAAAAATATGAACAAAAGAGTTCAATG |
| 94 | GAGTTCAATGTTTGCAAAATATGAAATATG | 85 | TTTGCAAAAATATGAACATATGAGTTTCGTA |
| 94 | ATTTCATATATTTGCAAACAATGAACTCAT | 85 | GTTCATATTTTTACAAACACAAAACTCATAT |
| 94 | AATGAGTTTAATGTTTGCAAATATATGAAAT | 85 | TATTTAT |
| 94 | TGAAATATGGAGTTCATATTTGCAAAAATA | 84 | ATATGCAGTTCGTATTTGTAAAAATATGAA |
| 94 | TGAAATATGCAGTTTAGTATTTGTAAAAATA | 84 | CGGATCTCTCTGAGCAA |
| 93 | AAATGAGTTAAATGTTTGCAAATATATGAA | 84 | ATTTATAC |
| 93 | TTTTTGAGGAGTTCAACTCC | 84 | AACTCATGTGTTCATATTTTTACAAATACGA |
| 93 | CATCAC | 84 | CTTCCAGAA |

| 84 | CATATTTCATATATTTGCAAACATTGAAACTG | 73 | TTCATATATTTACAAACACGAACTCATTTG |
| --- | --- | --- | --- |
| 84 | AAATATATGAAAAATGGAGTTTCATATTTGTA | 73 | TTTCATATATTTACAAACACTGAACTCATTT |
| 84 | CATCCAA | 73 | TTTCATATATTTACAAATACGAACTCCATA |
| 83 | TATTTGTAAAAATATGAAAAATGAGTTTAA | 72 | TATGAACAAGTGAGTTCAATGTTTGCAAATT |
| 83 | ATTGTA | 72 | CATATATTTACAAACATTAAACTCATTTTTT |
| 83 | AGTTTAGTATTTGTAAAAATATGAACAAATA | 72 | ATATATTTACAAATATTGAACTCATTTGTTT |
| 83 | AACTCATTTTTCATATTTTTACAAACACAA | 72 | AAACTCCATATTTCATATATTTACAAACACT |
| 83 | TATATTTGCCAACATTGAACTCATTTTTCA | 71 | GTAATGCATAAATTAACTATTGCT |
| 82 | AACTCATATTTCATATTTTACAAATACAA | 71 | GAGTTAAGTATTTGTAAAAATATGAACAAAT |
| 82 | AAAAATATGAACAAATGAGTTCAATGTTGC | 71 | ATTAC |
| 82 | AGACAAAGGAC | 71 | GGAGTTTCGTATTTGTAAAAATATGAACAAA |
| 82 | AAAATATGAACAAATGAAGTTCAATGTTTGC | 71 | AAAATATGAAAAATGAGTTCAATGTTTGC |
| 82 | AAATATATAAAATTGGAGTTTAGTATTTGTAA | 71 | ATGAAATATGGAGTTTCGTATTTGAAAAAAT |
| 82 | AGTTTCGTATTTGTAAAAATATAAAAAATG | 71 | AACTCCATATTTAATATATTTGCAAACATTG |
| 82 | TTTGCAAAAATATGAAATATGGAGTTTCGAA | 71 | TTTGCAAAAATATGAAATGTGGAGTTTTGTA |
| 82 | ATGAGTTAAATGTTTGCAAATATATGAAAT | 71 | ATGAGTTCAATGTTTGCAAATAAATGAAAT |
| 82 | TCGAGCACTTTTTTATTTTACTA | 70 | CAAACATTGAACTTCATTTGTTCATATATTTA |
| 82 | TTGAACTCAATTGTTCATATATTTACAAATA | 70 | TTTGCAAAAATATGACATATGAGTTTCGTA |
| 82 | AAAAATATGAACAAATGAGTTCAATGTTGT | 70 | TTGTAAAAATATGAACAATTGAGTTTAATAT |
| 81 | TAGG | 70 | ATTCTTCGAAGGAAA |
| 81 | TTTGTAAAAATATGAACAATGAGTTCAATG | 70 | AATGTTTGCAAATATATGAAAAATAGAGTTT |
| 81 | ACC | 70 | ATATATTTGCAAAAATTGAACTCATTTGTTT |
| 81 | AGTTTAGTATTTGTAAAAATATGAAAAAATG | 70 | TTCATAATTTTACAAATACGAAACTCATAT |
| 80 | GAGTTCAATGTTTGCAAATATATGAACATATA | 70 | AGTTTCGTATTTGTAAAAATATGAAATAAGG |
| 79 | TTTGCAAAAATATGAAATAAGGAGTTTCGTA | 70 | AGTTCAATGTTTGCAAAAATATGTACAAATG |
| 79 | GAGTTTAGTATTTGTAAAAATATGAACAAT | 70 | ACAAACATGAAACTCATATTTCATATATTT |
| 79 | GAGTTTTGTATTTGCAAAAATATGAAAAAT | 69 | AGTTTAATGTTTGCAAATATATGAAATATA |
| 79 | CAAACATTGAAATCATTTGTTCATATTTTTA | 69 | AAGTTTCATATTTGTAAAATATGAACAAAT |
| 79 | TATTTGCAAATATATAAAATATGAGTTTCG | 69 | ATTTGCAAACATTGAACTTCATATGTTCATAT |
| 79 | ATTTCATATATTTGCAAACATTGAACTCAA | 69 | CAAAACTCATATTTCATATATTTGCAAACA |
| 78 | GAGTTTCGTTTTGTAAAAATATGAACAAAT | 69 | ATTTGAAAACATTGAACTCATTTGTTCATAA |
| 78 | TTTGCAAACATTAAACTCATTTGTTCATAT | 69 | ATTGTTCAAGAA |
| 78 | AAACTCCATATTTCATATATTTACAAACATCG | 69 | TTTGTAAAAATATGAAAAATGAGTTTAATG |
| 78 | TTTGCAAAAATATGAACAAATGAGTTTTGTA | 69 | TTCATATATTTACAAACAATGAACTCAATTG |
| 78 | TGAGTTTAATGTTTGCAATTATATGAAATAT | 68 | AAACTCATATGTTCATATTTTTACAAAAACA |
| 78 | AACTCCATATTTCATATATTTGCAAAATTG | 68 | TTCATATTTTACAAATATGAAACTCCATAT |
| 77 | AAAAATAATAAA | 68 | ATATGAAATATGAGTTTAATATTTGTAAAA |
| 77 | TATTTGTAAAAATATGAACAAATGGAGTTTCA | 67 | AGCCTACTATTTCTTTACTTTTAACGGACC |
| 77 | ATATGAAATATGAGTTCAATATTTGCAAAA | 67 | TCGGCGCAGATCAAC |
| 76 | ATTTTTTATATTTGCAAACATTGAACTC | 67 | TTTGCAAAAATATGAAATATGCAGTTTGTA |
| 76 | TATTAA | 67 | TATATTTGCAAAAATATGAAATATGGAGTTT |
| 76 | TTCATATATTTACAAATACAAACTCATAT | 67 | AAATATATGAAAAATGGAGTTTCGTATTTGTAA |
| 75 | GAGTTTAGTATTTGAAAAAATATGAACAAAT | 67 | TTTGCAAAAATATGAATAAATAAGTTCAATG |
| 75 | TTTGCAAAAATATGACAAATGAGTTTAATG | 67 | TTTAAATTTTCAATAAAATTG |
| 75 | AAATATAAACATATGAGTTCAATGTTTGC | 67 | TTTTTTGATCACACAACCTTAATTTCTTTT |
| 75 | TCATAAATTTGCAAACATTGAACTCATTTG | 66 | TTCATATATTTACAAACAGTGAACTCATTTG |
| 75 | TTTTGCAAAAGTGTAAATTATAATTTACAC | 66 | TCATATTTTTACAAATACGAAACTCATATCT |
| 75 | ATTGAGTTTCATATTTGTAAAAATATGAACA | 66 | AATGAGTTCAATGTTTGCAAAAATATGAAAT |
| 75 | TTTGCAAAAATATGAAATATACAGTTTCGTA | 66 | ATATGAAATATGGAGTTTGTATTTGTAAAA |
| 75 | TTTGCAAAAATATGAAATGTGAGTTTAGTA | 66 | AAAAAAGTAAATTAACGTTGTGTGATGAA |
| 75 | TGCAAAAATATGAACAAATAAATTCAATGTT | 66 | AAATATATGAAAAATGAGTTTAATATTTGC |
| 74 | AGTTTAGTATTTGTAAAAATATGAACATATGG | 65 | TTTGCAAAAATATGAAATATGAATTTCGTA |
| 74 | AACTCCATATTTCATATATTTGCAAACATAG | 65 | CAGTTTCGTATTTGTAAAAATATGAACAAATG |
| 74 | ATATTTGTACAAATACGAAACTGCATATTTCAT | 65 | TATGAAATTGGAGTTTTGTATTTGTAAAAA |
| 74 | CACAAA | 65 | TTTTACAAACACAAAACTCCATATTTCATAT |
| 74 | AGTTCAATGTTTGCAAATATATGAAAAGTAG | 65 | AAAAATATGAACAAATGAGTTAATGTTTGT |
| 74 | TTCATATATTTGCAAACACGAAACTCAATAT | 65 | TTTGCAAAAATATGAAAAATGAGTTTCATA |
| 73 | TTTGCAAATATATGAACAAATGAGTTCCATG | 65 | AACTCCATAGTTCATATATTTGCAAACATTG |
| 73 | TTCATATATTTGCAAACGATGAACTCATTTG | 64 | AACTCCATATTTCATATATTTGCAAAAATTG |

| 64 | AACTCATATGTTCATATTTTTACAAACACTA | 56 | TTTGCAAAAATATGAACAAATTAGTTCAATG |
| --- | --- | --- | --- |
| 64 | ATATGAAAAATTAGTTCAATGTTTGCAAAT | 56 | TGTTTGCAAAAATATGAAATATGGAGTTTAG |
| 64 | AACTCATTTTTCATATTTTTACAAACACGA | 56 | ATATATTTACAAATATTGAACTTATTTGTTC |
| 64 | AAACTCCATTTTCATATATTTGCAAACATT | 56 | AAATATATAAAATGTGGAGTTTCGTATTTGTA |
| 64 | AAACTCATATTTCATATATTTGCAAATATCG | 56 | AAACTCAATTGTTCATATTTTTACAAATAAG |
| 63 | CATATTTCATATATTTGCAAACATCGAACT | 55 | AAAAGACTCTAAGTTAAATCGCA |
| 63 | CAAACATTGAACTCATTTGTTTATATA | 55 | ATATTTTTACAAAAACGAAACTCATATGTTC |
| 63 | TGCAAACACTGAACTCATTTTTTCATATATT | 55 | ATATTTGCAAACATTGAACTCATATGTTC |
| 63 | AAACTCCATTTTTCATATATTTACAAACATT | 55 | ACGCACACAC |
| 63 | ATATGAAAAATGGAGTTTCATATTTGTAAA | 55 | CAAATACAAAACTCCATATTTCATATTTTA |
| 62 | ACTCATTGTTCATATATTTACAAACATGA | 55 | TTCATATATTTACAAATACAAAACTCAAT |
| 62 | GAGTTTCGTATTTGTAAAAATATGAAAAATA | 55 | CATATTTTTACAAATACTAAACTCCATATTA |
| 62 | TTTACAAATACAAAACTCCAATTTCATATA | 55 | AACTCATTTGTACATATTTTTACAAATACTA |
| 62 | TTTCATATATTTGCAAACATGAACTCCATT | 55 | ATAAGTTTCATATTTGCAAAAATATGAAAT |
| 62 | TTTGCAAAAATATGAACAATTGAGTTCAGTG | 55 | ATTTGCAAAAATATGAACAAATGAGTTCGT |
| 62 | TTCATATATTTGCAAACAATGAACTCAATTG | 55 | CAAAAATTACTTATCAGCACCTGCAGGAAAAGA |
| 62 | TGCAAATATATGAAAAATGGAGTTAAATGTT | 55 | TATGAAATGTGAAGTTTAGTATTTGTAAAAA |
| 61 | TTTGCAAAAATATGAACAAATGAGATCAATG | 55 | TATATTTGCAAACATAGAACTCATTTGTT |
| 61 | CTGGGCCAGGTCGGGGC | 54 | AAAAATATGAAAAATGAGTTCAGTATTTGC |
| 61 | AATATTTGCAAATATATGAAATATAGAGTTC | 54 | TCATTTTTCATATATTTACAAACAATGAAC |
| 61 | AACTCCATTTGTTCATATATTTACAAACATTG | 54 | TTTGCAAAAATATGAAATATGAGTTTGTA |
| 61 | ATATGAAATATGAGTTTCGTTTTGTAAAA | 54 | AGTTCAATGTTTGCAAATATATGAAAAAATGG |
| 61 | AACTGCATATTTCATATATTTGCAAACACGA | 54 | CATATATTTGCAAACATTAAACTTCATATGTT |
| 61 | ATATGAAATATGGAGTTTTGTATTGTAAAA | 54 | CGTAATATTG |
| 61 | TGCAAAAATATGAAATATGGAGTTTTGTGTT | 54 | TCATATATTTACAAATACAAAACTTCATATT |
| 61 | AACTCCAAATTTCATATTTTGCAAACATTG | 54 | TAGAGTTTAGTATTTGTAAAAATATGAAAAA |
| 61 | TTTGTAAAAATATGAACATATGGAGTTCAATG | 54 | ATTTGTAAAAATATGAACAATTGAGTTCAAT |
| 61 | TTTGCAAAAATATGAAAAATTAGTTCAATG | 54 | AATGACTCTAAGTTAAAACA |
| 61 | ATATTTTGAAAACATTGAACTCATTTGTTC | 54 | AAATATAACAAATGAGTTCAATGTTTGC |
| 60 | AACTCATATTTCATATATTTACAAACACTA | 54 | GAGTTTAGTATTTGTAAAATTATGAACAAAT |
| 60 | AAACTCCATATGTTCATATTTTTACAAATACA | 54 | TTCATATATTTACAAACATTGAACTCATTAG |
| 60 | ATATATTTGCAAACACTAAACTCATTTTTC | 53 | AAATATATAAAATATGGAGTTTCGTATTTGTAA |
| 60 | CATTAAACTCACTTGTTCATATATTTACAAA | 53 | TTCATATATTTACAAATAATAAACTCATAT |
| 60 | TTTCATATATTTACAAACATTGAACTCAAT | 53 | AAACTCATATTTCATATTTTTACAAATACC |
| 60 | TATAATTCACACTTTTGCAAAAGTGTAAAT | 53 | TTCTCTTCTAAAGTTCTGGAATTTTCTGTGAGT |
| 59 | AACTCAATTTTCATATTTTCACAAATACGA | 53 | TTCATATATTTACAAACATAAAACTCATTTG |
| 59 | TTTGCAAATATATGAACATATGAGTTTCGTA | 53 | ACTTTAAATGACGTTCACCGCTGTGCTCACACA |
| 59 | AAGTAAGGCTAAATTAAGTG | 53 | AACTCATATCTTCATATATTTGCAAACATTG |
| 59 | AGCACAAACG | 53 | TTCATATATTTACAAAAACGAAACTTCATAT |
| 59 | TTTCATATATTTGCAAACATGAAACTCCATT | 53 | GTATTTGTAAAAATATGAAAAATGAGTTCA |
| 59 | GCTGATAAGCAATTTTTGCGGTTTCC | 53 | ATATTTACAAACATTGATTTCATTTGTTCAT |
| 59 | TTTGCAAAAATATGAACAAATGCGTTCAATG | 53 | TTGTCG |
| 58 | AACTCCACATTTCATAAATTTGCAAACATTG | 53 | TTCATAATTTGCAAACATTGAACTCATTTG |
| 58 | TATGAAATTGAGTTTAGTATTTGTAAAAA | 53 | TATGAAATGTGAGTTTAGTATTTGTAAAAA |
| 58 | ATTTGTATATATTTGCAAACATTGAACTC | 53 | AAATATATGAAAAATGAGTTTCATATTTGTA |
| 58 | CAAAAATAACTTATCAGCACCTGCAGGAAAAGA | 53 | TTTGCAAAAATATGAACAATTGAATTCAATG |
| 58 | TTTGCAAATATATGAAAAATGAGTTTCATA | 52 | ATATATTTGCAAACATTAAACTGAATTGTTC |
| 58 | TTCATATATTTGAAAACATTGAACTCAATTG | 52 | TTTGCAAAAATATGAAATATGCAGTTTCATA |
| 58 | TTCATATATTTACAAATACGAAACTCCATT | 52 | AACTCAATTGTCATATTTTTACAAATACGA |
| 58 | TTTGCAAAAATATGAAAAATGAGTTCAGTG | 52 | GAACTCATTGTTCATATTTTTACAAATAC |
| 57 | TTTGCAAAAATATGAAACATGGAGTTCGTA | 52 | AACTCATTTGTTCATATATTTACAAATACAA |
| 57 | AGTTTAATGTTTGCAAATATATGAAATATC | 52 | GCAAACATGAACTCATTGTTCATATATTT |
| 57 | TTCATATATTTACAAATACGAAACTCAAAT | 52 | TTTGCAAAAATATGAACTAATGAGTTCAATG |
| 57 | TTCATATATTTGCAAACATGAACTCAATTG | 52 | CAAACATTGAACTCATTTGTACATATATTTA |
| 57 | CATATATTTGCAAACACTAAACTCCATTTTT | 52 | ATATAAAATATGCAGTTTCGTATTTGTAAAA |
| 57 | AAAATATGAACAAATGAGTTCAATTTTTGCA | 52 | TGTTTGCAAATATATGAAAATTGAGTTTCAA |
| 57 | TGTTCATATTTTTACAAACACAAAACTCAAT | 51 | TATATTTTTACACATACGAAACTGCATATTTCA |
| 57 | AGTTTCGTATTTGTAAAAATATGAACAAATGA | 51 | TTCATATATTTACAAACGTTGAACTCATTTG |
| 56 | AATATGAACAAATGAGTTCAATGTTTGAAAA | 51 | TTTGCAAAAATATGAAATGTGAGTTTCGTA |

| 51 | TGTATG | 47 | TTCATATTATTACAAATACGAAACTCCATAT |
| --- | --- | --- | --- |
| 51 | TTGCAAAAATATGAAATATGAGTTTCAATAT | 47 | AACTCCATATTTCATATATTTGCAAACACG |
| 51 | AAATTAACGTTGTGTGATGGAAAAAAAAGG | 47 | AACTCCATATGTTCATATTTTTACAAACACTA |
| 51 | AAACTCACAGAAAATTCCAGAACTTTAGAAGAA | 46 | AAATGAGTTTCATATTTGTAAAATTATGAAC |
| 51 | TTCATATATTTACAAATTCGAAACTCATAT | 46 | AACTCATTGTTCATATTTTTACAAAAACGA |
| 50 | TTTGCAAAAATATGAACAATATGAGTTCAATG | 46 | TTCATATATTTGCAAACATTGAACTCAATTTG |
| 50 | TTTTATATATTTACAAACATTGAACTCATT | 46 | ATACTCAAAAC |
| 50 | TTCATATTTTTACAAAAACAAACTCCATAT | 46 | CATATTTTTACAAATACGAAACTCATATTA |
| 50 | GAGTTTCAAATTTGTAAAAATATGAACAAAT | 46 | ATATAAAATATGGAGTTTAGTATTTGTAAAA |
| 50 | TTCATATTTTTACAAATACGTAACTCATAT | 46 | CAAATACTAAACTCCATATTTCATATATTTA |
| 50 | ATTTTTTCCCCAGGCC | 46 | CATATTTCATATATTTGCAAACAAGAAACTC |
| 50 | AAATAAAATAGA | 46 | AACTCCATATTGCATATATTTGCAAACATTG |
| 50 | TATTTGCAAAAATATGAACAAATGAGTTAAA | 46 | CACAACATTAAGTTCTTTTTTTTTGATCA |
| 50 | GAGTTCAATGTTTGCAAATATATGAAAATATG | 46 | AAATATATAAAATTGGAGTTTCGTATTTGTAA |
| 50 | ATATATTTGCAAACACTAAACTCATATGTTC | 46 | AACTCATTTGTTATATTTTTACAAATACAA |
| 50 | ATATGAACAATGAGTTCAATATTTGCAAAT | 46 | GCTGGCCCGTGAAAAGTAAAGAAATAGTAC |
| 50 | AACTCAATTGTTCATATTTTACAAATACAA | 46 | AACTCATTGTTCATATTTTACAAATACGA |
| 50 | TGTTTGCAAAAATATGAAATATGAGTTTAG | 46 | ATTTGCAAACAATGAACTCATTTGTTCAT |
| 50 | TATAATTATATTTTATAT | 46 | AATGTTTGCAAATATATGAAAAATGAAGTTT |
| 50 | TTCATATATTTGCAAACAATAAACTCATAT | 45 | AACTCTATATGTTCATATATTTACAAACATTG |
| 50 | TTTTTTTTGATCACACAACATTAATTTCT | 45 | TTCATATATTTACAAACAAATAACTCATTTG |
| 49 | ATATGAACAAATGAGTTTCATATTTGCAAAT | 45 | TTTGCAAAAATATGAACAAATAAGTTCAACG |
| 49 | GTATTTGCAAATATATGAACAAATGAGTTCA | 45 | ATTTTTACAAATACAAAACTCCATATTTCATAT |
| 49 | AACTCATTAGTTCATATTTTTACAAATACGA | 45 | ACAACGTTGTGTTACTTTTTTTTTTGATCAC |
| 49 | AATT | 45 | ATATTTGCAAACATTGAACTCACTTGTTC |
| 49 | CAAATATATGAAAAATGGAGTTTAATATTTG | 45 | ATGAACAATGGAGTTCAATGTTTGCAAAAAT |
| 49 | TTCATATTTTTACAAAAACGAAACTTCATAT | 45 | TTCATATTTTGACAACATTGAACTCATTTG |
| 49 | GAGTTTAGTATTTGTAAAAATATGAACAAATA | 45 | GAGTTTCGTATTTGTAAAAATATGAACAATAT |
| 49 | AACTCATTTTTCATATATTTACAAATACAA | 45 | GAGTTAAATGTTTGCAAATATATGAATATG |
| 49 | TGCAAATATATGAAAAATGGAGTTTCGTATT | 45 | TATATTTGCAAACATTAAACTCCAATTTTA |
| 49 | AAACTCATATTTCATATATTTGCAAACATA | 45 | TTTGCAAAAATATGAAAAATTGAGTTCAATG |
| 48 | TTCATATTTTTACAAACATTGAACTTCATTTG | 45 | AACTCAATTGTTTATATTTTTACAAATACGA |
| 48 | GAGTTTGTATTTGTAAAAATATGAACAAAT | 45 | ATATTTTTACAAAAACAAAACTCCATATGTTC |
| 48 | TTTCATATATTTGCAAACATTGAACTACATT | 45 | TTTCGTATTTGTAAAAATATGAAACATGGAG |
| 48 | GTTTGCAAATATATGAAAAGTGAGTTTAAT | 45 | TATATTTGCAAACATTGAAATCATTTGTT |
| 48 | TTCATATTTTTACAAACAATGAACTCATTTG | 45 | TTTTTTTTGATCACACAACATTAGGTCCTAC |
| 48 | CATTTGTTCATATATTTACAAACATTAAACA | 45 | CAAACATTGAACTCATTTGTTCATAAATTTA |
| 48 | TTCATATATTTGCAAACATTGAACTCCAATT | 45 | ATATGACAAATAAGTTTAATGTTTGCAAAT |
| 48 | GAAACTCCATATTTCATATATTTGCAAATATC | 45 | AACTCAATTGTTCATATATTTACAAATACGA |
| 48 | ACATATTTTTCTAACTTTTAACGGACCAGTAGCCT | 45 | AGCATCCC |
| 48 | TTTGCAAAAATATGAAATTGGAGTTTCGTA | 45 | AAATATATGAAATATGCAGTTTCGTATTTGTA |
| 48 | TACAAAAACAAAACTCATTTTTCATATATT | 44 | ATATGAAATATGGAGTTTCGAATTTGTAAAA |
| 48 | TGAGTTCAATGTTTGCAAATATATAAAAT | 44 | TCATTTGTTCATATATTTGCAAACACGAAAC |
| 48 | AAGTTTCATATTTGCAAAAATATGAAATATG | 44 | TTTGCAAAAATATGAAAATGAGTTCAATG |
| 48 | TTTGCAAATATATGAACAAATAAGCTCAATG | 44 | TTTGCAAAAATATGAACAAATGAGTTATATG |
| 48 | AACGGACCAGCGTACAATTTCTTTACTTTT | 44 | AAACTACATATTTCATATATTTGCAAACATT |
| 48 | AGTGACAATTCCCTCCGTG | 44 | TATTTGCAAATATATGAACAAATGAGTTTCG |
| 47 | AACTCCATATGTTCATATTTTTACAAACACGA | 44 | TTTGCAAAAATATGAACAAATGAGTTCAAT |
| 47 | ATATGAACAAATGAGTTCAATGTTTACAAT | 44 | CATATTTTTACAAATACGAAACTCCATATTA |
| 47 | TTTTTTTAT | 44 | TATTTGCAAATATATAAAATATAAGTTTCG |
| 47 | AGTTTCATATTTGTAAAAATATGAACAAATA | 44 | CAAAATATGAACAATTGAGTTCAATGTTTG |
| 47 | AAATGAGTTTAATATTTGCAAAAATATGAA | 44 | GCGCCGACTTGATCC |
| 47 | TTTGCAAAAATATGAAAATGAGTTTCGTA | 44 | CTGGTCCGTTAAAAGTAAAGAAATAGTACG |
| 47 | ATGAACAATTGAGTTTCAATATTTGCAAAAAT | 44 | AGTTTCGTATTTGCAAAAATATAAAATATA |
| 47 | TTTCATATATTTACAAACATTGAACTTATT |
| 47 | TAATTTTGCTTTCAACA |
| 47 | CAAACACGAAACTTCATATTTCATATATTTG |
| 47 | TTCATATATTAGCAAACATTGAACTCATAT |
| 47 | TTTGCAAAAATATGAAATGTGGAGTTTCATA |

| **Bembidion laxatum (#SRR5514452)** | | | |
| --- | --- | --- | --- |
| 214579 | ATATGAACAAATGAGTTCAATGTTTGCAAAT | 3870 | ATGAGTTTAATGTTTGCAAATATATGAAAT |
| 67496 | TTCATATTTTTGCAAACATTGAACTCATTTG | 3855 | TTCATATATTTGCAAACATTGAACACATTTG |
| 66848 | TTTGTAAATATATGAACAAATGAGTTCAATG | 3611 | TTTTTGCAAACATTGAACTCATTTGTTTATA |
| 49637 | TTCATATATTTGCAAACATCGAACTCATTTG | 3585 | TTTGTAAAATATGAACAAATGAGTTTCGTA |
| 43021 | AAACTCATTTGTTCATATTTTTACAAATACG | 3425 | CACTTTTGCAAAAGTGTAAATTATATTCCA |
| 33382 | AACTCATTTGTTCATATTTTTACAAATACTA | 3377 | TTCATATATTTGCAAACATTGAACTCATATG |
| 27953 | TTCATATATTTACAAACATCGAACTCATTTG | 3326 | TTCATATATTTGCAAACATCAAACTCATTTG |
| 27496 | GAGTTTTGTATTTGTAAAAATATGAACAAAT | 3210 | TATTTGCAAAAATATGAAATGTGGAGTTTCG |
| 26042 | ATATTTTTACAAATACGAAACTGCATATTTC | 3103 | TTTGCAAAAATATGAAATATGGAGTTTAGTA |
| 24985 | AATGTTTGCAAATATATGAACAAATGAGTTT | 3101 | TTCATATATTTGCAAACATCGAACTCATAT |
| 24728 | TTTCATATATTTGCAAACATTGAACTCCATA | 3078 | TTTGTAAAAATATGAACAAATGAGTTCGATG |
| 23984 | GAGTTCAATGTTTGCAAATATATGAAAAAT | 3071 | AGAATGTAATGGGAAATGATGG |
| 22563 | GTATTTGTAAAAATATGAAATATGAGTTTC | 3068 | AAACTCCACATTTCATATATTTGCAAACATT |
| 16952 | ATTTCATATATTTGCAAACATTGAACTCCAC | 3047 | AGTGCTCGGTTAGAAAATAATA |
| 14979 | TTGCAAACATCGAACTCATTTGTTCATATTT | 3034 | AAACTCATTTTTCATATATTTGCAAACATT |
| 14551 | AATACGAAACTGCATATTTCATATTTTTGCA | 2929 | CAAATACGAAACTCATATTTCATATATTTG |
| 14094 | ATATTTCATATATTTGCAAACATTGAACTC | 2905 | AATGAGTTCAATGTTTGCAAATATATGACA |
| 13285 | AATACGAAACTCATATTTCATATTTTTGCA | 2858 | CAAATATTAAACTCATTTGTTCATATTTTTA |
| 12442 | TTTGCAAAAATATGAAATATGGAGTTTCGTA | 2799 | ATATATTTACAAATACGAAACTCCACATTTC |
| 11918 | TTTGTAAATATATGAAATATGAGTTTCGTA | 2788 | TTTGCAAAAATATGAAATGTGGAGTTTTGTA |
| 11348 | TATATTTGCAAACATTGAACTCATTTGTT | 2733 | AGTTCAATGTTTGCAAATATATGAACAAATGG |
| 11300 | TTCATATAATTGCAAACATTGAACTCATTTG | 2721 | AAATATATGAACAATTGAGTTCAATGTTTGC |
| 11098 | ATATGAAATATGGAGTTTCGTATTTGTAAAA | 2703 | AACTCATATTTCATATATTTACAAATACAA |
| 9842 | GAGTTTAGTATTTGTAAAAATATGAAAAAT | 2570 | CTCGATC |
| 9700 | TTTGTAAAAATATGAACAAATGAGTTCAATG | 2550 | GAGTTTGTATTTGTAAAAATATGAACAAAT |
| 9551 | AAATATATGAACAAATGAGTTCAATATTTGC | 2536 | GAGTTTTGTATTTGTAAAAATATAAACAAAT |
| 8876 | ATATATTTACAAATACGAAACTGCATATTTC | 2516 | TTTGCAAAAATATGAAATGTGGAGTTTAGTA |
| 8845 | CCATATTTCATATATTTGCAAACATCGAACT | 2511 | TTTGTAAATATATGAACAAATGAGTTCAATA |
| 8808 | TTCATATTTTTACAAATACAAAACTCATAT | 2501 | CTCATTTGTTCATATATTTACAAACACGAA |
| 8452 | ATTGAACTCATTTGTTCATATTTTTACAAAT | 2408 | TTTGCAAAAATATGAAAAATGAGTTCAATG |
| 7265 | AATATGAACAAATGAGTTCAATATTTGCAAA | 2376 | AACTCATATTTCATATTTTTACAAATACTA |
| 7139 | AACTCATTTGTTCATATTTTTACAAATTCGA | 2375 | AACTCCATAATTCATATATTTGCAAACATTG |
| 7075 | AACTCATTTTTCATATTTTTACAAATACGA | 2339 | ATATGAATAAATGAGTTCAATGTTTGCAAAT |
| 6971 | TGCAAACATTAAACTCATTTGTTCATATTTT | 2328 | TTTGCAAAAATATGAAATATGGAGTTTTGTA |
| 6669 | TTCATATATTTGCAAACATGAACTCATTTG | 2274 | TATATTTTACAAATACGAAACTCATATTTCA |
| 6540 | CAATGTTTGCAAATATATGAAAAATGGAGTT | 2248 | GAGTTTTGTATTGTAAAAATATGAACAAAT |
| 6226 | AAATATATGAACAAATGAGTTTAATGTTTGT | 2241 | TTTCGTATTTGTAAAAATATAAACAAATGAG |
| 6206 | ATGAGTTCGATGTTTGCAAATATATGAAAA | 2225 | TGTAAAAATATGAAAAATGAGTTTTGTATT |
| 6147 | TTCATATATTTGCAACATTGAACTCATTTG | 2217 | TTTGCAAAAATATGAACAAATGAGTTCATG |
| 5770 | TAT | 2190 | TTCATATTTTACAAATACGAAACTGCATAT |
| 5592 | TTCATATATTGCAAACATTGAACTCATTTG | 2188 | GCAAATATATGAAATATGCAGTTTCGTATTT |
| 5520 | AGTTTCGTATTTGTAAAAATATGAACATATG | 2160 | TTCATATTTTGCAAACATTGAACTCATTTG |
| 5509 | AAACTCCATATTTCATATATTTGCAAACATT | 2126 | TGAACTCATTTGTTCATATATTTACAAACA |
| 5430 | TTCATATTTTTACAAATACAAAACTCCATAT | 2094 | GAGTTTCGTATTTGCAAAAATATGAACAAAT |
| 5348 | TTCATATTTTTACAAATACTAAACTCCATAT | 2092 | AACTCATTTGTTATATTTTTACAAATACAA |
| 4969 | GAACTCATTTGTTCATATTTTTACAAATACG | 2091 | GAACTCATTTGTTCATATATTTACAAACATA |
| 4904 | AAATATATGAAATATGGAGTTTCGTATTTGTA | 2082 | TTCATATATTTGCAAACATTGAACTCCATATG |
| 4890 | TCATATATTTACAAATACAAAACTCCACATT | 2034 | TTCATATATTTACAAACATCAAACTCATTTG |
| 4814 | AAATATATAAACAAATGAGTTCAATGTTTGC | 2031 | ATGAAATATGCAGTTTAGTATTTGTAAAAAT |
| 4796 | ATATGAAATGTGGAGTTTCGTATTTGTAAAA | 2013 | TGTTCATATTTTTACAAATACGAAACTCCATT |
| 4749 | TTCATATATTTACAAACATTGAACACATTTG | 2012 | TTCATTTATTTGCAAACATTGAACTCATTTG |
| 4616 | GTATTTGTAAAAATATGAACAAATGAGTTC | 2011 | TTTGCAAAAATATGAACAAATAAGTTCAATG |
| 4609 | GAGTTCAATGTTTGCAAATATATGAATTGTG | 2005 | TGGAGTTTTGTATTTGTAAAAATATGAACAAA |
| 4268 | AAATATAAACAAATGAGTTCGATGTTTGC | 1997 | AACTCATTTGTTCATATTTTTACAAACACGA |
| 4144 | AACTCATTTGTTCATATTTTTACAAATACTG | 1984 | TATAATCCACACTTTTGCAAAAGTGTAAAT |
| 4112 | ACTCCATATTTCATATATTTACAAATACGAA | 1971 | GAGTTTAGTATTTGTAAAAATATAACAAAT |
| 4014 | ATATGAACAAATAAGTTCAATGTTTGCAAAT | 1968 | TTTGCAAAAATATGAAATATGGAGTTCCGTA |
| 4013 | TTCATATATTTGCAAACATAGAACTCATTTG | 1960 | ACATTTCATATATTTGCAAACATTGAACTC |

| 1911 | TTCATATTTTACAAATACGAAACTCCATAT | 1209 | TGCAAATATATGAAATATGGAGTTCGTGTT |
| --- | --- | --- | --- |
| 1890 | TTCATATATTTGCAAACACTGAACTCATTTG | 1203 | ATATGAACAAATGAGTTCGATATTTGCAAAT |
| 1885 | TTTTACAAATACGGAACTCCATATTTCATAT | 1198 | TTTGTTTATATATTTACAAACATTGAACTCA |
| 1877 | TTCATATATTTACAAATACAAAACTCCATAT | 1189 | AAACTCCATTTTTCATATTTTTACAAATTCG |
| 1865 | TCAGGAAAAAAAATT | 1185 | ATATTTGCAAACATTGAACTCATTTGTTC |
| 1834 | TTTGCAAATATATGAACAAATGAGTTCATTG | 1181 | TTTGCAAAAATATGAAATATGCAGTTTAGTA |
| 1821 | ATATGAAATGTGGAGTTTAGTATTTGTAAAA | 1175 | TTCATATATTTACAAATTCGAAACTCCACAT |
| 1796 | ATATGAAAAATGGAGTTTCGTATTTGTAAAA | 1169 | CAAACACGAAACTGCATATTTCATATATTTA |
| 1795 | AACTGCATTTGTTCATATTTTTACAAATACGA | 1164 | CATATTTTTACAAATACGAAACTCACATTT |
| 1786 | ATTT | 1148 | ATATGAAATATGAAGTTTCGTATTTGTAAAA |
| 1777 | ATATTTGCAAACATCGAACTCATTTGTTC | 1140 | AACTCATTTGTTCATATTTTTACAAAAACAA |
| 1772 | AAC | 1134 | TTCATATATTTACAAACATTGAACTCAATTG |
| 1730 | ATTTTATATATTTGCAAACATTGAACTCCAT | 1118 | CAATTTTTGCAAATATATGAACAAATGAGTT |
| 1721 | TTCATATATTTGCAACATCGAACTCATTTG | 1112 | CTCATTTGTTCATATATTTACAAACACGAAA |
| 1715 | AGTTCAATGTTTGTAAATATATGAAAAATG | 1105 | ATATGAACAAATGAGTTTAATATTTGCAAAT |
| 1676 | AACTCATATTTCATATTTTACAAATACGA | 1092 | GAGTTCAATGTTTGCAAATATATAAAATGTG |
| 1664 | AAATATAAACAAATGAGTTTAATGTTTGC | 1090 | AAATATGCAGTTTCGTGTTTGTAAAAATATG |
| 1661 | TTTCATATATTTGCAAACATCGAAACTCATT | 1086 | AAATGAGTTCAATATTTGCAAATATATGAA |
| 1657 | ATATGAAAAAATGAGTTCAATGTTTGCAAAT | 1081 | AACTCATATTTTATATTTTTACAAATACGA |
| 1643 | TTCATATATTTGCAAACATCGAACTTATTTG | 1079 | TTTGCAAAAATATGAACAAATAAGTTCGATG |
| 1592 | TGTTTGCAAATATATGAAATATGCAGTTTCG | 1078 | CTTTTGCAAAAGTGTGGATTATACTTTACA |
| 1576 | TAACC | 1077 | AAATATATGAACAAATGAGTTCAATGATTGC |
| 1567 | AACTCATATGTTCATATATTTGCAAACATCG | 1063 | GAGTTTTGTATTTGTAAATATATGAACAAAT |
| 1546 | AGTTTAATGTTTGCAAATATATGAAATATGC | 1060 | TTTGCAAAAATATGAAAAATGAGTTTAGTA |
| 1535 | ATATAAAATATGCAGTTTCGTATTTGTAAAA | 1054 | CATATATTTACAAATACGGAACTCATATTT |
| 1533 | TGTTCATATTTTTACAAATACGAACTCATA | 1049 | AACTCCATATTTCATATATTTGCAAACATG |
| 1525 | CAATATTTGCAAATATATGAAATATGGAGTT | 1043 | TTCATATAATTGCAAACATCGAACTCATTTG |
| 1521 | TATATGAAAAATGGAGTTTCGAATTTGTAAAAA | 1040 | TTTGCAAAAATATGAACAAATGAGTTAAATG |
| 1492 | CACATACA | 1037 | TTTGCAAAAATATGAAATATGAAGTTTCGTA |
| 1477 | TTCATATATTTGCAAATACGAAACTCCATAT | 1019 | AAATATATGAAATGTGGAGTTTCGTATTTGTAA |
| 1458 | TTTGCAAAAATATGAAATGTGGAGTTTCGAA | 1010 | CATTTTTCATATATTTGCAAACATGAACT |
| 1458 | AAAATATGAACAAATGAGTTTAATATTTGCA | 1009 | TTTGCAAAAATATGAACAATTGAGTTCAATG |
| 1444 | CTAACCGAGCACTTTTTATTTT | 994 | ATATTTTTACAAATACGAAACTCATATTTCAT |
| 1444 | ATATATGAACAAATGAGTTTCGTATTTGTAAA | 993 | TTTGCAAATATATGAACTAATGAGTTCAATG |
| 1440 | AACTCATTTGTTCATATTTTTACAAATATGA | 992 | AACTCATTTGTCATATTTTTACAAATACGA |
| 1432 | TCATATATTTGCAAACATTAAACTCATATGT | 985 | CAATATTTGCAAATATATGAAATATGAGTT |
| 1426 | TAAAAATATGAAATGTGGAGTTTCGAATTTG | 980 | TATAA |
| 1413 | AAACTCATTTGTTCATATTTTACAAATACA | 975 | GAGTTTCGTATTTGTAAAAATATGAACAATT |
| 1399 | TATGAACAAATGAATTCAATGTTTGCAAATA | 970 | TTTGCAAAAATATGAAAAATGAGTTTCGTA |
| 1394 | AACTCATTTGTTCATATATTTACAAATACGA | 966 | AAATATATGAAATATGCAGTTTCGTATTTGTAA |
| 1383 | GTTCAATGTTTGCAAATATATGAAAAATGCA | 965 | ATATGAAATATGCAGTTCGTATTTGTAAAA |
| 1382 | TGATGGTGAATGTAAGGGAAAT | 964 | TTTATATTTTTACAAACATTGAACTCATTTG |
| 1369 | TTCATATATTTGCAAACATTGAACTCATTG | 963 | TTCATATATTTGCAAACATTGAACCCATTTG |
| 1358 | GAGTTTAGTATTTGTAAAATATATGAACAAAT | 960 | ATTTGTAAAAATATGAACAAATGAGTTCA |
| 1334 | TATG | 940 | GAAAAATGAGTTTCGAATTTGTAAAAATAT |
| 1328 | TTCATATAATTGCAAACATTAAACTCATTTG | 934 | TTCATATAATTACAAACATTGAACTCATTTG |
| 1322 | TCATTTGTTCATATTTTTACAAACACGAAC | 926 | GAGTTCTGTATTTGTAAAAATATGAACAAAT |
| 1315 | TTCATATATTTACAAATACAAAACTCCACAA | 919 | AATGTTTGCAAATATATGAAAAATGCAGTTTC |
| 1314 | ATATGAAATATGAGTTCGTATTTGTAAAA | 918 | AGTTCGATGTTTGCAAATATATGAAAAATGG |
| 1298 | GAGTTTAGTGTTTGTAAAAATATGAACAAAT | 917 | TTCATATATTTGCAAACACGAAACTCCATAT |
| 1285 | TTTGCAAAAATATGAAATATGAGTTTAGTA | 908 | ATATGAACAAATGAGTTCAATGTTTGCTAAT |
| 1282 | TTTCATATTTTTACAAATACGGAACTCATA | 905 | TTGCAAATATATGAAAAATGAGTTTCAATGT |
| 1282 | AAAAATATGAACAAATGAGTTCGTATTTGC | 901 | GTTTCGTATTTGTAAAAATATAAAAATGA |
| 1279 | ATATGAACAAATGAGTTAAATGTTTGCAAAT | 901 | ATCCACG |
| 1253 | TCATATATTTGCAAACATTGAACTCCACATGT | 899 | GAACTCATATTTCATATATTTACAAATAC |
| 1237 | AACTGCATATGTTCATATTTTTACAAATACGA | 891 | ACCGAGCACTAATTATTTTCTA |
| 1234 | TGAGTTTAGTATTTGTAAAAATATAAACAAA | 883 | TTTGTAAAAATATGAACAAATGAGTTTCAATA |
| 1231 | TTCATATTTTTACAAATACAAAACTGCATAT | 882 | AAACTCCATATTTCATATATTTGCAAACATCG |
| 1224 | ACTCATATGTTCATATTTTTACAAATACAAA | 880 | AACTGCATTTTTCATATTTTTACAAATACGA |

| 868 | AATCCTC | 654 | ATATGAAAAATGAGTTCCGTATTTGTAAAA |
| --- | --- | --- | --- |
| 866 | AAATATATGAAATATGGAGTTTCGTATTTGTAA | 652 | CATATGGAGTTTCGTATTTGTAAAAATATGAA |
| 861 | CAAACACGAAACTCATATTTCATATATTTG | 652 | TTCATATATTTACAAACACTGAACTCATTTG |
| 857 | ATATGAACAAATGATTTCAATGTTTGCAAAT | 650 | TTCATATATTTACAAATACGGAACTCCATAT |
| 852 | ATATGAACAAATGAGTTCAATGTTTTCAAAT | 648 | AAATATATGAAATATGCAGTTTCGTATTTGTA |
| 849 | CGAAACTGCATATTTCATATATTTGCAAACAT | 647 | TAGCCCTACTTCACCTAATT |
| 847 | ACTCATTTGTTCATATTTTGCAAACATCGA | 645 | TACAAATACGAAACTCATTTTTCATATATT |
| 842 | TATGAAATATGAGTTTCGAATTTGTAAAAA | 638 | AGTTCAGTATTTGCAAAAATATGAAATATGG |
| 837 | TTCATATATTTGCAAAATTGAACTCATTTG | 636 | CTTTTTTGAGGAGTTCAACTC |
| 837 | ATATTTTTACAAATATGAAACTGCATATTTC | 635 | CATATATTGCAAACATTAAACTCATTTGTT |
| 836 | AACTCATTTGTTCATATTTTTACAAAAACGA | 635 | TTCATATATTTACAAATACGAACTCCATAT |
| 829 | AACTCCATTTGTTCATATATTTACAAATACAA | 634 | GAGTTTCGTATTTGTAAAAATATAACAAAT |
| 827 | TGAACTCATTTGTTCATATATTTACAAACAA | 631 | AGCCCTACTTCACTTAATTT |
| 818 | ATATGACAAATGAGTTCGATGTTTGCAAAT | 628 | ATCTATTTCCATTACATTTGGC |
| 817 | TTTCATATATTTGCAAACATTAAACTCCATT | 626 | TTTGCAAAAATATGAATATGCAGTTTCGTA |
| 816 | AACTCCACATTTCATATATTTGCAAACATCG | 625 | TTTGCAAAAATATGAACAAATGAGTTCAGTG |
| 810 | ATATGAACAAATGAGTTAATGTTTGCAAAT | 624 | TTTGCAAAAATATGAACAAATGAGTTTCGAA |
| 781 | AATCGAGGATTGAGGATCGTA | 621 | ATATAAACAAATGAGTTCGATGTTTGCAAAT |
| 780 | AAATGAGTTCAATGTTTGCAAATATATAAA | 616 | TTCATATATTGCAAACATCGAACTCATTTG |
| 779 | ATATATTTACAAACATCGAACTCATTTTTC | 616 | AACTCATTTGTTCATTATTTTACAAATACGA |
| 770 | TATGAAATATGGAGTTCGTATTTGTAAAAA | 615 | GGAGTTTTGTATTTGTAAAAATATGAAAAAT |
| 764 | TTCATATTTTTACAAATACAAAACTCACAT | 615 | ATGAGTTCAATGTTTGCAAATATATAAAAT |
| 761 | TATAATTTACACTTTTGCAAAAATGTGGAT | 609 | TTTGCAAATATATGAACAAATGAGTTCAACG |
| 760 | TTCATATATTTACAAACATTGAACTTATTTG | 609 | ATATGAACAAGTGAGTTCAATGTTTGCAAAT |
| 760 | AATTTTTTTTTCCTGA | 607 | TTCATATATTTGCAAACATTGAACTAATTTG |
| 752 | TTCATATTTTACAAACATTGAACTCATTTG | 604 | TGCATATTTCATATATTTGCAAACATCGAAC |
| 747 | AACTCATTTGTTAATATTTTTACAAATACAA | 603 | AACTCATTTGTTCATATTTTTACAAACACAA |
| 745 | TCATATTTTTACAAATTCGAAACTCCATATT | 603 | TTATACT |
| 745 | CGAAACTCATATTTCATATATTTACAAACA | 602 | ATATATTTACAAACATTGAACTCATATGTTC |
| 744 | TTCATATATTTGCAAACATCGAACTCCATATG | 596 | ATGAACAAATAAGTTCATGTTTGCAAATAT |
| 744 | TTTGCAAAAATATGAAATATGAGTTTTGTA | 595 | AAACTCATTTTTCATATATTTGCAAACATC |
| 731 | AAATATGAACTCATTTGTTCATATTTTTAC | 589 | TTTGCAAAAATATGAAATGTGAGTTTCGTA |
| 731 | AACTCATTTGTTCATAATTTTACAAATACGA | 587 | TGTATGTGTGTG |
| 723 | AACTCATTTGTTCATATATTTGCAAACACG | 585 | TTTGCAAAAATATGAAAAATGAGTTCGATG |
| 721 | TTCATATATTTGCAAACTTTGAACTCATTTG | 583 | CAAATGAGTTCAATGTTTACAAATATATGAA |
| 711 | TTTACACTTTTGCAAAAATGTGGAATATAA | 582 | TTCATATATTTACAAATACAAAACTGCATAT |
| 707 | GAACTCATATGTTCATATTTTTACAAACATC | 582 | TTTGTAAAAATATGAAATATGAGTTTCGTG |
| 706 | ATAAA | 579 | ATATGAACAATTGAGTTCGATGTTTGCAAAT |
| 705 | ATATGACAAATGAGTTTAATGTTTGCAAAT | 577 | ATATTTCATATATTTGCAAACATTGAAACTCC |
| 704 | AACTCCATTATTCATATATTTGCAAACATTG | 574 | AAAAATATGAACAAATGAGTTCGATATTTGC |
| 702 | TCATATATTTGCAAACATTGAACTCATTTGC | 573 | TTTGCAAAAATATGAAATATGCAGTTCGTA |
| 700 | TCATATATTTACAAACATTGAACTCATTTG | 570 | GAGCACCTATTCTATTACAACT |
| 697 | ACTCATATTTCATATATTTACAAATACAA | 569 | ATATGAACAAATGAGTTCAATGTTGGCAAAT |
| 696 | TTCATATATTTACAAAAATTGAACTCATTTG | 569 | GGGGAAAAAAGTGCC |
| 694 | AGTTCAATGTTTGTAAATATATGAAATATGG | 566 | CACATTCTCCATCAATTTCATT |
| 693 | GAGTTCAATATTTGCAAATATATGAAATGTG | 563 | AATGTTTGCAAATATATGAAAAATGGAGTTTC |
| 691 | GAGTTTAGTATTTGCAAAAATATGAACAAAT | 561 | TTCATATATTTACAAATATCGAACTCATTTG |
| 685 | GAGTTTAGTATTTGTAAAATATGAACAAAT | 556 | AATTCTTCGAAAAGC |
| 681 | AATATGAACAAATGAGTTCGATATTTGTAAA | 553 | ATGTTTGCAAATATATGAAAAATGGAGTTTCG |
| 681 | GAGTTTAGTATTTGTAAATATATGAACAAAT | 551 | ACAG |
| 680 | TTATATT | 550 | TCATATATTTACAAATACAAAACTCACATT |
| 674 | TTTGCAAATATATGAACAAATCAGTTCAATG | 549 | TGCAAATATATGAAATATGGAGTTCGTATT |
| 668 | TTGTTCATATTTTTACAAATTCGAAACTCCAT | 546 | ATTTCATATATTTGCAAACATAGAACTCCAT |
| 668 | TTTGCAAATATATGAAATGTGGAGTTTTGTA | 542 | TGTTTGCAAAAATATGAAATATGCAGTTTCG |
| 666 | TTTGCAAAAATATGAACAAATGAGTTTGATG | 541 | ATGTTTGCAAATATATGAAAAATGCAGTTTC |
| 664 | CAAATGAGTTCAATGTTTGCAAATTTATGAA | 540 | GAGTTTTGTATTTGTAAAAATATGACAAAT |
| 663 | TTTGCAAAAATATGAAATATGAGTTCGTA | 538 | AATGAGTTCAATGTTTGCAATTATATGAAA |
| 661 | AAATATATGAAATGTGGAGTTTTGTATTTGTAA | 537 | CATAAACA |
| 656 | AAATATATGAACAAATGAGTTCGTATTTGC | 533 | TTCATATATTTACAAACATTGAACTCATTTA |

| 532 | TCGTATTTGCAAAAATATGAAAAATGGAGTT | 438 | TCATATTTTTACAATACAAAACTGCATATT |
| --- | --- | --- | --- |
| 529 | AGCATACAATTTCTTTACTTTTAACGGGCC | 437 | TTCATATATTTGCAAATACGAACTCATAT |
| 528 | TTTGCAAAAATATAAAATATGCAGTTTCGTA | 436 | ATATGAACAAATGAGTTCAATGTTGCAATT |
| 528 | AAATGAGTTCAATGTTTGCAAATATATGA | 435 | TCGTTAGTGCT |
| 527 | CGAAACTCATATTTCATATATTTGCAAACAT | 435 | AATATGAACAAATGAGTTCAATGTTTGCAAAT |
| 520 | TTTGTTCATATTTTTACAAATACTAAACTCCA | 434 | CGTTAGTTTTAAGTCG |
| 520 | TTTGCAAAAATATGAAATATGCAGTTTCATA | 432 | TATTTGTAAAAATATAAACAAATGAGTTCAA |
| 514 | TTCATTATTTGCAAACATTGAACTCATTTG | 428 | ATGTTTGCAAATATATGAAAAATGAGTTTC |
| 514 | AACTCATATGTTCATATTTTTACAAATACGG | 428 | AGGACAAC |
| 512 | ATATTTTTACAAATACAAACTCATATTTC | 427 | TTCATATTTTTACAAATACGAAACTCACATG |
| 512 | ATATGAATTGTGGAGTTTTGTATTTGTAAAA | 427 | TGCAAATACGAAACTCCACATTTCATATATT |
| 511 | TTGCAAATATATGAAAAATGAGTTTCGTAT | 424 | GTGGATA |
| 509 | GTTTGCAAATATATGAAAAATGAGTTTCGT | 422 | TTTGCAAAAATATGAAAAAATGAGTTCAATG |
| 508 | AAACTCACATTTCATATATTTGCAAACATT | 422 | TTTGCAAAAATATGAACAATGAGTTCAATG |
| 507 | GAAACTGCATATTTCATATATTTGCAAACATT | 419 | AACACAA |
| 506 | TTCATATTTTACAAACATCGAACTCATTTG | 417 | TCATATATTTGCAAACATGAACTCATATT |
| 505 | TTTGCAAAAATATGAACAAATGTGTTCAATG | 417 | TTCATATATTTACAAACATTAAACACATTTG |
| 504 | AGATGCCGAATTTAATGGAAAT | 416 | AACTCCAATTTCATATATTTGCAAACATTG |
| 504 | AAATATATGAACAAATGAGTTCCATGTTTGC | 415 | AAATGAGTTTCGTATTTGTCAAAATATGAAC |
| 503 | TTCATATATTTACAAACATTGAACTCATTG | 415 | AAAAATATGACAAATGAGTTCAATGTTTGC |
| 502 | TTCATATATTTGCAAACATTAAACTCAATTG | 414 | ATATGAAATATGCAGTTTCGTATTTGTGAAA |
| 500 | TTCATATATTTACAAACATTGAATTCATTTG | 411 | TACATACG |
| 497 | GTTCAATGTTTGCAAATATATGAAAAATGAA | 410 | TATATTTGCAAACATAGAACTCATTTGTT |
| 497 | TTCATATTTTTACAAATACAAAACTACATAT | 405 | TATATGAAAAATGGAGTTTCGTATTTGTAAAAA |
| 491 | TTCATATATTTACAAATACGAAACTTCATAT | 403 | GTTTGCAAATATATGAAATATGAGTTCGT |
| 490 | GAATGTAAGGGAAATAGATGGT | 401 | ATATATTTGCAAACATTAAACTCCATTTGTTC |
| 490 | TGCAAATATATGAAAAATAGAGTTCAATGTT | 401 | TTTCATATTTTTGCAAACATTGAACTCATA |
| 487 | TTCATTTATTTGCAAACATTAAACTCATTTG | 400 | AGTTTCGAATTTGTTAAAATATGAACAAATG |
| 486 | TTCATATATTTTCAAACATTGAACTCATTTG | 399 | GAGTTTTGAATTTGTAAAAATATGAACAAAT |
| 483 | GAGTTTGATGTTTGCAAATATATGAAATATG | 395 | TTTTCGGTGAATTG |
| 477 | TTTGCAAAAATATGAACAAATGAGTTCTATG | 394 | TGC |
| 477 | ATATGAACAAATGAGTTCATGTTTGCAATT | 393 | TTCATATATTTACAAATACAAAACTACATAT |
| 476 | AATCGATTATACA | 393 | TTTGCAAAAATATGAATAAATGAGTTCAATG |
| 473 | AACTCCACATTTCATTTATTTGCAAACATTG | 392 | ATATGAACAAATAAGTTTAATGTTTGCAAAT |
| 472 | GTATTTGTAAATATATGAACAAATGAGTTC | 392 | TATCGCAAGATCAGC |
| 471 | TTTGTAAAAATATGAACAAATGAGTTTCAA | 392 | TGTTCATATTTTTACAAATATCGAACTCATA |
| 467 | ATTCTTCGAAAGCA | 392 | ATC |
| 467 | TTCATATATTTACAAACACGGAACTCATTTG | 392 | TATTTGCAAACATTGAACTCATTATTTCATA |
| 466 | GAACTCATTTGTTCATATTTTTACAAACACG | 392 | GAGTTTTGTATTTGTAAAATATATGAACAAAT |
| 466 | ACTCATTTGTTCATATATTTACAAACACTAA | 392 | TTCATATATTTACAAAATTGAACTCATTTG |
| 465 | TTTCATATATTTACAAACATTGAACTCATTT | 390 | CAAACATTGAACTCATATTTCATATATTTA |
| 461 | TTTGCAAAAATATGAAATATGAGTTTCATA | 390 | TTTACAAATACGAAACTCATATGTTCATATA |
| 459 | TTTGCAAAAATATGAAATGTGAGTTTTGTA | 388 | TTTGCAAAAATATGAACAAATGAGTTCAAT |
| 459 | AACTCATTTGTTCATATTTTTGCAAATACAA | 388 | AAACTCATTTGTTCATAATTTTACAAATACA |
| 458 | ATATGAAATATGAGTTTAATATTTGCAAAT | 385 | TTCATATTTTTGCAAACATTGAACTCATATG |
| 458 | GAGTTTCGTATTTGAAAAAATATGAACAAAT | 381 | GTTCATATTTTTACAAACACGAAACTCATAT |
| 457 | TCATATATTTGCAAACATGAAACTGCATATT | 376 | TTCATATTTTACAAATACAAAACTCCACAT |
| 457 | AATATATGAAATATGGAGTTTAATATTTGCA | 376 | TGTCCTCTGT |
| 456 | GAAATATGGAGTTCGTATTTGCAAAAATAT | 376 | ACTCATTTGTTCATATATTTACAAATATTAA |
| 455 | AGTTTAGTATTTGTAAAAATATGACAAATG | 371 | TATATTTGCAAACATTGAACTCATTTTT |
| 451 | ATTTCATATTTTTGCAAACATTGAACTCCAT | 371 | AACTCATTGTTCATATTTTTACAAATACGA |
| 450 | TTCATATATTTACAAATACGAAACTCACAT | 370 | ATATGAACAAATGAGTTCAATGTTTACAAT |
| 448 | ATCGAGGATTGAGGATCGTGG | 370 | AAAAATATGAACAAATGAGTTAATGTTTGC |
| 448 | TATTTTTACAAATATGAAACTCATATTTCA | 370 | TAGAATAGGTGCTCGGTTGGAA |
| 445 | TTTGTAAAAATATGAACAAATGAGTTCATG | 369 | GAGCACTTATTCTATTACAACT |
| 444 | ATATGAAAAATGAGTTCAATGTTGCAAAT | 368 | ATTTCATATATTTGCAAACATTGAACTTCAT |
| 444 | TTTGCAAAAATATGAAATATGAGTTCCGTA | 366 | TTCATATATTTACAAAAACAAAACTCCATAT |
| 442 | AAATATATGAAATGTGGAGTTTAGTATTTGTA | 366 | TTCATATATTTACAAACATTGAACCCATTTG |
| 441 | GAGTTCAATGTTTGCAAATAAATGAAATATG | 365 | ATATAAAAAATGAGTTTCGTATTTGTAAAA |

| 363 | AACTCATTTGTTCATATTTTTACAAATCAA | 314 | GTATGGTGGTAAAAAT |
| --- | --- | --- | --- |
| 362 | TTTCATATATTTGCAAACATCGAACTCATTT | 312 | AAATGAGTTCAATGTTTGTAAAAATATGAA |
| 361 | TTTGCAAAAATATAAACAAATGAGTTTAATG | 312 | ATGAACAAATGAGTTCGATGTTTGCAAATAA |
| 360 | TTCATATATTTACAAACATCGAACTCAATTG | 312 | AGTTTCGTGTTTGTAAAAATATGAACAAATGC |
| 357 | TTCATATTTTTACAAAAACGAAACTGCATAT | 312 | AAACTGCATATTTCATATAATTGCAAACATT |
| 355 | TCCCCATCAATTTCATTCACAT | 309 | TTCATTTATTTGCAAACATTGAACTCAATTG |
| 354 | TTTGCAAAAATATGAACAAATGAGTTCATTG | 307 | TTCATATTTTACAAATACAAAACTCCATAT |
| 352 | GAGTTCAATGTTGCAAATATATGAAATATG | 307 | GAACTCATTTGTTCATATTTTTACAAATTC |
| 352 | GAGTTTAGTATTTGTAAAATAATGAACAAAT | 306 | GTACCC |
| 352 | AAACTCCACAATTCATATATTTGCAAACATT | 306 | ACAAATACGAAACTGCATATTTCATATTATT |
| 351 | TTCATATATTTGCAAACATCGAACACATTTG | 305 | TTTGCAAAAATATGAAAAATGCAGTTTCGTA |
| 350 | TATTTGCAAACATCGAACTCATTTGTTCAT | 304 | TTCATATATTTACAAACATTGAACTCATTAG |
| 349 | ACAAACACGAAACTCCATATTTCATATATTT | 303 | TTTGCAAATATATGAAAAATGGAGTTTCGTG |
| 349 | TGCATATTTCATATAATTACAAATACGAAAC | 303 | GAGTTTAGTATTTGTAAAATTATGAACAAAT |
| 349 | AACTCATTGTTCATATTTTTACAAATACAA | 303 | TTTTCATATATTTGCAAACATTGAACTTAT |
| 347 | TTTGTAAAAATATGAAATATGCAGTTTCGAA | 302 | TGAACAAATGAGTTTCAATATTTGCAAAAATA |
| 347 | TTGTAAAAATATGAACAAATGAGTTCAATG | 301 | TGAGTTCAATGTTTGCAAATATATGAAATAT |
| 347 | TTCATATATTTACAAACATCGAACACATTTG | 300 | TATGAACAAATGAGTTCATATTTGCAAATA |
| 347 | AAAATATGAACAAATGAGTTTAATGTTTGC | 299 | AAATATATGAAATATGGAGTTTTGTATTTGTA |
| 347 | AAAAATATGAACAAATGAGTTCATATTTGC | 299 | TTCGTATTTGTGAAAATATGAACAAATGAGT |
| 346 | GAGTTTAATGTTTGCAAATATATAAAATATG | 299 | TTGTTCATATTTTTACAAATACGGAACTCCAT |
| 345 | TTATAAT | 298 | AACTCATATTTCATATTTTTACAAAAACGA |
| 345 | CAAAAATATGAAATATGAGTTTCGTGTTTG | 298 | GTAAAAATATGAACAAATGAGTTTCAAGTTT |
| 343 | TGATG | 297 | GTGTTTGTAAAAATATGAACAAATGAGTTCA |
| 342 | TTTGCAAAAATATGAACTAATGAGTTCAATG | 296 | TTCATATATTTTACAAATACGAAACTCCACAT |
| 341 | GAACTCATATGTTCATATTTTTACAAACACG | 295 | ATATGAAAAATGGAGTTTAGTATTTGTAAAA |
| 340 | GTATTTGTAAAATATGAACAAATGAGTTC | 295 | ATATGAAATGTGGAGTTTCGTATTTGTAAA |
| 340 | GCAAAAATATAAAATATGAGTTTCGTATTT | 295 | TTTGCAAAAATATGAAAAATGAGTTCCGTA |
| 340 | GTATTTGTAAAAATATGAACAAATAAGTTTC | 293 | TAATTTAAAAT |
| 339 | GAAACTGCATATTTCATATATTTACAAACATC | 293 | AACTCAACATTTCATATATTTGCAAACATTG |
| 339 | ATGAGTTTAATGTTTGCAAATATATAAAAT | 291 | TAC |
| 339 | TATGAACAAATGATTTCGATGTTTGCAAATA | 291 | TTTGCAAAAATATGAACAAATGGGTTCAATG |
| 338 | GAGTTCAATGTTTGCAAAAATATGAAATGTG | 291 | TTTGCAAAAATATGAAATATGAGTTCAGTA |
| 337 | GAGTTCAATGTTTGCAAATATATGAAATATA | 290 | ACGATCCTCAATCCACGATTT |
| 336 | AAATATATGAAATGTGGAGTTTCGAATTTGTAA | 289 | TTTGCAAAAATATGAAATATGGAGTTTCATA |
| 335 | ATTTATTA | 289 | TTCATATATTTACAAATACGAACTGCATAT |
| 334 | ATTTGTTCATATTTTTACAAACACGAACTCC | 288 | CAAATACAAAACTCATATTTCATATATTTG |
| 334 | TTTGCAAAAATATGAACAAATGAATTCAATG | 286 | GTATTTGCAAAAATATGAACAAATGAGTTCA |
| 333 | TTTCATATATTTGCAAACATCGAAACTGCATT | 286 | AACTCCATTTTTCATATATTTGCAAATATTG |
| 332 | AACTCCATTTTTCATATATTTACAAACATTG | 285 | TGAGTTCAGTGTTTGCAAATATATGAAAAA |
| 331 | ATATGAAATATGGAGTTCAGTATTTGTAAAA | 283 | TACAAAAACGAAACTCATATTTCATATATT |
| 331 | TTCATATTTTTACAAAAACAAAACTCCATAT | 283 | TCAATATTTGCAAAAATATGAAATATGGAGT |
| 328 | GAACTATAGTT | 282 | TTCATATATTTGCAAACATCTAACTCATTTG |
| 328 | TTCATATATTTACAAAAACAAAACTCCACAT | 281 | AAACTCATATTTCATATATTTGCAAATATCG |
| 328 | GTTGTGTGATCAAAAAAAAAGAAATTAAA | 281 | AAATATAAATAAATGAGTTCAATGTTTGC |
| 326 | TTCATTATTTTACAAATACGAAACTCCATAT | 280 | TTCATATATTTGCAAACTTGAACTCATTTG |
| 326 | TTCATATATTTGCAAACATCGAACTCCATTTG | 280 | AACAAATGAGTTCAATATTTGCAATTATATG |
| 325 | ACATCGAACTCATATGTTCATATATTTACAA | 280 | ATATAAACAAATGAGTTCAATATTTGCAAAA |
| 325 | GCAAATATATGAAATATGGAGTTCATATTT | 280 | AAAAATATGAAAAATGAGTTCAATATTTGC |
| 325 | TTTACACTTTTGCAAAAGTGTAGATTATAA | 280 | TTCATATATTTGCAAACAATAAACTCATTTG |
| 324 | GAGTTTTGTTTTGTAAAAATATGAACAAAT | 279 | CTTTTTACGGGCCAGCCTACTATTTTTTCA |
| 324 | GTTTCATATTTGCAAAAATATGAACAAATGA | 278 | GAGTTTAGTATTTGTAAAAATATGAACATAT |
| 324 | AAATATATGAAATGTGAGTTTCGTATTTGTAA | 278 | CTTTTTTATTTTACTATCGAGCAC |
| 322 | AAACTCATATTTCATATATTTGCAAACATC | 277 | AAACTCCATATTTCATATATTTGCAAACACA |
| 321 | TCATATATTTACAAACATCGAACTCATTTG | 277 | ATTTCATATATTTGCAAACATCAAAACTCCAT |
| 319 | TTCATATATTTGCAAACATTGAACTCCTTTG | 276 | TGTTTGCAAAAATATGAACAAATGATTTCAA |
| 318 | GAGTTTAGTATTTGTAAAAATATGAACAATT | 275 | TTCATATATTTACAAATACAGAACTCCATAT |
| 317 | ATATGAACAAATGAGTTTAGTGTTTGCAAAT | 275 | TTTGTAAAAATATGAAAAATGAGTTCAATA |
| 317 | TTTGCAAAAATATGAAATGTGAGTTTAGTA | 274 | AACTCCACATTTCATATATTTGCAACATTG |

| 272 | ATATGAACAAAAGAGTTCAATGTTTGCAAAT | 235 | TTATATATTTACAAATACGAAACTCATATT |
| --- | --- | --- | --- |
| 271 | AAATATATAACAAATGAGTTCAATGTTTGC | 235 | TTCATATATTTACAAACATCGAACTTATTTG |
| 271 | TTCATATATTTACAAACATTGAAATCATTTG | 235 | AACTCATTTTTCATATATTGCAAACATTG |
| 271 | GCAAAAGTGTAAATTATAATTCACACTTTT | 234 | TTCTAACTGAGCACTTATTATT |
| 271 | TTCATATATTTGCAAACATTAAACACATTTG | 234 | TTCGTGTTTGCAAAAATATGAACAAATGAG |
| 270 | CAAAAATTGAACTCATTTGTTCATATTTTTA | 233 | ATATGAAATATGCAGTTTCGTATTTGTCAAA |
| 269 | AACATTGAACTCATTTGTTCATATATTTAC | 232 | TTTATATATTTACAAATACGAAACTGCATAT |
| 269 | AAATATAAACAAATGAGTTCATTGTTTGC | 232 | ATATGAAATATGAGTTTCGTATTTGTGAAA |
| 269 | TTCATATTTTTACAATACAAAACTCCACAT | 231 | AACTCCACATTTCATATATTTGCAAACATG |
| 267 | TCATATATTTGCAAACATCGAACTACATATT | 231 | ATATAATTGCAAACATTGAACTCATTTGTTT |
| 266 | ATATGAAAAATGAGTTTTGTATTGTAAAA | 230 | TTTGCAAATATATGAAATATGGAGTTTAGTA |
| 266 | CTGAAATTTTTTTTT | 230 | AAACTCATATTTCATATATTTGCAAACATTG |
| 264 | AACTCATTTTTTCATATTTTTACAAATACGA | 229 | TTAATTTCTTTTTTTTTTGATCACACAACT |
| 264 | AGTTCCGTATTTGTAAAAATATAAACAAATG | 229 | GAGTTCAATGTTTGCAAATATATGAATGTG |
| 263 | TCATATATTTACAAACATTAAACTCATATGT | 228 | GAACTCCTTATTTCATATATTTGCAAACATT |
| 263 | AATGAGTTTAATGTTTGCAAAAATATGAAA | 228 | TTCATATATTTACAAATACGAAACTACATAT |
| 263 | TTCATATTTTTACAAATACAAAACTCAAT | 227 | AACTCATATTTCATATATTTACAAATTCGA |
| 263 | ATTTGCAAAAATATGAAATATGAGTTTCGA | 227 | ATATTTGCAAACATTAAACTCATTTGTTC |
| 263 | GAAAAATGGAGTTTCGTATTTGTAAATATAT | 227 | TTCATATATTTGCAAACATCGAACTCATTG |
| 262 | TTCATATTTTTACAAATACGAAACTACATAT | 227 | AAATATGAAATATGAGTTCAGTATTTGTAA |
| 262 | AAAATTTCAGGAAA | 225 | TTCATATATTTACAAACACAAAACTCATTTG |
| 262 | CTGGGGAAAAAAGGGC | 225 | TTCATATATTTACAAACATAAAACTCATTTG |
| 261 | TTCATATATTTGCAAACATGAAACTCATTTG | 225 | AGTTTAGTATTTGAAAAAATATGAACAAATG |
| 261 | AGTTTCGTATTTGTAAAATATAAACAAATG | 225 | TTTGCAAAAATATGAAATATGGAGTTTCGAA |
| 260 | AAACTCCATATGTTCATATATTTGCAAACATT | 225 | TTCATATATTTGTAAACATCGAACTCATTTG |
| 260 | GTAAATAAGAACTCC | 224 | CTTTTGCAAAAGTGTGAATTATAATCCACA |
| 259 | AAACTCCACATTTTATATATTTGCAAACATT | 224 | AGTTTAATGTTTGCAAATATATGAAAAATGC |
| 258 | TTCATAATTTGCAAACATTGAACTCATTTG | 224 | CATATTTCATATATTTACAAACACGAACTC |
| 257 | TATTTCCCTTACATTCGCCATC | 223 | TGTTTGCAAAAATATGAAATATGGAGTTTCG |
| 257 | AAATAAAAAAGGTGCTCGATAGTT | 223 | TTCATATATTTACAAACATTAACTCATTTG |
| 257 | TTCATATATTTACAAAAACGAAACTGCATAT | 223 | TCATATATTTGCAAACATTGAACTACATATT |
| 257 | AAATATATGAAATATGGAGTTTAGTATTTGTA | 222 | GAGTTTCGTATTTTTAAAAATATGAACAAAT |
| 255 | CACACACATA | 222 | ATATATTTACAAACATTGAACTCCATATGTTC |
| 255 | TTCATATATTTGAAAACATCGAACTCATTTG | 222 | AAACTCATTTGTTCATATATTTACAAACATCG |
| 253 | AACTCATTTTTCATATTTTACAAATACGA | 222 | ATATGAAATGTGAGTTTAGTATTTGTAAAA |
| 252 | CTGTCTTTAAG | 222 | AAACTGCATATTTCATATATTTGCAAACATC |
| 252 | AAGACAACGACTAGGTTTTCA | 221 | TTTGCAAAAATATGAACAAATGAGTTCAAAG |
| 251 | ACTCCATATTTCATATATTTGCAAATATGGA | 220 | TATGAACAATGAGTTTAGTATTTGTAAAAA |
| 250 | TCCTTTTCTTCTTACTGGC | 220 | GGATGCT |
| 249 | TATGAACAAATGAGTTCAATATTTGTAAAA | 220 | AACTCCATATTTCATATAATTGCAAACATTG |
| 249 | CGTATTTGTAAAAATATGAAAAATGAGTT | 219 | ACAAAA |
| 249 | ACTAGCCTACTTTTTTCTCTCACTTTTAACGGACC | 219 | AAATATAAACAAATGAGTTTGATGTTTGC |
| 249 | TCT | 219 | TTCATATATTTGCAAACATTGAACTCATTCG |
| 248 | AATGAGTTCTATGTTTGCAAATATATGAAA | 217 | TTTGCAAAAATATGAACAAATCAGTTCAATG |
| 248 | TCATATATTTACAAATACTAAACTCATATT | 217 | ATATGAAATATGCAGTTTCGTATTTGAAAAA |
| 248 | AACAAATGAGTTCCGTATTTGCAAAAATATG | 217 | ATATGAAAAATGGAGTTCCGTATTTGTAAAA |
| 247 | ATATGAACAAATGAATTCGATGTTTGCAAAT | 217 | TTCATATATTTGCAAACATTGAACTCCATCAG |
| 246 | TATATTTGCAAACATGAACTCATTTATT | 216 | ATATGAAATATGGAGTTTTGTATTGTAAAA |
| 246 | GAGTTCGATGTTTGCAAATATATAAAATATG | 216 | ATATATTTACAAACATCGAACTCATTTGTTT |
| 246 | GAGTTTAGTATTTGTAAATATATGAGCAAAT | 215 | TTTGTAAAAATATGAACAAATGAGTTTCGATA |
| 244 | TATTTGCAAATATATGAACAAATGAGTTTCG | 215 | GTATTTGTAAAAATATGAAAAATGAGTTCA |
| 243 | AAACTCCATATTTCATATATTTGCAAACACT | 215 | TTTGTAAATATATGAAATATGGAGTTTAGTA |
| 243 | TTCATATATTTACAAACATTGAACTAATTTG | 214 | TTCATATTTTTACAAATACAGAACTCCATAT |
| 243 | TTTTTACAAACATCGAAACTCATTTGTTCATA | 213 | TTTGCAAAAATATGAAATGTGAGTTTCGAA |
| 239 | AACTCATTTGTTCATATTTTTACAAACACA | 211 | AAACTCCATATTTCATATATTTGCAAACATG |
| 238 | TTCATATATTTACAAACTTTGAACTCATTTG | 211 | AAAATA |
| 236 | AACTCATTTGTTCATATTTTACAAATACGG | 211 | AGTGTAAATTATAATTTACACTTTTGCAAA |
| 236 | TGTG | 211 | AACAAATGAGTTTATGTTTGCAAATATATG |
| 236 | AACTCATTTTCATATTTTTACAAATACGA | 210 | TTCATATTTTTACAAACACGAAACTCATTT |

| 210 | TTTACAAACACGAACTCCATTTGTTCATATA | 189 | TTCATATATTTGCAAACATTGAACTGCATTTG |
| --- | --- | --- | --- |
| 210 | TTTTCATATATTTGCAAACATTGAACTCCA | 189 | TAAAGTTGTGTGAGCACAGCGGTGAACGTCTTT |
| 210 | TTCATATATTTACAAATACAAAACTACACAT | 189 | CATTGAACTCACTTGTTCATATATTTACAAA |
| 209 | TTCATAATTTTACAAATACGAAACTGCATAT | 188 | AACTCATATTTCATATTTTTACAAAAACAA |
| 209 | AAATATATGAAATATGGAGTTTGTATTTGTA | 188 | TTCATATATTTGCTAACATTGAACTCATTTG |
| 208 | TTCGCCATCAATTTCCCTTACA | 187 | TGAAATATGCAGTTTCGTATTTTTAAAAATA |
| 208 | TTCATATATTTACAAAAACGAAACTCCATAT | 186 | GAGTTCAATGTTTGCAATATATGAAATATG |
| 208 | AACTCCATATTTCATATATTTGCAAACAATG | 185 | ATATGAAATATGCAGTTTCGTATTTGAAAA |
| 207 | ATATGAACAAACGAGTTCAATGTTTGCAAAT | 185 | CAAACATTGAACTCATTTGTTCATATTATTA |
| 207 | GAGTTCAATGTTTGCAAATATATGAAAAGTG | 184 | TTCATATATTTGCAAACATTGAACTCATCTG |
| 206 | TCATATTTTTACAAATACAAAACTCCACATGT | 184 | TTGCAAACAATGAACTCATTTGTTCATATAA |
| 206 | AGTTCATGTTTGCAAATATATGAAAAATGC | 184 | TTTATATATTTACAAACATAGAACTCATTTG |
| 206 | TTTCGAAGAATTTTCC | 183 | TTTGCAAAAATATGAACAAATAAGTTTAATG |
| 206 | GAGTTTAGTTTTTGTAAAAATATGAACAAAT | 182 | AACTCATATTTCATATATTTACAAAAACAA |
| 205 | ATTTGTTCATATATTTACAAACATTGAACTCC | 182 | TTTGCAAATATATGAAATGTGGAGTTTAGTA |
| 205 | TTCATATATTTCAAACATTGAACTCATTTG | 182 | AAAAATTTCAGAAA |
| 205 | CAATATTTGCAAATATATGAAATATGCAGTTT | 182 | GAGTTATGTATTTGTAAAAATATGAACAAAT |
| 205 | TTTGCAAATATATGAAATATGGAGTTCCGTA | 182 | AAAAATATTG |
| 204 | GAACTCATTTGTTCATATATTTACAAACATG | 182 | AGGACAGAAAAAAAAGAAA |
| 204 | TGAACAAATGAGTTTCAATGTTTGTAAAAATA | 181 | TTTTTTTTTGATCACATAACCTTAAAATCG |
| 204 | AAAAAAAAGTAAATTTAGGTTGTGTGATGG | 181 | GAGTTTAGTATTTGTAAAAATATGAAAAAAT |
| 203 | TTCATATTTTTACAAATACGAAACTCAAT | 181 | CAACTGAGCACCTATTCCATTA |
| 202 | AAAAGTGCTCAGTTAGAAAATA | 181 | ATATGAATATGCAGTTTCGTATTTGTAAAA |
| 202 | ATATTTGCAAACATCAAACTCATTTGTTC | 180 | TCCATATTTCATATATTTACAAACATCGAAC |
| 202 | TTTCATATATTTACAAACATTAAACTCATT | 180 | GTTTGCAAATATATGAAAAATGCAGTTTCGT |
| 202 | CATTTTTCATATATTTGCAAACATTGAACA | 180 | TTCAATATTTGCAAAAATATGAAATATGCAGT |
| 202 | TTTGCAAAAATATGAACAAGTGAGTTCAATG | 179 | TTTGCAAAAATATGAAATATGCAGTTTTGTA |
| 202 | TTCATATTTTTACAAATACAAAACTCATCAA | 179 | ATTTGCACACATTGAACTCATTTGTTCATAT |
| 202 | AGTTTCGTATTTGTAAAAATATGAACAAATGA | 178 | ATATATTTACAAACATTAAACTCAATTGTTC |
| 201 | ATGTTTGCAAATATATGAACAAATGAGTTTCG | 178 | GAGTTTCGATTTGTAAAAATATGAACAAAT |
| 201 | TGTTTGCAAATATATGAAATGTGGAGTTT | 177 | ATATGAAATATGAGTTCAATGTTTGCAATT |
| 201 | ACAAATATGAAACTCATATTTCATATATTT | 177 | TTCATATTTTACAAATACAAACTCCATAT |
| 201 | TTCATATAATTGTAAACATTGAACTCATTTG | 176 | TTGCAAATATATGAAAAATGAGTTTAATAT |
| 201 | TTCATATTTTTACAAATACAAAACTTCATAT | 176 | AAATATAAACAAATGAGTTCCATGTTTGC |
| 201 | TTTGCAAAAATATGAACAAATGAGTTCAATT | 176 | AGTTTCGTATTTGCAAAAATATGAACAAATGC |
| 200 | GAGTTCGATATTTGCAAATATATGAAATATG | 176 | ATTTCATATATTTGCAAACATCGAACTCAC |
| 200 | TGAAATATGGAGTTTCGTGTTTGTAAAAATA | 175 | GTGCTCGGTTGGCATAGGACAG |
| 200 | ATATGAAATATGCAGTTTCGTATTTGTAAAG | 175 | TTTGCAAAAATATGAACAATTGAGTTTAATG |
| 199 | CATATATTTACAAACATAGAACACATTTGTT | 175 | GAC |
| 198 | ATATGAAATATGGAGTTTCATATTTGTAAAA | 175 | AAAAGTGTGGATTATATTTCACACTTTTGC |
| 198 | AACTCATTTGTTCATATTTTACAAATACA | 175 | ATTTGTAAAAATATGAACAAATGGAGTTCA |
| 198 | TTTGCAAAAATATGAAATATGCAGTTTCGAA | 174 | ATGAACAAATGAGTTCCATATTTGTAAAAAT |
| 198 | ATTCGCTCATCTGTCC | 174 | AACTCATATGTTCATATTTTACAAATACGA |
| 198 | GACCATTCGACATTTGG | 174 | ATATGAAATGTGGAGTTCGTATTTGTAAAA |
| 197 | TTCATATATTTGCAAACATTGAAACTCATTTG | 173 | TGTTCATATTTTTACAAATACGAACTCACA |
| 197 | TTTGCAAAAATATGAACAAATCAGTTCGATG | 172 | AAACTCCACATTTCATATATTTGCAAACATTG |
| 197 | TTCATATTTTTACAAATACAAACTCCATAT | 172 | AGAATATAATTAGTGCTCAGTT |
| 197 | AACTCTACATTTCATATATTTGCAAACATTG | 172 | TTGCAAACATTAAACTCATTTTTTCATATAT |
| 196 | AAAAATATGAACAAATGAGTTCAATGTTGC | 172 | TTCATATTTTTACAAATACGAAACTCCTTAT |
| 196 | TTCATATTTTTACAAAAACGAAACTCCATAT | 171 | GCCGAATGTAATGGAAATAGAT |
| 196 | AAATATATAAAATGTGGAGTTTCGTATTTGTAA | 171 | GAGTTTTCTATTTGTAAAAATATGAACAAAT |
| 195 | TCATATATTTGCAAACATTAAACTCATTGT | 170 | TATTTTTACAAACACGAAACTGCATATGTTCA |
| 195 | TTTGCAAAAATATGAATTGTGGAGTTTTGTA | 170 | ATTTGCAAACACAAAACTCATATTTCATAT |
| 195 | CATATTTCATATATTTACAAACATCGAAACT | 169 | ATATGAACAAATGAAGTTCAATGTTTGCAAAT |
| 194 | TTTGCAAATATATGAAATATGAGTTTCATA | 169 | AACTCATTAGTTCATATTTTTACAAATACAA |
| 193 | ATATTTGCAAACATTGAACTCACATGTTCAT | 169 | TTCATATATTTACAAACATCGAAATCATTTG |
| 192 | AACTCATTTGTTCATTATTTTACAAATACAA | 169 | ATATGAAATATGCAATTTCGTATTTGTAAAA |
| 191 | GAAAATAATTAGTGCTCAGTTA | 168 | GAGTTAAATGTTTGCAAATATATGAAATGTG |
| 191 | CTCATTTGTTCATATATTTGCAAACAGTGAA | 167 | GAGTTTAGTATTTGAAAAATATGAACAAAT |

| 166 | TCCACATTTCATATATTTGCAAACATAGAAC | 148 | TTCATATATTTGCAAACATTGAACACATTAG |
| --- | --- | --- | --- |
| 166 | AACTCATTTGTCATATATTTACAAATACAA | 148 | GAGTTAAATGTTTGCAAATATATGAAATATG |
| 166 | AACTCACAATTCATATATTTGCAAACATTG | 147 | TTCATATATTTGCAAAAATTAAACTCATTTG |
| 166 | TTTGCAAAAATATGAACAAAGGAGTTCAATG | 147 | AATATGAAAAATGGAGTTCAATGTTTGCAAA |
| 165 | CATATTTCATATATTTACAAACATCGAACT | 147 | TGAACAAATGCGTTCAATGTTTGCAAATATA |
| 165 | TTCATATATTTGCAAACATCGAACCCATTTG | 147 | TTCATATTTTTAAAAATACGAAACTCATAT |
| 165 | TTTGCAAAAATATGAACAATTGAGTTCGATG | 146 | TTCGATGCAACGCCAA |
| 165 | AAATATATGAAATATGGAGTTTCGAATTTGTAA | 146 | ATATGAAATATGAGTTTCGTATTTGAAAAA |
| 164 | ATATGAAATATAGAGTTTCGTATTTGTAAAA | 145 | TTTGCAAAAATATGAACAAATGAGTTCCATG |
| 164 | TACACA | 145 | AATATGAACAAATGAGTTTAATGTTTGCAAAT |
| 164 | AAATATATGAAATGTGGAGTTTTGTATTTGTA | 145 | AACTCATATTTCATATATTTACAAACACG |
| 164 | TTCATATAATTGCAAACATAGAACTCATTTG | 145 | TTTGCAAAAATATGAAATATGGAGTTTGTA |
| 163 | TCATATAATTGCAAACATTGAACTCATTTG | 145 | TTGTAAAAATATGAACAAATGAGTTAATGT |
| 163 | TATTTGTAAAAATATGAAAAATGAGTTTCA | 144 | TTTGCAAAAATATGAACAAATGAGTTCAAG |
| 162 | AAATATATGAAAAATGGAGTTTCGTATTTGTA | 144 | TTTTTGATCACACAACATTAAGTCCTACTTT |
| 162 | TTTGCAAAAATATAAACAAATGAGTTCGATG | 144 | AACTCATTTGTTCATATATTTACAAACATGA |
| 162 | GAGTTTCGTATTTGTAAAATATATGAAAAAT | 144 | GGATCTCTCTGAACAAC |
| 161 | ATATTTGCAAACATCAAACTCATATGTTCAT | 143 | TGTTTGCAAATATATGAAAAATGGAGTTTCA |
| 161 | TTTGCAAAAATATGAAAAATGGAGTTTCGAA | 143 | TGCAAACTCGAACTCATTTGTTCATATATT |
| 161 | AGTTCGATGTTTGCAAATATATGAAAAATGC | 143 | TTCATATATTTGCCAACATCGAACTCATTTG |
| 160 | AGTTTAGTATTTGCAAATATATGAAATATG | 143 | ATATGAACAAATCAGTTCGATGTTTGCAAAT |
| 160 | ATTTAGAGCTC | 143 | AACTCATTTGTTAATATTTTTACAAATACGA |
| 159 | TTTCATATATTTGCAAACACGAACTCCATT | 142 | AACGCGAAA |
| 159 | ATATATTTGCAAACATCGAACTCATTTGTTCAT | 142 | AAAATATGAACAAATGAGTTCAACGTTTGCA |
| 158 | AACTCCACATTTCATATATTTACAAACATTG | 142 | TTTGCAAATATATGAACAAATGAGTTCGAAG |
| 158 | TGGTGT | 142 | CAGTTTCGTATTTGTAAAAATATGAACAAAT |
| 158 | ATATATTTACAAATATTGAACTCATTTGTTT | 142 | AACTCCACAATTCATATATTTGCAAAATTG |
| 158 | TATATTTGCAAACATTGAACTTATTTGTT | 142 | TTTGCAAAAATATGAAATATGCAATTTCGTA |
| 158 | TTCATATATTTGCAAAAATCGAACTCATTTG | 142 | AAATATAAACAAATGAGTTCATGTTTGC |
| 157 | AATAAAACTTTAAATTTCT | 142 | ATTCT |
| 157 | TTCATATATTTACAAATTCGAAACTCACAT | 141 | GTATTTGTAAAAATATGAACAAATGAGTTTCA |
| 156 | AACTCCTTATTTGAGGAGTTC | 141 | TTCATATATTTACAAACGTTGAACTCATTTG |
| 156 | AGAT | 141 | GAGTTTAGTATTTGTCAAAATATGAACAAAT |
| 155 | TTTGCAAAAATATAAAAAATGAGTTCAATG | 141 | AATAC |
| 155 | ATTTACAAACACAAACTCATTTGTTCATAT | 141 | GAGTTTAGTATTTGTAAATATATGACAAAT |
| 154 | TTCATATTTTTCAAACATTGAACTCATTTG | 140 | AACTCCATTTTTCATATATTTACAAATACAA |
| 154 | GTGTGT | 140 | GAACTCAATTGTTCATATTTTTACAAATACG |
| 154 | AACTCACTTGTTCATATTTTTACAAATACAA | 140 | GAGTTTAGTATTTGTAAATATATGAAAAAT |
| 154 | AAATATGAACAATGAGTTCGATGTTTGCAA | 140 | TTTGCAAAAATATGAAATTGGAGTTTCGTA |
| 153 | TATTTGTAAAAATATGAAAAATGAGTTTAA | 140 | GAGTTCAATGTTTGAAAATATATGAAATGTG |
| 153 | GTATTTGTAAAAATATGAACAAATGCAGTTC | 140 | ATATATTGCAAACATTGAACTCATTTGTTCAT |
| 153 | TTTGTAAAATATATGAACAAATGAGTTCAATG | 139 | AAATATATGAAATATGAGTTTCAATATTTGC |
| 153 | TTCATATATTTACAAAAACGAAACTCCACAT | 139 | CTCATTTGTTCATATTTTTACAAACATAGAA |
| 153 | TTCATATATTTGCAACATTGAACACATTTG | 139 | TTTGCAAAAATATGAAATATAGAGTTTCGTA |
| 152 | TGTTCATATTTTTACAAATACGAAACTCCACA | 138 | TTCATATATTTACAAATACAAACTCCATAT |
| 152 | TGTTCATATTTTTACAAATACAAAACTCACA | 138 | ATTCATATATTTGCAAACATTAAACTCATTT |
| 151 | TTGAAATTTTTTTTC | 138 | CTTATATTTGCAAACATTGAACTCATTTG |
| 151 | AACTCATTTTTTATATTTTACAAATACGA | 138 | ATGAGTTAAATGTTTGCAAATATATGAAAT |
| 151 | ATATGAAAAATGAGTTTTGTATTTGCAAAA | 138 | CATATATTTACAAACACAAAACTCATATTT |
| 151 | AAATATAAACAATTGAGTTCAATGTTTGC | 137 | ATATAAACAAATGAGTTCAATATTTGCAAAT |
| 150 | AAACTCCATAATTCATATATTTGCAAACATT | 137 | TTATATATTTACAAATACGAAACTCCATATT |
| 150 | TTACTTTTAACGGGCCAGCGTACAATTTCT | 137 | ATATATTTGCAAACATTGAACTCATTTGT |
| 150 | CAAATTATTTTCTAACCGAGCA | 137 | ATATGAACAAATAAGTTCAATGTTTGCAATT |
| 150 | AACATAAACGTA | 137 | TTGCAAAAATATGAAATATGGAGTTTAATAT |
| 149 | GAGTTTTGTATTTGAAAAATATGAACAAAT | 136 | AATGCGTGCGGTAAAATAA |
| 149 | TTTGCAAAAATATGAAATATGAAGTTTTGTA | 136 | CCTTTAT |
| 149 | TTCATATTTTTACAAACATTGAACACATTTG | 136 | ATATATTTGCAAACATAGAACTCATTTGTTT |
| 149 | TTCATATTTTTACAAAAACAAAACTCCACAT | 136 | CAAATATATGAAATATGCAGTTTAATATTTG |
| 149 | TTTGCAAAAATATGAACAAAAGAGTTCAATG | 136 | TTTGCAAAAATATGAACAAATGGAGTTTCGAA |

| 136 | ATATAAACAAATGAGTTCATGTTTGCAAAT | 120 | TTCATATATTTACAAATCAAAACTCCACAT |
| --- | --- | --- | --- |
| 136 | TTTGCAAAAATATGAAATGTGGAGTTTGTA | 120 | TTCATATATTTACAAATACTAAACTCCACAT |
| 135 | AAACTCATATTTCATATATTTGCAAACATA | 120 | TTTGCAAAAATATGAAATGTAGAGTTTTGTA |
| 135 | ATATGAAATGTGAGTTTCGAATTTGTAAAA | 120 | ATTTGTAAAAATATAAAAATGAGTTTAGT |
| 135 | AAACTTCATATTTCATATATTTGCAAACATT | 120 | TCATTTGTTCATATATTTACAAATTCGAAAC |
| 134 | TTTGCAAAAATATGAAAAATGGAGTTTAGTA | 119 | TTTGCAAAAATATAAAATATGGAGTTTCGTA |
| 134 | GCAGTT | 119 | TTGTAAAAATATGAACAAATGAGTTTCATGT |
| 133 | TAAACA | 119 | TATTTGTAAAATATATGAACAAATGAGTTCAA |
| 133 | AGTTTAGTATTTGTAAAAATAAGAACAAATG | 119 | AACTCATTTGTTCTTATTTTTACAAATACAA |
| 133 | ATATGAAATATGCAGTTTCGTATTTGTTAAA | 119 | AAATATGCAGTTTCATATTTGTAAAAATATA |
| 132 | TTTGCAAATATATGAAAAATGCAGTTTCATA | 118 | TTCATATATTTACAAATACAAACTCCACAT |
| 132 | TTTGCAAATATATGAAAAATGGAGTTTCGTA | 118 | TACG |
| 132 | TCATATATTTACAAATACGAAACCCATATT | 118 | TTTGCAAAAATATGAAATATCAGTTTCGTA |
| 132 | AAATGAGTTCAATGTTTGCAAATAAATGAA | 118 | ATATGAAAAATGGAGTTCATGTTTGCAAAT |
| 132 | AAAATATGAACAAATGAGTTCAATATTTGC | 118 | ATTTGCAAAAATATGAACAAATAAGTTCAAT |
| 132 | TTTACAAACACGAAACTTCATATTTCATATA | 118 | TCATATATTTACAAACATTAAACTCATTTG |
| 132 | TTCATATATTTGCAATCATTAAACTCATTTG | 117 | TTTGCAAATATATGAAATATGCAGTTTCATA |
| 132 | ATGTTTGCAAATATATGAAAAATGAAGTTCG | 117 | ATATGAAATATGAGTTTAATATTTGCAAAA |
| 131 | AACTCCAAATTTCATATATTTGCAAACATTG | 117 | TTTGCAAAATATGAACAAATGAGTTCATG |
| 131 | TTTTCATATATTTACAAACATCGAACTCATT | 117 | AAATATAACAAATGAGTTCGATGTTTGC |
| 131 | TTCATATATTTACAAATTCGAAACTCCATAT | 116 | TTCATATATTTACAAAAATTAAACTCATTTG |
| 130 | TCATATATTTACAAACACAAAACTCCACATT | 116 | GAGTTTAGTATTTGTAAAAATATAAAAAAT |
| 130 | AATACTAAACTGCATATTTCATATATTTACA | 116 | AACTCCAATTTTATATATTTGCAAACATTG |
| 130 | TTCATATATTTGCAAACATTGAACTCATTATG | 115 | TGAGTTCAATGTTTGCAAATATATGAAAAT |
| 130 | AAAAATATGAACAAATGGAGTTTCGTATTTGC | 115 | AACTACATTTTTCATATATTTGCAAACATTG |
| 130 | TTTGTAAAATATGAAAAATGAGTTTAGTA | 115 | AAATATATGAAATATGGAGTTTCGTATTTGA |
| 130 | ACACTTTTGCAAAAGTGTGGAATATAATTC | 115 | TTCATATATTTACAAACATCGAACCCATTTG |
| 130 | GAACTCATATTTCATATATTTGCAAATATC | 114 | TTTGCAAATATATGAAATATGCAGTTCAATA |
| 130 | ATATTTTTACAAATACAAAACTCCACATTTTAT | 114 | TGCAAACGTTAAACTCATTTGTTCATATATT |
| 130 | TTCATATATTTGCAAACATAGAACACATTTG | 114 | TGAGTTCAATGTTTGCAAATATATGAAAT |
| 129 | TTTGCAAAAATATGAAATGTGGAGTTTTGAA | 114 | TCCTTCTCTTCTTACTGGC |
| 129 | GAGTTTCGTATTTGAAAAATATGAACAAAT | 114 | GAGTTTCAAATTTGTAAAAATATGAACAAAT |
| 129 | CAAACATTGAACTCAATTGTTCATATTTTTA | 113 | ATTTCATATATTTGCAAACACGAACTCCAC |
| 129 | AACTCCATATTTCATATATTTGCAAACACTG | 113 | AAACTCATATGTTCATATTTTTACAAATAC |
| 129 | TTCATATATTTACAAACAATAAACTCATTTG | 113 | TTCATATTTTTACAAAAACGAAACTACATAT |
| 129 | TTCATAATTTTACAAATACGAAACTCCATAT | 113 | AAACTCATATGTTCATATAATTGCAAACATT |
| 128 | TTTGCAAATATATGAAAAATGAGTTCGTA | 113 | TCATTTGTTCATATTTTTACAAATTTGAAC |
| 128 | AAACTCATTAGTTCATATTTTTACAAATACG | 112 | TTATAATTCACACTTTTGCAAAAGTGTGAA |
| 128 | ACTCATTTTTCATATATTTACAAACACGA | 112 | TTCATATATTTACAAACATCTAACTCATTTG |
| 127 | AAATATAACAAATGAGTTCAATGTTTGC | 112 | TTTGCAAAAATATGAAATATGGAGTTCCATA |
| 127 | AAACTCCATATTTCATATATTTACAAACACA | 112 | GAGTTTAATATTTGCAAATATATGAAATGTG |
| 127 | GAGTTTAGTATTTGTAATAATATGAACAAAT | 112 | AACTCATTCGTTCATATTTTTACAAATACAA |
| 126 | TTCATATATTTGCAAACACGAACTCCATTTG | 111 | TCATATATTTACAAATATTGAACACATTTGT |
| 126 | TTCATATATTTACAAATACGAAACTCCACAA | 111 | AACTCACTTGTTCATATTTTTACAAATACGA |
| 126 | TTGTTCATATATTTACAAACACAGAACTCAT | 111 | CAAACACAAAACTCCACATTTCATATATTTG |
| 126 | AAACTCCATATTTCATTTATTTGCAAACATT | 111 | AACTCATATGTTCATATTTTTACAAAAACGA |
| 126 | TTTGCAAAAATATAAACAAATGAGTTCATG | 110 | TTCATATATTTACAAACATCGAACTCATTAG |
| 126 | AACTCCATATGTTCATATTTTTACAAATACAA | 110 | ATAATTTTTTT |
| 126 | TTCATATATTTACAAACATCGAATTCATTTG | 110 | TTTGCAAAAATATGAAATATGAATTTCGTA |
| 124 | TTCATATATATGCAAACATTGAACTCATTTG | 110 | TTCATATATTTACAAAAATCGAACTCATTTG |
| 124 | CAAAACTCCACATTTCATATATTTGCAAACAT | 109 | GAGTTTGAATTTGTAAAAATATGAACAAAT |
| 124 | TTTGCAAAAATATGAAATATGCAGTTTAATA | 109 | AACTCCACATTTCATATATTTGCAAACAATG |
| 123 | TTTGCAAAAATATGAAATGTGGAGTTCGTA | 109 | TATATAAAATATGCAGTTTCGTATTTGCAAA |
| 123 | TTCATATTTTTACAAAAACGAAACTCCACAT | 109 | TTTGCAAATATATGAACAAATAAGTTCAATA |
| 123 | TTCATATTTTACAAATACGGAACTCATAT | 108 | TTTGCAAAAATATGAACAAATGAGGTCGATG |
| 122 | GAACTCCATATTTCATATATTTGCAAAAATT | 108 | TTCATATATTTACAAATACGAACTCCACAT |
| 122 | AACGATTTTAAAGTTATGTGATCAAAAAAA | 108 | TTTGCAAATATATGAAATATGGAGTTTCATA |
| 122 | TCATATATTAGCAAACATCGAACTCATTTGT | 108 | AAACTCATTTGTTTATATTTTACAAATACA |
| 121 | AACTGCATATTTCATATATTTACAAACATCG | 108 | TTCGTATTTGTAAAAATATGAACAAATGGAG |

| 108 | ATATATTTGCAAACATCGAACTCCACATGTTC | 98 | AACTGCATATTTCATATAATTGCAAACATTG |
| --- | --- | --- | --- |
| 108 | CATATTTTTACAAATACGAAACTTCATATGTT | 98 | TTCATATATTTGCAAACAACGAACTCATTTG |
| 107 | TTCATATATTTACAAATACGAAACTGCATTT | 98 | AAAAATATGAACAAATGAGTTTCAAGTTTGC |
| 107 | TTTGCAAAAATATGAAATATTCAGTTTCGTA | 98 | TTTATATATTTACAAACACGAAACTGCATAT |
| 107 | TTTGCAAAAATATGAACAAATGAATTCGATG | 98 | TTTATATATTTACAAACATTAAACTCATTTG |
| 107 | TTTGCAAAAATATGAACAAATGAGTTAGATG | 98 | TTCATATATTTACAAACATCGAACTAATTTG |
| 106 | TTCATATATTTACAAACACGAAACTCCACAT | 98 | AATTGATGGTGAATGTAAGAGA |
| 106 | TTCATATATTTACAAATACGAAACTTCACAT | 97 | GAGTTTTTATTTGTAAAAATATGAACAAAT |
| 106 | TTGCAAAAATATGAAATATGAGTTTCAATAT | 97 | TCATATTTTTACAAATACAGAACTCATATT |
| 106 | TTTGCAAAAATATGAAATGTGGAGTTTAATA | 97 | TTTGTAAAAATATGAAATATCAGTTTCGTA |
| 106 | GTATTTGTAAAAATATGAAATATGAAGTTTA | 97 | ATTATAATCCACACTTTTGCAAAAGTGTGG |
| 105 | TTTGCAAAAATATGAAATATGGAGTTCTGTA | 97 | TTCATATATTTGCAAACATCGAACTCATT |
| 105 | TGTTTGCAAATATATGAAATATGGAGTTT | 97 | AACTCATTTTTCATATATTTACAAATACAA |
| 105 | TATATTTGCAAACATTAAACTCCACATGTTCA | 97 | TTCATATTTTTACAAATACAAAACTCTACAT |
| 105 | AACTCATATTTCATATATTTGCAAACACTA | 96 | AACTCATTTGTTCATATTTTTACAAACATA |
| 104 | TTCATATATTAGCAAACATTAAACTCATTTG | 96 | CGCGACTTAAAACTAC |
| 104 | TTCGTATTTGCAAAAATATGAACATATGAGT | 96 | TTCATATTTTACAAATACGAACTCCATAT |
| 104 | TTTGTAAAAATATGAACAAATAAGTTCAATG | 96 | ACCATTTTTATTTAACTATCGAGC |
| 104 | AACTCATTTTTTCATATTTTTACAAATACAA | 96 | ATATATTTACAAACACGGAACTCATTTTTC |
| 104 | AACTGCATATTTCATATATTTGCAAACACG | 96 | ATATTTTTACAAATACGAAACGCATATTTC |
| 104 | TTCATATATTTACAAACATCAACTCATTTG | 96 | AACTCATTTGTTCATATATTTGCAAACACGA |
| 104 | AACTCCACATTTCATATATTTGCAAAAATTG | 96 | TGTGAGCACAGCGGTGAACGTCCCTTAAAATTG |
| 104 | AGTTTCGTATTTGTAAAAATATGAAAAATAG | 96 | GTTAAAAGTAAAGAAATAGTACGCTGGCCC |
| 104 | TTCATAATTTTACAAATACGAAACTCATAT | 96 | AACTCATTTGTTTATATTTTTACAAATACA |
| 104 | AAATGAGTTTAGTATTTGCAAATATATGAA | 96 | CTAACCGAGCACTTATAATTTT |
| 103 | TTTGCAAAAATATGAAATATTGAGTTTCGTA | 95 | TTCATTATTTTACAAATACGAAACTCATAT |
| 103 | CTCCACATTTCATATATTTGCAAACATCGAAA | 95 | ATATGAACAAATGAGTTCGATGTTTGCAAATT |
| 103 | AATATATGAAAAATGAGTTTCATATTTGCA | 95 | TATTTGTAAAAATATGAACAAATGGAGTTCAA |
| 103 | TATATTTTTACAAATACGAAACTCATATTTTA | 95 | AAATATATGAAATTGGAGTTTCGTATTTGTAA |
| 103 | GAACTCATTTGTTCATATATTTACAAACAAC | 95 | AACTCATATGTTCATATATTTGCAAACATAG |
| 103 | GAACTCATTTGTTCATATATTTACAAATACG | 94 | TGGTCACACAACGTTAATTTACTTTTTTTT |
| 102 | TTCATATATTTACAAATACAAAACTTCATAT | 94 | TTCATATTATTGCAAACATCGAACTCATTTG |
| 102 | ATATATTTGCAAACATTGAACTCCATCAATTC | 94 | ATGAAATATGAATTTCGTATTTGTAAAAAT |
| 102 | TTTGCAAATATATGAAATATGGAGTTTCAATA | 94 | TTTGCAAAAATATGAAAATGGAGTTTCGTA |
| 102 | TTTGCAAATATATAAAATATGGAGTTTCGTA | 94 | TTTGCAAAAATATGAAATGTGGAGTTTCATA |
| 102 | TCAATGTTTGCAAATATATGAAAAATGAAGTT | 94 | GAGTTCAATTTTGCAAATATATGAAATGTG |
| 102 | TTCATATATTTGCAAACATTGAACTCCATTTA | 94 | CATATATTTGCAAACATCGAACTCATTAGTT |
| 102 | CAAACATCGAACTCATTTGTTCATATAATTA | 93 | ACAAATACTAAACTCATTTGTTAATATTTTT |
| 102 | AGTTTAGTATTTGTAAAAATATGAATAAATG | 93 | ATATGAAATATGGAGTTTCGTATTTGTGAAA |
| 102 | AACTCCATAATTCATATATTTGCAAACATCG | 93 | TTTGCAAAAATATAAACAAATAAGTTCAATG |
| 102 | TTTGCAAAAATATGAAATGTGGAATTTCGAA | 93 | GAGTTTCGTATTTGTAAAAATAAGAACAAAT |
| 101 | TATGAAATATTCAGTTTCGTATTTGTAAAAA | 93 | GGAATAGAACAGGTGCTCGGTT |
| 101 | TTTGCAAAAATATGACAAATGAGTTCGATG | 93 | GAGTTTAGTATTTGTAAAAAATGAACAAAT |
| 101 | AACTCATTTGTCATATTTTACAAATACGA | 93 | ATGAACAAATGAGTTTAATGTTTGCAATAAT |
| 101 | AACTCATTTATTCATATTTTTACAAATACAA | 93 | AAAATATATGAACAAATGAGTTCGATGTTTG |
| 101 | AAAATATGAACAAATGAGTTCAATGTTTGAA | 93 | AAATAGAGTTCAATGTTTGCAAATATATGAAC |
| 101 | TTCATATTTTTACAAATACAAAACTACACAT | 92 | TTTGCAAATATATGAAAATATGAGTTCAATG |
| 101 | CACACTTTTGCAAAAGTGTAAATTATATT | 92 | GTATTTGTAAAATATATGAACAAATGAGTTC |
| 100 | AGTTTTGTATTTGAAAAAATATGAACAAATG | 92 | AACTCATTTGTTCATATTTTACAAACACGA |
| 100 | TTCATATATTTGCAAACATTAAACTCACTTG | 92 | TATATTTGCAAACATTGAACACATTTGTT |
| 100 | AACTCATTTTTCATATATTTTTACAAATACGA | 92 | ATATTTGCAAACATAGAACTCATTTGTTC |
| 100 | ATAAAATATGGAGTTTCGTATTTGTAAAAAT | 92 | TTTGCAAATATATGAAAAATGAGTTAAATG |
| 100 | GTATTTGTAAAAATATGAACAAATGGAGTTCA | 91 | TTTGCAAAATATGAAATATGCAGTTTCGTA |
| 100 | ATATGAACAAATGAGTTCGATGATTGCAAAT |
| 99 | AACTCATATTTCATATATTTGCAAACATAG |
| 99 | TTTGCAAAAATATGAAAATGAGTTCAATG |
| 99 | TTTGTTCATATTTTTACAAATACAAACTCCA |
| 99 | ATATGAACAAATGAGTTCAGTATTTGCAAAT |
| 98 | AACTCCAATTTCATATATTTGCAAACATCG |

| **Bembidion lachnophoroides (#SRR2939022)** | | | |
| --- | --- | --- | --- |
| 1167 | ATCTTA | 9 | AATTTTTT |
| 591 | TCTTAAA | 9 | AAGAAGAAGACGAAG |
| 442 | ATCACTA | 9 | TTTAACGGATAATGA |
| 421 | ATAG | 9 | AACAA |
| 390 | **GTTAG** | 9 | AAAACCACCCCCCA |
| 258 | TTATCAC |
| 214 | AAC |
| 179 | GTCG |
| 174 | GTACAA |
| 143 | AAGGTTAAGTC |
| 129 | GCGA |
| 112 | ATA |
| 96 | ACCTTGAAATG |
| 55 | ACTTAACCTTGAAATGACCTTG |
| 47 | TCTG |
| 46 | TCAAGGTCAT |
| 43 | AATGG |
| 39 | TATTTTT |
| 38 | AAAAAAAT |
| 38 | ACTCCG |
| 36 | TAAAATG |
| 34 | AGATTAGTGCTTAC |
| 34 | ATAC |
| 33 | TGAAAACA |
| 31 | ATCACTAATCACTT |
| 30 | AAAATAAAA |
| 29 | AATTGAGTGCACGATTCTGATCCAGCAATAGTA |
| 29 | TCG |
| 27 | ATTGT |
| 27 | AAG |
| 26 | AAAAAAAAAT |
| 26 | CATAAGA |
| 26 | TCGCCATCGCGG |
| 25 | AATA |
| 24 | ACCTTGACTTG |
| 21 | TTTTTA |
| 21 | AGACGGTGAC |
| 21 | TTAACGGATAGTGAC |
| 20 | **CCCAAAC** |
| 15 | CATCAA |
| 14 | CAAAAA |
| 14 | TTTGTTT |
| 13 | TCGCCG |
| 13 | GTGGTTTTTTTGGG |
| 13 | TCAGGTCTA |
| 12 | CCTTGAAATAA |
| 12 | AAAAAAAAAC |
| 12 | TCA |
| 12 | CGTATT |
| 11 | TGCTTT |
| 11 | TTATGAATA |
| 11 | TTTTTTTATTT |
| 11 | AAAAAAAAC |
| 10 | ATTTTTTTTTTT |
| 10 | GGTTTTTGGGGGT |
| 10 | ACCATACGC |
| 10 | GCTGTT |
| 10 | CACCAA |

| **Bembidion obscuripenne (#SRR5230404)** | | | |
| --- | --- | --- | --- |
| 30967 | TTTA | 252 | AGACATTTACACGAGTCGTGTG |
| 7256 | TTTTTAGTCACGGATTATTTTTTGCGCTC | 248 | GAAAAT |
| 5682 | GAATGTCTCACACGACTTGTGT | 245 | CATGTTTT |
| 4871 | TCACACAACTTGTGTGAATGTC | 242 | AAATGTTCTAAACGCA |
| 4835 | TAT | 239 | AGTCGTGTAAGACATTCACACG |
| 4583 | AGAT | 239 | AAGTTAC |
| 3498 | AATTAAAATTA | 236 | AATAAATATA |
| 3226 | AATTAAA | 227 | TTTTTGCGCTCTTTTAGTCACGGCTTA |
| 2119 | TGACAGCAATAATCGG | 226 | CGAATATT |
| 1843 | TGAATGTCTCACACGACTCGTG | 223 | GTCACGGATTATTTTTGCGCTCTTTTA |
| 1625 | CTTATAA | 214 | GTGTGAATGTCTCACACGATTT |
| 1444 | TTTATA | 213 | AAAAATAAAGTTAA |
| 1434 | GAGCGCAAAAATAATCCGTGACTAAAAA | 209 | AAATACAAAGAA |
| 1164 | TTTTAGTCACGGATTATTTTTTGCGCTC | 206 | TGACTAAAAGAGCGCAAAAAATATTCCG |
| 891 | TCTTACACGACTTGTGTGAATG | 206 | ATTGCTGTCACCAATT |
| 859 | TTTTTGCGCTCTTTTTAGTCACGGAATAT | 205 | CCAACTAAATAAACCAAAACCAAACC |
| 856 | GTTAAAA | 205 | CTTTTTATCACGGATTATTTTTTGCGCT |
| 800 | TGTGAGACATTTACACAAGTTG | 204 | ACATTAACACAAGTCGTGTGAG |
| 793 | CGTGTGAGACATTTACACAAGT | 201 | AAAAAATTAA |
| 736 | ATTAAA | 198 | ATTTA |
| 681 | GAATTTCAGATATCTGAAATT | 193 | TAAATTAA |
| 667 | TTTACTTA | 192 | AAACTAGAGCATTTTTTCGA |
| 667 | ACTAAATTAACCAAAACCAAACCCCA | 192 | TTAAAAAAAAAG |
| 595 | CTCGATGACGAAAACCAAGTCAGTGTA | 191 | AATATAAACGAATAA |
| 595 | CTT | 185 | ATTCACACAAATTGTGTGAGAC |
| 552 | TTTG | 182 | AAAAC |
| 543 | CTTATATA | 181 | ATTTGCAGAAAATGCAGATTT |
| 519 | AAAACTGCTCTAGTTTTCGC | 180 | ACAATTT |
| 505 | ACAAGTTGTGTAAGACATTCAC | 178 | ATTTTTTGATGTTTC |
| 500 | ACCTA | 177 | TGTCTCACACAACTTGTGTTAA |
| 482 | ATAAA | 176 | ATTATTT |
| 480 | GTAAAAT | 175 | ACGGCTATTTTTTGCGCTCTTTTTAATC |
| 470 | CAA | 170 | AAAAAATAAAGTT |
| 453 | TTCACACAAGTTGTGTGAGATA | 168 | TTTTTTTATT |
| 451 | AAAGCAAA | 166 | GGGTTTGGTTTTGGTTAATTTAGTT |
| 450 | AGATATCTGAAATTCAATTTA | 165 | AAAAATAAGCCGTGACTAAAAAGAGCGCA |
| 434 | CGAAAACTAGAGCAGTTGTG | 165 | ATAATATAATTATATAT |
| 418 | AATTGAATTTCAGATATCTTG | 165 | AAACGCAATTTTTCCT |
| 410 | TAAAAAGAGCGCAAAAATAAGCCGTGAC | 163 | TTAAAACTAACGCGAC |
| 404 | AATTTCAGATATCTCAAATTG | 162 | TGAAAAC |
| 382 | TGTGAATGTTTCACACAACTTG | 155 | TAAAAATAAA |
| 377 | TTTTTGCGCTCTTTTTAGTATCGGATTAT | 151 | GTCTCACACGACTCGTGTTAAT |
| 371 | TTAAGTT | 149 | TTTATATGTACATTTT |
| 369 | AGATCGGAAGAGCACACGTCTGAACTCC | 148 | ACATAGAAA |
| 368 | CTATTCGAA | 148 | GAAAGTATGAC |
| 358 | TCGACTACGAAAACCAAGTCAGTGTAC | 146 | CATTTACACGAGTCGTGTGAAA |
| 343 | ATATA | 144 | TTTAATTTTTT |
| 317 | AAAAATAATAAA | 144 | TGTGAGACATTCACACAAGTCA |
| 315 | AGTCGTGTGAGATATTCACACA | 142 | AAATAATAAAA |
| 307 | TTTTCGAAAAACTGCTCTAG | 142 | AATAAA |
| 305 | CAAAAAATAATCCGTGATTAAAAAGAGCG | 140 | AAAATAATC |
| 301 | GTAT | 139 | TAATATTAA |
| 300 | GTCGTGTGAAACATTAACACGA | 138 | TTGGTTTTGGTTAATTAAGTTGGGGT |
| 299 | GACTCGTGTGAATGTTTCACAC | 138 | AAAAAAAAAC |
| 298 | GAAAACCAAGTCAGTGTACTCGACAAC | 138 | AGACATTCACACAAGTTGTGTT |
| 295 | AATAAAATA | 137 | TATTTATA |
| 281 | ACAAAAATAATA | 136 | GAAAACCAAGTCAGTGTACTTCGACAAC |
| 273 | CGACTTGTGTGAATGTTTCACA | 135 | GAAA |

| 134 | AAAAAAATATAGAGTTA | 86 | GTTTTCGAAAAATTGCTCTA |
| --- | --- | --- | --- |
| 133 | ATTATTCATCTCTCCG | 86 | TGTGAATGTCTCACACGACCTG |
| 132 | TTTTTATTTTT | 86 | GACTTGTGTGAATGTCTCACAA |
| 131 | AGTTGTGTGAGACATTCACACG | 85 | GACTTGGTTTTCGTCATCGAAGACACT |
| 130 | AACAAAAATGAAAA | 85 | AAACGTAATTTTTCCT |
| 130 | TTTCAAA | 84 | TTCA |
| 128 | ATAAATAAAATAAGAACATAA | 84 | GACTTGGTTTTCGTCATCGAATACACT |
| 127 | CTGACCTAAA | 83 | ATATCCAAATTGAATTTCAG |
| 126 | AATTGATTTCGAGATATCTAA | 82 | AAAAGAGCGCAAAAAATAATCCGTACTA |
| 124 | GTTGTGTGACACAA | 81 | CTTGTGTGAATGTCTCACACTA |
| 124 | ATTTCAGATATCTGAAATTA | 81 | AAAAAAAAAATT |
| 123 | TTTTTTTTAG | 81 | ATAATTTGTCCTTAAAAATTTCAT |
| 120 | GTATTTT | 80 | TAATTTTGCTCATCA |
| 119 | AAGTTAAAAAAATATA | 80 | AAGTTAAAAGAAAC |
| 119 | GTTCAAATTCAG | 80 | ATTTTTTTAAAG |
| 118 | TTTTTTTA | 79 | TAAAATACA |
| 116 | CCAGTTTTCGAAAAAATGCT | 79 | ATTCGC |
| 116 | ATTCTAC | 78 | ATTTTTATA |
| 115 | CTTGCGTGAATGTCTCACACAA | 78 | TAATAATTT |
| 114 | ATAAATAAAAA | 78 | AAATACGAAATAGA |
| 113 | ACCGATTATTGCTGTT | 77 | TTTAAGATTT |
| 113 | TAATTTTGTGC | 77 | AAGATATCTCGAAATCAATTT |
| 112 | TGAGATATTCACACGAGTCGTG | 76 | GAATGTCTCACACATCTTGTGT |
| 112 | TTCGTATTTAGTAT | 76 | AGAAATATATTTATATTAAGATATT |
| 111 | AAATGTAC | 75 | ATTCGATCTATTCGAAT |
| 110 | AAATAATAATGATCTAAATTCT | 74 | ACTTATT |
| 109 | TCGAAGACACTGACTTGGTTT | 74 | ATATCTGAAATTGAATTTAAG |
| 106 | AATCCTG | 74 | CCCCCCCCCA |
| 105 | TACG | 73 | TTGCTGTCACCAAATA |
| 104 | GAATATA | 73 | TAGATATCTCGAAATCAATTC |
| 104 | TTTTTTTAACTTT | 72 | AAAACTGCTCCAGTTTTCGC |
| 103 | GTCGTGTGAGACATTCACACGG | 72 | TGAGACATTCACACAAGTTTTG |
| 102 | TTGAAAACCAAGTCAGTGTACTCGATG | 71 | ATTTTTTTA |
| 101 | TGCGAAAACTAGAGCAATTT | 71 | TGGAA |
| 100 | TCCAGAAAATAGTAAATAACAAG | 70 | TTTAACTAT |
| 100 | TTCAAATTCAGA | 70 | TGTGAATGTCTCACACGTCTTG |
| 100 | ATTTCAGATATCTAAAATTGA | 70 | TTTTTG |
| 99 | TTTTTGCGCTCTTTTAAGTCACGGATTAT | 69 | CAAATAAA |
| 99 | TAATTTTGCTCAACA | 69 | TAATAAAAA |
| 99 | AAAAAAAAAAC | 69 | CAACTTGTATCACA |
| 98 | GACATTCACACAAAATGTGTGA | 69 | CAAAAAATATTCCGTGATTAAAAAGAGCG |
| 98 | CTAGTTTTCGCAAAAATGCT | 69 | TACAA |
| 97 | AAAAGAGCGCAAAAAATAATCCGTGAATA | 68 | AAGTAAAAATA |
| 96 | TTTATTTAA | 67 | TTTATAATTTTTTTTAAC |
| 96 | AACTTTATATTTTTTTT | 67 | TCCAGTTTTCGCAAAATTGC |
| 96 | CAAGTCGTGTTAGACATTCACA | 66 | TTTTTGCGCTCTTTTTAGTCACAGATTAT |
| 96 | GTGTGAATGTCTCACACGAATT | 66 | TTTCTCGAAAACTAGAGCAA |
| 95 | CTGACCTAAT | 66 | TTATTTAT |
| 95 | TGACTTGGTTTTCGAAGTCAAGTACAC | 65 | ATTTAAC |
| 95 | TTTTTTGCGCTCTTTTAGTCACGGCTTA | 65 | ACAAGTTGTGTAAGACATTTAC |
| 94 | TATACATA | 65 | AAAAATATTCCGTGACTAAAAAGAGCGC |
| 94 | AAAGAAAAT | 64 | TTGCGAT |
| 92 | AATGTTG | 64 | TTGATTTA |
| 92 | AGATTGAATATTCGAAT | 64 | AAAAAAG |
| 92 | TGA | 64 | TAAAAATAAAA |
| 91 | ATAAAGTTAAAAAGGAAATAG | 64 | CGGATTATTTTTTGCGCTTTTTTTAGTCA |
| 90 | TGTACGTA | 64 | ATTTCTGG |
| 89 | ACACGACTTGTGTAAATGTCTT | 63 | AATTGAATTTGAGATATCTAA |
| 88 | AAACCAAGTCAGTGTCTTCGACAACGA | 63 | ACACGACTTGTGTGATTC |

| 62 | TATTTTTTGCGCTCTTTTATGTCACGGAT | 50 | ACCAAACCCCAACTAAATTATCCAAA |
| --- | --- | --- | --- |
| 62 | CCAA | 50 | TTGTTTCGATATTTTTTG |
| 61 | CCACCCCCCCCCCCC | 49 | CATTCACACAAGTCGTGTAAAA |
| 61 | ACCAAACCCTAACTAAATTAACCAAA | 49 | CGCT |
| 61 | GTGTGAATGTCTCAAACAACTT | 49 | CCCCCCCCCCCCA |
| 60 | TTGATATATTCACCCATTAT | 49 | ATTAATTTTAAAATTAATA |
| 60 | CCTGAAAT | 49 | TATCTTAATATAAATATAATTCTAA |
| 60 | TCGACTACGAAAACCAAGTCAGTGTCT | 49 | TTTTTGTC |
| 60 | AAAAAAATGTTAGG | 49 | TGTGAGACATTCACACGAGTCA |
| 60 | AATTCAATTTCAGATATCTTGA | 49 | CACACGACTTGTGTAAATGTTT |
| 59 | CTCGTGTTAATGTTTTACACGA | 49 | AAACTAGAGCATTTTTTCGG |
| 59 | CAAGTCGTGTGAGAAATTCACA | 49 | CAAAAATAA |
| 59 | GTTTCGATGCAACGAC | 48 | ACT |
| 59 | TTTTTTTAACTTTATAA | 48 | CATTCACACAAGTTATGTGAGA |
| 59 | TTTAATGTTTTTAATTTAAGTT | 48 | AAAAAAATAAAGTTA |
| 59 | ATTCACATAAGTCGTGTGAGAC | 47 | GTGTTAATGTTTCACACGACTT |
| 59 | TCACACAAGTTGTGTAAAACAT | 47 | TATATAAAATAAAATAT |
| 59 | ATTTAGATTTATAA | 47 | ACAAAAA |
| 58 | GGGTTTGGTTTTGGTAATTTAGTTG | 47 | TGTGAATGTCTCACACGACTCA |
| 58 | AGATATCTGAAATTCAATTT | 47 | TAATCCGTGACAAAAAGAGCGCAAAAAA |
| 58 | ATAACTAA | 46 | GTCTCACACAACTTTTGTGAAT |
| 58 | TTTTTTTTTTAT | 46 | TTCATAAATTTCATAATT |
| 58 | TTTCTTATTTT | 46 | AAATAC |
| 58 | AAATTAAAATAGTCGAAATCTGTTTT | 46 | ACTAAAAAGAGCGCAAAAATAATCCGAT |
| 58 | TTGTGTGAATGTCTCACACAC | 46 | ATTTTTTGCGCTCTTTTTAGTCACGGAT |
| 57 | GTGACAGTTCTCGCCACAAAA | 46 | TTTGTTTTTTTTA |
| 57 | TTCGTTGTCGAAGTCACGACTTGGTT | 46 | TCACACGACTAGTGTGAATGTC |
| 56 | TCACACAAGTCGTGTGAGACAA | 45 | AATATATTTTATTCAAAAGT |
| 56 | CCCCCCCCCCCACC | 45 | GACTTGGTTTTCGTAGTCAAGTACACT |
| 56 | GTGTGAGACATTCAAACAAGTC | 44 | TACAAACA |
| 55 | CTAAATTTACCAAAACCAAACACCAA | 44 | TTTTTGTTTTTT |
| 55 | TTATGAAAT | 44 | TTGCGCTCTTTTTAGTAACGGATTATTTT |
| 55 | TCTTACACAACTTGTGTGAATA | 44 | ATTTTTTGCGCTCTTTTTTGTCACGGAAT |
| 55 | TGTCACCGAATATTGC | 44 | TTCAGT |
| 55 | TGAAATTCAATTATGAGATATC | 44 | GCTAAGAACACCGCTTCTCAGCCTATG |
| 55 | TTTTTAATACATTTTA | 44 | AATTAACCAAAACCAAACCCTTACTT |
| 55 | TATTATAATATAT | 44 | TTTTTCGTTTGGATGT |
| 54 | AACTTTATACTTATA | 44 | GAAATAC |
| 54 | ATTTGTGC | 44 | TCTC |
| 54 | CCCCCCCCCCA | 43 | ACCAAAACCAAACTCCAACTAAATTA |
| 54 | AGATCGGAAGAGCACACGTCTGAACTCCC | 43 | GTTTTAATTT |
| 54 | CTGGAGCAATTTTTCGAAAA | 43 | TCACGGATAATTTTTTGCGCTCTTTTTAG |
| 54 | TGACTTGGTTTTCGTAGTCGAAGTACAC | 43 | ATCTTCAATTTTAAAATATG |
| 53 | GTCGCGTTAGATTTAA | 43 | GTGTAAAACATTCACACGAGTC |
| 53 | CGGATTATTTTTTGCGCTTTTTTAGTCA | 43 | ACAACTCAAG |
| 53 | ATATCTTAAATTAATTTCGAG | 43 | GTTATA |
| 53 | GGCT | 42 | AATTTCAGATATCTGAAATTA |
| 53 | ATTTAATTAAT | 42 | AGCACAGAATT |
| 52 | GAAAATTAAAGT | 42 | ACTTGATTTTTTTA |
| 52 | ACAAATTGTGTGAGACATTTAC | 42 | CCCCCCCCA |
| 52 | CTGAAG | 42 | AACTGCTCTAGTTTCCGCAA |
| 52 | ATATCTGAAATTCAATTATAG | 42 | TTAAATAAAAATTTTAT |
| 52 | GAAAACCAAGTCAGTGTACTCGATAC | 42 | CTCAG |
| 51 | GTCACGGATTATTTTTTGCGCTCATTTTA | 41 | GGAAACTGGAGCAGTTTTGC |
| 51 | GGTCAGGTTA | 41 | TTAAAAAAATAG |
| 51 | GTTATAAGC | 41 | TATA |
| 51 | TTTTTGCGCTCTTTTTAGTCACGGATTT | 41 | CCCCCCCCCACCCCCC |
| 50 | ACGGATTATTTTTTGCGCTCTTTTTATT | 40 | TATTATATTTATATTGATT |
| 50 | GTGTGAAACATTTACACAAGTT | 40 | TACATT |

| 40 | TTGGTTGTCT | 35 | ATTATATATAAAATTTAT |
| --- | --- | --- | --- |
| 40 | TCTCACACAACTTGTGTAAATA | 35 | CTAGAGCAGTTTGGCGAAAA |
| 40 | TAGTTACGGATTATTTTTTGCGCTCTTTT | 35 | TTTAATTTTTAATTTTA |
| 40 | GACTAAAAAGAGCGCAAAAAATAATCCGAT | 34 | GTTGGGGTTGGTTTTGGTTAATTTA |
| 40 | AAAAAATAT | 34 | GTATTTGT |
| 39 | TATCTTAAATTTAATTTGAGA | 34 | AATATCTTACACGACTTGTGTG |
| 39 | GCTTTT | 34 | ATTTATCTCTCCGATT |
| 39 | TTTATTAT | 34 | AGACATTTACACGAGTCGTGTA |
| 39 | TCTCACACGACTTGTGTAATG | 34 | TTCG |
| 39 | TTCAA | 34 | ATATCTTAATTAATTTCGAG |
| 39 | CGTGATTAAAAAGAGCGCAAAAAATAAGC | 34 | TTGTGTAATGTCTCACACAAC |
| 39 | CCCACCC | 34 | ACATTTTTTTACTA |
| 39 | ATATCTCAGAATTGAATTTCAG | 34 | GTGTGAAACAGTCACACGGGTC |
| 39 | TTTTAAATTG | 33 | AGATATCTTAAATTGAATTTG |
| 39 | ATATATGTATGTGT | 33 | GTAAATGTCTCACACGACCCGT |
| 39 | CCCCCACCCCCC | 33 | ATTATAT |
| 39 | GTGTGAAAGTCTCACACGACTT | 33 | ATTCGTG |
| 39 | TAAAAAATAA | 33 | TTATGTATTC |
| 38 | TATTCGAACTATTCGAA | 33 | TCTCACACGACTTGTGTAAATA |
| 38 | ATATCTCGAAATCAATTCAAG | 33 | TTTTCGAAGTCGAGTACACTGACTTGG |
| 38 | TTAC | 32 | TGTGAGACATTCACATAAGTTG |
| 38 | TTAATTTGAGATATCTGAAA | 32 | TCTTTGTATAATTTA |
| 38 | AAAAAAAAATAAA | 32 | AAAGAAAAC |
| 38 | GATTCTA | 32 | AAAACTAGAGCAAACTTCCG |
| 38 | CCCCCCCA | 32 | GACTTGGTTTTCGTTGTCGAGTACAC |
| 38 | TTTTTTTTAAGCACAGTGGTGAACGTTG | 32 | TTCTA |
| 38 | TAAAATATATAAAATATTTT | 32 | CACAAGTTGTGAGAGACATTCA |
| 38 | GTGTGAGACAATCACACAAGTT | 32 | AGTTTTTAAAGTTGAAATTTCA |
| 38 | GTGTGAGACATTCACACAAGCT | 32 | AAAAATCATCCAAACG |
| 38 | ACTTATCCTTCAGA | 32 | AAAAAATATT |
| 37 | ATGTTTCAGCA | 32 | GCGGTTTCTGAGCGGTTTTTA |
| 37 | TTTTAAA | 32 | AAACCGCTAA |
| 37 | TTAATAATTTT | 32 | GTGTGAATGTCTCACACAACTA |
| 37 | AATGTCTCACACGACTGTGTG | 32 | GACTTGGTTTTCGTTGTCGAAGCAC |
| 37 | TTTTATATTT | 32 | AGACATTCACACAAGTTGTGTC |
| 37 | CAGCAAAAATCGGTGA | 31 | ATGTCTACACAACTTGTGTGA |
| 37 | GGGTCGTGTGAAACATTCACAC | 31 | ACTAAATTGTTCATT |
| 37 | AGAC | 31 | TTAA |
| 37 | TAAATAAAAAAA | 31 | ACAAACAC |
| 37 | TCTTTCA | 31 | TAAAATACAAACGAA |
| 37 | TGTGAATGTCTCACACGACTTT | 31 | CTAA |
| 37 | ATTACTCGCGT | 31 | AAAAAAAAATTAA |
| 36 | GTTTTCT | 31 | TATATTTATTTTATAAAA |
| 36 | CAAGTTGTGTGAGAAATTCACA | 31 | TAAAAATTATTGAAATTAA |
| 36 | TCTTTGTTGTATATTCTTTCT | 31 | ATGCTGG |
| 36 | ATTTATTTTACTT | 31 | GCTCCAGTTTTCGAAAAACT |
| 36 | GTGTCAATGTCTCACACGACTT | 31 | AGAAAA |
| 36 | TACATA | 31 | TCTTTATTTTTCTTAAATTTGATGT |
| 36 | AGATCGGAAGAGCACACGTCTGAACTCCAGTCC | 31 | TAAACACG |
| 36 | AACTAAATAAACCAAAACCAAACCC | 31 | ACACTGACTTGGTTTTCGCAGTCAAGT |
| 36 | TAAAACAGGCTTAAAAGACT | 31 | GTGAGACATTCACACAAGTCGA |
| 36 | AATGTCTCACACGATTTGTGTA | 30 | CCCCCCCCCCCCCCCCAC |
| 36 | TTTTAACTTTATTC | 30 | TTATTTTATTTAT |
| 36 | AAAAATTA | 30 | TAATTTTTTA |
| 35 | TGAGACATTCACTCAAGTTGTG | 30 | ACCAAAACCAAACCCTAACTTAATTA |
| 35 | CTGAAATA | 30 | ATGGCTAAGAACATCGCTTCTCGGCCT |
| 35 | TTTACTTTAT | 30 | AAATAATTTAATTTATTTTGA |
| 35 | AATATATAAG | 30 | AAATGCTCCAGTTTCCGAAA |
| 35 | CTTGTGTGAATGTGTCACACGA | 30 | TTTGTTTTT |

| 30 | TTTTATTCATTTATA | 25 | TTTTTTAACTATT |
| --- | --- | --- | --- |
| 30 | ATTCGT | 24 | ATTGCCTTTTTGTTCATATTTCAGTT |
| 30 | CTTGTGTGAATGTCTCTCACGA | 24 | TTTATTGTATATTT |
| 30 | ACACGACTTGTGTGAATGTCT | 24 | TATTTTTTGCGCTCTTTTTAATCACGGAC |
| 30 | TGTGAATGTATCACACAACTTG | 24 | ATTTTTTGCGCTCTTTTAATCACGGATT |
| 29 | CCCCCCCCCCCCACCCC | 24 | GTGTGAATGTTTCACACAACTC |
| 29 | TAAATTAACCAAAACCAAACCCCAAT | 24 | ATATGTG |
| 29 | TTATTTTATTTCTAAA | 24 | GTGTAAGACATTCAAACAAGTC |
| 29 | AAAAATAATCCGAGACTAAAAAGAGCGCA | 24 | AAACTTGAAATTTCAACTTTT |
| 29 | ATTTTTTTTATTT | 24 | ATTTTTGA |
| 29 | CTCAGAAACCG | 24 | TTTTTTTTATAA |
| 29 | TTTAG | 24 | TTTATTATTA |
| 29 | TGTGAGACATTCACACAAGCCG | 24 | ATTTTCTTTTTAACTTTATC |
| 29 | AAAAAAAATAAAGT | 23 | TTTAAGTTTTGAATATTTAGTT |
| 29 | GTAAATGTCTCACATGACTTGT | 23 | TATATCGACTTTATTCTCAA |
| 28 | AATGTCTCACACGACTTGTGAG | 23 | TACAATAATAAGA |
| 28 | AGATCGGAAGAGCACACGTCTGAACTC | 23 | TGTGAAATATTCACACAAGTTG |
| 28 | AGATCGGAAGAGCACACGTCTGAACTCCAGTC | 23 | TTAAAGCAAAATAAA |
| 28 | AAACCCAACTTAATTAACCAAAACC | 23 | TGTATTTGTATATTTG |
| 28 | TATATATATT | 23 | CAATTTCGCGAAAACTAGAG |
| 28 | AACCAAACCCTACTTAATTAACCAA | 23 | ATTATTCATCTCCCCG |
| 28 | AGCATCAGT | 23 | TTTATTTATTTC |
| 28 | AAAAGAAA | 23 | TAATATAGATAGTTAT |
| 28 | CAAATTTGATAT | 23 | ATAAAAAAAAAAATTATA |
| 28 | AAAATAATAT | 23 | CTTAAATATTTTTAAA |
| 28 | GTCACGGATTATTTTTTGCGCTCCTTTTA | 23 | TAAATAC |
| 28 | TTTTTATTTTTTTT | 23 | TTGTTTTTG |
| 27 | AAAATACAAAAA | 23 | TCACACGACTCGTGTAATGTT |
| 27 | AGAGCGCAAAAAATAGCCGTAACTAAAA | 23 | TTTTTTTAACTTCATA |
| 27 | TTTTTTAAATTT | 22 | ATTTTAT |
| 27 | GTTTCACACAATTTGTGTGAAT | 22 | TTTTTTTTGTTC |
| 27 | AAAATTATTTTTTCTATT | 22 | ACAAGTTGTGTGAGACATTCAA |
| 27 | GACATTCACACAAATCGTGTAA | 22 | TGAATTTGAACT |
| 27 | AAAAAATAAATT | 22 | TAAATTAACCAAAACCAACCCCCAAC |
| 27 | CCCCCCCCCCCCCCCCCCA | 22 | GTTTACAACTGACTACT |
| 27 | AAAAAAATTAC | 22 | GTGTGAATATCTAACACAACTT |
| 27 | AGTTGAGGTTTGGTTTTGGTTAATTT | 22 | TAACCAAAACCAAACCCCAACTAAA |
| 27 | AGACATTCACACAAGTCATGTA | 22 | AATTTCAGATATCTCAAATTC |
| 27 | GTCACGGAATATTTTTTGCGCTCTTTTT | 22 | GACTTGGTTTTCGTTATCGAGTACACT |
| 27 | TCACACGACTTGTATGAATGTC | 22 | GTGTGAATGTCTCACACAAATT |
| 27 | TTTTTTGCGCTCTTTTTAGTCACGGACTA | 21 | TTCAGTCT |
| 27 | TTTTTTTTAACGTTTATA | 21 | AACCAAGTCAGTGTCTCGACAACGAA |
| 26 | GGGTTTGGTTTTGGATAATTTAGTT | 21 | TCACGGATTAATTTTTGCGCTCTTTTTAG |
| 26 | TATTTTTTGCGCTCTTTTTATCACGGCT | 21 | AAGATATCTTAAATTCAATTT |
| 26 | AAAAAAAC | 21 | AATACATCAAATAATGGGTG |
| 26 | TTTTTGCGCTCTTTTTAGTCACGAATTAT | 21 | TTTTTTTAAA |
| 26 | AAATGTCTCACACAACTCGTGT | 21 | ATTTTTAAAGACAAATTATATGAA |
| 26 | GGGGGAGG | 21 | TATTATTA |
| 26 | TTATTTTTGTTTT | 21 | AAGAAGAGTTTTCTA |
| 26 | ATATATATATATATAA | 21 | TGTTTATATA |
| 25 | TTTTC | 21 | TAATAAAATA |
| 25 | CGGATTATTTTTGCGCTCTTTTTAATCA | 21 | AGACATTCACACGAGCCGTGTG |
| 25 | AAAAAAATAAAATT | 21 | CCCCCCACCCCCCCCCCCCCC |
| 25 | AACTTTATATTTTTTTTT | 21 | TGTCTTCGATGACGAAAACCAAGTCAA |
| 25 | AAGATATCTGAAATTAAATTT | 21 | TTAAAAAAAG |
| 25 | AATACCTG | 20 | AACCGCTCAAA |
| 25 | AAAAAAATAAAGAT | 20 | AAAACCAAACCCTAACAAAATTAACC |
| 25 | ATATTATTATAAA | 20 | TCTATTTA |
| 25 | CACAAGGTGTGTGAGACATTCA | 20 | TTATTTTTCATTTGTT |

| 20 | CTCAGGAACCG | 17 | CTAAACGCAATTTGTTC |
| --- | --- | --- | --- |
| 20 | TAAAAATACA | 17 | CGAAAACCAAGTCATGTCTCGACA |
| 20 | GGCTACACTTGTTTTATCCTCT | 17 | TTCTTTTTAACTTTATAATT |
| 20 | TCTTCTATA | 17 | TTTTATTTG |
| 20 | TTATTTATTTA | 17 | TCTGGACTTGTTATTTAATATTT |
| 20 | AGATCGGAAGAGCACACGTCTGAACTCCAC | 17 | TTTCA |
| 20 | TAATTA | 17 | TAATTATGTGC |
| 20 | TTCTATATTCTA | 16 | AACTGCTCCAGTTTCCGAAA |
| 20 | ATATAAAATATTTTTAAGAA | 16 | GTGTGAATGTCTCACAAGACTC |
| 20 | AATTTAATTTGAGATATCTAA | 16 | TTTAATTATTTATGTTCTAT |
| 20 | AATGTCTCACACGACTTGTGCG | 16 | AATGCTCCAGTTTTCGCACA |
| 20 | TATTATGTTATG | 16 | ATTTTTTATTTT |
| 20 | ACTTTACCGCACGCATTTT | 16 | ACAACTTGTGAGAATGTCTCAC |
| 20 | TTTTAATTAAAACAGATTTCGACTA | 16 | GTCTCACACGACTTGCGTGAAT |
| 19 | GTTTTTAAGTCGCGTTA | 16 | AATGCCTCACACAACTTGTGTG |
| 19 | AATATT | 16 | GTGTGAGACATTCACACAAAAC |
| 19 | TAAAAAAATCATGAAGT | 16 | CCCCCCCCCCCAA |
| 19 | TCGCTAT | 16 | GCTCTTTTTAATCACGGTTATTTTTTGC |
| 19 | TTTTTTAATTTAAATAATT | 16 | TGTATTC |
| 19 | ATCGGATTATTTTTTGCGCTCTTTTAGTC | 16 | TCACACAACTTGTGCGAATGTC |
| 19 | AAATAAATAAAAATT | 16 | TATTATATTATTATAAAATA |
| 19 | CGACGTTTCGAACCAG | 16 | TACAAAAAAA |
| 19 | TTTTATGTATTATTA | 16 | AATGCCTCACACGACTCGTGTG |
| 19 | AATAGATCG | 16 | ATAATAATATTAT |
| 19 | TTGTGTAAATGTCTCACACGAA | 16 | AAAAAGGCAATAACTAAAATATAAC |
| 19 | ACTTGAGTGAATGTCTCACACG | 16 | TATTTTAATT |
| 19 | GTTTCAGCAAC | 16 | TTTTTGTTTTAATTT |
| 19 | TTTAATTATTTTAATAACT | 16 | TTCAGCGACTTGTTTTTCTTC |
| 19 | TTTTTTAACTTTC | 16 | TTCAGTCG |
| 19 | TCCCTTTTCTATGAACTCA | 16 | CGAAAACCAAGTCAATGTACTCGACTA |
| 18 | TTACTATATTTTT | 16 | CCCCCCCG |
| 18 | TCCTGTTGTCC | 16 | GGGGGAGGGGG |
| 18 | TTTTTTAATGTTTATAATT | 16 | TATTTTATA |
| 18 | TTTTTAACTTTATAAT | 16 | GTACATACGTAC |
| 18 | TCTCACACAACTTGTGTCAATG | 15 | AAGTCATGTCTTCGACAACGAAAACC |
| 18 | ACCCCC | 15 | TCGAGAGACACTGACTTGGTTT |
| 18 | CCCCCCCCCCCCCCG | 15 | TCC |
| 18 | ACGGATTATTTTTGCGCTCTTTTTAGT | 15 | AAAACTGAAATACTAAAAACTA |
| 18 | CCCCCCCCCG | 15 | TATTTTTAAAAATATAAAA |
| 18 | TTTTAG | 15 | TGCTGAA |
| 18 | TTTTTTTTGTTTAA | 15 | AATATTCGGATATTCA |
| 18 | TTTCAGATATCTAAAATTAA | 15 | CCCCCCCCCCCG |
| 18 | CACGGTTATTTTTTGCGCTCTTTTTAGT | 15 | GGATTATTTTTTGCGCTCTTTTTAGTCAA |
| 18 | CCCCAACTAAATTACCCAAAACCAAA | 15 | CCCCCCCCG |
| 18 | TTTTTTTAACTTTATT | 15 | TTGTGTGAAACATTCACACAAC |
| 18 | ACTTGTGTGAATGTCTCAAACG | 15 | GAAAACCAAGTCAGTGTAATCGATAC |
| 18 | TATTACTTTTATATAAATTTGTAATTTC | 15 | GTGGTGATTCGGCCGC |
| 18 | ATATAAATAATA | 15 | AATGTCTCACACCACTTGTGTG |
| 18 | ATCTTTGAGAACTT | 15 | TTTGTATTTTT |
| 18 | ACACAAGTCGTGTCAGACATTC | 15 | GACTTGGTTTTCGTTGTCGAATACACT |
| 18 | GATTATTTTTTGCGCTCTTTATAGTCACG | 15 | CACACGACTCGTATGAATGTCT |
| 17 | ATTTTTAT | 15 | ATTGCCTTTTTGTTCATATTTCATT |
| 17 | TTTGCTCTAGTTTTCGCACA | 15 | AAAAAAAAAACAA |
| 17 | TTATAAAATATAAG | 15 | GTCACGGATTATTTTTGCGCTTTTTTA |
| 17 | TATTTTTTGCGCTCTTTTTATCACGGC | 15 | ATTATTTTTTGTTGTTAA |
| 17 | CCAACTAAATTAACCAAAACCAAACA | 15 | AAAAATAAAGTT |
| 17 | CTTTTTAGTCACGGATTTTTTTTTGCGCT | 15 | CTACATTCTATATT |
| 17 | TTTTTGCGCTCTTTTTATTATCGGATTAT | 15 | TATTATTTTTATA |
| 17 | TTATTCAT | 15 | AATGTCTCACAAGACTTGTGTA |

| 15 | TTTTTTCG | 13 | GCCCCCCCCCCCCC |
| --- | --- | --- | --- |
| 14 | AGACATTAACACAAGTCGTGTA | 13 | GTCGAGACACTGACTTGGTTTTCGTA |
| 14 | TACACTGACTTGGTTTTCGTCATCAAG | 13 | TCTTTTTATTCACGGAATATTTTTTGCGC |
| 14 | TGTCAATGTCTCACACGACTCG | 13 | TTAAATAT |
| 14 | TGAATGTCTCACACAACTTGTA | 13 | GTATCTCA |
| 14 | CTAAAAAGAGCGCAAAAAATAACCCGTGA | 13 | GGGGGAGGG |
| 14 | TCACACGACTCGTATTAATGTT | 13 | GCCCCCCCCCC |
| 14 | AAATGCTCTAGTTTTCGAA | 13 | GAATATTTTTGCGCTCTTTTAGTCACG |
| 14 | GGGGGGGGGGGA | 13 | AACTTTTTT |
| 14 | TCTTCTAAAA | 13 | TTTTTTTAATTTTATAT |
| 14 | TTTTTTAG | 13 | AAAAATTATTAAAATAAT |
| 14 | TATTTTCTA | 13 | TTTCAAT |
| 14 | TCTCACACGACTCGTGTAATG | 13 | TAAAAAAATAAAAA |
| 14 | AAAACTTAAATATATACAAATATTC | 13 | TTATATATTTTAT |
| 14 | TTTAGAAAATTTTGCG | 13 | TCACGGATTATTTTTTGCGCTCTTTCTAG |
| 14 | AACAAAAAAGAAAA | 13 | TTCAAA |
| 14 | ATTTTTTTTTAAT | 13 | GTCACGGATTATTTTTTGCGCTCTTATTA |
| 14 | CTTCTAAAT | 13 | CTTGTGTGAATGCCTCACACGA |
| 14 | ACCAAAACCAAACCCCAACTTAATTT | 13 | CCCCCCCCCCAA |
| 14 | TAAATGTTTTACACGACTCGTG | 13 | CTGCTCTAGTTTTCGCAAAAA |
| 14 | AACTTTATATTTATA | 13 | TTCAGATATCTGAAATTGAA |
| 14 | GGAGGGGGGG | 13 | GAAAACCAAGTCAGTGTCCTCGATGAC |
| 14 | ACACTGACTTGGTTTTCGTAATCGAGT | 13 | GTGATTAATAAGTAAGTC |
| 14 | AAAAACATCCAAACG | 13 | CGAAAACCAAGTCAGTGTCCTT |
| 14 | TGAATGTCTAACACGACTCGTG | 13 | ACCAAACCCCAACTAAATTAACAAAA |
| 14 | ACACGACTTGTGTAAATGTCTA | 13 | TTCACACAAGTCGTATGAGACA |
| 14 | TGACTTGGTTTTCGCTGTCGAGACA | 13 | TAATTTATTGC |
| 14 | TATTTTTTGCGCTCTTTTTTATCACGGC | 12 | GTTTGGTTTTGGTAAATTTAGTTGGA |
| 14 | AATTCAATTTCAGATATCTTG | 12 | ACGGCTATTTTTTGCGCTATTTTTAGTC |
| 14 | CTTTTTTTT | 12 | TTTCTTAAATAATT |
| 14 | TTTTTAATTTCAATATAT | 12 | CTAATTTTTTTAA |
| 14 | TATTTTTTACT | 12 | AAATTATAAAAAATATTA |
| 14 | TTTTATTTTTTTTAA | 12 | ATTTTATAAAT |
| 14 | AAGTTAAAAAAAATAAATAAAAAAGA | 12 | ATTTCAGACGTCTGAAATTA |
| 14 | ATTTTTTTGTTAT | 12 | TTCACACAAAAAGTGTGAGACA |
| 14 | ATTTTTAATT | 12 | CCAACTAAATTAACCAGAACCAAACC |
| 14 | TCGAAGTACACTGACTTGGTTTTCGTCA | 12 | ACTGAAAAACCAGAAATCCAAGAA |
| 14 | CCCCCCCCCCCCG | 12 | GTTGTGTC |
| 14 | TGTAGTAAT | 12 | AAAAAGACAAAAAGTA |
| 14 | ACAACTTGTGTGAAAGTCTCAC | 12 | AAAAAGAGCGCAAAAAATAATCCGTGACC |
| 13 | ATATTTTTTTAA | 12 | GAATCGT |
| 13 | GTAAATGTCTAACACAACTTGT | 12 | ACATATAGTTGCTGTATACGAAC |
| 13 | ATTGAATTTCAGACGTCTGAA | 12 | TTTTTAAAATTA |
| 13 | AACTAAATAAACCAAAACCAAACCCT | 12 | TGTGAATGTATCACACGACTTG |
| 13 | GGATTTTTTTTGCGCTCTTTTAGTCAC | 12 | TTTTTTTTCT |
| 13 | TTTATTATTATTA | 12 | ATTTATTTATGTTCTTTTTA |
| 13 | GGTTTATTTTTTTTGAGTTTA | 12 | CACGTAATTTTT |
| 13 | GTCACGGATTATTTTTTGCGCTCGTTTTA | 12 | TTCTATACTA |
| 13 | ATAATTTAAAAA | 12 | ATTTTTGTTTTAATTT |
| 13 | TTCTTAAATC | 12 | TTTATTTTATATAC |
| 13 | AGCTGTA | 12 | AATTTTCTTTATTTATTTTTACACCTG |
| 13 | TCGTGTGAAAGTCTCACACGAC | 12 | GTGTGAATGTCTCACACGAATC |
| 13 | GTTTTCGCCAAACTGCTCCA | 12 | ATAATTTATCAACAAAAA |
| 13 | ACATTCACACAACTTGTGTGAG | 12 | TATTTTTTTTA |
| 13 | GTTAC | 12 | TTTTGCGCTCTTTTTAGTCATGGATTATT |
| 13 | TAGTTGTCCTTTGCTC | 12 | CTCTAGTTTTCGAAAACTG |
| 13 | AAACTGCTCTAGTTTTCGAG | 12 | TAATAAT |
| 13 | ATTTTTTTAATATTTTTTTA | 12 | AATGTCTCACACGACTTCTGTG |
| 13 | TGTAAATATCTCACACGACTCG | 12 | ACACAATACATA |

| 12 | TTTTTGTTTTAATT | 11 | TAAAATATAAAAT |
| --- | --- | --- | --- |
| 12 | GAAAACCAAGTCGTGTCTTCGACAAC | 11 | AAATTGCGTTTAGAAC |
| 12 | ACTTGTGTGAATGTCACACACA | 11 | TTTTGTCTTT |
| 12 | TTTTTTTACTTTA | 11 | TTTCTTTTAAAAAAAACCTGATTTGATCAAA |
| 12 | AAATTTATCAAAACCAAACACCAACT | 11 | TAAAATATAAA |
| 12 | TTTTTGATTTTT | 11 | AATATCTCACACGACTCATGTG |
| 12 | TGAATTTCAGATATCTGAAA | 11 | TGTTTCAAACAACTTGTGTGAA |
| 12 | TTTTCATTTTTATT | 11 | TAAATAAAAT |
| 12 | CTAAAATTCAATTTCAGATAT | 11 | TTAAATATAAAAATATTCTTAA |
| 12 | AAAACAAATTAA | 11 | ATATCTTAAATTAATTTCAAG |
| 12 | TTTTACAT | 11 | ACATGTGTGAATGTCTCACACA |
| 12 | TATTTTTTGCGCCCTTTTTAGTCACGGAT | 11 | TTTTTGGAGTTTTCA |
| 12 | AGCTGTTTTTA | 11 | CTTTGGTAGT |
| 12 | AAATTACTATTAAAA | 11 | GTGTAAATGTCTCACACACTT |
| 12 | CACAAGTTGTGTGACACATTCA | 11 | TTTTAAGATG |
| 12 | TTTTTTAATTTAAATAAT | 11 | GTCACGGATTATTTTTGCGCTCCTTTTA |
| 12 | TTTACGTTA | 11 | TATTTTTGTTTAATTAA |
| 12 | CGTGTTAATGTCTTACACGACT | 11 | TTTTTTTTGTAC |
| 12 | GTTTTCAATATCGAGGACACTGACTTG | 11 | AAACCAAACCCCAACTAATTAACCA |
| 12 | CAAAAACTAGAGCATTTTTT | 11 | GACTTGGTTTTCGTTGTCAAGTACACT |
| 12 | TGAATGTCTCACACGACATGTG | 11 | TCTTCTTCAGAAGTCTGATCA |
| 12 | TAGTTTCCGAAAAACTGCTC | 11 | TTCCTCAGAC |
| 12 | TAAATTTAAGATATCTTTAAT | 11 | TATTTAAAAG |
| 11 | CTATTG | 11 | TTTTTCG |
| 11 | AATTCCAAAAATTATATTTTGTTGTA | 11 | ATTCACACAAATCGTGTGAAAC |
| 11 | AGATAAAAATCCCGAATT | 11 | ACATTCACACAAGTTGAGTGAG |
| 11 | TTTTTTAATTGAAT | 11 | GTCACGGAATATTTTTTGCGCTTTTTTA |
| 11 | ATAAAGATAAAAA | 11 | GAGTCGTGTGAGACATTCACAA |
| 11 | TTTTTTGCGCTCTTTTTAATCACGGATA | 11 | AATGTCTCACAGGACTTGTGTG |
| 11 | AAAACATAA | 11 | CGTGTGAATGTTTACACGACT |
| 11 | ATTCACACAAGTTGTGCGAGAC | 11 | AAAAAGGCAATAATTAAAATATAAC |
| 11 | ACGGATTATTTTTTGCGCTCTTTTAGTA | 11 | AGATCGGAAGAGCACACGTCTGAACTCCAGTCCC |
| 11 | TCATCT | 10 | GTGTAAATGTCTCACACAACTTGTGTGA |
| 11 | GAC | 10 | TCTAAAATTCAATTTTTGATA |
| 11 | TATTATATA | 10 | TTAAAATATT |
| 11 | GACACTGACTTGGTTTTCGATGTCAA | 10 | TTTTGTAATAATACTTTATTG |
| 11 | ATAAATATTTATTAATTA | 10 | TATATATTTAT |
| 11 | TTTATTTATTTATTTAATT | 10 | AGTTCTGTGAAACATTCACACA |
| 11 | TCACACAAAATGTGTAAGACAT | 10 | TAAACCAAAACCAAACCCCAACAAAA |
| 11 | ACCAAAACCAAACCCCAACTAAATTT | 10 | TTTTTTTTTTTAAT |
| 11 | TTTTAAATACATTTTA | 10 | TATTTCT |
| 11 | GCCTATGGCTAAGAACATCGCTTCTCA | 10 | AAATAAATATCAAAAATCCAG |
| 11 | CAGACGTCTAAAATACAATTT | 10 | AGATCGGAAGAGCACACGTCTGATCTCC |
| 11 | ACAACTTGTGTGATTCAC | 10 | GAAAACCAAGTCAGTGTACTCGATGC |
| 11 | AATGTCTCACACGACCTGTGTA | 10 | ATTGCTGTCACCGATA |
| 11 | TTTATTTATTTATT | 10 | TTTTTTTAG |
| 11 | ATTTTTTTAAA | 10 | AAAAAAATAAATT |
| 11 | TATCACCAG | 10 | AAATTAAAAATT |
| 11 | AACAAAAAAAATCGAAAC | 10 | TAATTTTATTTTT |
| 11 | TGTCTCACACAACTTGCGTAAA | 10 | AATTAAAAAC |
| 11 | TTTTTTACTTTC | 10 | TTGTGTGAATGTCTCACACAT |
| 11 | TCGAAGACACTGACTTGGTTTTCGAAG | 10 | TATTAATATA |
| 11 | CTTCTTTCTTCTACCTCAACTT | 10 | CCCCCCCCCCCCCCCCCACCCC |
| 11 | TAATAC | 10 | TAATATTTTTGTAATACAAA |
| 11 | AATAAATAAAAT | 10 | AATTAATTATTAATATTAA |
| 11 | AAATTCAATTCAAGATATCTA | 10 | GTGTGAGACATTCACACGAATC |
| 11 | TTTATTTTTTTTTTAAC | 10 | TCTTTGTAAAATTTA |
| 11 | AAAAAGTA | 10 | TTTTTTAAATATTT |
| 11 | TTTTTGC | 10 | TATCTATAATTAAATTAAAGA |

| 10 | GACTTGTGTAATTCACAC | 10 | TCAATTTTCAATACTTT |
| --- | --- | --- | --- |
| 10 | ATTTATAAATTAA | 10 | TATAGTTTTAAGTCGCAA |
| 10 | TTCACACAAAATCGTGTGAGACA | 10 | TTCTTTTTTCTAACAATTATT |
| 10 | AAATTAAACTTAA | 10 | GTGTGAATGTCTCATACAACTT |
| 10 | TTTTTTTATTATT | 10 | CCAGCATC |
| 10 | TTATTTCTTCCTTTAAAAATAA | 10 | TCACGGATAATTTTTGCGCTCTTTTTAG |
| 10 | ACAATTTTTTTATATTT | 10 | ATACATAA |
| 10 | TATTTTCAG | 10 | CGAAAACCAAGTCAGTGTCTCCGACA |
| 10 | ACTTGAGTAAATGTCTCACACG | 10 | TTTGC |
| 10 | AGATCGGAAGAGCACACGTCTGAACTCCATCC | 10 | ATTTTTTTTAATTTC |
| 10 | ATTTTTTTTAA | 10 | ATTTTTTTATTTA |
| 10 | TTGTTTTATT | 10 | CAAAAAAAATTA |
| 10 | ATTCACACGAGTCGTGTGAGAA | 10 | ATAATTATATTTTATAAA |
| 10 | TCTTCTTTATTGTTG |
| 10 | ATAAAATATAAA |
| 10 | AACAAATC |
| 10 | TAATTTTATTTTTTATTT |
| 10 | TTCTTAAGGTTAAG |
| 10 | GTCGAGTACACTGACTTGGTTTTCGAT |
| 10 | ATACTGA |
| 10 | AAAAATATTTTATATTTTTT |
| 10 | TAAAAAGAGCGCAAAAAATAAACCGTGAC |
| 10 | TGTGTGTGTGTTTA |
| 10 | ATTTCCCAGG |
| 10 | ATAATTAAAAAATAT |
| 10 | TTGGTTTTCGTCATCAAGACACTGAC |
| 10 | GACTTGGTTTTCGTAGTCGAAGACAC |
| 10 | TTTTCTGA |
| 10 | ATTTGTTGTCAGTTAGGAATTGTA |
| 10 | TCTCACACGACTTGTGTGAAGG |
| 10 | CGAAAACCAAGTCAGTGTCTTCGACTG |
| 10 | TTAAAATT |
| 10 | CCAAGTCAGTGTAATCGACAACGAAAA |
| 10 | CGTTTAGAACAAATTA |
| 10 | AGATATCTCTAAATGAATTTC |
| 10 | CCAGATCGGAAGAGCACACGTCT |
| 10 | TTTTTGAAATCTCTT |
| 10 | TTTTTCAATTTT |
| 10 | TTTTTGCGCTCTTTTTAGTCACGAAATAT |
| 10 | TATTTTTTTTTAAC |
| 10 | CGACCTGTGTGAATGTCTTACA |
| 10 | ATTAAAAAAAATAATA |
| 10 | GAAAACCAAGTCAGTGTAATCGATGAC |
| 10 | CACGGATTATTTTTTGCGCTCTTTTGAGT |
| 10 | AGTCGTGTGAAACATAAACACG |
| 10 | TTTTTGCGCTCTTTTTAGTCACGGAAAT |
| 10 | GACTCGTGTGAATTCCTCACAC |
| 10 | GGTTTGGTTTTGGTTAATTTTGTTGG |
| 10 | TTGTGTGAATGTCCCACACAAC |
| 10 | GTGTGAATGTCTCACACAACTTA |
| 10 | TTTCTG |
| 10 | CCCCCCCCCCCCCCGCC |
| 10 | TCACACAAGTTGTGTGAGACATTCACAA |
| 10 | TTTTTAGTCACGGCTTATTTTGCGCTC |
| 10 | TTTGGATTC |
| 10 | CTTTTTGTA |
| 10 | ACACAAGTCGTGTGAGACATTCA |
| 10 | GGGGTTCACACGACGCTTGTGATGCCTCTG |
| 10 | GCCCCCC |

| **Bembidion ulkei (#SRR5230416)** | | | |
| --- | --- | --- | --- |
| 44137 | ATAG | 287 | CTATTCGAA |
| 30831 | TTTTAA | 284 | GTGTGAGACATTCACACAAGTT |
| 24414 | AGTTAAA | 282 | AAC |
| 22953 | AAAT | 282 | AGCACACGTCTGAACTCCATCCAGATCGGAAG |
| 15185 | TAACTTA | 278 | AGATCGGAAGAGCACACGTCTGACTCC |
| 10385 | **GGTTA** | 273 | GAATGGAATCGAATG |
| 8459 | TCCAT | 270 | GAGCAGTTTTTGCGAAAACTA |
| 7250 | AGATCGGAAGAGCACACGTCTGAACTCC | 268 | GAAAACCAAGTCAGTGTACTCGATGAC |
| 6373 | ATA | 261 | TTCCATTCGATTCCATTCGATGA |
| 5599 | CTGCTCTAGTTTTCGCAAAA | 257 | AGATCGGAAGAGCACACGTCTGAACTCCAC |
| 5161 | TATTTA | 254 | AAAAATGCTCTAGTTTCCGA |
| 4212 | GAGCAGTTTTTCGAAAACTA | 251 | GAGCAGTTTTCGAAAACTA |
| 3468 | AAGA | 245 | TTTTTATATTT |
| 2529 | AAATTAA | 243 | TTTTTTTTAA |
| 1625 | GTTTTCGCACAACTGCTCTA | 238 | TTTTTTAACTTGAT |
| 1466 | ATGCTCTAGTTTTCGAAAAA | 233 | AGATCGGAAGAGCACACGTCTGAACTCCT |
| 1453 | ATTTAATTTTA | 233 | CTTCTTTTATTT |
| 1251 | AGTTACA | 225 | GAGCAGTTTTGTCGAAAACTA |
| 1242 | TTC | 218 | AGATCGGAAGAGCACACGTCTGATCCC |
| 1188 | ATTATTGCTGTCACCG | 209 | ACAAAAATAATA |
| 1114 | TTTTGCGGAAACTAGAGCAG | 209 | TATG |
| 1069 | AGATCGGAAGAGCACACGTCTGAACTCCC | 205 | AGATCGGAAGAGCACACGTCTGATCTCC |
| 975 | AATATAAAATAA | 205 | AAAAGCAA |
| 952 | AACA | 202 | TAATTTTTAT |
| 872 | AAAAT | 202 | AAATAAAAT |
| 821 | GAAG | 201 | TAATTTTTATTTGTT |
| 792 | TTTTTGCGCTCTTTTTAATCACGGATTAT | 200 | GGGGAGGG |
| 747 | TTTATATTTTA | 199 | TTTTTGCAAAACTGCTCTAG |
| 608 | GCAGTTTTGCGAAAACTGGA | 198 | GTTTTCGCCAAACTGCTCTA |
| 569 | AAAACTGCTCCAGTTTTCGA | 197 | TTAG |
| 545 | GAGCATTTTTGCGAAAACTA | 197 | AGAGCACACGTCTGAACTCCATCAGATCGGA |
| 533 | AATATATA | 190 | AATAATAAAGAT |
| 510 | CTGGAGCATTTTTTCGAAAA | 189 | TTTTCATG |
| 503 | AGATCGGAAGAGCACACGTCTGAACTCCAGTCC | 188 | TAGAGCAGTTGTTCGAAAAC |
| 489 | CGAAAACTAGAGCAATTTTT | 185 | TTTTCGAAAAATTGCTCCAG |
| 477 | TTGCTCTAGTTTTCGAGAAA | 182 | TGATGATTCCATTCGATTCCATTCGA |
| 473 | GCGAAAACTGGAGCAATTTT | 179 | CCAGATCGGAAGAGCACACGTCT |
| 452 | AGATCGGAAGAGCACACGTCTGAACTCCAGTC | 173 | ATAATATAATTATATAT |
| 446 | AGCAGTTTTGCGGAAACTGG | 171 | AAAAAG |
| 441 | TGCTCTAGTTTTCGCAAAAT | 170 | CTTGTGTGAATGTCTCACACGA |
| 425 | CAGC | 170 | ATAAATA |
| 425 | AGATCGGAAGAGCACACGTCTGAACTC | 170 | CGAAAACTAGAGCAGTTTCG |
| 418 | AAAAATAATAAA | 167 | TTTTAACTTTATTC |
| 405 | GACAGA | 164 | TGCTCTAGTTTTCGAAGAAA |
| 392 | TTTTCGACAGTTTTGCTCCAG | 160 | AATTGAATTTCAGATATCTGA |
| 390 | TTTAAA | 159 | TAAAATATAAAAT |
| 381 | AAACA | 159 | TAAATAATAAA |
| 379 | TTTTTA | 159 | CTTTC |
| 374 | AAAAAC | 159 | ATATTAATA |
| 357 | TTTCCGAAAAACTGCTCTAG | 157 | TCTCAGATCGGAAGAGCACACG |
| 353 | TTCTT | 156 | TTTTTTTAACTT |
| 341 | TCCCTC | 156 | TTTTCGCGAAATTGCTCTAG |
| 321 | AACTTAA | 155 | TCCCCCC |
| 314 | TTTTATATTATA | 154 | TTTTTTAT |
| 309 | CCGAAAAAATGCTCCAGTTT | 153 | CAAACTTCCGAAAACTAGAG |
| 303 | CTC | 153 | ATGATTCCATTCGATTCCATTTGATG |
| 299 | TTTTTGCGCTCTTTTTAGTCACGGATTAT | 152 | CCCCCTCCC |
| 288 | ACTAGAGCATTTTTCGAAA | 151 | TTTTCGTCAGTTTTGCTCCAG |

| 151 | GTTTCCGAAAAACTGCTCCA | 91 | AGATCGGAAGAGCACACGTCTGAACCCC |
| --- | --- | --- | --- |
| 151 | TAAAAATAAAA | 91 | TCCAGATCGGAAGAGCACACGTCTGAACTC |
| 150 | CTTCTTT | 90 | TTTTCAA |
| 147 | GAGA | 88 | AGATCGGAAGAGCACACGTCTGAACTCCAGTCCC |
| 147 | AGATCGGAAGAGCACACGTCTGAACTCCACC | 87 | TTTTAAGTCGCGTTAG |
| 147 | AGATCGGAAGAGCACACGTCTGCTCC | 87 | AAAACAAAAA |
| 145 | CTCTAGTTTTCGAAAATTTG | 87 | CCCCTCCCCCCC |
| 144 | ATTTCAGATATCTTGAATTGA | 86 | TAGAAGATA |
| 144 | CACGGAATATTTTTTGCGCTCTTTTTAGT | 85 | AGCAATTTCGCGAAAACTGG |
| 143 | ATCCCAGATCGGAAGAGCACACGTCTGAACTCC | 84 | TTTTTTTTTC |
| 142 | GATTCCATTC | 83 | ATAAACAA |
| 142 | CTCCCCCCCC | 82 | TTCAATTTGAGATATCTGAAA |
| 141 | TGA | 80 | TATAGTTGCTGTATACGAACACA |
| 141 | GAAAACTAGAGCAAAACTGTC | 80 | AGATCGGAAGAGCACACGTCTGCC |
| 138 | CCCCTC | 80 | TGACTTGGTTTTCGTTGTCGAAGTACAC |
| 136 | AGTGG | 80 | CCAT |
| 133 | CCGTGATTAAAAGAGCGCAAAAAATAAT | 80 | AAATGCTCCAGTTTTCGAA |
| 132 | CTCTCAGATCGGAAGAGCACACGTCTGAA | 79 | GAAAACCAAGTCAGTGTACTCGACTAC |
| 131 | GTTTTCGAAAATTGCTCTA | 77 | TTTTTTGCGCTCTTTTTAGTCAGGGATTA |
| 131 | AGATCGGAAGAGCACACGTCTGAACTCCTCC | 77 | TTGCTGTCACCAATTA |
| 129 | TTCAAATTCTTC | 77 | AGATCGGAAGAGCACACGTCTGTCC |
| 128 | CCCCCCCTCCC | 76 | GAAGGATGGAAGAGAAGGTCGAAGAAACTTCCA |
| 125 | AGATCGGAAGAGCACACGTCTGAACTCCTC | 76 | AAAAAAAAATT |
| 125 | AATAAAA | 75 | TCTTCTAAAA |
| 124 | TATTTTTTGCGCTCTTTTTAATCACGGC | 75 | AAAAATAAT |
| 122 | CCCAGATCGGAAGAGCACACGTCTG | 74 | AGATCGGAAGAGCACACGTCTGAACTCCA |
| 122 | GCAAATGTTCGAAAACTAGA | 74 | ATATATA |
| 122 | GTTTTCGAGAAACTGCTCTA | 74 | GCTCTAGTTTTCGGAAATTT |
| 122 | AGTTTTCGCACAACTGCTCC | 74 | CCAGATCGGAAGAGCACACGTCTGACTC |
| 121 | TATTCGAA | 74 | AAAAAAATAA |
| 120 | CAACAACTA | 74 | GTCCTTGCTC |
| 119 | AGTTTTCGCAAAAATGCTCC | 73 | TGC |
| 117 | CGTTTAGAACATTTTG | 73 | TTTTTCAAATTC |
| 117 | TTTTCGACAAAATGCTCTAG | 73 | TTTGTTTTT |
| 116 | AAAAATAGCCGTGACTAAAAAGAGCGCA | 73 | AACC |
| 115 | AGATCGGAAGAGCACACGTCTGAACCC | 72 | TCCCAGATCGGAAGAGCACACGTCTG |
| 114 | GTTTTTGAAAAAATGCTCTA | 72 | AAGTTAAAAGAAAC |
| 114 | AGATCGGAAGAGCACACGTCTGAACTCCAT | 71 | TAATTTTGCTCAACA |
| 113 | CTAGAGCATTTGTTCGAAAA | 71 | AAAAATAATCCGTGACTAAAAAGAGCGC |
| 113 | CCTTTT | 71 | GTAC |
| 113 | ACCCAGATCGGAAGAGCACACGTCTG | 70 | AGATATCTCGAAATCAATTTA |
| 112 | TCTTC | 70 | AGATCGGAAGAGCACACGTCTGAACTTCC |
| 108 | ATTTTTTAACTTT | 70 | GAAAAAA |
| 106 | GATCCAGATCGGAAGAGCACACGTCT | 70 | ATTTGTCCTTAAAAATTTCATATA |
| 104 | TTTTTCCTTTTC | 70 | AGATCGGAAGAGCACACGTCTGAACTCCAGTCAC |
| 101 | GAATTTCAGATATCTTAAATT | 69 | AGACATTCACACGAGTCGTGTG |
| 100 | TCTCTCTT | 69 | TGGCTAAGAACATCGCTTCTCGGCCTA |
| 99 | ACCCTA | 69 | TTAGTTATATTAAC |
| 99 | TTGATGATGATTACATTCGATTCCAT | 67 | AATATAAAATAAA |
| 99 | AAATAAAAAT | 67 | TTTTTTATA |
| 97 | GAATGGAATCATT | 66 | TTTCTTGC |
| 96 | TCGACAACGAAAACCAAGTCAGTGTAC | 66 | TATATATATATAATTTAT |
| 94 | CCAGATCGGAAGAGCACACGTCTGAACTCC | 66 | CCTTTTCTTCTCTCCTTC |
| 94 | AAAAGAAA | 65 | TGCTCCAGTTTTCGAAGAAA |
| 93 | AACTAAATAAACCAAAACCAAACCCC | 65 | AGATCGGAAGAGCACACGTCT |
| 92 | ACTGCTCTAGTTTCCGCACA | 64 | CCAGATCGGAAGAGCACACGTCTGA |
| 92 | TTCCTC | 64 | AAATTTT |
| 92 | GAAT | 64 | CCCCCCCTCCCCC |
| 92 | AATATAATAAAAA | 64 | CCATTCGATGATT |

| 64 | TTCCATTCGAGTCCATTCGATGA | 50 | CTGTCACCAAATATTG |
| --- | --- | --- | --- |
| 64 | GCGA | 49 | TTGCTCCAGTTTTTGCAAAA |
| 63 | ATTATTCATCTCTCCG | 49 | TCCAACTC |
| 63 | AGTTATATTAACTA | 49 | TTCCATTCGAGTCCA |
| 63 | AGATCGGAAGAGCACACGTCTGAATCTCC | 49 | AGATCGGAAGAGCACACGTCTGAACTCCACTC |
| 63 | GCTCTAGTTTTCGCACAATT | 49 | ATTCCATTCGATTCCATTCGATGAT |
| 62 | GTCAGTGTCTTCGATGACGAAAACCAA | 49 | TGTTTTT |
| 62 | ACGAAAACCAAGTCATGTCTTCGACA | 48 | TTTTTTGCGCTCTTTTTAGTCACGGATA |
| 62 | AGATCGGAAGAGCACACGTCTGAACTCCAGTCT | 48 | AAGAAAGG |
| 61 | TAGAGCATTTTTCCGAAAAC | 48 | GTTTTCGAAAAAACTGCTCTA |
| 61 | TTTTTTTTTGT | 48 | ATTTTTTTTTT |
| 61 | TTTTTTTAACTTT | 48 | GCATCT |
| 61 | TTAGGAAAAATTGCGT | 48 | AACCAAACCCCAACTAAATTAACCAA |
| 59 | ACACAACTTGTGTAAATGTCTC | 48 | ACATATAT |
| 59 | GTCT | 47 | TTGAAAACCAAGTCAGTGTACTCGATG |
| 59 | TTTTTTTAAACTTTATTCTTTTAACTTT | 47 | AGATCGGAAGAGCACACGTCTGATCTC |
| 59 | AGATCGGAAGAGCACACGTCTGAACTCCATCTC | 46 | CAGATCGGAAGAGCACACGTCTGACT |
| 59 | TTTCTTTTT | 46 | AAATTGCTCCAGTTTCCGCA |
| 59 | ATTATATATAAAATTTAT | 46 | TTTATTTTC |
| 58 | ATCA | 45 | TTTTTGAA |
| 58 | TCTTTC | 45 | TGACTTGGTTTTCGTGTCGAGACA |
| 58 | TCACGGATTATTTTTTGCGCTTTTTTAA | 45 | CGAAAACCAAGTCAGTGTCTT |
| 58 | AAAAAATAATCCGTGATTAAAAAAAGCGC | 45 | TTTGTTTTAA |
| 58 | TCTCTC | 45 | ACACG |
| 58 | TTTTCTC | 45 | CTTTTTAATCACGGAATATTTTTTGCGCT |
| 58 | ATTTTCTTTTTAACTTTATC | 45 | TTTTTGCGCTCTTTTTATTCACGGAATAT |
| 57 | AGATCGGAAGAGCACACGTCTGATCTCCC | 45 | AGATCGGAAGAGCACACGTCTGAACCTCC |
| 57 | AAAACTAGAGCAGTTTTCCG | 45 | CTCCC |
| 57 | ATTTTTTGATGTTTC | 44 | GATGATTCCATTCGAGTCCATTA |
| 57 | TAGATATCTCGAAATCAATTT | 44 | CGATGATTCCATTCAAGTCCATT |
| 57 | AGATCGGAAGAGCACACGTCTGATCCTC | 44 | AAAAAGAGCGCAAAAAATAATCCGTGAAT |
| 56 | TAAAATTAAAA | 44 | TTCTA |
| 56 | GATTCCATTCAAGTCCATTCAAT | 44 | AAGAGCACACGTCTGAACTCCACTCCAGATCGG |
| 56 | AACTTATCTTTGAG | 44 | TGCTCTAGTTTTCGACAAAAA |
| 55 | CATTCCATTCCACTC | 44 | ATGATTCCATTCGATTCCATTCA |
| 55 | CTCC | 44 | TTGCTGTCACCGAATA |
| 54 | CACGTTA | 43 | TTTAAAGTAGT |
| 54 | TAAATTTTATA | 43 | AGATCGGAAGAGCACACGTCTGAACTCCATTCC |
| 54 | CCAGATCGGAAGAGCACACGTC | 43 | GATCGGAAGAGCACACGTCTGAACTCCACCCA |
| 54 | CTTCC | 43 | AAATGCTCTAGTTTCCGCAA |
| 54 | AAAAATGCTCTAGTTTCCGT | 43 | TTCTCTTCCTCTTC |
| 54 | TATA | 43 | TTGCTCCAGTTTTCGCACAA |
| 54 | TTAAATAAAAATTTTAT | 43 | TCTCTCTGTC |
| 53 | TACATAAAATATGAAAG | 42 | TGATTCCATTCGATGA |
| 53 | TCCTTC | 42 | CTCTAGTTTTTGCAAAATTG |
| 53 | TTTATCTA | 42 | TAAAATTTA |
| 53 | ATATCTTAAATTAATTTCGAG | 42 | CAATTGCTCCAGTTTTCGCG |
| 53 | AGATCGGAAGAGCACACGTCTGAATCCC | 42 | CGCTCTTTTTAGTTACGGATTATTTTTTG |
| 52 | CCGATTATTGCTGTTA | 42 | ATTATTTTTGCGCTCTTTATAGTCACGG |
| 52 | GTTTTGCTCCAGTTTTCGAA | 41 | CTTTTTTTTTT |
| 52 | CCATTCCACT | 41 | ATCTAAATTCTAAATAATAATG |
| 52 | CAAAAAGA | 41 | AAATAAATAAAAAT |
| 51 | TTGCGTTTAGAAAATT | 41 | CAGATCGGAAGAGCACACGTCTCC |
| 51 | GAAAACTAGAGCAAAACTGCC | 41 | GTTTTTGAAAAACTGCTCTA |
| 51 | AGATCGGAAGAGCACACGTCTGAACTCCAGTCCT | 41 | GTTTTCA |
| 50 | AGATCGGAAGAGCACACGTCTGAACTCT | 41 | TTTTTAACTTTATT |
| 50 | ATTCCATTCCATTAG | 40 | TAAACTTTATACTTA |
| 50 | CTGTCACCGATTTTTG | 40 | ATTCCATTCATTTG |
| 50 | TCACGGACTATTTTTTGCGCTCTTTTTAA | 40 | AAAACCAAGTCAGTGTCTTCGACAACG |

| 40 | TTTTTTTTTTCTACTTGCATTGCCGTTACC | 33 | CTATATTTT |
| --- | --- | --- | --- |
| 40 | TTTTTTAACTT | 32 | GAATATTCGAATAGTTC |
| 40 | TTCTTCTCCAGTTAAACATAGA | 32 | CATTCGATAATTCCATTCGATTG |
| 39 | TGCTCTAGTTTTCGCAGAAAC | 32 | TAACAAATTAC |
| 39 | TATTTTTTGCGCTCTTTTTAATCACGGA | 32 | AGATATCTGAAATTCAATTTT |
| 39 | TTTTCA | 32 | ATATA |
| 39 | AGGAG | 32 | TCACGGAATATTTTTTGCGCTCTTTATAG |
| 39 | TGCTCTAGTTTTCGGAAAAT | 32 | TAATTTTATA |
| 39 | GAGTGGT | 32 | ATTATTTTTTGCGCTCTTTTTAATGCACGG |
| 39 | TGCTCCAGTTTTCGACAAAA | 32 | AGAAGGAAGGAA |
| 38 | AGATCGGAAGAGCACACGTCTGAACTCCATCCT | 32 | TATTTTTTGCGCTTTTTTTAATCACGGAC |
| 38 | TGCTTC | 32 | GCATTTTTGCCAAAACTAGA |
| 38 | AGATCGGAAGAGCACACGTCTGAACT | 32 | TTCTAATATCTTAATATAAATATAT |
| 38 | AGATCGGAAGAGCACACGTCTGC | 32 | GTCCATTCTATTCCC |
| 38 | AAGCAA | 31 | AAGC |
| 38 | TTTTCTTCCGTTCCGTTC | 31 | TTTTTTGTTTTAATT |
| 38 | TTTTCAAGAAATTGCTCTAG | 31 | TTCCTACTTTTCTGAT |
| 38 | AAAAAAGAAAAAAATTT | 31 | GATTATTTTTTGCGCTCTTTTTATCACG |
| 38 | TATTCCTA | 31 | CCCCCCCTTCC |
| 37 | AGATCGGAAGAGCACACGTCTGAACTCCAGT | 31 | TTAATAAT |
| 37 | AATGGAATCATCAAATGGACTCG | 31 | ATTAAAATAAAATATAA |
| 37 | CTTTTTGTA | 31 | TATTTTTTCATTTTTTA |
| 37 | GCTATTTTTTGCGCTCTTTTAAGTCACG | 31 | CCCCCCCCTT |
| 36 | AAGGTTAAGTTCTT | 31 | TTTTTTTTACTATA |
| 36 | AGATCGGAAGAGCACACGTCTGACCTCC | 30 | TTTAAATATTAATTTTAACAAATA |
| 36 | TGCTCCAGTTTTCGGCAGTTT | 30 | CCCCCCCCCCCCCCTC |
| 36 | GTAGAAT | 30 | CAGATCGGAAGAGCACACGTCTGAT |
| 36 | ATGATTCCATTCGATTCCATTCTATG | 30 | AGATCGGAAGAGCACACGTCTGAACTCCAGC |
| 36 | AAAACTGCTCTAGTTTTCAA | 30 | AAAATAAAAATAAATT |
| 36 | TGCTCTAGTTTTCGGAAGTTT | 30 | AATAAGTATAATAATAAATA |
| 36 | TTCG | 30 | TATATTT |
| 36 | ATTTTTTTT | 30 | CTTCCCCCCCCC |
| 36 | TGCTCTAGTTTTTGAAAAA | 30 | CTGTTTCAGAG |
| 36 | ATATATTGTATGTTATATATC | 29 | TTTTTATCTTTATT |
| 36 | CCCCCCCCCTCCCC | 29 | AGATCGGAAGAGCACACGTCTGATTCC |
| 36 | AGATCGGAAGAGCACACGTCTGATCCTCC | 29 | AAGATATCTTAAATTCAATTT |
| 35 | TTTTTGCGCTCTTTTTAATCACGGATTT | 29 | CTCTGTCT |
| 35 | CGAAAACCAAGTCATGTCTCGACAA | 29 | GTG |
| 35 | TTCGATTCCATTCAATGATGATTCCA | 29 | TATTATATATAATATA |
| 34 | TTTTTATTATT | 29 | TTTCCCT |
| 34 | ATATATATAA | 29 | AGATCGGAAGAGCACACGTCTGATTCCC |
| 34 | CATTCGATGATTCCATTCGATTG | 28 | TCCCCCCCTC |
| 34 | TGGGGCTAGGGTTTAGGTTAGGTGGTGGTGG | 28 | ATTTTTTTTTAT |
| 34 | AATTTCT | 28 | TCGGAAGAGCACACGTCTGAACTCCAGTCTCAGA |
| 34 | CTA | 28 | TTAAAACAGATTTCGACTATTTTAAT |
| 33 | ACCAAACCCCAACTTAATAAACCAAA | 28 | CTCTAGTTTCCGCGAAACTG |
| 33 | CGGAAGAGCACACGTCTGAATCCAGAT | 28 | ATAAATAAAT |
| 33 | TATTTTTTGCGCTCTTTTTAGTAACGGAT | 28 | AATCAGCTCTCTGTAAAACGGACC |
| 33 | TTTTCGAATAAATGCTCCAG | 28 | ATCATCAGAATGGAATCGAATGGA |
| 33 | CACCAG | 28 | ATATCTGAAATTAAATTCCAG |
| 33 | ATTATA | 28 | GTGCAGA |
| 33 | CTGACCTAAA | 28 | ACCACAC |
| 33 | TTTTCGCACAAATGCTCCAG | 28 | TCTTCCTAACTCTTCTAAAA |
| 33 | ATATATACAT | 28 | TTTTTTTTTCTACTTGCATTGCCGTTACC |
| 33 | TTCGAATATTCAATCTA | 28 | GGAATCATCATCAAATGGAAACGAAT |
| 33 | TCTAGTTTTTGCAAAAATGC | 28 | ATTGCTTCAAAGTCTTGTAAACTCTTCTAA |
| 33 | TTTTAACTTTATATTT | 27 | TGGAATGCAA |
| 33 | AAAGCTGTTTC | 27 | TTTATATTTTATTCG |
| 33 | TGTTCCGGTTTCTCG | 27 | CTGCTGA |

| 27 | TTGCCGTTACCTTTTTTTTTTTCTACTTGCA | 25 | GCGTCCTCATCAGCACC |
| --- | --- | --- | --- |
| 27 | AGATCGGAAGAGCACACGTCTCTC | 25 | TGAGCGGTTTC |
| 27 | TTTCTTTTCTTTC | 25 | TTTTTTATTTATTTATTTTA |
| 27 | CTTTTTAATCACGGTTATTTTTTGCGCT | 25 | TACGAAACCCATCAGGCTGT |
| 27 | AATTTTTTTGTTTAT | 24 | AGATCGGAAGAGCACACGTCTGAACTCCATCT |
| 27 | CCAAAACCAATAGAACAA | 24 | ATTTCAA |
| 27 | AGATATCTTAAATTCAATTTC | 24 | AACTTTCATATTTTTTT |
| 27 | TAAAATAAAATATTATA | 24 | TTATTTTGA |
| 27 | TTAATTGATTACT | 24 | AGGAAGAAGAGAGAG |
| 27 | TCCCCCCCCCCCCCC | 24 | ATCATCGAATGGAAACGAATGGAATC |
| 27 | CAGACTACTAACTA | 24 | CCAGATCGGAAGAGCACACGTCTCT |
| 27 | CTTGTGTAAATGTCTCACACGA | 24 | ATGTCTCACACGACTTGTGTTA |
| 27 | AAAAATGCTCTAGTTTCCG | 24 | CCCCCCCCTCT |
| 27 | AGATCGGAAGAGCACACGTCTGAACTCCAGTCACTCC | 24 | TTGCTCTAGTTTTCGATAAAA |
| 27 | AATTTAATTTGAGATATCTAA | 24 | TGTTCCTTAGCTCTAT |
| 27 | AGATCGGAAGAGCACACGTCTGAACTCCAGTCACC | 24 | GAAAAAAAAAAA |
| 27 | AGATCGGAAGAGCACACGTCTGTCTCC | 24 | GAATCATCATCAAATGGAATTGAATG |
| 26 | TTCAAAA | 24 | AGGTTT |
| 26 | GCTGACTTTGTGTCT | 24 | GAGCAAAATTATGAT |
| 26 | ATCGGAAGAGCACACGTCTGACCCCAG | 24 | CGGCTT |
| 26 | GGGAGAGA | 24 | GTCTTTCCTTTCTTCCT |
| 26 | AAATGCTCTAGTTTTCGCAC | 24 | CGAGCA |
| 26 | TTTATTTTTGTTTAA | 24 | TATAATTTATATATAA |
| 26 | GACACGACACGT | 24 | TCCCTTTTCTATGAACTCA |
| 26 | TCTAGTTTTTGAAAATATGC | 24 | TATATACACA |
| 26 | AAAGAAGG | 24 | AGATCGGAAGAGCACACGTCTGAACTCCACT |
| 26 | TTTTTTGCGCTTTTTTTAATCACGGCTA | 24 | ATATATATATATATAA |
| 26 | AATTAACGGTTTAATATTCA | 24 | AAATTTCATATAATTTGTCTTTAA |
| 26 | AGATCGGAAGAGCACACGTCTGAATCTCCC | 24 | TTTTAATTTTTTTAATTTTTTTTTAATTTG |
| 26 | ATTTTTTTAAAG | 24 | GAATGGAATCAAATG |
| 26 | GATTATTTTTTGCGCTCTTTTTAATCACA | 23 | CTGACCTAAT |
| 26 | CATTCCATTCGACTC | 23 | CATTCGATGATTCCATTCAATTC |
| 26 | AAAAAAATAC | 23 | CCTGCTCTCAGCTCCTTA |
| 26 | AGATCGGAAGAGCACACGTCTGAATCCTCC | 23 | CTTCTCCTT |
| 25 | TTTTTGCGCTCTTTTTAGTCACGGCTAAT | 23 | ATTCCATTCGTGATGATTCCATTCA |
| 25 | TTTTTGTTTTTT | 23 | GTTTTTTGTTT |
| 25 | TTTAAGATTT | 23 | TTTATTCTCAATATATCGAC |
| 25 | AACCAAGTCAGTGTCTCGACAACGAA | 23 | TTTTGGCCCGAAATGCTCTAG |
| 25 | TTTTTTAAATTTTT | 23 | GTCAGTATAG |
| 25 | TGTGTGGTG | 23 | GATGATTCCATTCAATTCCATTCGAT |
| 25 | ATTCGAGTCATTTCC | 23 | CCCCCCCTT |
| 25 | TTCTTC | 23 | AGCACACGTCTGAACTTCCCAGATCGGAAG |
| 25 | ATTCCATTCGATTCCATTCGATA | 23 | AAAATATAAA |
| 25 | TTAAT | 23 | AAAAATGCTCTAGTTTTGGA |
| 25 | TATCTTAATATAAATATAATTCTAA | 23 | CATTCCATTCCAGTA |
| 25 | ACCCGT | 23 | CTTGTGTGAATGTCTTACACGA |
| 25 | TTCATCTCCTCGATTA | 23 | GAGAGGG |
| 25 | ATATAATAAAATAAA | 23 | GATGACTCCATTCGAGTCCATTA |
| 25 | TAACAC | 23 | AGCTCTGACGCATCG |
| 25 | CCAGATCGGAAGAGCACACGTCTT | 23 | ACACATACATATAT |
| 25 | TATATATATAAA | 23 | GATTCCATTCGACTCCATTCGAT |
| 25 | TTTCGGCCAAAATGCTCTAGT | 23 | CTTGGGCGATTC |
| 25 | GTCTTTTCCTC | 23 | GATCGGAAGAGCACACGTCTGAATCCCCA |
| 25 | CATTGT | 23 | TTTTTTAACTTGTT |
| 25 | AATTCAATTTAGATATCTGA | 23 | TTCCATTTGATGACGATTCCATTCGAA |
| 25 | CAACAACAGCACAGC | 23 | TTCGCCCGC |
| 25 | AATAC | 22 | TTTTTTTAACTA |
| 25 | TTTTTTTTGTCTTAA | 22 | CGGATTTTTTTTGCGCTCTTTTTAGTCA |
| 25 | ATGATGATTCCATCATTTCCATTCG | 22 | CAATAAAGAAGAAATCCAACAAAGAAGACAA |

| 22 | TTTTTTTAA | 20 | CTCTTCTTAA |
| --- | --- | --- | --- |
| 22 | CGTTTAGGAAAAATTA | 20 | AGATATCTCGAAATCAATTCA |
| 22 | GTTCAAATTCAG | 20 | GAATCGAATGGAATCATCGCAAATG |
| 22 | TTTTTTTATTTATT | 20 | ATATCGATAA |
| 22 | TTGATATGGTTGTTG | 20 | CCCTCCCCCCCCCCCCC |
| 22 | TCCCCCCCCCTC | 20 | CTACAT |
| 22 | ACTGGGATAAACATA | 20 | AGATCGGAAGAGCACACGTCTGAACC |
| 22 | ATTCTTATC | 20 | GAATATTTTTTGCGCTCTTTTTATTCACA |
| 22 | TTTCGCAAAACTGCTCTAG | 20 | TGCTCTAGTTTTCGCAGAAAT |
| 22 | CATTGGCGCCATGGCG | 20 | ATATATAAAA |
| 22 | TTTAGTT | 20 | TCATCAAATGGAATCGAAAGGAATCA |
| 22 | TTTTGG | 20 | TATTTCCGAA |
| 22 | GTTTCTGATAAAAAT | 20 | TAAAATATATATTCATGTGTTGAAG |
| 22 | TCTCCTCT | 20 | TTTTATTTTAATTTTTTTTA |
| 22 | ATCGGAAGAGCACACGTCTGACCTCAG | 20 | TATTTTTTGCGCTCTTTTTAGTCACGGT |
| 22 | TTTTAATTTATA | 20 | AGACAAAC |
| 22 | ATCCAAGAAGATGTCGAAGAG | 20 | TTTAATTTATTTTGAAAATAA |
| 22 | GCCGACTCTC | 20 | CAAAACTGCTCTAGTTTTCA |
| 22 | GGAAGG | 20 | ATTTTTTGCGCTCTTCTAGTCACGGAT |
| 22 | AATGTCTCACACGACTCGTGTA | 20 | AAATAAATATA |
| 22 | GGGACACTATGATGAGGCCAGGTGAGTGCACACT | 20 | AATGAAATGGAATCG |
| 21 | GTTTTCGAGAAAATGCTCTA | 20 | TTTTTTGCGCTCTTTTTAGTCACGGCT |
| 21 | TCCCCCCTC | 20 | CCTCCTGCTCTCGGAGCATCT |
| 21 | GGGTAAGCCGAGGCTTGTTCCTGGTCC | 20 | TTTGCTCTAGTTTTCGAAGA |
| 21 | GAATTATTTTTTGCGCTCTTTTTAGTCAC | 20 | CCCCCCCCCCA |
| 21 | TGCTCTAGTTTTGAGAAAC | 20 | TCTAGTTTTCGAAAAAATGCT |
| 21 | AGAAC | 20 | ATGGAATCATCGAATGGACTGGA |
| 21 | TATATACACACA | 19 | AATACA |
| 21 | CTCTAGTTTTCGACAGAAATG | 19 | ATGGACTCAAAGAGA |
| 21 | TCTCTCTC | 19 | AACAAAATAA |
| 21 | CAGATCGGAAGAGCACACGTCTGT | 19 | ATTTTTGTTTTAATTT |
| 21 | AGTTTGCATTAGCAAAT | 19 | TATAGGTTTAGACTAAGGT |
| 21 | TCAGATCGGAAGAGCACACGTCT | 19 | ATTATATATA |
| 21 | TTTTTGCGCTCTTTTTATGCACGGAATA | 19 | TTCAT |
| 21 | CTTCATG | 19 | AGATCGGAAGAGCACACGTCTGATACTCC |
| 21 | GGAATCGAATGGAATAATCATCGAAT | 19 | CAAACT |
| 21 | AATTATTTAACTA | 19 | GGTTTGAAGTAGTTTGAAAT |
| 21 | GAATGGAATCGAATGGAAACATCATG | 19 | AATGGAATAATCGAATGGACTCG |
| 21 | TCGGAAGAGCACACGTCTGAACTCCATCTCCAGA | 19 | GTAATTC |
| 21 | TCCCTTTTCTTTCTTCCT | 19 | TTTTGTTTTTTTCTTC |
| 21 | CAGATCGGAAGAGCACACGTCTGAACCT | 19 | TTTTTTCAATTT |
| 21 | CATGGAGCACTG | 19 | ATTCAATGAATCCATTCGATTCC |
| 21 | TTCCTTTTTC | 19 | TGTTCACCAAG |
| 21 | GGCAAACTTGAGCTC | 19 | TTTTTTTTAACTTTATAA |
| 21 | AGATCGGAAGAGCACACGTCTGCTCCC | 19 | ATTATTTTTTGCGCTCTTTTAAGTCACGG |
| 21 | AGATATCTCAAATTAAATTTC | 19 | GCTACGTTTGTG |
| 21 | ACAGACACACACAC | 19 | TGCGGCAGCAACAAAGCC |
| 21 | TCCTTTTCCTTATCTCTA | 19 | ACGAAAACCAAGTCAGTGTACTCGAT |
| 21 | CTCTAGTTTTCGCAAAAAATG | 19 | GTTTCTCTTCCAATGTTTTGGATTGTTCTAGAA |
| 20 | TCGCAGCC | 19 | TGTCGTTTCGACGGG |
| 20 | TCGGGAC | 19 | TCCAGATCGGAAGAGCACACGTCTGACTC |
| 20 | TAATTGAATGTAA | 19 | ATTATCAC |
| 20 | TGGTGATGGTGGTGT | 19 | AATTAAATTTAGATATCTCA |
| 20 | TTTTAAATAATAAAATTAAGA | 19 | TGCTCCAGTTTTCGCAGTTT |
| 20 | TTTTTATTTTATTA | 19 | GATTCCATTCGATTCCATTTATGAT |
| 20 | TATTAAT | 19 | AACTAAGTTAAATAT |
| 20 | GAATGGAATCGAATGCAATCATC | 19 | AGTTATG |
| 20 | ACTGCTCCAGTTTTCGAAAAA | 19 | ACCCCGCAAGTA |
| 20 | ATCGAATGGAATT | 19 | ATGTTTGT |

| 19 | TTTCTTGCTTTC | 18 | GGCGAA |
| --- | --- | --- | --- |
| 19 | GATTCAA | 18 | CTCCGCCTC |
| 19 | AATTATTAAT | 18 | AGATCGGAAGAGCACACGTCTGAACTCCATTC |
| 19 | AAATTTATAAATT | 18 | TCCTCTCC |
| 19 | TTGGTGGACAGG | 18 | AAATACG |
| 19 | CTGCTCTAGTTTTCGTAAAA | 18 | TTGCCGTTACCTTTTTTTTTCTTCTACTTTCA |
| 19 | AGATCGGAAGAGCACACGTCTGCTC | 18 | AAGATTT |
| 19 | TATAAATATATAAAAT | 18 | TTTTTTAACTCTATATT |
| 19 | GCCTTTAAAA | 18 | GAGTCCATTCATTT |
| 19 | TTCCTTTCTTTC | 18 | GTTTTGGTTAATTTAGTTAGGGTTGG |
| 19 | TTTCTGCTGCT | 18 | AGAAACAATC |
| 18 | AGATCGGAAGAGCACACGTCTGAACTCCAGTCACCC | 17 | TAAAGCTTTTT |
| 18 | TAACCAGCGG | 17 | TAATAATAAAAT |
| 18 | ATTTGCAGAAAATGCAGATTT | 17 | TGCTCTAGTTTTCGCGAAAAC |
| 18 | TTAGTTAATCTTAG | 17 | CCATTCGATTACATT |
| 18 | AAAACAACGAAAAA | 17 | TTTCTCCTTTCTT |
| 18 | TAGAAATAATGTTAGAGT | 17 | ATTCCATTCGATGCCATTCGATG |
| 18 | CTTCTTCT | 17 | TTTATATT |
| 18 | GAAATACTAAAAACTAAAAACT | 17 | GACGACGACGAGTCC |
| 18 | TCACGGATTATTTTTAGCGCTCTTTTTAA | 17 | GATCGGAAGAGCACACGTCTGATCCCCCA |
| 18 | TAAAACTCTCCCATTC | 17 | TTTTTTTAAA |
| 18 | CGACTCGTGTGAATGACTCACA | 17 | CAGATCGGAAGAG |
| 18 | TTTCAAATTTTGAGG | 17 | TTTCTTCAGCTTCGTTTTCA |
| 18 | TGCTCCAATTTTCGAAAAAA | 17 | TCTACTGTAT |
| 18 | TTTTGCCACATAAAAGGAGTAG | 17 | CCATTCAATGATTCCATTCCAGT |
| 18 | GAATGGATTCGAATG | 17 | ATCATCAAATGGAATCGAATGGA |
| 18 | CTTTCTC | 17 | AGAAGAAAGAG |
| 18 | TGCTCTAGTTTTCGGCAGTT | 17 | ACTGTTTTAT |
| 18 | TTTTATGTATTATTA | 17 | TTCATCTCTCTGATTA |
| 18 | TGGTCCG | 17 | AAACTTGACTAGCTAAACTTATAATGT |
| 18 | AGTTAGAAATTAA | 17 | TCAGATCGGAAGAGCACACGTCTGACTCC |
| 18 | TTGCCTTA | 17 | AACTGCTCCAGTTTTCGAA |
| 18 | TCACGGATTAATTTTTGCGCTTTTTTAA | 17 | CAAATTTGATAT |
| 18 | CTGCTTTTTATTC | 17 | TTTTTTTTCATCTTC |
| 18 | GATCGGAAGAGCACACGTCTTCTCCA | 17 | ATTAAATTTAAGATATCTCAA |
| 18 | TTTAATTTCAAA | 17 | TCTAAAGAAGATGCT |
| 18 | ACAAACTTGTTTTTCTG | 17 | CTTATCCTTCAGAA |
| 18 | CTTAATTGAT | 17 | TATTTGTGGAATTGATG |
| 18 | GTGATGGTG | 17 | AGATCGGAAGAGCACACGTCTGAATTCC |
| 18 | AAAAACAATTTTTAG | 17 | AATGGAATCGAATA |
| 18 | TTTATATAATTTTTATT | 17 | TTATTGCTGTCTCCGA |
| 18 | ATCTTCCTAGCTCTTCTAAA | 17 | CTACTCTTTGCTGGCACTGCACCAA |
| 18 | TCCTTGCTTTTTCATCTTGATTTTCCACATA | 17 | CACCCTGACCCAGGAATGGGCTCACCCTGAC |
| 18 | ATATTTGATGTACTTTC | 17 | ATTCAATTCGATTCCATTCGATGTTG |
| 18 | CCTCCCTCCCCC | 17 | CTGACGA |
| 18 | TGCTCTAGTTTTTGGAAAAC | 17 | GGGAAGTAGAGTATTAAT |
| 18 | GGGAAGA | 17 | AATGGAATCAAATGGAATCAATGA |
| 18 | TGTTTTAATTTTTTTTATATT | 17 | TTGTTTTTGTTTATAAA |
| 18 | GAACCGTCACCG | 17 | AAGGGGCTTGC |
| 18 | TGGAGTGGAG | 17 | TTCCAAAATCTTCACTTCTGGAAATCGAGT |
| 18 | TTCTTCCATCCTTCTGGAAGTTTCCTTCGACCT | 17 | CCAGATCGGAAGAGCACACGT |
| 18 | AAAACTTAAATTAAAAACATTA | 17 | CACCGACACGGATGCCGA |
| 18 | ATTTTTTTTTTAAT | 17 | TGCGCTCTTTTTAATCACGGATTTTTT |
| 18 | TTATTCA | 17 | ATGTTGA |
| 18 | AGAAAAGAAGAGAGAA | 17 | ACACACACAGACTCACAC |
| 18 | AGTTTTCGAAAGAATGCTCC | 17 | TCCGCT |
| 18 | GCAGGTGTGA | 17 | TTTTTTCGT |
| 18 | CTGACCTGAGCC | 17 | ATTCAACA |
| 18 | CTTTAGAATTTCAGTTACA | 17 | ATGAATGGATGGATGGGTAA |

| 17 | AATTGCTCCAGTTTTCGCGG | 16 | TGCTCTAGTTTTCAAAAAAA |
| --- | --- | --- | --- |
| 17 | TATGTGTGTGTGATGA | 16 | ATCTGGAAG |
| 17 | CTTCTGC | 16 | AACAACTCAGGGTTCCACACC |
| 17 | ATGGAATCAAATGGAATCATCGA | 16 | AAAATATGCTCTAGTTTTCG |
| 17 | AATTTAGATTTATA | 16 | GGATTATTTTTGCGCTTTTTTAGTCAG |
| 17 | CCAGAG | 16 | GTGATGA |
| 17 | GTTTTCGCTAAAATTGCTCCA | 16 | CTCTTTTTT |
| 17 | ACCGAGT | 16 | TTCTTCTTTT |
| 17 | GAGCATTTTTTCGAAACTA | 16 | TTTTCGCAAAATTTGCTCTAG |
| 17 | TAGCTGCTG | 16 | AGATCGGAAGAGCACACGTCTGTCCCC |
| 17 | CCTCCTCCCCC | 16 | TCACGGAATACTTTTTGCGCTCTTTTTAG |
| 17 | GAGTCCATTCAATGATTCCATTT | 16 | TTTTCCATCTTCTATGG |
| 17 | AAAGAAGAAGGAGGAG | 16 | TTCACACGACTCGTGTGATGTTTCTTGCGTTTT |
| 17 | GGGACCGTGGTCGTCGTGACC | 16 | TCTAGTTTCCGTAAAACTGC |
| 17 | AAAAAAATATAAAGTTA | 16 | ATTTTTCAAATATTTTTTGACCGTT |
| 17 | GAATGGAATCATCAATGGACTC | 16 | AGGTTGGGC |
| 17 | TATTTTTTAATTTTTTA | 16 | CCGATCAAGAGC |
| 17 | ATTGCTCCAGTTTTCGCAAT | 16 | TCTTTTTTGTCGCGGATTATTTTTGCGC |
| 17 | AGATCGGAAGAGCACACGTCTGAACTCCAGTCTCC | 16 | TTCTTCGTTCTTCGTTTTCAT |
| 17 | TTTCACACGACTCGTGTGAATG | 16 | GGGCACTCCAACACCTGTGCCACACCT |
| 17 | ATCCTCAGATCGGAAGAGCACACGTCTGA | 16 | TTCTATTCCA |
| 17 | AACTTTACCTTAAG | 16 | ATTTTTTTTGCTTT |
| 17 | TTTCACGAGCAAC | 16 | GCTCTGTC |
| 17 | GGAATCAACATCGAATGGAAACGAAT | 16 | GACACGACACGTGACAC |
| 16 | TGTCGTGTTCGGCC | 16 | TTTTTGTAATTCA |
| 16 | AAAAACTTTTTTTGG | 16 | TTTTTCTTTTTCTTCTCTTCT |
| 16 | AGATATCTTAAATTTAATTATG | 16 | AAAAAACTATGAAGTTA |
| 16 | TTCTCTCTGTCTG | 16 | TTAAAATCGAAGATT |
| 16 | AAAGTAAATCATCAAATAA | 16 | TCCATTCCACTCCACTCCTT |
| 16 | CCTTCCCCCCCCCC | 16 | TTTTTGATTTTTTATA |
| 16 | TCCATTCGATGATACCATTCAAT | 16 | TGCTCTAGTTTCCGAAAAAAT |
| 16 | AGAAGAGCCTGAGCTTGA | 16 | TTATTATACTTATTTATGTA |
| 16 | CAGGGAGCCG | 16 | AAAAAAAAACCCTAACTAAAAAGAGCGC |
| 16 | TTTTTTGAATTAATTTAACGT | 16 | GATTCCATTTCATTCCATTTGAT |
| 16 | GAATGGAGTGGAGTG | 16 | GACGTCACCT |
| 16 | TTGAATGGAA | 16 | AAAAATTTAAATGAATTTAAATGC |
| 16 | TGATTCCATTCGATTCCATCCGATGA | 16 | TTTTTAATACATTTTA |
| 16 | CCCCCACCC | 16 | CAAGTCAGTGTTTCGACAAGAAAAC |
| 16 | AATTGCTCTAGTTTTCAAAA | 16 | AATTTTTTTGT |
| 16 | TTATTCCGGATTATTTTTTGCGCTCTTA | 16 | TTTTTAAATTG |
| 16 | ATATAATATATCAA | 16 | AATTCATGCAGGAAATCCAAC |
| 16 | CTTCTGCGGCGGGAGCCT | 16 | TTTCTGGTTTTTCAGTTATATGAA |
| 16 | TATATATCTTAATATA | 16 | TCACGGACTATTTTTTGCGCTTTTTTTAG |
| 16 | CACACACAGA | 16 | ATAGATAATTACAGAAGTGAT |
| 16 | AGATCGGAAGAGCACACGTCTGAACTCCCT | 16 | TGCTCTAGTTTTCGGACATTT |
| 16 | GCTTCGCTCTTGC | 16 | CTAAATCG |
| 16 | CATTCGATTCCATCTATGATGATTC | 15 | TCAGCTTCTTTCTTTATTTTTTCC |
| 16 | TCTTCCATCCTTCTGGAATTTTCTTCGACCTTC | 15 | GAAGAAACCGAGAGAGAGAGAGCG |
| 16 | AACAAATC | 15 | GGATCAGGCTCGGGCAGCAAGGGATCT |
| 16 | ATGTATATAATAT | 15 | CATCCAC |
| 16 | AAAAATTAT | 15 | ACAATTTTAAGTAT |
| 16 | TTGCTCTAGTTTCCGAAAAA | 15 | TGCAAATG |
| 16 | TTATTCATCTCTACGA | 15 | TCAATATATTTTTTAATA |
| 16 | TGTTTTTTGA | 15 | TTTTTTAGTCACGGCTATTTTTTGCGCC |
| 16 | TTCCTTTCTGTCGGTCT | 15 | TTTTTTTTTATTATA |
| 16 | TGCTCCAGTTTTTGAAAAAC | 15 | GAATTGTAATTTGTTGTCAGTTAG |
| 16 | ATGATGATTCCTTTCATTTCCATTCG | 15 | TTTTTTTAGTCCTCTA |
| 16 | TTTTTTAATTTT | 15 | TCTCCGATTATTCA |
| 16 | TGTGATGTTTCACACGACTCG | 15 | GCCAGACAAAGAAG |

| 15 | GAAAAATA | 15 | AAGGGGAC |
| --- | --- | --- | --- |
| 15 | TATATATACAG | 15 | ATTCCATTCATTCCATTCGATGATG |
| 15 | CTCTAGTTTTGGCCAAAAATG | 15 | AGTTAGAAAGACAGTGTGAGCC |
| 15 | TCCCCCCTCCC | 15 | GTTCGCA |
| 15 | CTCCCTTC | 15 | GAATGGAATCAAATGGAATCATCATC |
| 15 | AATGTAATGGATTCA | 15 | CTGCTCTAGTTTTCGAACAAA |
| 15 | AATATTTGTTTCTCA | 15 | CCATTGCGCTCAGAGGCTCTCTGCCAAAAA |
| 15 | AAAGAAAGAAGCTGAAGAAAAAGT | 15 | GCTGCCATG |
| 15 | TTCCCAGATCGGAAGAGCACACGTC | 15 | GTTTTTGAAAAATTGCTCTA |
| 15 | AATGGAATGTACTCG | 15 | TCTCTTCCTCTTCC |
| 15 | AAGAAAAAAT | 15 | ATATCTCAGAATTGAATTTCAG |
| 15 | CTTTTGC | 15 | AGATCGGAAGAGCACACGTCTGATCTCTC |
| 15 | CTAGAGCATTCTTTCGAAAA | 15 | CAGCTC |
| 15 | GGCATGGGC | 15 | TCGAATGGAATCATC |
| 15 | GGATTATTTTTGCGCTCTTTTAATCAC | 15 | TTTTTTTGTCTTCA |
| 15 | TTCATCATAATTTTGCT | 15 | ATAGAC |
| 15 | CTACGTGTAAATCACCTCTGTC | 15 | ATTTATTATTTATGTTTTGTTTAACT |
| 15 | TGTTGTCAGTTAGGAATTGTATT | 15 | TATAAACATTTTAATATATTAAA |
| 15 | GGAACTTCTACA | 15 | AATGGAATCAAATC |
| 15 | ATTTCAGATATCTGAAATTA | 15 | TTGCAATTGCTACCTTGCTAATTCG |
| 15 | TAGACAC | 15 | TTCCATCTCTATGGTTT |
| 15 | TAATTAAATTTAAGATATCTC | 15 | TTTATATTTATTTTA |
| 15 | CTTTGAA | 14 | AAAGATAAGTTCTGAAATATAATATCTA |
| 15 | GTACCAG | 14 | TTTATATGTACATTTT |
| 15 | TCTTTTTTAACTAGTTTTTTTACTT | 14 | CATCATCAAATGGAATTCAAAGGAAT |
| 15 | GCCGGACACCAACGCATT | 14 | TGCTCTAGTTTTCATAAAAT |
| 15 | CCTCCTCCCCCC | 14 | TCATCTTCTTCT |
| 15 | ATGATTCCATTCGAATCCATTCCATG | 14 | GTATATAACT |
| 15 | CAAAAATAATCCGTGATTTAAAAAAGCG | 14 | CCACTCTA |
| 15 | ATATCCACAATTGAATTTCAG | 14 | AGATCGGAAGAGCACACGTCTGTCCTCC |
| 15 | AATCTGAATTTG | 14 | ATATACACATATCTAT |
| 15 | AAAATAATCCGTGATTA | 14 | AGCAGTTTTGGCGAAAACTAG |
| 15 | TTTTTTCGAAAACTGGAGCAA | 14 | TGGGGACATCACCGC |
| 15 | CCAACCTGAGCGGCG | 14 | ACCTCAGGGGCGCGA |
| 15 | TCAGATCGGAAGAGCACACGTCTGAA | 14 | TCTTTCTTTCTTTATGTCCG |
| 15 | ATAAGAGGTTGGGGTGCGGAA | 14 | TTTCAGAGTTTCAGCA |
| 15 | TGTTTTCAATCGTATTT | 14 | AAAGTAAGCAAAACAAGAAATA |
| 15 | AAACCGCTCAA | 14 | GAAGACG |
| 15 | GAATCGAATGGAAT | 14 | TTAACTAGAACCTACTC |
| 15 | TTTTTTATTCAATTG | 14 | ATTAATTTTAAAATTAATA |
| 15 | TTATTTTTTGCGCTTTTTTTAATCACGA | 14 | TTTTAATCACGGATATTTTTTGCGCTC |
| 15 | AGAATCCTGAATCGTTTAAGTT | 14 | AGATCGGAAGAGCACACGTCTGAACTCCATT |
| 15 | TTCCATTCCATTCCATTCCT | 14 | TATTTTTTTTTCTTTTTCATAGACTACTATA |
| 15 | TCCTGTTGTCC | 14 | CGAAAGGAATCATCATCGAATGCAAT |
| 15 | CCAGATCGGAAGAGCACACGTCTGAACTCCAGC | 14 | ATTTTATTATGTTCAA |
| 15 | TAATTTGTCCTTAAAAATATCATA | 14 | TGTTCCTTTGCTTTAT |
| 15 | AATGGAATCATCATGAATGGAATCG | 14 | ATTATTTTTTGCGCTCTTTTTAATTACGG |
| 15 | ATTATTTTTTGCGCTCTTTTTTATCACGG | 14 | TTTTTTGCGCTCTTTTTAATCACGGCTT |
| 15 | CAGCTT | 14 | GCGCAAAAAATAATCCATGACTAAAAAGA |
| 15 | ATGATTCCATTTGATTCCATCTTG | 14 | CTCAG |
| 15 | TCCGACGCTCTCAC | 14 | TATATATTTTAAAAATATTT |
| 15 | AAAACTGCTCCAGTTTTCGT | 14 | TTTTATTTCGTTTTAA |
| 15 | AAATGGAATCATCTAATGGACTC | 14 | AGAAGCAGA |
| 15 | TTTTTAGAACTTTC | 14 | TGCTCCAGTTTTTGAAGAAA |
| 15 | TTTTCTGTA | 14 | ACCAAGTCC |
| 15 | GTCTCTCTCTGTCTGTCTCT | 14 | AGTTATGTAGTAACTT |
| 15 | AGATCGGAAGAGCACACGTCTGTTCC | 14 | CGATTGC |
| 15 | CATCATCAC | 14 | AGACAGCCAAGGTTGC |
| 15 | CTTCTTGCAAC | 14 | TAAATTTATAAAAAAGTGC |

| 14 | ATCATCGAATGGAAATGAATGGAATC | 14 | AAAATTTCGTTACA |
| --- | --- | --- | --- |
| 14 | TTTTTAATATTTTTTAA | 14 | AGATCGGAAGAGCACACGTCTGAACTCCAGTCACT |
| 14 | GGCAGGAGGAAGAGGAAGA | 14 | ACGACAGAGTCAGAGCCA |
| 14 | GCCCGTGCAGTCCCTGCA | 14 | TATTTTTTGCGCTCTTTTTAGTCACGGAC |
| 14 | ATCGAGAAAGAAA | 14 | TCTGTCCCTCTCTCTCTCTG |
| 14 | GTTCACCACTGTGCTTTAAAAAAAC | 14 | CAGCATTAG |
| 14 | TAGAGCACTCTTTCGAAAAC | 14 | GACACTGACTTGGTTTTCGTGTCGA |
| 14 | TTTTAATCACGGCTTTTTTTGCGCTTT | 14 | CATCCGAGCACTCGCAGC |
| 14 | TGCTCCAGTTTTCGGAAAAA | 14 | ACGAAGAAAGAAGAAATGAAA |
| 14 | TTTGTATCTTCTAAA | 14 | CTCCTG |
| 14 | TGCCTTCGAGCAGG | 14 | TCTTTCCTTCTT |
| 14 | GACTTGGTTTTCGTTGTCGAGTACAC | 14 | TTTTTTAATA |
| 14 | GTTTTCGTCAGTTTTGCTCTA | 14 | TTTTCTTCCGTTACGTTC |
| 14 | CAGTATTT | 14 | TGCTTACCTGGGCCCATCCTGAAGGAAAGGTCCA |
| 14 | GTTCGTATACAGCAACTATATGC | 14 | TTCGATTCCATTAGATGATGATTCCA |
| 14 | CAAAAACG | 14 | ATATCCATAATTAAATTTAAG |
| 14 | TTTCTTCGACCTTCTCTTCCATCCTTCTGGAA | 14 | GAAGTGCACAAAATAAAATG |
| 14 | ATTCTTGATTCTTGATTCTGG | 14 | ACTGCTCCAGTTTCCGCAAAA |
| 14 | CATTTCT | 14 | AGCGCAAAAAATAAGCCGTGACTAAAAAA |
| 14 | CCCCCCTCTCCCC | 14 | TGATCAAGTCCATAGCTGACC |
| 14 | ACACAGCCCTGAGTCCTCCCT | 14 | TCTTCCTCC |
| 14 | TAGTCGA | 14 | GGCGTTTTGATAGGCGAC |
| 14 | ACCTTAGT | 14 | ATTCTCG |
| 14 | TCACTCGATAGATCTCTAATAC | 14 | AAAAAATTATTTTTACA |
| 14 | AGATCGGAAGAGCACACGTCTGAACTCCAGTCACTC | 14 | CGAAAAGAATCGAAT |
| 14 | TTTTTTGCGCTCTTTTTGATCACGGCTA | 14 | GGCGCGGGTCTTCGTTC |
| 14 | ATTCCATTCCATTCCATTCA | 14 | GGAATGGAGAGCAATGGAATGGAAT |
| 14 | AATCG | 14 | TTTCGAAGTCAAGTACACTGACTTGGT |
| 14 | ATCTCAT | 14 | GCACACGTCTGACCTAGATCGGAAGA |
| 14 | GTCACGGCTATTTTTTGCGCTTTTTTTA | 14 | GTTTTCGCTAAATTGCTCCA |
| 14 | CATCAGTACAGCCGCTC | 14 | AAAAACAA |
| 14 | GAGCAGGA | 14 | AGATCGGAAGAGCACACGTCTGATCCCT |
| 14 | CCCGATTGTCGAATCATC | 14 | TTTTTTGCGCTCTTTTTGATCACAGATT |
| 14 | GCGTGCA | 14 | GACTTGGTTTTCGTTGTCGAAGCAC |
| 14 | TACTTATCTC | 14 | CTGGATG |
| 14 | CTTTGACTAT | 14 | TTCATTTATTTTTGGT |
| 14 | AAAAGCT | 14 | GTAGGCCACGACTGTGTCTAAATGTC |
| 14 | ACGGAATATTTTTTGCGCTCTTTTTAGCC | 14 | ATAATTC |
| 14 | CCCTGCACTG | 14 | TTTCCTCCTTCCCTTTTC |
| 14 | AGCACACGTCTGAACTCCTCCCAGATCGGAAG | 13 | ATTTTTATTTATTATA |
| 14 | GATGAGCCCCAAGCTACCACGCTC | 13 | GGTGGCAGGCCGCTTCCAAGAT |
| 14 | GTGGGGGTAGGGTTTAGGTTAGGTG | 13 | AATGCAATAT |
| 14 | CCTGAG | 13 | TTCTTCTCTTCTTCCCTT |
| 14 | AATTGCTCTAGTTTTCGAAC | 13 | GAGATGTTGCTAATACTGAAA |
| 14 | AAGAAGATAGAAAA | 13 | CCCCCCCCCTCCT |
| 14 | CCATTCCACTCCAGT | 13 | TCTGTCTCTTCTCTCTGTCTG |
| 14 | ATTGCCTTTACCTTTTCTTTTCTTCTAGCTGC | 13 | AATTGTTTATAAAT |
| 14 | GGGTTGACG | 13 | TGGATGAATGGGAAGATGGA |
| 14 | ATCATCAATGGAATCGAATGGA | 13 | TTTGG |
| 14 | TTTCCAG | 13 | GGTTCGCCCGATGGCGGTGTT |
| 14 | TGCACACACAGGGCACAGGTACACGCAGGCACACG | 13 | TTTTGCTCCAGTTTTCGAAA |
| 14 | CTCGGCAGTGTTCGA | 13 | AGTAAC |
| 14 | ATTTCAGATATCTAAAATTA | 13 | AAATTTACCAATTCATCAA |
| 14 | GTTTTCGGCAGTTTTGCTTCA | 13 | CGGTGACAG |
| 14 | AAAACAATTTT | 13 | GTTAGAGGAAGATTTCG |
| 14 | ACCAGTTATACCATCAGTGACATAGTCT | 13 | GTACTCGACGACGAAAACCAAGTCAGT |
| 14 | TTTTTTTCTTCTACTTGCATTGCCTTTACCT | 13 | ACCCCC |
| 14 | CTTTCTTCCTGTCGGTCTTTC | 13 | CTATCTATCAT |
| 14 | ATTCAGG | 13 | CAGATCGGAAGAGCACACGTCTGAACTT |

| 13 | CTATTATTTTTTTTT |
| --- | --- |
| 13 | AGATCGGAAGAGCACACGTCTGAACTCCTCT |
| 13 | GTGTCAG |
| 13 | GAAAATGCTCTAGTTTTTCGC |
| 13 | ATTACACAAGTCGTGTGAGAC |
| 13 | AAATAAATAAAAATT |
| 13 | TATTTTTTGCGCTCTTTTTTATTAAGGAA |
| 13 | TCCATTAGATGATTCCATTAGAT |
| 13 | TGAAAGTATATATC |
| 13 | TATTTTTTGCGCTCTTTTAATCACGGC |

| **Bembidion clemens (#SRR5230406)** | | | |
| --- | --- | --- | --- |
| 309379 | AATA | 188 | CTAAACC |
| 39595 | CCCGGCCCTAATACCTGCCCT | 179 | GGTAGGTCAGGTTAGGGCAGGCGG |
| 23079 | AGGGCAGGG | 177 | TATATTTATT |
| 18125 | GGCCCTAACACCTGCCCTCCC | 176 | GGCCGGGAGGGCAGGTATTG |
| 13809 | CAAA | 174 | CCCTCCCGGCCCCAACACCTA |
| 12938 | CCGGGAGGGTAGGTGTTAGGG | 173 | TAACACCTACCCCCCCGGCCC |
| 4520 | AAAAATT | 173 | TACCTGCCCTTCCGGCCCTAA |
| 4424 | TAGGTATTAGGGCCGGGAGGG | 164 | CCCGGCCCTAACACCTCCCCT |
| 3495 | CCCGCCCCC | 162 | TTAGGGCCGGGAGGGCAGGG |
| 3450 | AAT | 161 | AAGAAGGAAGCAGAAGAAAAAGCA |
| 3322 | AATAATA | 160 | GGGCAGGTATTAGGGCCGGA |
| 3289 | **AACCT** | 160 | CCTGCCCTCCCGGCCCTTATA |
| 3016 | AACAAAT | 153 | CCCTCCCGGCCTAATACCTG |
| 2166 | CCTGACCTACCCCCGCCTGCCCTAA | 153 | TTTTTCTTCAGCTTCCTTCTTTGC |
| 1715 | AGATCGGAAGAGCACACGTCTGAACTCC | 153 | CCCACCCCC |
| 1544 | CCCTCCCGGCCCTAAACCTG | 152 | ACCTGCCCTCCCAGCCCTAAT |
| 1402 | CAA | 151 | ACCCTCCCGGCCCTAACACCC |
| 1393 | AGGGCCGGG | 151 | GCCCCAACACCCACCCCCCCC |
| 1161 | TAACCTAAACC | 150 | AGGTGTTAGGGCCGGGAGGGA |
| 936 | TTATATTT | 150 | CCTGCCCCCCCGGCCCTAATA |
| 914 | TGGAA | 149 | AAGT |
| 751 | AGATCGGAAGAGCACACGTCTGAACTCCC | 148 | CCGGGAGGGCAGGTGTTAGAG |
| 694 | AAATAAAATTA | 147 | GCCCTAACACCTACCCCCCCC |
| 691 | AAATAAAG | 135 | ACCCCCCCCCCC |
| 680 | CTCCCGGCCCTAACACCAACC | 134 | AGCTGTCCTTA |
| 633 | TTGAATT | 131 | CTTCTTTTCGT |
| 608 | AGGGCCGGGAGGGCAGGG | 129 | GTTTGGTTTAG |
| 599 | AAAAAGAAGACG | 125 | CCCCGCCCCAACACCCCCCCC |
| 594 | TTGGGGGCCTTAGGGCGGGCA | 125 | TAGA |
| 559 | AGGGCGGGG | 125 | TACA |
| 514 | AGGTTAGGTTA | 125 | ATACC |
| 477 | GTATA | 125 | GTATATA |
| 474 | TTATT | 124 | CCTGCCCTCCCGGCCCTCATA |
| 455 | TCCCGGCCCCAATACCTGCCC | 124 | GGGAGGGCAGGTAGTAGGGCC |
| 431 | AGGGCCCGGAGGGCAGGTATT | 121 | TTTC |
| 426 | TCT | 114 | AAGAAGCTGAAGTAAAAGCAAAGA |
| 376 | CGCT | 114 | CCCCCCCGCC |
| 357 | CCCTCCCAG | 111 | AGATCGGAAGAGCACACGTCTGAACTCCAGTCC |
| 311 | GAAGACGAAAAC | 110 | GTTAGGGCCGGGAGGGCAG |
| 300 | CTCCCCGCCCTAACACCTACC | 110 | AGATCGGAAGAGCACACGTCTGAACTCCAGTC |
| 289 | CCTGCCCTCCCGGCCCTAAAA | 110 | AGAAGAAAGAAGACA |
| 285 | TTAATCTTTAATC | 110 | CCTCCCGGCCCTAATACCTCC |
| 284 | TATAATAAAAATATATA | 108 | CCCCTCCCC |
| 280 | AGGGAGGGCAGGTATTAGGGC | 107 | CCCCGCCCCCACACCCCCCC |
| 274 | CCTACCCTAACCTGA | 103 | TCGGAAGAGCACACGTCTGAACTCCTCAGA |
| 273 | AGGGCCGGGAGGGCTGGTATT | 99 | CCCCGCCC |
| 268 | CCTGCCCTCCCCGCCCTAATA | 98 | TAG |
| 254 | GGAGGGCAGGTATTAGGGCCGGGAGGGCAG | 98 | AGCTGTTTTAAA |
| 253 | CAT | 98 | TTGAAA |
| 235 | GGTGTAAATGGGTTA | 97 | GCCCTCCCGGCCCTAATACCC |
| 213 | CCCTCCCAGCCCTAACACCTA | 97 | CCCTCCCGGCCCTAATACCTT |
| 213 | CCCTAAAACCTACCCTCCCGG | 96 | AGGTATTAGGGCCAGGAGGGC |
| 211 | TATTTT | 93 | TTTTTCGTCTTCT |
| 211 | AAGCTGTTTT | 91 | CCCCCACCCCCCCC |
| 207 | ACCTGCCCTCCCGGCCCTAT | 91 | CGGGAGGGAGGTGTTAGGGC |
| 207 | CCGGGAGGGCTGGTGTTAGGG | 91 | GAGGGCAGA |
| 204 | ACCTGCCCTCCCGGCCCTAC | 90 | TGTG |
| 199 | GGGATTAGGGCCGGGAGGGCA | 90 | AGATCGGAAGAGCACACGTCTGAACTCCT |

| 88 | TACACCTAACACATT | 56 | TTTAAG |
| --- | --- | --- | --- |
| 88 | CCTGCCCTCCCGGCCCTAAT | 56 | CCTGCCCTCCCGGCCTTAATA |
| 88 | CCTGCCCTCCCGGGCCTAACA | 56 | ACAACAA |
| 87 | ACCCCCCCCCCCC | 55 | TATTTTT |
| 84 | AATCTTT | 55 | ATTTCTATTTATTT |
| 82 | TTGGTCAGGTTAGGGCAGGCGGGGG | 55 | TCCAGATCGGAAGAGCACACGTCTGAACTCCA |
| 82 | TTAGGGCCGGGAGGGCTGGGAGGGCAGGTA | 55 | CCCCGCCCCAACCCCCCCCC |
| 81 | AGCACAAAATT | 55 | CCCTACCCTCCCGGCCCTAA |
| 81 | CCTTTTTCGTCTT | 54 | CCCTCCCGGCCCTAATACCAA |
| 79 | TAAATAAAA | 53 | ATGTTTTC |
| 78 | ACACTAT | 52 | AGGTTAGGACAGGCGGGGGTAGGTC |
| 78 | AGATCGGAAGAGCACACGTCTGAACTC | 52 | TATGTTTTAAATT |
| 77 | AAGCTGTTTAA | 52 | GCTAAACAGACA |
| 76 | TTAATTGAAT | 51 | TAAT |
| 76 | CCTACCCCCGCCTGCTCTAACCTGA | 51 | CCCCCCCCCCGCCCCCAAACC |
| 75 | TTTCGTCTTCTG | 51 | CCTCCCGGCCCTAATACCTGA |
| 75 | AGACAGCTTAA | 50 | GGGAGGGCAGGTATTAGGACC |
| 75 | TCAT | 50 | CTAACACCTACCCTTCCGGCC |
| 75 | CCTGCCCCC | 50 | CCTACCCCTAACCAAA |
| 74 | ATTTA | 50 | CCCCCCCCCA |
| 71 | TTTTAAGTTCGGGTAG | 50 | CCTGCCCTCCCCGCCCCAATA |
| 71 | CCTCCCGGCCCTAATTCCTGC | 49 | ATTTACA |
| 71 | CTGTCGTGT | 49 | ATTCCATTCGATTCCATTCGATG |
| 71 | CCCTCCCCGCCCTAACACCTG | 49 | TATGTAT |
| 69 | ACATTT | 49 | CCTCCCCCCCCCCC |
| 68 | CAACACATTTACACC | 48 | AAAAAAAT |
| 67 | ATTAAG | 48 | ACCCACCCCCCCCGCCCCCAA |
| 67 | AGGGCAGGGAGGGCAGGTGTTAGGGCCGGG | 48 | TCGGAAGAGCACACGTCTGAACTCCATCCCAGA |
| 66 | CCCTGCCCTCCCGGCCCTAAC | 48 | CCCCCCCCAAG |
| 66 | ATTAAATTTATTATATTAT | 47 | ATATACA |
| 64 | CCCCCCCCGCCCCAACACCC | 47 | TTGGGGCGGGCA |
| 64 | AACACCTGCCCTCCCGGCCCC | 47 | TTAAATAATTATAATTAT |
| 63 | TGGTGCACCAGTCTC | 47 | CTCCCGGACCTAACACCTACC |
| 62 | GGGAGGGTAGGTGTTAGGCCC | 47 | CCTGCCCTCCCGGCCCTAACT |
| 62 | TCCCCGCCCTAATACCTACCC | 47 | AGGGCAGGGAGGGCAGGTGTT |
| 62 | CGGGAGGGCAAGTATTAGGGC | 46 | GCTTTTTCTTCTGCTTTCTTTTTA |
| 62 | TCTTTTTTGCCTTTTCTTCAGTT | 45 | ACCA |
| 61 | AAACAAATCA | 45 | CCCTCCCGGCCCAAATACCTG |
| 61 | GTAGGTTTGGTGAGGG | 45 | CCAATACCGGCC |
| 61 | ACACCCACCCCCCCCGCCCCC | 45 | TGG |
| 61 | TTTTTAATTT | 45 | AGATCGGAAGAGCACACGTCTGACTCC |
| 60 | TTTTCGTCTTCC | 44 | CCCGGCCCCAACACCTACCCC |
| 60 | CCCCCCCCCAAG | 44 | CCTAACCTGACCTACCCCCGCCTG |
| 60 | CCTGCCCTCCCGGCCCTCACA | 44 | GCCCCAACACCTACCCCCCCC |
| 60 | TAATA | 44 | CCCACCCCCCCCGCCCCAAAA |
| 60 | CCCCCCCA | 44 | ACCTACCCTCCCGGCCCCAAT |
| 59 | TTTTG | 43 | TCCCAGCCCTAACACCTGCCC |
| 59 | TGC | 43 | AGATCGGAAGAGCACACGTCTGAACTCCAC |
| 59 | ACCCCCCCCCC | 43 | AGGTGTTAGGGCCAGGAGGGC |
| 58 | GTATTAGGCCCGGGAGGGCAG | 43 | CCCGGCCCCAACACCCACCCC |
| 58 | CCCCCCCCGCCCCCACACCCC | 42 | CCCTCCCCCCCCC |
| 57 | TCTTCTTTCCTCG | 42 | CCGGGAGGGCGGGTGTTAGGG |
| 57 | AGATCGGAAGAGCACACGTCTGAACTCCTCC | 42 | CGGCCCTAATACCTACCCCCC |
| 56 | AATAAATAATA | 42 | CCCCCCCCCGCCCCCAAACC |
| 56 | TTTTCGTCTTAG | 41 | AGATCGGAAGAGCACACGTCTGATCCC |
| 56 | CATGGAGGTGGCGGTGGAGGT | 41 | CCCCCCAACCCC |
| 56 | CCCCCCCCCCCACCC | 41 | CAGCTTAAA |
| 56 | CCCCCCAACCCCC | 40 | GTC |
| 56 | CTTAAAACTAAACGGAT | 40 | AGATCGGAAGAGCACACGTCTGATCTCC |

| 39 | ATTCTAC | 29 | ATACCTGCCCTCCCGGCCC |
| --- | --- | --- | --- |
| 38 | ATACTATACC | 29 | ACCTGCCCCCCCGGCCCCAAT |
| 38 | AGATCGGAAGAGCACACGTCTGACTCCC | 29 | AACTTATAACTACCCG |
| 38 | CCTGCCCCCCCGGCCCTAACA | 29 | AAGAAAAAGCAAAGAAGAAAGCAG |
| 38 | CAAAAAAAAA | 28 | CCCCCCCT |
| 38 | AAGCTGTTTATATCTGTTTT | 28 | AGGA |
| 38 | ATCA | 28 | GGTTAGGGCAGGCGGGGGTAGGCCA |
| 38 | AGTT | 28 | CCCAACCCCCCCCCCC |
| 38 | CCCCGCCCCCAACCCCCCCC | 28 | CTAATACCTCCCTCCCGGCC |
| 38 | AGGGCCGGGAGGGCAGGCATT | 27 | TTTGTT |
| 38 | GGGGCCGGTATC | 27 | GAATA |
| 38 | GAACTCCACCAGATCGGAAGAGCACACGTCT | 27 | CGGGAGGGTAGGTGTTAGAGC |
| 37 | CATTTACACCTAACG | 27 | ACCTACCCCCCCCTGCCCTAACCTG |
| 37 | ACCCCCCCCCA | 27 | TTTTCTTCAGCTTCTTTCTTTGCT |
| 37 | CCCACCCCCCCCGCC | 27 | ACGAA |
| 37 | AGATCGGAAGAGCACACGTCTGAACTCCAGTCCC | 27 | TAGGGCCGGGAGGGCAGG |
| 36 | CCCTCCCCGCCCCAACACCTA | 27 | AGGTGTTAGGGCCGGAAGGGC |
| 36 | GATTCCATTC | 27 | CCCGGCCCTAACACCCACCCC |
| 36 | CCCTCCCGGCCCCAACACCCA | 27 | TTTTATTTATT |
| 36 | GAAAAAAAAA | 27 | ACCCTCCCGGCCCTAATACCC |
| 36 | CCTCCCCCCC | 27 | GGCCGGGAGGGCAGGGAGGCAGGTATTAG |
| 35 | TTCTTTC | 27 | TTCGACATTTGGGAA |
| 35 | TAGGGCCGGGAGGGTAGGTG | 27 | GCTTCCTTTTTAGCTTTTTCTTCT |
| 35 | TTTTTTG | 27 | CACAATTCGTCTCC |
| 35 | CGGGGGTAGGCCAGGTTAGAGCAGG | 27 | CCCAACCCCCCCCCC |
| 34 | AGTGG | 27 | CCCCCCCCAAA |
| 34 | AGAAGACGAAAAAGAAGACGAAAAC | 26 | GATTTTTAACGATGCGAC |
| 33 | ACATA | 26 | TGTCAAACC |
| 33 | TGGTTCTTTAGT | 26 | CCCCCCCCAAAG |
| 33 | CCCGCCTACCCTAACCTGACCTACC | 26 | ACCCCCCCCCCCCCCCCCCAA |
| 33 | TATTTATATATATT | 26 | TTAGGACAGGTGGGGGTAGGTCAGG |
| 32 | AAAAAAAATAAAT | 25 | CCCTCCCGACCCTAACACCTA |
| 32 | TATTTAG | 25 | CCGGGAGGGTAGGTGGTAGGG |
| 32 | CCTGCCCCCCCGGCCCTAAAA | 25 | AACCTAACCCTAACCA |
| 32 | ACCTACCCTCCCGGCCCTAA | 25 | ACACAC |
| 32 | ATGCGACACTTTAAC | 25 | ATATAA |
| 32 | AAAAAAAAT | 25 | ATAAATTAAACTTTATAT |
| 32 | CCCTCCCCACCCCC | 25 | AGGTGATAGGGCCGGGAGGGC |
| 31 | AATTCTGTACAG | 25 | CCCTACCCTCCCGGCCCTAAC |
| 31 | ATCATCGAATGGAATCGAATGGAATC | 25 | TATATAAAATTTAATTTA |
| 31 | CCCAGATCGGAAGAGCACACGTCTGA | 25 | ACCTACCCTCCCGGCCTAAT |
| 31 | GGTGGTGGA | 25 | CCCTCCCCGCCCTAACACCCA |
| 31 | CCCAGATCGGAAGAGCACACGTCTGAAC | 24 | TAACCTGACCTACCCCCGCCCGCCC |
| 31 | AACACTA | 24 | CCTACCCTCCCGGCCCTAATACCTGCCCTC |
| 31 | TTCTTTGCCTTTTCTTCTGCTATCT | 24 | ATTCCATTCCATTCG |
| 31 | GCCGGGAGGGCAGGTGAGG | 24 | TTTTTTTG |
| 31 | GAGGGCAGGCGTTAGGGCCGG | 24 | CCCCCCCCGCCCCAAACCCC |
| 30 | AATATAAAAAT | 24 | TGCTGGA |
| 30 | AATTCACAATTTCAATTTC | 24 | ACCCCCCCCCAA |
| 30 | GGGGCGGGAGTT | 24 | GGGAGGGTTGGTGTTAGGGCCGGATGGGCA |
| 30 | CAGATCGGAAGAGCACACGTCTC | 24 | TTTTCTTCAGCTTCTTTTTTAGCA |
| 30 | TGCCCTCCCGGCCCCAAAACC | 24 | AAAATTT |
| 30 | ATTTCAA | 24 | ATCAAC |
| 30 | CTCCCGGCCCTAAAACCAGCC | 24 | TATATTATTTGT |
| 30 | CGTCTTCTTTTC | 24 | GACAGCTAAAA |
| 30 | CTCCAGATCGGAAGAGCACACGTCTGAACT | 24 | CCCCCCCCCCCCAACCCCCC |
| 29 | AATAATAT | 24 | CCCGGCCCTAACACCTACACT |
| 29 | CTTTA | 24 | AGATCGGAAGAGCACACGTCTGAACTCTC |
| 29 | GCCCCAACCCCCACCCCCCCC | 24 | CCCCCCCCGCCCCCAACACCC |

| 23 | CCTGGTCGAGTCTA | 19 | AGGTCAGGTTAGGGAGGCGGGGGT |
| --- | --- | --- | --- |
| 23 | TTAGAGCCGGGAGGGCAGGTA | 19 | AAAATAAAAAA |
| 23 | CCCGGCCCC | 19 | CCTCGCCGCCC |
| 23 | GGA | 19 | AAATAAAT |
| 23 | GGGAGGGCAGAGAGGGCA | 19 | GGCCGGGAGGGCAGGTTAAG |
| 23 | CCGGGAGGGCAGTGTTAGGG | 19 | TCAGATCGGAAGAGCACACGTC |
| 23 | GGGAGGGCAGGTATTACGGCC | 19 | GTATTAGGCCCGGGAGGGTAG |
| 23 | CCCTCCCGGCCCTAACACCTGCCCTCCCAG | 19 | CCCCCCCCCGCCCCCCCC |
| 23 | AAAGAAAGCAGAAGAAAAGCAAAG | 19 | GCCCTAATACCTGCCCTCACTGCCCTCCCG |
| 23 | CACGCGACACTTTAA | 18 | AGGTGTTAGGGCAGGGAGGGT |
| 23 | AAAGAAGCAGAAGAAAAGCAAAG | 18 | CCTGCCCTCCCGGCCCTT |
| 23 | CCCAACCCCCCCCC | 18 | CCCCCCCCGCCCCCACCCC |
| 23 | TTAGGGCCGGGAGGGTAAGTG | 18 | CCCCCCCCCCT |
| 23 | GCCCTAACACCCACCCCCCCC | 18 | CCAACCCCCC |
| 22 | TTAATA | 18 | TGTAAAA |
| 22 | GGAGGGCAGGTGTTGGGCCG | 18 | AGATCGGAAGAGCACACGTCTGAACTCCATC |
| 22 | GGCGGCGGTAGGTCAGGTTAGGGCA | 18 | AGGTTAGGGCAGGCGGGAATAGGTC |
| 22 | GGGGCGGGGGTT | 18 | CATGACCACCACCGCCACCACCTC |
| 22 | TATTAGGGCCGGGAGAGCAGG | 18 | AGATCGGAAGAGCACACGTCTGAACTCCAGTCACTCC |
| 22 | CCCCCCCCCCCCCCCAACCCC | 18 | CCCCCCA |
| 22 | CCCCCCCCCCCCCGC | 18 | CCCCCCCCCCCCCGCCCCCA |
| 22 | AAAAAAGAAAGCAGAAGAAAAAGC | 17 | CCCCGCCCCACACCCCCCCC |
| 22 | AGATCGGAAGAGCACACGTCTGAACTCCAGTCACC | 17 | ATATGTATTTAATAAT |
| 22 | CCCCCCCCACCCCCCC | 17 | ACACCTACCCTCCCGGCCCTC |
| 22 | CCCCCCCCGCCCCAAAACCCC | 17 | ACCCACCCCCCCCCCCCCCAA |
| 22 | ACCTACCCTCCCGGCCCAAT | 17 | AGATCGGAAGAGCACACGTCTGAACTCCAGTCAC |
| 22 | TTTTTTTC | 17 | CCCAGATCGGAAGAGCACACGTCT |
| 22 | AACACACGACAGATT | 16 | AAAAAACAAAA |
| 22 | TAACCC | 16 | TACCCCCCCCCGCCCTAACCTGACC |
| 21 | CAGGTATTAGGGCCGAGAGGG | 16 | AGATCGGAAGAGCACACGTCTGAACCC |
| 21 | ATTCAGT | 16 | CCCGGCCCTAACACCTACCCA |
| 21 | CCCCCCCCGCCCCAAAACCC | 16 | CCCCCCTCCCCC |
| 21 | GCCCTAATACCCGCCCTCCCC | 16 | CATAAAAAAATACATTAA |
| 21 | CTGAAGAAAAATGCTAAAAAAGAAA | 16 | TCCCGGCCCTAACACCTACCA |
| 21 | TTAATAATCAAAATT | 16 | CCCCCACCCCCACCC |
| 21 | GGGCCGGGAGGGCAGGTTGA | 16 | CACTAAACACTATGCACTGAA |
| 21 | CCCCCCCCCCACCCCCCCCCC | 16 | CCCCCCCCGCC |
| 21 | CAACA | 16 | GTCTCTTACACAATTC |
| 21 | CCCCCCCCCCCCCCCCAACA | 16 | CCCCCCCCCCCG |
| 21 | ATATATATAAAT | 16 | TCCACCGCCTCCTCCATG |
| 21 | CCCTCCCGGCCCCAAAACCTA | 16 | ACCCCCCCCCCCCCAA |
| 21 | CCTAATACCTGCCCTCCCGGA | 16 | AGATCGGAAGAGCACACGTCTGATCTCCC |
| 20 | CCCTCCCCGCCCCAACACCCA | 16 | AGGGCCGGGAAGGCAGGTATT |
| 20 | TCCCGGCCCTAAAACCAACCC | 16 | AGATCGGAAGAGCACACGTCTGAACTCCCCC |
| 20 | CCCTAACCTGACCTACCCCCCCCG | 16 | AGATCGGAAGAGCACACGTCTGAACTTCC |
| 20 | TAAACAGCTTTTAAAACAGCT | 16 | GGGCCGGGAAGGCAGGTGTTA |
| 20 | CCCCCCCCCGCCCCCAACCCC | 16 | CCTCCCGGCCCTCCCTGT |
| 20 | CCCTCCCCCCCCCCC | 16 | TTGGGGGCTTAGGGCGGGCA |
| 20 | TTGGGGGCTTTTGGGCGGGCA | 16 | TCTTCTTCTCT |
| 20 | GCTCCAGATCGGAAGAGCACACGTCT | 16 | GGGAGGGCAGGTATTAGGGC |
| 20 | AAAAAAACA | 15 | AGATCGGAAGAGCACACGTCTGC |
| 20 | CCCCCCCCGCCCC | 15 | AGGGTTGGTGTTAGGGCCGGGAGGGCAGGG |
| 20 | AGATCGGAAGAGCACACGTCTGAACTCCAGTCT | 15 | ATCTCAGATCGGAAGAGCACACGTCTGAACTCC |
| 20 | GGCCCTAATACCTGCCCTCCTGCCCTCCC | 15 | TTTTCTTCAGCTTCTTTCTTTGCC |
| 20 | AGATCGGAAGAGCACACGTCTGATCCCC | 15 | AGATCGGAAGAGCACACGTCTGCCC |
| 20 | CCGGGAGGTGTTAGGG | 15 | CCTGCCCCCCCCGCCCCAAAA |
| 19 | TCGTCTTCTCTTT | 15 | CAGCAACAA |
| 19 | GTTGGGTGTAAATGC | 15 | CCTGCCCTCCAGGCCCTAATA |
| 19 | CCCTCCCGGCACTAACACCTA | 15 | CCCGGCCCTAATACCTGCCCA |

| 15 | CCCCGCCCCCCCCCCCC | 13 | CCCCCCCGGC |
| --- | --- | --- | --- |
| 15 | AGATCGGAAGAGCACACGTCTGAACTCCAGTCCT | 13 | AAAAAAAAAAAT |
| 15 | CCCGGCCCCAACACCCCCCCC | 13 | CCTAATACCTGCCCTCCCGAC |
| 15 | CCCTCCCGGCCTTAACACCTA | 13 | GCCCTCCCGGCCCCAATACCA |
| 15 | TAAAAAAAAA | 13 | CCCTCCCGGCCCTTACACCTA |
| 15 | TATTAGGGCCGGGAGGGCAG | 13 | CCCTCCTGGCCCTAACACCTA |
| 15 | CCCGGCCCTAACACCTCCCCC | 13 | GCCCTAATACCTGCCCCCCCC |
| 15 | CCTTCCCGGCCCTAACACCTA | 13 | GGGGTAGCTCAGGTTAGGGCAGGC |
| 14 | AGATCGGAAGAGCACACGTCTGTCCC | 13 | ACCCTCCCGGCCCAAACACCT |
| 14 | CCTCCCGGCCCTAACACCTAA | 13 | CCCCCCCCCCCCCCCCA |
| 14 | AGATCGGAAGAGCACACGTCTGAACTCCAGTCACCC | 13 | TAATCGTCGAAAAATCTACGT |
| 14 | TAACTATTATATT | 13 | AACACCTACCCTCCCGCCCT |
| 14 | CCCCAATGCCGCCCTAAGGC | 13 | CCCCCCCCCCCCCG |
| 14 | CCTGCCCTCCCCGCCCTAAAA | 13 | AAATTAATTG |
| 14 | CCCTCCCGGCCCCAATACCCG | 13 | CAATTCGTCTTCCA |
| 14 | CCCCCCCCCCCCCCGCCCCAA | 13 | GGCGGTGGAGGT |
| 14 | CCCTCCCCGCCCTAAAACCTA | 13 | CGTGCGAAAGTGTTTTTCAC |
| 14 | CCGGGAGGGCAGGTGTTAGG | 13 | TGGTCA |
| 14 | AAATACATCGTTATAAATTGA | 13 | CCGGCCCAACACCTACCCTC |
| 14 | AGATCGGAAGAGCACACGTCTGAACTCCAT | 12 | TTTTC |
| 14 | CCTCCCGGCCATAACACCTAC | 12 | ACCTCCAGATCGGAAGAGCACACGTCTG |
| 14 | TCATCATCAAATGGAATCGAATGGAA | 12 | AAACTTTGAAG |
| 14 | CCCCCCCCCCAAAC | 12 | AATATATATA |
| 14 | GGCCGGGAGGGCAGGGAGTAG | 12 | CCCTCCCGGCCCTAACACCCC |
| 14 | TACCCCCCCCCCCCCA | 12 | CCCTCCCGGCCCTAAAACCCA |
| 14 | CTAACACCTACCCCCCCAGCC | 12 | CCCCCCCCCCCCCCCCCAAA |
| 14 | CAAAGAAAGAAGCAGAAGAAAAAG | 12 | CCGGCCCTAACACCTACCCT |
| 14 | CCTCCCGGCCCTAACACCTA | 12 | GTGTTAAAGTGTCGA |
| 14 | AGGGCCGGGAGGGCAGATATT | 12 | TTAGGGCCGGGAGGGTAGGAG |
| 14 | GGATAGGTCAGGTTAGGGCAGGCGG | 12 | GAAGAGCACACGTCTGAACTCCACTCAGATCG |
| 14 | CGTCTTCTT | 12 | ACCTACCCTAACCAA |
| 14 | GCCCCCCCCGCCCCAAAACCC | 12 | CCCCCAACCCCCCCCA |
| 14 | CCCCCCAATCCC | 12 | CCCCCCCCCCCCAACCCCC |
| 14 | TAGGGCCGGGAGGGCAGGG | 12 | CCATGC |
| 14 | ACCATTTCGTCACAAGAAGACGACGAG | 12 | CCCCCCCCCCCCCGCCCCAAA |
| 14 | ACCCCCCCCGCCCCAAACCC | 12 | CCTGCCCTAACCTGACCAACCCCG |
| 14 | CCCCCCCAAG | 12 | CCCCGCCCCACACCCACCCC |
| 14 | CCCCCCCGCCCCCACCCCCAC | 12 | AGAGTCTACCTGGT |
| 14 | AATCCCAGATCGGAAGAGCACACGTCTG | 12 | ACCTTCATTTCC |
| 14 | GATCGGAAGAGCACACGTCTGAACTCCCTA | 12 | CAGATCGGAAGAGCACACGTCTGAC |
| 14 | TTTTCT | 12 | TTGCTTCTTGCG |
| 14 | CACGTGATCAGTGCATGATCAGC | 12 | TCCATGACCTCCACCGCCACCACC |
| 14 | CCCCACCCCCCCCCCCCC | 12 | AAAAAATTATAC |
| 14 | ACCTAA | 12 | CCCCAAACCCCCCCC |
| 14 | CCCGGCCCTAACACCAACCCC | 12 | CCGGGAGGGTAGGTGTTAGGA |
| 14 | CCCGCCTGCCCTAACCTGACCCACC | 12 | ATAATAT |
| 13 | AATATAA | 12 | AAAACAAAAAAA |
| 13 | CTCCCGGCCCTAACACCCTCC | 12 | TGTCGCGTGTTAATC |
| 13 | AGCTGTTTTAA | 12 | CCTGCCCCCCCGGCCCCAAAA |
| 13 | TCGGAAGAGCACACGTCTGATCCAGA | 12 | AGGTCAGGTTAGGGAGGCGGGAAT |
| 13 | GAGGGCCGGGAGGGCAGGGAT | 12 | GGCCCGGAGGGCAGGGAG |
| 13 | TTATTTATTATT | 12 | GGGCGGGCATTGGGGCCTTA |
| 13 | AGGGCAGGGAGGGCAGGTTT | 12 | TTCTGAA |
| 13 | GCCGGGAGGGCAGGGGGTAGG | 12 | GCCCCAACACCTCCCCCCCC |
| 13 | AACGGT | 12 | GCCCCCCCA |
| 13 | CCTGCCCCCC | 12 | AGATCGGAAGAGCACACGTCTGAACCTCC |
| 13 | CCCCCCCCGCCCCAAAACCCT | 12 | CCTGCCCTAATACCTGC |
| 13 | CGAATA | 12 | TTAGTTATAAGGCTGCGA |
| 13 | CCCTCCCGGCCCCAACACCTC | 12 | AGATCGGAAGAGCACACGTCTGATCCTCC |

| 12 | **AACCCCA** | 10 | CTCCCGGCCCTTACACCTGCC |
| --- | --- | --- | --- |
| 11 | AGAAACTGAAGAAAAGGCTAAAAA | 10 | TGAAA |
| 11 | ACCGTTCT | 10 | CATCG |
| 11 | ACCCCCCCCGCCCCCA | 10 | GCAGAGATGGCAGGGAGG |
| 11 | CCGGGAGGGCAGGTGGTAGGG | 10 | AATTTTATTTTATTTTA |
| 11 | TTTCAATTCACAATTTCGA | 10 | CCGCCACCTCCATGTCCTCCACCA |
| 11 | TCTTCTTCGTCATCC | 10 | GGGAGGGCCGGGAGGGTA |
| 11 | CCCTAACACCTACCCTCCAGG | 10 | CTATTTA |
| 11 | CCCCAATGCCCGCCCCAAGGC | 10 | TTAGGGCCGGGAGGGCTGGAGGGCAGGTA |
| 11 | CCTGCCCCCCCCGCCCTAACA | 10 | TAAAAATCGTCGCATCGC |
| 11 | CCCTAACCTGACCTACCCCCCCTG | 10 | CCCCCCCCGGCCCCACACCCC |
| 11 | TAATACCTGCCCTCCCCGGCCC | 10 | GGGAGGGTTGGTGTTAGGGCCGAGAGGGCA |
| 11 | ACCTACCCTCCCGGCCCTCAT | 10 | CCCCCCCCCCCCCACACCCCC |
| 11 | CTAACTCTAAC | 10 | ATCGCAAGATCAGCT |
| 11 | ATCGGAAGAGCACACGTCTCTCCAG | 10 | TTAGGGCCGGGAGGGCGGGT |
| 11 | CGCCTGCCTAACCTGACCTACCCG | 10 | TAAAAT |
| 11 | CCCGCCCTAATG | 10 | CACCTCCTC |
| 11 | ATTTATATATATATATA | 10 | AATTAAAA |
| 11 | CCCCCCCCCAACACCCACCCC | 10 | AGCACACGTCTGAACTCCACCCAGATCGGAAG |
| 11 | GCAAGTGCAAGAT | 10 | TGAAAAAAAATACATCCTAACGC |
| 11 | CCCTCCCGGCCCCAAAACCCA | 10 | CCCCCCCCAACCCCCCC |
| 11 | AAGAAGCTGAAGTAAAACAAAGA | 10 | CCCTCCCGGCCCCAACACCAA |
| 11 | AGATCGGAAGAGCACACGTCTGTCC | 10 | AATTCGC |
| 11 | GCTCACT | 10 | ACCTACCCCCCCGGCCCCAAT |
| 11 | ATTTTTTC | 10 | CGAAACAAAT |
| 11 | GGGGTGGGAGTT | 10 | CCCCGCCCCCCCCGCCCCAAC |
| 11 | TTTGCCTTTTCTTCAGCTTCATT | 10 | AGGGCCGGGAGGGCAGGTACT |
| 11 | GGATTGTATTCACCTTGATTTCTGCTT | 10 | GGTAGGTCAGGTTAGGGCAGGCGGA |
| 11 | AACCAAC | 10 | GCCCTCCCCGCCCCAAAACCT |
| 11 | CCCCCCCCCCCCGGCCCCAAA | 10 | AGATCGGAAGAGCACACGTCT |
| 11 | GCTTTT | 10 | CAAAAC |
| 11 | TAGGGCCGGGAGGGCAGGGG | 10 | AGGTATTAGGGCCGGGAGGGCT |
| 11 | AGGGCCGGGAGGGCTAGAGGGCAGGTATT | 10 | AGGGCCGGGAGGGCTGGGAGGCAGGTATT |
| 11 | TCACCA | 10 | TAGGGCCGGGAGGGCAGGAG |
| 11 | CAACACATGGCACTAATGAGAGAC | 10 | TATATTATTTTT |
| 11 | TTATTAAATACATAAA | 10 | CCCTCCCGGCCCTAATACCTGCCCTCTCTG |
| 11 | TATATTATATAATAATT | 10 | AGATCGGAAGAGCACACGTCTGAACTCCAGTCTC |
| 11 | CCCTCCCCCCCCGCCCCAACA | 10 | ACCTACCCCCCCCGCCCCAAA |
| 11 | CAATA | 10 | CAAAC |
| 11 | CCTGCCCTCCCGGCCCGAATA | 10 | AGGGCAGGTATCAGGGCCGGG |
| 11 | CCCTGCCCTCCCGGCCCCAAC | 10 | TCTTCTTCGTCACCT |
| 11 | ACGACGAGAGTCACACGGAGG | 10 | AGATCGGAAGAGCACACGTCTGATCCTC |
| 11 | GAAGTT | 10 | TTTTCAT |
| 11 | TGTGTCGGAACGTGACACTCCGATCCCCTACA | 10 | CCCCCCCCAAT |
| 11 | AAAAAATACAGCATAATAG | 10 | TCAAAA |
| 11 | ATATTCAATCAGTGTTTTAATCAATTCGC | 10 | AATTTTTTTTT |
| 11 | GGAAACATTTA |
| 10 | CCCCCCCCCAACACCCCCC |
| 10 | AGGTATTAGGACCGGGAGGGT |
| 10 | ACACCTACCCTCCCGGCCCCC |
| 10 | ACCCACCCCCCCCCCCCCCAT |
| 10 | GCCCTAATACCTCCCTCCCTGCCCTCCCG |
| 10 | GTGCAGCAGAGACTG |
| 10 | CAACAG |
| 10 | TATATATATTTATA |
| 10 | GCCCCCCCGGCCCTAATACCC |
| 10 | AGATCGGAAGAGCACACGTCTGATTCCC |
| 10 | TTGTTC |
| 10 | CCTGCCCCCCCGGCCCCAACA |

| **Bembidion scenicum (#SRR5230409)** | | | |
| --- | --- | --- | --- |
| 2774 | GTTTAGGTTAG | 105 | TTTGGGTAGGTAGC |
| 985 | AATGG | 102 | GATCGTCCTTTGACTCAACT |
| 787 | **TTAGG** | 101 | TAAACC |
| 694 | AATGAAAG | 98 | CTAGGTTCTGGTTTGATTGTTGAGTTAGG |
| 659 | TTG | 93 | AACTGCATTTCTATTGGCTGTG |
| 635 | AACATACCAC | 90 | AACA |
| 619 | TTTTTCTTGAAATTTCAACA | 90 | GGTTTAGGTTG |
| 592 | GTTTGGGTGA | 89 | TTCTGG |
| 556 | TAACATACCA | 88 | GAAAATTCATCGCA |
| 547 | ACACTAA | 88 | GGACAATTGATCCCCT |
| 546 | AAAAGGTGAAATTTCAAGAA | 86 | CACTTTCG |
| 508 | TGGGTGAGTTTGGGTAGGTAGCTT | 85 | CTTGAAATTTCACCATTTTT |
| 418 | AACCTAACCTC | 84 | CAAATGTCTGTCGAG |
| 397 | ACCCAAACTCACCTGAAGTC | 84 | CTTTTTTA |
| 368 | TTC | 83 | CATTATGTGAGGG |
| 367 | GGAAGAGAAGA | 82 | TTCG |
| 327 | TTAGTGTTTAGTGCA | 79 | CATC |
| 278 | TTTAAG | 78 | GAGAGAAGAGG |
| 277 | GTGTGTCC | 78 | AGTTTGGGTAGGT |
| 245 | CTAA | 77 | TTTTTG |
| 244 | TCTTCTCTTTCGTTT | 76 | TTCTCCTTC |
| 242 | ACTGGAACACACGAC | 76 | AGATATCTGAAATTCATTTTA |
| 240 | CGCT | 75 | AGACGAATTGTGTAGG |
| 235 | TTTAGGTTTGG | 75 | TAAATTTAAAACA |
| 229 | TGTA | 72 | CAGTTCATTTCATTAAGATA |
| 223 | CCTAACTTAACTCAACAATCAAACCAAAA | 70 | TTGACGTCCG |
| 223 | TTTACTTTAAG | 69 | CTCTTC |
| 221 | CTGTA | 69 | TAGTTTTAAGTTCGGG |
| 220 | TAGGTTTGGGTGAGTTTGGGTAGG | 68 | CTTCAGCTTCTTTCTTTGCCTTTT |
| 210 | TAGGGACAGTTTTCCGGAGGGAGGT | 68 | GGGATTTTGCCTTGTTTCTGCTATGCTATAGTTC |
| 205 | TATAAGAGT | 67 | AAACAAGGCAAAATCCCGAATGCAG |
| 195 | GAAAGAAGAGAAGAA | 65 | CGGCAA |
| 184 | TTTTTTC | 65 | TCTTCTTTTTTCG |
| 176 | CCTACCCAAACTCACCCAAATCTA | 65 | CTCACCTGAA |
| 167 | CCTAACCTGAA | 64 | AAACG |
| 164 | TCA | 63 | TCG |
| 158 | TTCT | 62 | AAAAAAAAGA |
| 158 | AATA | 61 | CTACC |
| 144 | TTCTTTTC | 60 | TTTTGTTTTGT |
| 144 | CAATTCAAGATATCTGTAATT | 60 | TTTGCTCGACAGACT |
| 136 | AGGTGAAATTTCAAGAAAA | 59 | TGAAATTTCAAGAAAATGT |
| 130 | GGTGAGTTTGGGTGACATCA | 59 | TGAGGTTAGGG |
| 129 | TTTTG | 58 | CTGTCGAGCAAAAGTC |
| 128 | AACGGCGGGTA | 58 | GAAAATACAAAATCA |
| 128 | CTACT | 58 | CTTTATTTT |
| 126 | CAATTCAAGATATCTGAAATT | 57 | TCTGCCTCACGTGTTAAACTTATTGGCAACG |
| 124 | CAG | 56 | ACTTTATG |
| 121 | AGATATCTTAAATTCAATTCA | 56 | ATGTT |
| 118 | TAC | 56 | GTTTGGGTAGGTAG |
| 116 | TATCTTCAATTGAATTTCAGA | 56 | AATGAATTTAAGATATCTTAA |
| 115 | GAGTAATAA | 56 | CAAACCTACCCCTAAC |
| 115 | AATG | 56 | AAGAA |
| 115 | AAT | 55 | ATAAATAACA |
| 113 | TTAGATTTAGG | 54 | GTTTGGGTGGGTAG |
| 113 | CTTTTTTCTTGAAATTTCAA | 54 | AAAAGGAATAAAAGAAAACTAAAGA |
| 113 | TTTCTT | 53 | AATGGAATCGAATGGAATCATCATCA |
| 109 | CAGA | 53 | GTTG |
| 105 | TTTCGCGATCACCGTTTA | 52 | TTTCAAGATAAAAGGTGAAA |

| 52 | AGATATCTGAAATTCAATTAA | 35 | ATCTA |
| --- | --- | --- | --- |
| 52 | CGTGCGAAAGTGTTTTTCAC | 34 | TTCACCGCTCACAA |
| 51 | ATTTTCGGTTTTTGT | 34 | CAGAA |
| 51 | ATACCTAAAAAGAAAA | 34 | TTCGACATTTGGGAA |
| 50 | GAGAAGAGGAG | 34 | TTTCTTCTTTTTCAA |
| 50 | GATTTTGTATTTTCG | 34 | TAACTACAGATGGAAATTCTGGAGAAG |
| 50 | GGCGGATAAAC | 34 | AATTCAAGATATCTCAAATTC |
| 50 | AGAT | 34 | CCGTAG |
| 50 | CACGCACGCA | 34 | TTTTTTTAT |
| 50 | TTGATG | 34 | TTTTGAGAGAGCAAAAAA |
| 49 | CGTAT | 34 | AAGAAAAGAAAAAG |
| 49 | GTGAATTGTGCGCG | 34 | GTTCGAGTTATCGAA |
| 48 | AGATATCTCAAATTCAATTGA | 33 | CACCCAAAGCTACC |
| 47 | TGTG | 33 | GGAGACGAATTGTG |
| 47 | TGAGTTTGGGTGGGTAGCTTTGGG | 33 | AAAAAAAAG |
| 47 | AAAAT | 33 | GTATGT |
| 46 | CAAAATGGCCGCTA | 33 | AGATATCTTGAATTGAATTAA |
| 46 | AAGG | 32 | TTCATTTGAAGAAATCTTAAA |
| 45 | AAAGGC | 32 | TTTTTTAG |
| 45 | AAAAAAAAAAGTT | 32 | GGAAAAAAGG |
| 44 | TTTTTTATTT | 32 | CTGTCGAGCAAGTGT |
| 44 | CCTAACCTCCCTCCGGAAAACTAGT | 32 | TGAAATTTCAGTAT |
| 44 | TTTTTGT | 31 | CCCACCCAAACTCACCCAAACCTA |
| 43 | ATTGAAATTTCAAT | 31 | TGAACTGTATCTAATGAAA |
| 43 | AGGGAAT | 31 | CCTTGTTTGTGCATTCGGGATTTTG |
| 42 | TGTTTTCA | 31 | TAGAA |
| 42 | TCAATT | 30 | GTTGATAATTTCTGTTTTCTTGCCTTG |
| 42 | CCTCCCTTCGGAAAACTGTCCCTAA | 30 | GGTGAGTTCG |
| 41 | ATCA | 30 | AACCTACCCCTCACCA |
| 41 | TTTTTTCT | 30 | TTTTTTTTTTAACA |
| 41 | TTAGGG | 30 | ATATTGAAA |
| 41 | TGTACGGATACGTAGTTTCTCTA | 29 | ATTCCATTCG |
| 40 | AAAATGTGAAATTTCAAGA | 29 | AGAAGAAGAAAAAGATGAAGAGAAGCAACA |
| 40 | GTTAGGTTTTGGTTTGATTGTTGAGTTAAGTTAG | 29 | AAAACAAAAA |
| 40 | CAGCAA | 29 | AAAAAAAAAAT |
| 39 | TTGTAT | 29 | ATCTTCTTTCTTCGTCTTCAA |
| 39 | TATTTTAA | 29 | AATGCGACG |
| 39 | TAGTTTTAAGATCCGTT | 29 | GAAAGCTAGAAAGGAAAAAAA |
| 39 | ATTTTGTGGCGGCC | 29 | TTCTGTT |
| 38 | GGT | 29 | TTTTATT |
| 38 | TCTTCTTCTCT | 29 | CCTCTAGACCGTTTTGGACTA |
| 38 | ACAAC | 28 | GTATTTA |
| 38 | CTCCACCAATTCTTTC | 28 | TTAGGTTTTGGTTTGATTGTTGAGTTAGG |
| 37 | TTCCATTCGATTCCATTCGATGA | 28 | AAGAAAACGAAAGAGAAGAGA |
| 37 | TAGGGCCAGTTTTCCGGAGGGAGGT | 28 | AAAAACTCA |
| 37 | TAAG | 28 | TTTCTTCTGTGTCTTCTATAA |
| 37 | CACCCAAAGCTAC | 28 | ACTGTCGCG |
| 37 | ATAGAAAAAAGCGCAT | 28 | CAAAATGGCTGCCA |
| 37 | TTTTTCTTGAAATTTCACCTT | 27 | AAACGCGAAACAGAACGGTGCGCGAT |
| 37 | GATGATGATTCCATTCGATTCCATTC | 27 | TTGCTCGACAGTCTT |
| 37 | TTAAAGATATCTCTAATTCAA | 27 | TTCTCG |
| 36 | GAATAC | 27 | ATTTAGTT |
| 36 | AATAAG | 27 | ATATAAAAAAATAC |
| 36 | AGGTGAGTTT | 26 | TGACCTTCAGAGCAAAAGTCA |
| 36 | CCTAACTCAACAATCAAACCAGAACCTTA | 26 | GTTAGGGGTAGGTTTC |
| 35 | AATTAC | 26 | TTAGTGTTAAGTGCA |
| 35 | AAAAAACA | 26 | TTACAAT |
| 35 | ATGTATTTATTTAT | 26 | CGGTGAAAAACACTTCGCA |
| 35 | CACTAAACACTAAG | 26 | TCGTGTGTTCTAGTG |

| 26 | TTACT | 16 | CCATTCGATGATT |
| --- | --- | --- | --- |
| 25 | AATTGAATTAAAGATATCTCA | 16 | AGCTGTTTAT |
| 25 | CCTAACAAGCGAAAAACCTCC | 16 | CCAAATGTCGAATC |
| 25 | CAAAAAAAAAA | 16 | GTCGTCG |
| 25 | ATTTTGTA | 16 | TATTTT |
| 25 | ATTTTTCTTAAAATTTCAAC | 16 | TGAAATTTCAAGGTCTGAATAGG |
| 24 | AAACAGCTTTA | 16 | TAACGATGTATTTTCAATTTA |
| 24 | AATGAATTTAAGATATCATAA | 16 | TTTCCCGAATACTAA |
| 24 | AGATATCTCAAATTCAATTAA | 16 | TTTTATTT |
| 24 | TATCTCGAATTGAATTAAAGA | 16 | TTCTC |
| 24 | AGAAAAAAAAA | 16 | TGCG |
| 24 | GCGCCGAGTTGATCC | 16 | CCTG |
| 23 | ACCGTTTCTGTTTCGCGTTTATCGCGC | 15 | TTCAATTGAATTTAAGATATC |
| 22 | AAATTTCAACATTTATCTTG | 15 | TACACTTCCTGTGTTAATAACTGATTC |
| 22 | TTGAAATTTCACCTTTTTAGAAC | 15 | AGATATCTCTAATTCAATTGA |
| 22 | AATGCGTAT | 15 | CTTGAAATTTCACATTTTTT |
| 22 | GCTGGT | 15 | ACACCG |
| 22 | CTGTCGTGA | 15 | TTGCTCGACAGGACAT |
| 21 | ATGAA | 15 | CTGACAGA |
| 21 | AAAAGTGCGCGGTCGTAA | 15 | TATTCAG |
| 20 | ATTTCAATTTATAACGATGT | 15 | AAAACCTAACATAACTCAACAATCAAACC |
| 20 | TTCGCGTTTATCGCGCACCGTTTGTAT | 15 | CCTAACCTCAG |
| 20 | ATGTATGG | 15 | TTGCGAA |
| 20 | ATACA | 15 | GTTTAAT |
| 20 | TTGAAATTTCAACATTTTTT | 15 | ACGGTTATTCGGTCGC |
| 20 | TATAAACAGGTTTGAAACAGC | 15 | CAACG |
| 20 | ATATCTGAAATTCATTTCAAG | 15 | AAGAAAGAAGCAG |
| 20 | TTTTCGT | 15 | ACCTAAACTAA |
| 20 | AAAATCTTCTATACATTTTTCATTTG | 15 | TCTTCTCTTTCATTT |
| 20 | GGAG | 15 | CGTTGT |
| 20 | CAAACCAGAACCTAACCTAACTCAACAAT | 15 | CTCTTCTTTTC |
| 20 | TTTGCT | 14 | TGTGGA |
| 19 | GTCGCGACAGACACGACA | 14 | ACAATTCGTCTTCC |
| 19 | GAAATTTTTTTTCTGTCGTGT | 14 | TTAAAGCTGTTTCAAACCTG |
| 19 | ATATCATAAAATGAATTTCAG | 14 | CTTCAA |
| 19 | TTGAAATTTCAACATTATTC | 14 | AAAAAACAA |
| 19 | TGTTGTTGC | 14 | TTTTTTCCTC |
| 19 | TTATAACTACCCGAAC | 14 | TCGAGCAAATGTGCTG |
| 19 | TGACGCCCGT | 14 | CCCACAATTCACCG |
| 18 | CTTGAAATTTCACCTATTCAGAA | 14 | TTTTTGCA |
| 18 | AACCCCTAACCT | 14 | TTTAATTTAAG |
| 18 | GGGGATCAACTGTCCG | 14 | TTTGATTTTCAGAAACTTCATTG |
| 18 | AAACTCCAACTCT | 14 | TTTTTTTTGAAATTTCACC |
| 18 | ACTCACCTGAACTCACCCAA | 14 | TGTATA |
| 18 | AAAATAACTAA | 14 | CGTTA |
| 18 | CAATT | 14 | CTTCTTT |
| 18 | CGAAATA | 14 | TTCCATTCGATGATGA |
| 18 | AATGAATTTCAGATATCTTCA | 14 | CACTAC |
| 18 | CTACCCACCCAAA | 14 | ATTCGAGATATCTGTAATTCA |
| 18 | CAGTTTTCCGAAGGGAGGTTAGGA | 14 | AACTGC |
| 18 | ATTCCATTCGAGTCCATTCGATG | 13 | AGACTTTTGCTCGAA |
| 17 | GGGGGCGAAATGCT | 13 | AGACTTTTGCTCGAAT |
| 17 | TTTCTAGCTTTCCTTTTTTCC | 13 | CACT |
| 17 | ATAACATACCACAACATACC | 13 | AAAATAC |
| 17 | AATAATTATTTA | 13 | TTTCTGCATTCGGGATTTCGCCTTG |
| 17 | TCACCTGAAG | 13 | GGAGT |
| 17 | TGAAATTTCACTTTTTTCT | 13 | AAGAAAAAAG |
| 16 | TTTTGTAGCAGCCA | 13 | CCGTTGACGCCCGTGACGG |
| 16 | TCC | 13 | AATTTTTTTTTTCAA |

| 13 | GCCATAC | 10 | GTTTGGGTGGTAG |
| --- | --- | --- | --- |
| 13 | TTTTGTTGT | 10 | GAAAAAAGCAAAGCTAGAAAG |
| 13 | ACCAA | 10 | CAGTTCATTTCATTAGGATA |
| 13 | CAAAATGGCCCCCAAAATAG | 10 | AAAAAAACTC |
| 13 | TCGCATT | 10 | GTAAAAT |
| 13 | TTTTTGTACTTTCGG | 10 | TGTTTTTC |
| 13 | TTTTTTCTTGATATTTCACC | 10 | TTCTTCTTCTACTGCTTT |
| 13 | GATTCCATTCCATTC | 10 | CAAAACAAAT |
| 13 | TCTTCTTCGTCATCT | 10 | AGCC |
| 13 | ACGATG | 10 | GTCTGCAGTGA |
| 13 | TAATG | 10 | TGAACTGTATTTCTATTGGCTG |
| 12 | TTGCCACCTTAAAATTTTT | 10 | GAATATTCTCATTCTAGTAG |
| 12 | TTCATC | 10 | CACTCTCG |
| 12 | AAAGGTGAAATTTCAAGAAG | 10 | CAGCAT |
| 12 | TCTTTA | 10 | ATCCATACATAC |
| 12 | TATAGCAATTGCTA | 10 | GACGAGATATTTAATC |
| 12 | TAGAAGTTTCTTCAAGTTTTTCTTCCAAAGTTC | 10 | GGCCATTTTTGGGGC |
| 12 | GTGTGTGTGTCC | 10 | TTCTCGT |
| 12 | TATACTGCGCGGAGCCTACGCGTATA | 10 | TTTCTTTAGTTTTCTTTTATTCCTA |
| 12 | ACAAGT | 10 | AAAAAAAAAAAC |
| 12 | AGCAACG | 10 | TTTTCA |
| 12 | AAAAGTTGAAATTTCAAGA | 10 | TTTTAAA |
| 12 | TATGCGCTTTTTTCCA | 10 | TTCCCG |
| 12 | ATGAAAA | 10 | CTTGAAATTTCACCTTTTAAA |
| 12 | TCGTCGACGAAAC | 10 | TTTCCTCAAAG |
| 12 | AATCGTG | 10 | AACATCTTCCAACAG |
| 12 | CTCACCCAAAG | 10 | TTTCATCGACGAG |
| 12 | ACCATGCGCACCGAATA | 10 | AGAAAGAGAAA |
| 12 | TGAAATTTCAAGTTTTTAAAAGG | 10 | AACCTG |
| 12 | AAAACGAAAGAGAAGAGAAG | 10 | AAACCCAACCC |
| 12 | AGCAAGTTAACGA | 10 | GCAACC |
| 12 | GAAATTTTAACATTTTTCTT | 10 | GCAGAGCA |
| 12 | ACAATCA | 10 | CACACA |
| 11 | ATACT | 10 | GATATCTTTAAATGAATTTCA |
| 11 | AAAACGT | 10 | TCCACCCAAA |
| 11 | ACACAGAC | 10 | TCTTCAAAGTT |
| 11 | ATCGAC | 10 | CTTTTTCTTTTTCTCATCTTC |
| 11 | AACCTACCCTCACCA | 10 | ACCAACA |
| 11 | CTGTTT | 10 | AAGAAAAAGAAAGAGAAGAGA |
| 11 | CTTCATCTTTTTCTTTTTCTTGTTGCTTTT |
| 11 | GCGCAAATAGAAAAAA |
| 11 | AACGCA |
| 11 | TTTTACAACTTGAAAAATTTTCAACAT |
| 11 | AACTGCAACTCTA |
| 11 | AAATACATAATTAAAACTTCG |
| 11 | GAGTT |
| 11 | AACGCTTTGCATTAACCAGATATGTTGAAAGAA |
| 11 | CCGCAAT |
| 11 | CTGCTGG |
| 11 | AATTCAAGATATCTTGAATTC |
| 11 | CGG |
| 11 | AATGAATTTAAGATATCTTTA |
| 11 | AAATGTTGAAATTTCAAAT |
| 11 | AAACAGCTTTAAAACAGATAT |
| 10 | TCTATTCTTCTAACG |
| 10 | TCTTATT |
| 10 | TAGAAATTCGAAATACATCGT |
| 10 | ATCCAGT |
| 10 | CTAACCAAACCTACC |

| **Bembidion musae (#SRR2939016)** | | | |
| --- | --- | --- | --- |
| 732 | ATA | 13 | ATATTAATAATT |
| 293 | TCTTATTCCC | 13 | AGGGGGGGGG |
| 291 | TGTTTGC | 13 | TACT |
| 289 | **AGGTT** | 12 | TCTTTTTTTTTT |
| 261 | GCCATAGGCTGAGATGAAATACACTATGTTCTTA | 12 | TTGTTGAAGAATCAA |
| 234 | AAAAT | 12 | ATGAAAA |
| 169 | TAAA | 12 | AAAGAGT |
| 164 | TGT | 11 | ACCTGTTTTGAACCTTTTTTGCCCCTGA |
| 163 | TATATATAAA | 11 | TACTTTAATTCAATTGACTC |
| 117 | CGAG | 11 | TTAATA |
| 117 | AGAGAGAA | 11 | CTTT |
| 99 | TTTTC | 11 | ATGTTCTTAGCCATAGGCTGAGATAAAATACACT |
| 85 | TTTTTTTG | 11 | ATTTATT |
| 76 | AAGAAATACACTACGTTCTTAGCCATAGGCCGAG | 11 | GGGGGGGGA |
| 71 | ATTATAT | 11 | GTTTTCT |
| 71 | TTTTAT | 11 | AATATATATATAAA |
| 65 | TTTTATT | 11 | TGTTCTAAGCCATAGGCTGAGATGAAATACACTA |
| 64 | TACACTATGTTCTTAGCCATAGGATGAGATAAAA | 10 | ATTATA |
| 54 | TATG | 10 | GCATCCA |
| 54 | CAG | 10 | ATAGGCTCAGATGAAATACACTATGTTCTTAGCC |
| 53 | AAAAGA | 10 | TCTTCG |
| 48 | TTATA | 10 | ACTAATAATAATTAAAATCT |
| 46 | TCT | 10 | CTTTCTCTCT |
| 45 | AAAAAAAT | 10 | AAATGTA |
| 44 | TTTTATTTT | 10 | ATTTTTA |
| 41 | CTTTTTTTTT | 10 | AAGAACATAGTGTATTTCTTCTCGGCCTATGGCT |
| 39 | TTTTTTTTC |
| 39 | CATTAA |
| 39 | TTGTTGAAGAAACAA |
| 37 | AGTTGAGTTTTAAGTTC |
| 36 | GTTTTT |
| 36 | TGTATTTTATCTCATCGCATTGCTAAGAACATAG |
| 35 | AAACATG |
| 34 | ATTTA |
| 33 | TTTTTTTATT |
| 32 | AAAAGAAA |
| 31 | TTTTCTT |
| 30 | TGA |
| 29 | GTAAATT |
| 29 | AAACA |
| 27 | ATATAAA |
| 27 | TATTTCTTCTCGGCCTATGGCTTAGAACGTAGTG |
| 26 | CATTTGA |
| 25 | CAATTGTTGAAGAAC |
| 23 | TTTTTTCTTTT |
| 20 | TAAT |
| 20 | AACAAAA |
| 19 | GGGGGAGGGGG |
| 19 | AACTCG |
| 18 | CGA |
| 18 | TGGGAAATAACTGGAGCAC |
| 17 | CGCCCCTTGCTCTATGGCCA |
| 17 | ATTAATATATATATATATA |
| 15 | TAC |
| 15 | AAATAAAAAAA |
| 15 | CCTGGAGA |
| 14 | TAAATTA |
| 13 | ACAAAAT |

| **Bembidion sp. (#SRR2939015)** | | | |
| --- | --- | --- | --- |
| 1530 | TCGGTGACAGGAATAA | 47 | GACATATGACATGGTTTTCGTCGTCGA |
| 1066 | TTCTTGCTTTCATTCCCACGACTCGTGTAAGT | 46 | TTTTCGTTGTCGAGACATATGACATGG |
| 794 | CAAGAAACTTACACGAGTCGTGGGAATGAACG | 45 | ATGTTTCGAATGATGTTTTACACGACTCGTGTG |
| 717 | TCATGTGTCTTCGACGACGAAAACCAAG | 45 | GATGTTTTTTCGTTTTTTACACGACTCGTGG |
| 669 | AGAAACTCCCACGAGTCGTGGGAATGAAAGCA | 45 | ACGAAAACCAAGTCATATGTTTTCGAAG |
| 606 | GTAC | 44 | ATGTTTCTTGCGTTTTTTACACGACTCGTGGG |
| 567 | CGAGTCGTGGGAATGAACGCAAGAAACTCCCA | 44 | AGTTTCTTGCTTCATTCCCACGACTCGTGTA |
| 549 | TCATATGTCTTCGACGACGAAAACCAAG | 43 | AATAATCGGTGACAGA |
| 516 | TTTTTTGCGCTCTTTTTTATTAAGGAATA | 42 | TATATTCTTA |
| 441 | GACATATGACTTGGTTTTCGTCGTCGAG | 41 | CGATGTGAAAGAATCACACGAGTCGTGTGAA |
| 406 | CACATGACTTGGTTTTCGTCTTCGAAGA | 40 | AAAAGAGCGCAAAAAATATTCCGTGACTA |
| 392 | GCTCAAAACGACGATTTT | 40 | CTCGTGTTATGTTTTACACGA |
| 382 | **AGGTT** | 40 | AAAGAGCGCAAAAAATATTTCTTAATAAA |
| 356 | AAAACCAAGTCATATGTCTCGACGACG | 38 | TAG |
| 341 | ACTTGGTTTTCGTCGTCGATGACATATG | 38 | TTATAAG |
| 283 | ATGACTTGGTTTTCGTCTTCGAAGACAT | 37 | CGACTCGTGTGATGTTTCACA |
| 227 | AAC | 37 | ATGACTTGGTTTTCGTCATCAAAGACAT |
| 208 | TTA | 36 | CCAGCAT |
| 182 | AAAAAGAGCGCAAAAAATAAGCCGTGACT | 36 | ACTTGGTTTTCGTCGTCGAAAACACATG |
| 164 | TGTGTGATCGATCACACATGA | 36 | ATGACTTGGTTTTCTCGTCGAAGACAC |
| 159 | ACCAATTATTCCTGTC | 35 | ACCAAGTCATATGTCTTCGACAGACGAAA |
| 149 | GACGAAAACCAAGTCATGTGTCTTCGACA | 35 | CTCGTGGGAGTCTTTCACAGCGTTTACACGA |
| 128 | TGACATGGTTTTCGTCGTCGAAGACATG | 35 | ACATACGT |
| 120 | CTTGGTTTTCGTCGTCGGAGACATATGA | 34 | CATTC |
| 118 | TGACTTGGTTTTCATCGTCAAAGACACA | 33 | TACAATACTTATT |
| 112 | ATGTCTTCGACGACGAAAACCAAGTCAC | 32 | ACGAGTTGTGGAATACATCCCC |
| 100 | TTTA | 32 | CGATGTGAAAGAATCCCACGAGTCGTGGAAA |
| 99 | AATATTTTTTGCGCTCTTTTTATTAAGG | 32 | GCCTACGCTATAT |
| 95 | GACACATGACTTGGTTTTCGTCGTCGA | 32 | GAAACTCACACGAGTCGTGGGAATGAAAGCAA |
| 90 | TAAACGATGTGAAAGAATCCCACGAGTCGTG | 32 | GAGAGCAAAAATCGGA |
| 85 | GTTGGGGTTTGGTTTTGGTTAATTTA | 31 | TCGAATAT |
| 83 | GTCGAAGACACATGACTTGGTTTTCGCT | 31 | TCT |
| 82 | AGACACATGACTTGGTTTTCGTCATCGA | 30 | TATGGTCTTGTATCTC |
| 77 | TGTA | 29 | ATTGTCGTGTG |
| 77 | CACGAGTCGTGTAAACGATGTGAAAGAATCA | 29 | TGCGATAGCTCATCT |
| 77 | CACTGATTATTCCTGT | 29 | GGCGAGAACTGTCACTTTTAA |
| 76 | GCAAGAAACTTCCACGAGTCGTGGGAATGAAA | 26 | TTCATTCCCACGACTCGTGGGAGTTTCTTGC |
| 75 | GGCTGCGGTTGCCT | 25 | GTTTCTTAGCT |
| 73 | ATGTTTCTTGCGTTTTTTTACACGACTCGTGGG | 25 | AAGGTCGACAGGCCGAAAAATAAA |
| 72 | TGGTTTTCGTCGTCGGAGACACATGACT | 24 | TTTTTCGGGATGTCGAGTTTTTGACGATT |
| 66 | CTGTTTTTAG | 24 | GATTTGAAAAGTCGTGT |
| 65 | CAT | 24 | CAAGAAACTTACACGAGTCGTGGGAATAAACG |
| 60 | GGAATGAACGCAAGAAACTTCCACGAGTCGTG | 23 | TTTTTGCGCTCTTTTTTATAAAGGAATAT |
| 60 | CTTCGACAGACGAAAACCATGTCATATGT | 23 | CATGACTTGGTTTTCGTCGTCGAGGACA |
| 56 | AATATTCGGTGACAGG | 23 | GAGCAAAAATCGGAAA |
| 55 | CGACAACGAAAACCAAGTCATATGTCAT | 23 | TTTTTATGAA |
| 55 | AAGCCCAGG | 22 | GAAAACCAAGTCATGTGTATTCGACGAC |
| 55 | CTTTCTCCGATTTTTG | 22 | TTGCTTTCATTTCCACGACTCGTGGGAGTTTC |
| 54 | AACATCCCACGAGTCGTGTAAAACAACGCAAGA | 22 | TTTTTCTGA |
| 54 | TATAATAAATTTAC | 21 | TAAAAAAAAA |
| 53 | CTTGGTTTTCGTCATCGAAGACATATGA | 21 | GTTTTCGTCATCGAAAACATATGACTTG |
| 53 | AACC | 21 | TATGACATGGTTTTCGTCGTCGAGGACA |
| 52 | GCGA | 21 | TGGTTTTCGCTGTCGAAGACATATGACA |
| 52 | CTCGTGTTAGTTTCTTGCTTTCATTCCCACGA | 20 | CGACGTTTCGAACCAG |
| 52 | AGATCAGCTATCGCA | 20 | AATATTTTTTGCGCTCTTTTTATCAAGG |
| 51 | ATGACATGGTTTTCGTTGTCGAAGACAC | 20 | GTCACATGTCTTCGACAACGAAAACCAT |
| 49 | CTGTCTTTAAG | 20 | AAAACCAAGTCATGTGTCTCCGAAGACG |
| 48 | GTCATATGTCTCGACAACGAAAACCAA | 20 | GTACGTAC |

| 19 | CGAAAACCAAGTCATATGTCTTCGACAG | 10 | TGGTTTTCGTCGTCAAAGACACATGACT |
| --- | --- | --- | --- |
| 19 | CTCTTTTTTATTCAAGAATATTTTTTGCG | 10 | GGAGGCTTGACTACTGGT |
| 19 | GACACATGACTTGGTTTTCGTTGTCGAA | 10 | AAGTCTTCACCTGCC |
| 19 | TGGTTTTCATCGTCGAAGACACATGACT | 10 | TTAAGGAATATTTTTTGCGTTTTTTTTA |
| 18 | AGAGCGCAAAAAATAAGCCTGAATAAAAA | 10 | GACACATGACTTGGTTTTCATCATCGAA |
| 18 | GAAAACCAAGTCATATGTCTCCGACAAC | 10 | AAATCGACATCCCGAAAAATAAAAACGTCAAA |
| 18 | ATTTTTCCTAAACGCA |
| 17 | GCTAAGAAACAGCTAAAAACA |
| 17 | GTCGTGGGAATGAACGCAAGAAACTCACACGA |
| 17 | CAACTCGTGGGAATGTATTCCA |
| 16 | AATAATCGGAGACAGG |
| 16 | CTTGGTTTTCGTCGTCAAAGACATATGA |
| 16 | CCTTTAGCTTCCCCAATCTGCATG |
| 16 | GAAAACCAAGTCACATGTCTTCGACAAC |
| 15 | ATCGAATATTCGAAT |
| 15 | AAAAAGAGCGCAAAAAATATTCCTTAAAA |
| 15 | CTTGGTTTTCGCTGTCGAAAACACATGA |
| 15 | AACTAT |
| 15 | TTCAAGTTGTGCGA |
| 14 | GGAGGCTTGACAATTGGT |
| 14 | TTTGATATCAAA |
| 14 | GGGGGACAAATGGC |
| 14 | TTTTTTGCGCTCTTTTTAGTCACGGCTT |
| 14 | AAAAAAGTCGACAGGCCGAAAAAT |
| 14 | AGACACATGACTTGGTTTTCGTCATCAA |
| 13 | GGAGGCTTGATGGGGGGCTTGTAGACTGGA |
| 13 | AAAC |
| 13 | GGTAGGGTTTAGGTTAGGTGGTGGTGG |
| 13 | TATTCCTTAATAAAAAAAAACGCAAAAAA |
| 13 | ACGAAAACCAAGTCATGTGTCTTCGAA |
| 13 | GTTTTCGTCGTCGAAGACACATAACTTG |
| 12 | GGAATATTTTTTGCGCTCTTTTTTATCAA |
| 12 | CATAGACTTTACGCGTAG |
| 12 | AAAAAAGAGCGCAAAAAATAAGCCGTGAC |
| 12 | ACATTACA |
| 12 | CTTTTCAAAACACACGA |
| 12 | TCGACGACGAAAACCAAGTCACATGTCA |
| 12 | TTGCTTTCTCCGATTA |
| 12 | TGGTTTTCGTCTTCGAAGACATGTGACA |
| 12 | TCACTTTTGTGGCGAGAACTG |
| 12 | CCTTTAGCTTCTCCAATCTGCATG |
| 12 | GATATTCGAATAGTTC |
| 11 | TTTATTTACCGCACGCAT |
| 11 | TGAAAACCAAGTCATGTGTCTTTGAACGA |
| 11 | AAACAGCTAAA |
| 11 | GTCGCGTTAGTTTTAA |
| 11 | AAAAATGTCAAAAACTCGACATCCCGAA |
| 11 | TGATGTTTCACATGACTCGTG |
| 11 | ACTCGTGGGAGTTTCTTGCTTACATTCCCACG |
| 11 | CTCAG |
| 10 | AAAGATAACACGACTCGTGTG |
| 10 | CAAAAAATAATCCTTAATAAAAAAGAGCG |
| 10 | TAAAAAGAGCGCAAAAAATATTCCGTGAA |
| 10 | ATTTTCAATACATTTCA |
| 10 | CAAGCCTCCTCCAGTTA |
| 10 | AACTCCCACGAGTCGTGGGAATGAAAGCTAGA |
| 10 | GAAAACCAAGTCATATGTTTTCGACAAC |
| 10 | GGAATATTTTTTGCGCTCTTTTTTTTAA |
| 10 | GAATAAAAAGCTACGC |

| **Lionepha casta (#SRR5230408)** | | | |
| --- | --- | --- | --- |
| 2898 | CACTCTTTCACCCA | 50 | TGGATCTGT |
| 2377 | ATTCC | 49 | GCTAAACTTA |
| 1866 | **GGTTA** | 49 | AAGTATTC |
| 1544 | GTGGGTTGAAGAGT | 48 | TTTTTTTCTTT |
| 1133 | GAGTGTGGGTGAA | 47 | CCTAAC |
| 579 | GAGTGTGGGTGGAA | 46 | CGG |
| 416 | TGT | 46 | ATGGAATCGAATGGAATCATTGA |
| 379 | GCGA | 44 | GGAATCATCGAAT |
| 334 | TTTTTTTTTGT | 42 | TGAAGAGTGTGGGG |
| 313 | CACTCGTCCACCCA | 42 | AAAAACAAAAAA |
| 292 | TGAAAGAGTGTGGGT | 40 | GCAACA |
| 258 | CACACTCTTCCCCC | 40 | GTTGAAGAGTGTGA |
| 222 | AAGGAGG | 40 | GGCAAC |
| 217 | AAAAAAAACA | 40 | CACACTCGTTCACC |
| 196 | TGC | 40 | CCCCCCCCCA |
| 195 | CACTCTTTAACCCA | 40 | AAAAAAC |
| 194 | CTT | 39 | ACCAGAAAC |
| 173 | TATGAACACCTTTTTGTCTGTG | 39 | TTTAGTAAGAAAAATACTGT |
| 166 | AAAAAAATA | 39 | AGAG |
| 160 | TAGTCAGACAAAATGTCGTCAG | 39 | CGATTGAGGGTGA |
| 132 | AACACAC | 38 | AGAC |
| 131 | TAGCCG | 38 | TCTTCCTCT |
| 129 | ATCATCGAATGGAATCGAATGGAATC | 38 | TTTTTG |
| 124 | ATTCGATTCCATTCGATGATTCC | 35 | GAGTGTGGGTAGAA |
| 119 | TTTTTTTGT | 35 | TTTTTC |
| 116 | ATTGTTCTGTTTTTTC | 35 | CTCCGG |
| 115 | TCATCAAATGGAATCGAATGGAATCA | 35 | **GGGTTG** |
| 110 | GAGTGTGGGGGGAC | 34 | AGAGTGTGGGGGAA |
| 109 | GAAG | 34 | GGGGA |
| 107 | CTTT | 34 | **GGTCGT** |
| 107 | AAAAAATAAA | 34 | GATTCCATTCGATGAT |
| 94 | AGTGG | 34 | ACCCCCCCC |
| 93 | GGAGGAGGTTCA | 33 | CAT |
| 90 | GTTTT | 32 | TATGGC |
| 88 | TTTTACCCACACTC | 32 | ATAG |
| 83 | ATCGAATGGA | 32 | GGGGAGGAGTGG |
| 83 | TGGA | 32 | AAACAGTATTTTTCTTACTAA |
| 81 | ATTTGTTGATTC | 31 | CCACCCC |
| 78 | TTTTTTTG | 30 | AGGGTGGGGGGA |
| 76 | TATGTATA | 29 | ACGGCG |
| 75 | TTAAAACAGTATTTTTCTTAC | 29 | GGGCGG |
| 69 | GAGTGTGGGGGAC | 29 | GCGGTG |
| 68 | GAAGAGTGTGGGAG | 29 | GGAATGGAGT |
| 67 | AATGGACTCGAATGGAATCATCG | 29 | ATAC |
| 62 | AAAC | 28 | CCCCACCCCCC |
| 62 | GAG | 28 | ACCCACACTCGTC |
| 61 | GCTGGCTTG | 27 | TTTTCTTTTTCAGCT |
| 61 | TGGC | 27 | TCGTCTTCTTTCTTCG |
| 60 | GAGG | 27 | CGCCCCCCC |
| 58 | CATTCGATTCCATTC | 26 | AGAAACCGTGCACAAGA |
| 56 | AAAACAGTATTTTTCTTACCA | 26 | TGAAAAAACAGAACA |
| 56 | TTCTTCTTCGTTCTTCTC | 26 | CCCCCCCCCCG |
| 55 | TAAA | 25 | AAAAAAAT |
| 54 | GGT | 25 | CCCCCA |
| 54 | GGTTGTTCC | 25 | CGTACC |
| 53 | GAAAAAAAAA | 25 | CCCGCCCCCC |
| 52 | GAC | 24 | GAAAAAAAA |
| 51 | AAGAAAAAGAAAG | 24 | TCTTCTTCTCCTCCT |

| 24 | AGAAGAACAAAGAAG | 11 | AAAAAAATT |
| --- | --- | --- | --- |
| 24 | AGAAGAAGGAGG | 11 | TCTCTCTGTC |
| 23 | AGTGTGGGGGAAG | 11 | TCATCAAATGGAATCGAATGGAA |
| 23 | TCCATTCAATGAT | 11 | TCTCTG |
| 22 | CCCGCCCC | 11 | AAGCAG |
| 22 | TTTCT | 11 | GACATTCACACAAGTCGTGTGA |
| 22 | CCATTCGATTCCATTCGATGATATT | 11 | GGTCGC |
| 22 | CACTCGTCAACCCA | 11 | CCCGCCCCCCCCC |
| 21 | CCCCCCCA | 11 | TCCATTCCATTCGAA |
| 21 | ACCCCCCCCCCC | 10 | GTAGGC |
| 21 | GAGAGG | 10 | CAGCTTG |
| 21 | TTTTCTTTTTCAGT | 10 | CTTTCCTTCTTCT |
| 20 | GGGGAG | 10 | AAAAAAATAAAAA |
| 20 | GAAAAAAA | 10 | GTAGAAGAA |
| 19 | AAAAAAAAAAACA | 10 | GAGGAC |
| 17 | CCCGCCCCCCCC | 10 | CACAGACA |
| 16 | TTCCT | 10 | CGGAGG |
| 16 | GGAATCATCAAATGGACTCGAAT | 10 | TTTCTTTTTACG |
| 16 | AAAAACAAAAAAAA | 10 | CGGTTTCTGAC |
| 16 | TTCTGTTTTGTCATG | 10 | GGAAACGAAGGA |
| 16 | ATCCTCACCCTCA | 10 | CACTCGTCCCCCTA |
| 15 | CGACGATGAGTTCGATGA | 10 | CTGGCA |
| 15 | GCTTTTTATTC | 10 | ACCCACACTCTTT |
| 15 | TCTTGCTTGTTCTTTCT | 10 | GTGTGGGTTGAAAA |
| 15 | GGGGGAAGAATGT | 10 | CTTAC |
| 15 | CACTCGTCTCCCCA |
| 14 | AGGGA |
| 14 | **GTTTGGG** |
| 14 | TTTAT |
| 14 | GACGCC |
| 14 | GTCTCTCT |
| 13 | GAAAAAAAAAAA |
| 13 | CCACACTTTTCAC |
| 13 | TCATCGTCGTCATCAAATTCGTCA |
| 13 | GAAGAGTGTGGGGA |
| 13 | TGAGGGGGCG |
| 13 | CCATTCAATGATTCCATTCGAGT |
| 13 | GAATGGAATCATCATCGAATGGAAAT |
| 13 | GGACAG |
| 13 | CCCCGCC |
| 13 | AAAAATAAAAA |
| 13 | GAATGGAATCATCTAATGGAATC |
| 13 | GCAGCC |
| 12 | TCTTCTTCTTTCT |
| 12 | ATGCCCTG |
| 12 | CCGACC |
| 12 | AAAAAAAAAAATT |
| 12 | CCAGCA |
| 12 | CCTG |
| 12 | CATTCCATTCCAGTA |
| 11 | CCCACACTTTTTCA |
| 11 | AACCCTA |
| 11 | GCTCACT |
| 11 | AATCATCATTGAATGGAATCGAATGG |
| 11 | CACA |
| 11 | CTCTTTCACCCACACTCGTCCCCCCACA |
| 11 | GAGTGTAGGTGAAA |
| 11 | ATCGAATGGAAT |
| 11 | ACGTAGGTTTTCCCA |

**¨**

| **Lionepha chintimini (#SRR2939021)** | | | |
| --- | --- | --- | --- |
| 945 | TGTTATT | 21 | TTTTGCGTAAAATATATATATTTTTTCGCGTTC |
| 848 | **TTAGG** | 19 | ATATATTTCACGCAAAAAACGCGAAAAAATAT |
| 550 | GAATG | 17 | TATGTTATAATATAAT |
| 521 | GTCGTAA | 16 | TTTTTTGCGTAAAATATATATA |
| 517 | ACGACTC | 16 | TTCACCGAAAAGACAA |
| 375 | TGT | 15 | ATTTTTTTTTTAA |
| 314 | TAT | 15 | AAAATATATATATTTTACGCAAAAAAACGCGAA |
| 209 | CATCCAGACAGA | 14 | ATATATTTTACGCAAAAAACGCCAAATAATAATA |
| 156 | GGGGAAGGAGTG | 14 | ATTTGATATCAA |
| 125 | CAATGAAAAAACAGAA | 13 | GAGGGGGGGGGG |
| 125 | GTTACTT | 13 | AAACCGCTCAG |
| 121 | AAGGAGTGGGGGGA | 13 | GGGGGGGGAG |
| 115 | GGTCTT | 13 | TTTTAT |
| 110 | AATA | 12 | TTTTATT |
| 104 | AAGGAGTGGGGGA | 12 | AGTGGGGGGAAAG |
| 98 | TTTCCCTTTCACT | 12 | ACAATGAAAAACAAA |
| 91 | CGT | 12 | TATAATTATTTCGCGTCTTTTGCGTAAAATATAT |
| 63 | AAAAAAAT | 12 | AGGGGGGGG |
| 61 | ATGGCCAGTAATCA | 12 | ATGAATTTACCGGCG |
| 59 | AATAAAAAAA | 11 | TTTTCGACTTATAGAGG |
| 59 | TCTG | 11 | AATATATTATTTTATTTCGCGTCTTTTGCGTAA |
| 54 | TCA | 11 | TTTG |
| 53 | AGCG | 11 | AAGGAGTGGGGGAA |
| 51 | ATTTTTTTTTT | 11 | CAAAAAAAA |
| 50 | AAAAAAAAAATT | 10 | TTTAAATTTTAA |
| 47 | GCGGTTTCTGACCGGTTTATA | 10 | GGGGGCGGGGG |
| 45 | TTTGTT | 10 | AAACCGCTACTGGTTCTTAAA |
| 45 | TTTTTTTTA | 10 | TCTTCTTTCTTCGTCG |
| 44 | CCCCCACTCCTTC | 10 | TATTTCT |
| 40 | TGC | 10 | CAC |
| 39 | CGGTTTCTGAC | 10 | AAAAAAAATAAAAA |
| 38 | TGAAT | 10 | AACAAAA |
| 38 | AAATTA | 10 | AAAACAGAACATGGAA |
| 38 | GATTTCTTGTGAGCG |
| 37 | AAG |
| 37 | AATTGTTTTCGGTG |
| 36 | TAAAAAAAAAAA |
| 34 | TTTTAAATTTTT |
| 32 | GTTG |
| 32 | TAGCGGTTTTTTAAGAACCGG |
| 30 | TTTTG |
| 30 | TTTC |
| 29 | CTAT |
| 28 | AGGGGGGGGGG |
| 28 | ATTCAG |
| 28 | AAAAT |
| 28 | CAAAAT |
| 27 | TTAAAAAAAAA |
| 27 | AAAACAAGTTTATTTTATGT |
| 26 | ACTTTTGCCTTTC |
| 25 | TAGCGGTTTA |
| 24 | ATGT |
| 23 | ATTTTTTTTTTTA |
| 23 | TAAAAAAAAAAAA |
| 22 | CATGACAAAACAGAA |
| 22 | CTA |
| 21 | TATTTATTCAAAACAAGTT |
| 21 | AAAAAAAATTT |

| **Lionepha erasa (#SRR5230423)** | | | |
| --- | --- | --- | --- |
| 94252 | CATTC | 301 | AGACAG |
| 4089 | CCCAAAC | 296 | ATTCCATTCC |
| 3075 | TTCGATTCCATTCCA | 292 | GGGAGA |
| 2949 | GAAA | 278 | ATTCCATTCGATTCCATTCGATGAT |
| 2390 | GAGTG | 268 | TTGATGATGATTACATTCGATTCCAT |
| 2195 | CAAAA | 267 | TTTATT |
| 2091 | ATTCCATTCGATTCCATTCGATG | 266 | AATGGAATCAAATC |
| 2017 | TGATGATTCCATTCGATTCCATTCGA | 264 | TCGAATGGAATCATCTAATGGAA |
| 1923 | GATTCCATTC | 259 | ATTCGAGTCATTTCC |
| 1879 | ACA | 257 | TCCATTCGATTCCATTCTATGATGAT |
| 1878 | TTCC | 254 | ATTCCATTCATTTG |
| 1833 | AAAC | 253 | GATT |
| 1826 | AAAT | 250 | ATGGAATCAAATGGAATCATCGA |
| 1823 | TTTGTTA | 247 | AATCAAATGG |
| 1601 | AAATGGAATCGAATGGAATCATCATC | 247 | CATTCCATTCCAGTA |
| 1506 | ATGG | 245 | GGAATGGAATGGAAA |
| 1332 | TATC | 244 | TTTCCATTCGATGATGATTCCATTCG |
| 1134 | TTAG | 243 | TGATGATGATTCCTTTGAATTCCATT |
| 1098 | TCCATTCGATGAT | 238 | GTCGTGA |
| 1013 | GATGATTCCATTCGAGTCCATTC | 229 | AGAGACAGAG |
| 968 | GAATGGAATGGACTC | 228 | AATGGAATGGAATG |
| 954 | TGC | 224 | TTTCTTTTTT |
| 935 | GGAGTGGAAT | 223 | ATCGAATGGAATT |
| 773 | GATTCCATTCGATGAT | 210 | CCCCCAAA |
| 731 | GAT | 209 | CAGG |
| 720 | AAAAT | 207 | ATGTACTTTCATATTTT |
| 713 | CAAAAA | 207 | AATGGAATCATCATCGAATGGAATTG |
| 673 | GTA | 207 | TTAGG |
| 651 | AATGGAATCGAATGGAATCATCA | 197 | CCATTCCATTCGAAT |
| 642 | ATGT | 191 | ATTCCATTCGATTCCATTCGATGATT |
| 625 | AAG | 190 | GAATGGAATGTAATC |
| 592 | AGCC | 188 | GATGATTCCATTTGATTCCATTCGAT |
| 588 | TCGAATGGAATCATCATGAATGGAA | 187 | GAATGGAATCTAATG |
| 566 | TTGGGGTT | 186 | TTTTCTTTT |
| 558 | GAGA | 184 | TGATTCCATTCGATTCCATTAGATGA |
| 558 | TGGCTGGCT | 184 | GTTTAGG |
| 555 | AATGGAATGGAATCA | 183 | CATCAC |
| 549 | GTTAGG | 178 | TCCATTCCATTCCATTTCAC |
| 541 | AAAAG | 175 | AAACAAAA |
| 532 | AGTCCATTCCATTCC | 175 | AATGGAATGC |
| 475 | TCCACTCCATTCCAT | 175 | TTCCATTTGATGA |
| 464 | AAATGGACTCGAATGGAATCATC | 172 | TTTTTTCT |
| 454 | GGT | 171 | AATGGAATCATCATCAAATGGAATTG |
| 447 | TTCA | 169 | TCATT |
| 444 | TCAATGATTCCATTCGATTCCAT | 169 | AAGAAAA |
| 425 | CTCC | 168 | AAATATGAAAGTACATC |
| 418 | CAATGATTCCATT | 166 | GGAATGGAATTGAAT |
| 413 | TTTTTTG | 164 | ATGGAATGGAATAGA |
| 409 | CCATTCAATGATTCCATTCGAGT | 161 | ATCGAATGGAAT |
| 408 | TCC | 158 | TTTATTT |
| 408 | TAT | 156 | CTTGCTTT |
| 384 | AACCCCC | 154 | GAGTCCATTCTATTT |
| 376 | TGCAATGGAATGGAA | 151 | GGAATTATCGAATGCAATCGAAT |
| 359 | AGAC | 150 | AATGAAATGGAATCG |
| 346 | GAATGGAATCATCATTGAATGGAATC | 150 | AAAAAAAGAAA |
| 341 | TCCATTCCACTCCAC | 149 | TTCGAGTCCATTAGATGATTCCA |
| 329 | GTGA | 148 | CGTCAGCATA |
| 316 | AAAAAG | 148 | TTTCC |

| 144 | TAAAAAAA | 80 | GTGCT |
| --- | --- | --- | --- |
| 144 | CCATTCAATGATTCCATTCAAGT | 80 | CACCATCAT |
| 142 | TGAAATGA | 78 | GAGATGAAATGAT |
| 142 | GTATT | 78 | GGGGAAAGGAGTG |
| 141 | CAGCAA | 78 | ATCATTGAATGGAATAGAATGGAATC |
| 140 | CCCTC | 77 | GAATCGAATGGAATACATCGAAT |
| 138 | AGAGAG | 77 | TCATCATCAAATGGAATCAAATGGAA |
| 136 | TCCATTCAAGTACAT | 77 | CCCCAAAAC |
| 132 | GAATCATCATCGAATGGAAATGAATG | 77 | ATTCCACTCCACTCCATTCC |
| 130 | GGAATGGAATGGAATGGAAA | 77 | CGATATGATTCCATTCGATTCCATT |
| 128 | TCGAATGGAATCATCATCGAATGGAG | 75 | GAATGGAATCGAATA |
| 127 | GTCCATTCTATTCCC | 75 | CCAAA |
| 127 | ATGGAATGGAAACGA | 75 | GGGAGGAA |
| 124 | GTCGTAA | 75 | TCGATTCCATTTGATGATGATTCCA |
| 122 | CATTCAGATGATTCCATTCGATTC | 75 | CAGCAG |
| 121 | TCTCTCTG | 75 | CCATTCGATTCCATTCGAGGATAATT |
| 121 | TCG | 74 | AAGC |
| 121 | CATTCCATTCGACTC | 73 | TTTTTTTGTTT |
| 119 | ATGAAAAAACAGAACA | 73 | CATTCGAGTCCATTCGATGATATTC |
| 117 | GAATGGACTCAAAT | 72 | ATCATTC |
| 114 | ACCAT | 71 | ATGATTCCATTCGAATCCATTCGATG |
| 112 | GGGAA | 70 | GAATGGCATCAAATG |
| 110 | CCATTCCACTCCAGT | 70 | TGCAATCGAATGGAATCATCGAA |
| 110 | TCCATTCTGTTCCAT | 69 | TTCTA |
| 110 | AATGGAATCGAATA | 68 | TGTG |
| 109 | GGAATCATCGAATGGAACCGAAT | 68 | AATGGAATCACCATCGAATTGAAACG |
| 108 | TGCTGCTCC | 67 | ATTCCATTCCATTCATTGATG |
| 107 | AATGGAATGTACTCG | 66 | TCTCCT |
| 107 | AATGGACTCG | 66 | GATAATTCCATTCGATTCCATTCGAT |
| 105 | CCATTCAATGATTCCATTCCAGT | 66 | TCCTC |
| 104 | AAAAAAAAAC | 65 | ATGATTCCATTCAAGTCCATTCG |
| 103 | AAGGAGTGGGGGGA | 65 | TGGTGATGG |
| 102 | ATGGAATCATCGAATGGACTGGA | 65 | CTCCTTCC |
| 100 | GATTCCATTCGAGTCCATTCGATGAT | 64 | ATGGACTCAAAGAGA |
| 99 | TGATTCCATTCGATTCCATTCGATAA | 64 | TCATTTCATCATT |
| 98 | GAATCATCAATGGACTCGAATG | 64 | CCTAATAAC |
| 96 | CGTTCCATTCCATTC | 64 | TGTTGTTGC |
| 95 | CTTCT | 64 | ATCATCGAATGGAATAGAATGGAATC |
| 95 | ATTCCATTCA | 64 | TCCATTCCATTCCATACCATTGCTC |
| 93 | CCAA | 63 | AATGGAATCATCATCGAATGGAACCG |
| 92 | CGATAATTCCATTCGATTCCATT | 63 | GAGAGACAGAGA |
| 92 | GGGGTT | 63 | ACAGACAC |
| 91 | GGAATCATCATCAAATGGAAACGAAT | 63 | GGAAGGGAATCGAAC |
| 90 | GAATGGACTCAAATG | 63 | GAATAGAATGGACTC |
| 90 | TTCCATTCTCATTCGA | 62 | AGCTACTAGTGTATGAGGCCTTA |
| 90 | AAAAAAACA | 62 | AATGGACTTGAAAGGAATCATCA |
| 89 | AATGGAATGGAATGA | 62 | ACACA |
| 88 | TCGAATGGAATCATCTCAATGGAA | 62 | ACTCGAATGGAATCATCATTGA |
| 88 | CATTCCACTCGATTC | 62 | TTTTTTATT |
| 88 | AAGGAGTGGGGG | 62 | CTCTTTCT |
| 88 | ATGGAATCGAATGGAATCATCATCA | 62 | GATAATTCCATTCGATTCCATTTGAG |
| 87 | GAATGGAATGGAAC | 61 | ATACACAC |
| 86 | GATGATTCCATTTGAGTCCATTC | 61 | ATGATGATTCCATTCGATTCCATCCA |
| 85 | GATTCTATTCCATTA | 61 | AATGGAATCATCAGAATGGAATCG |
| 85 | TTTTCTTTTTTT | 60 | ATTCCATTCAATTCCATTTCATTGATG |
| 83 | TTCCATTTGATGACGATTCCATTCGAA | 60 | GTGTG |
| 83 | ATCATCGAATGGAATTGAATGGA | 59 | TTTTTTGTTTTT |
| 82 | ATTCCATTCGATTCCATTCCATGATG | 59 | ATGGAATTGAATGGAATCATTGA |
| 81 | GAATAGAATG | 59 | TGTGTA |

| 58 | GATGATTCCATTCCATTCCATTC | 44 | ATGATGATTCCTTTCGATTCCATTCG |
| --- | --- | --- | --- |
| 58 | GATGATTCCATTCGATTCCATACGAT | 44 | AAAGAAAGAG |
| 58 | AATGGAATGT | 43 | TCCGT |
| 57 | CTTTCTTTT | 43 | GAGAAAGAGAGA |
| 57 | GATTCCATTTCATTCCATTCGATGAT | 43 | TTCCAATCCA |
| 57 | AGTCCATTACTATCC | 43 | AAAAAGAAAG |
| 57 | TCTTCA | 43 | TCCATTCCTTTCCAC |
| 57 | TCCACTCCAC | 43 | TCCATTACGATGATTCCATTCGAT |
| 56 | CACATACACA | 42 | AAAAACAAAAC |
| 56 | TCTCTT | 42 | GATAGAT |
| 56 | GCG | 42 | GGAATCGAATGGAATCATCATCGGAT |
| 56 | TCGAATGGAATATCATCAAATGGAA | 42 | ACCCATCC |
| 56 | CATAG | 42 | GCTGGCTGGCTTG |
| 55 | GTGTGT | 42 | ATGGACTGGATAGAA |
| 54 | TTTCCCTTTCACT | 41 | TTTCTTTTTTCT |
| 53 | GAATCATCATAGAATGGAATC | 41 | AATCTG |
| 53 | GAATGGAATGGAGAGCAATGGAATA | 40 | TCATC |
| 53 | CACACACGCA | 40 | CGGCTC |
| 53 | GATGATTCCATTAGATTCCATTC | 40 | CTGGGG |
| 53 | TGGAATGGCATCGAA | 40 | CCATTCGATGATTCCATTTGATC |
| 53 | GGAATTGAATGGAATCATCATTGAAT | 40 | ATTCCATTCGATTCTATGCGATG |
| 52 | CATCATCAAATGGAAATGAATGGAAT | 39 | CATCGAATGGAGTTGAATGGAATCAT |
| 52 | ACAACG | 39 | TGCAGTAGTGCTCTGTGGTGGTGTTTGGTGTAGT |
| 52 | GAAGGAAA | 39 | TCATCATAAAATGGAATCGAATGGAA |
| 51 | GGTGG | 39 | TAAAT |
| 51 | TTCCATTCAATGAATCCATTCGA | 39 | GATTCCATTCGATCTCATTCGAT |
| 51 | ATGATTCCATTCGAATCCATTCCATG | 39 | GGGGAG |
| 50 | TCTTCC | 38 | CACACCACA |
| 50 | GTCA | 38 | TGGGA |
| 50 | ATTCCATTCGAGCC | 38 | ATCACCGAATGGAATCAAATGAA |
| 50 | ATGATTCCATTGGATTCCATTCAATG | 38 | TATACA |
| 50 | GTCTGT | 38 | TGGAATCGAATGGAATCATCAATGAA |
| 49 | TATCATCGAATGGAATCGAATGA | 37 | TGTGA |
| 49 | CATCGAATGGAATCGAATGGAATCAC | 37 | CTCAAGAGTTGCAAGCTGTTAAGTTTGA |
| 49 | GAATGGAATAGAATGGAATCATC | 37 | TGGAATCATTGAATGGAATAGAA |
| 49 | AATGGAATAGTAATCC | 37 | GCCACC |
| 49 | AATT | 37 | TCATTGAATGGAATCAAATGGAA |
| 49 | ATGATTCCATTAGAGTCCATTCA | 37 | TCCATTTGATGATTCCATTTGAT |
| 48 | AGAGAGAGACAGAC | 36 | AAAAACAAACAA |
| 48 | TCCATTCCATTCCATTCCATTGATC | 36 | CCGACC |
| 48 | GATGATTCCATTCAAATCCATTCGAT | 36 | ATGGAATGAATGGAATCATCATCAA |
| 47 | ACTGA | 36 | TCCATTCCGT |
| 47 | CGCA | 36 | TATATATGTG |
| 47 | CCTTG | 36 | GGGCAG |
| 47 | ATCATCACCACC | 35 | ATTTTTTTTT |
| 47 | ATTAAA | 35 | CCATTCGAGTCCATTGGATGATT |
| 47 | ATGGAATGAA | 35 | TTTCTTCTCCTTTTTCTA |
| 47 | GATTCCATTCAATTCCATTAGATGAT | 35 | TAGAGA |
| 46 | CATTCGATGATTCCATTCGAATC | 35 | AATAGAAAGAATTGAATGG |
| 46 | CACACACATG | 35 | TGGTC |
| 46 | AGAGACAGAC | 35 | GGAATGTAATGGAAT |
| 46 | CCTA | 35 | ACAGAGAGAGAGAGAGACAG |
| 46 | TGGAAAGGAA | 34 | AACTTCA |
| 45 | TCAATTCCATTCGAT | 34 | GATGATTCCATTCAAGTCCATTT |
| 45 | ATATAG | 34 | AATCATCATCAAATGGAATCTAATGG |
| 45 | TATTCCATTAGATTAC | 34 | GGTGGT |
| 44 | CAGCAC | 34 | TGATGATTCCATCTGATTCCATT |
| 44 | CTGGA | 34 | CATTCAATGATGATTCATTTCGATTG |
| 44 | AAAGAAAGAAA | 34 | ATTCCATTACACTCCATTCC |

| 34 | CAAACAAAA | 27 | CTCTGTAAAATGGACCAATCAGCT |
| --- | --- | --- | --- |
| 33 | AAAAAAAATAA | 27 | ATTCGATTCCATTCTATGACGATTCC |
| 33 | GCATACACTAGTAGCTTAAGGCC | 26 | CGATTCCATTCGATGATGATTCAATT |
| 33 | TTCCATTCGATCCA | 26 | GAATGAACTCGAATGGAAACATC |
| 33 | GGAATGGAATGGAATGGAGT | 26 | CCTCGGCTGCTGCAGCTACTCGTTTTG |
| 33 | TCTCTGTCTT | 26 | CCCTCCCC |
| 33 | AATCATCGAATGGAATCGAATGGAAC | 26 | TCCATTCGAATCCATTCCACGATGAT |
| 32 | TGATTCCATTCGATTCCATTCAG | 26 | ATTCGATGATGATTCCATTCGGATCC |
| 32 | GATGATTCCATTAGAGTCCATTC | 26 | AATGAAG |
| 31 | CCATTCATTCCATTCGATGATGATT | 26 | GATGATTCCATTCGAGTCCATTTGAT |
| 31 | GAATCGAATGGAATC | 26 | GATGATTCCATTCGATTCCATTTCAT |
| 31 | TTTCCATTCCACTCC | 26 | AATGGACTCGAATGGGATCATCATCA |
| 31 | GATAATTCCATTCGATTCCATTGAG | 25 | GATTGCG |
| 31 | TTCGATGATACCATTCAATTCCA | 25 | ATTCGAGTCCTTTCC |
| 30 | GGAATCAAATGAAATCATCGAAT | 25 | TGATTCCATTCGATTCCATTCGACAA |
| 30 | TGAGAG | 25 | TCATTTCATC |
| 30 | ATATC | 25 | AATCATCGAACGGAATCGAATGGAAT |
| 30 | GAATGGAATCCAATG | 25 | TCACGACCGT |
| 30 | TTCGATTCCATATGAAGATGATTCCA | 25 | AGAAGAA |
| 30 | CTGCTCCTGCTGCTG | 25 | ATGGAATCGAATGGAATGGA |
| 30 | TTTTTTCTTTTTT | 25 | TTCTATTCCATTCCATTCT |
| 30 | CCATTCCACTCCACTCCACT | 25 | CCAGAG |
| 30 | TGATGATTCCATTCAATTCCATTCA | 25 | CCCCCCCCA |
| 30 | TTCCATTCGAGTCCATTCGATGATTG | 25 | AAAAACAAAC |
| 30 | CACACAC | 25 | CATTCGAATCCATTTGATGATTC |
| 30 | ATTCCATTCGTTTCAATTCGATGGTA | 25 | TGATGA |
| 30 | CCATTCGATTCAATTCGATGATGATT | 25 | TCCATTCGTGATGATTCCATTCGAT |
| 30 | TGGGGG | 25 | TGATTCCATTCGATTCCATTCGAGA |
| 29 | TTTTTTAA | 25 | TTTATTTAT |
| 29 | CCTCTCTC | 25 | ATACATAT |
| 29 | TGATTCCATTCGATTCAATTCATTGG | 25 | AAAAAAAAGAAAG |
| 29 | GGCTG | 25 | AATGGAATGAAATGGAATGG |
| 29 | CACACT | 25 | ATGGAATAGAATCGA |
| 29 | TTTTTGTTTGT | 24 | AAAAAAAAAAAAAG |
| 29 | ATTGA | 24 | TATTAAGGCTC |
| 29 | ACAACA | 24 | GGTGTTAG |
| 29 | TATTTGATGTAATTTCATATA | 24 | CCACCCCCCCC |
| 29 | CACACACATACA | 24 | AAGGAATCATCATCAAATGGAATCGA |
| 29 | CATTCGAGTCCATTCGATGATTATTC | 24 | GGATGATTCCATTCGATTCCATTCGA |
| 29 | CTCCCCCCCC | 24 | AATGGAATCGAATGGAATACATCG |
| 29 | GCCGTA | 24 | AGACAGACAGAGAAAG |
| 28 | GGTGGTGTTTGGTGTAGTTGTAGTAGTGCTCTGT | 24 | CCTGCTGCC |
| 28 | CCTCCCCCC | 24 | ATTCGAATCAATTCCATGATGTTTCC |
| 28 | GAATGGAATCGAAAG | 24 | TTCCATTCCATTCCACTGCATTCCG |
| 28 | CAAACCCCAAACCCC | 24 | AGGGGAG |
| 28 | GAATGGAATCATCGAATGGACAC | 24 | CACGCA |
| 28 | TCGAATGGAATCATCG | 24 | AAAAAAATAC |
| 28 | AATGGAATCGAATGGAATCATC | 24 | GGAATCGAATGAATCATCATCGAAT |
| 28 | TCGTGG | 24 | TGACCC |
| 28 | CCCAAACCCCAAAC | 24 | AAAGTGGTCCAAAATGGGGGTCAA |
| 28 | GAATCATCATGAATGGAATCGAATA | 24 | GATGATTCCATTCGATTCCATTCAA |
| 28 | CGAATGGAATCATCATCGAATAGAAT | 24 | AAAAAAACAAAAA |
| 27 | GTCCTCATCAGCACCGC | 24 | TTCCTTCC |
| 27 | ATATACATAT | 23 | AATGGAATCGAATGGATATCATCG |
| 27 | TCCATTCCATTCAAG | 23 | GCCTCCCA |
| 27 | CATTCGATTCCATCTATGATGATTC | 23 | CCGTCG |
| 27 | TCAAATGAATCGAATGGAATCATCA | 23 | TATACACATATA |
| 27 | GAATGGAATCGAATGGAATCATCAAC | 23 | GAATGGAATCATCATCAAATGGAACC |
| 27 | CGAATGGAATCATCATTGAAT | 23 | CTTCTCCTC |

| 23 | ACACCC | 20 | TCGAATGGAATCACATCGAATGGAA |
| --- | --- | --- | --- |
| 23 | CACTA | 20 | ATCTCAA |
| 23 | CAAGAC | 20 | GATGATTCCATTCGATTCCATATAAT |
| 23 | GATGATACCATTCGATTCCATTC | 20 | GAATGGAATCAATG |
| 23 | GGGGAAGGAGTGG | 20 | CTTCTTCTA |
| 23 | GATAC | 20 | CCATTCAGTTCCATT |
| 23 | CCAAAAAATG | 20 | ATCAACATCGAATGGAAACGAATGGA |
| 23 | TTCTG | 20 | CCCCCAC |
| 23 | CACACACACCA | 20 | GAATGGAATCGAATT |
| 23 | CACCGA | 20 | AATCATCATCGAATGCAATCGAAAGG |
| 23 | CGTGAACGGG | 20 | TCCATTCCATTCGTG |
| 23 | GAGGAGGAA | 20 | GTGTGTGTT |
| 23 | TCATTCAC | 20 | TGATTCCATTCGAGTCCATTCGATAA |
| 23 | CCCCCCACCC | 20 | GAATGGAATCATCATCGAATGAGATC |
| 23 | TCGAATGGAATCGAATGGAATCATAA | 20 | TGATTCCATGCGATTCCATTTGATGA |
| 22 | GATGATTCCATTCCAGTCCATTA | 20 | TCCATTCGATGATGTTTCCATTCAAC |
| 22 | GAATTATCGAATGGACTTGAATG | 20 | AATGGAATCGAAAGA |
| 22 | CATCCAGACAGA | 20 | TCTGCT |
| 22 | CCCAAACCCCCAAC | 20 | TCATAGAATGGAATCGAATGGAA |
| 22 | TTTCTCTCTC | 19 | GTTTGTGT |
| 22 | AATCGAATGCAATGG | 19 | GAATGGAATGTACTA |
| 22 | TTCCATTCGATGATGATTCCATACGA | 19 | GATGATACCATTCAAGTCCATTC |
| 22 | CATGCATC | 19 | CGATGATTCCATTCGATTCCATTCAA |
| 22 | TATATATATGTGTG | 19 | AAAAAAGTCA |
| 22 | TTCCATTCGATTCCATTCCAATCCC | 19 | GTTGCC |
| 22 | AATTGAAACGAATGGAATCATCATGA | 19 | TTCCATTCGATTCCATTCGGTGA |
| 22 | CATTGGTTCCATTCTATGATGATTA | 19 | AACACACACACA |
| 22 | AATGGAATCAACCCAAGTGC | 19 | AATGGAATCGAATGGAATCATCATT |
| 22 | AATGGAATCATCTCGAATGGAATCA | 19 | CCCCCCCCCG |
| 22 | TGGAATGTACATGAATGGAA | 19 | TAGC |
| 22 | CACATATACA | 19 | CGAATGGAATGCAAT |
| 22 | GAATTGAATGGAATCATCAAATG | 19 | TGGAATCTCGAA |
| 22 | TGATTCCATTTGAGTCCATTCAA | 19 | GGAAGG |
| 22 | ATTCCATTCCATTGCATTCC | 19 | CCCAATC |
| 22 | GAATGAGATCGAATGGAAACATC | 19 | CCTCCCCCCCCC |
| 21 | TTTTATTTAT | 19 | GGAATCATCATCGAATGGAATCAAAG |
| 21 | GGTGTGTG | 19 | CCCGAT |
| 21 | TTCCGTTCGATTCCA | 19 | GAATGGAATCAAAGGAAATCATC |
| 21 | GCACACACACA | 19 | TATCATCGAATGGAATCGAATAGA |
| 21 | CCATTCCATTTGACT | 19 | CACTCATTCACT |
| 21 | TTTTTTGTTTTGT | 19 | CCATTCCTTTCGAGT |
| 21 | AATGGAATGGAATGG | 18 | ATCACCTCC |
| 21 | GAATGGAATGGAATGGAAACAACCC | 18 | AGACAGAAAG |
| 21 | TATATACACACA | 18 | TTCCATTCGATTTCA |
| 21 | GGTG | 18 | GAATCATCATCGAATGGAAATGAAAG |
| 21 | TTGTTTAAAA | 18 | CACACACACT |
| 21 | AAACGGACCAATCAGCACTCTGTA | 18 | GACAGAAAGAAAGAGAGA |
| 21 | CCCCTCC | 18 | GGGGC |
| 21 | ATTCCATTCGATTCAATTCGATGGTG | 18 | ATTCGATTCAATTCCATGATGATTCC |
| 21 | GATTCCATTTATGATGATTCCATTC | 18 | AATGGAATGGAATAGAATGG |
| 21 | CGAATGGTATCATTGAATGGAAT | 18 | ACATT |
| 21 | GCAGAG | 18 | GATTTTT |
| 21 | CCCGTTCACGACCGCTCACG | 18 | ATGGAATCATCACCGAATGGATTCCA |
| 21 | ATTACATTCCAGTTCATTAC | 18 | CCCTCCCCCCC |
| 20 | TGGCCC | 18 | ATTACAA |
| 20 | GGACAGGACAAGAACTAAGCT | 18 | ATTCCATTCAATGATGATTCA |
| 20 | TTCCATTTAATTCCA | 18 | CTCTCTTTCTGTCTTT |
| 20 | CCCTCTCTCT | 18 | AAAACAG |
| 20 | AATGGAATCATCGAATGGAACG | 18 | AATGCAGTGGAATGGAATGGAATGG |

| 18 | AATGGAATCGAATGGAAACATCATGG | 15 | ATGATTCCATTCGATTCCATTCTA |
| --- | --- | --- | --- |
| 18 | ATCATCGAATGGAACCAAATGGA | 15 | AATGGACTCGAATGGATCATTG |
| 17 | GATTTTG | 15 | GGTACG |
| 17 | GATGATTCCATTCGATTCCAATCGAT | 15 | GAAGAAGATGATGAT |
| 17 | ATTTGATGATTCCATTTGATTC | 15 | AAATGGACCAATCAGCACTCTGTA |
| 17 | GATTCCATTCGATGATTTCCATTT | 15 | AGGAATCGAATGGAA |
| 17 | CAGCAT | 15 | GAAATGAAATGATGATGAAATGAT |
| 17 | GAATGGAATGGACTA | 15 | CATTCATGATGATTCCATTCGTTTC |
| 17 | CATGCCATGCCACGGCCTCGGCCCG | 15 | CTGTGTG |
| 17 | TTTTTTTTTTTA | 15 | GAATGAGACTCGAATGGAATCATC |
| 17 | CCCACCCC | 15 | GTGACTA |
| 17 | ATACATAC | 15 | CCAGATCACACTGTGCCACACTCT |
| 17 | TTGAATTTTATTA | 15 | TTGTGC |
| 17 | AACTCAT | 15 | GATGATATCCATTCAAGTCCATTC |
| 17 | GTCCATTCGAGGATTCCATTCGA | 15 | AAGAAAAGAAGG |
| 17 | GAGGAGGAGAGAGAAG | 15 | TGTGTAGTTTGTG |
| 17 | AAAAACAAAAAC | 15 | CCCTGCCCTGG |
| 17 | TGAGCC | 15 | TTCCCCCACTCCTT |
| 17 | TTCCATTCGTTCCATTCGATGATGA | 15 | CCTTACA |
| 17 | CTCCAATCCA | 15 | GATGCGGTGCT |
| 17 | CATAA | 15 | GAATCATCATAGAATGGAATCAATG |
| 17 | TCGAATGAAATCATCGAATGGAA | 15 | AATGGAATCACCATCG |
| 17 | GGAATCGAATGGAATCATCATCGAAC | 15 | ACGCCG |
| 17 | CACACACACATG | 15 | AATAATCATTTG |
| 17 | ATGGATCATCATCGAATGGAATCGA | 15 | GAATGCAATGGAATGGAAACAACCC |
| 17 | TCTGTCTCTTTCTCTC | 15 | CACACATTCA |
| 17 | TCACTCAC | 15 | TCACACAC |
| 16 | ACTCGAATGGAATCATCATCAAATGA | 15 | GAATATGTTAT |
| 16 | CTCACGACCG | 15 | TAGAG |
| 16 | CATCGAATGAACTTGAATGGAAG | 15 | TTTCTTA |
| 16 | GATGATTCCATTCGATCCATTCGAC | 15 | TGCTGTTGCTGTTGCTGCAGC |
| 16 | AGTGGAATGGAGTGA | 15 | GTACGAA |
| 16 | ATGATGGTGATG | 15 | TGGATGGGTGGG |
| 16 | CAAAAAGGGG | 15 | TGATTCCATTAGATTCCATTCGAGA |
| 16 | ACGAGTG | 14 | TCCATTCGATGATTCCATTCAGT |
| 16 | GAATGGAAGGTAATC | 14 | TGATTCCATTCATTTCCATTCTG |
| 16 | CACACAGGCACTCA | 14 | GCATATACATACCCACACAT |
| 16 | TCCATTCGATTATTCCATTCGAT | 14 | TGTTCTGTTTTTTCCA |
| 16 | GAATCATCATAGAATGGAATGAATG | 14 | GAATGGAATGCAAAG |
| 16 | TGAATGGAATCAAATGGAATCATCA | 14 | ATGATTACATTCGGTTCCATTCAATG |
| 16 | ACTGAGC | 14 | CACGCACA |
| 16 | CACCCATCGTG | 14 | CCCCCCCCCACC |
| 16 | CCCAACCCCCAAACCCCAAACC | 14 | AATCATTGAATGGAATCGAATGGAAT |
| 16 | ATTCGATGTTGATTCCATTCGATTCC | 14 | TGATGATTCCACTTCGATTCCATTCA |
| 16 | AATGGAATCATTGAATGGACTCC | 14 | GATGACTCCATTCGAGTCCATTA |
| 16 | GAATGGAATGGAATCATCAAATG | 14 | TTCTTCCTC |
| 16 | GGAATGCAAGGGAATGGAATCAAC | 14 | GTGTATGTATGT |
| 16 | TCCATTTCATTCGAA | 14 | CACACATG |
| 16 | AAACCAAAACCAAAAC | 14 | ACACACACAGTCAC |
| 16 | TTACTTG | 14 | GAGAAAGAGAGACAGA |
| 16 | AATCACAT | 14 | TTCTTTTTTTC |
| 16 | TATAACCTG | 14 | GGGGTA |
| 16 | GAATCACATCGAATAGAATCGAATT | 14 | TGTGGTA |
| 16 | ATGAATCGAATGGAATCATCTA | 14 | GGAGTGGAGTGGAAAGGAAT |
| 16 | CATTCGATTCCATTCGGATGATTC | 14 | ATTCGATTCCATTCGATGATGATTCG |
| 16 | CTTCTTCTCCTC | 14 | CAACAT |
| 16 | AATGATTCCATTCGATTCCATTTGA | 14 | AAGGAAGGG |
| 16 | CGAATGGAATCATCTCAAATGGAAT | 14 | CATTCCATTCATTACATTC |
| 15 | AAAAAAAAAAAAAAC | 14 | TGGAAATGGAA |

| 14 | CGCACACACACA | 13 | AATGGAATCGAATGGAATCATATCA |
| --- | --- | --- | --- |
| 14 | GTGTGTGGAT | 13 | AATGGAATCAAAGGGAATCATCG |
| 14 | AAACCA | 13 | GTAGTAGTC |
| 14 | TGATGATTGCATTCGATTCCATT | 13 | CGAATGGAATCGAATGAAATCATCAT |
| 14 | TAATTG | 13 | ACCTTAT |
| 14 | GTGGAG | 13 | CATCCACT |
| 14 | AGTAT | 13 | GCGA |
| 14 | ACTGCTTCTGCAACA | 13 | TATGGC |
| 14 | TTTATTTTAT | 13 | AGGGATTGGGGCGCAGAAATA |
| 14 | AAAAGAAAGAAAG | 13 | GGGCGAAGGCCACCTTT |
| 14 | TCTGTCTCTCTCTC | 13 | ATCAGCGAATGGAATCGAATGGAATC |
| 14 | AAGGCCGGTGGGGGGGGAGGGTGG | 13 | AATCATCGAATGGAATCGAATGGAA |
| 14 | AAACACACAG | 13 | AAAATAAAAAATA |
| 14 | CTGGACCAA | 13 | GGAATGGACTGGAGAT |
| 14 | ACAGCAGCAACA | 13 | CATCCATT |
| 14 | CCTCCCTTCCTT | 13 | AGTTCTGTCAAGTGACTGTCTCGGG |
| 14 | CCCAGATGTTAGCAACCAGCTCCATGGACT | 13 | GGAATCATCATCAAATGGAAATGAAA |
| 14 | GTACT | 13 | TGCACGCGATTCGCC |
| 14 | GAGCTG | 13 | GGATA |
| 14 | AAAAGAAAAAG | 13 | TCACC |
| 14 | ACACACATACACAACAT | 13 | GCAGGTGGCGGT |
| 13 | CAAACGAATACGTCAATAAAT | 13 | CGATGATATCATTCAATTCCATT |
| 13 | CCATTCCATTCAGT | 13 | AGAATGTACCCATCGTGC |
| 13 | AGGGGACAGTG | 13 | GAAGTAA |
| 13 | AACACACACACT | 13 | CAGGCAG |
| 13 | GCCCCCCCC | 13 | GGATGGATGGGT |
| 13 | CGTTTCATT | 13 | TCCATTCGATGATTCCATTTCAT |
| 13 | TTTTTGTTGTTG | 13 | AAAAAAGTCGCGAACCGCG |
| 13 | ATTCGAGTCCATTTGATGATTTG | 13 | TGAATGGAATCGAA |
| 13 | TTCCATACAATGATGATTCCATTCGA | 13 | GGAATCATCGAATGGAATGAAT |
| 13 | CTGTCTCGGGAGTTCTGTCCAGTGA | 13 | TAAAACTA |
| 13 | AAACCCCAAACCCCA | 12 | GCAGCACTCACCAGTACCA |
| 13 | TTCCATTCATGA | 12 | GATGATTCCATTTGATTCCATTCAGT |
| 13 | CTATACC | 12 | ATCATCGAATGGACTCGAATGAA |
| 13 | TCCTGT | 12 | CATCTATCCATT |
| 13 | ATTCCATTCGATGATGATTCCATGCA | 12 | GAATCGAATGGAAACATCGAAT |
| 13 | ATTCAATGATGATTTCATTCGATTCC | 12 | GGAATCATCAAATGGAATCGAAA |
| 13 | GATGACTCCATTCATTTCCATTCGAT | 12 | TTCCATTCGATGATTCCCTTGA |
| 13 | AAAACTC | 12 | GATGTAC |
| 13 | ACACACACACCCCACC | 12 | GAATGCAATGGAATGGAATCAACTC |
| 13 | TGATTCCATTCGATTCCATTTGATG | 12 | AAACCAAAA |
| 13 | CACTCACAC | 12 | ATTCCATTCGAAGATGATTCCATTCA |
| 13 | CGGAATGA | 12 | TTCCATTCCAATCGAGTCCATTCCA |
| 13 | GCTAG | 12 | CCTCCTA |
| 13 | TGATTCCATTCAATTCATTTGATGA | 12 | AAATTTCGTC |
| 13 | CCCTGGGCTCACAGCCTCGCGCT | 12 | GCGTGCGTGTGT |
| 13 | TCAAATGGAATCGAATGGAATAATCC | 12 | AATGGAATCGAATGAAATCATTACG |
| 13 | CAGCAGGT | 12 | TTCGAGGATTCCATTCGAGTCCAT |
| 13 | ATATGCATATATATGCAT | 12 | AAAAAATTAC |
| 13 | CCGTCGGAAGC | 12 | ATTCAATGAGGATTCCATTCGATTCC |
| 13 | TCTCTCTCTCTGTCTG | 12 | TCCATTCGATGATGATTCCAATCAA |
| 13 | AAAAAACC | 12 | GCTGGAGT |
| 13 | TATCTAGCTCTCAAAA | 12 | ATGATTATTCCATTCGAGTCCATTCA |
| 13 | ATTCCAATCGATTCC | 12 | GCAGAGATTAG |
| 13 | GTCCTTCTTCTC | 12 | ATGTATATTTAGACCGGAGT |
| 13 | ATCGAATGGAATTGAATGGAATT | 12 | ACTTTGCCCCAGCC |
| 13 | TTGAGTCCATTCCCT | 12 | ATTCCATTCGTGATGATTCCATTCA |
| 13 | GATGTCCACCTGAGTCCT | 12 | TCGCC |
| 13 | GGGAGCCTGTCCCGGTCCACTC | 12 | TTGTGG |

| 12 | AGAGACAC | 12 | ATGATTCCATTCAAGTCCATTCTATG |
| --- | --- | --- | --- |
| 12 | GGGGTCTGGTAGACACGGATGCCACAT | 12 | CATATG |
| 12 | CCCTGTGA | 12 | TACTAGTGTATGAGGCCTTAAGG |
| 12 | CGGTGACCG | 12 | TGATGTTTCCATTTGATTCCATTCAA |
| 12 | GGGGAAAGAGTGG | 12 | AAATGGAATGGAATGGAATGGACTC |
| 12 | CCATTCGATTCCATTCGATGATAGTT | 12 | TCCACTCCACTCCATTACAC |
| 12 | GATTCCATTTGATGAT | 12 | TCCATTCGATGATGTTTCCATTCGAC |
| 12 | ATCATCAAATGGAATCGAATGAATC | 12 | AATGGAATCAAATGGAATCAATGA |
| 12 | TTAATTGACACATAG | 12 | CCCCCGCC |
| 12 | GCTGAAATCAATTGATTGATTTTCTTGA | 12 | GGAATCATCGAATGGAAGCGAAT |
| 12 | GATGATTCCTTTCGATTCCATTCCAT | 12 | TTCCTGATG |
| 12 | TGGAGTATAATGGAATAGAA | 12 | ATTTTCAGCGTTTTGTACAAACTTTTG |
| 12 | ATATGGAAAAGATTCCATGT | 12 | CTGGCTCCCGCCTGTCACGTAGATATG |
| 12 | TGATGATTCCATTAGATTCCATTCGA | 12 | TTGATGATGGTTATTTTTGATTCCAT |
| 12 | GGGCTGGCGGGCAGG | 12 | ATCATCAAATGAACTCGAATGGA |
| 12 | GAGAGAGACAGAGACA | 11 | GTTGCTTCGGCT |
| 12 | TGATGATTCCATTCGATTCATTCA | 11 | AAGCGTTACGTAGTAACAAT |
| 12 | TCGAATGGAAC | 11 | AATGGAATCATCAAGATGGAATCA |
| 12 | ACACATATACACACAC | 11 | ATCAAATGGAAATCATCGAATGGG |
| 12 | ATCATCGAATGGAATCGAAGTGGA | 11 | TCGT |
| 12 | AATGGAATCGAACGGAATCATCATCA | 11 | CCCACCCACACCAGTGAGGGTCAGCCACAG |
| 12 | CATGGCCCTGTGTGCTCATC | 11 | TGTGGTGTGTGTATGTGGA |
| 12 | CGGTCTCTGACACATCCT | 11 | TGATGATTCCATTCGTATCCATTCGA |
| 12 | GGAATCGAATGGAAACATCATCAAAT | 11 | TCTGTCTCTCTG |
| 12 | AATGGAATCATCGAATGAGTTG | 11 | TTACCC |
| 12 | CAACAGAATATAAAACACGCCACCCAA | 11 | TTCTTCCCCTTCTT |
| 12 | GGACTCGAATGGAATCATCCAT | 11 | GAATGGAATCATCATCAAATGAAATC |
| 12 | CTTCCTTG | 11 | GAATGGATTCGATTGGAATCATCATC |
| 12 | TTCTTCTTT | 11 | TATATGTGTGTGTGTGTATA |
| 12 | GGCTTCTCGAGGCTCCGGCGGCTCAGCTCACT | 11 | AGATCTT |
| 12 | AATTACG | 11 | GCTGGGTTCATGCG |
| 12 | CTGGGTCCAGGATTCTGGTTCTGAG | 11 | CCACAG |
| 12 | TTCATATTCTTTAGC | 11 | CATCTA |
| 12 | TCTCTCTCTTTCTCTGTCTG | 11 | CTGATTGGTGCATTTTACAGAGCA |
| 12 | CACCCAG | 11 | TTCTCTCTGTCTG |
| 12 | AATTTAAATTAA | 11 | TTCCATTCCAATCCA |
| 12 | TTCCATTCTATTCCTTTCCATACGACTCAA | 11 | TCTGTCTTTCTCTG |
| 12 | TCCATTAGATGATGATTCCATGCAAT | 11 | ATTCCATTCGATAATTCCGTTTG |
| 12 | GAATGGACACGAATGGAATCATCATC | 11 | TCTTTA |
| 12 | GATTAT | 11 | AGGTGCTGCGATGGGTGGCT |
| 12 | GGTGTAGACACGGTGGTG | 11 | AATGGACTGGAGTGAAATGGATTCGAATGG |
| 12 | ACACATACAT | 11 | GAATGGAATCATCTAATGGAATG |
| 12 | ATGATTCCATTCGAGTCCAATTG | 11 | TGCAGGGCATGG |
| 12 | TGGAGGG | 11 | TGGAATGGAATGGAATGGAGTAGAG |
| 12 | TTTCTTTTCTTTTCTC | 11 | ATACGTGAGC |
| 12 | AAAGAGAAGA | 11 | GAATCGAATGGAATATCATCGAAT |
| 12 | AATGGAATCTAATGGAATCATCA | 11 | TGTAGTGGAG |
| 12 | AATGGAAACATCACTGAATGGAATCA | 11 | TCCATTTGAGGATTCCATTCGAG |
| 12 | TGGCAGCAGCAACCAGGGCGGATA | 11 | GCTGCTGCGGCT |
| 12 | ATTCCATTCCAGTCCATGATG | 11 | TCCACA |
| 12 | GCAGGA | 11 | GGGGAGGTAGGAGGTG |
| 12 | GGACCGTGGGAACA | 11 | CACGGG |
| 12 | CACCGT | 11 | CCGAGTCACCCCGGGGACAGTGGGCG |
| 12 | CCCCCTCCCCACAGAAGAGTGCT | 11 | AGGAAGGGAGGGAAG |
| 12 | TCGTGTGCGGTG | 11 | AATGGAATCATCATAATGGAATCG |
| 12 | ATCATATATTGTATGTTATAT | 11 | AAAAAAAGAAAG |
| 12 | GTGCGCGA | 11 | AACAAAC |
| 12 | AAAGC | 11 | AGAATGTCGTTCCATCG |
| 12 | CCATTACATTCCATTCCATTCCACA | 11 | ATGATTCCATTCGAGTACATTCG |

| 11 | AGAACA | 11 | CCCCCTCTCTCT |
| --- | --- | --- | --- |
| 11 | CCCCCCCCCCGC | 11 | CAAGGTCT |
| 11 | TGACAAAACAGAACA | 11 | CTAGTAATCAAAAA |
| 11 | CATGGAA | 11 | AATGGACTCGAATGGAAATCATCG |
| 11 | GATGATTCCATTCGATTCCATATGAT | 11 | TCCATTCATGATGATTCCATTCCAT |
| 11 | CATTCTGAGTCCATTC | 11 | CTCTGCGG |
| 11 | CAAACAAACAAAA | 11 | TTTTGCC |
| 11 | AAAAAAAAAATT | 11 | CCTTGG |
| 11 | TCCTAC | 11 | TCAAATGGAAACATCATCGAATGGAA |
| 11 | ATGATGATTCCATTCGATTCCATCC | 11 | GGGCGCTGTGGACGCGGTGA |
| 11 | GACCATCCATAGACCACACT | 11 | AATGGAGTGA |
| 11 | GAGAGAACTAGACACGGCGCCATGT | 11 | ATAAATCAC |
| 11 | AAGAAAGG | 11 | CTCAGTC |
| 11 | TTCCGTTCGAGTCCA | 11 | AAATTCGCGCAGGTA |
| 11 | CAGCATGATCCACCCACCAGAAGCTC | 11 | GTACAGTAGCTGTTGCTGCACC |
| 11 | ACATACACACACAT | 10 | TTCTTC |
| 11 | ATTCCATGCCATTCC | 10 | CCCATCCATCCG |
| 11 | GAATGGAATGGAATTGAACC | 10 | CTGTTTCTCTCTCTCTGT |
| 11 | ATCATCTAATGGAATCAATGGA | 10 | CCTCTCTGGAAGGCATACCAAGAGGCAAAGGCA |
| 11 | CACGCCCCGCCATC | 10 | GAGGGAGGAAGCAAAGGAGG |
| 11 | ACGGTCGCCGAG | 10 | GAAGCAGCA |
| 11 | CGTCATCAT | 10 | GGATGGTCCTCACTCTCTG |
| 11 | ATCTTTCTACATTGC | 10 | CTTCGACTCCGCTCC |
| 11 | GAAATGGAAG | 10 | AGCCAGCCAAGCCACCC |
| 11 | ATGATTCCATTTCGAGTCCATTCG | 10 | GATGAGCACACGGGGCCATG |
| 11 | AATGCAATAGAATGG | 10 | GGGGAAAGGAGT |
| 11 | ATCATGGAATGGAAACGAATGGAATC | 10 | GTGACAGTGCATGTGT |
| 11 | GGAATCGAATGGAATCATCACCGAAT | 10 | CTGACCT |
| 11 | ATGTGTGTAG | 10 | ACACCTGCAGGCACACGCAGCCCTGCCGTCC |
| 11 | AAAAAGAAGA | 10 | ATTCCATTTGATCGTGATTCCATTCG |
| 11 | TTTCAGT | 10 | CTGCAAACCAAGAAGCAGGA |
| 11 | ATATACATACAAATA | 10 | TGCATATTTGACCCGGGCCTCCTGGTGGTGTGG |
| 11 | GCTTGGCTGGCTGGCTG | 10 | CCCGTGAAGC |
| 11 | AACGGAATCCAATGGAATCATAG | 10 | CAGGACAACACCTAAGCTGGA |
| 11 | CCCTGCGGGCCGTGTGCCATGAGGAGGCCAGC | 10 | GAATGGAATCATCATTGAATGGAAAT |
| 11 | ATTCAATGATGATTACATTCGATTCC | 10 | AATGAACTCTTCTAATTCAAACGCACAGGG |
| 11 | TCCTGCAGGTACAGTGCATCGCTTTCTACC | 10 | ACCACACAGCTCACGACAGC |
| 11 | TTCCATTCCAGTCAA | 10 | CTTCGACGA |
| 11 | ATCATCGAACGGAATCGAATGGA | 10 | CATTCGATTCCATTCGATGATGG |
| 11 | TCCTCCATTTTATTGCAAAATG | 10 | TTTTTATTTTCGGC |
| 11 | TGAATA | 10 | GTGTGTGTGTGCACATGGT |
| 11 | CGCCCCC | 10 | TCATCACCATCATCATTA |
| 11 | CGAC | 10 | CTTCTCCTTTCATTC |
| 11 | CTGCATCAC | 10 | ACTTTATAA |
| 11 | GAGTGTCCTCACACCCATCT | 10 | GGAAGGGAGG |
| 11 | GCAGTGGGTCAC | 10 | AGTCCATTTCATTCC |
| 11 | GAATGGAATCATCAAATGGACAC | 10 | AATCGAATGGAATCATCATCAATAG |
| 11 | TGTGTGTGTGTATGTA | 10 | TTCACTC |
| 11 | GTATAGATT | 10 | TGATCCAG |
| 11 | TCTCTCTTTCTT | 10 | GGGTGGTTCCGGTGGAGGGCCCGTTTCT |
| 11 | CACACACGCACGCGCG | 10 | TATGTTAAAA |
| 11 | CTACTACTACAGCTG | 10 | TGGCCGGCTCTCCCCTGCATCTCT |
| 11 | GAATCGAATAGAATCATCATCGAAT | 10 | ATATGTACATATAC |
| 11 | GTGTGAGTGA | 10 | CTCCCGCCCCGGCGCA |
| 11 | GTTTCTT | 10 | AAAAGAAG |
| 11 | TCCTCTCTC | 10 | TGTAAAGACTGTGTCTGAAGCCG |
| 11 | AAATTGG | 10 | ATGGAATCATCATCGAATGAATCGA |
| 11 | CGATGATTCCATTCGAGTCCATTTGA | 10 | AACTTCCT |
| 11 | TTTGATTCCATTCGTGATGATTCCA | 10 | AAACCGCTATAAACCGCCTAG |

| 10 | ATTTCCTCCT | 10 | TAGGGGATCCAGACA |
| --- | --- | --- | --- |
| 10 | CTCATTATATCATT | 10 | GAATCATCATCGAATGCAATCACAAG |
| 10 | AGAGAGGACAGAGAAAG | 10 | TGTCTGAGAA |
| 10 | ACACACATACATGTAC | 10 | CTGGCCTTGCCCTTCC |
| 10 | GAAAGAAGAATTGGCAGAAAATTCTAGAACTTT | 10 | ATTCCATTCAAATCCATTCGATGACT |
| 10 | CACACACACAAATA | 10 | AGCTC |
| 10 | CTGCCCCAATCCCGTATTTC | 10 | AATTTCT |
| 10 | GACCATTTTTT | 10 | TCCCTG |
| 10 | GGTGAGGGCA | 10 | TACATATACATATA |
| 10 | CCCCCCCACCCCCC | 10 | GAATGGAATCGAATGGAATCACCATT |
| 10 | AAAGAAAAGAAAAAA | 10 | TCATTCTCTTTCTTTCTT |
| 10 | AAAAACAAAACA | 10 | TGTGTGTGATGAA |
| 10 | TCTCTCTCTTTCTG | 10 | GAAGAGGAGGAGGAGAAGAG |
| 10 | GAGAGAGAGA | 10 | TTTAAAAGTACAT |
| 10 | GAGGGACTTGCAACAATGGG | 10 | AGGGAGGGAGGA |
| 10 | ACTACGC | 10 | CACAAGCAC |
| 10 | TGTTGTCGT | 10 | CATGGCTCACCA |
| 10 | CCTTCCCCCCCC | 10 | GATGATTCCATTCGAGTCCATTCA |
| 10 | TACCAACAGCATCAACCA | 10 | TCCACTCTAT |
| 10 | TCCATTCCATTCAAA | 10 | AGAGAACATAATCATC |
| 10 | CTACAAG | 10 | GGCTTGGCTGCT |
| 10 | GGGAAGGAGGTGGGGAGGGA | 10 | GTAAGAAAAATTA |
| 10 | TTTTGTTTTAACTTTG | 10 | GACCATTGT |
| 10 | CCCCTTCCCCCCC | 10 | TCCCACCGA |
| 10 | CAACAGGTG | 10 | CCAGGCT |
| 10 | GGGGCGCAT | 10 | CTTCATGGCT |
| 10 | GGTCCCCCAGCTCAGACAG | 10 | GATGATTCCATTCGATTACATTTGAT |
| 10 | CTGAGGGAAGAGGGTC | 10 | ATGACTGT |
| 10 | GGTGGAGGTGAT | 10 | ATCATCGAAAGGACTCGAATGGA |
| 10 | TGCCGCATCCCA | 10 | TTCTTGCTCTTC |
| 10 | TGCAGCTAGCCACAG | 10 | CTTGACCTTGCAC |
| 10 | ATATATATTTGGGTGACTTA | 10 | GGAGGC |
| 10 | CAGTTGGACCTGTTTGAC | 10 | GGGGGAGGA |
| 10 | ATTCCATTAGATGATGATTCCTTTGA | 10 | TTCTTTTTTTTTTTTT |
| 10 | TCTCGCACGCAGAAAAATTATTGGTCGCGGGTC | 10 | GGCCCA |
| 10 | AAAACAAGTTTATTTTATGT | 10 | GGCGAG |
| 10 | CCCGGAATGTCATGATG | 10 | CCCTGATG |
| 10 | TTTTTTGAAT | 10 | CCCCAAAAA |
| 10 | TACTTC | 10 | TCTCACC |
| 10 | AGGAGCAGGAGG | 10 | GGAATCATCATCTAATGGAAATGAAT |
| 10 | ATGGACTGGAAAGAA | 10 | CAGAGACACACATTG |
| 10 | CGATCAAATCTTCATGATTCT | 10 | TGAATGGAATCGAATGGAATCATTAT |
| 10 | GACTTCT | 10 | TGCTCCATCCTAACTCATTCTGATCACC |
| 10 | GCGCGCTGGCG | 10 | GGGGAAAAGAGTGG |
| 10 | CATGGACAGTTTCCT | 10 | GGCTATCA |
| 10 | TCCATTTGATATGATTACATTCGAT | 10 | TAGTAGAATTGATTCTT |
| 10 | ACACAGACAC | 10 | CCGCCCCCCCC |
| 10 | TATCGGCAACGG | 10 | GCTGCTGCTGTT |
| 10 | AAAGATTTGCCTTAAT | 10 | GTTCTTGTT |
| 10 | GATAATAAAACT | 10 | AGAGACAGAAAGAGAGAAAC |
| 10 | TCCATGCCAT | 10 | CTCGAATGGAATCATTAAATGGA |
| 10 | AAGATGAAGATC | 10 | TAGGGGATCCAGACA |
| 10 | TTCTGG |
| 10 | TCCATTCGAGTCCATTCTTTGAT |
| 10 | TACAAAAAAAAAA |
| 10 | GCTACTG |
| 10 | CCTCCTCCACACGCC |
| 10 | TGATGGTGATGGTGA |
| 10 | ATTTCCTCCT |

| **Lionepha lindrothellus (#SRR5230400)** | | | |
| --- | --- | --- | --- |
| 6604 | TTTGTTA | 82 | AATCTG |
| 2641 | TCCAT | 80 | ACAAAAAATA |
| 1466 | GATCGGAAGAGCACACGTCTGAACTCCA | 80 | TTTTTTTTTTGTA |
| 1163 | TAAAAT | 79 | AGATCGGAAGAGCACACGTCTGAACTC |
| 750 | TTA | 76 | AAAAAAAAAAATA |
| 674 | AAAT | 76 | TTTATAAATTTTTATT |
| 643 | GAAATAA | 75 | TTTTTTTTGTAA |
| 580 | TTTTTTTTTTAA | 74 | AGAT |
| 518 | AAAAAAAAATTT | 73 | TGAAT |
| 514 | CGTGAGT | 72 | ATTCCATTCGATTCCATTCGATG |
| 432 | TAAAAAAA | 71 | TTATTTGTAATTTT |
| 420 | TTG | 70 | AAAACAA |
| 418 | CTTACGA | 69 | GGTCTT |
| 388 | ATTGTTCTGTTTTTTC | 66 | GAGTG |
| 351 | AAAAAAAATTA | 61 | TGATGATTCCATTCGATTCCATTCGA |
| 347 | **GGTTA** | 61 | AGATCGGAAGAGCACACGTCTGAACTCCAC |
| 339 | ATTTTTTTTTTAA | 61 | CTTCTT |
| 295 | AATGTATAATTATTTATT | 60 | CAGAACATGGAAAAAA |
| 292 | CGGAAGAGCACACGTCTGAACTCCCAGAT | 59 | AAAATTTAAATT |
| 272 | TTAAAAAAAAAAA | 58 | TTAAAAAAAA |
| 272 | CTT | 58 | TAATAAATTTATTATTAATATATA |
| 227 | AAAGTGAAAGGGA | 57 | AGAAT |
| 223 | TTTTTTATT | 56 | ATAAATATTTATTAATTA |
| 215 | ATAAAAAAAA | 55 | TGGA |
| 211 | GAAA | 55 | GAATGGAATC |
| 209 | CCCACTCCTTTCC | 55 | ATGATTCCATTCGATTCCATTTGATG |
| 204 | TTTAT | 54 | AAAAAAAAAC |
| 203 | AAGGAGTGGGGG | 53 | GCA |
| 188 | TATTATATAAATATA | 52 | TGGAATCGAATGGAA |
| 182 | CCCCACTCCTTTCC | 51 | TTTCGAAGAATTTTCC |
| 179 | ATTTATATATTAATATAA | 51 | CATGACAAAACAGAA |
| 176 | TTTTTTTTAAA | 50 | ATTTTTTTTTTTAA |
| 166 | ATCAAA | 49 | ATTTGTA |
| 162 | AAATACAAAAAA | 49 | AAAAAAAAAAATTA |
| 149 | TTTTTATTTTT | 49 | CATGAAAAAACAGAA |
| 147 | ACAAAA | 49 | TATAATTATTTATTAATA |
| 142 | AATTTTTTTTAA | 48 | GAC |
| 140 | GTTACTT | 47 | CCTTATTTCTTTTTGATT |
| 127 | TATTTTT | 46 | TTTAA |
| 126 | ATTACAAAAAAAA | 46 | AGATCGGAAGAGCACACGTCTGACTCC |
| 122 | TGTCTGTCTGGA | 45 | AAAAAATAAT |
| 122 | TCCAGATCGGAAGAGCACACGTCTGAACTCCAG | 44 | CCCACTCCTTTTCC |
| 122 | TCTTTTCTT | 44 | GATTTCTTGTGAGCG |
| 117 | AGATCGGAAGAGCACACGTCTGAACTCCAGTC | 43 | CTCCACTCACTCA |
| 112 | ATTACAAAAA | 39 | GGT |
| 112 | AAAC | 39 | CTTTTCCCCTTCA |
| 108 | AAAAAAAAATAA | 38 | TTATTATATATATTAATAATATAA |
| 107 | CTTACTT | 38 | TTCCCCCCCCCCC |
| 105 | TTTTG | 37 | TTTTTGTAATT |
| 102 | TTTTTA | 37 | AAAAAAAC |
| 98 | TTTTGTATTT | 37 | AGATCGGAAGAGCACACGTCTGAACTCCT |
| 96 | AAATAAAAT | 37 | AATG |
| 95 | TTTCTATTTCTTCTCCTT | 36 | ATCATCGAATGGA |
| 90 | TTCC | 36 | AGATCGGAAGAGCACACGTCTGATCTCC |
| 88 | TTTTTTTTAAAAT | 36 | GGGGAAGGAGTGGG |
| 87 | GGGGAAGGAGTGG | 36 | AGATCGGAAGAGCACACGTCTGAACTCCATCC |
| 85 | TTCTCT | 34 | AAAAAAATT |
| 82 | CAT | 34 | TTTTATTTTAT |

| 33 | AATATATAAAA | 19 | AAAAAAAAAATACA |
| --- | --- | --- | --- |
| 33 | CTTCCTCTTCGTCTT | 19 | ATATTTGATGTACTTTC |
| 33 | AAAAAGTATCTCATTTAAT | 19 | AAAAAAAAC |
| 33 | AAACAAAAAAA | 19 | TTTTTTGAATTT |
| 32 | TTTTTTC | 19 | AAAAAATAAAAAAC |
| 32 | AGATCGGAAGAGCACACGTCTGAACTCCAGTCCC | 19 | AGATCGGAAGAGCACACGTCTGAACTCCAGTCAC |
| 31 | AAAGA | 18 | TTTTCAAAATTTT |
| 31 | TTTTTTTATTAAA | 18 | ATACAAAAAAA |
| 31 | GGGGAAAGAGTGG | 18 | TTTTTTTTTAATTTTTTTTAAAATT |
| 31 | TATGTTATAATATAAT | 18 | AGATCGGAAGAGCACACGTCTGAACTCCAT |
| 31 | AAGGAGTGGGGAGA | 18 | GGGGAAAGAGTG |
| 30 | AAATATATATATTTTTTCGCGTTCTTTTGCGTA | 18 | AAAAAATT |
| 30 | ATAC | 18 | AGATCGGAAGAGCACACGTCTGATCC |
| 29 | ATGAATTTACCGGCG | 18 | TTCCATTCGATGATGA |
| 29 | TTATTATATATATTAATAAATAA | 18 | TTCTTCTAC |
| 29 | TTTGTTATTTTTTATTT | 17 | TAAAAAAATAA |
| 29 | AGAC | 17 | CTA |
| 29 | TTCTTCTTTCAAAGTTCTAGAATTTTCTGTCAA | 17 | GTTCTGTTTTTCCATT |
| 29 | AGATCGGAAGAGCACACGTCTC | 17 | CCCTAA |
| 28 | TTTTTTTAAA | 17 | AGATCGGAAGAGCACACGTCTGAACTCCAGTCT |
| 28 | AGATCGGAAGAGCACACGTCTGAACTCCTC | 17 | GCTTCCCCAATCTGCATGCCTTTA |
| 28 | TTATTTGTTATTTT | 17 | ATCTTC |
| 28 | TAGCGGTTTCTGACCGGTTTA | 17 | CTCCCCCCC |
| 28 | TGATTCCATTCGAGTCCATTCGA | 17 | AAATAATCGATTTATTAAG |
| 27 | TCC | 17 | ATTTCTA |
| 27 | TTATTTGTAATT | 17 | CAGATCGGAAGAGCACACGTCTC |
| 27 | CCTTTCACTTTTG | 16 | AAAAAAATAAAAAACC |
| 27 | AGATCGGAAGAGCACACGTCTGAACTCCCC | 16 | TCTCAGATCGGAAGAGCACACGTCTGAAC |
| 26 | CTCT | 16 | CTCCCCCCCCCC |
| 26 | TATAATAATTATATTATTAATTTA | 16 | AAAAAAAAATC |
| 26 | CAAAAAAAAAAA | 16 | TTATTATATTAT |
| 26 | TCTTCAC | 15 | TTTGCTTTCAACATTTTCAACTAATGCAAAGCGT |
| 25 | AGATCGGAAGAGCACACGTCTGAACCCC | 15 | TCAAAATTTTTT |
| 25 | AATAT | 15 | CCTTCCCCCCCCCC |
| 25 | CCAGATCGGAAGAGCACACGTCTGAACTCCA | 15 | CCATTCAATGATT |
| 25 | ACGCAAAAAAACGCGAAAAAATATATATATTTT | 15 | AGATCGGAAGAGCACACGTCTGAACTCCATCCC |
| 25 | CCTAAAC | 15 | TTTTGTAATTTTTGTTATTTTTTTT |
| 25 | ATCGGAAGAGCACACGTCTGAACTCCATCAG | 15 | TTTGTTATTTTTATTT |
| 24 | TTTTTTTAAAAAT | 14 | AAAAAATATA |
| 24 | TTTTTGTTATTTT | 14 | TTTAATTTA |
| 24 | CCCAGATCGGAAGAGCACACGTCTG | 14 | TTTTATTTCTTAT |
| 23 | TTTTTTTAAAA | 14 | TTTTTTGTA |
| 23 | AGATCGGAAGAGCACACGTCTGAACCC | 14 | CTCACA |
| 22 | GTACATAAAATATGAAA | 14 | GGGAAAAGAAGTG |
| 22 | AGATCGGAAGAGCACACGTCTGATCCC | 14 | GGAAGAGCACACGTCTGAACTCTCCAGATC |
| 22 | TTCTTTTT | 14 | ATTG |
| 22 | GGGAAAAGAGTGGG | 14 | CCCAGATCGGAAGAGCACACGTCTGA |
| 21 | GCGA | 13 | AGATCGGAAGAGCACACGTCTGAACTCCAGTCCT |
| 21 | AATTATATA | 13 | CTTTTT |
| 21 | GGGGAAAGGAGT | 13 | TTTTTCCATTGTTTTG |
| 21 | AAGAAGTGGGGG | 13 | TTTCTTCTTTTGTGGCC |
| 21 | AAATATATATATTTCACGCAAAAAACGCGAAA | 13 | TTCCTTCTAGATTTTT |
| 21 | TTTTTTTTGAATTT | 13 | AAAAATTTATTATTTAAA |
| 20 | CCCCCCCCCCCCT | 13 | TGTTGTTTCTGACGTAGATTGTTC |
| 20 | GTCGTCTTCTTTCTTC | 13 | TTTGTTATTTTTTTTT |
| 20 | ATATTAAT | 13 | CAATGGCCAGTAAT |
| 20 | ATTA | 13 | AGATCGGAAGAGCACACGTCTGCTCC |
| 20 | AAAAAATAAAAAACC | 13 | AATTTATT |
| 20 | TCCAGATCGGAAGAGCACACGTCTGAACTCC | 13 | TTTTTTTTGTAATTT |

| 13 | AATAAAAAAAAATT | 10 | AAATATATATATTTCACGCAAAAAAACGCAAAA |
| --- | --- | --- | --- |
| 13 | TTTCCCCCCCCCCC | 10 | GGGGAAAGGAGTGGG |
| 13 | GACAATTCACCGAAAA | 10 | TAAAAAAAAATAT |
| 12 | TTTTTTCTA | 10 | CTTGCTTT |
| 12 | ATCGCCGTTTCACAGCTCAA | 10 | CTTTCCTCTAATTTTTGTTCCAATGTTCTAGAA |
| 12 | AGATCGGAAGAGCACACGTCTGAACTCCA | 10 | TATAAATTAATT |
| 12 | TGATTCCATTCGATTCCATTAGATGA | 10 | AGATCGGAAGAGCACACGTCTGAACTCCATCTC |
| 12 | TATTG | 10 | AAAATAATAAT |
| 12 | AATGGAATGGAATCA | 10 | AAACCGCTCAG |
| 12 | TTTTTTCTTT | 10 | CCTTTAGCTTCTCCAATCTGCATG |
| 12 | CAAATTTGATAT | 10 | GTTCCTTCTTCTTCTTCC |
| 12 | TCCATTCCAC | 10 | TTTCCCCCCCCCC |
| 12 | CCCCCCCCCCTT | 10 | ATGATTCCATTCGATTCCATTGATG |
| 11 | GGAAAAGAGTGGG | 10 | AAAAAAATAAAAAA |
| 11 | AAAAATATACATATTTAACGCA | 10 | TATAAATTAATAAATAATTATTA |
| 11 | CCATTCGAGTCCATT |
| 11 | ATTTTTATAT |
| 11 | TATTAAAT |
| 11 | TTTTTTTTTGTAAT |
| 11 | AGATCGGAAGAGCACACGTCTGAACTCT |
| 11 | AAACGCGAAAAAATATATATATTTTACGCAAA |
| 11 | CCCCCCCT |
| 11 | AGATCGGAAGAGCACACGTCTGACTCCC |
| 11 | GAATCATCAAATGGACTCGAATG |
| 11 | GAACAATCTACGCCAGAAACAACA |
| 11 | AGATCGGAAGAGCACACGTCTGAACTCCAGTCACT |
| 11 | TTTTTTTTGAATT |
| 11 | CACAAGAAGCCGTG |
| 11 | AAAAAAAATAAT |
| 11 | AAACAAAACAATGAAA |
| 11 | TCCATTCCATTCCAC |
| 11 | AGATCGGAAGAGCACACGTCTGAACTCCACCC |
| 11 | AGATCGGAAGAGCACACGTCTGAACCTCC |
| 11 | TCCATTCCATTCGAA |
| 11 | AGATCGGAAGAGCACACGTCTGAACTCCAGTCACC |
| 11 | AAACCGGTCAG |
| 11 | TGATGATTCCATTCGATTCCATTCA |
| 11 | TATTTGTTGGTATTATTTG |
| 11 | AATTTACCACGCGATG |
| 11 | AACC |
| 10 | CTCTTCGTC |
| 10 | AAGAGGAAGAAG |
| 10 | GCACACGTCTGAACTTCCAGATCGGAAGA |
| 10 | CCATTCAATGATTCCATTCGAGT |
| 10 | ATTTGATGATGATTACATTCGATTCC |
| 10 | GCGTCGCGGCCACTCATTTCTG |
| 10 | GCCACTTCCCTGTGTGTCC |
| 10 | AAAAAAACAAAAA |
| 10 | ATTATAT |
| 10 | TTGTTGT |
| 10 | GTTTTATCATTGTTCT |
| 10 | TTTTATTTTTTA |
| 10 | TTAAAT |
| 10 | AAACAATTAATTATAT |
| 10 | TTTTTTTTGAAA |
| 10 | CCCTCCCCCCC |
| 10 | TTTTTTTTC |
| 10 | TCATTTG |
| 10 | CTCTCC |

| **Pogonus chalceus (#SRR5427959)** | | | |
| --- | --- | --- | --- |
| 8413 | **AACCT** | 13 | TGC |
| 6024 | TATATACT | 13 | TATTAA |
| 1088 | TGT | 12 | ACAAGA |
| 905 | ATAG | 12 | ACCTAACACTA |
| 635 | TTA | 12 | TTAATATA |
| 323 | GAC | 12 | GTATATACTTATATAC |
| 240 | AACTAACCGAACTTAA | 11 | TTATCATTCGGCGCCT |
| 217 | AATA | 11 | TAC |
| 165 | AAAG | 11 | AAAAAAAT |
| 122 | ACAT | 11 | TATAG |
| 122 | TGTGTTGTATTT | 11 | TTAGTTATAAGTTCGG |
| 118 | ATATATAAACGTT | 10 | TTGCGAGCTA |
| 117 | AGTATATACTTATATA | 10 | TAATAATAAAA |
| 112 | ATTTACTGA | 10 | TATATAATTATATAAG |
| 100 | AACCTAACATAACCT | 10 | ATTGTCGTA |
| 99 | TCA | 10 | TATATG |
| 86 | ACCA | 10 | AGCAAC |
| 80 | CAAAATACTGAAAACATT |
| 80 | TGTTA |
| 75 | AAAAATGAAA |
| 74 | CTTA |
| 74 | AAG |
| 72 | ATAATTAT |
| 66 | AGCTCACAGAC |
| 54 | CTCGTCTTGT |
| 49 | TAGTTTTAAGTTCGCG |
| 46 | CGTCT |
| 45 | GTATATAC |
| 44 | CTTGTACA |
| 43 | GTATATAT |
| 43 | TAAAATTCTCTA |
| 42 | AAAATCAGTA |
| 38 | TAAAA |
| 37 | CGAACTTAAAACTAAA |
| 36 | CCGTTTAGTTTTAAGT |
| 34 | AAGTTCGGTTAGATTT |
| 31 | GTTTTCAAAAATATGTCTTA |
| 30 | GTAAC |
| 30 | AATTTTTCTTCT |
| 28 | TTATTGTTTTA |
| 28 | AAGTCCGGTTAGTTTT |
| 28 | ACCTAACCTTC |
| 27 | TGTTTTA |
| 27 | ACTTTTTTCAAGAAAAATTAC |
| 26 | ACAAC |
| 26 | ACAAAAC |
| 25 | ATAATAATAAT |
| 25 | CTTTTTTTTT |
| 24 | AACA |
| 24 | TTATTG |
| 21 | AAAAAAAAT |
| 20 | TTTTTTTTTCA |
| 18 | CCTGAAGA |
| 17 | AGCAA |
| 17 | AATT |
| 15 | TTTTTAACAAAT |
| 15 | TACCCGAACTTAAAAC |
| 14 | ATTCC |

**Dytiscoidea, Dytiscidae:**

| **Stictotarsus aequinoctialis (#SRR1145745)** | | | |
| --- | --- | --- | --- |
| 9954 | ATAC | 186 | GTTCTGTTTTTTCT |
| 7604 | **AACCT** | 183 | CTCTATATAAGCAAC |
| 2613 | ATACCGGT | 176 | GTGTTT |
| 2349 | TGTG | 175 | CGACATTTTGTCCTCG |
| 1703 | GTGTAT | 175 | CTTTTTCTCCTTAGCAAGATGTGTAAAA |
| 1703 | TTAGCAAGACGTGTAAAACTTTTTCTCC | 172 | AAAC |
| 1671 | ATACACAC | 162 | ATGATGAATAACGAATACTCACGA |
| 1650 | TCTTACTAAGAAGAAAAAATTTTACTCG | 153 | GAACTGGTGAACAGACCTGTTT |
| 1354 | TCGCTGTTCTGTTTTG | 152 | ACACACAC |
| 1324 | CACG | 148 | GACA |
| 1119 | GTGCTAGAGTGATA | 141 | AATACCGGTATT |
| 1118 | GTTCTGTTTTTTCAT | 138 | ATTATTGACCTATGAAAATA |
| 1040 | TACATA | 138 | GTTTTACTCGTCTCACTAAGAAGAAAAA |
| 1016 | CCTATGAAAATAATTATCGA | 137 | TGTGTGTTTGTG |
| 985 | CACACA | 133 | ATTCGTTATTCATCAATC |
| 930 | GTGTGC | 122 | GTAAAATTTTTTCTCCTTAGTAAGACGT |
| 820 | CTTAGTAAGACAAGTAAAATTTTTTCTT | 121 | GGACAAAATGTCGCC |
| 818 | CAAAACAGAACAGTGA | 118 | CCTTAGCAAGACGAGTAAAATTTTTTCT |
| 693 | ATGTGTGTGT | 118 | ATT |
| 624 | TAGTAAGACAAGTAAAATTTTTTCATCT | 116 | TGTATGTGTGTA |
| 585 | GTAGTGTAAAGTGGATGGTGTGTAGTTG | 116 | CCATCCACTTTACACTACCAACTACGCA |
| 575 | GCCCAGTTGTCCGTC | 115 | CACTAAGATGAAAAAATTTTACTCGTCT |
| 573 | TCTTACTAAGGAGAAAAAATTTTACTCG | 112 | GTTTTACTCGTCTTGCTAAGAAGAAAAA |
| 520 | TCTTAGTAAGACGAGTAAAATTTTTTCA | 111 | CACACACT |
| 493 | TCCCGGGA | 111 | ACATACACACACAT |
| 478 | CGTCTTGCTAAGAAGAAAAAGTTTTACA | 109 | GACCGAAC |
| 473 | TTTTCTCCTTAGTAAGACAAGTAAAATT | 108 | GTCCAGTTGTCCGTC |
| 446 | GTGTGTATGTGT | 105 | CTAAGAAGAAAAAATTTTACACGTCTCA |
| 440 | AAACAGAACATGACA | 104 | GTATATGTGTGT |
| 431 | AAACAGAACACGAAA | 104 | ATGTATAT |
| 419 | GTGTGTGC | 104 | AGTAAGACAAGTAAAATTTTTTCTCTT |
| 395 | TTAGCAAGACGAGTAAAATTTTTTCTTC | 104 | GTGAGTGTGT |
| 388 | ACTAAGAAGAAAAAATTTTACTCGTCTC | 102 | ATACACAT |
| 357 | ACCAACTACACACC | 100 | GTGTGTGTGT |
| 352 | ACACCATCCACTTT | 95 | TTTTTCTCCTTAGTGAGACGAGTAAAAT |
| 351 | GAAGAAAAAATTTTACACGTCTTGCTAA | 92 | TGTCTTACTAAGAACAAAAAATTTTACT |
| 349 | CGTGTAAAATTTTTTCTCCTTAGCAAGA | 89 | TTTTTCATGTTCTG |
| 327 | TGTTCATACGAACGTACATT | 88 | GTGATATTTTTCGC |
| 316 | GTGCTAGAGTGATG | 88 | CACACACACACT |
| 315 | AAAT | 87 | GTGCGTGCGTGT |
| 301 | GTTCTGTTTTTCGCT | 87 | TAGTAAGACGAATAAAATTTTTTCTTCT |
| 283 | GTAGTTGGTGGTGTAAAGTGGATGGTGT | 86 | CACACACACGCACA |
| 260 | GAAGAAAAAATTTTACACGTCTTACTAA | 86 | TCTTACTAAGAAGAAAAAGTTTTACACG |
| 232 | ATAG | 85 | TGACAAAACAGAACTA |
| 226 | TGTGTTTGTG | 83 | GAGT |
| 225 | GTGTGCGTGT | 82 | TAATTATTTTCATAGGTTGA |
| 222 | TGTTCGTACGAACGTACATT | 82 | CAGTAGAAATATTAATAAGAAA |
| 221 | CACACACACACG | 81 | CACTCA |
| 217 | CTAGCACTATCACA | 80 | TAGAGTGACAGTGC |
| 214 | AAAACAGAACAGCGAA | 80 | GGAAAGTTCACCGAC |
| 212 | TCT | 80 | TGTGTATGTA |
| 210 | ATACTGACCATTTAATTAAA | 79 | TTCACCGACTGCAA |
| 206 | GTCGGTGAATTTTCCA | 76 | CACACACACAAACA |
| 206 | GTGTGTTT | 72 | TAAAATTGGA |
| 202 | ACACACACACACAT | 71 | CGTCTTACTAAGAAAAAAAAATTTTACT |
| 201 | TACCACACAAATCTACCAGAGACACC | 71 | TTCA |
| 193 | TCA | 69 | CTAGCACCATCACA |
| 191 | TCATATGTTCTGTTTTG | 69 | GTCTTACTAAGAAAAAAAATTTTACTT |
| 68 | TATTCGTCTTACTAAGGAGAAAAAATTT | 40 | GACCAACAATTTTTTTTTTCTTAGTAA |
| 67 | AAATA | 40 | CACTCACACACACA |
| 67 | TATTCGTTGCCACCTTTA | 40 | GTCTTACTAAGAAGAAAAATTTTACTT |
| 67 | TGTATATGTG | 39 | TTTGTGTGTGTATG |
| 65 | TTTTTTTTTA | 39 | TCTTAGTGAGACAAGTAAAATTTTTTCA |
| 63 | AATACTGACCATTTATATAA | 39 | ACACTACCAACTAC |
| 61 | GTTGCTTATAAAGAG | 39 | GGTGTGTAGTTGAT |
| 61 | ACACACACATATAC | 38 | GAAAAAGTTTTACACGTCTTGCTTAGGA |
| 61 | CTAATCAATTTCAAAATGAGA | 38 | ACATATATACACGTACAT |
| 60 | GTCTTACTAAGGAGAAAAATTTTACTT | 38 | GTACAAGACAAAATTTTTTTTTCTTAGTAA |
| 59 | TTTTGAAATTGATTATTCTCA | 38 | CACACACATACACAAA |
| 58 | TGGTGGTGTAAAG | 38 | ACAAAAAATA |
| 58 | GTCTTACTAAGTTGAAAAAATTTTACTT | 38 | TATAAAAAAATG |
| 58 | AAAAAAAAT | 37 | ACAGTG |
| 58 | TGTGTGTATGTGTT | 37 | TACTATTTCTTATTAATATTTC |
| 57 | CTACACACCATCCA | 37 | CCACTAATACACGCACACACCAAATA |
| 57 | GTCTTAGTAAGTGAAAAAAAAACATTTTTTCTTG | 37 | CACACATACACACT |
| 54 | CTAACCTTAC | 37 | AGACGAGTAAAACTTTTTCTCCTTAGCA |
| 54 | TGCTAGAGTGATGA | 36 | ACATTATAAAATAATTGAAAA |
| 53 | TATGTGTGTGTGTGTG | 36 | ATGTAC |
| 53 | TTTTTTTTTTA | 36 | GTCCGTTATATCGAG |
| 52 | TGTACGTA | 36 | GTTCTGTTTTTCACT |
| 52 | GTCAGTATTTAAATTAAATG | 36 | GGGCGACGGACAACC |
| 52 | TTGAAATTGATTATTCTCAAT | 35 | TTTTATTT |
| 52 | TTCTTAGTAAGACGAGTAAAACTTTTTC | 35 | TCTGTG |
| 51 | ACTAAGACCAAGAAAAAATGGTTTTTTTTCACTT | 35 | ACGT |
| 51 | GTGTAC | 34 | ACACACACATAA |
| 51 | GAAAAAATTTTACTTGTCTTAGTAAGGA | 34 | TGTATGTATGTACG |
| 50 | TACTAAGACAAACAAAAAATTTTTTCTTCT | 34 | GTAAGACAAGTAAAATTTTTTCATCTAA |
| 50 | TAATGAAAATTTTT | 34 | GTGTAAAATTTTTTCATCTTAGTAAGAC |
| 49 | CACATACACACATA | 34 | ATATGTATGT |
| 49 | TCACTGTCACG | 34 | CACATACACACACAAA |
| 49 | TCTTACTAAGACAAACAAAAAAATTTTTTCT | 34 | TAGTAAGACATGTAAAATTTTTTCATCT |
| 48 | AAAGTTGATGGTGTGTAGTTGGTAGTGT | 33 | AACGAATGATGAAT |
| 48 | AAATTTTACTCGTCTTACTAAGAAGAA | 33 | CAACTACACACCATC |
| 48 | AAACAGAACAGCGAAT | 33 | TCACACACACTC |
| 48 | CATGCATA | 33 | TCTTACTAAGAAGAAAAAATTATACACG |
| 47 | GCGTAT | 33 | CACATG |
| 47 | ATTTTGTCCGCGAC | 33 | ATGC |
| 47 | ATCTTGTACTTACTAAGAAAAAAAATTAC | 32 | TGTCAGTC |
| 46 | CAACAACAACCT | 32 | GTATTTGATGTGTGCGTGTATTAGTG |
| 45 | TAGTAAGACGAGTAAAATTTTTTGTTCT | 32 | CAACAC |
| 44 | TAG | 32 | AATAAAA |
| 44 | TTTTACACGTCTTGCTAAGAAGAAAAA | 31 | GTCTTAGTAAGAAGAAAAAATTTTTTTCTTT |
| 43 | GTATATATGT | 31 | TTTTACTCGTCTCACTAAGTTGAAAAAA |
| 43 | CGAGAAAATATCACGG | 31 | GTGATATTTTCGC |
| 43 | TATAGGTCAATTGATAATTTTC | 31 | CACGCACACG |
| 42 | AGGTCAAACAGAC | 31 | ATCAGAT |
| 42 | TAGTAAGACGAGTAAAATTTTTTCATTT | 31 | TACTAAGACAAGTAAAATTTTTTCTTCT |
| 42 | AAACACACACAA | 31 | CGTACGAACAAATGTACATT |
| 42 | GACGAGTAAAATTTTTTCTCTTAGTAA | 31 | GTGTTTGTATGTGT |
| 41 | TGT | 30 | GTAGTAGTTGTA |
| 41 | CACACACATACTCA | 30 | TCAACTTCAAAATGAGACTAA |
| 41 | GTTTAACCTGTCTGTCT | 30 | TTAAAAAAAAA |
| 41 | AAAAATATTATAAAATAGTTG | 30 | ATATACATACAT |
| 41 | GAGA | 30 | AAACACAAACAA |
| 41 | GTCGATGGTGTGTAGTTGGTAGTGTAAA | 30 | ACACACACATTCAC |
| 41 | AGTAAAATTTTTTCTTAGTAAGACA | 30 | GTGTGCATGTGT |
| 40 | TAGTAAGACGAGTAAAATTTTTTTTCT | 30 | CACATATACATA |

| 30 | TACATTTGTTCGTACGAACA | 22 | CACATACAAA |
| --- | --- | --- | --- |
| 30 | ATTTTACTCGTCTAACTAAGAAGAAAAA | 22 | GTGTGTGCGCGT |
| 30 | GCCGTCTCAGATGCT | 22 | TGTTCATACAAACGTACATT |
| 30 | TTGCTAAGGAGAAAAATTTTACACGTC | 21 | GATAAATAACGAATACTCACGAAT |
| 30 | ACACACACATATAT | 21 | TTTGAT |
| 30 | AGAGAC | 21 | AAACACACACACACAC |
| 29 | GAAATTCACCGACT | 21 | AGTTGGTAGTGTAA |
| 29 | TTACTAAGAAGAAAAAATTTTAGACGTC | 21 | GTCTGTAT |
| 29 | AAATGTACATTCGTATGAAC | 21 | AACATACATTTGTTCATACG |
| 29 | CTGTT | 20 | CTCTCT |
| 29 | CATATG | 20 | TAGTAAGACAAGTAAAATTTTTTCTACT |
| 29 | AGTAAGACAAACAAAAAATTTTTTCTTCTT | 20 | AGGACGAAAGACGA |
| 28 | AAACAGAACACGACA | 20 | TATATGTATGTACGTATA |
| 28 | GTATGTATATGTGTGT | 19 | TACACACACACAAA |
| 28 | CACCTGGAC | 19 | AAAAACAGAACAGCGAC |
| 28 | TCACACACACACTC | 19 | AAGCAACCTCTATAC |
| 28 | GTACGAACGTACATTTGTTT | 19 | CACACACACACGCACA |
| 28 | TTTGTAAATT | 19 | ACACACACAG |
| 28 | TAGTAAGACAAGTAAAATTTTTTGGTCT | 18 | GACGTGATATTTTC |
| 28 | TTTTTTTTTCACTTACTAAGACCAAGAAAAAATA | 18 | AAACAGAACAATGACA |
| 28 | GTGTATGAACTAAGAAAATATTTTTTTTTCTCT | 18 | ACACCACCACTAC |
| 27 | ACACACACACATAA | 17 | ATTTTACTTGTCTCACTAAGAAGAAAAA |
| 27 | GAAATATTAATAAGAAATAGGA | 17 | AACCTCGATATAACG |
| 27 | CTTAGTAAGACGAGTAAAATTTTTTCAA | 17 | GTCTTAGTGAGTGAAAAAAAAATATTTTTTCTTG |
| 27 | CAAAA | 17 | TTCACCGACAGCAA |
| 27 | GTGCGC | 17 | AGAACAGGAAAAAAC |
| 27 | TTGTTTTGTCT | 16 | TACGTGTG |
| 27 | TGGTGTATAGTTGG | 16 | AGAAAAAGTTTTACACATCTTGCTAAGA |
| 27 | ACATACACAAAC | 16 | ATTTATCTCTTCTTTTACCTC |
| 27 | TCTGTTTTTGCGAGT | 16 | GTCCTTATTCCAGTGTGAGATATTTTTCCATGT |
| 26 | ACACCACCAACTACACTCCATCCACTTT | 16 | GTTTTACACGTCTCACTAAGAAGAAAAA |
| 26 | TTTTTTTTCTTAGTAAGACAAGTAAAAT | 16 | AGTTGGTGGTGTAA |
| 26 | ACAGAACACGAAAA | 16 | CACACACATACACACACA |
| 26 | CACAGACA | 15 | GACC |
| 26 | GTATAC | 15 | AAACAAGATTTACAGTC |
| 26 | CAAAACGAAGAAATGAAGAAG | 15 | ACTT |
| 26 | TGTATGTA | 15 | GTCTTACTAAGACCAAAAAATTTTACTC |
| 26 | TTTTTTTTGT | 15 | TACTAAGACAAACAAAAAAAATTTTTTCTTCT |
| 25 | TTGTCTGTGTTT | 15 | TTTCGACGTGATATTTTTTCGCGTGATAT |
| 25 | CACACACACATACATA | 15 | AAAAAAAAAATA |
| 24 | TCTTAGTAAGACGAGTAAAATTTTT | 15 | ATACACACATACAT |
| 24 | TTACTAAGGAGAAAAAATTTTACATGTC | 15 | CAAAAAAAA |
| 24 | AATACCGGTATACCGGTATT | 15 | TGTTCTGTTTTGTCC |
| 23 | ATATATACACAC | 15 | ATACAAACACAC |
| 23 | TGTGTGTGTATT | 15 | CGGAAAATTCACCGA |
| 23 | CTACACACCATCCACTTTACACAACCAA | 15 | AGTAAGACAAGTAAAATTTTTCATCTT |
| 23 | TGTATGTGTGTGTATG | 14 | AAAAAAAAAACA |
| 23 | TATGTATATGTGTG | 14 | GTGCTAGAGTGATGA |
| 23 | ACAAAATTTTTTTTTCTCTTACTAAGACAA | 14 | TTTTTTTGTTCTTAGTAAGACCAACAA |
| 23 | CAACAACACCTTCAGAGA | 14 | TGTGTGTGTGTGCA |
| 23 | TATAAGCAACCTCTC | 14 | ACAAGCACACAC |
| 23 | TTTCTTCTTAGCAAGACGAGTAAAATT | 14 | CACACACGCACGCA |
| 22 | TACTAAGACAAACAAAAATTTTTTCTTTCT | 14 | TACCACTAATACACGCACACACAAA |
| 22 | GACAAGTAAAATTTTTTCGTCTTAGTAA | 14 | GAAGAAATGAAACAACAAAAT |
| 22 | CAAAACAGAACAGAGA | 14 | ATAGACCTATGAAAATAATT |
| 22 | TACTAAGACAAACAAAAAAAATTTTTTCTTTCT | 14 | CTTT |
| 22 | TAGTAAGACGAGTAAAATATTTTCTCCT | 14 | TTTTGCGCTGTTCTGT |
| 22 | GTAAAATTTTTTGTACTTAGTAAGACGA | 14 | **CCCCAAA** |
| 22 | GAAATTGATTAGTCTCAATTT | 13 | ACTAAGAAGAAAAATTTTACTCGTCTC |

| 13 | AAGATAAACACATCCAGTCTGAT | 11 | TGACATGACAG |
| --- | --- | --- | --- |
| 13 | TACTAAGACAAACAAAAATTTTTTCTTCT | 11 | TTGTTGGTCTTACTAAGAACAAAAAAAA |
| 13 | ACACACACAAACAA | 11 | AAGAAGAGATAAATGAAGTAA |
| 13 | AAAAAATTAAAA | 11 | CGGTGAATTTTCCATG |
| 13 | GTCTAACTAAGATGAAAAAATTTTACTT | 11 | GGCGACGGACAACT |
| 13 | AGTCACAGCACAA | 11 | CTATCACTCTAGCAC |
| 13 | CCGCGACATTTTGTC | 11 | TATAGATCAATTGATAATTTTC |
| 13 | ACACAAC | 11 | TTGCTAAGGAGAAAAGTTTTACACGTC |
| 13 | GTCTTACTAAGAAGAAAAAATTTACTC | 11 | ATTTTGTACTGTAA |
| 13 | TAATAAGAAACAGGAGAAATAT | 11 | TCTGTTTTTCCGAGT |
| 13 | CTGTCTGTCTGTTTAACCTGT | 11 | ACAGGTCAAACAGACAGACAG |
| 13 | GTCTTAGTAAGAAGAAAAAAATTTTTTTTGTTT | 11 | CGTCTATAGAACAAATGAA |
| 13 | TATAATTCGT | 11 | ATTTTCTTCAGTTGTGGTACCTTCTGTCTT |
| 13 | CACACACGCG | 11 | TACACACACACACATA |
| 13 | TATACACACACACATA | 11 | GTTTACAGTACAAAT |
| 13 | TAGTAAGACGAGTAAAATTTTTCTCCT | 11 | GTCTTACTAAGAAGAAAAAATTTTTCATTT |
| 13 | AAATGTACGTTCATACGAAC | 11 | ACACATAA |
| 13 | CTCTAGCACCTGTCA | 11 | CCATTTAATTAAAATACTAA |
| 13 | GTACTTACTAAGGAAAAAAAAATTTTGTCTT | 11 | ACACGCACACACGC |
| 12 | GACACTGTTGAAAAAGTTAACGCCA | 11 | CACAAACACACATACA |
| 12 | CACACCATCCACTT | 11 | CAAAACAGAACAGCGACAAAACAGAACAGTGA |
| 12 | CTACACACCACTCACTTTACACCACCAA | 11 | AGCAACATCTATATA |
| 12 | TCCCGGAA | 11 | AGCACACACACACA |
| 12 | AAAAAAAAACA | 11 | TACTAAGACAAACAAAAAAAATTTTTCTTTCT |
| 12 | AAAAATTTTACTTGTCTTACTAAGAA | 11 | AAAAAATTTTC |
| 12 | CCATCCACTTTACACTGCCAACTACACA | 11 | TATGTTTGTGTG |
| 12 | CCTAACCTAACCTTA | 11 | AACAAAA |
| 12 | GTTGGTGGTGTAAAGTGGATGATGTGTA | 10 | TTTTTATACTGACCAAAAAATA |
| 12 | TTTTCTTTCTTACTAAGACAAACAAAAAAAAA | 10 | GTGATATTTTCTCGC |
| 12 | GCGCGTGTGTGTGT | 10 | AACTTAAAATT |
| 12 | TATA | 10 | CATACACG |
| 12 | CAAAACAGAACAGCAA | 10 | TCTTAGTAAGATGAAAAAATTTTTGTTTG |
| 12 | TGTGTGAGTA | 10 | AAATATCTCAGGTACACCCGAACGCTCGTC |
| 12 | TATCACTGTTCTGTTT | 10 | CCAGTTGTCCGTCAC |
| 12 | CTACCACTGTGTCAA | 10 | ATCTTGTACTTACTAAGAAAAAAAAATTAC |
| 12 | TAGTAAGACGAGTAAAATTTTATCTTCT | 10 | GTGTGTGTGTTGT |
| 12 | CTTTACACCACCAACTACGCACCATCCA | 10 | TTTACACGTCTTGCTAAGGAGAAAAAG |
| 12 | GTCTTAGTAAGAAGAAAAAAAAATTTTGTTT | 10 | CTCACAAACACATA |
| 12 | TACTAAGGAAAAAAAATTTTGTCTTGTACT | 10 | GAAAAAATTTTACTCTTCTTACTAAGAA |
| 12 | ACTATTGC | 10 | TTATTTTCAATTTT |
| 12 | GTTGCTCTGAAGGTGTT | 10 | ACACACACGTAC |
| 12 | CACACACAA | 10 | AAAGAGAGAG |
| 12 | ACACACGCACGCACAC | 10 | ACACCAACCACTTT |
| 12 | ACTATACACCATCC | 10 | CGGTGAATTTCCAGT |
| 12 | CACAAACACACATA | 10 | AGTTGATGGTGTAA |
| 12 | AAAGAAAAAA | 10 | TGTTCTGTTTAGTCAC |
| 12 | AGTCGGTGAATTGT | 10 | ATAGGTTAATAATTATTTTC |
| 12 | AAATGTACGTTTGTACGAAC | 10 | TCTTACTAAGGACAAAAAATTTTACACA |
| 12 | TTGTCCGTCGCCCAA | 10 | GATT |
| 12 | CAATTTTA | 10 | AAATAATACAATGT |
| 11 | TACACATATACACA | 10 | ATACCGGTATTCGGT |
| 11 | TAAGATAAGAGA | 10 | GTGTGCAT |
| 11 | GGTCAGTATTTAATTAAAT | 10 | ACACACAATCAC |
| 11 | TCTTACTAAGAAGAAAAAATTTACTTG | 10 | CCACCAACTACACACCATCCATTTTACA |
| 11 | ACATACAA | 10 | TTAGTATAATA |
| 11 | GTGTGTGTGG | 10 | ACTTAGTAAGACGAGTAAAATTTTTTCT |
| 11 | TTTTTTCTTTCTTACTAAGACAAACAAAAAAA | 10 | TTCATTTCTTGT |
| 11 | ATAATTATTTTCATAGGTT | 10 | CCACACCGGTGACAACTA |
| 11 | CTTAGTATGTGAAAAAAAAACATTTTTTCTTGGT | 10 | GTCTTAGTAAGAGACAAAAATTTTTTTCATTC |

**Chrysomeloidea, Cerambycidae**

| **Phymatodes lengi (#SRR6984058)** | | | |
| --- | --- | --- | --- |
| 7296 | ATA | 100 | TCTGTATGTATG |
| 2361 | AAAT | 99 | TTTCAAAAATTCAAAAT |
| 2245 | GTAT | 92 | AAAAACGA |
| 1970 | **CTAAC** | 90 | TATATAA |
| 927 | CAAAAATTTAAAATTTG | 89 | GATTGAT |
| 796 | GTCTGTAT | 88 | CTTTGAAGTGACAATTGTCA |
| 777 | AATTTTGGATTTTTGAA | 88 | TACGTACA |
| 595 | TAGA | 86 | AATTA |
| 566 | TAC | 85 | ACCTACAT |
| 450 | AGAC | 85 | ATACAGACAGAC |
| 394 | CCTTTGT | 84 | TTTTGAAAATTTTGAT |
| 389 | ATTTCAT | 83 | GCA |
| 341 | CCCTGCCG | 83 | GTTTATCAATCGC |
| 285 | TAGAA | 79 | CCTATTATC |
| 271 | TATGTA | 78 | ATATGTTCGGGAACACTCGG |
| 258 | GGTA | 75 | AAAAAAAT |
| 256 | AATTGTCGATTCAAAGTGAC | 74 | ATATAATAGTCA |
| 255 | TGT | 72 | CTACGATGATATACTTTATATT |
| 249 | TCTGTATA | 71 | TTTTTTA |
| 248 | TGAGTATCAT | 71 | ATCCT |
| 246 | GTAAGTAAAAC | 71 | TAAAGAAAAAC |
| 233 | ATAAT | 68 | TTTATTTTA |
| 221 | TAATAG | 68 | TTTTTTTTTA |
| 201 | ATTTTTGAAA | 67 | ATGTAATGT |
| 201 | AAATA | 67 | GTTTTACTTACT |
| 200 | TTATGTTCTACGATGATATACT | 67 | ATGTTTTAAAC |
| 199 | TCA | 66 | TTCAA |
| 195 | TAGGTT | 65 | ATAGTAATA |
| 194 | GCAAAATTTAAAATTT | 65 | TCTAATTCCCACGACA |
| 191 | TAATAAAATTCCGAGTTTAAAGTCGGAATT | 65 | ATAAAT |
| 185 | GATTTTTGAAAATTTTT | 63 | ATTATTA |
| 176 | AATCTTT | 63 | TTTTGA |
| 172 | CAGT | 62 | TATATTTA |
| 166 | AGCCACAGAGCATCC | 62 | ATTTCCTTAGATGCAGGAAATATTCAG |
| 164 | GTCTC | 61 | GAAATACATG |
| 164 | TTTAAAGTCGGAAAAAATAAAATTCCGAG | 61 | TAAG |
| 163 | AAAC | 61 | TGTGCGTGTG |
| 154 | CGT | 61 | ATAAGTAAAAC |
| 151 | TAAAATATA | 60 | GAGGAA |
| 147 | TGG | 60 | ATAGAACT |
| 133 | AGATATAAGTCGTAATTCCG | 59 | GATGCAGGAAATACTCAGATTTCCTTA |
| 132 | GATG | 59 | TGCTGCATTATCTTTTTATCAAATGCAGGAG |
| 128 | TTC | 58 | TATCAATCGCTA |
| 128 | TAAATAA | 57 | TTTTTATTT |
| 128 | AATT | 56 | CACG |
| 127 | CGAGATATAAGTCGTAATTA | 56 | TTTTTGAAAATTTTAAA |
| 126 | ATATATAC | 56 | AAAATTGAA |
| 118 | ATTGT | 55 | GTATGTATAT |
| 118 | CGACTTTAAACTCGGAATTTTATTTTTTTC | 55 | CAGGTAGA |
| 109 | TTTTTATTCTAC | 55 | AATACT |
| 109 | ACGCAC | 55 | CGAGTTTGAAGTCGAGATCT |
| 108 | TGCTGCACTTTATTAGCTAATGCAGGAT | 54 | TTAATA |
| 107 | GACAGACC | 53 | ACTACCTTAATTAACA |
| 106 | ATATGTTTGGGAACACTCGG | 52 | TGTATGCG |
| 105 | TCCTGCATTTGATAAAAAGATAATGCAGCAA | 52 | AATATAC |
| 104 | ATTATATT | 51 | TATGTGTA |
| 102 | ATTTTT | 51 | TGTTAATTAAGGTAGC |
| 101 | TTAGCTAATAAAGTGCAGCACTCCTGCA | 49 | CAATATAGA |
| 48 | AGTATTCCCAAACATATCCG | 33 | TGATGAA |
| 48 | ACAAAAAAAA | 33 | TTATTTCA |
| 48 | ATTAGTA | 32 | GACTTATATCTCGTAATTGC |
| 48 | TCTGTGGCTGGGTGC | 32 | ATCTAT |
| 47 | GTATATATCAAATT | 32 | AAGCCACAAAGCAAC |
| 47 | AAAAAAAAAAT | 32 | ACATATT |
| 47 | TTTATAT | 31 | TAACTAAA |
| 47 | CGGAAAATAATAAAATTCCGAGTTTAAAGT | 31 | CACA |
| 46 | CATTTA | 31 | TAATC |
| 46 | ATTATTATTT | 31 | AATTAAAATGA |
| 46 | GAAGAAA | 31 | CTTTATGTCCTACGATGATATA |
| 45 | TCATGAA | 31 | AAAAATAAT |
| 45 | CTATTCGTT | 31 | ATAATATAATA |
| 44 | ATGTTCGGGAAACTCGGAT | 31 | ACCTTGAACAAGGTCGTTAACCTTTA |
| 44 | ACTATAAT | 30 | TTTAAAT |
| 44 | ATTG | 30 | TGAATTGAGAAAAA |
| 44 | AAGTAAAACAA | 30 | ATTGGC |
| 44 | GATTTTTGAAAATTTTC | 29 | AGCACAAAATT |
| 44 | TTTTGAAAA | 29 | ACTATAT |
| 43 | TAATATTAA | 29 | GAGAGAG |
| 43 | TAGTC | 29 | AGTATTTAAATACTTTCAT |
| 43 | CGGA | 29 | AATAAAAT |
| 43 | TTAGGTTTAGG | 29 | TCTTTC |
| 43 | CAACCATCTTTT | 29 | GTAAATAA |
| 43 | TTATAA | 29 | ACACCC |
| 42 | TACGAAA | 29 | TGGCGG |
| 42 | TACTA | 29 | AATAATTAAATA |
| 41 | TTAG | 29 | AATTTTGGATTTTTTAA |
| 41 | TTTAAT | 29 | ATAATTATTG |
| 41 | AGTATTTAAATACTTTTG | 29 | TTGATTTTGAAA |
| 40 | TTTTAG | 28 | ACACAGAC |
| 40 | ATTTTTGAA | 28 | ATATATAAT |
| 40 | AAAGGACA | 27 | ATGAAATGA |
| 40 | TTTAG | 27 | GTGTAT |
| 40 | TGTGTG | 27 | ATGTTTTAT |
| 39 | CGATGTAGATCTAGATGACTTTTCGTCTGA | 27 | GTTCATGATTCGTTC |
| 39 | AAATTTTTGC | 27 | TACTCAAATGA |
| 39 | TTAATTTAA | 26 | ATGGTTGTAT |
| 39 | TTTTACTTATTG | 26 | TAATTAATAA |
| 38 | AACATAGA | 25 | TCCCATCCA |
| 37 | TGCTATTGCTT | 25 | TTCAAT |
| 37 | TATAGTGTAG | 25 | TTGACAAGTC |
| 37 | CAATTA | 25 | ATTTAAAGTGACAATTGTCG |
| 36 | CACTTCACATT | 25 | TATATCTATC |
| 36 | TTAACTTAAT | 25 | TGTTT |
| 36 | CAATAAT | 25 | CTAATTCCCCA |
| 35 | ACTGTT | 25 | CCTTAA |
| 35 | TAAGTA | 24 | TTCAAAAA |
| 35 | TATAGATA | 24 | ATTTTTTA |
| 35 | TTTTTGT | 24 | TATGAATT |
| 34 | TATTAAAT | 24 | GCG |
| 34 | TCC | 24 | TATATTTTATATCGAAG |
| 34 | TTTTTATA | 24 | CGTCTCATCT |
| 34 | CTCGGATATGTTCGAGAACA | 24 | AGGAAGAA |
| 34 | AATGCAGCAATCCTGCATTAGCTAATAA | 23 | CCTTA |
| 34 | AAACAAAA | 23 | AAAGCGTAT |
| 33 | AATACTAA | 23 | TGTTTTTTTTT |
| 33 | AAATAAAC | 23 | CTTTAACCTTCAACTAGGTCGTTAAC |
| 33 | GTTTGTAT | 23 | AAATTACGAGATATAAGTCG |

| 23 | TATA | 14 | TCGGGTATGTTCGGTAATAC |
| --- | --- | --- | --- |
| 23 | GATATAT | 14 | TTGTCACTTTGAATGACAA |
| 23 | GAATTAGATGTCATGG | 14 | TATAGGAA |
| 22 | GTATGTATAGT | 14 | AAGCCACAGAGCAAC |
| 22 | TTGATTTAAATCAC | 14 | TGATAT |
| 22 | ATTTG | 14 | TTTTAAAA |
| 22 | GGAATTAGATCTCGTG | 14 | ATGTA |
| 21 | TTTCTCTCTC | 14 | GTAATATA |
| 21 | AGAG | 14 | ATATATGTAT |
| 21 | TTACTTTCA | 14 | ATGAAGTTTCT |
| 21 | TTTTAGA | 13 | ATTATATA |
| 21 | AAACCGG | 13 | GTATAATAGTCA |
| 21 | TTAGAATTAT | 13 | ATTATTTATTATA |
| 21 | TTAATTAATTA | 13 | AACATT |
| 20 | AACGTATTCCTCTTGGGA | 13 | AAATTAAT |
| 20 | TATTAAATA | 13 | TATTTAAA |
| 20 | ACAGTAAAATGATC | 13 | AACCTGTCACGTCTTTATTACCGTG |
| 19 | TTAAAGGTCAACGACCTAGTTCAAGG | 13 | ACACGCAC |
| 19 | CCGAACATATCCGAGTATTC | 13 | TTCTTT |
| 19 | TCTCTATCTC | 13 | TAAGAGAT |
| 19 | ATTCCCAAACATATCCGAGC | 13 | ATATACTGA |
| 18 | AAGTATTTAAATACTTTT | 13 | TTTTAAAAATTTTGGAT |
| 18 | AAAAATTCTAAATTTGC | 13 | ATTATATAAT |
| 18 | AATGCAGGATTGCTGCATTCTCTTTTTATCA | 13 | GAAATTTAAA |
| 17 | TATTTCCTGCATCTAAGGAAATCTAAA | 13 | TAATGA |
| 17 | AAAAGTATTTAAATACTAT | 13 | AAATAAAAAAT |
| 17 | AATGCAGGATTGCTGCATTATCTTTTATCA | 13 | AAATTTTGC |
| 17 | TATAATAAAAATAAA | 13 | AATTACGAGATATAAGTCAT |
| 17 | GTTATT | 13 | TAATGGAATT |
| 17 | TTCCA | 12 | TAAAACGAAAG |
| 17 | CATTAAT | 12 | CTGTAACTAGTAA |
| 16 | AATACGTTCCTCAGGAGC | 12 | TTTGTT |
| 16 | TAATATAAAATTAAAATCA | 12 | TTATTTATAT |
| 16 | AATGCAGGAGTGCTGCATTTTATTAGCT | 12 | TATATA |
| 16 | AAAACAGACCTGTAT | 12 | TTTTTAAAATTTTGGA |
| 16 | ACTCT | 12 | CAAATGCAGGAGTGCTGCACTTTATTAG |
| 16 | ATACAAAT | 12 | AATTACGACTTATATCTCAT |
| 16 | CATTAGATAATAAAGTGCAGCACTCCTG | 12 | TTTATTCA |
| 16 | CATTATTC | 12 | TTGAAAAA |
| 16 | TAAATAAATATAATAAA | 12 | TTGAAATT |
| 16 | ATTC | 12 | CTGGTGGTGCTACTTCAATTT |
| 15 | CACTTTGAAGTCGACAATTGT | 12 | AAATATAAT |
| 15 | TTCTCTC | 12 | AATGAAACG |
| 15 | AAATATACA | 12 | GAAAAAATATAAAATGTTCAG |
| 15 | TTTAATA | 12 | TCATAA |
| 15 | TGAGGAACGTATTCCTCC | 12 | AAAATTTTGGATTTTTAA |
| 15 | TATAAAGTA | 12 | TAAATGTA |
| 15 | AGTGAT | 12 | AATACGTTCCTCAAGAGC |
| 15 | CTAAATTAA | 12 | TATAATTAT |
| 15 | TGCAGCAATCCTGCATTAGATAATAAAG | 12 | ATTTCCGTGG |
| 15 | TTTTGAAAATTTTAGAT | 12 | AGATAGAG |
| 14 | ATTTCCTTAGATGCAGGAAATATTAAG | 12 | GCTATTTTC |
| 14 | TAAATAAA | 12 | GTGATAATTTTACT |
| 14 | TATTCGAA | 12 | CTACTACCACTA |
| 14 | TACTG | 12 | AGTAA |
| 14 | TAATTCTG | 12 | AAGTC |
| 14 | TTATAATA | 12 | TTTTAAAATA |
| 14 | TATACTATA | 12 | TTTACTTACTG |
| 14 | ATTTTAAAATTTTGCAA | 11 | ATATAAATAT |

| 11 | CCACCGCCG | 10 | GACAATTGTCACTTTCAATC |
| --- | --- | --- | --- |
| 11 | CTATTTAC | 10 | TAATCGTTA |
| 11 | TATACATAC | 10 | CCTGCATTTGATAAAAAGAAATGCAGCAAT |
| 11 | CCCGTAATGTTTTTCGAAGGTCCGGG | 10 | TCTAGATATTC |
| 11 | TATTTTG | 10 | TCAAAGTGACAATTGTCGAC |
| 11 | TTTTCAGAATTTTCCTGAACA | 10 | TTGAATATA |
| 11 | TAGTTAT | 10 | TAATATAAA |
| 11 | AGAA | 10 | CAAGTGTGAATTCACAGTTA |
| 11 | TTTAAA | 10 | TTATTATTTAT |
| 11 | TCGTATTGATGAGCAGTATCA | 10 | TAAATAG |
| 11 | TGTTGATTAGGTCCT | 10 | ATATATAAAAA |
| 11 | AAATAATATA | 10 | TTTCAC |
| 11 | AATTATTAA | 10 | TGAAC |
| 11 | TTCAAAATCAAA | 10 | TATATACAA |
| 11 | ATATTATTAT | 10 | GAATAACTGAA |
| 11 | ATAATGA | 10 | AAAAAAAAG |
| 11 | AATAGTCAATA | 10 | AATTCAATTCC |
| 11 | ATATTTTATT | 10 | AATTAATTAT |
| 11 | ATCCCATACCC | 10 | TAGTTGTCAGTTCTGCTA |
| 11 | ATATATAATATA | 10 | AATATGAAA |
| 11 | AAATTATG | 10 | GAAG |
| 11 | AATTTG | 10 | ATTTTTA |
| 11 | AGATTTA | 10 | ACTCGGGTATGTTCGGGTAT |
| 11 | TCTATATTC | 10 | TATAGAA |
| 11 | GATTTCTGC | 10 | AAATTCCGAGTTTAAAGTCGAAAAAAATA |
| 11 | TAATTC | 10 | ATTTTATAC |
| 11 | AGAATATAAGGTATATCATCGT | 10 | TTGTATTGTTA |
| 11 | AAAAAAAAAAAT | 10 | AATTGTATTTCAGAAAATGTC |
| 11 | TTATTTTTGAAAT | 10 | TAAATAATAT |
| 11 | TTTAAAATTTGCAAAT | 10 | TATTTATATTA |
| 11 | GACTATATA | 10 | TATTGATCT |
| 11 | TATTCTATTTC | 10 | TTGAATA |
| 11 | TTTAATTA |
| 11 | CTAATTCCCA |
| 10 | ACATCTACAT |
| 10 | TTTTACA |
| 10 | AATTAGTGAGG |
| 10 | GGTATATAGGA |
| 10 | TAACCTTCAACAAGGTCGTTAACCTT |
| 10 | TTATATC |
| 10 | AATGTAC |
| 10 | ACTAAGATACTCTAAAAGTA |
| 10 | AATTTATTTTA |
| 10 | GCATAAAGCGTAA |
| 10 | CATATTTAA |
| 10 | ATAACG |
| 10 | TAACTTTTTG |
| 10 | TGTATT |
| 10 | TATATTAA |
| 10 | ATTTTTCAAAAATCTAA |
| 10 | ATAGAA |
| 10 | TTTGTTTTT |
| 10 | TCAATATA |
| 10 | TATGTGTATG |
| 10 | ATATCAAAT |
| 10 | TACTTT |
| 10 | CACACATA |
| 10 | TAATTATTAA |
| 10 | GAAAAATAGC |

| **Anoplophora glabripennis (#SRR941723)** | | | |
| --- | --- | --- | --- |
| 686037 | GTAGCA | 508 | TTTTAGTA |
| 174469 | TTG | 505 | GTTCTT |
| 24884 | GACAAC | 501 | AGACACACAC |
| 24638 | ACT | 495 | CTGCTACTGCTACTCCTACTGCTA |
| 20217 | CTGTTGTTGCAGCTGTTG | 494 | TGATAA |
| 19535 | TAACAACAACAACAG | 483 | ACGTGACGTAAA |
| 10839 | AACAACAGC | 477 | TTTTACCCGGAG |
| 7894 | TGA | 470 | ATCATCTACATCACC |
| 7251 | ACAGCA | 443 | GTTGTTACTGGTGTT |
| 6693 | AACAACAGCAACAGCAGC | 435 | TAACAACAG |
| 6134 | CAACAACAACAGCTG | 433 | CTGCTC |
| 4184 | GCTT | 420 | GAA |
| 4037 | GTAG | 414 | GCTGTTGCTGTTGTTA |
| 3945 | AACAAC | 396 | CGTTAGGTTTGATATTATTTA |
| 3684 | GTC | 394 | TAGTTGAC |
| 3667 | TAACAACAACAACAGC | 378 | TTGAGTGAAAT |
| 3185 | CAACAACAACAGCTGCAA | 368 | AGTGGTAGT |
| 2919 | CATCAACAT | 357 | CTGTTGTTGTTGTTGTTA |
| 2381 | TTGAAAAG | 356 | ATCACCATC |
| 2335 | TCATCAGCA | 347 | GTAGTG |
| 2136 | TGGTGT | 346 | GTGAGGACAA |
| 2051 | TGTTGCTGTTGT | 329 | CAACCTGTTTTTTACTGCTA |
| 1844 | CAACTTGT | 329 | GACCTTGACCCCAGGT |
| 1771 | AGTAGG | 321 | TGTG |
| 1654 | TGAAGATGA | 310 | TCATCGTCA |
| 1600 | ACCATC | 299 | CAACAACAACAACTG |
| 1585 | GATGTT | 286 | GGT |
| 1578 | TGTTGTTGTTGCACTGT | 283 | AACTTGAC |
| 1426 | GATGAC | 281 | GTGTGTCTGTGT |
| 1330 | ACAACAACAACAGCT | 277 | GATGAT |
| 1322 | ATCAGCATCATC | 276 | GGTTATGG |
| 1255 | TGGA | 270 | CAACAACAACAACCG |
| 1254 | AGCATC | 267 | GTCATCCCAAG |
| 1142 | GTTGTTGTC | 266 | CAGCAACAGCAG |
| 1100 | CCTGTTTTTTACTGCTACAGA | 262 | **CCACGA** |
| 1035 | CTAAC | 262 | AGACTGGATAATCATCACGTAA |
| 1034 | TTGTTAGCCGG | 261 | AGTAAC |
| 1016 | TTAAAAGT | 261 | CAACAACAACACCAG |
| 1005 | AGAT | 260 | ATTGTCGAC |
| 964 | TGTTGTTGCTGCTGTTGT | 260 | ACGACAACG |
| 915 | CAACCA | 259 | TTTTG |
| 852 | TGC | 257 | TGATTA |
| 839 | GTCTGT | 255 | TGGATAATCATCACGTAAGAC |
| 802 | AAACTCCGGGTAA | 254 | GACCTTGGAT |
| 754 | TGTCTGTG | 253 | TGTAAAAAACAGGTC |
| 750 | CCGGCAAAAATACCGGACT | 252 | CGCACACA |
| 706 | AAGT | 247 | ACACAC |
| 684 | GGTTGTC | 239 | ACTACCACC |
| 680 | TTACGTGATGATTATCCAGTT | 239 | CGTCGT |
| 651 | ATAA | 237 | GTGC |
| 637 | AGTAGCAGTAGGAGTAGC | 229 | TCATCT |
| 609 | TATG | 229 | TATCATCAT |
| 592 | CAACAACAACAACAG | 228 | TTGTTGTTGTCG |
| 573 | AAAAAACAGGTTGCAGGCTGT | 226 | ATTTACGTTAGGTTAGCTTTCA |
| 572 | TAA | 226 | GTTGCC |
| 553 | TGTC | 223 | AACAACAACAGT |
| 518 | TTATTG | 223 | GTAGTA |
| 515 | CAACAACAA | 222 | ACAAGG |

| 222 | AACAACAACAACAGAGC | 117 | TCAAATTTCAAAACAAAGGTG |
| --- | --- | --- | --- |
| 220 | CATCATCCAT | 116 | TCC |
| 218 | CTATTA | 116 | AACCTAACGTAAAAAATATCA |
| 218 | TTTTTGCCGGAGTCCGGTT | 116 | TGTAGATGATGGCGA |
| 218 | CGGCTACCAAC | 113 | AGTTGACG |
| 206 | AACAACACCAACAGCAGC | 110 | CCCGGAGTTTC |
| 203 | ATGA | 110 | CTGGTG |
| 201 | TAGCCTGTCAGCCAGTTGGT | 108 | CTTGGGGTTAATGAGTTGACTCG |
| 199 | TCATCATCAA | 108 | TGACCTTCATA |
| 197 | GTCAAATTACAAAACACGGT | 108 | AAAACAGGTTGCAGGCAGTAA |
| 195 | CTACTGCTACTCCTACTCCTACTGCTACTG | 108 | TTGTTCTTG |
| 192 | GTTTTACGTCACGTCGTCAC | 107 | CTATAC |
| 191 | TTTG | 105 | TTTAG |
| 187 | TAATGG | 104 | TCATTG |
| 186 | CATGACCTTGA | 104 | GGTGACAA |
| 186 | GTAGGAGTAGCA | 101 | TGTGCGTGTGTG |
| 180 | AACCTGTAGAAAACAGGTCTG | 101 | AACAACAACAGTAC |
| 178 | AACAACAACAACAGCATC | 100 | TCCTA |
| 177 | CATCAACAACAT | 100 | GAAGAGTT |
| 174 | ATACCA | 99 | AAAAACAGGTCTGTAGCAGT |
| 171 | AGTAGGAGTAGGAGTAGC | 99 | AACAACAACACC |
| 170 | TTTGTGGG | 98 | AAAACAGGTTACAGGCTGTAA |
| 168 | TGAATTGAATT | 98 | CTGTTGTTGATGCTGTTG |
| 164 | TGTTGCTGTAGT | 97 | AGGTA |
| 158 | GCTACC | 97 | TGTGTGTGTGGG |
| 158 | AGGATG | 96 | ACCTGTTTTTTACTGCTGCA |
| 156 | GTAACCGTCACGGGTATCCATGG | 95 | AACGTC |
| 153 | AGCATCACCATC | 95 | TGAATGACCT |
| 153 | TCGAGAAA | 94 | AAACTCCGGGCAA |
| 148 | ATCGAC | 93 | AGTAGCAGTAGCAGAAGC |
| 146 | CACAGAGA | 92 | GTGTGTGCGT |
| 146 | CAGTAGCAGTAGCAGTAGCAGTAGGAGTAG | 92 | AATCATCTT |
| 145 | AATAA | 92 | TGTTCGTGCGTG |
| 145 | AATAG | 89 | TGGACAAGGACAA |
| 143 | TGCTGCTGTTGTTGT | 89 | TGCTACTGCTCC |
| 142 | AACAACAGCAACAGCTAC | 88 | ACTAAAACTACT |
| 141 | TCCGGGCAAAAC | 87 | CTTGCTTA |
| 141 | AAACCCCAAACCCAC | 87 | TGTGGG |
| 138 | ATTGAGTGAA | 87 | AGCCGTTGTT |
| 137 | GTAAAAATCCGG | 87 | TTGATGCAGCTGTTGTTG |
| 137 | CAACAACAC | 86 | ACAAAA |
| 135 | AGGAGTAGT | 86 | AATT |
| 134 | CACTACTACCAC | 85 | AAAAAATA |
| 131 | TGTTGTT | 85 | GACGAGGACGATGAT |
| 131 | CCCAATAA | 85 | AGTAGTAGTTGT |
| 131 | CTGCTACTCCTACTCCTACTGCTA | 85 | GTTGTTATT |
| 130 | GATGATGATAAT | 84 | TCAA |
| 128 | GTGGATAGTAAT | 84 | GGTGAC |
| 127 | TATTTTAT | 83 | ACTACG |
| 126 | GCGACA | 82 | GTTGTTACTGCTGTT |
| 126 | GACGATGAC | 82 | AAAAAACAGGTTGTAGGCTGT |
| 125 | CACACG | 81 | GCCACT |
| 123 | GAATAGAATAGTTCAGCCTACTCTTTAAACAAA | 81 | CTGAACCTGTAGAAAACAAGT |
| 122 | ATCATCA | 80 | GTTGTTGTTGCTGTTGCT |
| 119 | TGGTGATGG | 79 | CTACAGACCTGTTTTTTACAG |
| 119 | CTGC | 79 | CAAATCTACATTACTATCCA |
| 118 | CCCACCTAGGGCTTTGC | 79 | TGTGTGTGTGTGTC |
| 118 | CTACTGCTA | 79 | TAGTAGTAA |
| 118 | GCTACG | 79 | GATAAC |

| 78 | CTGTCTAT | 56 | AACGACAAG |
| --- | --- | --- | --- |
| 78 | TTGTCTACA | 56 | TAGCAGTAGCAG |
| 78 | TACTGCCTACAACCTGTTTTT | 56 | TTCAAATAACCGATTA |
| 77 | CTGTTGCTGCTGCTGTTG | 55 | AAAAGTCCGGGG |
| 77 | TGGTGG | 54 | GTTGTTGTTGTCACT |
| 77 | GCTGTTGCTGTTGTTGTCA | 53 | CCATCAT |
| 77 | AAACTCCGGGGAA | 52 | AGAAGATTTACAAAAATT |
| 77 | CAACAGCAG | 52 | CCCCATCGTCAC |
| 76 | TCACCC | 52 | TTATCC |
| 76 | GACCTTGGGT | 52 | TACTGTTGCTGTTGT |
| 75 | TGTAATTTGACACCATGTTT | 51 | TATTAG |
| 73 | GAATAA | 50 | TAAAAAACAGGTCTGCAGCAG |
| 73 | TTTTGCA | 50 | ATTTTTGCCGGTCCGGT |
| 73 | CAACAACAACAGTAG | 50 | AATTC |
| 73 | TTTCTACTGGTTCAGACCTGT | 50 | ACAAACTCCAAGCCC |
| 73 | CTGTTGTTGTGCTGTTG | 50 | AGTTGTAA |
| 73 | CAAGTTGACGA | 50 | TTTGCCGGAGTCCGGTAAT |
| 73 | GCCAAC | 50 | CCACACAC |
| 73 | CAGTAGCAGGAGCAGTAG | 50 | GTTGGTT |
| 72 | TGATTATCCAGTTTTTACGTGA | 50 | CCTGA |
| 72 | AGATTACGT | 50 | TCCTTAAATTTAGCCTG |
| 72 | ATGGCG | 49 | GTCTTTTTCTATCTTCTTCTT |
| 72 | CCCGGAGTTTT | 49 | AGGAAT |
| 71 | ACAACAATAATA | 49 | GGAC |
| 70 | ACCTGTTTTTT | 49 | CAGGCTGTAGAAAACAGGTTG |
| 69 | TGATAG | 49 | CGTAAGACTGGATAATCTTCA |
| 69 | ACCTGTTTTTTACAGCTGCA | 49 | TGTTGTTGCAGCTGTTGA |
| 67 | AAGTCCGGGGAAA | 48 | TGTGTGTGGGGTG |
| 67 | GTGGACCTG | 48 | AGATT |
| 66 | CTCGTC | 48 | TGTTTAGAT |
| 66 | AAACTCCGGGAA | 48 | TGGGGTCAAGGTCACA |
| 66 | ACGGTTATCCAGTTTCACGTG | 48 | CTACTACTA |
| 66 | TACTAGCTGATTAC | 48 | CAATA |
| 65 | CAACAGCAACAACAG | 47 | CTACTCCTC |
| 65 | ATCATCATC | 47 | AACAACAACAACAGCTAGC |
| 65 | TCACGTGAAACTGGATAATCA | 47 | TTATTATTG |
| 64 | TAAC | 46 | CCCACAAACCCCAAG |
| 64 | ATGGATAG | 46 | AGCAACAGCTAC |
| 63 | TAGCCTGTTGGTAGTCTGTATAG | 46 | GTCACGGGTATCCATGGATAACC |
| 62 | TTTGAA | 45 | TATAAATAAATAATTAT |
| 62 | CACAGACAGACA | 45 | CACCGA |
| 62 | CACACACA | 45 | CGTCACGTTTA |
| 61 | CCCAGAGAGGCCATTAGATGT | 44 | ATGTAATTT |
| 61 | GATGATGATGAC | 44 | TGTGTGTTTG |
| 60 | GATGACGACGAT | 44 | GGGAAAACTCCG |
| 60 | ATGATC | 43 | TGATGATTA |
| 60 | TGTTGTTGCTGCTGTTGTTGT | 43 | ATGACGACGACG |
| 60 | ATGATCATC | 43 | GACCTGTTTTTTACAGCCTGCA |
| 59 | TGATGAGGA | 42 | GAGGAACGGTTCCG |
| 58 | TACAGACCTGTTTTTTACAGCC | 42 | TGTGTGTTTGTGTTT |
| 58 | TCCGGGAAAAAAACCGGAC | 41 | AGCAGA |
| 58 | TGTGTGTT | 41 | ACGATGATC |
| 57 | ACTTCACTT | 41 | TCAAGGTCATC |
| 57 | GTAAAACTGGATAATCGTCAC | 41 | TTCAACAT |
| 57 | TGCTAT | 40 | GACTCGTTTGGGGTTAATGAGTT |
| 57 | AGTAGTAGTAGC | 40 | TGTGGATAGTAATGTGGATT |
| 57 | CAAGTGAGGACAACATTGAGGA | 40 | GAAA |
| 57 | CACACGCACGCA | 40 | CTGTTGCTGTTGTTGTTA |
| 56 | ATACTG | 39 | TGTTAGCCGGTTGTTAGCCGT |

| 39 | TCTGAATTCAGAGTTTAAAG | 30 | GGGTTAAG |
| --- | --- | --- | --- |
| 39 | TAGGCGTCGG | 30 | ACCTGTTTTTACAGCCTGCA |
| 39 | GTAGA | 30 | CACACACACA |
| 39 | CATTATTAT | 29 | TGATTGATTTATGACTTTCACGACTGG |
| 39 | CGTCACGCGAAACTGGATAAC | 29 | AAACTCCGGGTAAA |
| 39 | TGTTGTTGCTGTTGTTGT | 29 | CTGTTGCTGTTG |
| 38 | TTAAA | 28 | CACCCTCACCAT |
| 38 | TTACGTGATGATTATCCAGT | 28 | AACCGGCTAACAACCGGCTAAC |
| 37 | AGAACG | 28 | TGCCTGCT |
| 37 | AGAAA | 27 | GTAAAAAACAGGTCTGTAACA |
| 37 | GGCGGCG | 27 | TGTGGGTGTG |
| 37 | GGTCAAGGTCACCTGA | 27 | ACTG |
| 37 | TGATGTTGT | 27 | AGAGAT |
| 37 | AGTAGTAGTAGG | 27 | ATTATTTATTA |
| 36 | TTATCTGTAATCTACGTAATC | 27 | TGTGTGTATGTG |
| 36 | TTTTTGCCGGAGTCCGGTTT | 27 | AATTAATGTT |
| 36 | AATTTAAAGCTAACCTAACGTA | 27 | TTGCTGTTGTTGTTGTTGCTG |
| 36 | AGATTACAT | 27 | AACAGGCTACCA |
| 35 | AACCTAACGTAAATAATATA | 27 | ATTTG |
| 35 | CTACAA | 26 | AAAATAAAAAAAT |
| 35 | ACAACGACAACGACAACA | 26 | GTAAAAAACAGGGTACAGGCT |
| 35 | AGGTAGAT | 26 | ACAACAACATCA |
| 35 | TGTGTTTT | 26 | CACAAACCCCAATCC |
| 35 | CAGACCTGTTTTTTACTGCCTA | 26 | TGTTGCTGCTGT |
| 35 | TTCTG | 25 | GCGCACAC |
| 35 | TGTAATCTATC | 25 | AAGTCCGGGAAA |
| 34 | AGTAGCGGT | 25 | TCTTC |
| 34 | TTATAGGG | 25 | TTGTTGCTGCTGCTGTTG |
| 34 | GTTGTTGTTGTTGTT | 25 | TTATCCCCAA |
| 34 | AACAACACCAACAGCTGC | 25 | CTGTTGGTGTTGTTGTTA |
| 34 | CGCCCCAATGGCACCAGT | 25 | GGTCAA |
| 33 | TTGTCCTCAT | 25 | CCGCACG |
| 33 | CTATA | 25 | ATTTACTAAT |
| 33 | GTCGTGAAAGATAAGTCATTCACCA | 24 | AAAACAC |
| 33 | GAGATCCGTTGATCTCATCGTATTA | 24 | GGTTGAAAATTT |
| 33 | CTGTCTATCTGT | 24 | AGGTAGGG |
| 33 | AAACTAAAACT | 24 | ACACATAC |
| 33 | ATAGTTGA | 24 | GTGTA |
| 32 | ACTTTACTTAA | 24 | TTGTTGGTGGTG |
| 32 | TTAGGTTAGCTCACATTTTACG | 24 | TTGTTGTGCTGCTGTTGCTG |
| 32 | TTGTTCTTGTTG | 24 | AGTCT |
| 32 | TTTTTACAGCCTGAACCTAT | 24 | GCAGTAGCAGTAGCAGTAGCAGTG |
| 32 | CGGCAT | 24 | TTGTTTTTG |
| 32 | CAATTATCCCC | 24 | TGTTGTGCGTG |
| 31 | CAAGCAAGTAAG | 24 | TCTTAC |
| 31 | AAAAAATACGTGC | 23 | AAAAATAAAA |
| 31 | CCGGAGTTTAC | 23 | TGTTGTGGT |
| 31 | TTGTCCA | 23 | ATCTACAGCACCATC |
| 31 | CTGCTACTGTTACTGCTA | 23 | TGGTGATGGTGG |
| 31 | GTTACTTTGGAACC | 23 | CAGTAAAAAACAGGTTACAGG |
| 31 | GATGGTGATGAT | 23 | TGTTGTTGTTAT |
| 30 | GGATCGTTGTCCTTT | 23 | AACAACAACAACAGCTAAC |
| 30 | GTCGGCGTCCTTT | 23 | TGCTACTGTTGCTGTTGT |
| 30 | TAGAAG | 23 | GATGTAGATGATAGC |
| 30 | TTTCAACC | 23 | AGGGGGATAATTGTCAATGAA |
| 30 | TGCTAATCCTACTCCTACTGCTACTGCTAC | 23 | CGCATT |
| 30 | CAGCAG | 23 | TTTTACCCGGAC |
| 30 | TAGCTGTAAAAAACAGGTTG | 23 | ATACAA |
| 30 | GGTGTCAAATTACAAAACAAA | 22 | GTTGTTATTGCTGTTGCT |

| 22 | GGTTAAATGTCCCGATATTGTTGAAGTG | 16 | CGACGACGA |
| --- | --- | --- | --- |
| 22 | CTCCTACTCCTACTGCTACTCCTA | 16 | AGGCTACCAAC |
| 22 | CATTTGTGTTATGGTGAAG | 16 | CGGTGC |
| 22 | AGAGTTGCCAA | 15 | TGTTTTTTT |
| 22 | TTTGAAATTTGACACCGTGT | 15 | TGTTGTTGCTGCTGTTGA |
| 22 | TTAATTATTATATTCTA | 15 | ACCTGTTTTCTACAGCCTCGA |
| 22 | AGTAGCAGTAGCGGTAGC | 15 | ACTGTTGTTGCTGTT |
| 22 | AGTAGCAGC | 15 | TTGTCGTTGTCGTTTTAGTTGTCG |
| 22 | ATTTACGTTAGGTTAGATTTCA | 15 | GCAGTAGCAGGAGCAGTAGCAGTA |
| 22 | AACAACAACAACAGCTGCAGC | 15 | CAACATCATCAT |
| 22 | AAGTAAGTAAGC | 15 | TTAGTTAGCTCACATTTTACG |
| 21 | TAGTAGTAC | 15 | CGAACAAA |
| 21 | ATTTTTT | 15 | GTGTGTGTAT |
| 21 | TGTTGTTGTTTT | 15 | TGTTGTGCTGTTGTTGTTGT |
| 21 | ACTCCGGGTAAAAAATCCGGGAAA | 15 | TAGCAGAAGCAG |
| 21 | CAGTAGCAGGAGCAGGAG | 15 | CAAGGTCACCTAGGGT |
| 21 | TTGTCCTCAATG | 15 | TTTATG |
| 21 | GTTATCCAGTTTTACGTCACT | 15 | CTGTTGTGCTGCTGTTG |
| 21 | GCTGCTACTGTTGTT | 14 | TAAAACTG |
| 21 | TCCAGTTTTACGTGACGGTTA | 14 | CACACCACACACA |
| 21 | TTGGTGCTG | 14 | TGTTGTTGCTGCTGTTGCTGT |
| 21 | ACCTGTTTAAA | 14 | ACAATAA |
| 21 | CCATCATCATCG | 14 | CCTGCACAGGTCTA |
| 21 | AACAACAACAACAGCTAC | 14 | AAGCAAGC |
| 21 | AAAACATGCTATCTCAAATTTCGGTTGG | 14 | TTGCTGCAGCTGTTGCTGTTG |
| 21 | TTTTTACTGTCTGCAACCTGT | 14 | AAAAATCCGGGTA |
| 21 | GTTGTTGTACTGTTGCT | 14 | TGTTGTACTGCTGTTGCTGT |
| 20 | AACAACAACAACAGTAGC | 14 | AGCAGTAGCAGC |
| 20 | TGATAGA | 14 | ATTTAC |
| 20 | GTAGCAGTAGCAGTAGTAGTAGTA | 14 | AAACCCACAAACCCCAAACCCAC |
| 20 | CATTA | 14 | ATAATAACG |
| 20 | AAATTTTACTC | 14 | ACAAAC |
| 19 | AACAACAACGACAACAAC | 14 | GTTACGGG |
| 19 | ACCTGTTTTTTACAGCCTGT | 13 | CTACAGACCTGTTTTTTTACTG |
| 19 | CAATCAGAGAGCCGGGAATTCTAATCCGTC | 13 | TTTGTTTTAT |
| 19 | ATGTTTTCCAACTGAAATTTGAGATAGC | 13 | CAAAACTCCGGGT |
| 19 | TGAGGACAACAG | 13 | GTTGTTGTTGAGCTGTT |
| 19 | AGTGTGTGTG | 13 | CGGACAGGCTAAATTTA |
| 19 | TTTTACGTTAGGTTAGCTACA | 13 | GTTAGCCGGTTG |
| 18 | ATATCAATGATAT | 13 | ACGGAAAACGTGACGACGTG |
| 18 | ACTTCCTCCAA | 13 | CACCGC |
| 18 | CCAGGTCCACTAGGTCCA | 13 | AAAGCCAA |
| 18 | ATTATCCAGTTTCACGTGACG | 13 | GTTGTTGTTGCCAGCTGTT |
| 17 | TCCTACTGCTACTGCTACTGCTACTGCTAA | 13 | CAATTGGATTTC |
| 17 | AACAACAACAACAGCTGCAAC | 13 | TTATTCGAATAACCGG |
| 17 | CTGTTGTTGTTAGCTGTTG | 13 | ACGGATGG |
| 17 | CGTCGC | 13 | TTTTACCCGGAA |
| 17 | AACAACTGC | 13 | GGATCTTCT |
| 17 | TTTGTGTGTGTTTGTGT | 13 | TGTTGTTGCTGCTGCTGC |
| 16 | TCAC | 13 | CATAGCGAA |
| 16 | GTTCCT | 13 | TGTTTTCTACAGGTTCAGACA |
| 16 | AATGACCTTGA | 13 | GACC |
| 16 | CGTGACGGAAAA | 13 | GTCGTTGTCGTC |
| 16 | GGTTTGTGGGGTTGG | 13 | TGCTGTTGCTGTTGC |
| 16 | ATTGAGTAAA | 13 | GTGTGAGT |
| 16 | GACCTTGACCCAGGT | 13 | GCTGTAGAAAACAGGTTCCAG |
| 16 | ATTCTTTGTTTAAAGAATAGGCTGAACTATTCT | 13 | TGAAATGAA |
| 16 | TCTATCTTCT | 13 | GGTGGG |
| 16 | TTTACGTTAGGTTAGCTTTAA | 13 | AATTTTATCTGTAATCTATGT |

| 12 | ATGTGGATACTA | 11 | GAAAAAGGATAACCGTCACGC |
| --- | --- | --- | --- |
| 12 | CACACACCACA | 11 | AAGTA |
| 12 | GCTACCTA | 10 | GCAGCGGGGGGCG |
| 12 | GATGACGAG | 10 | TACCCA |
| 12 | CTACTCCTACTC | 10 | CATAAATCAATCACCAGTCGTGAAAAT |
| 12 | ATGATCATCATG | 10 | CTGTTGTTGCTAGCTGTTG |
| 12 | CTGTTGATGCTGCTGTTG | 10 | AGGAGA |
| 12 | AGACCA | 10 | GGTGTCAAATTACAAAACAA |
| 12 | AAAAAACCGGACTCCGGC | 10 | **GTTGGG** |
| 12 | TGATGACGATGACGA | 10 | AAGATTTAAATC |
| 12 | CTTTGCCCCCACCTAGGG | 10 | CTATCCGT |
| 12 | ATTAGAATAAGCTTAATA | 10 | AAGCCATCC |
| 12 | TGTTGCAGCTGTTGTGT | 10 | TATCATGATATA |
| 12 | TTCAAATGACC | 10 | GATGATCATGAC |
| 12 | TGAGTG | 10 | TCTCTAATACGACGAGATCAACGGA |
| 12 | AGGAAGTCTGG | 10 | CTACTGCTACTGCTAATG |
| 12 | CACCAACAC | 10 | TTTTACTGCTACAACCTGT |
| 12 | AATACAAAATAA | 10 | CCGAGTTTAAAGTCAGAATT |
| 12 | CTCTAC | 10 | ATCGTCAGC |
| 12 | CCTACAAACTCCAAG | 10 | ACGTGACGTGAA |
| 12 | CTGACTTTAAACTCAGAATT | 10 | ACAACAACAACAGCTGC |
| 12 | CAGTAAAAAACAGGTCTGTG | 10 | ACCTGTTTTTATAGCCTGCA |
| 12 | AACGACAACGACAACTAA | 10 | GTAAAAAAACAGGTTGCAGGCT |
| 12 | ACCTGTTTTTTACAGCCTGAA | 10 | GCTGTTGATGTTGTTA |
| 12 | CCTAACATGA | 10 | AAACTGGATAATCATCACATA |
| 12 | GTGTCGGT | 10 | GTCACTTGTCTACTCAGAGGCAGTATCGGCCTCA |
| 12 | TCAGACCTGTTTCCTACAGGT | 10 | TTTTTTACTGCTACATACCTG |
| 12 | TATCCCCCTTTCATCGACAAT | 10 | TGAAACGTGACGACGTGACG |
| 11 | CGACGAGGACGA | 10 | GATGGAGATGTAGAT |
| 11 | CATGGATAACCGTCGGGTATC | 10 | CGTAGCAGTAGG |
| 11 | ACTACTACAATT | 10 | CTTCTTCTAATTTTTGTAAATCTTCTTTTCTAC |
| 11 | AACCTAACATAAAATGTGAGCT | 10 | GGACCTGGTGGACCTGGAGC |
| 11 | AAGG | 10 | GATTTTTTTGATTTAAATCA |
| 11 | CAACAACAACAGTGCAG | 10 | TGTGCGTGCG |
| 11 | TAATTTTA | 10 | CAGTTTACGTGACGATTATC |
| 11 | ACAGG | 10 | GAGTGAGGGAAAT |
| 11 | CTACTGCTACTGCTAATGCTACTC | 10 | GAATGA |
| 11 | TTTTTACTGACAGACCTG | 10 | TACTGCCTGAAACCTGTTTTT |
| 11 | TGTTGTTGTGCTACTGG | 10 | CTTGCTTGCTGG |
| 11 | GTTTATGG | 10 | CGGGTAAAAACTCCGGGAAAACTC |
| 11 | ATAAACCCTAAGCCC | 10 | TTCCAACTAAATTTGAGATAGCATGTT |
| 11 | TAGCGGTAGCGGTAGCAG | 10 | TTGTTGTTGTTTTTG |
| 11 | CCTGTAGAAAACAAGTCTGGA | 10 | AACGTGAGGCTCGAAAA |
| 11 | AATGAGGACAAGTGAGGACAAC | 10 | AACAACAGCAACAGCTACAAC |
| 11 | CCTAGGTGGGGCAAAGA | 10 | AAATAA |
| 11 | CGGACCGGCAAAATTAC | 10 | AACAACAGCAGCAGCTGC |
| 11 | TGTTGTTGTTATTGC | 10 | ACCTGGGAACGCGTGCGTTCTGTGGTGTCGCGT |
| 11 | GTCAACTG | 10 | TGTTGTTCTGTTGT |
| 11 | GTAGCAGCAGCAGTAGCA | 10 | CAACAACAGGAA |
| 11 | CTGCTACCA | 10 | CCTATC |
| 11 | GTTGTTGTTGTCGTA | 10 | CAACACAACAGCAGCAA |
| 11 | AAAACACACACAAACAC | 10 | TGTTGTCACTGTTGTTGT |
| 11 | TTTTTTATT | 10 | GGCTACCAAACAGGCTAACCAACCGGCTAACA |
| 11 | GAACCTCGATAATCC | 10 | CGTCACGTTTT |
| 11 | AGTAGCAGTAGAAGTAGC |
| 11 | ATCCACATCGTCGCCTTC |
| 11 | TATATTG |
| 11 | ACCCGGAGTTTC |
| 11 | ACACAAAACACG |

**Chrysomeloidea, Chrysomelidae**

| **Callosobruchus chinensis (#SRR949786)** | | | |
| --- | --- | --- | --- |
| 63032 | TTTTACGATTTTGGA | 167 | AGAGAAAGAG |
| 3994 | ATCGAAA | 166 | CAATTCATGA |
| 3352 | AGAT | 163 | TTCCTAGT |
| 2805 | **GGTTA** | 160 | TTCA |
| 2297 | CTTTCGA | 154 | AAGGTCG |
| 1818 | AAT | 147 | TCACGGTCGG |
| 1678 | TATT | 145 | AATCTTTAG |
| 1471 | ATAC | 136 | ATTGT |
| 1353 | AAAATCGTAAAATCT | 132 | TTAGAGATT |
| 1310 | ACAGAAA | 129 | CAGAAGA |
| 925 | ATTTTCT | 128 | GGATGGAC |
| 911 | AAACCAG | 127 | GAATTGTCAG |
| 857 | GAAAATCTT | 124 | AATTTTCCA |
| 739 | GTTTGGCGCAACCTAATTTC | 124 | GATTTTTC |
| 738 | TTCTGTTGTT | 123 | CATTATTGTATTCGA |
| 720 | TTTCTGGTTTCTGT | 115 | AGTCGTGGCTTGAAGAAGA |
| 530 | ACT | 114 | TAGAA |
| 515 | AAATTTTGA | 113 | AATTCAAGGC |
| 489 | GAAAGGAA | 112 | TCTGTCTC |
| 484 | TCTC | 110 | TATACAC |
| 481 | TTGGCGCCAAACGAAATTAGG | 109 | AAATCGTAAAATCCG |
| 467 | CAAACGTTTG | 108 | GCCCAGTAAAAGATGTTCTATG |
| 446 | TCCATTT | 107 | CAAAT |
| 437 | GATTTTAC | 107 | AGG |
| 406 | GATG | 107 | ATTTTACA |
| 405 | AGGATTTTT | 106 | AGTAATAA |
| 402 | CATTTGCAAA | 105 | TGTTAGCCGTTTGCTAACCGTTTTTATG |
| 401 | AAAATCC | 104 | AACGAAGTTAGGTTGGCGCCA |
| 378 | GAGACA | 103 | ATCTTCT |
| 363 | TTTTACTGGGCCATAGAAACC | 102 | AAAAATCGTGCCGGTTTTTTCCGTACGGCC |
| 329 | ATC | 102 | TTGTAAATT |
| 314 | AATTTTCTA | 101 | AGAAGG |
| 312 | ACTATAG | 97 | TTTCGTC |
| 304 | CAGAAAATT | 96 | TGTC |
| 303 | CATGAC | 95 | AATTTTTGA |
| 302 | AGAGAG | 93 | CAATTTT |
| 300 | AAATTCTGA | 93 | CGCGCCGAGTGTGTGCGCT |
| 283 | TCGTCATTATTGTCT | 90 | TTCTGCT |
| 255 | GGCAATTCAT | 90 | CTCTTCTCT |
| 244 | TTTTCTAGA | 89 | TGT |
| 242 | ATTTTGAGA | 89 | ACCAACTTTCATTCATTCCAAACTGTGCGATAAAC |
| 237 | TTC | 89 | GGACTTT |
| 234 | TTTCTCTC | 87 | TTCTTCT |
| 233 | TGTCGACAA | 87 | CACTCGGCGCGAACGCACA |
| 231 | GTCAC | 87 | AAAGATCCT |
| 225 | CTTGAGAAT | 87 | CTTTTCT |
| 224 | AATCTTCAA | 86 | TTCATTTGGCGCAACCTAAT |
| 224 | TTTCCAAAA | 81 | ACCAACTTTCATTCATTCCAAACTGTGCGATAACA |
| 219 | AATCTGGAA | 81 | TCAAAACTT |
| 213 | GTCAG | 80 | TTAC |
| 212 | GCCCAGTAAAAAGGTTTCTATG | 80 | GTCGAACGCCGGAGAGAGAAGAGG |
| 203 | AAAG | 79 | TATCCATT |
| 194 | CTTGAAATT | 79 | GAATTTTTG |
| 194 | AAGG | 78 | GAAAAATG |
| 179 | GACTTCTTCTTCTAGCCAC | 74 | ATCCTAAGA |
| 176 | CTTTCT | 74 | TTTCAAAGA |
| 172 | GTTTTCC | 72 | AATCTTGGA |
| 172 | GTTCTCT | 72 | ATTTTCGAG |
| 71 | ATTCA | 39 | AAAATTGG |
| 70 | AGACAGATAGAC | 39 | TTAATCAA |
| 70 | ATAGAGAG | 38 | CTCGATTAACCGAGT |
| 70 | TGAAAATTG | 38 | TGTCACCATACCTTGTCATTT |
| 70 | GAATGAAAGTTGGTTGTTATCGCACAGTTAGCAAT | 38 | ATGACG |
| 70 | GAAAAG | 38 | **CTAGC** |
| 69 | TGTAGGACACACAGGTGTCAGATCTCGGAATTTTC | 37 | CGTACGATTTTTTGCCGTACGGTAAAAAC |
| 68 | ACAACAAAAA | 37 | GAAGGCAA |
| 67 | ATTTTAG | 37 | ACAATTCAAGGCAATTCATG |
| 66 | GCAAACGGCTAACATATAAAAACGGTTA | 36 | TCCGACGACGA |
| 66 | TCTAAATTC | 36 | AAATCTTGT |
| 66 | CGTCTTTACGTCTTTTTCTCAGCGAAATGCCTCA | 36 | CTATATC |
| 66 | TAGCCCAGTAAAAGGTTTCTA | 35 | ATTTATTATT |
| 64 | CTAAGACTATAGA | 35 | GGCAATTCCA |
| 63 | GGAAAGAA | 35 | AGAGCGAGAGAG |
| 62 | AAACAATTC | 35 | AAAAGGG |
| 61 | TCGACTA | 35 | ATAAACGA |
| 61 | TGGATTTT | 34 | TATCGTATCGT |
| 61 | GGTTTCTC | 34 | GCCCAGGAAAAAAGTCTATG |
| 61 | ATATACAT | 34 | ATGAGGTTTCTTCTGTAGGTG |
| 60 | AAGAGAATC | 33 | AAGTAGAGTCTGGTGGTT |
| 59 | GATTCGGATAATCGGA | 33 | AAAAAGATTTAAATCATC |
| 58 | TATTGTCTTCGACAT | 33 | CATATACACA |
| 58 | ATGAATCA | 33 | CAG |
| 57 | AGAGAGAG | 33 | AAAAAATAAA |
| 56 | TTTCTTGTTTCTGT | 33 | AAGAACG |
| 53 | TATTCGA | 32 | GAAAAATAG |
| 53 | ACCAT | 32 | GGCAAAAAATCGTACCGGTTTTTTCCGTAC |
| 53 | AGAAAATTTA | 32 | TTAGGTTGCGCCAAACGAAC |
| 52 | TTAGGTTGCGCCAAATGAAC | 32 | ATTCATTT |
| 52 | GATGGATG | 32 | TACCTT |
| 51 | GAACTCCGATTATCC | 32 | GACAGAAAAC |
| 50 | TTCTCTC | 32 | ACACAGGTGTCAGATCTTGGAATTTTCTGTAGGAC |
| 49 | ATTATG | 32 | AGAGAGAAAGAG |
| 49 | AAACAATTCAAAAAATTCT | 32 | AGAGAT |
| 49 | GTACGGAAAAAACCGGTACGATTTTTGGCC | 32 | TCAAAATT |
| 47 | AGCGAG | 32 | GAAGTGGAC |
| 47 | GAAAACAG | 32 | CCTGAC |
| 47 | AAGAAACC | 31 | ACAGTCTATAGTCT |
| 46 | AAAAAGGTCTTCTGCCCAGG | 31 | GGAGAGAA |
| 45 | CCAAAATAT | 31 | ATTACC |
| 45 | CTTATATCTCTGAAAACT | 31 | AAAGAGAAATAAAAACT |
| 45 | ATGGCCCAGGCAAAAGGTTTCT | 30 | TTCTGTTTTCTAGT |
| 45 | ATTTCAGAG | 30 | GACCTGACGT |
| 44 | ACAGATTC | 30 | GAAATAG |
| 44 | GCCCAGTAAAAGATTTCTATG | 30 | GGAATTGCCG |
| 44 | AAAATCCTG | 29 | TACAT |
| 43 | CGTTATT | 29 | ATATAACT |
| 43 | TGAT | 29 | AATGAC |
| 41 | ATCTGTAAA | 29 | GCACGATTTTTGGCCGTACGGAAAAAAACCG |
| 41 | TTTTTCCTGGGCAGAAGACCT | 29 | ACAAAGTCCTCCTGTGGAGGCA |
| 41 | AACAACAGA | 29 | AGGTTGCGCCAAACGAAGTT |
| 40 | AAAAAGGAGTCTTCTGCCCAGGAAA | 29 | TCTTGTACTAAATTTTATCTTTGATAAAAAAA |
| 40 | AGAGACAGAG | 28 | AGAGGA |
| 40 | CCTTCTTGTCTA | 28 | CAGGTATACCTGTA |
| 39 | GTCACTGTCACCT | 28 | CATTGTTGA |
| 39 | TTGACCT | 28 | TACCCGGTTCCGAAA |
| 39 | AGGTATGGTGACATTAATTAAATGACA | 28 | GCAGGAAACCGAGTTTTATGTGATCT |
| 39 | TCTCCTCCT | 28 | AGAAGGAGA |

| 28 | GAGAGAGAGA | 18 | CTAACTAACCTAAC |
| --- | --- | --- | --- |
| 28 | AACGGAGGATTG | 18 | CAGAAAT |
| 27 | AAGGAATA | 18 | CCAAACGAAATTAGGTTGAG |
| 27 | ACAAATAA | 18 | GAAATCTT |
| 27 | GAGAAAAG | 18 | GACAAAA |
| 26 | TTGCTATGAAACAGGCAAATAACAGGT | 17 | AAGAAGAAGG |
| 26 | TACAAGTGAAATCTATATAACCT | 17 | TAGAGAATC |
| 26 | AAACAGT | 17 | CCAGAAAAT |
| 26 | GGTAAACCTGACA | 17 | TAGCCCAGTAAAAAGGTTTCTA |
| 25 | CGAATACAATAATGA | 17 | TGTTGTTTTATGTTGTTTTC |
| 25 | GGTCAC | 17 | TGAAGAT |
| 25 | AAGAAGG | 17 | TTTTTTTCCTGGGCAGAAGACTCTT |
| 25 | GAAAAATCTA | 17 | CTTGTAAGTTATATAGATTTCA |
| 25 | TGTGCATTTGAACTAC | 17 | GTACGTAGCATAGAT |
| 25 | AACGAGAAAGAG | 17 | ATGGCCCAGGAAAAAAGTTCT |
| 25 | AAGAAGAGG | 17 | AAGGACA |
| 25 | AAGGAT | 17 | CAATTCCAGGCAATTCATGA |
| 24 | CATACTT | 17 | AGAAAGAGAA |
| 24 | ACCAACTTTAATTCATTCCAAACTGTGCGATAAAC | 16 | AAAGAAGAG |
| 24 | TCTGTAGGACACACAGGTGTCAGATCTCAAAACTT | 16 | ACCAGAAACAGAA |
| 24 | CAGGAATTGC | 16 | TGAAACT |
| 24 | TAGTA | 16 | GCGTAAAG |
| 24 | AGAAACTAGGAAACT | 16 | GGAAGAT |
| 23 | AAACAATTCATAGAAATTCT | 16 | AATGGAAG |
| 23 | GAAGGAAAGATT | 16 | GAAATTTTACCACTCTAGGTAAAAG |
| 23 | TTTTCAAA | 16 | TGATA |
| 23 | TGTCTTCTGT | 16 | GAAAAGACTAAGGATGAACTGAAA |
| 23 | TCACTG | 16 | TACAGAAAACAGAA |
| 23 | AATTCCGAA | 16 | AGAGAGACAGAG |
| 23 | GGTAATATTACC | 15 | AAGATCAGTACA |
| 23 | AAAGAAGAAG | 15 | GAAGATGAA |
| 23 | AGACAGGG | 15 | ACGATAA |
| 22 | AAGCAACAGA | 15 | GGTGAAGATGATGAAAATGGAAAGAAAAAAGGT |
| 22 | ACGACTTCTTCTGCTAGCC | 15 | CAGAAACCAGAAAACAGAAAC |
| 22 | CTTTTTTTTCCTGGGCAGAAGACT | 15 | AATGAAGG |
| 22 | ATGAA | 15 | GATTCTGAA |
| 22 | TGAAACAGGCAAATAACAGGCTTGCTA | 15 | ATGAATGAAAGTTGGTGTTTATCGCACAGTTAGGA |
| 22 | AAAGGTTTCTATGGCCCAGGAA | 15 | GTCTATAGTCCAA |
| 21 | AGAGAGAGAGAGAC | 15 | CAAAGATC |
| 21 | CTATATCTACAAATTAATCTACAATT | 15 | ATGAAGTTGAAGAAG |
| 21 | TGAAAAGATTTAAATCATTT | 15 | GAGAGAGAGAGAAA |
| 21 | GCCTCTTTTGTCATTCAAGTA | 15 | CTATAGACTTAGA |
| 21 | GAGAGAGC | 14 | AATTGATG |
| 21 | GAGAGCGAGA | 14 | TTTTTTTTTCCTGGGCAGAAGAC |
| 20 | AAACAG | 14 | CCGTACGATTTTTTGCCGTACGGTAAAAC |
| 20 | TCAGAAAAA | 14 | GAATGAAC |
| 20 | GAACGGA | 14 | CTAGAAGAT |
| 20 | TCTAAAAATC | 14 | GACAGAAA |
| 20 | AAAACAGAAACAGG | 14 | GAAGGCA |
| 20 | CTGGGCCATAGAACATCTTTTTA | 14 | GGGGAAA |
| 19 | AGAGAGAGG | 14 | CAACAGA |
| 19 | AAGAGAGG | 14 | AGACTGAC |
| 19 | ATAAAA | 14 | ACAATTCAAG |
| 19 | TCTTTTT | 14 | AGAAGAAGC |
| 19 | ACAGAAAAG | 14 | AGAGATAT |
| 19 | CAATTCATAAAAATTCTAAA | 14 | AGGAAAG |
| 18 | AAGTAGAAG | 14 | AATT |
| 18 | GCTGTGAA | 13 | GATGAA |
| 18 | GTAAG | 13 | TTTTTACTGGGCCATGAGAACATC |

**¨**

| 13 | ACCTGTTCGA | 11 | AGAAGAAGTCGTGGCT |
| --- | --- | --- | --- |
| 13 | AAATGACAAGGTATGGCGACA | 11 | AATAGAG |
| 13 | TCTTCTTCAAGCCATGAAT | 11 | ATTTAAATCTTTCGAAAAG |
| 13 | **TTGGGG** | 11 | AGAGAGATAGAG |
| 13 | GGATACTAGATACT | 11 | AAACAGAAAACAG |
| 13 | AGAGAGAGCGAGAG | 11 | AAAAATTG |
| 13 | GCACACACTCGGCGCGAGA | 11 | GCCGTACGGTAAAAAACCGTACGATTTTTT |
| 13 | TGAACGGACGAT | 11 | GTATCAA |
| 13 | ATTTTTCTGATTAGTTTA | 11 | TCAAATACACAGTAGT |
| 13 | AGGGACA | 11 | AAGAGGGCGTCATTGGTGCCT |
| 13 | ACCTAATTTCGTTTGGCGACA | 11 | ATGGGAAA |
| 13 | CAAAATCGTAAAATG | 11 | AGGATAACATTCGTATC |
| 13 | CGATTTTTTTCCGTACGGTTAAAAACCGTA | 11 | GAGAGAGAGAGG |
| 13 | AAAAATTGGAAATTTTGCG | 11 | TAAGAATGAACTAGAGAAAAGTAT |
| 13 | CAGACAAA | 10 | CCGGGTAGAACCGGGTATTTCGGAA |
| 13 | ATATT | 10 | AGAACG |
| 13 | CAGAAAAGAA | 10 | TCAAGATAT |
| 12 | TCGTGACTAGAAGAAGAAG | 10 | AATATTTTGCTC |
| 12 | AAATACATTTCAAAATGATTT | 10 | AAAGAGAGAGAAAG |
| 12 | GTTGGCGCCAAACCAAATTAG | 10 | ATGAACTGAAAGAAAAGATTAAAA |
| 12 | TACAAGTGAAATCCATATAAACT | 10 | AAATAAT |
| 12 | ACCT | 10 | AACTTACCCGACACGTGTTTCGCT |
| 12 | AGAGAGAGAGAG | 10 | GTTAAATC |
| 12 | TACTTACCCAGAATAGTAGTCGTTATAC | 10 | TTTTTCTTT |
| 12 | TTACCGTAATA | 10 | AATAAAAA |
| 12 | AAGACGG | 10 | CCCACCCCC |
| 12 | AAAAAGAG | 10 | GAAATAGA |
| 12 | GTGAACAA | 10 | GACCTTGAAAAACCAACTT |
| 12 | TTTGTA | 10 | ATCATT |
| 12 | GAGATCTGACACCTGTGTGTCCTACAGAAAATTTT | 10 | CATAGGA |
| 12 | CTTATATCTCTGAAAACA | 10 | GCCCAGGAAAAAAGGTCTATG |
| 12 | AGGTTGCACCAAACAAACTT | 10 | GAAATTAGGTTGTGCCAAAC |
| 12 | CAATTTTTCCAAAAATTTC | 10 | AAAAAAAAGGAA |
| 12 | GAATTGGCAT | 10 | AACAAGAGAA |
| 12 | AGATACAGGATACA | 10 | GGAAAAA |
| 12 | CCTTTTTTTCCTGGGCAGAAGA | 10 | ACAATCTTT |
| 12 | TATCCGAAA | 10 | AACCTAAGTTCGAACCTAACTTCATATGGCGC |
| 12 | AATTTG | 10 | CCCCCCCCCA |
| 12 | ATGACCT | 10 | AATAATGTCGAATAG |
| 12 | GTCGAACGCTGGAGAGAGAAGAGG | 10 | GTTACC |
| 12 | AAGAAGTCGTGGTTAGAAG | 10 | AGAAGGG |
| 12 | GAGAGCA | 10 | TGATCTTCATAC |
| 12 | CAAACATTTA | 10 | TTTTTAGCCGTACGGAAAAAACCGGTACGA |
| 11 | TACAAGTGAAATCTATATAAACT | 10 | GTAAAATCGAAAATC |
| 11 | GAGAGAGCGAGAGAGA | 10 | TAGCGACATCACAAAAGCACGTTGAGGAACAGC |
| 11 | TCACAGAA |
| 11 | AGTCGAG |
| 11 | TTTAG |
| 11 | TAGACC |
| 11 | TTCTTTTT |
| 11 | AAAAAAAAAC |
| 11 | AAGACAGAAAAGACAG |
| 11 | AGATTCTGAGTTCT |
| 11 | GAGAGACAGAGATAGATAGATAGAGA |
| 11 | GAACGTC |
| 11 | GATTTTTGG |
| 11 | AGGTCCA |
| 11 | AAATA |
| 11 | AAGAG |

| **Gonioctena quinquepunctata (#SRR1867765)** | | | |
| --- | --- | --- | --- |
| 156243 | TTCAATTCGA | 608 | CCGTTTTC |
| 23182 | CACTTT | 532 | ATCTT |
| 20987 | AAACG | 531 | ATGTGT |
| 12599 | GAATT | 514 | TTCACTTCTTTCACT |
| 12090 | GATTC | 514 | ATATCC |
| 10789 | GATAC | 494 | ATCA |
| 10054 | AGAGTCGAA | 466 | TTCACC |
| 9634 | ATCGTATCCT | 448 | TCGAAATATG |
| 9193 | TCGAATCA | 443 | AATCGAATTC |
| 6665 | AACGGGAT | 442 | GGATAACGGGAAAACG |
| 4880 | GAAAGTGAAAGTGT | 439 | TTTGACCTTGAGA |
| 4769 | AATTCGATTA | 438 | CGTATCGTTT |
| 4122 | TAA | 427 | ATCGC |
| 3343 | GAAAGTGT | 423 | AGTTCGTT |
| 2792 | CGTTA | 418 | TCAT |
| 2549 | CGAAAACGA | 403 | GGAAAACGGGATAACA |
| 2399 | GCCATCCGTAGCCGGGAATATAGCTGTTTG | 403 | ATGATCCG |
| 2246 | GATGATTT | 395 | AACCGCGA |
| 1884 | CTTTCACTTTCACT | 377 | GAAATGT |
| 1780 | TGAGAG | 373 | CATCGAATC |
| 1666 | TATTCA | 365 | AGTTT |
| 1648 | ATGATA | 363 | AATTTTGAGAAAAAAAGTCAA |
| 1594 | GAATG | 358 | GAAAAATGACAT |
| 1502 | CAATGTCAAATCT | 358 | TTTTTTCTCAACT |
| 1406 | ATCCT | 356 | GTCT |
| 1390 | TACCG | 336 | TTC |
| 1256 | **TTAGG** | 330 | TTATCTATC |
| 1179 | ATACGATTACG | 327 | GATCCATCC |
| 1090 | ATGAAATGAAAA | 317 | TAGCTGTTTGGCCATCCGTAGCCAGGAATA |
| 1063 | ACACGAAAAATG | 314 | ATC |
| 1043 | GGATTGGAT | 310 | TCGCTT |
| 1020 | TCAAATCTCAAAG | 307 | GATG |
| 1005 | AAAAATGT | 305 | AAATA |
| 996 | TGACGATT | 304 | AATTGTCACTTCGAAGTGAC |
| 977 | TAGGATATGA | 293 | GAATCGAATTGAATC |
| 935 | ACATG | 286 | AATTCACGAC |
| 927 | AGAAT | 281 | ATGT |
| 924 | TTGTCAGTTCAAAGTGACAA | 269 | AGTGAAAG |
| 912 | TAGA | 268 | TCACTTCGAACTGACAATTG |
| 842 | AACA | 267 | TTCTCTTCCAGATTTCTAGAATTTTCTTCAAGC |
| 803 | GTCACTTCAAAGTGACAATT | 260 | AATAT |
| 789 | ACGAAAGGAA | 257 | ACGCA |
| 774 | ATCATCT | 254 | AAGACG |
| 756 | TCATT | 251 | ATACATAT |
| 746 | ATAA | 249 | CACATTC |
| 729 | CATTCATTT | 246 | ATATAGCTGTTTGGCCATCTGTAGCCGGGA |
| 719 | TTTTTTCATTTCGT | 237 | TCGTCTA |
| 707 | GATAT | 233 | GACTCTCTC |
| 702 | AGACG | 232 | AGAATGAC |
| 688 | TCGT | 231 | GACGATTC |
| 678 | GATGGATTT | 228 | AATCGAATTGAATTG |
| 660 | AGGATG | 227 | TTTTACA |
| 644 | AATTGAATCA | 224 | GATGAAT |
| 643 | ATATAC | 223 | TTCCCT |
| 641 | TGAAGCTTCTCTTCCAGATTTCTAGAATTTTCT | 214 | CGAAAA |
| 632 | TCAATTCGAA | 210 | TTTGAAGA |
| 626 | CATATTCTATTC | 208 | ACTATCT |
| 617 | TTCACTTCT | 207 | CAATAAAATTGAA |

| 206 | TCATTTACCC | 125 | ATAT |
| --- | --- | --- | --- |
| 203 | AACATTC | 125 | ACGATACGATAGGAT |
| 203 | CATTA | 125 | ATTGAATTCGA |
| 194 | AATTGATTC | 125 | AAACAGCTATATTCCCAGCTACGGATGGCC |
| 189 | CCGTTATCCCGTTTTT | 125 | AAAATAAAGTTGAA |
| 189 | GATACAATAG | 124 | TTGTTATTTGATAA |
| 188 | CCGTCT | 123 | AAAACGGAATAACGGG |
| 187 | TTAAAAA | 123 | GTCATCC |
| 187 | AAAATTG | 122 | CATCCGA |
| 187 | TGTTGATGGG | 122 | TTCACGAACAGGTG |
| 185 | AGCGAAAGAAGTGAA | 122 | TTGCAC |
| 184 | CCGT | 121 | CCGCTTCGCGGTAGAAAGT |
| 181 | CGACATT | 120 | ATATATACAT |
| 179 | ATAGAT | 119 | ATTCAATTCGATTAAATTCG |
| 178 | AGAGAA | 118 | ATGTGTCCCC |
| 177 | CAATAAAATTGAT | 118 | ATGAACAAAT |
| 176 | TTTTTTCTCAAAATTTTGAGA | 118 | TAACGAAACG |
| 175 | TGACCTTGAGTTT | 116 | TATTTATA |
| 175 | GATGAC | 116 | GTGAAAA |
| 172 | CATTCTAT | 115 | TGTGAAATGAAATT |
| 172 | GAATTGAATCGATTT | 114 | CAATA |
| 169 | CTCGTGCAAC | 114 | CGGAATTCCGACTTACAAGT |
| 167 | AACC | 112 | TATAAA |
| 167 | CGATAATT | 111 | TTCGATTCAT |
| 166 | TGATT | 111 | GGATAACGGAAAACG |
| 164 | TTACTTCTTATT | 111 | AAACCG |
| 161 | AACGG | 111 | GAATAC |
| 158 | TGTTA | 110 | TCAGAACCGTACATTA |
| 157 | GTACAGA | 110 | GGGATAACGGAATAAC |
| 156 | GAATTCCGACTTGTATCTCG | 110 | ACTT |
| 154 | TGTGAA | 109 | AAACTCAATGTCA |
| 154 | CACTTC | 105 | ATCTCTCT |
| 154 | AATTTATTCG | 104 | TTTTATA |
| 154 | AAAAGTCAAAATTTTGAGAA | 103 | ATCCTTTCGT |
| 154 | ATGACA | 101 | TATCTCATC |
| 153 | TGAATCGAATTGAATCGAATTGAATCGAAT | 100 | AATATTCCATG |
| 151 | GTCTCTC | 100 | AAATATGAAATG |
| 149 | AACGGGATAACGAAAA | 100 | TCTTTCTC |
| 148 | ATGTTGG | 99 | TTTGTA |
| 146 | AAAAAAGTTGAAA | 99 | GAGAT |
| 145 | TCACA | 99 | ATACACATAT |
| 145 | AAAAAAAAGTTGAG | 99 | ATATTCCCGGCTACGGAAGGCCAAACAGCT |
| 142 | ATTTATTC | 99 | TATCGTATCGTTTCG |
| 138 | CTTCATC | 98 | GTACCCCCAG |
| 135 | TACTTATACTT | 98 | AAATTTT |
| 135 | GATATAT | 98 | TCTGTC |
| 135 | CGTTATTCC | 97 | TCGATTT |
| 134 | ATTGTTA | 95 | CGGTTTCGCGGTTCG |
| 132 | ATAGAATTTTCTTGAAGCTTCTCTTCCAGATTT | 95 | ATATGAATAA |
| 131 | CGACTTGTATGTCGGAATTC | 95 | CATTCTATTC |
| 131 | TCCAATCGTA | 94 | AATATTCCAG |
| 129 | CTGGTAA | 93 | ACCAGTTACCAGAG |
| 129 | TGAGAA | 93 | GAAAGGT |
| 129 | AAATTG | 92 | TGCGATGA |
| 129 | CTATATTCCAGGCTACGGATGGCCAAACAG | 92 | CAAATGAAAAAAAC |
| 128 | CCGGCAACAC | 91 | CCTCTGGGCTGGTCT |
| 127 | AAAAAACGAAATG | 91 | AAGA |
| 127 | CGTTATTC | 90 | CGGTTCG |
| 126 | CAGATCAT | 90 | GTTATC |

| 89 | ACGATACGAT | 61 | TGTCTGTT |
| --- | --- | --- | --- |
| 88 | CGGTTCTGATAATGTC | 61 | AAATAATAAT |
| 87 | TAC | 61 | AGTTATCCCGTTTTCC |
| 85 | TATATTTT | 61 | TGAGAAAAAAAGTTGAAAATACT |
| 83 | TTGGGTTGAG | 61 | ATAATGG |
| 83 | GTTATCCA | 61 | ATATAGGCAATACGGTACG |
| 81 | ATTTTATTCT | 60 | AAAATAAAAA |
| 81 | GATGGACG | 60 | GTTTCCCGTTATCCC |
| 80 | GATACG | 59 | TAGTAAAAA |
| 80 | AAATAAC | 59 | TCGAATTGAATCGAATTGAA |
| 79 | TTGAGAAAAAAA | 59 | TTCCT |
| 79 | ACGATAGGAA | 59 | TTTATATGTT |
| 79 | ATTATTATC | 59 | GGATAACA |
| 78 | AAAAAACCAAATG | 58 | AGTAG |
| 78 | AATCGATTC | 58 | ACTCTA |
| 76 | GATGAAAAATGA | 58 | AAATAA |
| 76 | TGTTGTTGGTG | 57 | CATTCATC |
| 76 | ATATATAATA | 57 | ATCACT |
| 75 | ACAAAAGGAA | 57 | CGTCGTTTTCGATACGCCATTCT |
| 75 | GGATGGACT | 57 | ATAAAACAT |
| 74 | GTTTCC | 57 | GGAAAGCAGT |
| 74 | ATTTGA | 56 | AATATATATA |
| 74 | TCGTCGTTTTCGATACGCCATTCTCGTCGTTT | 56 | TTGAATCGAATTGAATCAAA |
| 74 | AATAGAT | 56 | TCGATTCAATTCGAA |
| 74 | GTCTCTCCTC | 56 | AACGTG |
| 73 | TATGATACGA | 56 | GGTATGGTAC |
| 72 | AAAGTGAAAGAGCG | 55 | GCGCCATTCTCGTCGTTTTCGATACCAATACT |
| 72 | ATATAT | 55 | TATCCCCGACAAAA |
| 71 | GTGAAAGTGTGAAAGTGAAAGT | 55 | CTGGAAGAGAAGCTTCAAGAAAATTCAAGAAGC |
| 71 | TCATCCA | 54 | GTC |
| 71 | TTTCATTTTTTTCA | 54 | TTGAATTGGG |
| 70 | AGAATATTCCTGTATGAATATTCAT | 53 | CGGAAGAGT |
| 69 | ATCCATAAGAT | 53 | ATGTATATGT |
| 69 | CCATCA | 53 | ATAAAAAA |
| 69 | TTTTTTCTCATTATAATGAC | 53 | GAAAGAAGC |
| 69 | ATCCATAAGATATCCTTCAAGGAT | 53 | AACGGGATAATGGGAA |
| 69 | AACTTTC | 53 | CATAAA |
| 69 | AAAAAAAGTTGA | 53 | AATTGAATTG |
| 69 | TATTCTCAAAATTTTGACTTT | 53 | GAAAGTGAAAGGGT |
| 69 | TTTATTTATACTAT | 53 | GCCATCCGTAGCCGGAATATAGCTGTTTG |
| 68 | AAACGGG | 53 | AAAAATTATGAGACG |
| 68 | CACACT | 53 | CAACACACAAA |
| 67 | TCCCGTTATCCCATTT | 53 | ATATTCC |
| 67 | CCGACT | 53 | ACAGATAAGATAA |
| 67 | TTTTTTATT | 53 | TATTTC |
| 67 | TATATAT | 52 | CTGGTCT |
| 67 | TCTGTATG | 52 | TTGTTAATTGATAA |
| 66 | ATCCCATT | 52 | ATGTTGTATGT |
| 66 | ATTGAC | 52 | AGTGTAT |
| 66 | CAAAAAGTG | 52 | TCGAATGA |
| 66 | AAACAGCTATATTTCCGGCTACGGATGGCC | 52 | AAACGGA |
| 65 | AAAATGACACG | 52 | AGAAATGAAATG |
| 65 | AATCAATCA | 51 | CGAGAACCGCGAAAC |
| 64 | ATTCGAATCGA | 51 | AATTGAATCGAATTTAATCGAATTGAATCG |
| 64 | ATCCA | 51 | AATCGAACTG |
| 63 | GAGAGTC | 51 | TAGTA |
| 62 | GTTCGTGTGTT | 51 | AATCAA |
| 62 | ATAAATA | 51 | AGAAAATATGAA |
| 61 | TTTTTTCTCAAC | 51 | TTTTTTTCTCAAT |

| 51 | CGATTTAATT | 40 | GAATGAAAAATG |
| --- | --- | --- | --- |
| 50 | ATTTCGA | 40 | TTTAA |
| 50 | GATACGGTAC | 39 | GGGACACCTG |
| 50 | ATATATGTATAT | 39 | TCACT |
| 49 | ATTCAACTTTCAAC | 39 | CGGACAGA |
| 49 | AACGGGATAACAGAAA | 39 | ATAATGA |
| 49 | AATTGAATTCAATTC | 39 | ATTCCGACTTATAAGTCGGA |
| 49 | ATACC | 39 | GCTTTCCGCT |
| 49 | AACGAACC | 38 | GGCCAAACAGCTATATTCCCCGCTACGGAT |
| 49 | GGATAACGGGATAACA | 38 | TTTTATACA |
| 49 | TACCATC | 38 | GTCTTTC |
| 48 | TTTTTTCAATTT | 38 | ACCGTACCGTATCGT |
| 48 | GTCCCGAGGT | 38 | TTCGTTCA |
| 48 | AAAGTCTAA | 38 | GAAAAAAACAAAT |
| 48 | GTTTTCCTGTTATTCC | 38 | CTTTAT |
| 48 | ACGAACGG | 38 | TCACACTTTTACTT |
| 48 | ATTTTTTTTTT | 37 | TATCTTATTTATCT |
| 48 | TGAATTT | 37 | TCAAAATTTTGAGAAAAAAAC |
| 48 | TCATAC | 36 | TTTAAAAAC |
| 48 | TTTCTTCTTGTTTC | 36 | CATGTC |
| 47 | GACAGACACACA | 36 | ACTG |
| 47 | AGAACGA | 36 | ACTAACTATACAG |
| 47 | TGCAACCTCA | 36 | CGTACCGTAC |
| 47 | TCAAAATTTTGACATTTTTTC | 35 | TTATAACAGAAAATAGTAA |
| 47 | AATTTCGACTTTTTTTCTCAA | 35 | AAGCACT |
| 47 | TTTTATT | 35 | TCTTTCATTTCA |
| 46 | GTGAAAGTACAA | 35 | GGAATATTCTG |
| 46 | GAATATTCCTGTAT | 35 | TTACCGTTCA |
| 46 | GAAGAAGAACAA | 35 | ATACATG |
| 46 | ACAGTG | 35 | TTTACTTATTAC |
| 45 | TACCT | 35 | ACTCGC |
| 45 | ATTACTTTTATGCTATTTT | 35 | TTGGAG |
| 45 | CACTTTCACTTTCACACTTT | 35 | AAGAGGTGAAAGTGA |
| 44 | ATTCGAATCG | 34 | TCATGT |
| 44 | TCTC | 34 | AAATAAATA |
| 43 | GAGAGC | 34 | GAATGG |
| 43 | AAACGATACGAAACG | 34 | AGTGAGAGTGAA |
| 43 | ATAACGG | 34 | TTCACTCTCTCACT |
| 43 | TCGAATTTAATCGAATTGAATCGAATTTAA | 33 | GGAATGAAT |
| 43 | AACGGGATAACAGGAAT | 33 | TTATCCCGTTATTCCCG |
| 43 | AATATC | 33 | TATCCTATCGTATCC |
| 42 | TTGTATTTTGTA | 33 | TTGTCAAATA |
| 42 | GATTGTA | 33 | CGAGT |
| 42 | ATCGTA | 33 | ATCAGA |
| 42 | GTTATCTC | 33 | ATTTCGTTTTTTTT |
| 42 | TTGAAAAAAAAAAG | 33 | AAGATAAGAT |
| 41 | AATAATAT | 32 | AGAAGAT |
| 41 | TCATTCGTTCTG | 32 | CTAATAA |
| 41 | GAAAGTGAAAGTGC | 32 | ATCCCCGGACAAAAT |
| 41 | AGATAG | 32 | TTTTTTTAATTT |
| 41 | ATCCCGTTTTCTCGTT | 32 | GTCTTAGTC |
| 40 | ATGTATT | 32 | TAAGTCGGAATTTTAAAATTCCGAGTTA |
| 40 | TGTAGA | 32 | TCGAAGC |
| 40 | CAGAGAGA | 32 | ATAATGGGATAACGGG |
| 40 | GAAAAT | 32 | ACGATCCG |
| 40 | AGAAAATTCAAGAAGCCTGGA | 32 | AATCTC |
| 40 | AGGATACGATACGAA | 31 | TAGCTGTTTGGCCATCTGTAGCTGGGAATA |
| 40 | AAACCCT | 31 | TGAAATTTTGACCT |
| 40 | GGTTTCGTTTC | 31 | GACGGAGA |

| 31 | GGATAACGGGAAAACA | 25 | AAAATAC |
| --- | --- | --- | --- |
| 31 | AGAGAGTTG | 25 | CTTTCCA |
| 31 | ATACGATAGGAAACG | 25 | AGAGAG |
| 31 | ACTTGTAACTCGGAATTCCG | 25 | CGTTACGTTA |
| 31 | AGAGGCGAA | 25 | ATGAATATTCATAC |
| 31 | ATTACAT | 25 | AAAAAACATGGCCGCTTGTCATTGG |
| 31 | ATTGTTCAC | 25 | TGAG |
| 31 | AAAAG | 24 | GTGTCGTATC |
| 31 | CTTCTC | 24 | AATATTCAATTC |
| 31 | ATTTTCAACTTTTTTTTCTCAAGT | 24 | TCTACTAC |
| 31 | ACTGGAAAATTACCCCGATTGTG | 24 | TAAACATCAAAACAAAATAAAT |
| 30 | TATACTATTTCATT | 24 | ACCCTCATAC |
| 30 | AAAGTAAAAGAGTG | 24 | TGACAATTGTCACTTCAAAA |
| 30 | ATCGG | 24 | CTTCTTT |
| 30 | TACTGAA | 24 | CGTTATCCCGTTATCC |
| 30 | GACGAGAGACGA | 24 | GAAGGAAGT |
| 30 | TTAATG | 24 | TATGGATATCTTTGAAGGATATCT |
| 30 | CGAAAGTGTGAAAG | 24 | GTAAACGAAAC |
| 30 | TTTGAAGTGACAATTGTCAA | 24 | TCAATAAA |
| 30 | GAAAGTGAAAAAGT | 24 | AACGTCG |
| 30 | TAAACTTA | 23 | GTTAAA |
| 30 | ATATATATT | 23 | GAAAAATGACAG |
| 30 | TTGCCGAACACCCTG | 23 | TATGTGTGTATA |
| 29 | TTATTTCTTATT | 23 | TCAACTTTTTTCTCAAGTATTT |
| 29 | AGTTG | 23 | TGTATGTATG |
| 29 | TCGACTCTTTCGACTCTC | 23 | ATTATTGATATTG |
| 29 | ATATCATTC | 23 | AAAGAAAAAA |
| 28 | ATCAATCG | 23 | ATAAAGTGTATAAGATAAGAT |
| 28 | ATAAAATAGACAGCAAATACAAT | 23 | TAATAGTTACAAGTA |
| 28 | CATTCG | 23 | TGAGAAAAAAAGTCAAATTT |
| 28 | CATTTTCCTGTTATCC | 23 | TATTGCCTATATCGTTATTG |
| 28 | AGTCA | 23 | AAATCTTCAAATCGTC |
| 27 | TAAAGAGTC | 23 | TTCAAAGTGACAATTGTCAA |
| 27 | GAATTCAATT | 23 | GGGAATATAGCTGTTTGGCCTTCCGTAGCT |
| 27 | AAAAAATAAAATTG | 23 | GCGTGAT |
| 27 | TTTCTCTCTC | 23 | CAA |
| 27 | TAGTTCACGTGTGAGACGGGCTT | 23 | TTGGCCATCCGTAGCCAGGAAATATAGCTGT |
| 27 | CGGGATAAAGAAAAAA | 23 | TTTTTTTCATTTAA |
| 27 | TTTTCCGATGACAGACGGCCATGTT | 23 | GAGAGGGCTTTAGTTCACGTGT |
| 27 | ATAAATAACATTTAG | 22 | AGGAAACGATACGAA |
| 27 | ATGTCT | 22 | CGTCA |
| 27 | CAGAACCGCACATTAT | 22 | AAATTTTGAATTTTTTCTCA |
| 27 | AGGTATATTAATAAATAAAATGA | 22 | AGTTGTCCAACACAACACAACCAACACGTTCA |
| 26 | ATCCCCTT | 22 | TTCGTG |
| 26 | GTACTGATTATT | 22 | AAAGGGTG |
| 26 | TTCACCAGAT | 22 | TTCGACTCC |
| 26 | GTGTATATATAT | 22 | CCCCTGTACC |
| 26 | AGAGGG | 22 | TTCGTGTAATTT |
| 26 | GGGTGTTTAG | 22 | AATATCA |
| 26 | TTTCACTTTCATAC | 22 | ATATATAT |
| 26 | TCAAATCATCAAATCG | 21 | GATTCC |
| 26 | TTTCCCAGTACTGTGCTCCCA | 21 | TGCTGAA |
| 26 | CACACGGGGA | 21 | TAAAATAGTTTATAATA |
| 26 | GCATG | 21 | TTTTATATT |
| 26 | AGATCAA | 21 | ATTTTTC |
| 26 | TTTTTTTTCATTTCG | 21 | TTCAATACGA |
| 26 | ATATATACATAC | 21 | CTGACTTGCATCTCAGAATT |
| 26 | AAAACAATGTTAGAGAAA | 21 | TGAAAAAAAAAAT |
| 26 | AATGGAAAATGA | 21 | TTTGAGAAAAAAAAGTCAAAAT |

| 20 | CTTGAAGCTTCTCTTCCAGACTTATAGAATTTT | 15 | TTGTTATTTGATAT |
| --- | --- | --- | --- |
| 20 | TATTGGTCAATTT | 15 | AAGTCGGAATTCCGAAATAC |
| 20 | ATTCGATTCGAATCG | 15 | CGAACGAT |
| 20 | CCTAACCTA | 15 | AATTCGAATCAATTCGATTC |
| 20 | TCTCCA | 15 | ATTCATCT |
| 20 | ATCGTATCGTAATCGT | 14 | ATCCCGTTATTCCCTT |
| 19 | TCGATTCAAA | 14 | TATCCCGC |
| 19 | TTTCTTCGTGTTTC | 14 | AAATGAAAAAAACA |
| 19 | AAAATTTAAAAAC | 14 | TCTGAAAGATATCTTATGGATATC |
| 19 | CGAATTGAATCGAATTGAATCAAATTGAAT | 14 | GTGTCAATGACAACTTCAAATGTCAA |
| 19 | AAGATATCTTTG | 14 | TTTTTTTCAAC |
| 19 | AAAATTTTGAGAAAAAACTC | 14 | AAATAAAAATA |
| 19 | AACGAAACGG | 14 | GTCTATTTTT |
| 18 | TTGAAATGACAATTGTCACT | 14 | AATTCCCGAA |
| 18 | ACGACA | 14 | AATGACAACTTCA |
| 18 | AAGAGGGAAAGTGA | 14 | AATCAACCA |
| 18 | TCTTTTACTTTCACT | 14 | AACTGACAATTGTCACTTCA |
| 18 | CCTTCACTTTCA | 14 | GAATATTCACACAG |
| 18 | TTGAGAAAAAAAC | 14 | ATCGAAT |
| 18 | TTCCAGATTTCTAGAATTTTCTGAAGCTTCTC | 14 | CTCAAAATGTTGACTTTTTTT |
| 18 | ACGATACGAG | 14 | CGCAC |
| 18 | GTTTCTC | 14 | AATCGAATTTAATTG |
| 17 | ATATGGCGTCTGGTTGTCGCC | 14 | AAGCTTCTCTTCCAGATTTCTAGCATTTTCTTC |
| 17 | TTAA | 14 | AAACAACA |
| 17 | TCCAATGACAGACGGCCATGTTTTT | 14 | ATCTCTCA |
| 17 | ATCGTATCCTATCGTATCCA | 14 | GAAATAAAAAAAC |
| 17 | TTTTTTTTTG | 14 | GTTTTTTCTCGCATGACAGATGGCCAT |
| 17 | GACAAAATTCAATATGGC | 14 | CCCAAGTATTCTGAGATC |
| 16 | TTTTTTCATTTCT | 13 | AAACTCAATGATCA |
| 16 | CCGCCATCTTGGAT | 13 | CTATAGA |
| 16 | TTTTTTCTCTAACATTG | 13 | CGAGG |
| 16 | AAAATTAATATTTTAATAA | 13 | CATTCATCT |
| 16 | AATTGAATTGAATTG | 13 | AGGACCATGTGTCGATGTG |
| 16 | TGATTTAAATCACA | 13 | TGCCTATTTTCG |
| 16 | AGAAACCTGGAAGAGAAGCTTCAAGAAAATTCA | 13 | TACC |
| 16 | GATATAGGCAATTCAATAA | 13 | CATAAGATATCTTT |
| 16 | ATCGTATCCTATCATATCCT | 13 | TATGTATTTT |
| 16 | ATATTTTGTCTTGGGG | 13 | GAATCGAATC |
| 16 | TTTATTAAAATATTATT | 13 | TATAAG |
| 16 | AATTGAATCC | 13 | GAAAAAACATGGCCGCCTGTCATTG |
| 16 | CCGATCCATC | 13 | CGTACA |
| 16 | AACGTTC | 13 | ATGATATATATC |
| 16 | TATATTC | 13 | GTCCAAC |
| 16 | TTATCTTATCTTGT | 13 | ATTTCGATG |
| 16 | TATTCCATTCGG | 13 | AATGAAAG |
| 16 | TCATCGA | 13 | CACTTCTCT |
| 16 | TTCTGATAATGTCCGT | 13 | TTATATATT |
| 15 | AATCGAATTAAATCGAATTG | 13 | TGACATGACATGTCA |
| 15 | AACGGGATAACAGAAAA | 13 | AATTGAATATTC |
| 15 | AAACAA | 13 | ATAAATATAA |
| 15 | CTGGAAGAGAAGCTTCTAGAAAATTCAAGAAGC | 13 | CAGATCTAGGAAAAAAAATAAAAAATCCC |
| 15 | GGGGTACCGG | 13 | CCATACGATA |
| 15 | GATAAAGGGAAAACGG | 12 | ACTTTCCCTTTC |
| 15 | GAAG | 12 | TAAAATGTAAAATA |
| 15 | TCATAA | 12 | ATATAGCTGTTTGGCCATTCGTAGCCGGGA |
| 15 | AATTGTCACTTTAAAGTGAC | 12 | TACGATATAGGCAATACAA |
| 15 | GATATGATAGGATACGATAGGATACGATAG | 12 | ACTTTGAT |
| 15 | TTTTCATTTAGTTT | 12 | CGAGAC |
| 15 | TTCATTTTAATTATTAATATACC | 12 | TGACAATTGTCAGTTCTAAG |

| 12 | TTCTGATAATGTACGT | 11 | TTATCCCGTTATCTCG |
| --- | --- | --- | --- |
| 12 | TTTTTTTATTTT | 11 | ACGGTG |
| 12 | TCCTAACCCCATACAGAT | 11 | ACACCCGTTGCGGA |
| 12 | GAAAAATGACAA | 11 | TCGTTTTCA |
| 12 | TTATTATTT | 11 | TTTATATAATAATATTATT |
| 12 | TCCTAGTTTTA | 11 | GATAACGGGAAAACGA |
| 12 | TAGTG | 11 | CATTTTCCGTGT |
| 12 | TTCACCAACAGGTG | 11 | ACATTATCAGAAACGA |
| 12 | ATCAATCAT | 11 | GAAGAG |
| 12 | AGTTTTGATATTG | 11 | GAATCGAATCGAATC |
| 12 | CTTACTTACTA | 11 | CAATCTACATCT |
| 12 | GATATAGGCAATACAATAA | 11 | TCCCGTTTTTCCTGTTA |
| 12 | AATTCGATCA | 11 | ATTTTTTATT |
| 12 | TATACGTATA | 11 | TTCTCAAAATTTTGAATTTTT |
| 12 | TTGATAA | 11 | AAAAAATACAGATAAATATAT |
| 12 | TCAATTCGATTCAATTCGATTCAATTCGAA | 11 | TGCC |
| 12 | AGACGAAAA | 11 | TGCCTGTC |
| 12 | TATCCTTCATGGACATCCATAAGA | 11 | ACATATAAAC |
| 12 | TTTTTTTCTCAACTT | 11 | TTCAGCC |
| 12 | TATGAGA | 11 | AAGTCAATATAATGAGAAAA |
| 12 | TTTCTCAC | 11 | GCTTTCACTTTCAC |
| 12 | GAATAAATATGAACAAATAT | 11 | TTATCCCATTTTCCG |
| 12 | ATCGTATCCC | 10 | CAAATCTCAAAGA |
| 12 | ACATATAC | 10 | CGGCAT |
| 12 | TCTAGAAGCTTCTCTTCCAGATTTCTAGAATTT | 10 | ATAATAATATA |
| 12 | CCGTACT | 10 | ATGTTTTTTCCAATGACAAGCGACA |
| 12 | ATGTTTTTTCCAATGACAAGCGCC | 10 | AACAGTGTATAAAG |
| 11 | AAGCTA | 10 | ACTTA |
| 11 | ACATACATACAC | 10 | CTGAAA |
| 11 | AATTATCAAGTAAC | 10 | CAAGATT |
| 11 | TCCTGTTT | 10 | GTTTGGCCATCAGTAGCCGGGAATATAGCT |
| 11 | CACTC | 10 | CAACAAAAC |
| 11 | CGAAACGATAGGAAA | 10 | TCAATTCGA |
| 11 | ATATACCCTC | 10 | ATCAAAAT |
| 11 | CCCAATTTTCA | 10 | TTTTTTTCTCTCCAATGACAGACGACCATG |
| 11 | AAACGAAACA | 10 | TTACGTTATG |
| 11 | ACCCCCATGT | 10 | TCATATCATATA |
| 11 | GAGAAC | 10 | ACGATTCGAT |
| 11 | AATTGAATCGTATCG | 10 | ATTCTGACTTGCAAGTCAGA |
| 11 | CCCACT | 10 | TCGTATCGTATCATA |
| 11 | GAATCGAATTGAATCGAATTGAATC | 10 | TCAATTTGAA |
| 11 | CCCCATGTTC | 10 | CATTATCAT |
| 11 | TAAATTTG | 10 | GAGTCGAAAGAGTCGAAAGAGTCGAAA |
| 11 | TTTTTTATTCTATATGTA | 10 | TACGGTACGGTATGG |
| 11 | ACGATACGATACGATAGGAT | 10 | TGATTTAAATCA |
| 11 | AAATCC | 10 | ATTATAATA |
| 11 | ATACATC | 10 | GTAGAAAAGTCCGCTTCGCG |
| 11 | TCCCATTTTCCAGTTA | 10 | TACCAAACCA |
| 11 | AAAACGA | 10 | ATTATA |
| 11 | AAAAATACAAATAGAATAA | 10 | TAGAATAAAAAATACAAA |
| 11 | CTTCCACT | 10 | TTTCAACT |
| 11 | ATTCATACATAC | 10 | CAGATT |
| 11 | ACTTTG | 10 | CGATACGATACGATA |
| 11 | ATCAGAACCGGACATC | 10 | ACCCATAAAC |
| 11 | TACGATACGC | 10 | GGGATAACGAAAAAC |
| 11 | AAAAAAAAC | 10 | AAGAAAGG |
| 11 | ATTGCCTATATCGTACCGA | 10 | AAACAGCTATATTCCGGCTACGGAAGGCC |
| 11 | CACTTA | 10 | AAGTGACAATTGTCACTTAG |
| 11 | AAAAAACCAAATAA | 10 | AGG |

| 10 | TTTTTTCATTTCGTTT |
| --- | --- |
| 10 | TACCCCAGGT |
| 10 | CTATATTTCCGGCTACGGATGGCCAACAG |
| 10 | CGTGCTGGTTCTTGTCA |
| 10 | AAAAATTC |
| 10 | CAAAAAGTA |
| 10 | CTCTATCTCT |
| 10 | TCTCTCTCTG |
| 10 | CTGGGGATTTTTTATTTTTTTCCCCTAGAT |
| 10 | ATGTAAG |
| 10 | ATAAAATAGACAGCAATACAAT |
| 10 | TACTG |
| 10 | AAGGGATA |
| 10 | TATTGATCAATT |
| 10 | GCTCAA |
| 10 | AAAATACTTGAGA |
| 10 | ATCCC |
| 10 | TAAAAG |
| 10 | GTCAG |
| 10 | ACATTATCAGAACCGA |
| 10 | TTTCCA |
| 10 | AATATCCTT |
| 10 | ATGGAACGA |
| 10 | AACAAACG |
| 10 | TTTTTTTG |
| 10 | TTAACGAAACGTAACGAAATGTAAC |
| 10 | TATATATATGTATA |
| 10 | CGCCATCTTGGATT |
| 10 | AATGACAGATGTAG |
| 10 | GAGGGT |
| 10 | AAGAATCCAACCCGTGGA |
| 10 | ATACAATACAAT |
| 10 | TCAAAACTCAAAG |
| 10 | AACAGAACAGAG |
| 10 | TTTCTAGAATTTTCTTCCAGA |
| 10 | ATTCTTCT |
| 10 | ATACCG |
| 10 | ATTGAATAGA |

| **Plagiodera versicolora (#SRR8064752)** | | | |
| --- | --- | --- | --- |
| 105766 | TGATGACGA | 285 | TCATCATCATCCTCA |
| 31867 | **TAACC** | 270 | GGGTTA |
| 22386 | ATGACG | 255 | TACGTCCATTGTACATCCCGTATG |
| 20178 | TTC | 255 | ACGATGATGACGATGATGACGATGATG |
| 17661 | GAAGATGAAC | 253 | AAATAACAAATATC |
| 15863 | CAT | 243 | TATTTTATT |
| 14822 | TCTTCGTTCT | 232 | TCATCATCGTCATCATCA |
| 11278 | TAT | 225 | TTGATTGATCTAGGT |
| 7588 | ATGATG | 222 | AAGAACCTGATGGTGTTA |
| 6776 | TCCTCA | 219 | AATC |
| 4963 | AGGATA | 214 | GATTATTCTGGGTCTCCTG |
| 3446 | GAAGAAGAAGAAC | 210 | TGGTTTCAGGTTCCAAAG |
| 2932 | AAAAAG | 210 | TCAATGTGTCAATG |
| 2785 | TATT | 209 | GTTACTGGAATGACACCCTATG |
| 2424 | TCTCCT | 208 | ATAAA |
| 2421 | GACGTTTC | 205 | TCATCGTCATCATCG |
| 1637 | TTTCTGCCTATCAAATTTTAAAATAAAT | 201 | TAACAAATATCAAG |
| 1619 | ATAG | 199 | ACGACGAAACGTTGAAAACGTCAAGCGTCAA |
| 1594 | AAGAAGAAAAAG | 198 | GATGATGATGATGAC |
| 1476 | GGTA | 197 | GAATTATTTATTTCAG |
| 1417 | GGAATAATCCAGGAGACCC | 196 | AGACAAG |
| 1395 | ATGATGATG | 196 | CGCGGCGGCATGACGGTT |
| 1296 | AATAATTCCTGAAATAA | 195 | TTTCAGGAATTATTTTT |
| 1211 | CTGTCATCCCAGTACCTTCTT | 195 | AATAATTCCTGAAAGAG |
| 1128 | GACGCTTGACGTTTTCGACGTTTCGTCGTTT | 195 | TGTCAAA |
| 1112 | GAAGAA | 191 | TCTTTC |
| 1036 | GAATA | 190 | TTTC |
| 1027 | TTTCTGCCTATCAAATTTTAAAATTAAAT | 183 | GGAATAATCCGGAGGACCC |
| 951 | CGGATTATTCCGGGTCTCC | 182 | AATGCTAAT |
| 928 | AAGAAGAAG | 180 | ATTTCAGGAATTAATTT |
| 875 | ATGGACGTACAGACAGGATGTACA | 178 | TATCAT |
| 835 | TTGTTAT | 175 | CGTTTGACGTTTCGT |
| 762 | ATGA | 171 | TAAATGAATAAA |
| 752 | AATGAATA | 171 | ACTGTC |
| 736 | GTCA | 169 | ATTTG |
| 627 | AAGAGATTAGAGAAAGTGAAAGTTCCTAGAATA | 165 | TCAAG |
| 610 | TTCTTTTTC | 155 | AATAATCCGGGAGACCCAG |
| 572 | AATAATGAT | 155 | TTCTTCG |
| 545 | TTGATTGATCTACGT | 154 | TAATC |
| 495 | CAGGAGACCTGGAATAATC | 152 | AGT |
| 477 | GGAGAAGAA | 151 | ACTTTCTTTAATCTCTTCATTCTAGGA |
| 453 | TTTATATT | 151 | GCAAAGTTTG |
| 440 | GAATAATCCGGGAGACCTG | 149 | ATTCA |
| 426 | CAAACGTAGATCAATT | 148 | CAACTACAGAGCCAA |
| 415 | GAAATGAAATAATTCCT | 147 | GCGGC |
| 395 | AATGTCACGTTGTCACGTCA | 147 | ACAGAAG |
| 391 | TTGACA | 147 | CGTCATACCGCCACTCCTTTCT |
| 389 | GGATGACAAAAAAGGGGCTACTC | 147 | ACGTCGAAA |
| 372 | CGCTCCGCAC | 146 | CGTTTCGT |
| 365 | AAATGAATGAAT | 145 | CTTAGGTTAGACACA |
| 350 | TTTTTTCAGGAATTAT | 144 | GGGATATCAGAAGT |
| 348 | GTCATCGTC | 142 | CTGTCATCCCAGCATTCTCTT |
| 335 | GTTTGAC | 142 | AGAATATTCC |
| 333 | GAAAGTTCCTAGAATGAAGAGATTAGA | 141 | GGATTATTCCGGGTCCCCT |
| 320 | GATGATGACGAT | 141 | GTCATCCCAGTACCCTCTACCTC |
| 290 | TTTCAGGAATTATTCTT | 139 | GATGACGATGATGACGAT |
| 287 | TTCGTTCTTT | 138 | TTGTTGCT |
| 285 | CAACTTAA | 138 | GTAT |

| 137 | GAAGAAGGCACTGGGATGACA | 86 | TCATCGTCATCATCATCG |
| --- | --- | --- | --- |
| 136 | TCAGCCCCACCG | 85 | GAGAAGA |
| 135 | ATGACAG | 84 | GGAGACCGGAATAATCCA |
| 132 | CCGAGATGATACCGACTGCCA | 84 | TGGACATTCAT |
| 132 | ATGACA | 84 | TTTTACAT |
| 131 | GAAACGTTGAAAACGTCAAGCGTCAAACGAT | 83 | TAACG |
| 130 | TTTTTCTTCTTCTTC | 83 | ACTT |
| 129 | TTTTTTTTCTTCTTC | 81 | TTATTCCGGTTTCTCCCGGA |
| 128 | CTGGGATGACAAATAGGGGTG | 81 | AAGAAAG |
| 128 | ACTGTCA | 81 | TAATAATGATAA |
| 127 | GAATATTCTAGATATTCATG | 81 | TTCATTCG |
| 126 | TCTGAAATCCCACT | 80 | CGTCTATAAGG |
| 125 | TCATTCATTC | 80 | TTTTTCTTCTTT |
| 125 | CTGGATTATTCCGGATCTC | 79 | GAGATGATACCGGCTGCCACC |
| 124 | TCTTCCTTCA | 79 | TCTTCTTC |
| 123 | ACATCCATTGG | 77 | GATTATTCCGGGCCTCCTG |
| 122 | GGATGACAAAGAAATAGGCACTG | 77 | ATTCAAAGAT |
| 121 | ATTATAGAGAGGCACTGGGATGAC | 77 | TTATTTTTTT |
| 121 | AAACGTCAAGCGTCAAACGATGAAACGTCGA | 77 | AAAATATCG |
| 120 | AAGAAGAGA | 76 | AGAAAAGAAAA |
| 118 | CTTCAT | 76 | ATGGTAATTTTGATCTTG |
| 115 | GATTATTCCGGGTCTCCCA | 74 | GGATTATTCCGGGTCCCCC |
| 115 | CCAA | 74 | TAAGAATGAACTGAAGAAAATAT |
| 115 | TACATT | 73 | ATTAAAA |
| 114 | ATATTCTGAGA | 72 | GGACCTCAAGGTGATAAA |
| 113 | TGAAA | 70 | ATTGCATGCAAT |
| 113 | GGGTCTCCTGGATTATTTC | 70 | AAGATGAACGAAGATGAACGAAGAAGAACG |
| 113 | GGGCACTGGGATGACAAAAAAG | 70 | ATGACAA |
| 112 | TGTCAATG | 70 | ACAAATGA |
| 108 | GATGATGACGATGAG | 70 | TGATGATGATGATGACGATGATGACGA |
| 108 | GATGAGGATGAC | 70 | TGGACATCCAC |
| 107 | ATATA | 69 | ATTATG |
| 105 | GCCACCGCGAACCGTCAT | 69 | TATGGTT |
| 105 | GGAAAGAA | 69 | TGTCAATGTCAAC |
| 103 | AAATACAA | 68 | ATTGACAAAGAC |
| 103 | TTCTTCGTTT | 68 | TTTTATTT |
| 101 | TTGACGTGACAACGTGACGT | 68 | AAATAGAAAAATTATATAAGG |
| 100 | AATGAAGA | 68 | CCTTGAGCACCTTTTTCT |
| 98 | ATTTTTGAC | 67 | AGTTAGTCTTG |
| 98 | AAAAATAG | 67 | GAAAGAGA |
| 98 | AAGAAGAAAG | 67 | TGGTTGTAACCGCCT |
| 98 | TAGTTGTCGACTCTG | 67 | GAATATTCTGGATATTCATG |
| 98 | AAAAACCGCG | 67 | AAAAATCGATTTTTT |
| 98 | GGAATAATCCGGAGACCC | 66 | GATCAACCAA |
| 96 | CAGAGAAGA | 66 | GACGTCCATTG |
| 95 | CGGCTCAGCAAGACAAAATAA | 66 | TTGACATTC |
| 95 | TCC | 66 | ATCCGAGGGACCCGGAATA |
| 95 | AAACGACGAAACGTCG | 65 | AAATCGATTCTAAA |
| 94 | GGATTATTCCGGATCTCCC | 65 | ATTATTTTCTTTCAGGA |
| 94 | TTTCAGTTCATTCTTAATATTTTC | 65 | GATGACGATGAAT |
| 94 | TTGCAGCAATATTGCAGAACG | 64 | CACTGTCATTGT |
| 94 | TCAAGTCAAGTAATGAATATCTCAAATCAAG | 64 | TTATTCCGGGTCTCTCGGA |
| 93 | TCATCATCGTCG | 64 | AATCAATCATG |
| 90 | AGAAAGAGA | 63 | TGGCTCCGTAGTTGT |
| 88 | CTTTATCACCTTCAGGTC | 63 | CATTTCTTG |
| 87 | AATAATCCGGGAGACCGG | 63 | GATGATGATGAT |
| 87 | TCTTCGTTAT | 62 | TTCTCTATTCTA |
| 86 | TGTCATCCGAGTAGCCCCTTTCT | 62 | GGATTATTCCGGGCCTCCC |
| 86 | TGTTCCATGTCATCGCTGTCAATATTATTG | 62 | CATTCTAGAAACTTTCACTTTTCTAATCTCTT |

| 62 | ATTATTTTT | 50 | TTACA |
| --- | --- | --- | --- |
| 62 | TCCTCTTTC | 49 | AATAAT |
| 62 | AACGAAGATGAACGAAGAAG | 49 | CTTCGT |
| 62 | GTCAGTTGTCAAAAGCT | 49 | TTG |
| 61 | GGGCTGAGAAGAAGAGGACCAGGC | 48 | TCCACTGGATG |
| 61 | TTTTGGGATT | 48 | ACTGAC |
| 61 | GAAGAAGTTTCCAGAAAAAGTGATGCAAAATTG | 48 | TTCCAAAGACCACCA |
| 60 | CACCGTTAGCCC | 48 | GACGTTTCGACGTTTTC |
| 60 | CTAGAGACTAGA | 48 | TGACATTGAC |
| 59 | ATATTTT | 47 | ATTTCAGGAATTATTCT |
| 59 | ATTATTATCATC | 47 | CTAACGGTGGGGCTGACGGTCGGG |
| 59 | TCTCCTC | 47 | AAGGGGGACAAGCAGGAC |
| 59 | ATTTATT | 46 | TGTCATA |
| 58 | AAGACTCCCCG | 46 | TCCTCATCT |
| 58 | CTGTCATTCCAGTGCCTCCTTCCC | 46 | TTCTTTGTTC |
| 58 | ACCCAACCTA | 46 | CATAGAATGTC |
| 58 | TCCGGATTATTCCGGTTCT | 46 | CCTGGATTATTCCGTGTCT |
| 58 | GCACTGAGATGACAAAAAAAGGA | 46 | ATAATCCGGAAGACCCGGA |
| 58 | AAATAA | 46 | TACCGCGGCGTATGTTAGCATTATA |
| 57 | AATAATAAC | 45 | TTTTTTTCTCTAACATTG |
| 57 | TTTTATTTT | 45 | GATATTTT |
| 56 | ACGTTTTCGACGTTTCGTCGTTTGACGCTCG | 45 | AGGTCCCAAGGGCCCCAT |
| 56 | TCAACCAAACTG | 45 | AAGAGAAACT |
| 56 | GAATATTCCAT | 45 | TTTAC |
| 55 | AAACGTCAAGTGTC | 45 | ACTTTCTCTAATCTCTTCATTCTAGAA |
| 55 | CTAAACC | 45 | TCAAGTCAAGTAATTAATATCTCAAATCAAG |
| 55 | TAGGTTAAGT | 45 | GCACTGGAATGACAGGGAAGGAA |
| 55 | TCCGTAGTTGTCGAC | 45 | TATCTTTC |
| 55 | AATAATCCGGGAGAACCGG | 45 | TCTTCTTAAT |
| 55 | CAATA | 44 | ACATGCACTGG |
| 55 | CGAAGGAGAAGAA | 44 | TCATCATCATCATCA |
| 55 | CATGCTCAAAATGTAGTTGCTAG | 44 | CAAAGTGGTTTCAGATTC |
| 54 | CTTGTCCTG | 44 | TTTTGACATTTTTGACATGACTTTC |
| 54 | GTC | 44 | ATGAAAATTCAAATCGACCAGACTTCC |
| 54 | TTTTGGAATGGTGGTCTTTGGAAATTA | 43 | ATTTCAGGAATTAATTC |
| 54 | GGATTATTATGGGTCTCCC | 43 | ATAATCCAGAGACCCGGA |
| 54 | ACTATCTA | 43 | TTATAGA |
| 53 | AAGAAAAAA | 43 | ATGATGACGATGATGATGATG |
| 53 | TTCCAGCAATCTCTAAATAT | 43 | TTTCCTTAGATAATTTCTCTA |
| 53 | GCAGATGCCAAAGAAAGAATG | 42 | GGTCTCCCGGATTATTCCT |
| 53 | GCACTGGGATGACATCAAAGGA | 42 | AATAACCAAGAAGCATAG |
| 52 | CAACCAAATTTCCAAAGACCC | 42 | AAATCTGATTCGCAATTTCTTCACGTTGTGCAG |
| 52 | AGCTT | 42 | GATGACGATGAC |
| 52 | ACACACACGAGATCACTGGAA | 42 | TTATC |
| 52 | AAAATTAAATTTT | 42 | GAAGAAGAAGAACGAAGAAGAAC |
| 52 | CTCAGAACCTCTCCTCCAAACAC | 42 | TTCAGGAATTATTTCT |
| 52 | TGTACAATGTACGTACAGACAGGA | 42 | AAAATGCA |
| 51 | AATAATAT | 41 | ATAATAAATA |
| 51 | ATGGACGTACATACAGGATGTACA | 41 | ATCACGAAACAACTGTCAAAT |
| 51 | AAAATTATTAAAAACGACCAGATTTCCAAA | 41 | ATGTAAA |
| 51 | AAATAATCCGGGAGACCCG | 41 | GACAAGT |
| 51 | CGTTCATCTTCGTTCTTCTTCGTTCTTCTT | 41 | CCATT |
| 51 | ATCTCTCT | 41 | TTTACTCGATTGTGACTT |
| 50 | CAACAAAGAAG | 41 | TAATATA |
| 50 | TCGAAAACGTCAAACG | 41 | AAATCGATTTCTAAA |
| 50 | GTTGGGATCTTTGGAAATTTG | 41 | GAGTCGACAACTACGGAGCCAACAACTACA |
| 50 | ATTTTATTTCAGGAATC | 41 | CCACTTCTGATTGAAAAATC |
| 50 | AAAAG | 41 | TTCCACCTTTTTTT |
| 50 | GGATTATTCCGGGTCTCAT | 41 | ATATTTCA |

| 40 | TATTAC | 34 | GGATATACACGCCTCGT |
| --- | --- | --- | --- |
| 40 | GGTTGTGGTTCA | 34 | AATGAAATGATTCCTGA |
| 40 | TGTCAAAAC | 34 | ATGTGTC |
| 40 | GATAGATAGAAAGATC | 34 | ATGCTCATTACGCT |
| 40 | AGACATTTAGTGCAATTGTCTTAATGTTTAC | 33 | TGACGGAAGTAATTGAGGTTATGTTCAAAT |
| 40 | AATGTCACA | 33 | TCAAGTCAAGTATATAAATTGTCACGAGCTTGATT |
| 40 | AACTGCTGGCA | 33 | ATGTCCAATGC |
| 40 | CCTTGGGA | 33 | AAGAAAAAGGAAAG |
| 39 | AAGCC | 33 | AGATACTTCCAGATAGTCTC |
| 39 | AGAGACAGA | 33 | AAATGACACAAAATATGTGAC |
| 39 | AAGTATG | 33 | AATCTAACCTAACCTAACCTC |
| 39 | CTAACCTAAGCCTA | 33 | CTCACTTA |
| 39 | ATTTGACGTGACAACGCGAC | 32 | AATGACATATGAC |
| 39 | TAGAAA | 32 | ATGTCAGAA |
| 39 | ATTACAAA | 32 | AAAGGAAGGAGCATCCAACGAATAAATAAGG |
| 39 | GGATGACATTATAGGAGCACTG | 32 | ATTTCTGCCTATCAAATTTTAAAATTAA |
| 39 | GATTATTCCAGGTCTCCCA | 32 | GGGCACTGGGATGACAAAAAAAG |
| 39 | AAAATGAAAT | 32 | TGACAGATT |
| 38 | GGATTATTCCGGGTCTCCA | 32 | AAGTGAAAGTTTCTAGAATGAAGAGATTAGA |
| 38 | TTCTTTTTCTTT | 32 | AATAATCCAGGGACCCGG |
| 38 | AATGAAT | 32 | AAAGGAGCACTGGAATGACAAAA |
| 38 | GTCTGTCAGT | 32 | GGGCACTCGGATGACAAAAAAG |
| 38 | CCGGATTATTCCGAGTCCT | 31 | AGAGTAATA |
| 38 | CATTGTCATCTGT | 31 | TTAA |
| 37 | TCATCATCGTCATCATCATCG | 31 | TGTCAATGTCAG |
| 37 | CTTTTTCTTCGTT | 31 | ATTACAATTTACA |
| 37 | TTCCAGGTCCCCCGGATTA | 31 | AAATAATTTCTGAAATA |
| 37 | AAAAAAAAAG | 31 | TTAGTCTTGAA |
| 37 | AATTA | 31 | TGTCATCCCAGTGCTCCTTTTTT |
| 37 | TTTTTAT | 31 | TTTTCTTTTTTT |
| 36 | AACGTAAG | 31 | ATTATACTTATCCCACTTCTG |
| 36 | GACGATGACGATGATGACGATGAT | 31 | TGGACGTCCAC |
| 36 | TCCCCGGATTATTCCGGG | 31 | CATGTCAAAAATTGTCAATATTAGTGTGTTT |
| 36 | TTTTTTTTTCT | 31 | GGAACATTATGATCGATATTT |
| 36 | CAACAGAAGATCCATGGATTA | 31 | TGGATTATTCCAGGCCTCC |
| 36 | GCTGTCACTT | 31 | GGTGTCATCCCAGTGCCCAGA |
| 36 | ATGAATGA | 30 | ATATATATAC |
| 36 | GAAAGACGAAACGTCG | 30 | GAAAATGGACTGAAATTGGCAAAAATGACAAA |
| 35 | GGAGAAGGA | 30 | TTTTTTTTATT |
| 35 | AACGTGATCATAGCGTGTCAC | 30 | GTCACTGTCAAT |
| 35 | TTCTTCTTCTTC | 30 | CAAATTCTA |
| 35 | AAACTTTCACTTCTCTAATCTCTTCATTCTAG | 30 | AATACTATCCA |
| 35 | ATTCTATTCC | 30 | ATATTCAAGG |
| 35 | TTTAATTCAA | 30 | TCTATCTCTC |
| 35 | TCCTTTCT | 30 | TTCTCTGCGTTTTAAATG |
| 35 | TCAAAGA | 30 | AATAATTCCTGAAAAG |
| 35 | TCAATGTCATCTG | 29 | GAAACAACTGTCAAACATCAC |
| 35 | AGAAACGAT | 29 | ATCGAATAAATGACTGAATAATTTTATG |
| 35 | TCATTTTAT | 29 | GGACAAAAAATGACCAAAA |
| 35 | GTTA | 29 | CGAATCAGATTTCTGCACAACGTGAAGAAATTA |
| 35 | TATTTATTCATTCATT | 29 | AATAATCCGAGGAGACCCGG |
| 35 | CTCCTCTT | 29 | TTTTTATGGAAATCTGGTCGTTTTGAA |
| 35 | CCTCTTCTT | 29 | TAAATAC |
| 34 | TGTCTTT | 29 | TCATTTAC |
| 34 | CATTTTGTCTTGCTGAGCCGT | 29 | CGACCAGACTTCCATAAAAATTCAAAT |
| 34 | TTATCATCA | 28 | AAAAAGTACATAAAA |
| 34 | ACTCTTC | 28 | AACAAAAAGAGAAGAAC |
| 34 | ACATTTACATT | 28 | TGC |
| 34 | TTATTCTCAATCAATAAATGT | 28 | ACAATTT |

| 28 | TGGACATCCAA | 24 | GTTGGCCCAGT |
| --- | --- | --- | --- |
| 28 | ATTGCTACAACGTTGCAGCAAT | 24 | GATGACGATGAGAT |
| 28 | CAGGACACTATATCTGTCATCTGTCA | 24 | TTTTTATTTATACATTA |
| 28 | GTCCATTTTAC | 24 | GTCCTAAGGAT |
| 28 | TATTTCATT | 24 | TTCTTTTCTTC |
| 28 | AACTTTCACTTTCTCTAATCTCTTCATTCTAGG | 24 | AAAAGG |
| 28 | GCGTGTATATCCATGAA | 24 | ATCCGGGGGACCAGGAATA |
| 28 | GGAGACCCGAATAATCCA | 23 | CAGCTT |
| 28 | ATTGATTGATG | 23 | CGC |
| 28 | ACATTCTT | 23 | ATTTGA |
| 27 | CGTTTTA | 23 | TTGTGAAAT |
| 27 | AACTGTCAAAGCTCCCTTGTC | 23 | GGAGACCTAGAATAATCCA |
| 27 | AAAAGTATCCGAA | 23 | AATAAACGAACG |
| 27 | AGACAAGAGACA | 23 | GAAGAAGAAGAACGAAAAAGAAC |
| 27 | ATATAA | 23 | ATAATAATCCAGGAGACCC |
| 27 | GGGAGAACCAGAATAATCC | 23 | TAGTATTTTA |
| 27 | CAACTTTAAATACGTAATCAGAACATGG | 23 | CATAGGGTGTCATCCCAGTAAC |
| 27 | ACATTAATTTA | 23 | ATTAGAGAAAAAATCAACCACCC |
| 27 | TCTCTA | 23 | TCCAGCTTCACCAGATATTCTTTGTTGAA |
| 27 | TTTAGAAT | 23 | AAAGAGGCTACTCGGATGACAAG |
| 27 | GGTTAGATTA | 23 | CACTTGA |
| 27 | ATTTGT | 23 | GAATAATCCGGGAGACCTA |
| 27 | TTTTTTAAT | 23 | ATGACGTTACAGA |
| 27 | TCATTATTATTATTA | 23 | TCTTTTTCTTCTTTT |
| 27 | ATTTGTCATGTC | 23 | CATTGAAACGGTATAATCATA |
| 26 | GTCGTCGTAGTAGTAGGG | 23 | TTAGGTTAGGTTAGA |
| 26 | ATCATTGTCTCA | 23 | ATTCCAGGTCTCCTAGATT |
| 26 | GAGAAGAAAAAG | 23 | TGACAGAAA |
| 26 | CGTAACCCAACGTAA | 23 | ATCAGAAATCAAAT |
| 26 | TCACATTTCACAT | 22 | TTCTTCTTCTTG |
| 26 | TTGACACAGAACATGATTCGGT | 22 | TTGGAGTTGGTTCTGGTTGAGGCC |
| 26 | ACAGTTGCAAA | 22 | AAAATATG |
| 26 | GAAGTAATT | 22 | TGTCAAT |
| 26 | TCAATTTAT | 22 | ATCCATAGAGATATCCTTCAAGGAT |
| 26 | CAAACCTAGATCAATT | 22 | CGTTACGTTTA |
| 26 | AAGAAGAAGAAAAAGAAA | 22 | TTTGATA |
| 26 | GGACATAAATT | 22 | AAGAAGAGAAG |
| 25 | ATTGAGGTTAG | 22 | ATTGCGTGATCACAAAGTATC |
| 25 | CTTGTTTACGTT | 22 | TTTTTGGCAT |
| 25 | AAAGTGTCAATGTC | 22 | TTCTTCTCGTTC |
| 25 | TATTTCGA | 21 | TGGACGTTCAT |
| 25 | TTCTGAGATCCCAC | 21 | AGAAGAAGAAGG |
| 25 | GACGTTCGACAGCTGTCAATGAAT | 21 | ATAGTATCGATAGAAAAGGTCG |
| 25 | GGACATGAATT | 21 | TTTCAACGTTTCGTCG |
| 25 | ATGAATAAATGAATGA | 21 | GTTAAAGAACCTGATGAT |
| 25 | ATCCCAGTGCCCATTTTTTGTC | 21 | TTGAAAATTTGGTTGGGGTCT |
| 25 | CAAACCGCATTTTCAT | 21 | TCGTTGATTA |
| 25 | AAGAGAAAGAAG | 21 | ATTCCTGAAAGAGAATAATTCCTGAAATAAAATA |
| 25 | GACGTTTGACGCTT | 21 | AACAACAAA |
| 25 | AACCGTCATACCGCCGCG | 21 | CGGATTATTCCAGGTCTC |
| 25 | AGAACAG | 20 | GATAAACGAA |
| 24 | ATAATCCGGAAGACCGGA | 20 | GATGTGTCAATGTC |
| 24 | GACATATGACAAGT | 20 | CGTTTCGACGTTTGACGCTTGA |
| 24 | CTCATAC | 20 | ATATGACAGTTGAC |
| 24 | AATATAAACATATTACAT | 20 | TGCCAGCAATT |
| 24 | GAACCAACCCCAAGGCCTCAACCA | 20 | GAATAATCCAGGAGAACCA |
| 24 | ACGTACATACGGGATGTACAATGT | 20 | TTCTGATATTCGAT |
| 24 | AACCAGAATAATCCGGAAG | 20 | GGTCTCCTGGATCATTTCG |
| 24 | TGTCAGAAGAAAGTCATGTCAAAAA | 20 | TCATTAATTTATATTTTCATT |

| 20 | ATCCAGAATAATCCGGGAG | 15 | TTATTTCAGGAATCAT |
| --- | --- | --- | --- |
| 20 | CCGGATTATTCCGGTTTCC | 15 | ACGTTTCGTCGTTTGACGCTTG |
| 20 | AGACTAGAACT | 15 | GAGACCCTGAATAATCCAG |
| 19 | TATGTGTGTG | 15 | AGAAGAAGAAAAGAAAA |
| 19 | TTTTCTTCA | 15 | GTTGTCCAATTCCAACTGGGA |
| 19 | GATGACGATGACGAC | 15 | ACGAAACGTCGAAAACGTCAAACG |
| 19 | AAGTCATGTCAAAAATGTCAGAAAA | 15 | ACTTGTTGATTCACTCGAATC |
| 19 | ACCACAACCAGA | 15 | AAAAAGATCAGGAAACT |
| 18 | AAATAATAAAG | 15 | AAGAAAAAAAAAA |
| 18 | TTGACATTGACAC | 15 | ATCATCATCATCATCATCATCATCGTC |
| 18 | TCAATAATTTGACAGTTTGTCATTG | 15 | AATTTTTATGGAAGTCTGGTCATTATTAAA |
| 18 | AAGAGAAC | 15 | TCATGACATCTGTCAAAACTGTCAACTT |
| 18 | TCTTCGCTTATGTCC | 15 | GAAGAAGAAGAAA |
| 18 | TTCTGAT | 15 | AAGAAGAAAGAG |
| 18 | GGAGACCCGGAATAATCTA | 15 | GTAAAC |
| 18 | TGACAAGTGACAGTAA | 15 | TTTTTACTCGATAGTGAC |
| 18 | ATTTAATTTTAAAATTTGATAGGCAGAAAC | 14 | AAAATACAAAAATATA |
| 18 | AGAACCGGAATAATCCGG | 14 | TGTCATCCCAGTGCCCTTTTTT |
| 18 | GAAAGATAGAAGAAACTTCTC | 14 | TGACAGATGACAGATATAGTGTCCTA |
| 18 | ATTTATTATTT | 14 | GACGATGACGACGATGATGACGATGAT |
| 18 | TGGAATGACAGAAGAGAGCAC | 14 | CGGATTATTCCGGGTCTCA |
| 17 | ATATC | 14 | ACGTGTTTATGAAGACGTCTCATAA |
| 17 | AATAACGGTTTCTGCCTATCAAATTTTAA | 14 | CATTAGACAAGTGACAGCAGTCAAA |
| 17 | ACCGCGACCGCCGCCATCTTAGTAT | 14 | ATAATATAAATAAA |
| 17 | TTCATTGATTCA | 14 | ACGCTTGACGTTTTCG |
| 17 | ATTTATTCATTA | 14 | AGAAGAAGAAAC |
| 17 | TAACT | 14 | GAAAAATAGAAGAAACTTCTA |
| 17 | CTGAAAAAATAATTC | 14 | GAATAATCCAGGAGACCAG |
| 17 | TAATTTATTTCAGGAA | 14 | ATCAATTGGAC |
| 17 | GACGAATTTGTTGAT | 14 | AAATGTCAATAATATCGA |
| 17 | ACGTTAT | 14 | TGTGGTCCAGGT |
| 17 | GAAGAAGAAGAAGAAC | 14 | AATGTCACATGGTGTTACATG |
| 17 | ACTTGTCAAATGTTTGACTGCTGTC | 14 | AGTATCTGAG |
| 17 | ATTATTCCAGATCTCCCGG | 14 | CCAATGTTGTCAACGCTTCTG |
| 16 | TCATCATCATCC | 14 | TGAAATAAATTGATTCC |
| 16 | TGTCAATGTCAAAAGTCACC | 14 | TTTTTCTAGAAGTTTCTTCCA |
| 16 | GATGATGATGACGAC | 14 | GGGATTTTT |
| 16 | AGACTAACCCC | 14 | TTTTTCTCTAACATTGT |
| 16 | CTTGCTATTGC | 14 | ATGGACATGAA |
| 16 | GACATATTGACATT | 14 | GTTATTC |
| 16 | AGATATCCTTCAA | 14 | GTAACGTATAAC |
| 16 | AATATTGAA | 14 | CTAGTA |
| 16 | GAATAATCCAGGAGACCA | 14 | ACATCAGAA |
| 16 | ATATATTG | 14 | ATTCTATTTT |
| 16 | CCAGTTCTCCATAAACCTCTAATGAA | 14 | TTACCATAATCTG |
| 16 | ACGAAGAAG | 14 | ATATTCATGG |
| 16 | ATCCTCAACATCATC | 14 | AAATGTCAAACGTCAA |
| 16 | AGAAAAAGAAAAAGAAGAAAA | 14 | ATTCTAGGAACTTTCACTTTCTCTAATCTCTTA |
| 16 | AGAC | 14 | GAGGTTTTGAG |
| 16 | AAATTG | 14 | ACGTTGACAATTGACGTGTCTACAGCAACAGGTG |
| 16 | GATGAGGAT | 14 | CGTCAAACGATGAAA |
| 15 | AAGAAAAAAAGAAGAAGAAA | 13 | GCCCAACCGTTA |
| 15 | TTTTATTATATTA | 13 | TATTTTAAAATTTGATAGGCAGAAACAGT |
| 15 | GGATATCTTAT | 13 | GTTGTACTTCTGGGACCATAA |
| 15 | TCCCGGATTATTCAGGTC | 13 | ATATCAATA |
| 15 | GACAACTACAGAGCC | 13 | TTTTGGGAGT |
| 15 | TCATACTTCATTTT | 13 | TTATTTTTCTTT |
| 15 | GAAGAGGACAGAAGC | 13 | ATTATATTATT |
| 15 | AAGAACGAAAAAG | 13 | GGTCCCAAA |

| 13 | AGGTTAGGTTAGATTG | 12 | AATTAATGAATG |
| --- | --- | --- | --- |
| 13 | AACGTGTTTATGAAGACGTCTCATG | 12 | CATAA |
| 13 | CCGGAATATCCGGAGTTCAAA | 12 | AATCAATG |
| 13 | ATGTCATATGTCA | 12 | AATCATGAAA |
| 13 | ATGAAGAAAAAG | 12 | AATAAATA |
| 13 | ATGTCACCTGTTATTTGTCAA | 12 | AGGAATTATTTCTTTTC |
| 13 | AAGCATAGAATAATCAAG | 12 | CCCATAGGT |
| 13 | TGTCATAATCAACAAGTATC | 12 | GATGACGATGAA |
| 13 | AAAGAC | 12 | ATATGGAAATA |
| 13 | TGTCATCCCAGTGCTCCTTTTCT | 12 | TGCC |
| 13 | CTGACATTA | 12 | AAAAACGAAGAAG |
| 13 | TTGCAACTCAGTAAGCATTAT | 12 | GTCAAATGTCAACGTCATGT |
| 13 | AATAATCCGGAAGACCCAG | 12 | GACGTTTTCGACGTTTCATCGTTTGACGCTC |
| 13 | TAAAAAAAAAAA | 12 | TTCCGGATTA |
| 13 | AAAAATCCGGAAGAACCAG | 12 | ATCTCTAGT |
| 13 | GACGATGACGATGATGAC | 12 | GCCCCGACAGCAGGCCTCCACCA |
| 13 | CCAACCAA | 12 | TCTAACGATACAGGTTTCTGTGTT |
| 13 | CCTTTTTT | 12 | AATTAGTTTGTTC |
| 13 | TGGAATATTCTAGATATTC | 12 | ATCATCTCTAGAAATGGGCAT |
| 13 | CGTCAAACGACGAAACATCGAAAACGTCAAG | 12 | TTTTTGGGAA |
| 13 | ACATTACGTAAC | 12 | CAAACATAGATCAATT |
| 13 | AGCTTCCAAAGTGGTTTC | 12 | ATAATATT |
| 13 | GGATTATTCCGGTCCTCC | 11 | TGACACATGACAT |
| 13 | AGAGTAA | 11 | TTTTTTTTATTTT |
| 13 | GGACGTCCTAT | 11 | AAAAATGTTGAAATGT |
| 13 | AAATTGA | 11 | AAAATTCGTGTTTTTGGATC |
| 13 | AAAAATGACAAAGAAAATGGACTGACATTGGC | 11 | GGTTGAGGTTA |
| 13 | TTCC | 11 | AAGAGAAA |
| 13 | ACGTTTCATCGTTTCG | 11 | ATAGGATAGA |
| 12 | TTTTTATGAAA | 11 | TAATCCGGGGGAACCCAGAA |
| 12 | AATATAAAA | 11 | CTCCTATTT |
| 12 | CTGGATTATTCCGGGTCCTC | 11 | ATCGATAGAAAG |
| 12 | TAGAAGAAGAAAATG | 11 | ACAGTTCCAAAAACGGCACAGTGACTTTG |
| 12 | GGATTATATCGGGTCTCCT | 11 | AAGGCTAATTCAAGATTGATTT |
| 12 | TCCTGAAATAAATAAA | 11 | GAATAAACACTTATTGATTGA |
| 12 | TGATAATAATGATAA | 11 | AGAGAG |
| 12 | AAAAGAAAG | 11 | AAAAAATAC |
| 12 | AAAATGTATAAAT | 11 | TGACAACTGACAATTG |
| 12 | TTCCAAATATCGACCATAATG | 11 | CATTCCTT |
| 12 | CATCATCGTCATCTT | 11 | TCTTTCTGTTCTACTGGCTCT |
| 12 | TCCTCTTCTTCGCAGCCCGCCTGG | 11 | ACGTCCAATTG |
| 12 | ATTTCATTTCAGAAATT | 11 | TTTTTTTTGTT |
| 12 | ACTGTGAACAAATTTCGTGAA | 11 | AGTTGTCGGCTCCGT |
| 12 | TCATGCATTCGAAG | 11 | GATGTCCAATA |
| 12 | CATTTTATATTTATA | 11 | AAAGAAGAAGAAAAAA |
| 12 | CCTAACCTAACTTAAT | 11 | GTGGGATTGGTGAAATCAGAA |
| 12 | AGAATATTCCATGAATATCAT | 11 | GTGACAAGTGTCAATA |
| 12 | AATAAAATAAA | 11 | AGAAACCATC |
| 12 | ACCCGGAATAATCCGAAGG | 11 | GCAGTTGACATGTGACATTTG |
| 12 | GTTAGGTTAGGTTAAA | 11 | ATGACGATGAAT |
| 12 | GATTATTCCGGGCCTCCG | 11 | GATGACGATGATGAG |
| 12 | CAAAGAATTT | 11 | AGAAAAAGAAAAAGAAGAAGA |
| 12 | CCGGATTATTCCAGGTCCT | 11 | TGTTCCCAAATTATCCTG |
| 12 | TATTAGAA | 11 | CATTTGACGTAACAACGCGA |
| 12 | ATTTTTGGC | 11 | TGAGTGAGTAAG |
| 12 | GATCATAATGT | 11 | GGGGACCCAGAATAATCCA |
| 12 | AAATAAAATAAA | 11 | TGTCACTTGTCAAA |
| 12 | AACGATAGAATAAACA | 11 | TGTCAGTCATC |
| 12 | ATATTCGGCAAAAGATCCGA | 11 | TAAATTCTCA |

| 11 | TTTATTGGACA | 10 | ATAAGAAA |
| --- | --- | --- | --- |
| 11 | CTTCTTCATTCTTCTC | 10 | ATGAATTGA |
| 11 | CATTAGACAGTCTGACAGGTGA | 10 | ATAAACAAATAA |
| 11 | CCGGAATAATCCGGGGGAA | 10 | CCCAAGAAGT |
| 11 | TAATGAAA | 10 | GGATTATTCCGGATCCTCC |
| 11 | GGATTATTTCGGGTCCTCC | 10 | ATTGGACAACTCCCAGTTCGA |
| 11 | GCACTGAGATGACACCTCTGG | 10 | AATATCCAG |
| 11 | AAAAAAAATTCCTGAAA | 10 | TTCAATTTCA |
| 11 | AAACGACCAGACTTCCATAAAAATTAATTAA | 10 | TTGGAACTGTCAAAGTCACTGTACCGTTT |
| 11 | CAATGAATGAATAAAT | 10 | CCGGAATGAT |
| 11 | ACGTATT | 10 | GGATTATTCCGGGTCCTCT |
| 11 | TTTGTAACTC | 10 | CTCTCAGCCTGCCTGGTCCTCTTC |
| 11 | AGAAAAAAAAGAAGG | 10 | GAGGACCACCTG |
| 11 | TAGGTTATGTTAGGT | 10 | CTGAAAAAAAAATTC |
| 11 | TTCTATA | 10 | ATAGTCTCTG |
| 11 | AAGTTA | 10 | TATTCCATA |
| 11 | CCAGTGCCCCTATGATGTCATC | 10 | CATTTTTGACATGTGA |
| 11 | ATTTTGAC | 10 | TTTTTTGAAGAG |
| 11 | TTTATGGAAGTCTGGTCGATTTGAAT | 10 | TTGAATTGATTGA |
| 11 | GAAGAAGGAC | 10 | GGTCCACTTCCTCTCAGCCTGCTC |
| 11 | AAATAAATAC | 10 | TTAGGTTAGG |
| 11 | GTCCCGACGGACGTCCATCAAAT | 10 | GAATAATCCGGGAGAAACCA |
| 11 | AAGAAGACGAAAAGAC | 10 | TTTTCAACGTACACAGTCACGTC |
| 11 | ATAAAAAAAT | 10 | TTATGACGTCATTACTGTTTGAA |
| 11 | TGACAAGTGACATTGACATT | 10 | TGTTGCTGTTGTGGATGTCCC |
| 11 | TTTTCGACGTTTCGTCGTTTGACGCTTGAAG | 10 | GAGTAAGAGTAAC |
| 11 | ACAACGTGA | 10 | TATCCAAC |
| 11 | TAGCCATTATGGTCCA | 10 | GTGTTTGGAGGGCTTGTTCTGAG |
| 11 | AAAATCAATTAAAAACGACCAGATTTCCATA | 10 | TGGAAGTCTGGTCATTATTAAAAATTTTTTA |
| 11 | AGAGCCTTAAA | 10 | ACAAAGAATATCTGGTGAAGCTGAGTTCA |
| 11 | CTTTCGTT | 10 | AAGCAACAAAAAGTGTG |
| 11 | GACGTCAAACGGACATCCTGTGGATATCGTATG | 10 | CAGCAGCAA |
| 11 | CATTTTTTG | 10 | CAAGAAACATCAAGACAT |
| 11 | TTTTTTTGAGAATCC | 10 | TATGGGCCATAATGGC |
| 11 | CGGGAGCCCGGAATAATC | 10 | AAAGAGAAATGGCAGATGCC |
| 10 | AAAATCAATGACAGATGACAGCTGAC | 10 | ATTTCAGGAATCAATTC |
| 10 | AAGCTGTATTGAGA | 10 | ATAATCCAGAGACCCGAA |
| 10 | CAGTTGT | 10 | AGGAGAAGGAGAAG |
| 10 | AAAAAAAAGAAAAAG | 10 | TCATTTATTATTCAT |
| 10 | AAGAAAGAAGAAA | 10 | TTAACA |
| 10 | AATATTGTA | 10 | TTCTCTTTTC |
| 10 | AGGGAGAG | 10 | CCATTG |
[truncated: 587,069 more chars]
